# Supplementary material for: Modern groundwater reaches deeper depths in heavily pumped aquifer systems
Source: Nat Commun. 2022 Sep 7;13:5263. doi: 10.1038/s41467-022-32954-1 (PMC9452510; doi:10.1038/s41467-022-32954-1)
Supplement: Supplementary file 1 — Supplementary Information [file 41467_2022_32954_MOESM1_ESM.pdf]

Supplementary Information

Title: Modern groundwater reaches deeper depths in heavily pumped aquifer systems

Thaw, M. [a,*], GebreEgziabher, M. [a], Villafañe-Pagán, J.Y. [a,b], Jasechko, S. [a,*]
[a] Bren School of Environmental Science and Management, University of California, Santa Barbara, California, USA
[b] Department of Geology, University of Puerto Rico, Mayagüez, 00682, Puerto Rico
[*] authors contributed equally

Table of contents

Supplementary Note 1. Well depth versus modern groundwater plots for study aquifers 4

Supplementary Note 2. Estimating groundwater withdrawals within each study area 97

Supplementary Note 3. Regional hydrogeology and hydrostratigraphy of study aquifer systems 109

3.1 Sacramento Basin, California Central Valley 129

3.2 San Joaquin Basin, California Central Valley 132

3.3 Tulare Basin, California Central Valley 135

3.4 Eastern Carrizo-Wilcox, Carrizo-Wilcox 138

3.5 Eagle Valley, Carson River Basin 141

3.6 Central Wabash and Bloomington Ridged Plain, Central Lowland Till Plain 143

3.7 Palouse Slope, Columbia Plateau Regional Aquifer System 146

3.8 Umatilla Basin and Horse Heaven Hills, Columbia Plateau Regional Aquifer System 148

3.9 Yakima Basin, Columbia Plateau Regional Aquifer System 151

3.10 Stockton Plateau, Edwards-Trinity Aquifer System 153

3.11 Trinity Aquifer System, Edwards-Trinity Aquifer System 155

3.12 Dougherty Plain and Marianna Lowlands, Floridan Aquifer System 158

3.13 Eastern Flatwoods Southshores, Floridan Aquifer System 161

3.14 Lower Coastal Plain, Floridan Aquifer System 164

3.15 Ocala Uplift, Floridan Aquifer System 167

3.16 Sea Island, Floridan Aquifer System 170

3.17 Tifton Upland, Floridan Aquifer System 174

3.18 Vidalia Upland, Floridan Aquifer System 177

3.19 Catahoula Area, Gulf Coast Regional Aquifer System 180

3.20 Houston-Galveston Area, Gulf Coast Regional Aquifer System 183

3.21 Lafayette Area, Gulf Coast Regional Aquifer System 186

3.22 Southern Hills, Gulf Coast Regional Aquifer System 189
3.23 Central High Plains, High Plains 191
3.24 Northern High Plains, High Plains 194
3.25 Southern High Plains, High Plains 197
3.26 Albuquerque Basin, Middle Rio Grande 200
3.27 San Luis Valley, Middle Rio Grande 204
3.28 Central Mississippi Embayment, Mississippi Embayment 206
3.29 Eastern Mississippi Embayment, Mississippi Embayment 209
3.30 Delmarva Peninsula, North Atlantic Coastal Plain 212
3.31 Maryland Western Shores, North Atlantic Coastal Plain 217
3.32 New Jersey Coastal Plain, North Atlantic Coastal Plain 220
3.33 North Carolina and Virginia Coastal Plain, North Atlantic Coastal Plain 224
3.34 Powder River Basin, Northern Great Plains 228
3.35 Williston Basin, Northern Great Plains 231
3.36 Eastern Silurian-Devonian Aquifers, Northern Midwest Aquifer System 234
3.37 Mississippian-Silurian-Devonian Carbonates, Northern Midwest Aquifer System 237
3.38 Northeast Missouri Carbonates, Northern Midwest Aquifer System 243
3.39 Northern Cambrian-Ordovician Aquifers, Northern Midwest Aquifer System 247
3.40 Upper Carbonate Aquifer, Northern Midwest Aquifer System 249
3.41 Western Cambrian-Ordovician Aquifers, Northern Midwest Aquifer System 251
3.42 Mesilla Valley, Rincon-Mesilla Valleys 254
3.43 Lower Santa Ynez Valley, Santa Ynez Valley 257
3.44 Boise Valley and Homedale Murphy Area, Western Snake River Plain 259
3.45 Mountain Home Plateau, Western Snake River Plain 261
3.46 Antelope Valley 263
3.47 Big Bear Valley 265
3.48 Bighorn Basin 267
3.49 Black Hills Uplift 269
3.50 Black Warrior River Aquifer System (Eutaw and McShan Formations and Tuscaloosa Group) 272
3.51 Castle Hayne Aquifer 275
3.52 Coachella Valley 279
3.53 Cuyama Valley 281
3.54 Denver Basin 283

3.55 Eastern Dakota Aquifer ... *285*

3.56 Eureka and Eel River and Mad River Plains ... *288*

3.57 Garber-Wellington Aquifer ... *290*

3.58 Honey Lake Valley ... *292*

3.59 Long Island ... *294*

3.60 Los Angeles Basin ... *297*

3.61 Michigan Basin ... *300*

3.62 Mojave Basin ... *303*

3.63 Northern Green River Basin ... *305*

3.64 Ozark Plateaus Aquifer System ... *308*

3.65 Pearl and Chattahoochee Aquifer System ... *311*

3.66 Salinas Valley ... *314*

3.67 Salt Lake Valley ... *316*

3.68 San Pedro Basin ... *318*

3.69 Santa Clara-Calleguas Basin ... *320*

3.70 Santa Rosa Valley ... *323*

3.71 South Park Basin ... *326*

3.72 Tijuana-San Diego Basin ... *329*

3.73 Upper Santa Ana Basin ... *332*

3.74 Utah Lake Valley ... *334*

Supplementary Note 4. Multiple rank correlations to account for interrelationships among our two potential explanatory variables ... 336

Supplementary Note 5. Rank correlations between groundwater withdrawals and the depth below which modern groundwater is scarce if we exclude aquifer systems with shallow depths to confined conditions ... 339

Supplementary Note 6. Potential explanations for deep modern groundwater ... 340

Supplementary Note 7. Modern groundwater prevalence in wells defined by the USGS as tapping a confined aquifer ... 345

Supplementary Note 8. Locations of hydrogeologic cross sections ... 347

Supplementary Note 1. Well depth versus modern groundwater plots for study aquifers

The following Supplementary Figs. 1-91 provide a set of plots showing how modern groundwater varies with well depth; each plot represents a single aquifer system (i.e., all groundwater tritium measurements collected from within the boundaries of a given aquifer system).

The title of the aquifer system is identified in the figure caption. Each yellow circle represents the calculated maximum fraction of the well water sample comprised of modern water (yellow circles correspond to the x-axis labels on the bottom of the plot).

The blue line presents the proportion of all groundwater samples collected from wells deeper than a given depth that contain minimal (<25%) modern water; we only present a blue line if at least five modern groundwater fraction data points are available for deeper wells (i.e., deeper than any given well).

The three diamonds represent the depths at which one of the following criteria are met:

- **Red diamond** – the depth below which >60% of well water samples contain less than 25% modern water

- **Purple Diamond** – the depth below which >70% of well water samples contain less than 25% modern water

- **Green Diamond** – the depth below which >80% of well water samples contain less than 25% modern water

The y-axis on each plot extends to a depth of 1,000 m below land surface; however, a small number of groundwater ^{3}H samples were collected from wells with depths exceeding 1,000 m, and are thus not plotted in Supplementary Figs. 8-98. There are only n=5 samples in our dataset collected from wells with depths exceeding 1,000 m (representing 0.05% of all of the n=9,333 groundwater tritium samples plotted in Supplementary Figs. 8-98). These n=5 samples are located in the Yakima Basin (n=1 sample from a well with a depth exceeding 1,000 m), the Western Carrizo-Wilcox (n=1 sample), the Lower Coastal Plain (n=1 sample) and the Ozark Plateaus Aquifer System (n=2 samples).

In most, but not all, of our aquifer systems, our method that identifies the depths below which over 60%, 70% or 80% of deeper wells contain minimal modern groundwater (<25%) appears to adequately capture the depth below which modern groundwaters become scarce (see diamonds in Supplementary Figs. 1-91). However, by plotting all n=91 study aquifer systems, we identified some cases where our method does not adequately capture the depth below which modern groundwaters become scarce for at least two broad reasons: (a) Well water tritium data are limited or absent for substantial well depth intervals, or (b) modern groundwater fractions decline to a certain depth, but increase at deeper depths.

These n=17 aquifer systems (where our method was deemed imperfect following visual inspection) are listed in Supplementary Table 1. We excluded these aquifer systems from further analyses and from the plots and correlations presented within the main text.

Supplementary Table 1. Aquifers where the depth below which over 60%, 70% or 80% of deeper wells contain minimal modern groundwater (<25%) appears an imperfect estimate of the depth below which modern groundwaters become scarce

Example	Aquifer System	Supplementary Fig.
(a) Well water tritium data are limited or absent for substantial well depth intervals (i.e., y-axis values in Supplementary Figs. 1-91)	Western Mississippi Embayment	37
	Valle de Juarez and Hueco Bolson	54
	Blue Mountains and Clearwater Embayment	9
	Espanola Basin	33
	Judith Basin	71
	Peedee and Black Creek and Cape Fear Aquifers	79
	San Antonio Creek Valley	82
	Southern San Juan Basin	87
	Bacon Terrace	16
	Central Carrizo-Wilcox	4
	Spanish Springs Valley	88
	Big Chino Valley	59
(b) Modern groundwater fractions decline to a certain depth, but increase at deeper depths	West Salt River Basin	52
	Eastern Cambrian-Ordovician Aquifers	44
	Intermediate Aquifer	19
	Western Carrizo-Wilcox	6
	Walla Walla Basin	12

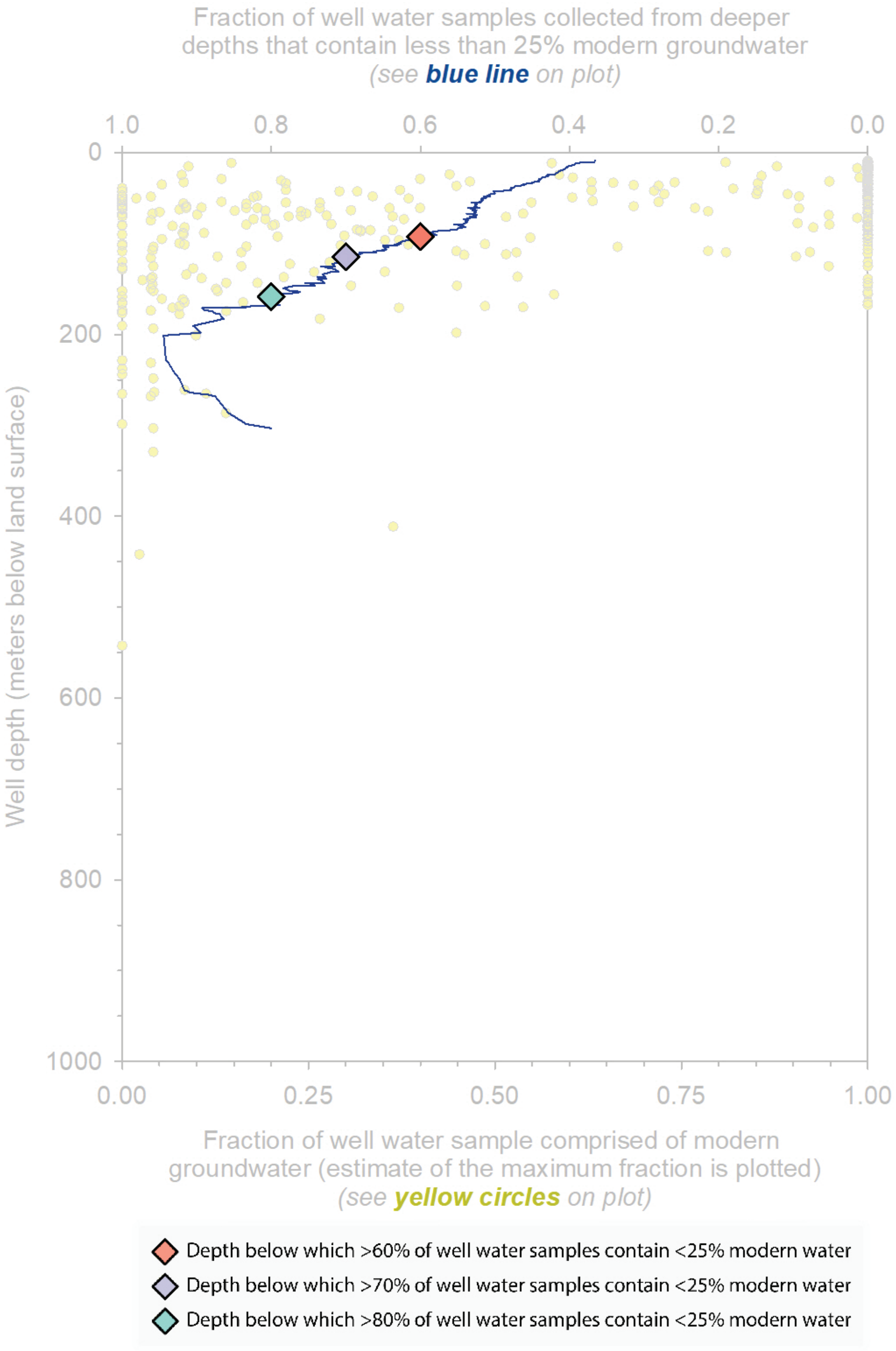

Supplementary Fig. 1. Sacramento Valley modern well water prevalence with depth. For details on symbology see the paragraph at the beginning of Supplementary Note 1.

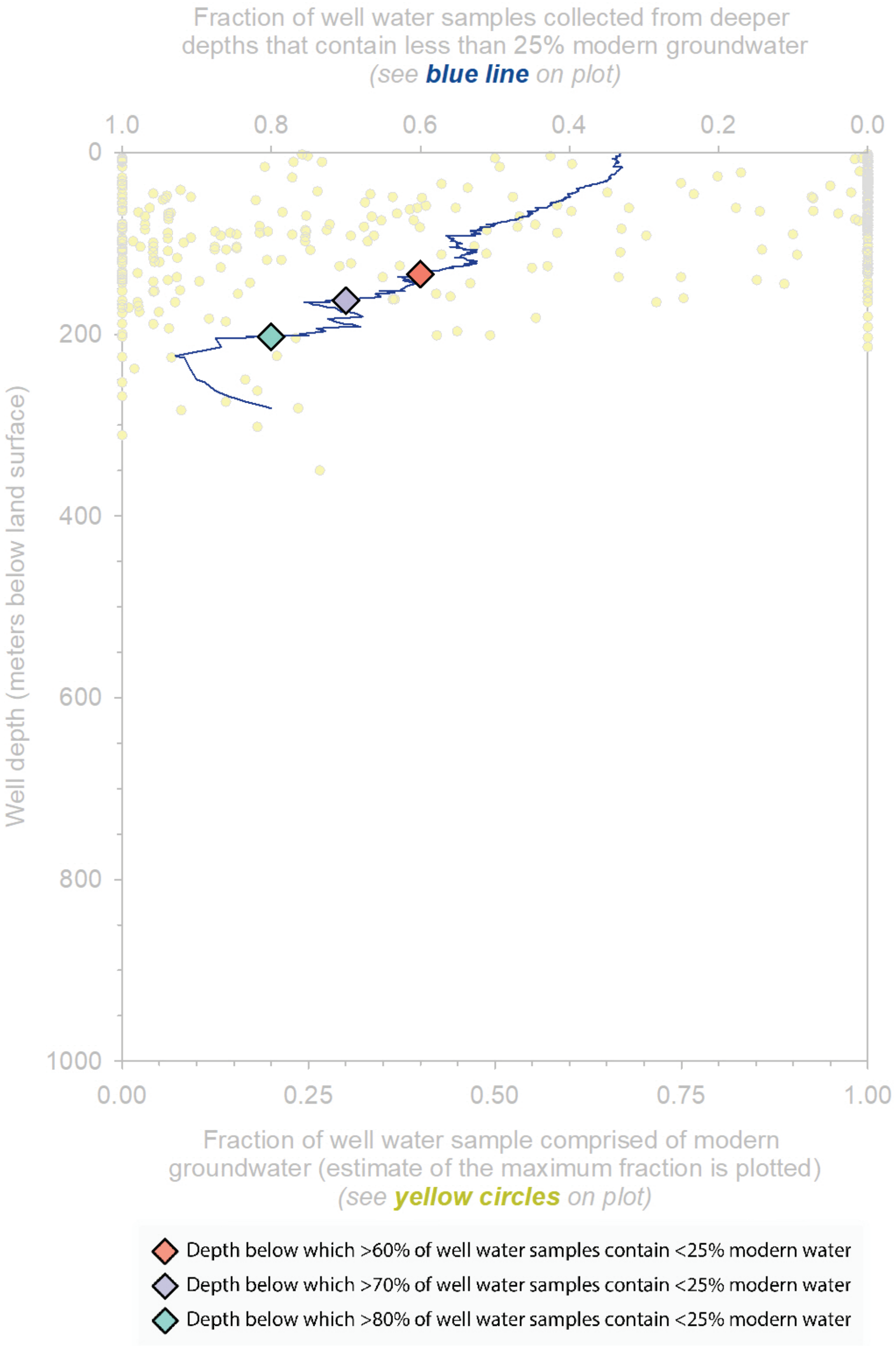

Supplementary Fig. 2. San Joaquin Basin modern well water prevalence with depth. For details on symbology see the paragraph at the beginning of Supplementary Note 1.

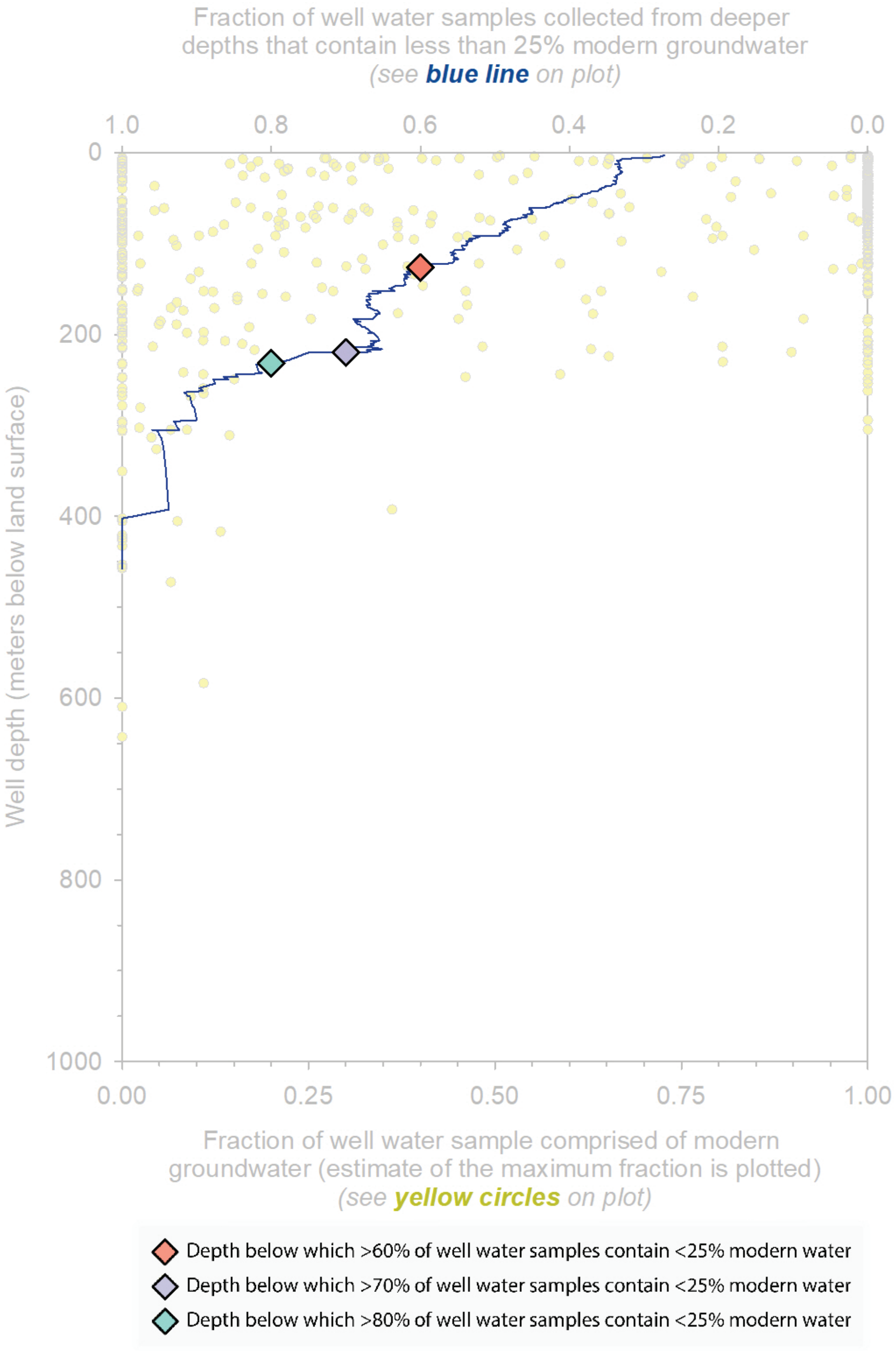

Supplementary Fig. 3. Tulare Basin modern well water prevalence with depth. For details on symbology see the paragraph at the beginning of Supplementary Note 1.

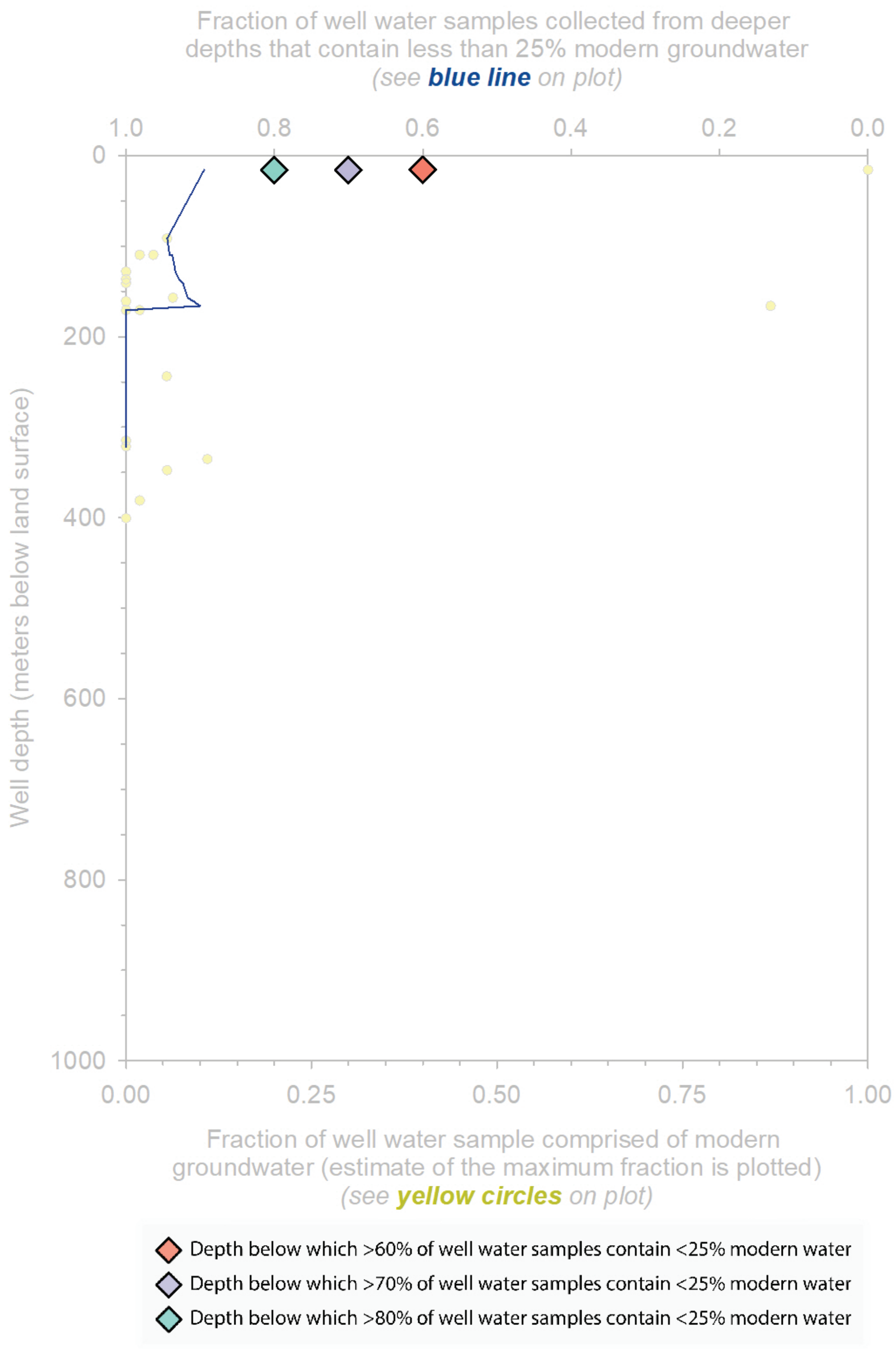

Supplementary Fig. 4. Central Carrizo-Wilcox modern well water prevalence with depth. For details on symbology see the paragraph at the beginning of Supplementary Note 1.

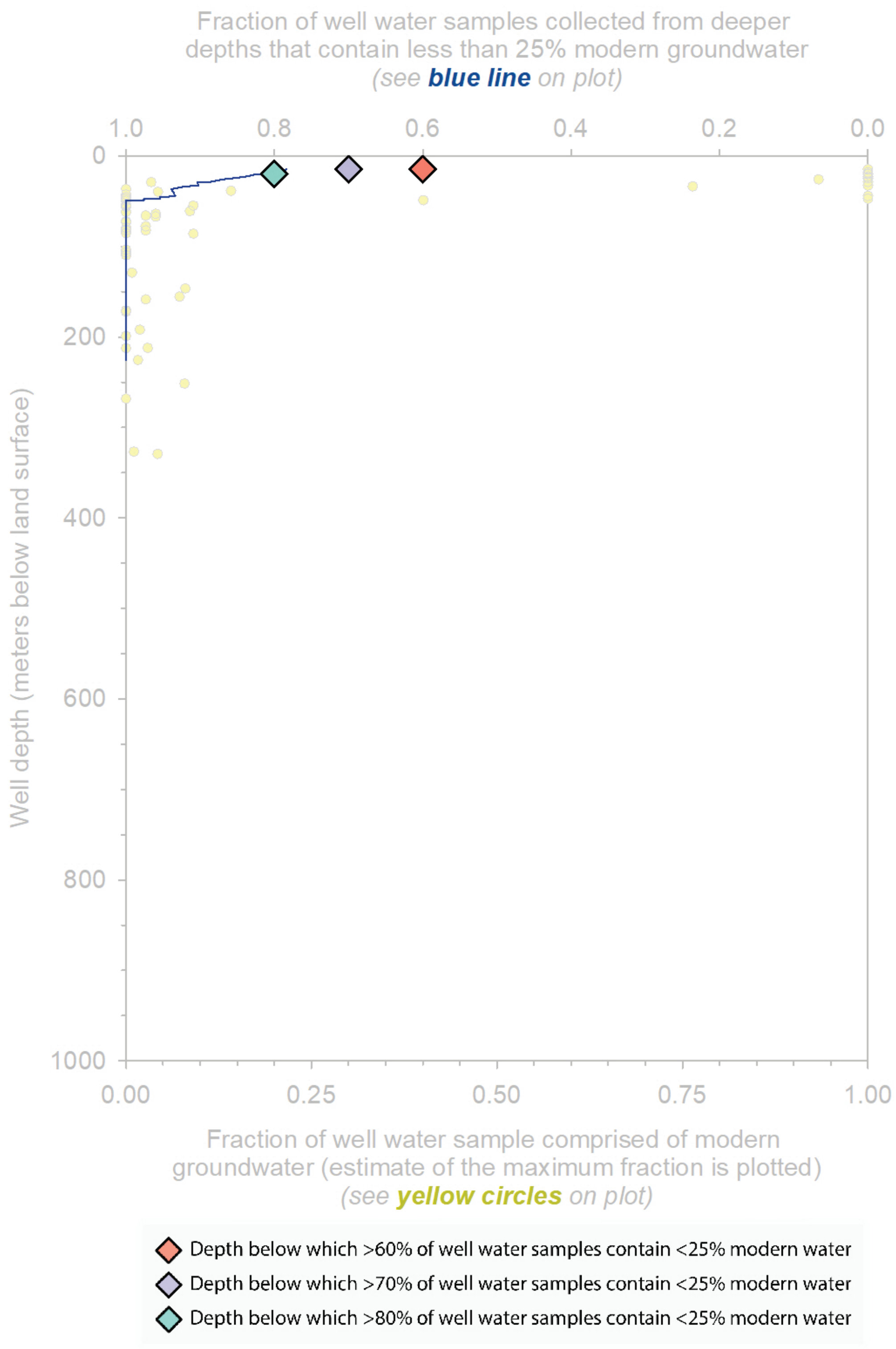

Supplementary Fig. 5. Eastern Carrizo-Wilcox modern well water prevalence with depth. For details on symbology see the paragraph at the beginning of Supplementary Note 1.

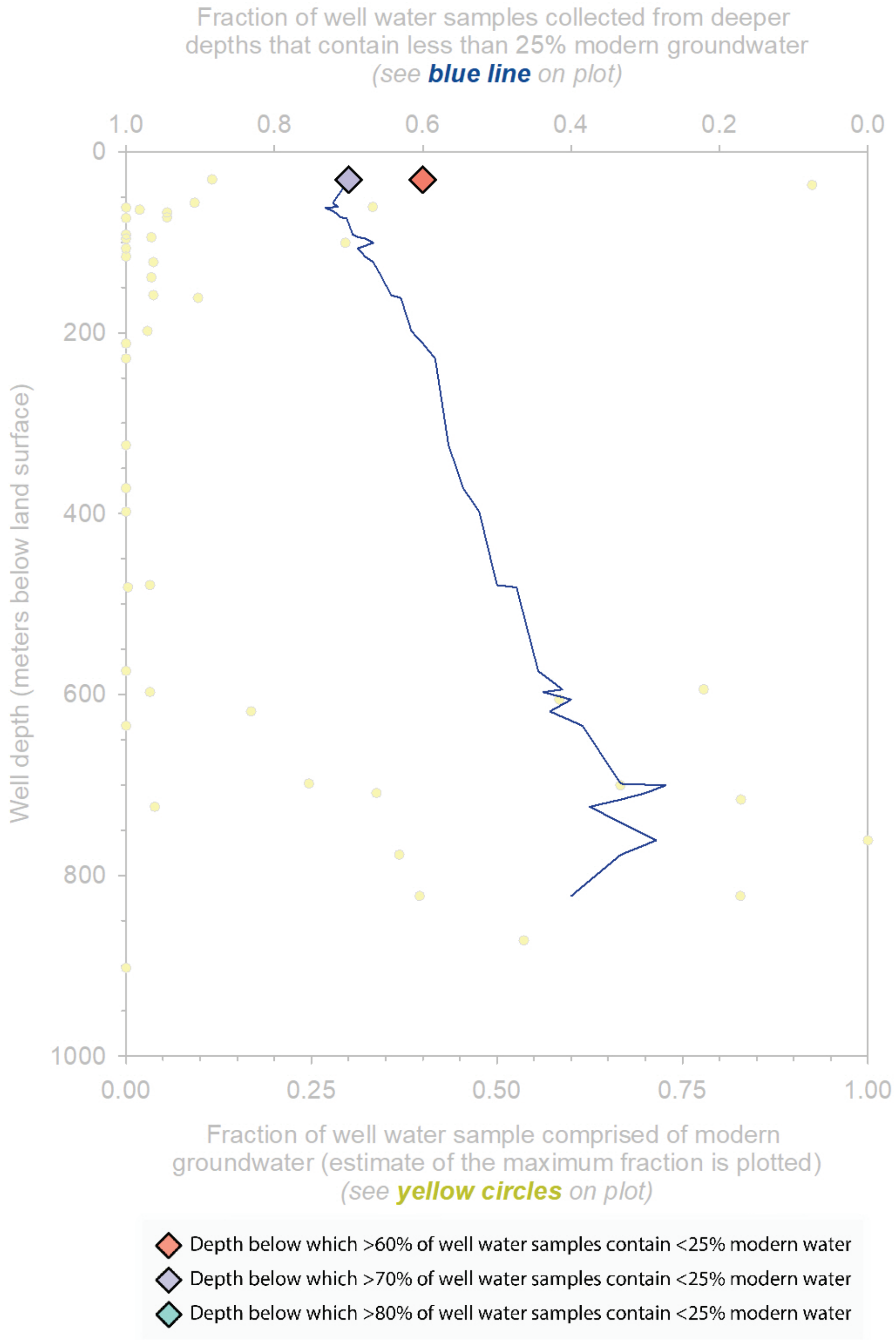

Supplementary Fig. 6. Western Carrizo-Wilcox modern well water prevalence with depth.
For details on symbology see the paragraph at the beginning of Supplementary Note 1.

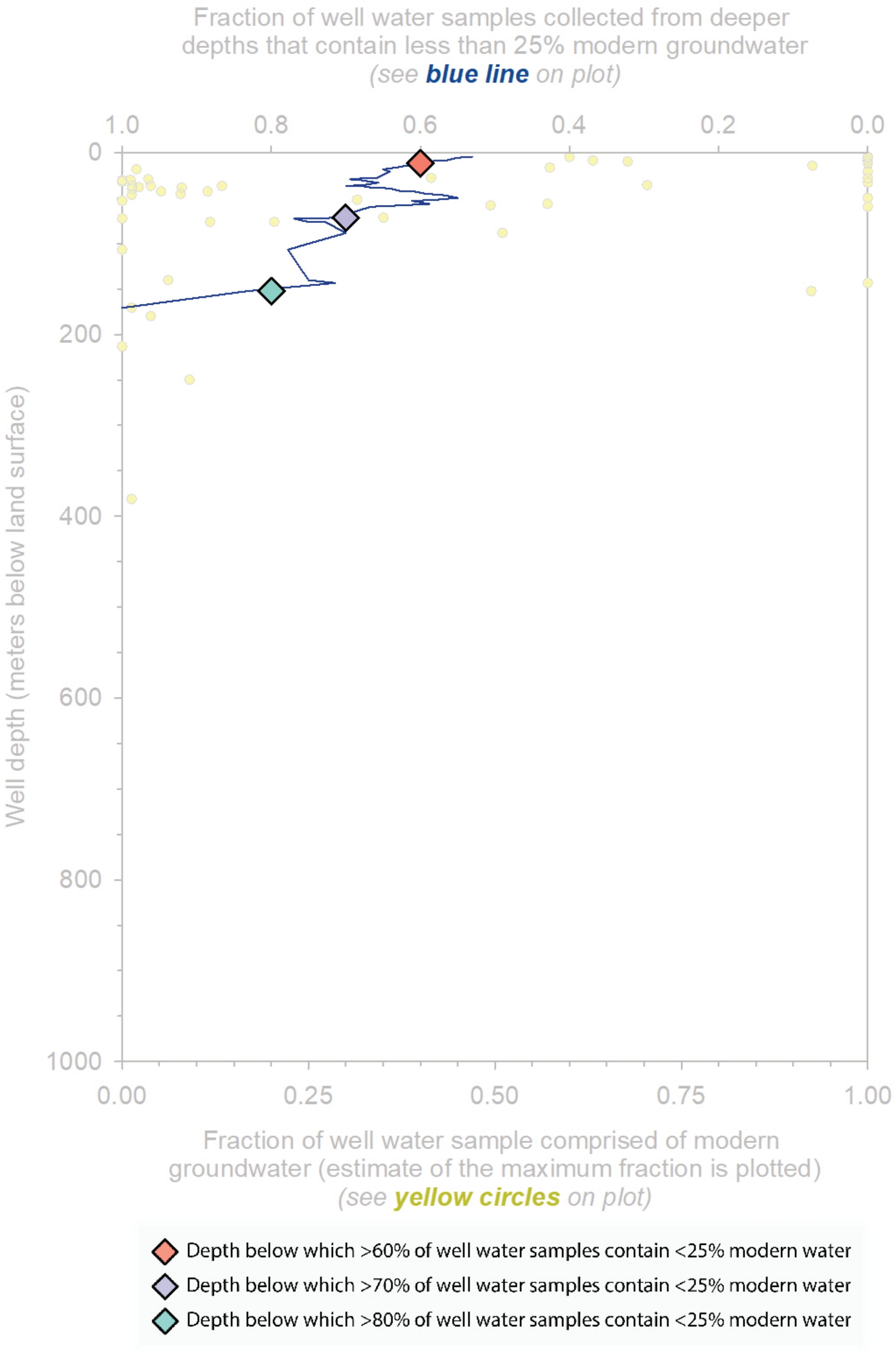

Supplementary Fig. 7. Eagle Valley modern well water prevalence with depth. For details on symbology see the paragraph at the beginning of Supplementary Note 1.

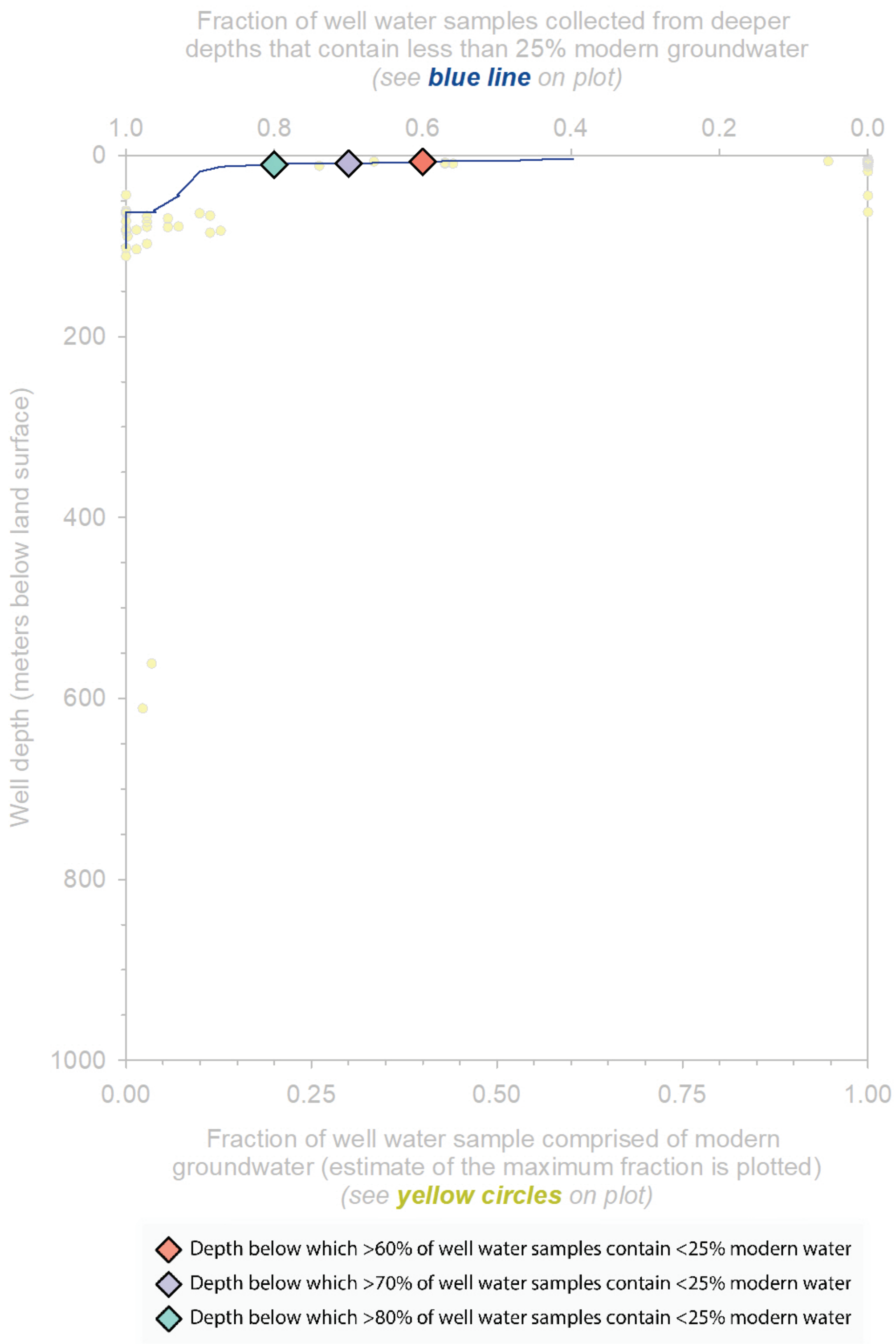

Supplementary Fig. 8. Central Wabash and Bloomington Ridged Plain modern well water prevalence with depth. For details on symbology see the paragraph at the beginning of Supplementary Note 1.

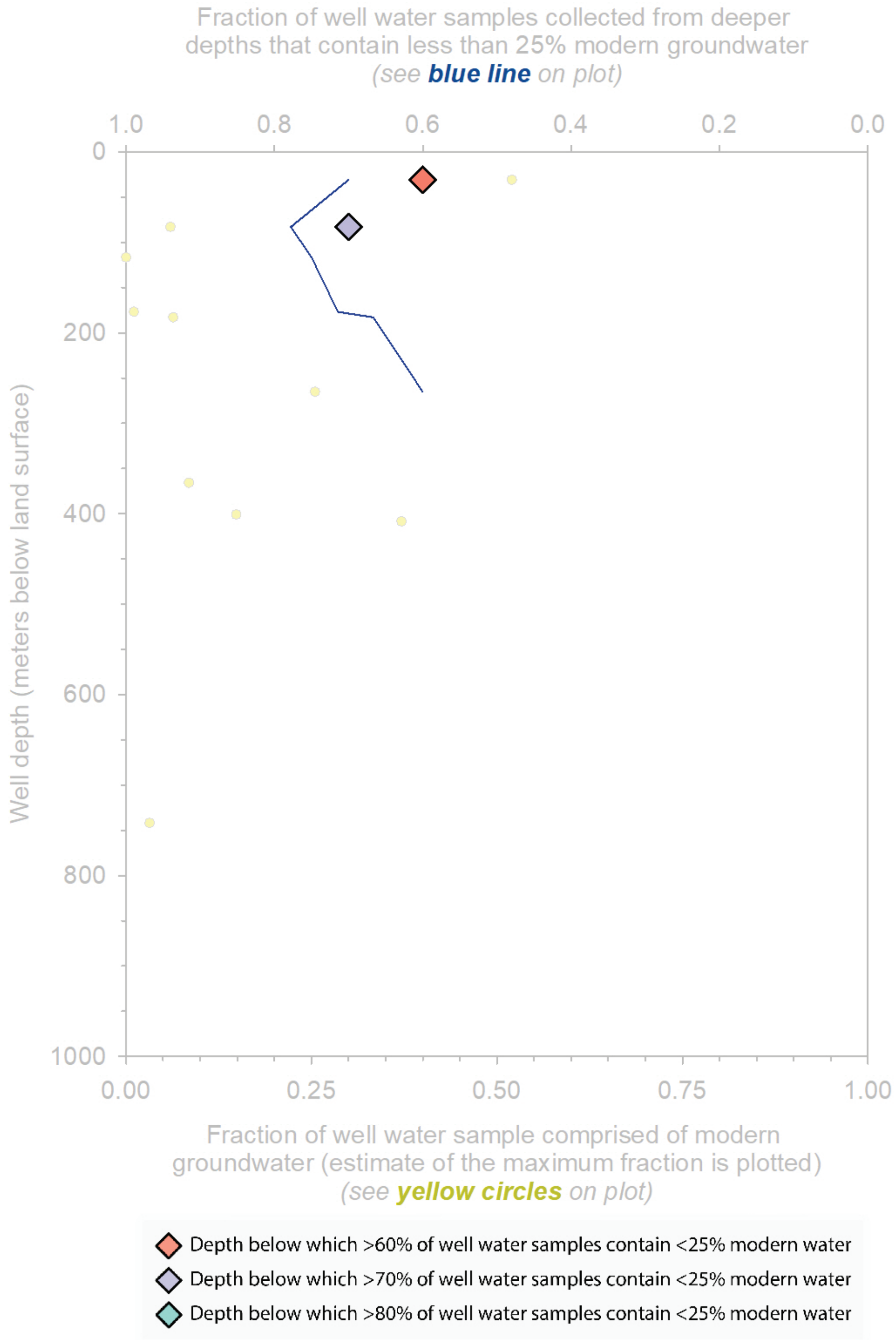

Supplementary Fig. 9. Blue Mountains and Clearwater Embayment modern well water prevalence with depth. For details on symbology see the paragraph at the beginning of Supplementary Note 1.

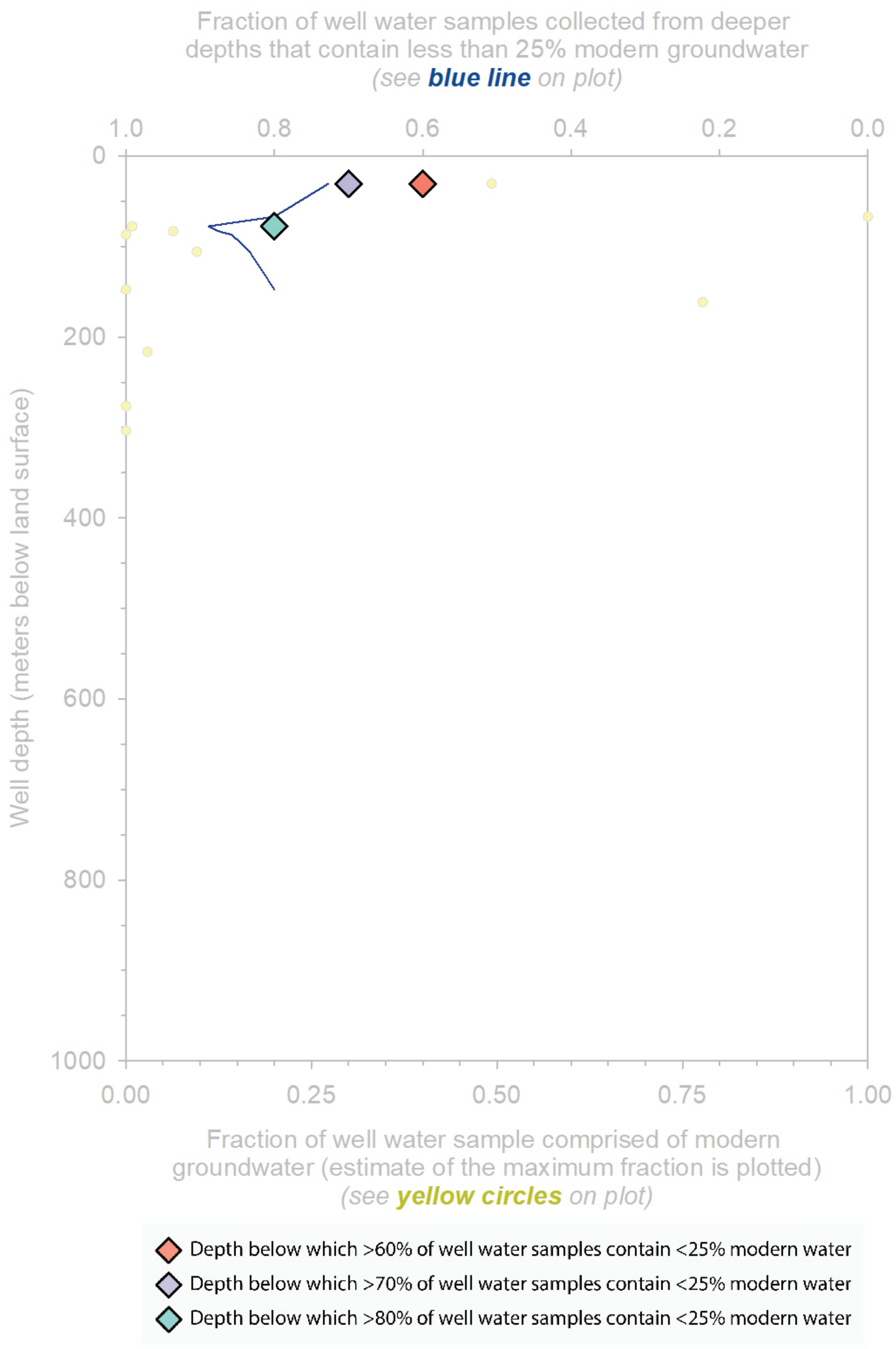

Supplementary Fig. 10. Palouse Slope modern well water prevalence with depth. For details on symbology see the paragraph at the beginning of Supplementary Note 1.

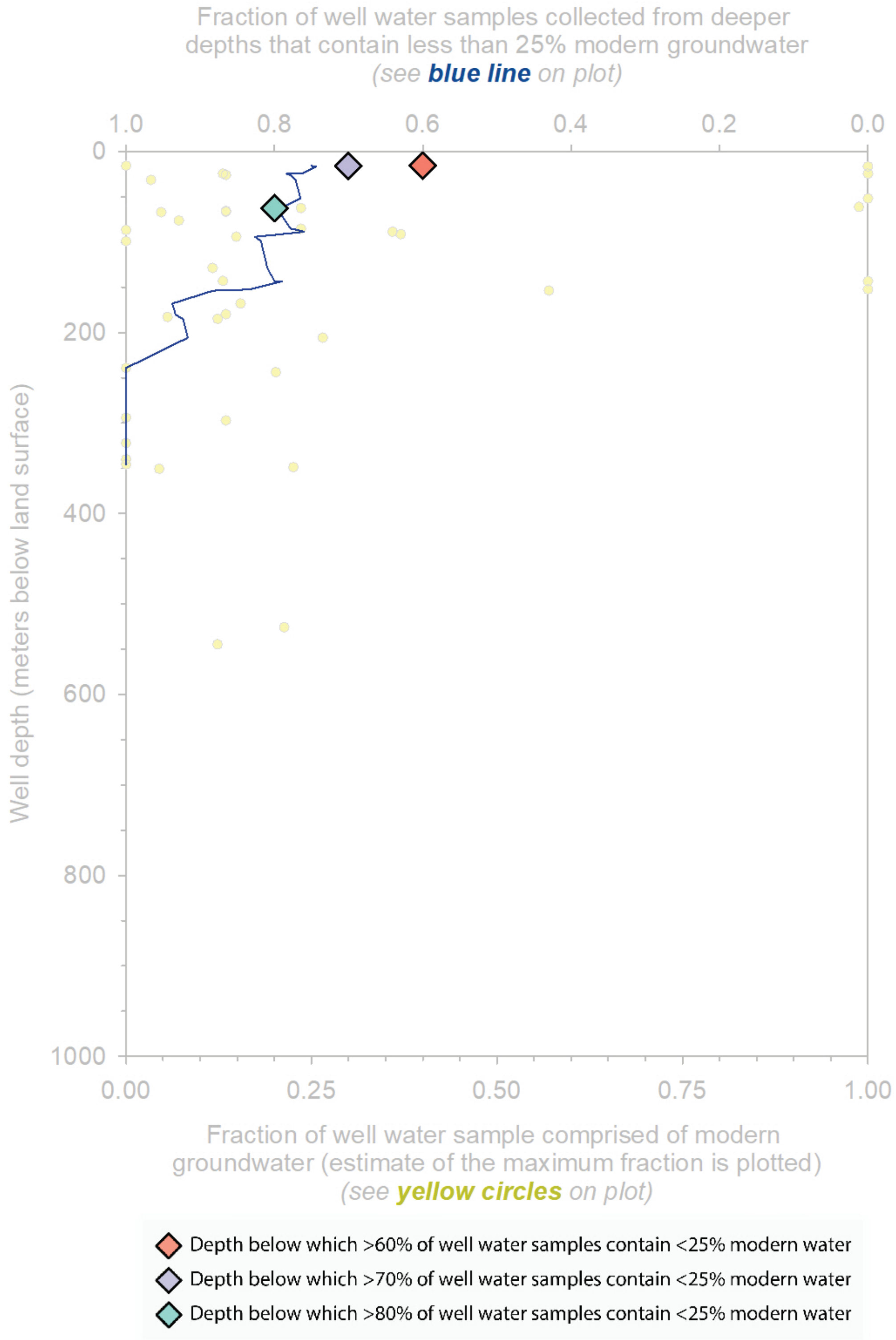

Supplementary Fig. 11. Umatilla Basin and Horse Heaven Hills modern well water prevalence with depth. For details on symbology see the paragraph at the beginning of Supplementary Note 1.

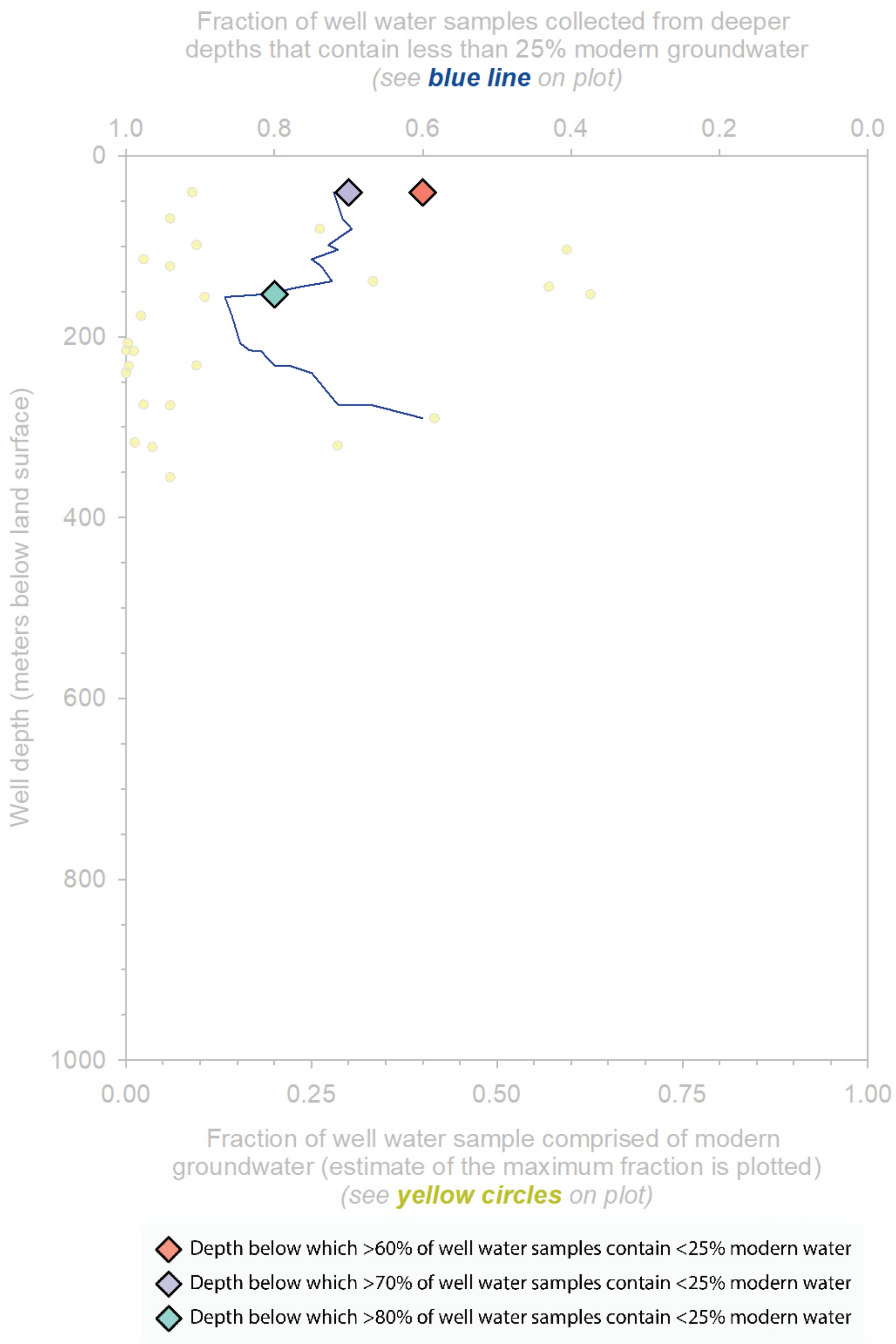

Supplementary Fig. 12. Walla Walla Basin modern well water prevalence with depth. For details on symbology see the paragraph at the beginning of Supplementary Note 1.

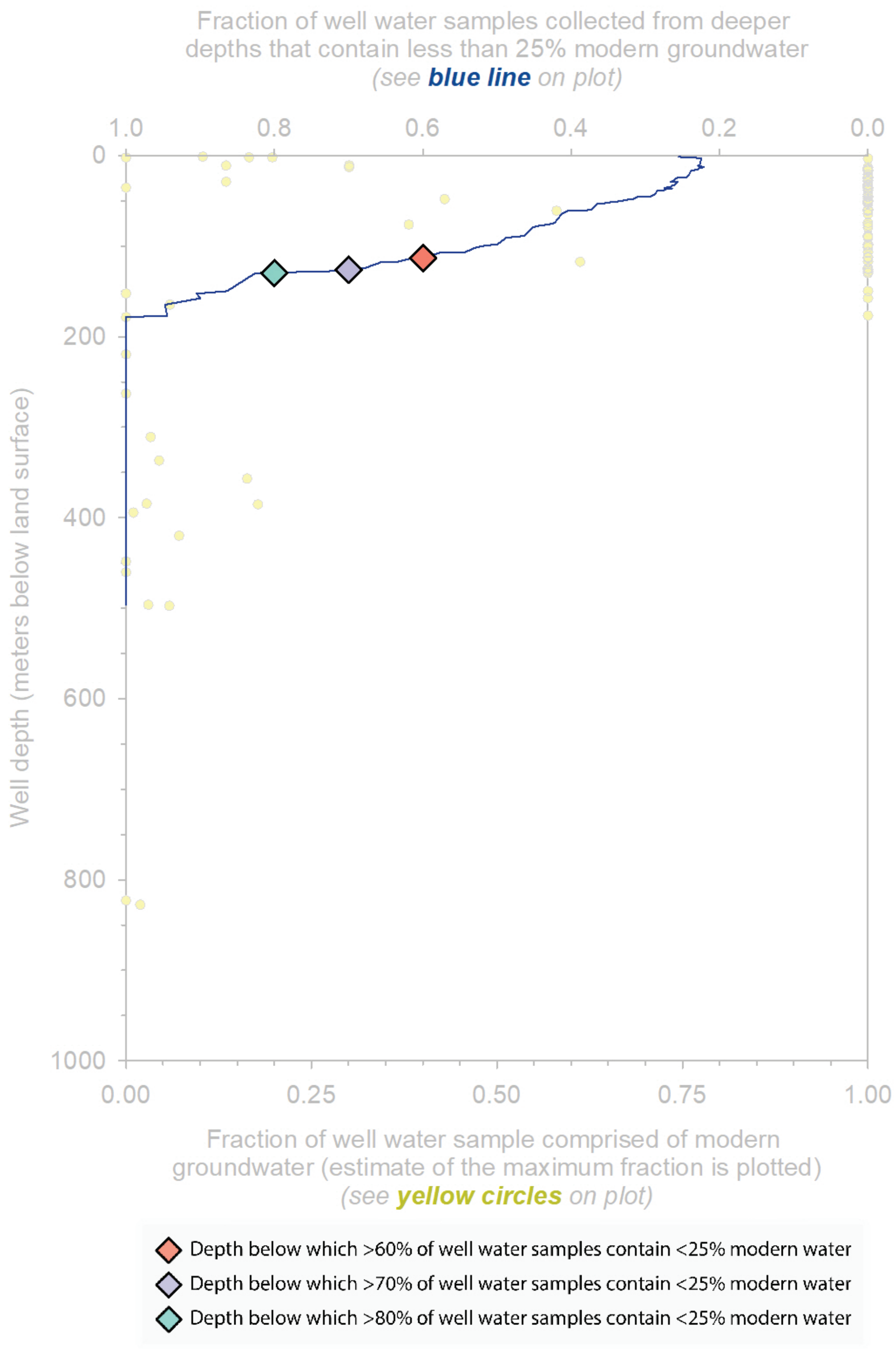

Supplementary Fig. 13. Yakima Basin modern well water prevalence with depth. For details on symbology see the paragraph at the beginning of Supplementary Note 1.

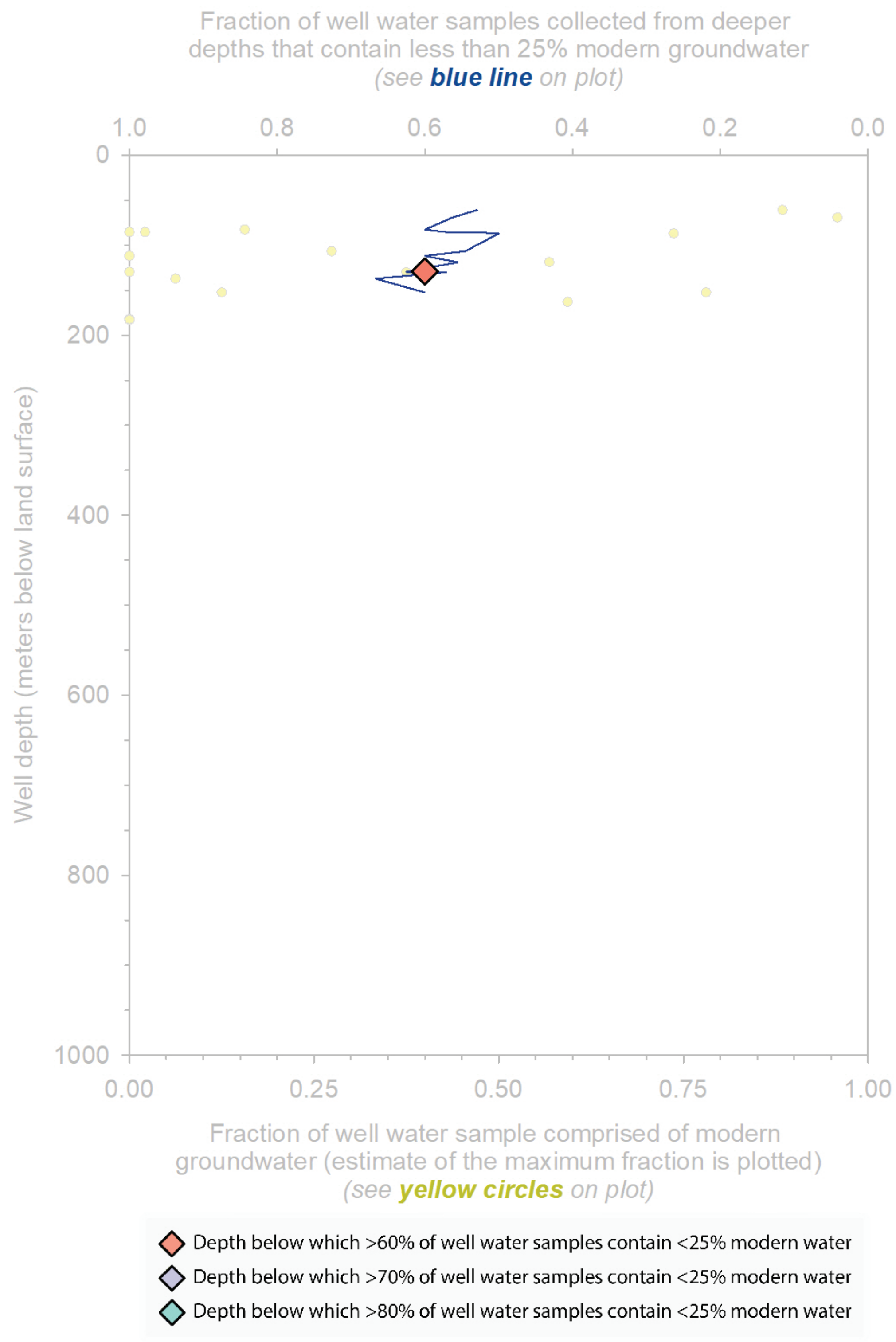

Supplementary Fig. 14. Stockton Plateau modern well water prevalence with depth. For details on symbology see the paragraph at the beginning of Supplementary Note 1.

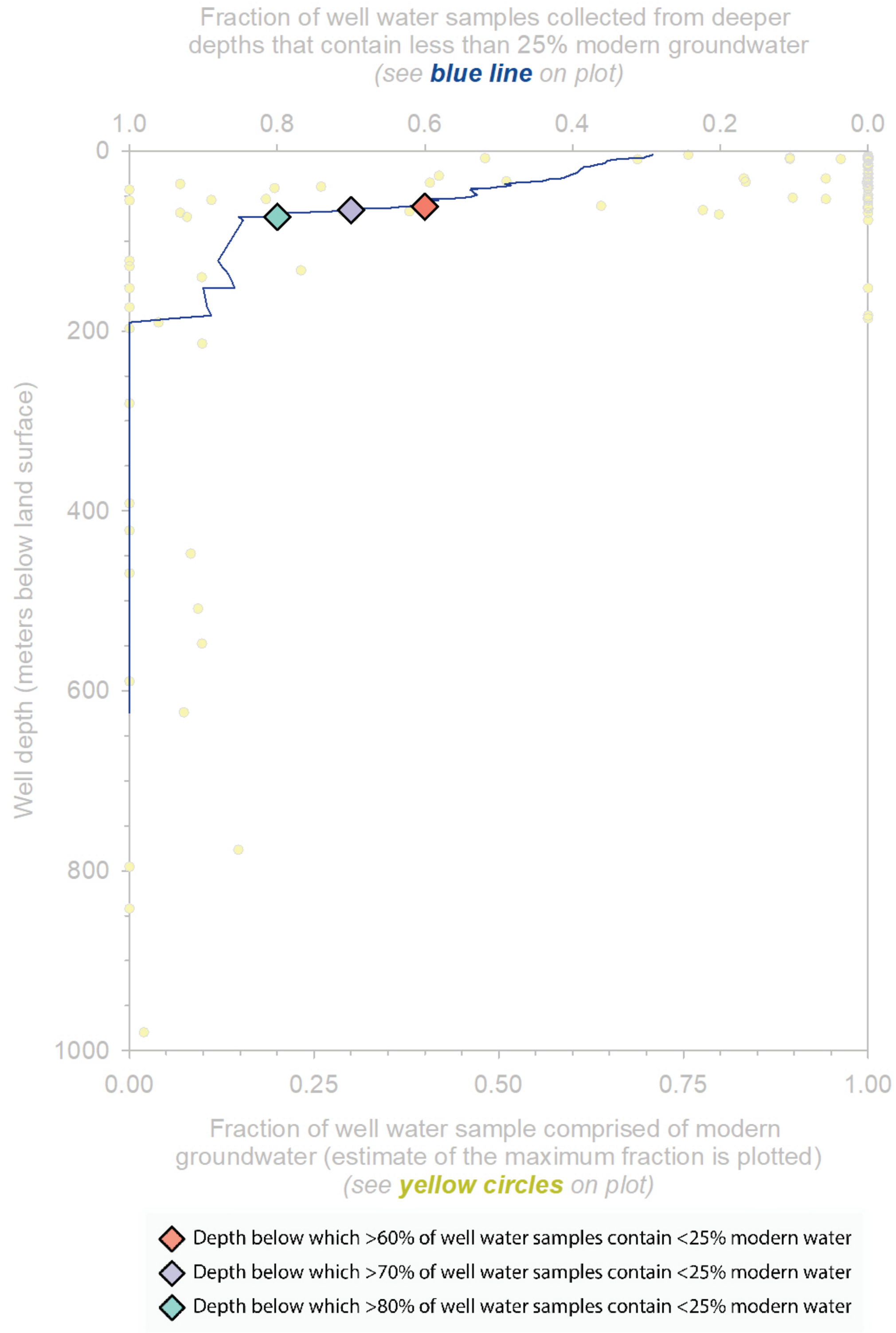

Supplementary Fig. 15. Trinity Aquifer System modern well water prevalence with depth. For details on symbology see the paragraph at the beginning of Supplementary Note 1.

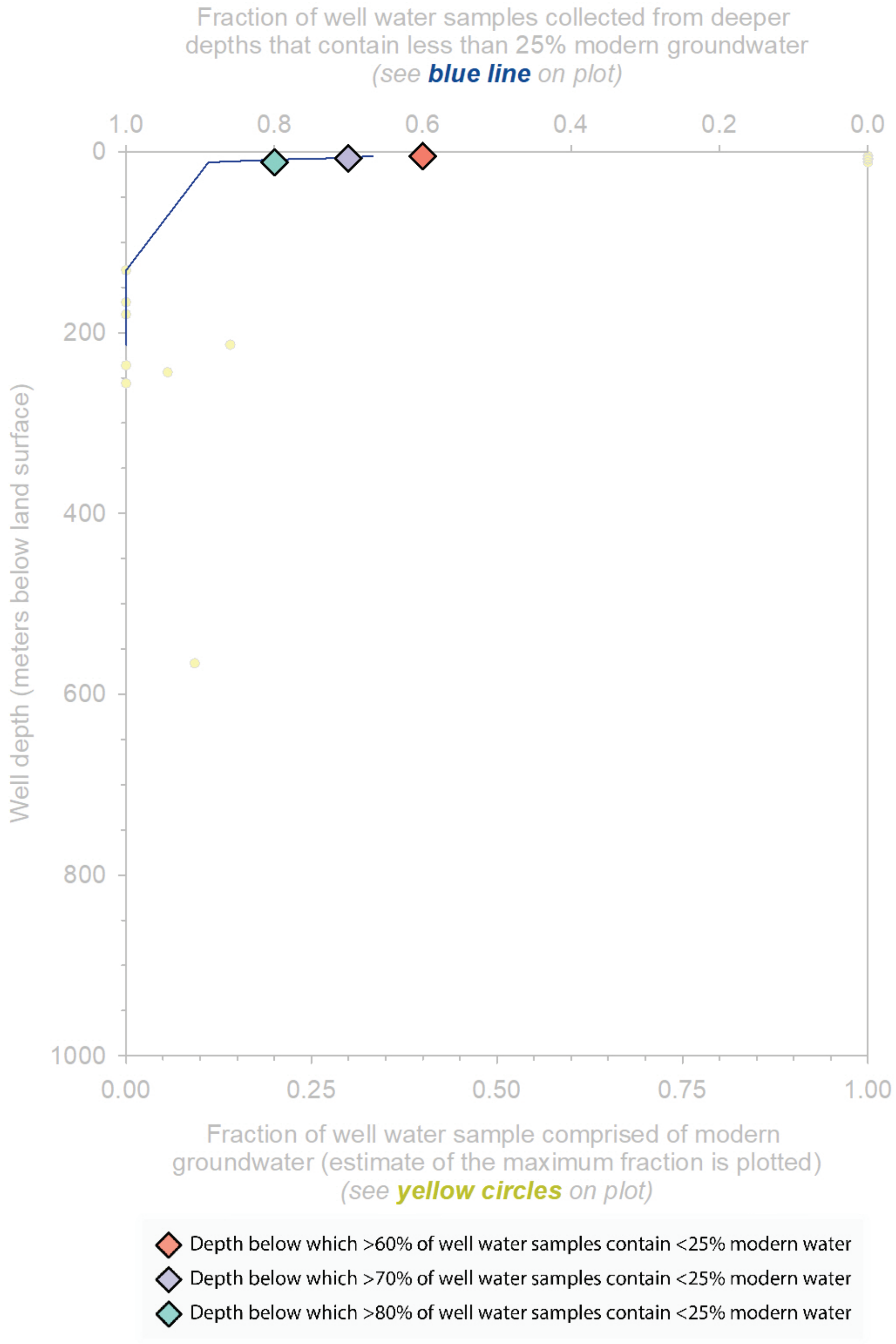

Supplementary Fig. 16. Bacon Terrace modern well water prevalence with depth. For details on symbology see the paragraph at the beginning of Supplementary Note 1.

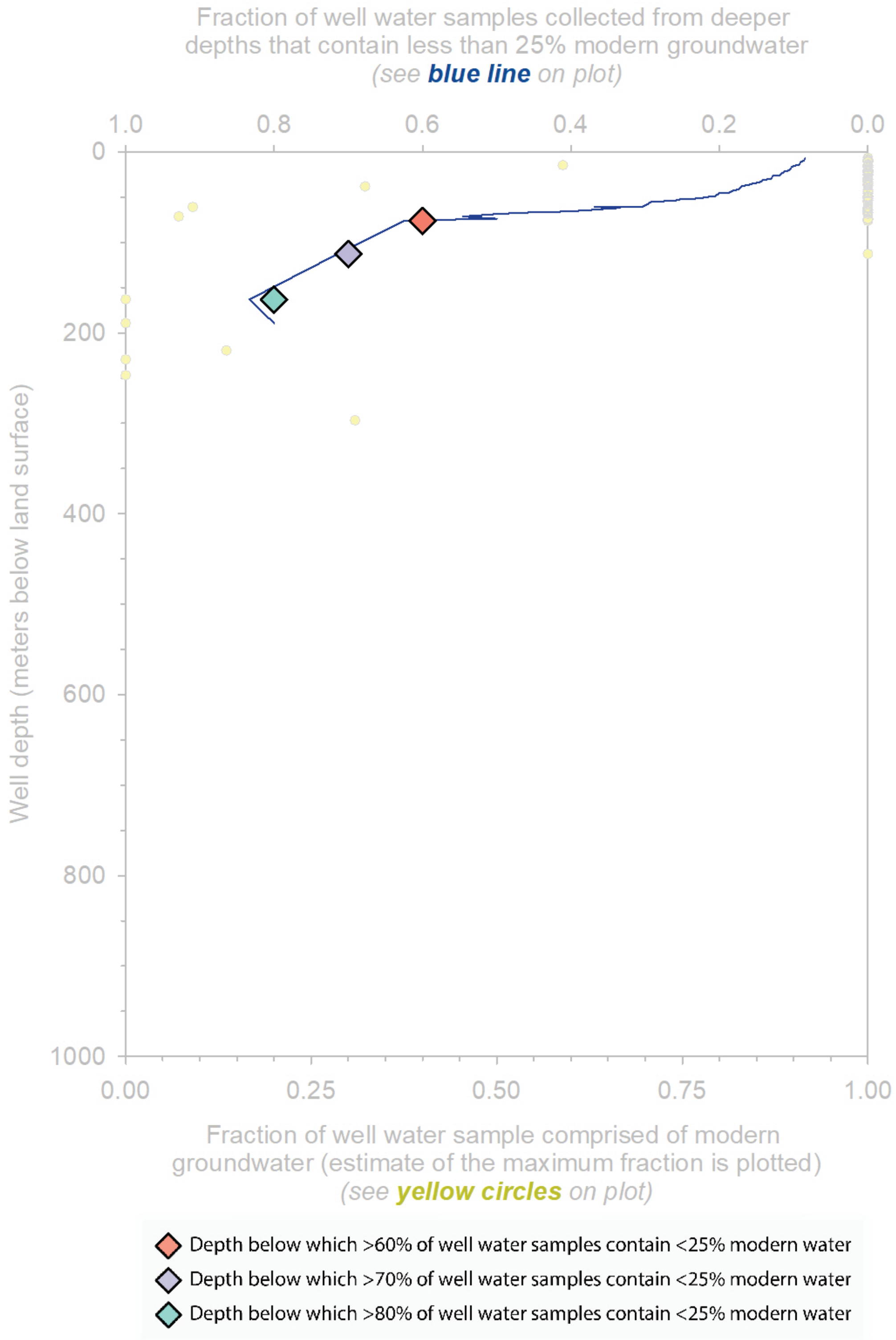

Supplementary Fig. 17. Dougherty Plain and Marianna Lowlands modern well water prevalence with depth. For details on symbology see the paragraph at the beginning of Supplementary Note 1.

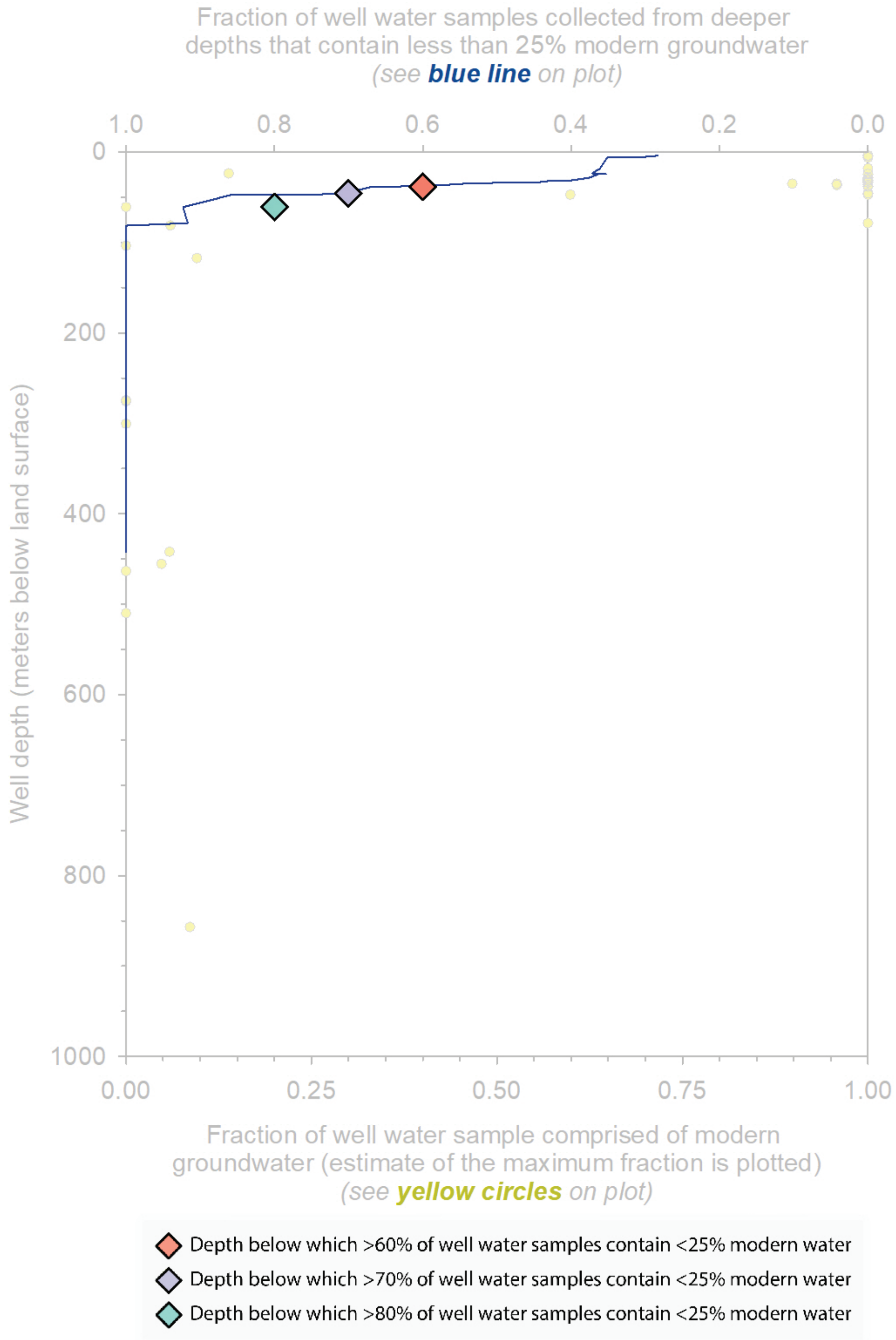

Supplementary Fig. 18. Eastern Flatwoods Southshores modern well water prevalence with depth. For details on symbology see the paragraph at the beginning of Supplementary Note 1.

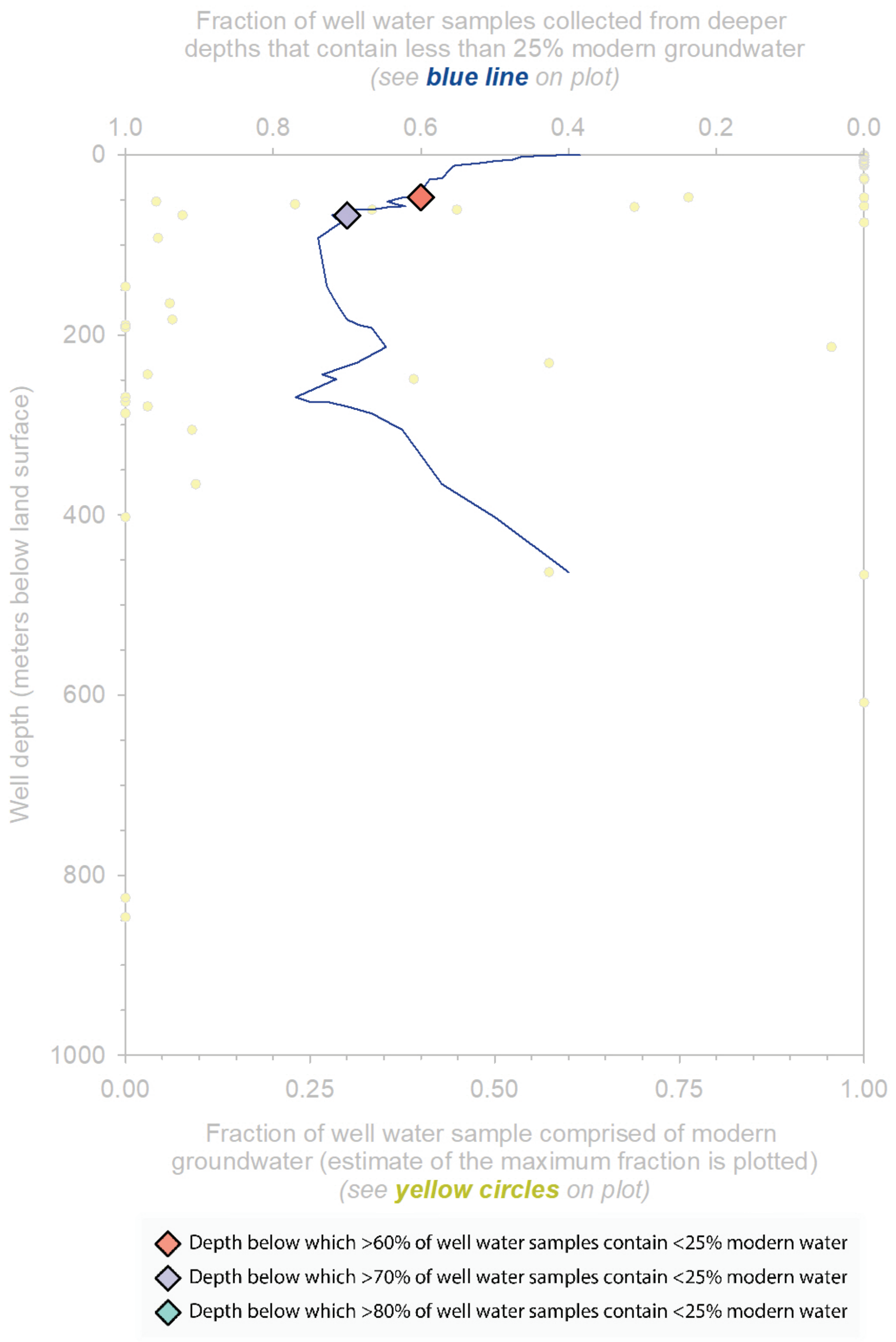

Supplementary Fig. 19. Intermediate Aquifer modern well water prevalence with depth. For details on symbology see the paragraph at the beginning of Supplementary Note 1.

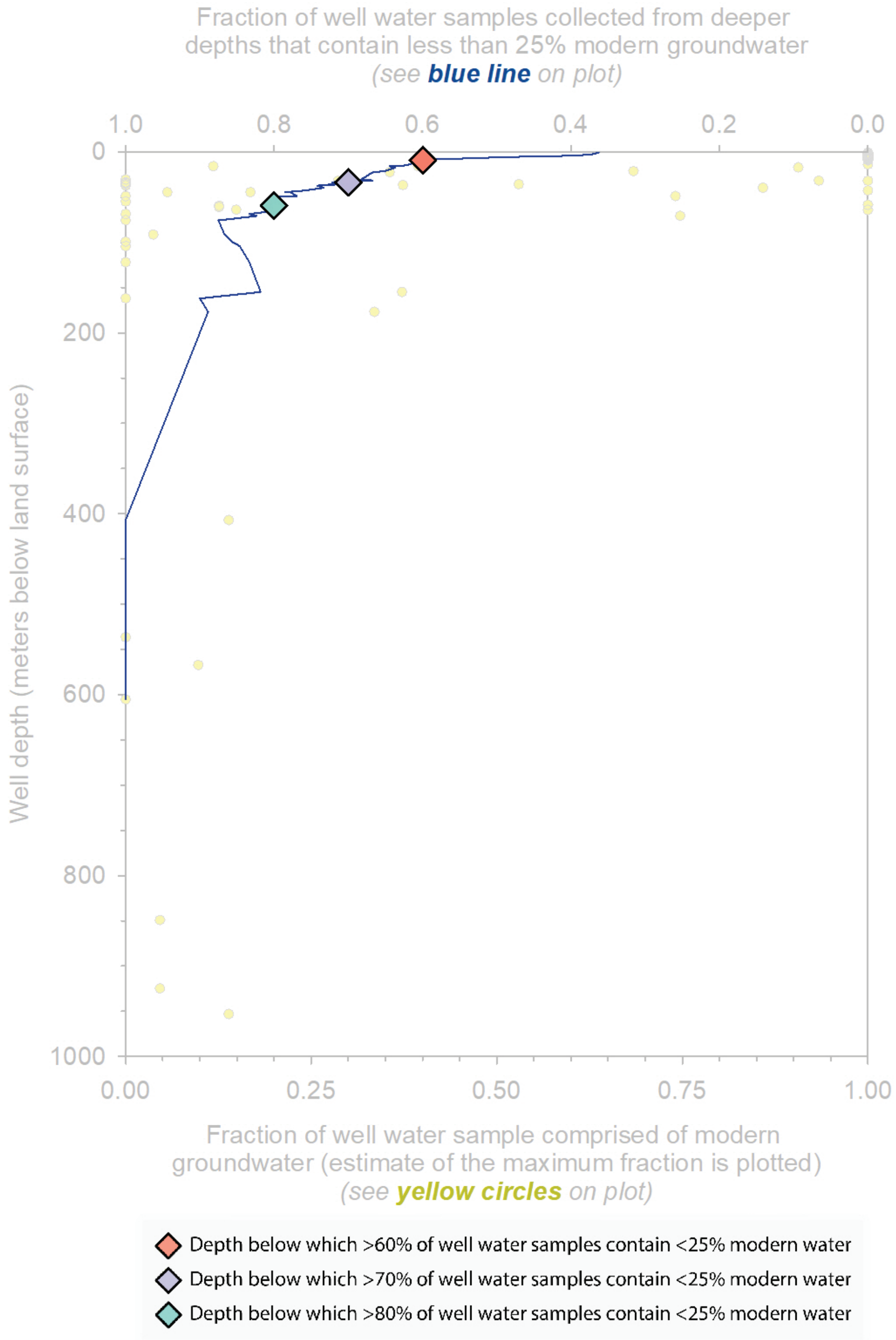

Supplementary Fig. 20. Lower Coastal Plain modern well water prevalence with depth.
For details on symbology see the paragraph at the beginning of Supplementary Note 1.

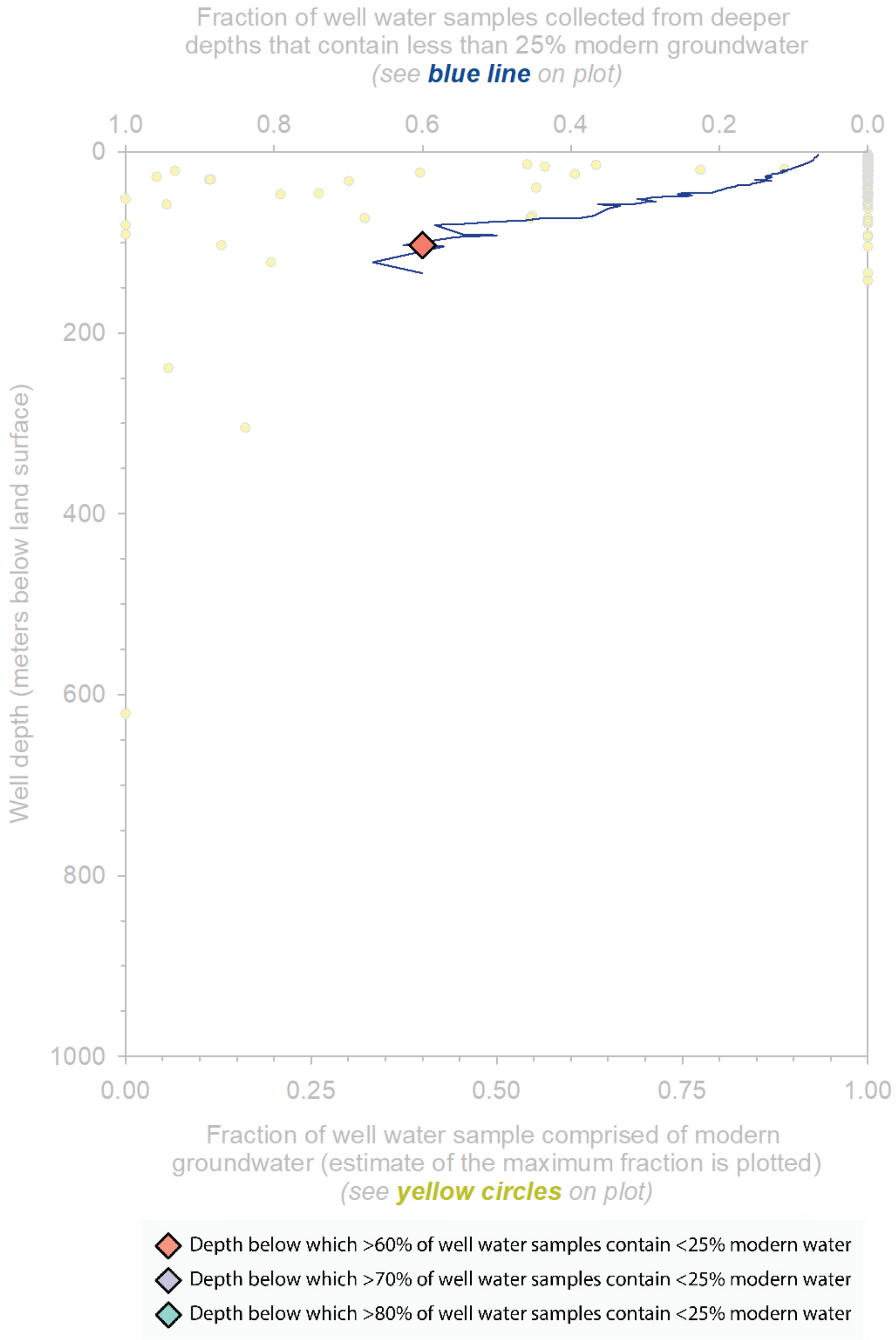

Supplementary Fig. 21. Ocala Uplift modern well water prevalence with depth. For details on symbology see the paragraph at the beginning of Supplementary Note 1.

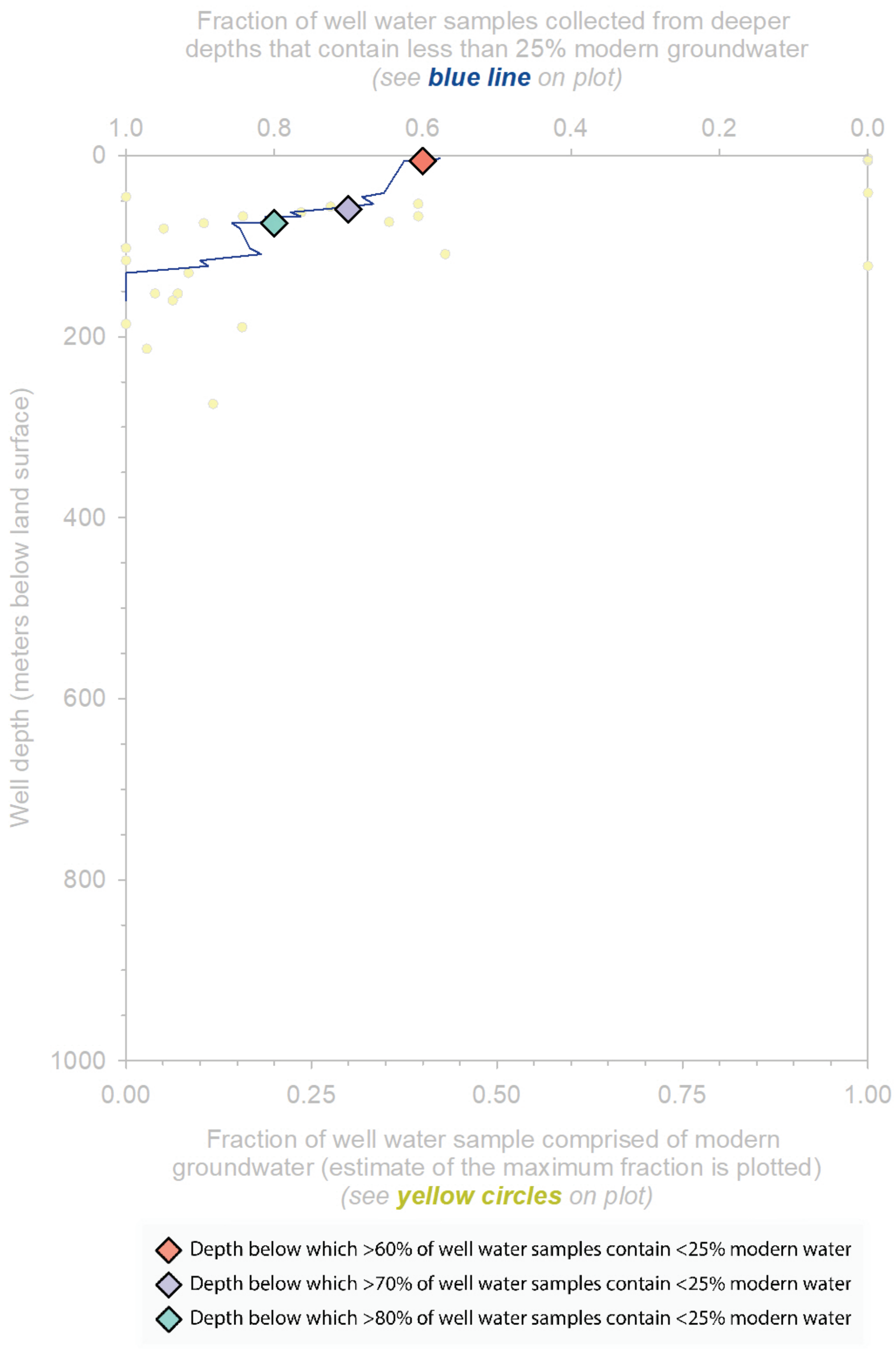

Supplementary Fig. 22. Sea Island modern well water prevalence with depth. For details on symbology see the paragraph at the beginning of Supplementary Note 1.

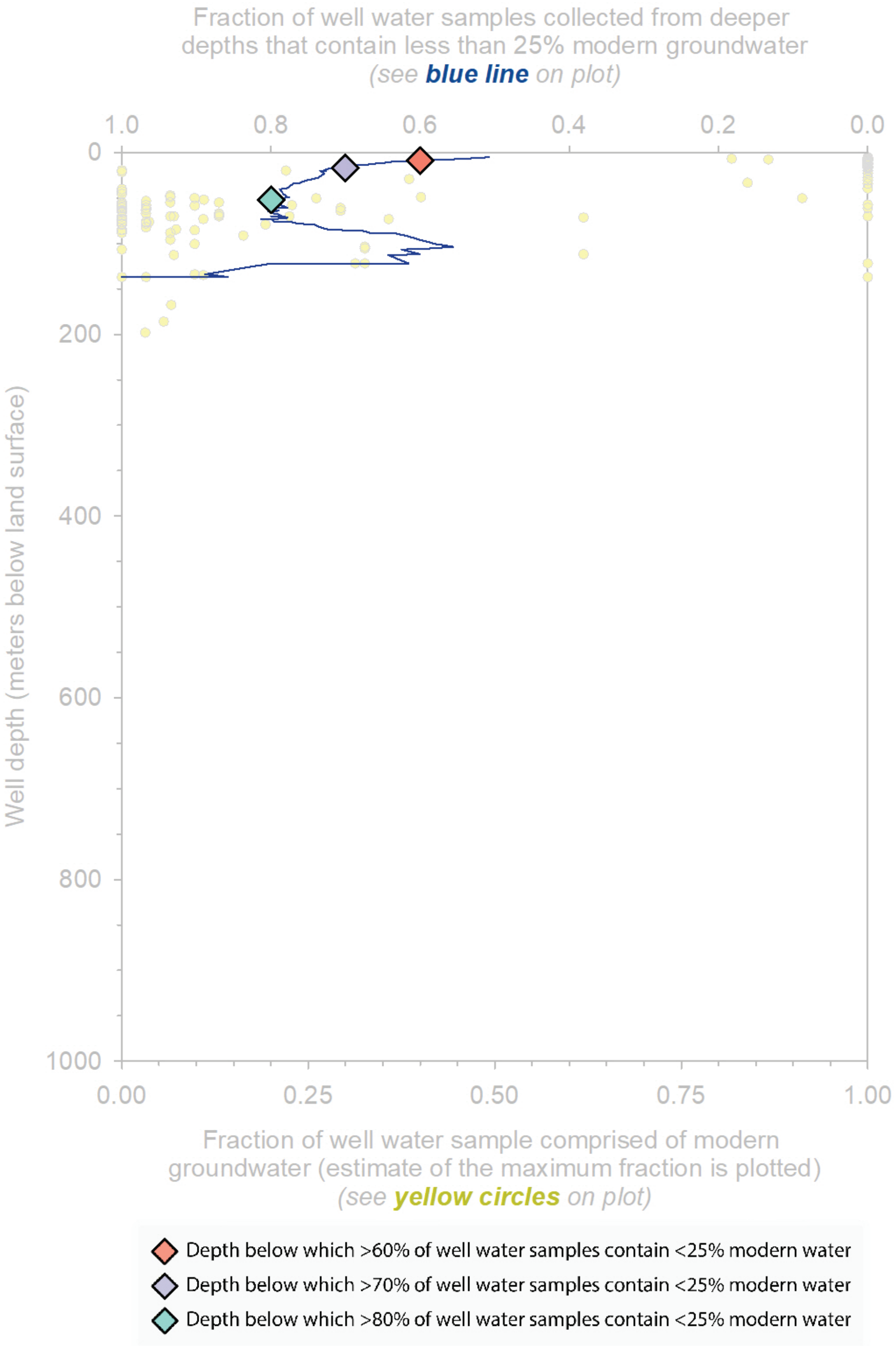

Supplementary Fig. 23. Tifton Upland modern well water prevalence with depth. For details on symbology see the paragraph at the beginning of Supplementary Note 1.

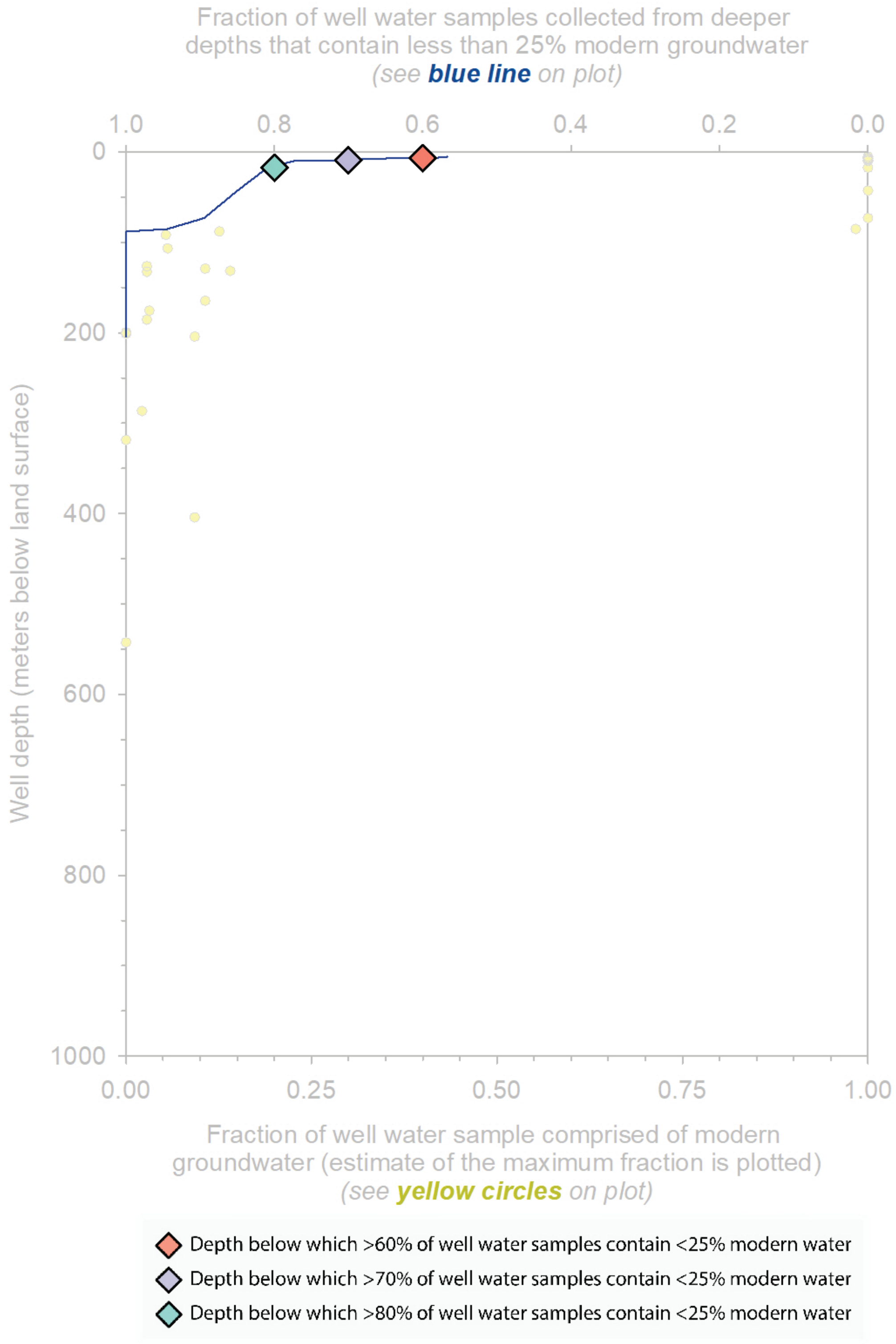

Supplementary Fig. 24. Vidalia Upland modern well water prevalence with depth. For details on symbology see the paragraph at the beginning of Supplementary Note 1.

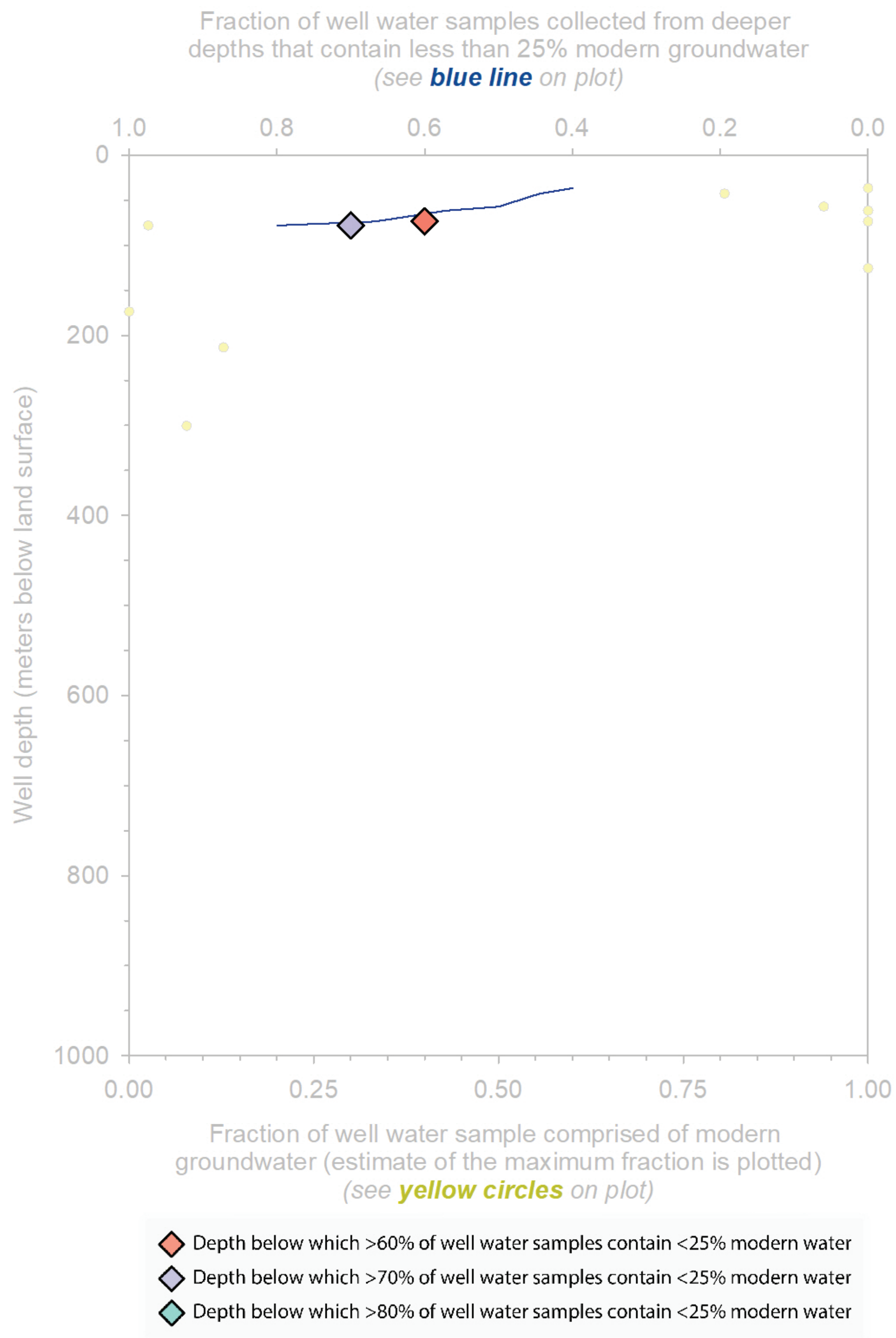

Supplementary Fig. 25. Catahoula Area modern well water prevalence with depth. For details on symbology see the paragraph at the beginning of Supplementary Note 1.

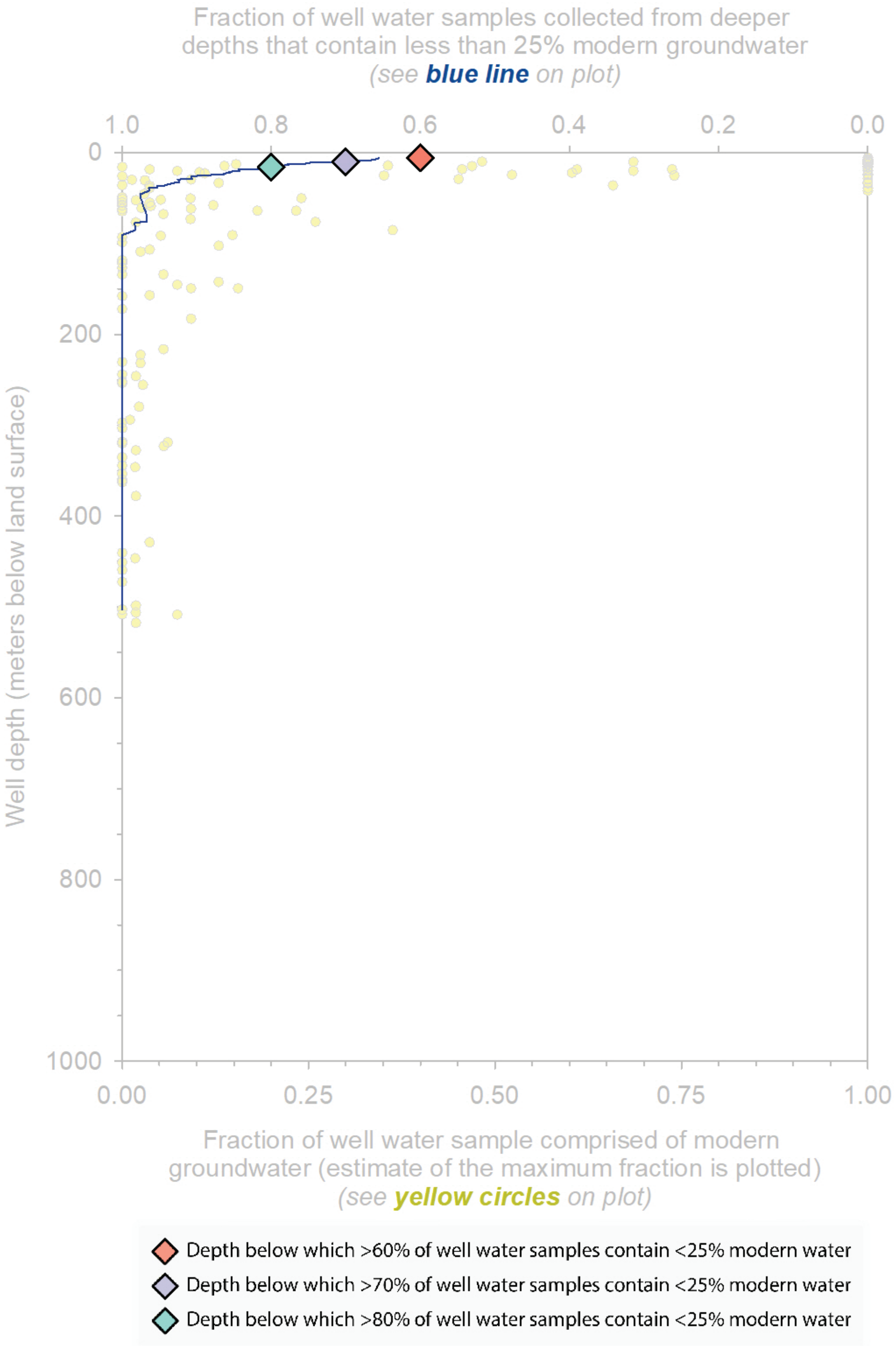

Supplementary Fig. 26. Houston-Galveston Area modern well water prevalence with depth. For details on symbology see the paragraph at the beginning of Supplementary Note 1.

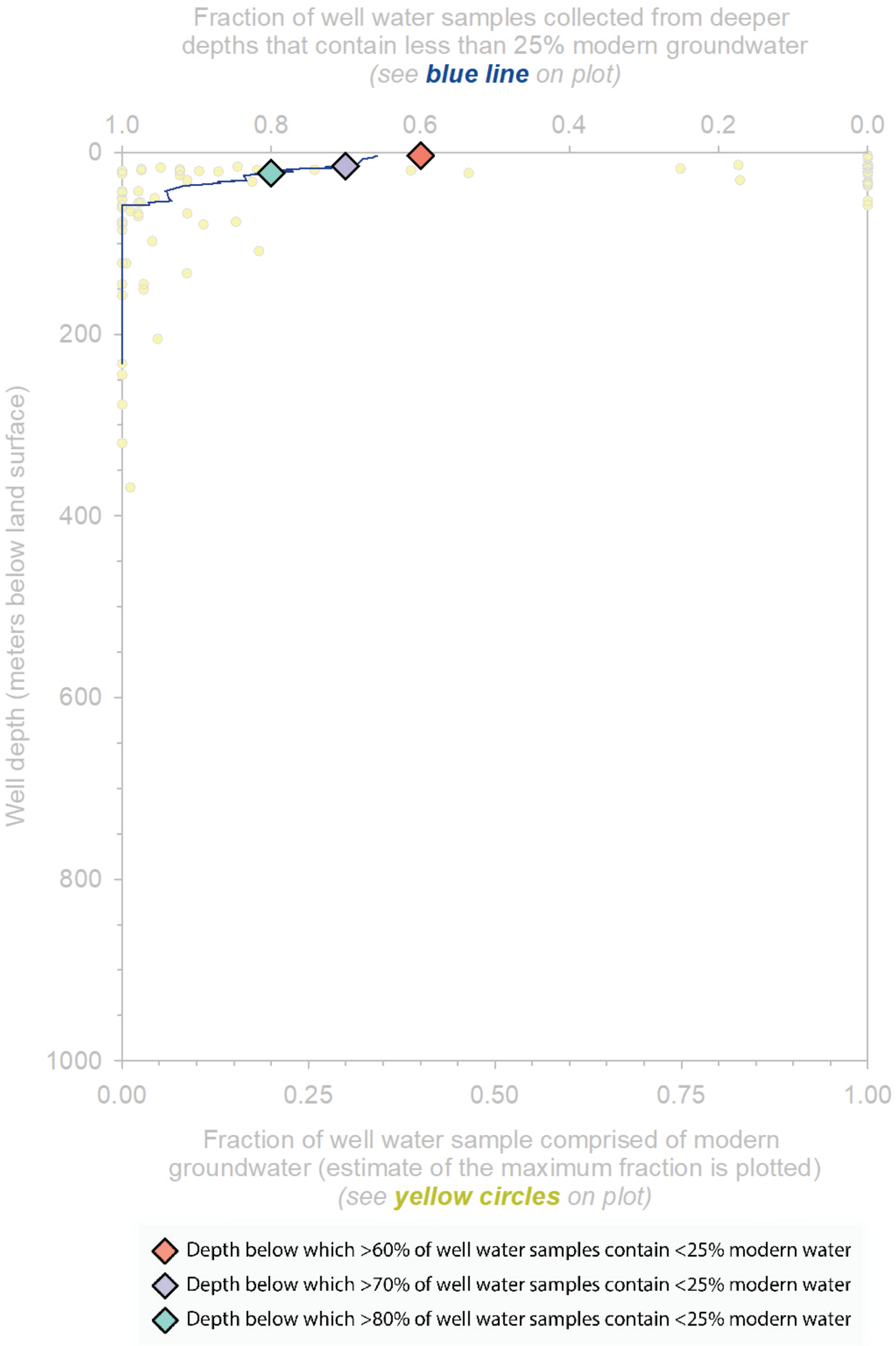

Supplementary Fig. 27. Lafayette Area modern well water prevalence with depth. For details on symbology see the paragraph at the beginning of Supplementary Note 1.

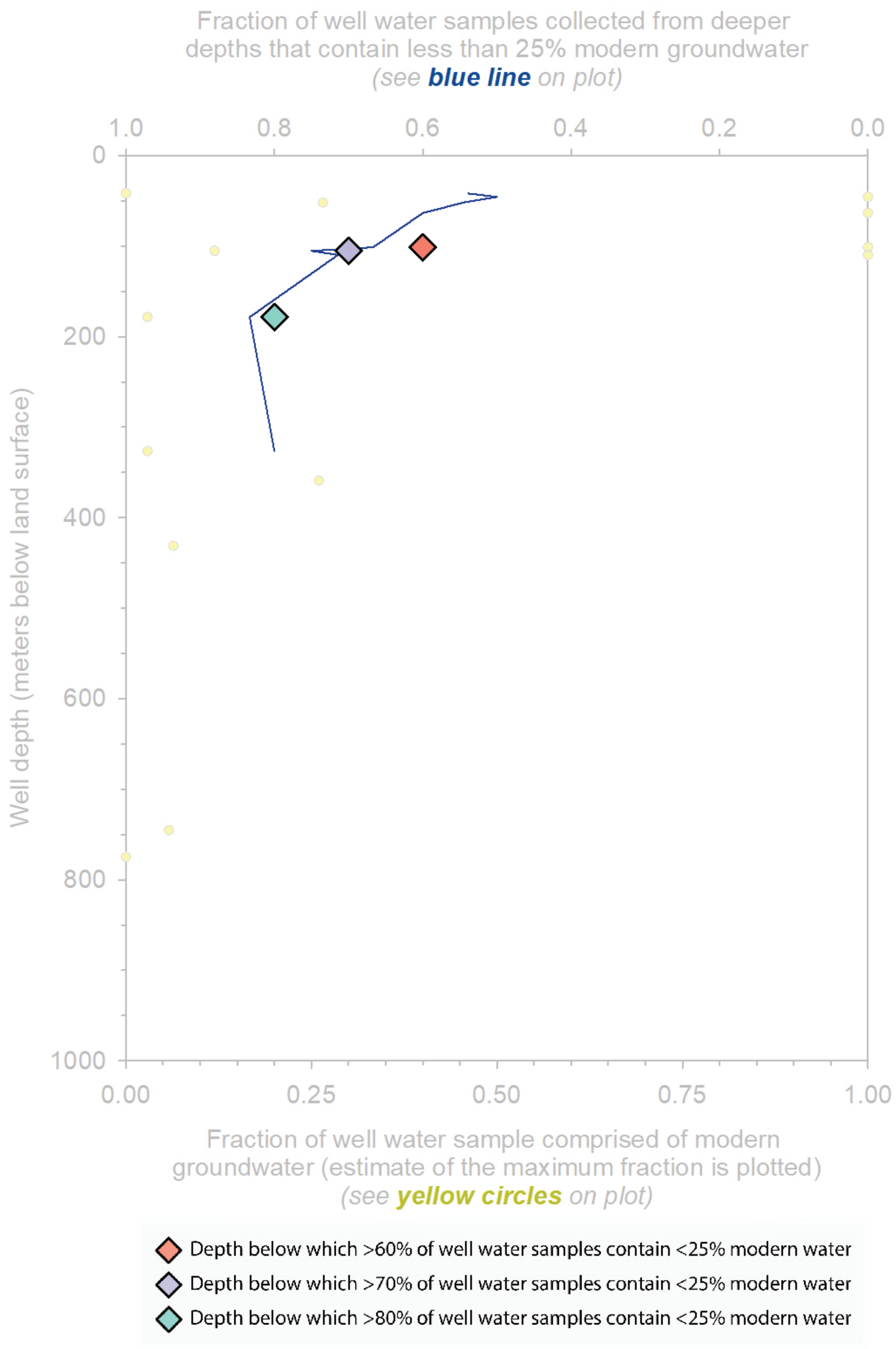

Supplementary Fig. 28. Southern Hills modern well water prevalence with depth. For details on symbology see the paragraph at the beginning of Supplementary Note 1.

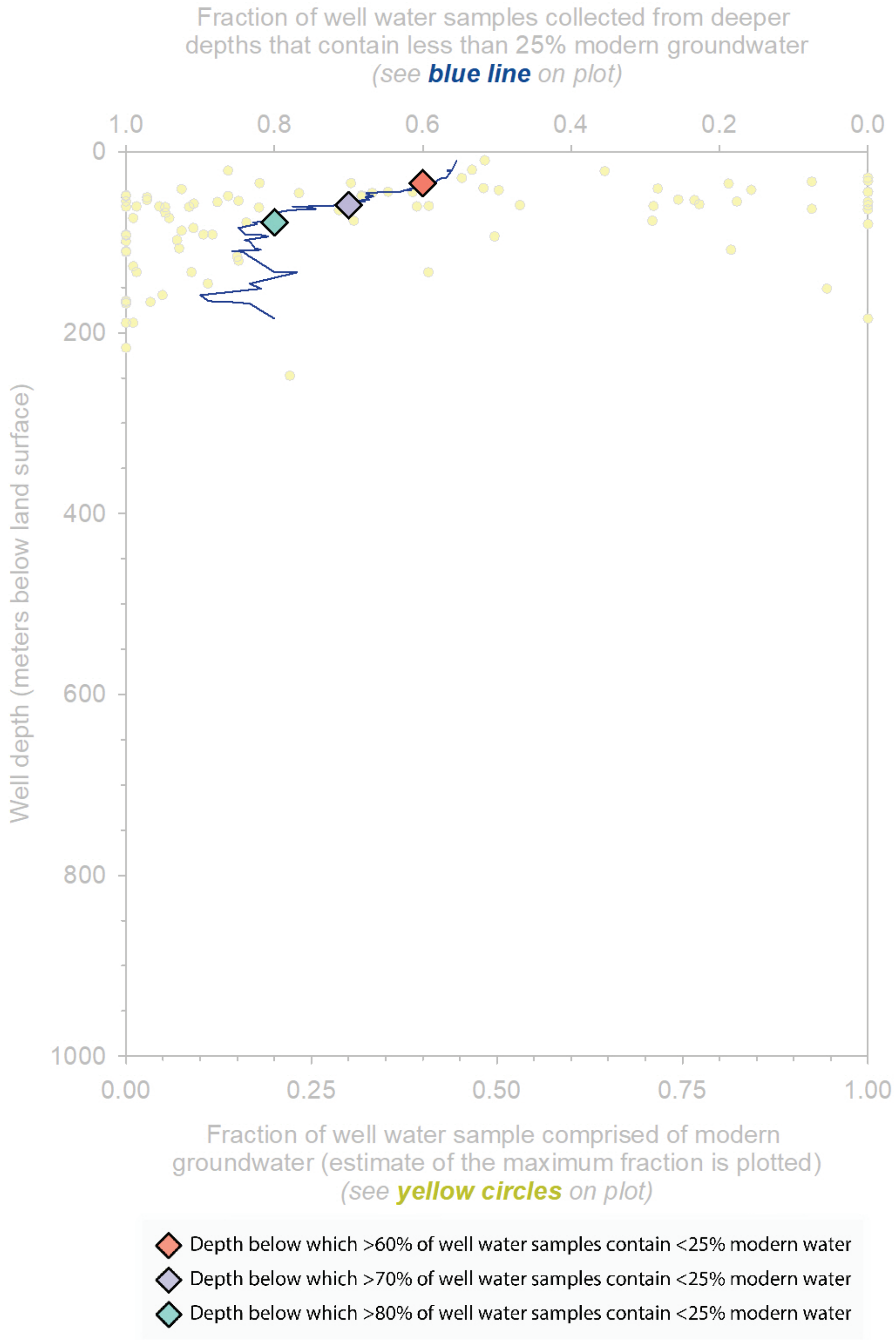

Supplementary Fig. 29. Central High Plains modern well water prevalence with depth. For details on symbology see the paragraph at the beginning of Supplementary Note 1.

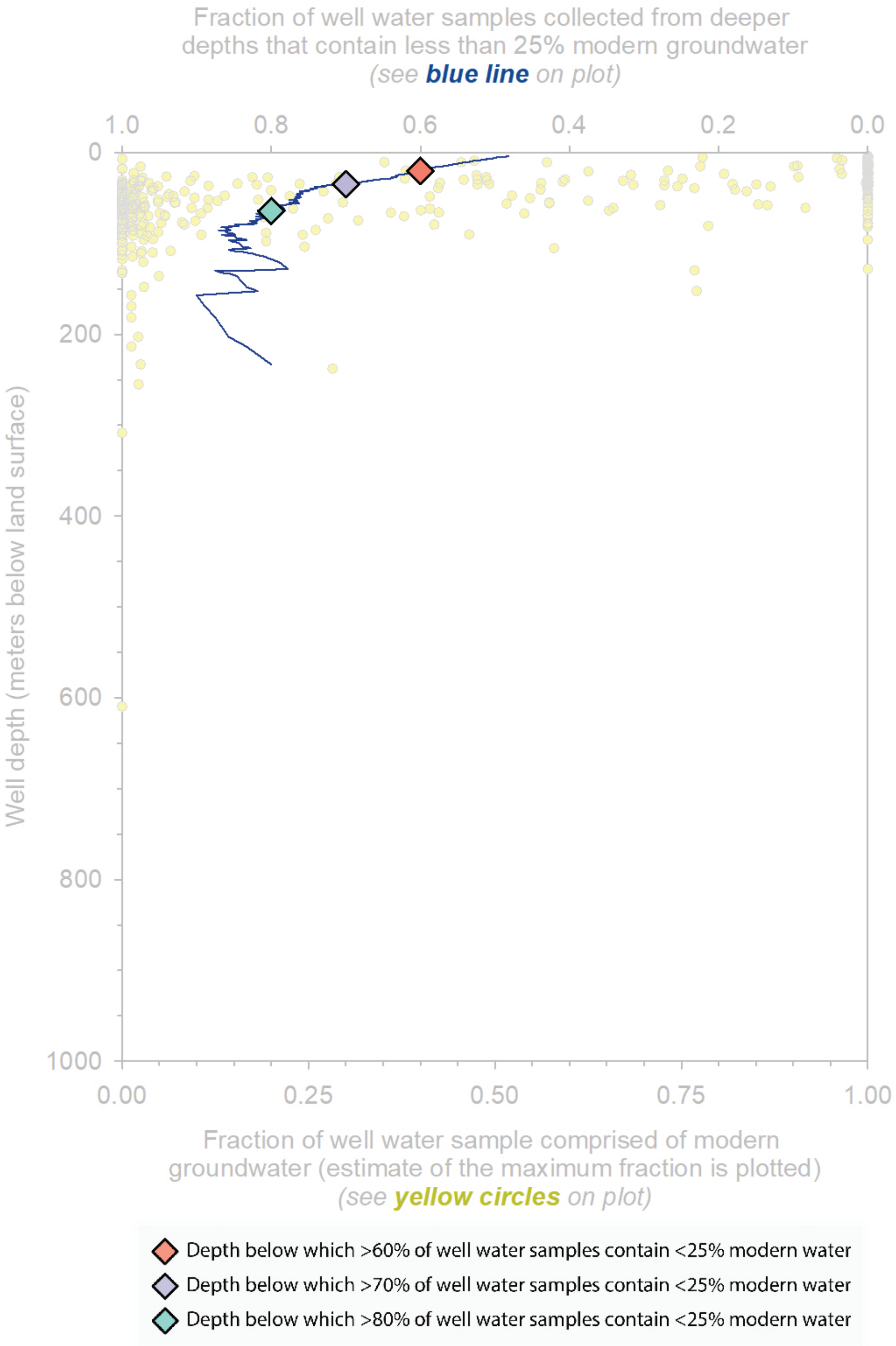

Supplementary Fig. 30. Northern High Plains modern well water prevalence with depth. For details on symbology see the paragraph at the beginning of Supplementary Note 1.

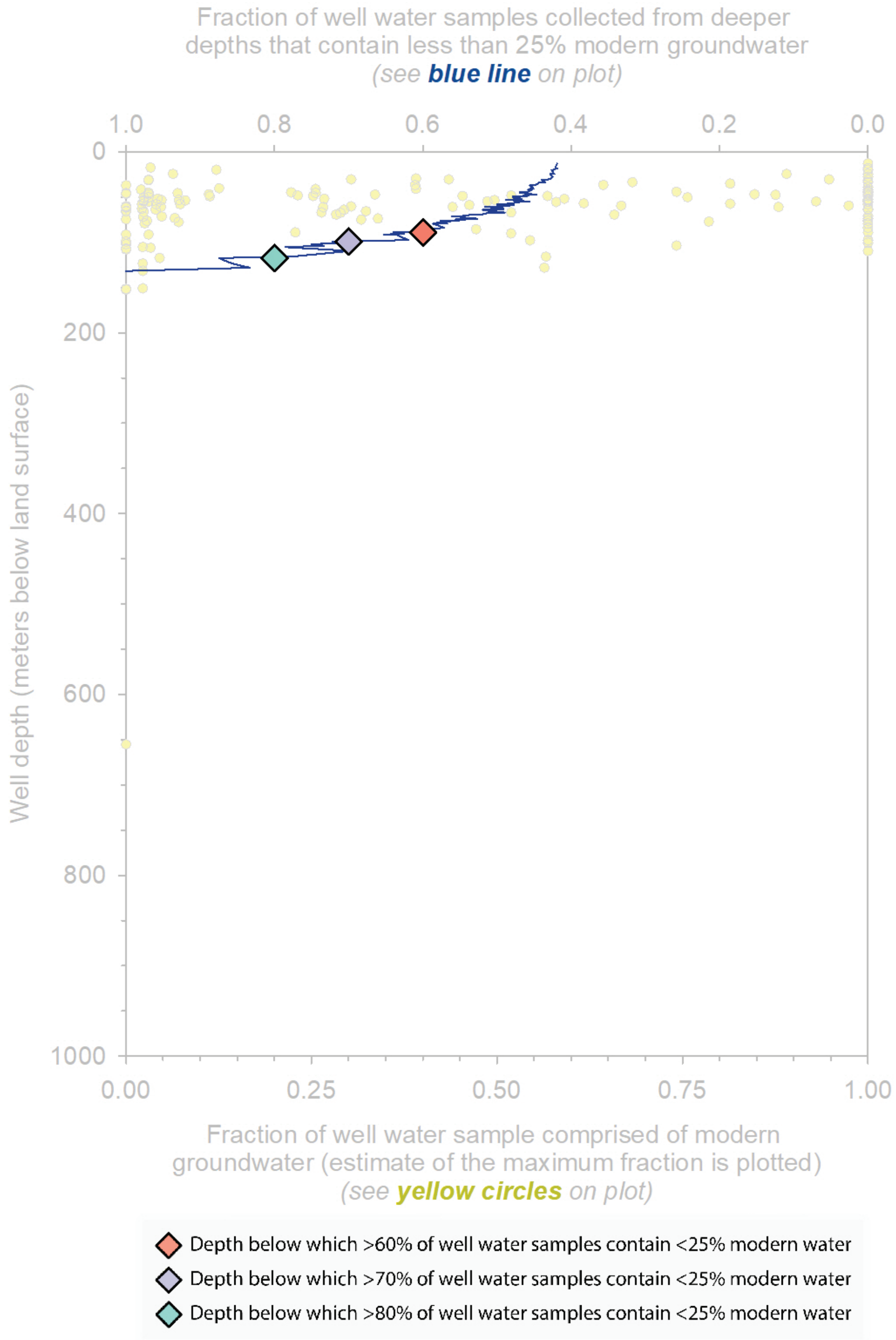

Supplementary Fig. 31. Southern High Plains modern well water prevalence with depth. For details on symbology see the paragraph at the beginning of Supplementary Note 1.

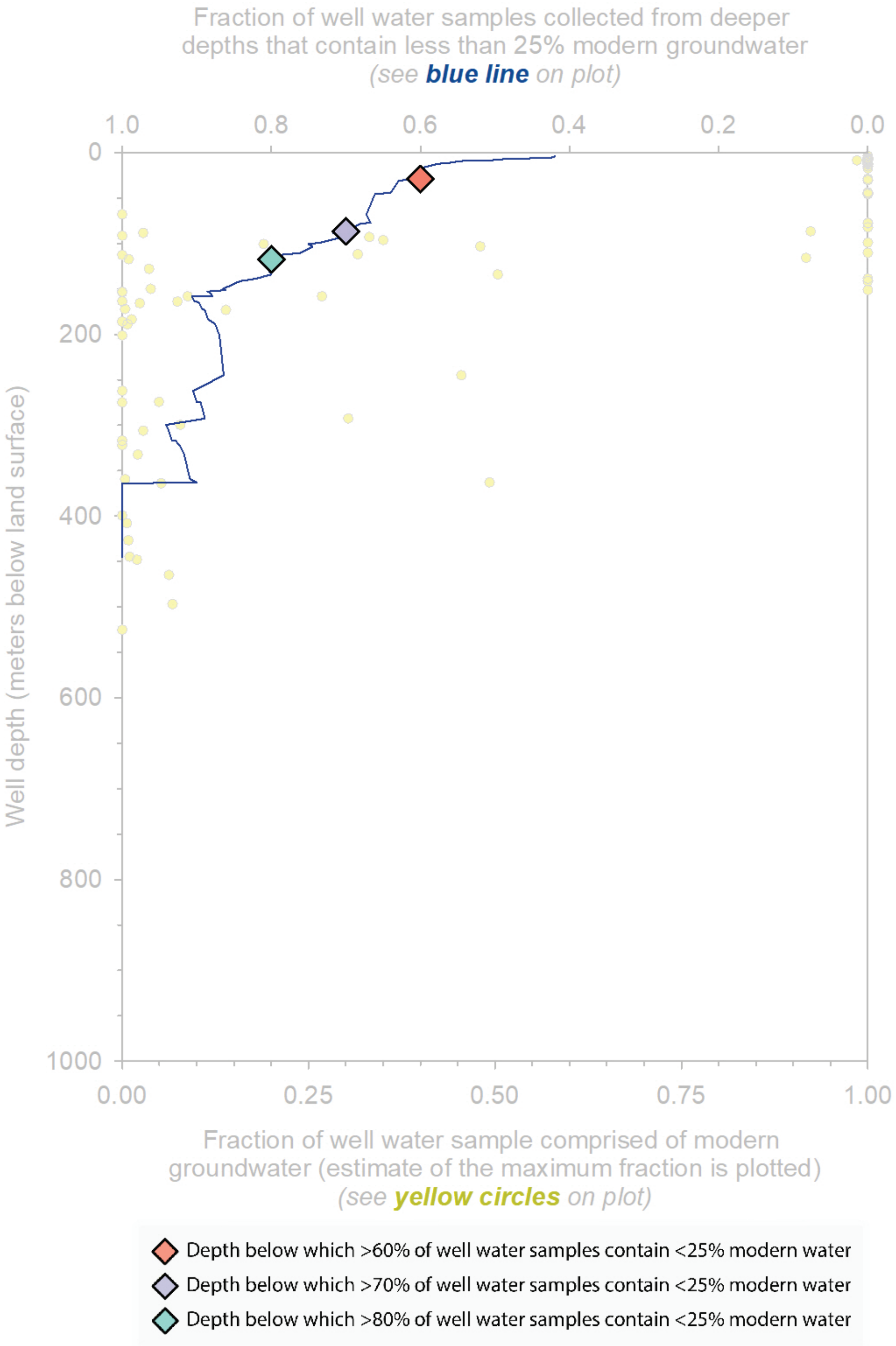

Supplementary Fig. 32. Albuquerque Basin modern well water prevalence with depth. For details on symbology see the paragraph at the beginning of Supplementary Note 1.

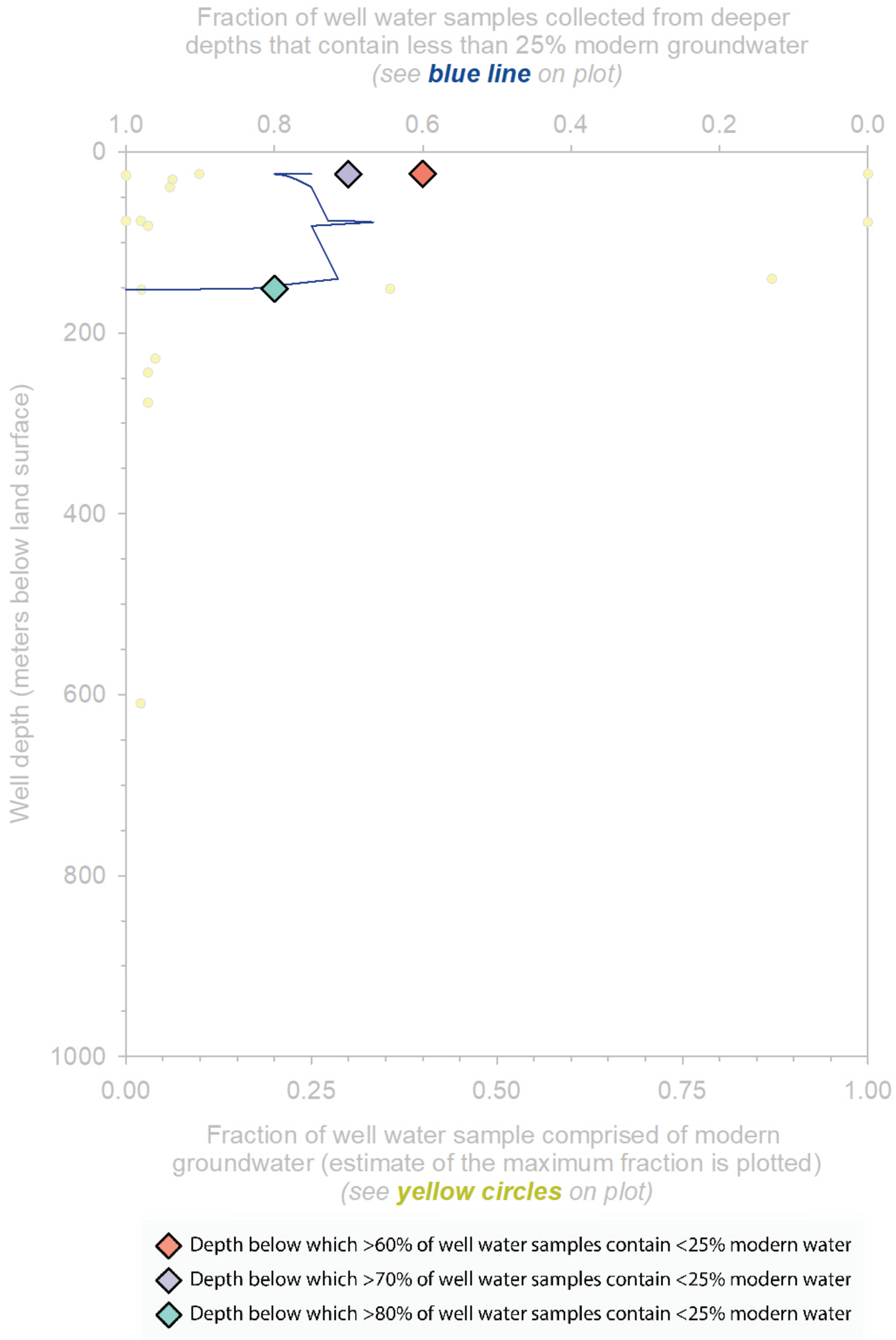

Supplementary Fig. 33. Espanola Basin modern well water prevalence with depth. For details on symbology see the paragraph at the beginning of Supplementary Note 1.

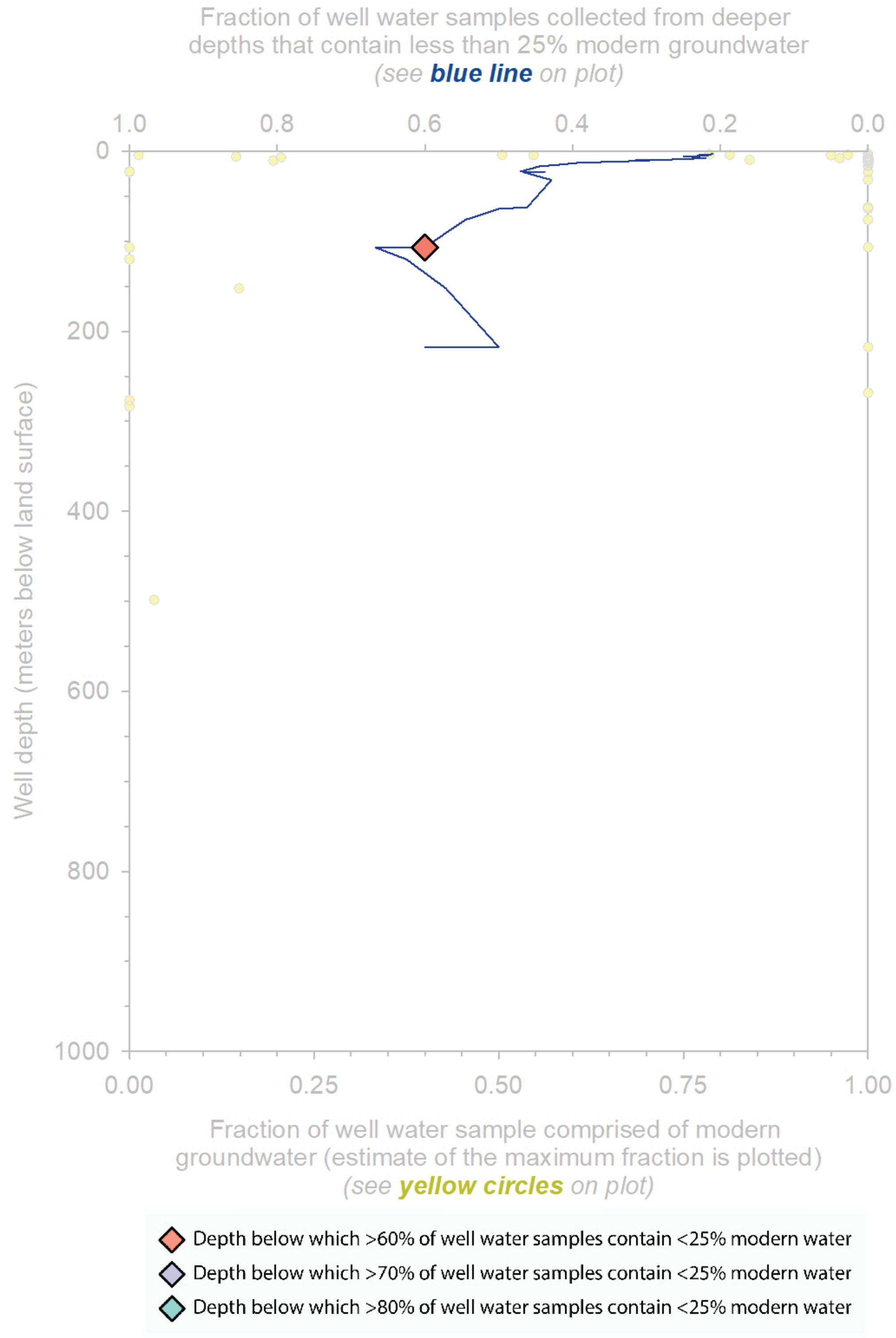

Supplementary Fig. 34. San Luis Valley modern well water prevalence with depth. For details on symbology see the paragraph at the beginning of Supplementary Note 1.

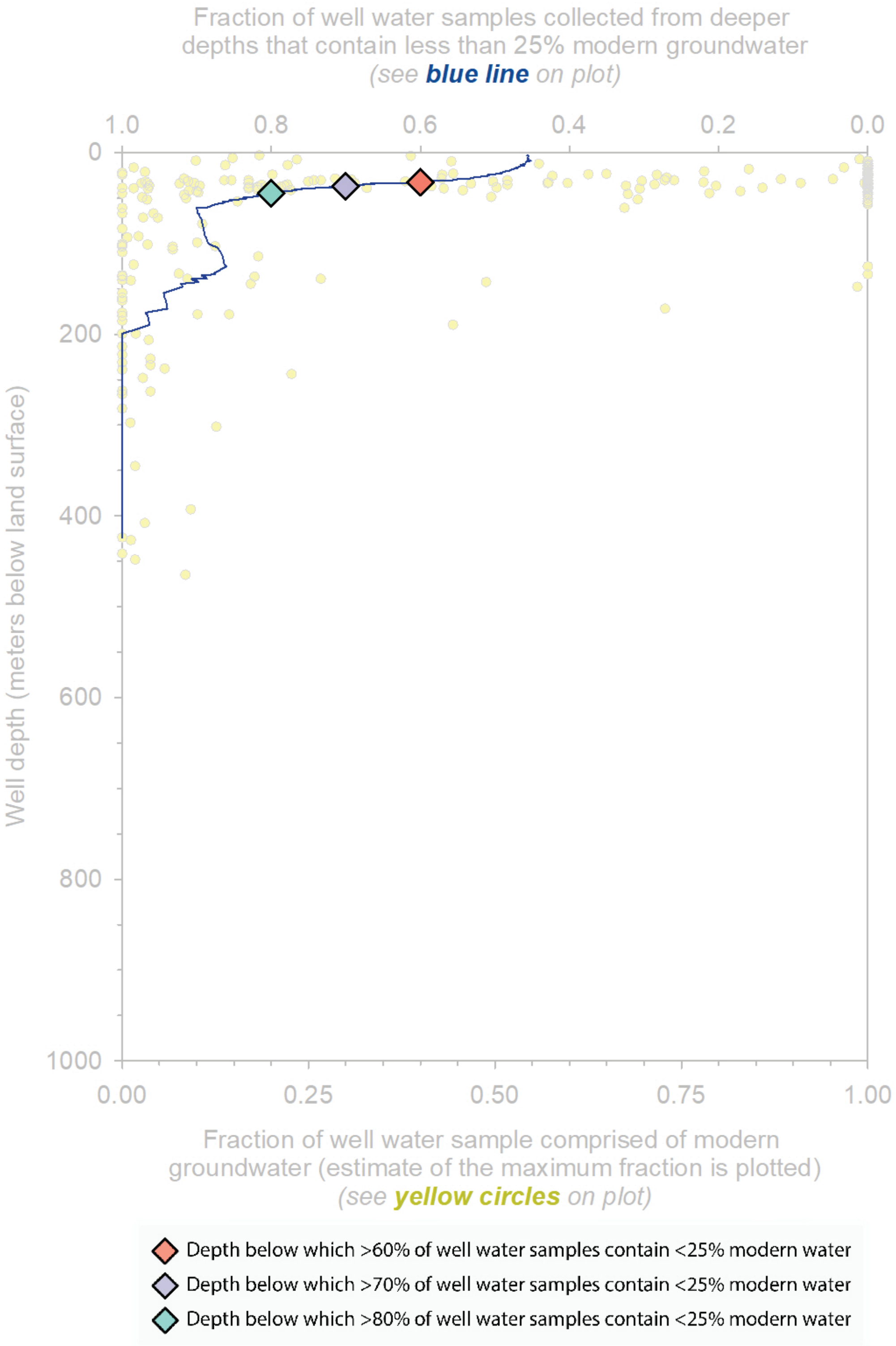

Supplementary Fig. 35. Central Mississippi Embayment modern well water prevalence with depth. For details on symbology see the paragraph at the beginning of Supplementary Note 1.

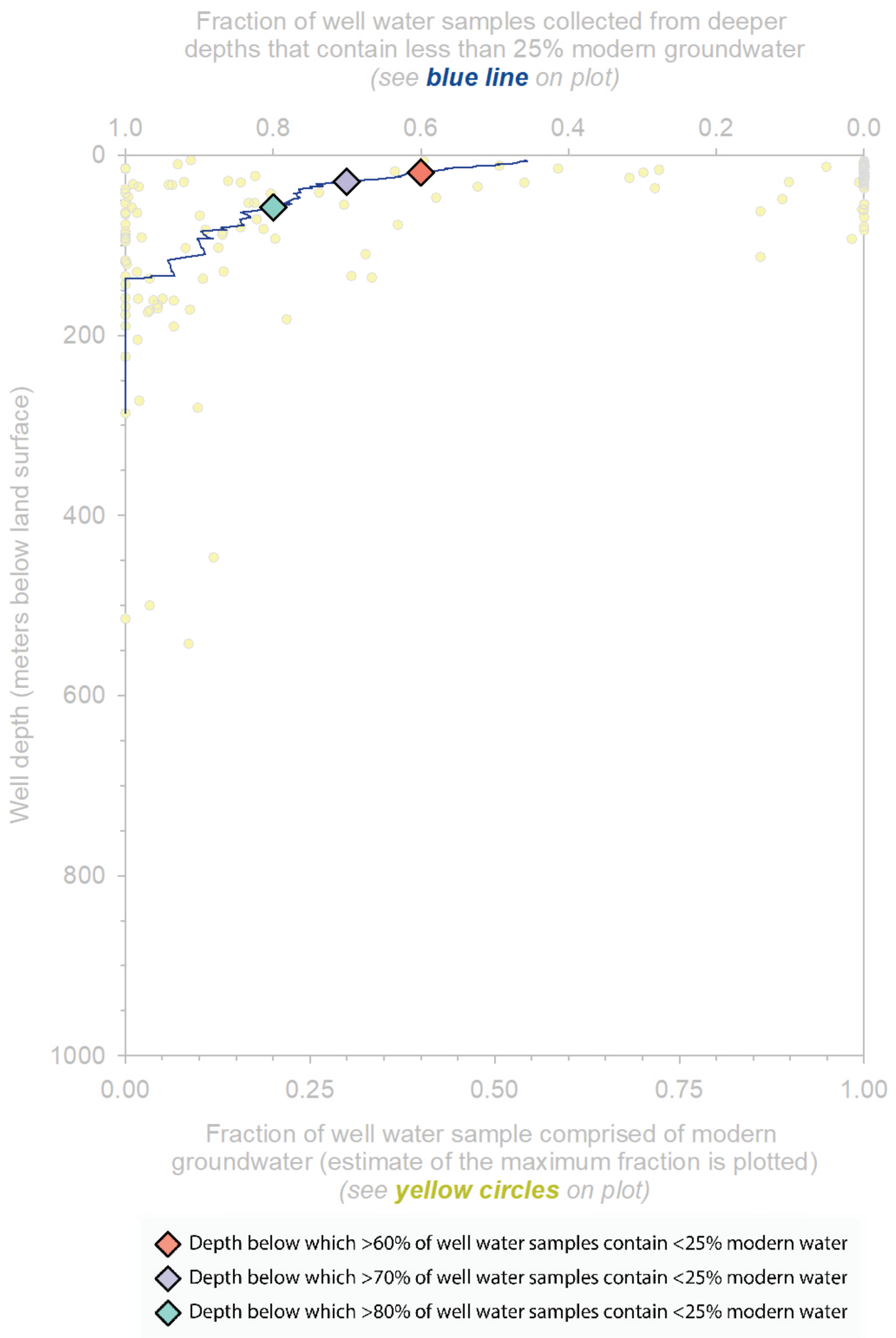

Supplementary Fig. 36. Eastern Mississippi Embayment modern well water prevalence with depth. For details on symbology see the paragraph at the beginning of Supplementary Note 1.

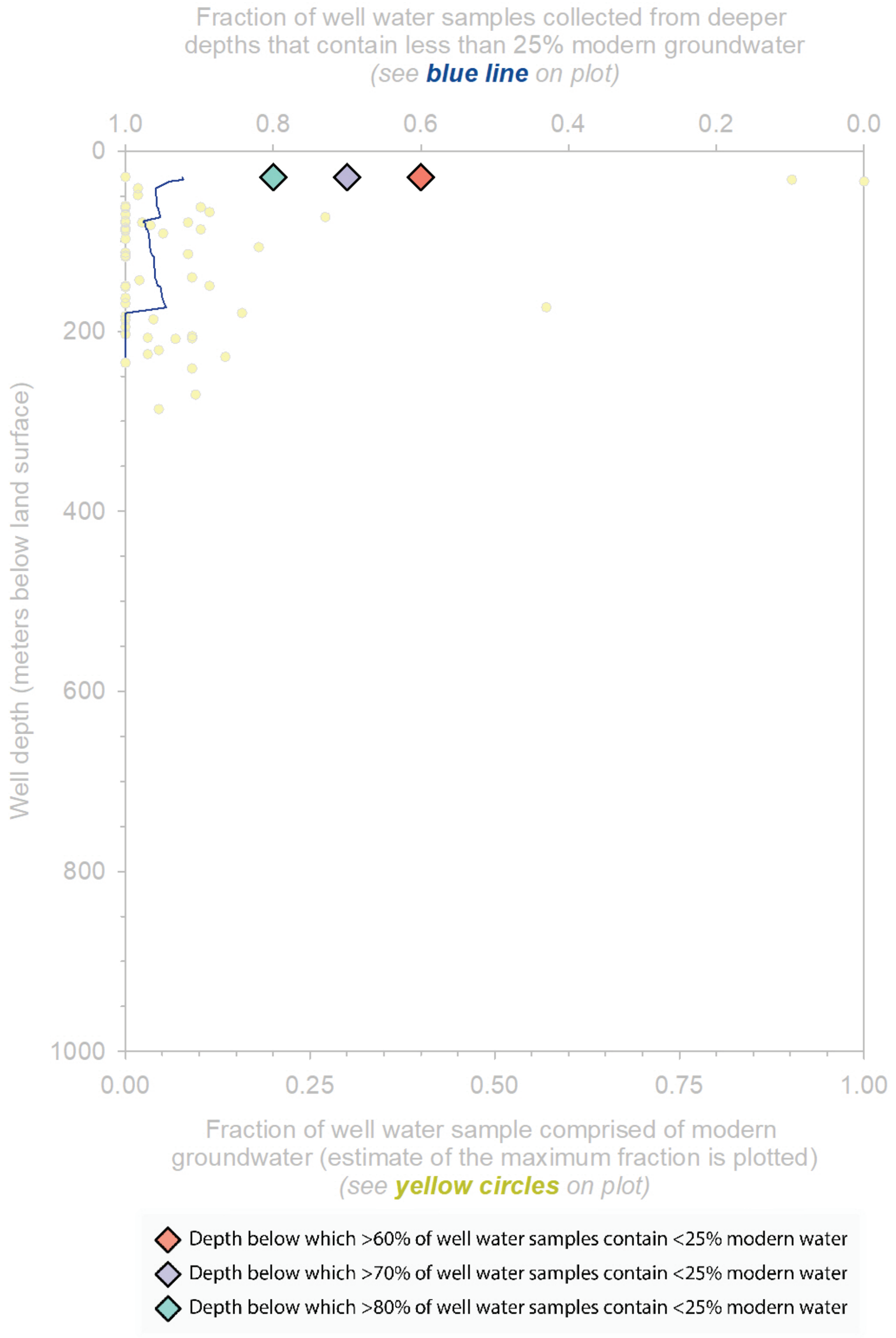

Supplementary Fig. 37. Western Mississippi Embayment modern well water prevalence with depth. For details on symbology see the paragraph at the beginning of Supplementary Note 1.

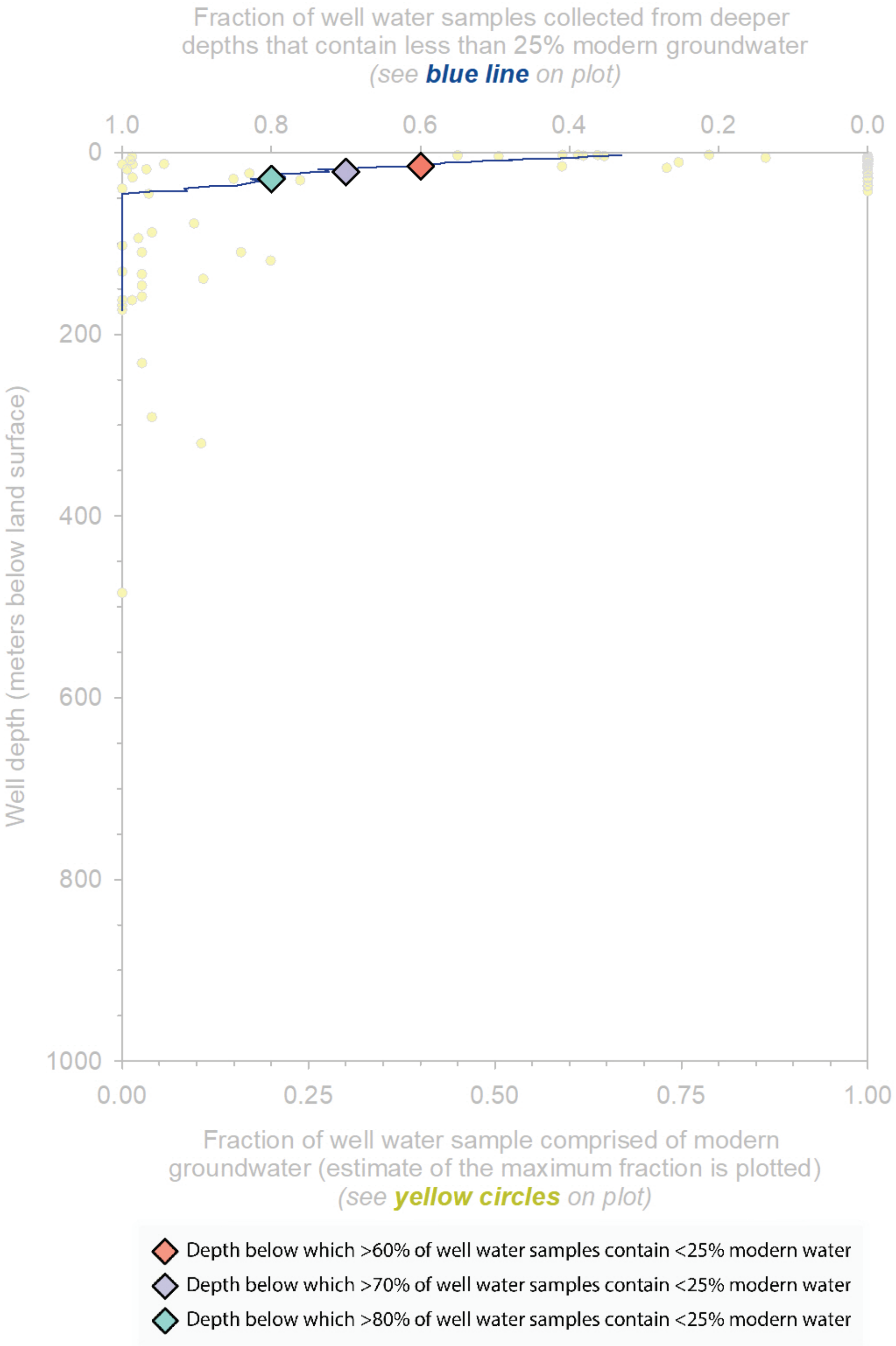

Supplementary Fig. 38. Delmarva Peninsula modern well water prevalence with depth.
For details on symbology see the paragraph at the beginning of Supplementary Note 1.

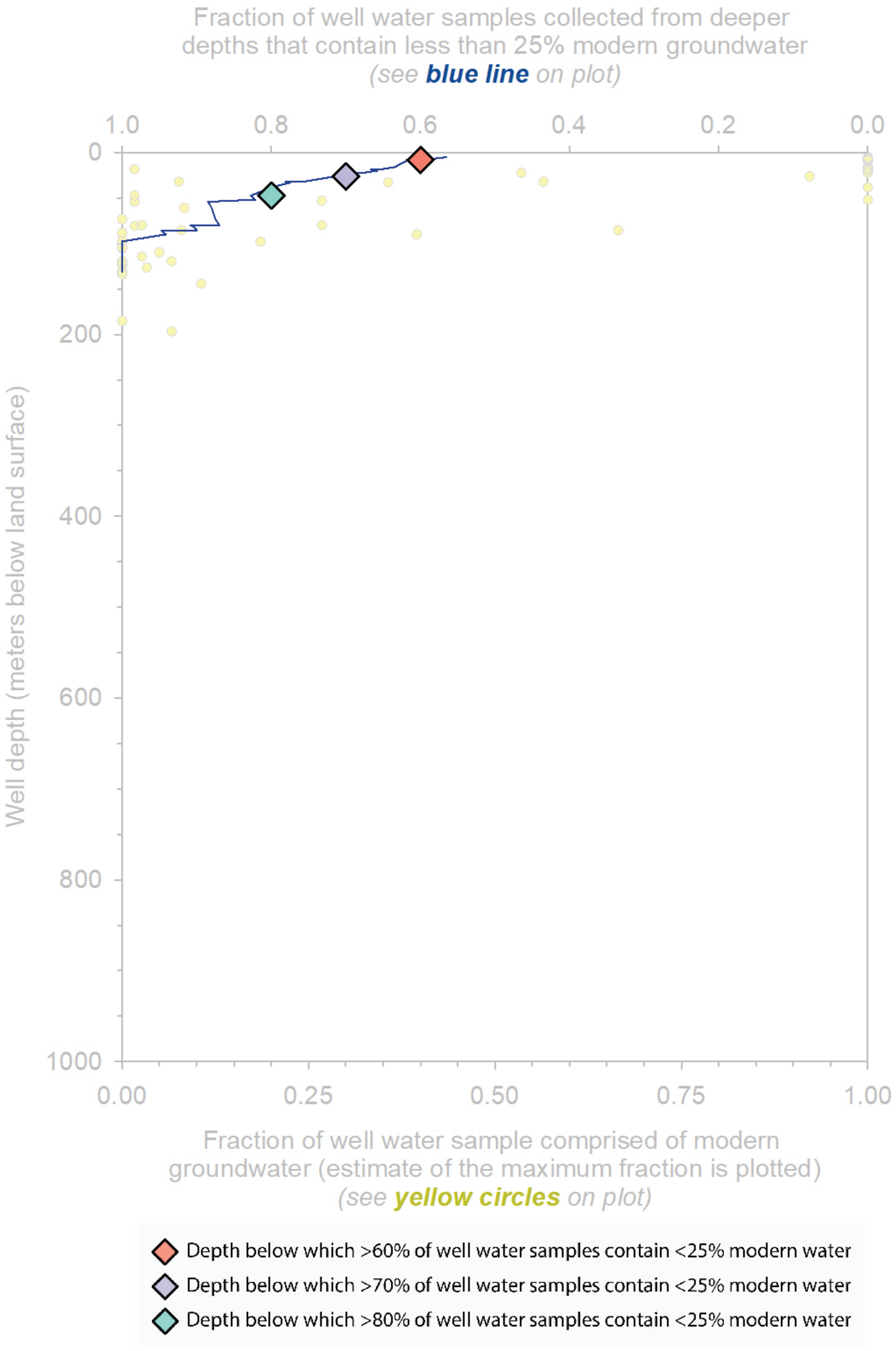

Supplementary Fig. 39. Maryland Western Shores modern well water prevalence with depth. For details on symbology see the paragraph at the beginning of Supplementary Note 1.

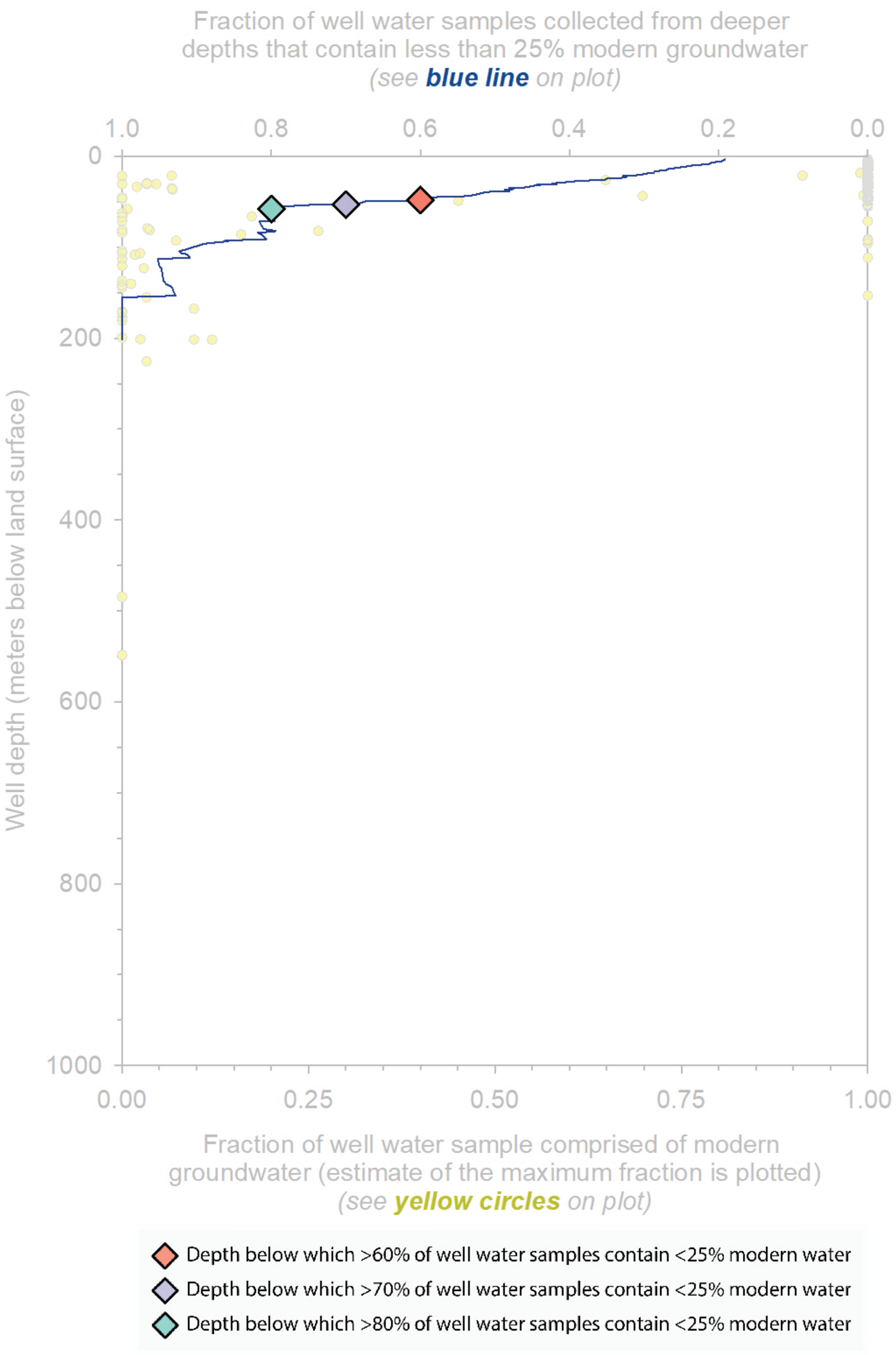

Supplementary Fig. 40. New Jersey Coastal Plain modern well water prevalence with depth. For details on symbology see the paragraph at the beginning of Supplementary Note 1.

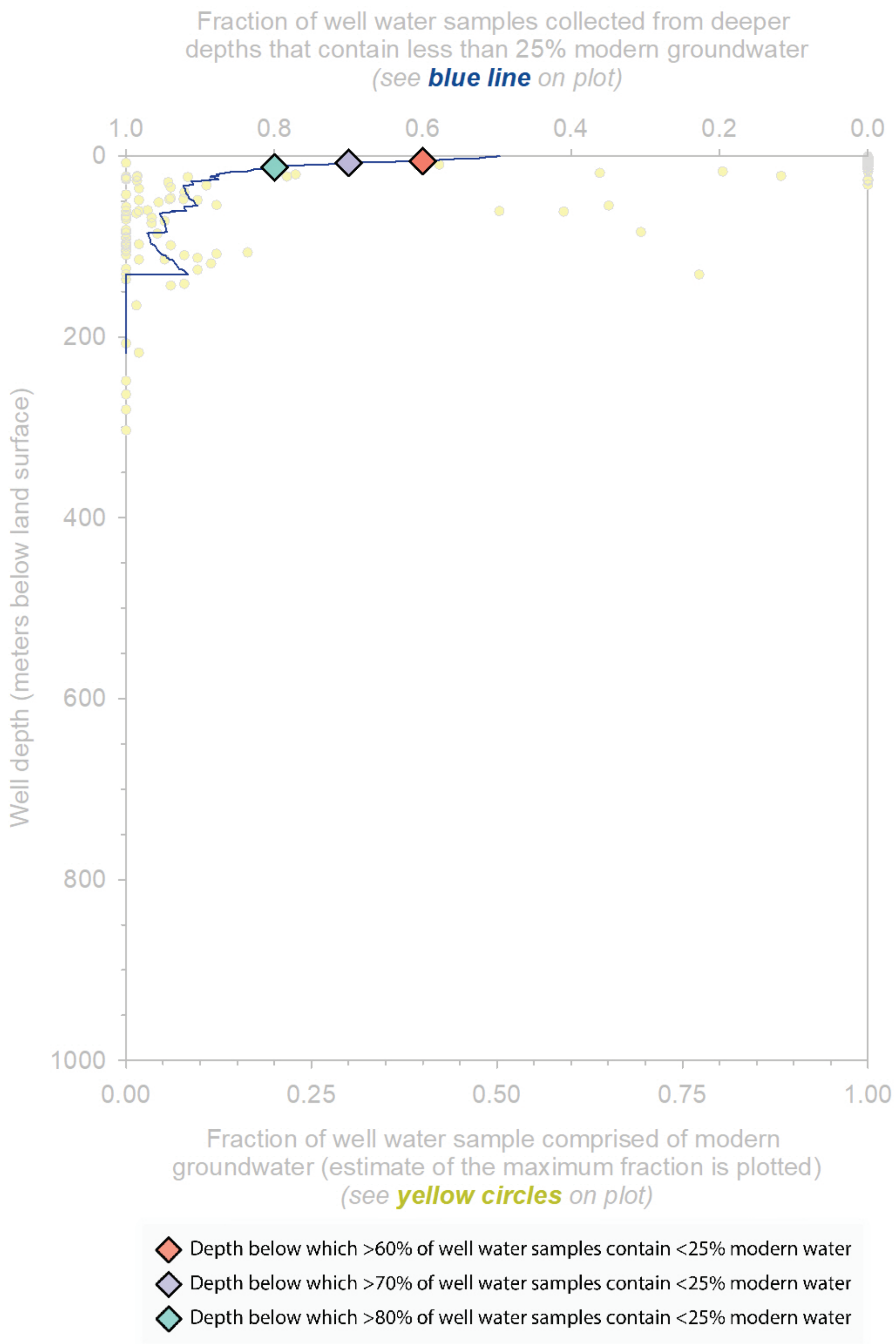

Supplementary Fig. 41. North Carolina and Virginia Coastal Plain modern well water prevalence with depth. For details on symbology see the paragraph at the beginning of Supplementary Note 1.

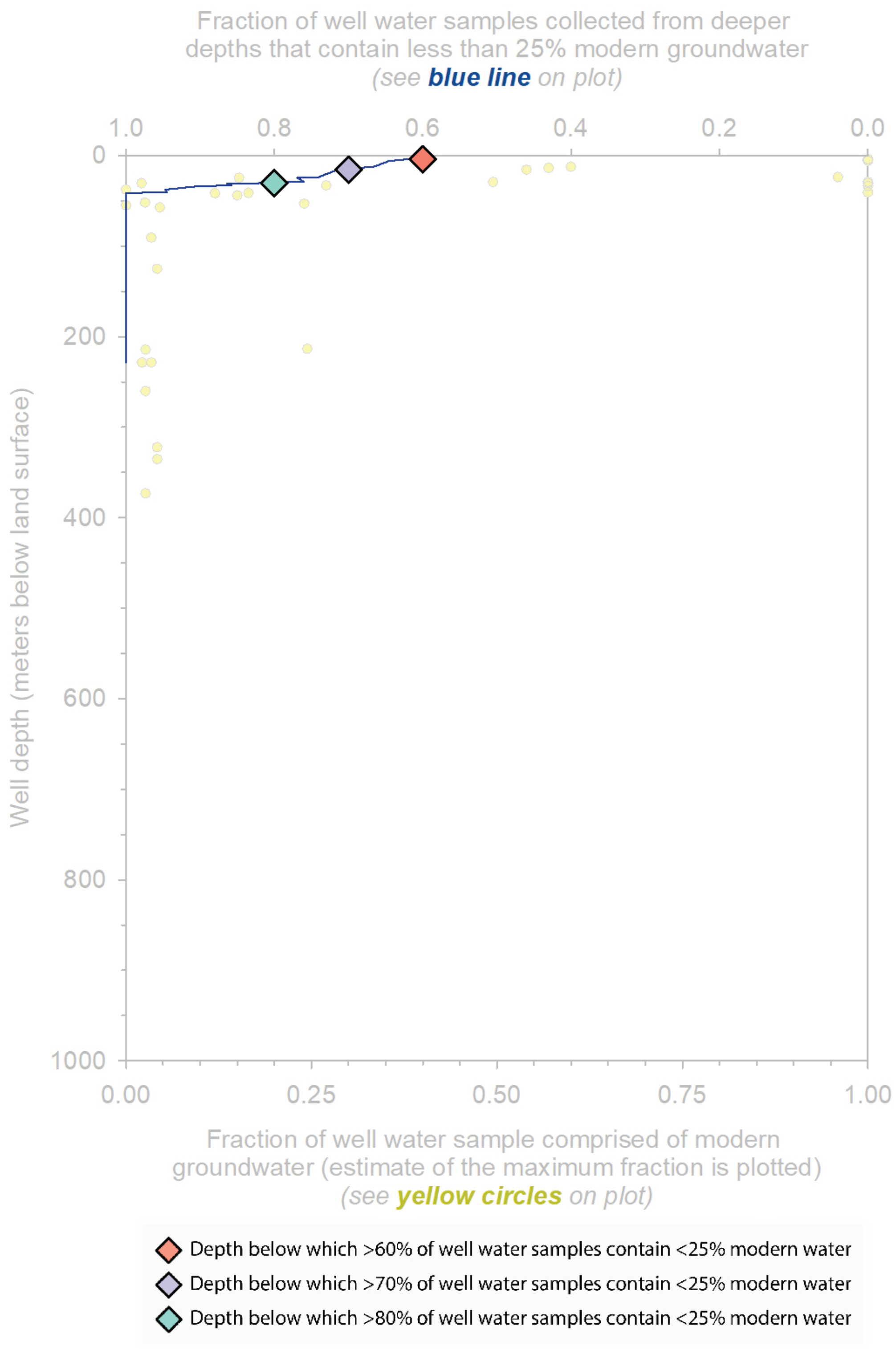

Supplementary Fig. 42. Powder River Basin modern well water prevalence with depth. For details on symbology see the paragraph at the beginning of Supplementary Note 1.

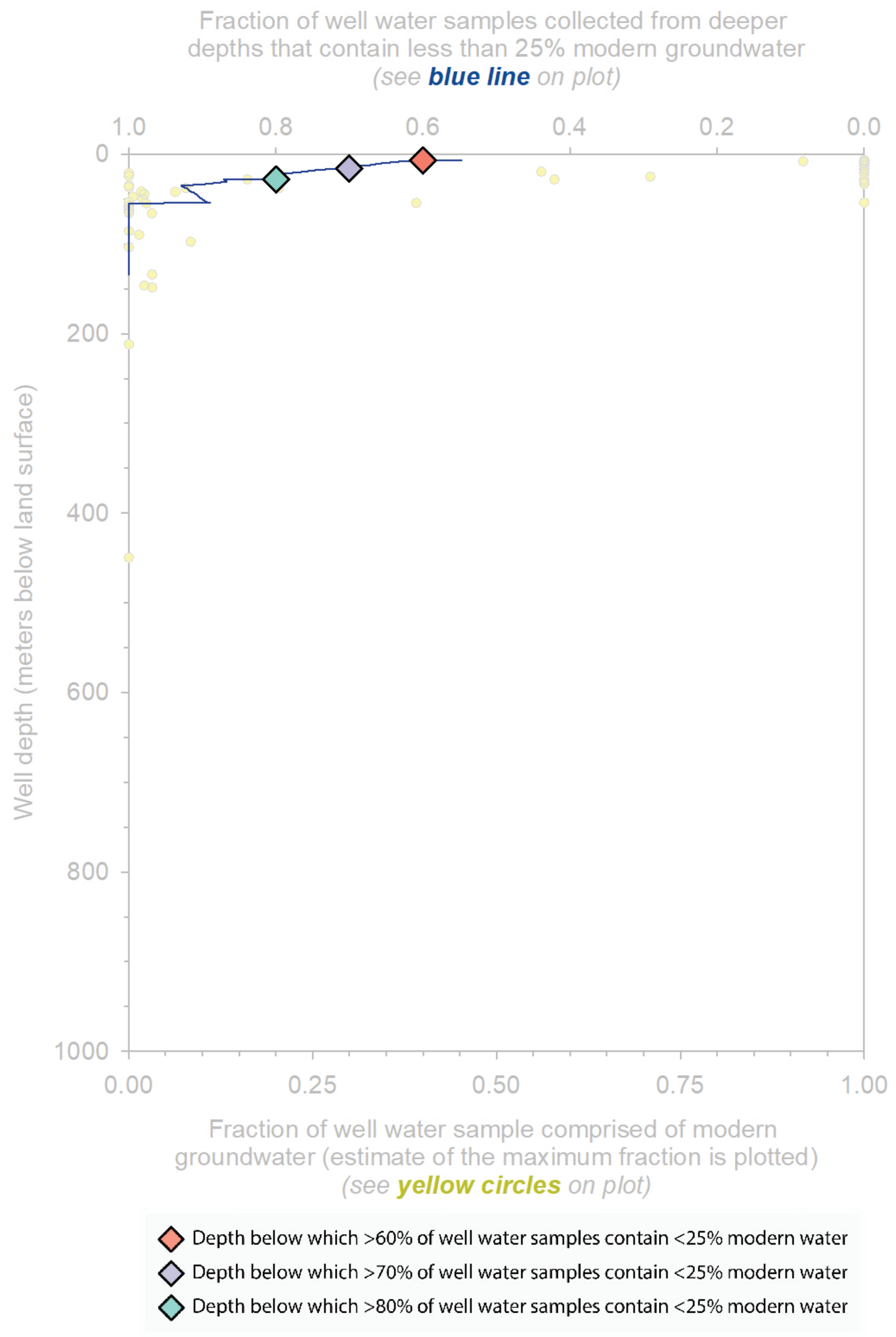

Supplementary Fig. 43. Williston Basin modern well water prevalence with depth. For details on symbology see the paragraph at the beginning of Supplementary Note 1.

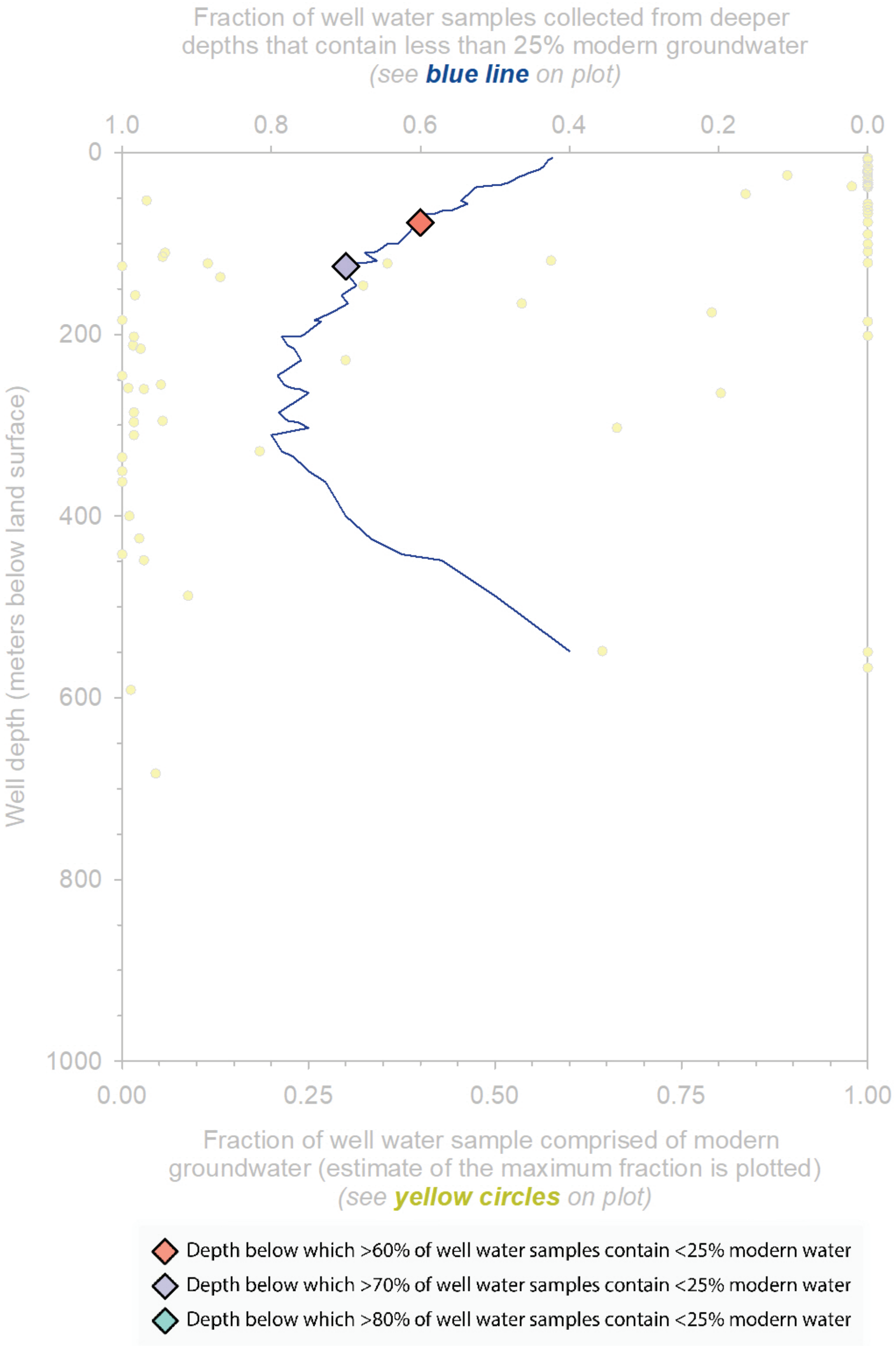

Supplementary Fig. 44. Eastern Cambrian-Ordovician Aquifers modern well water prevalence with depth. For details on symbology see the paragraph at the beginning of Supplementary Note 1.

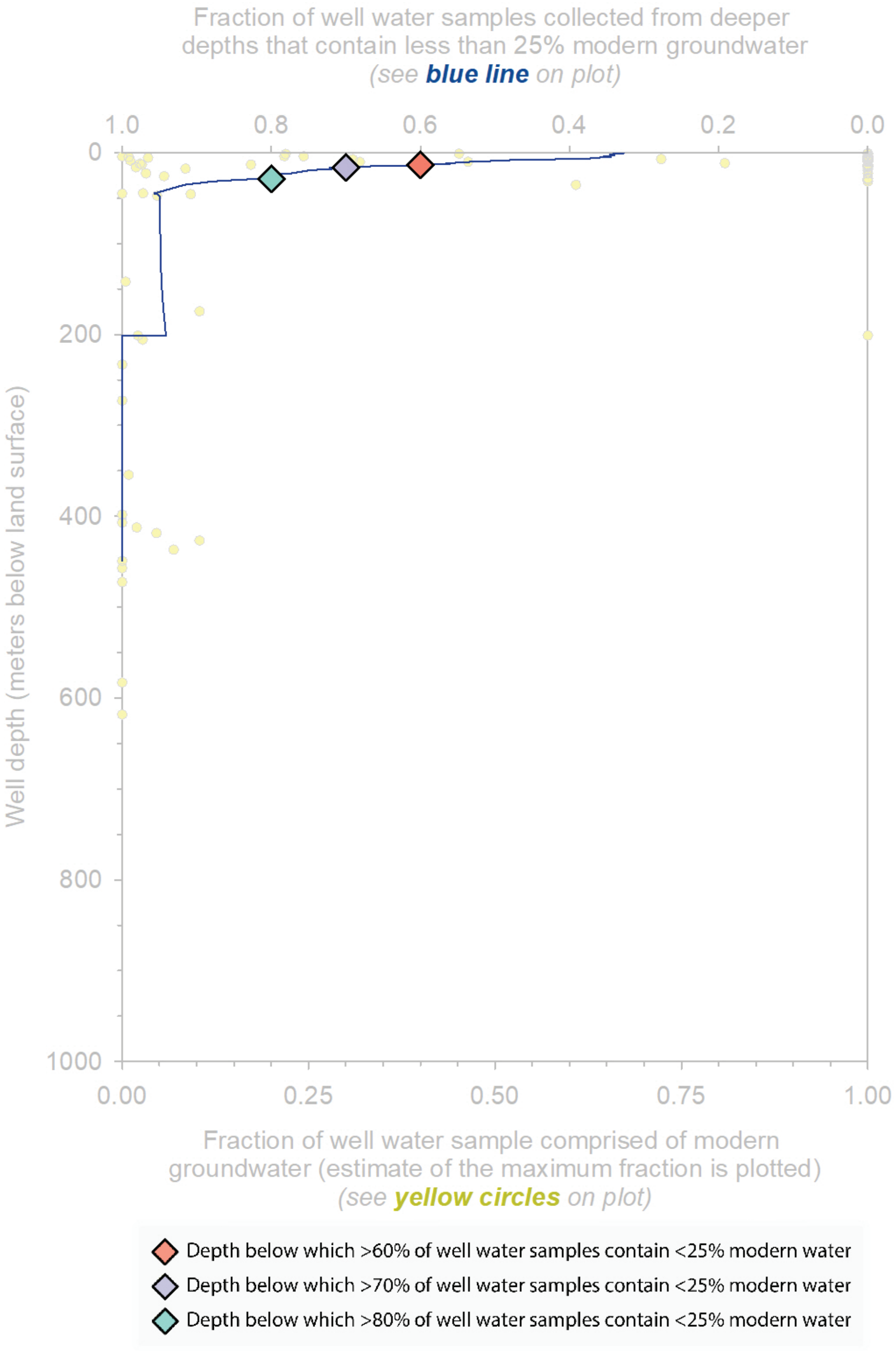

Supplementary Fig. 45. Eastern Silurian-Devonian Aquifers modern well water prevalence with depth. For details on symbology see the paragraph at the beginning of Supplementary Note 1.

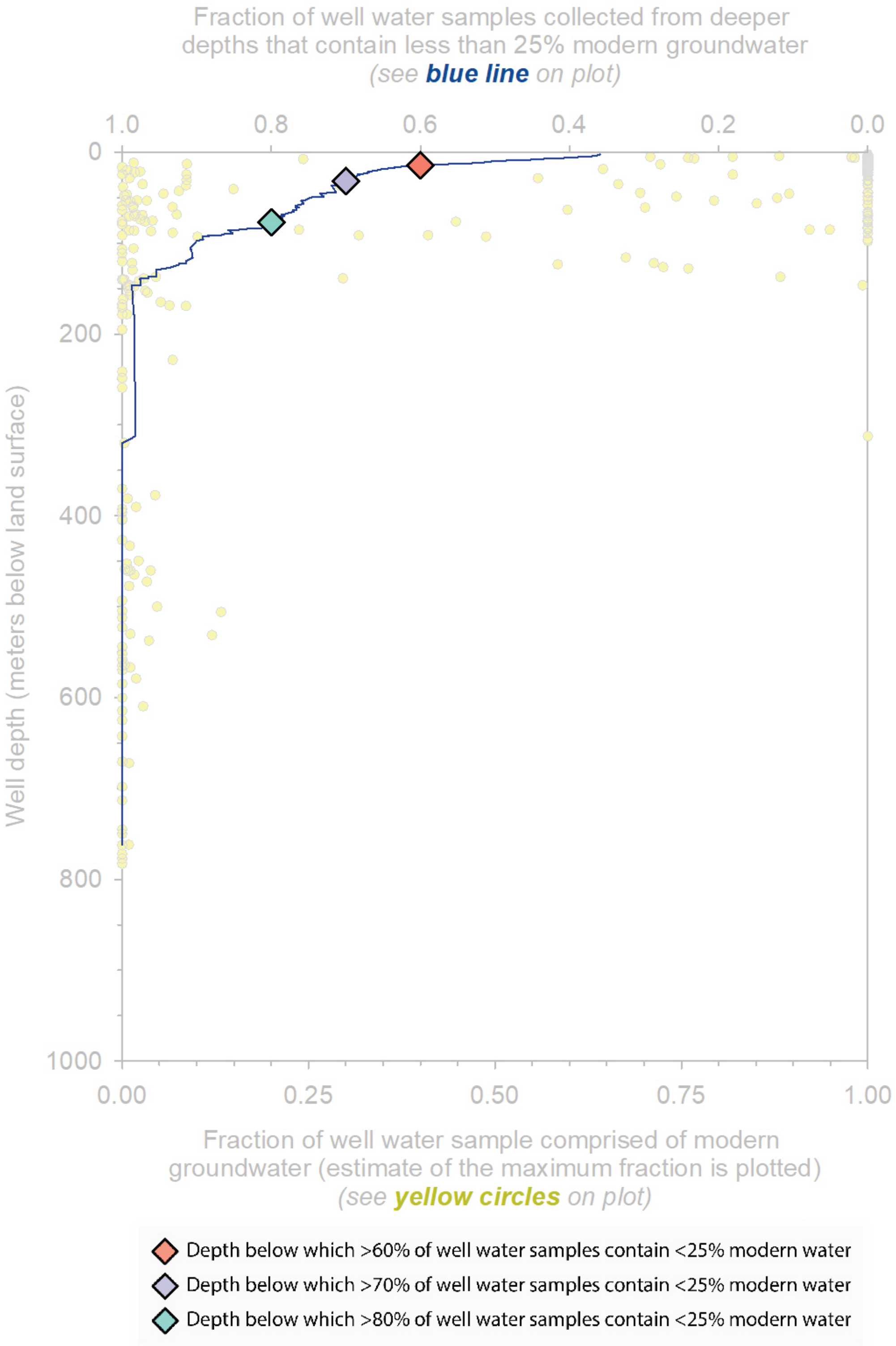

Supplementary Fig. 46. Mississippian-Silurian-Devonian Carbonates modern well water prevalence with depth. For details on symbology see the paragraph at the beginning of Supplementary Note 1.

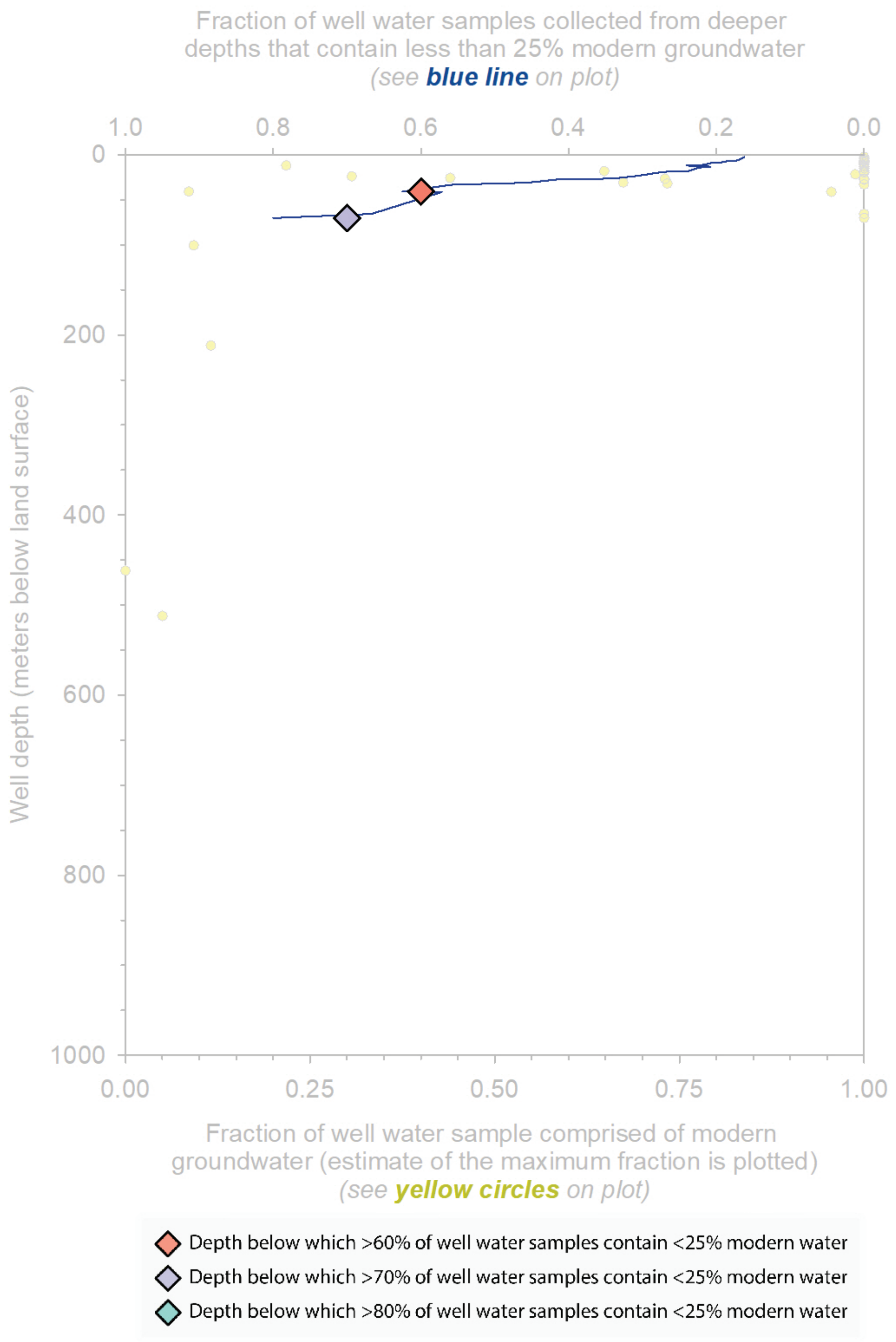

Supplementary Fig. 47. Northeast Missouri Carbonates modern well water prevalence with depth. For details on symbology see the paragraph at the beginning of Supplementary Note 1.

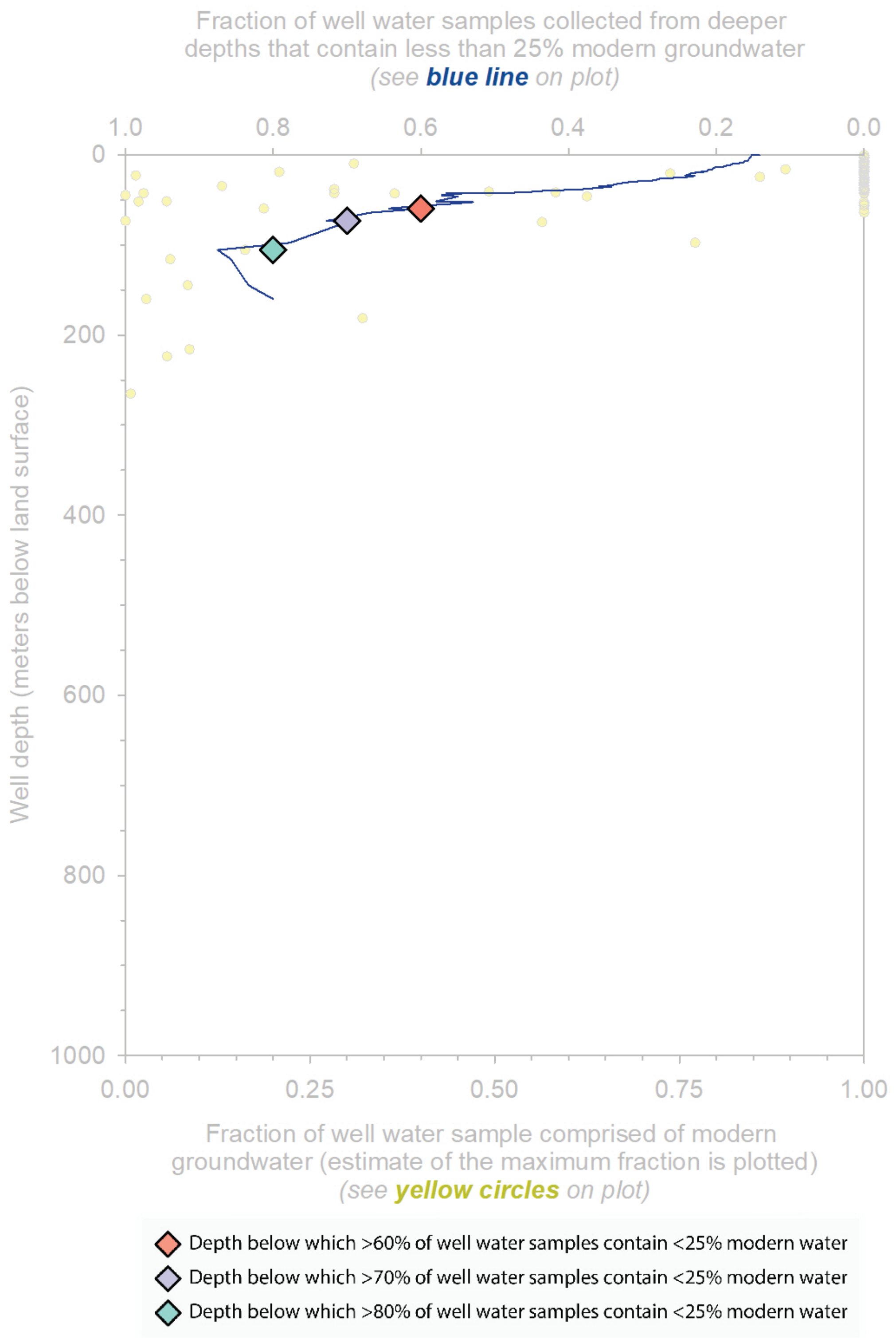

Supplementary Fig. 48. Northern Cambrian-Ordovician Aquifers modern well water prevalence with depth. For details on symbology see the paragraph at the beginning of Supplementary Note 1.

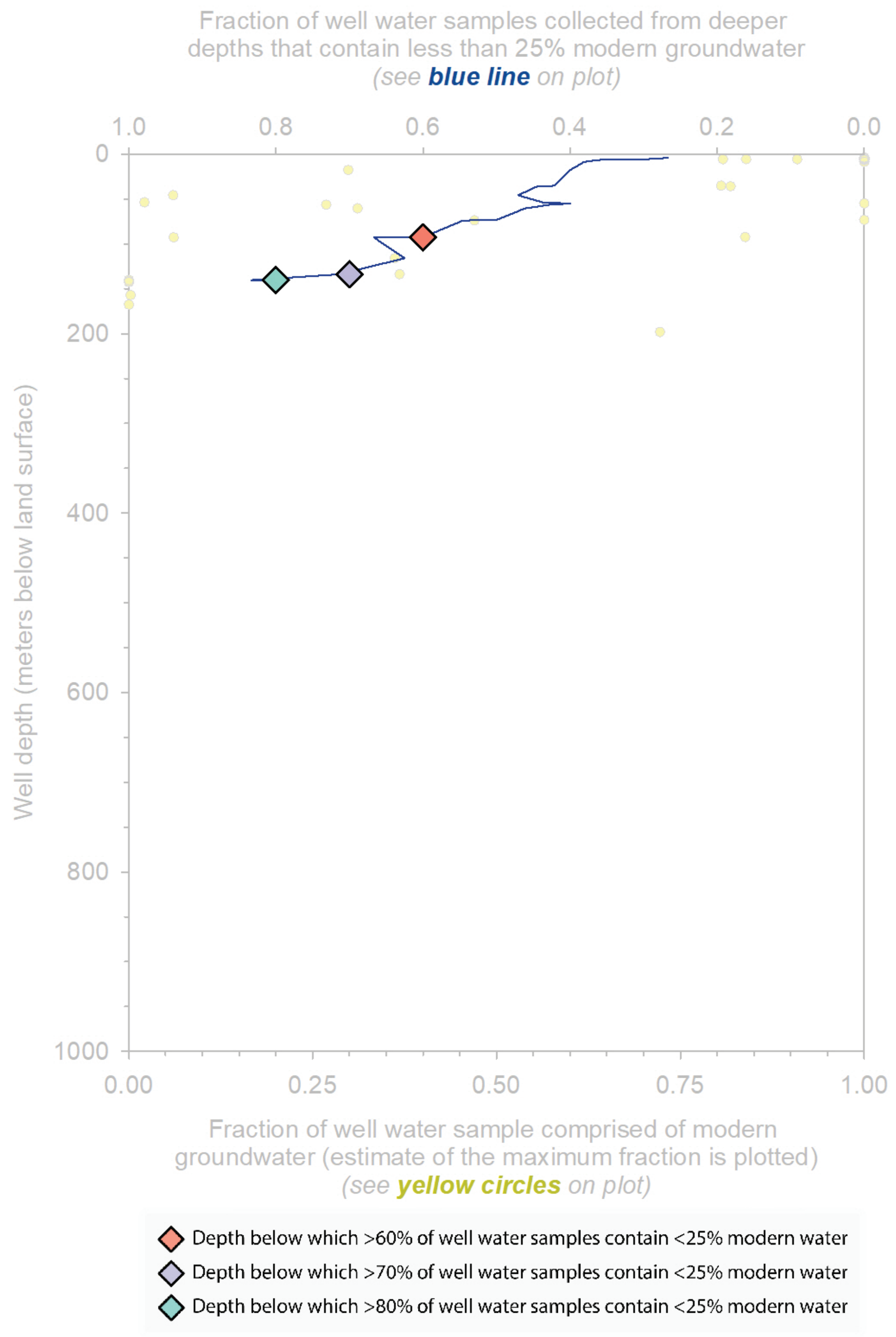

Supplementary Fig. 49. Upper Carbonate Aquifer modern well water prevalence with depth. For details on symbology see the paragraph at the beginning of Supplementary Note 1.

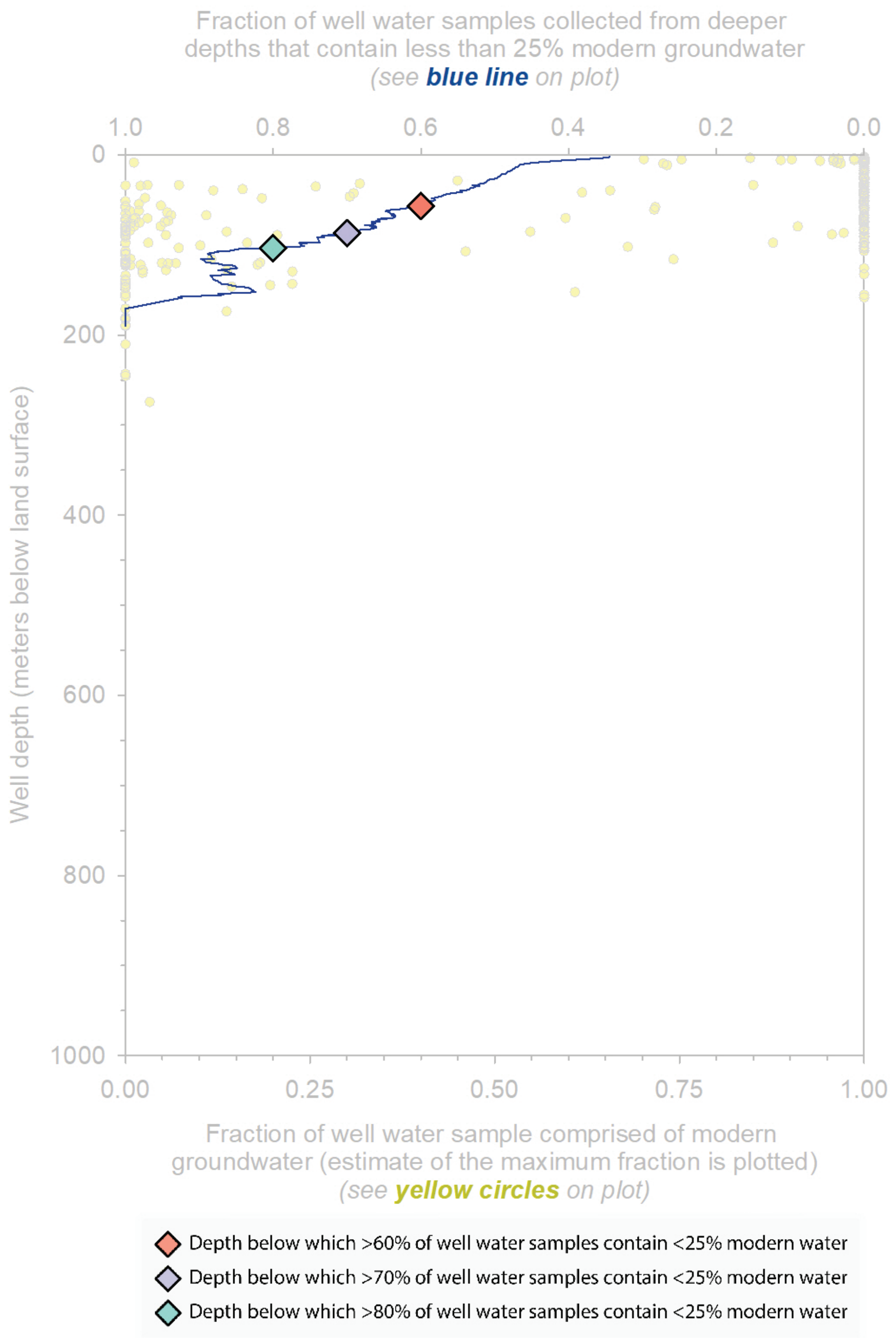

Supplementary Fig. 50. Western Cambrian-Ordovician Aquifers modern well water prevalence with depth. For details on symbology see the paragraph at the beginning of Supplementary Note 1.

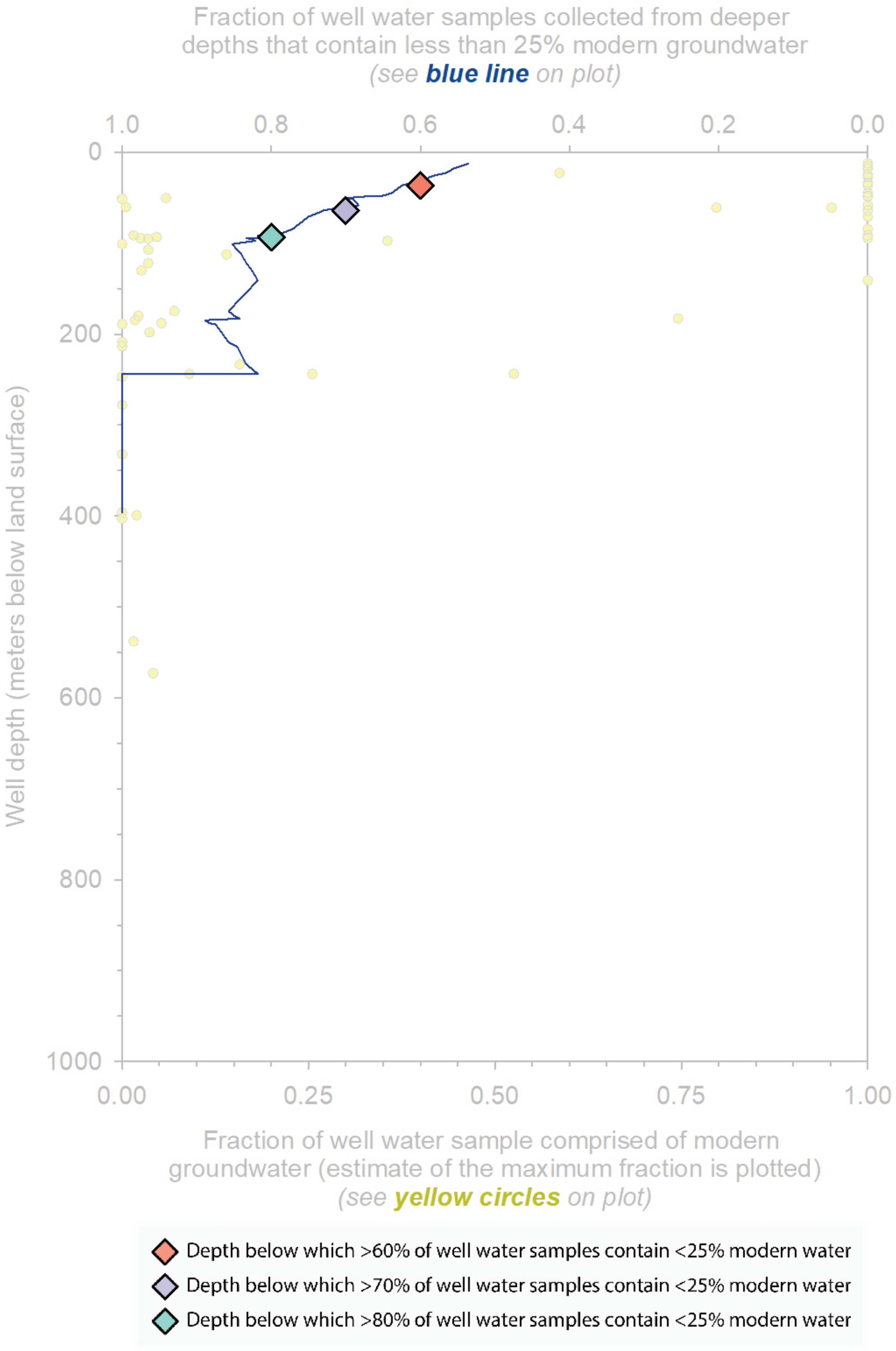

Supplementary Fig. 51. Mesilla Valley modern well water prevalence with depth. For details on symbology see the paragraph at the beginning of Supplementary Note 1.

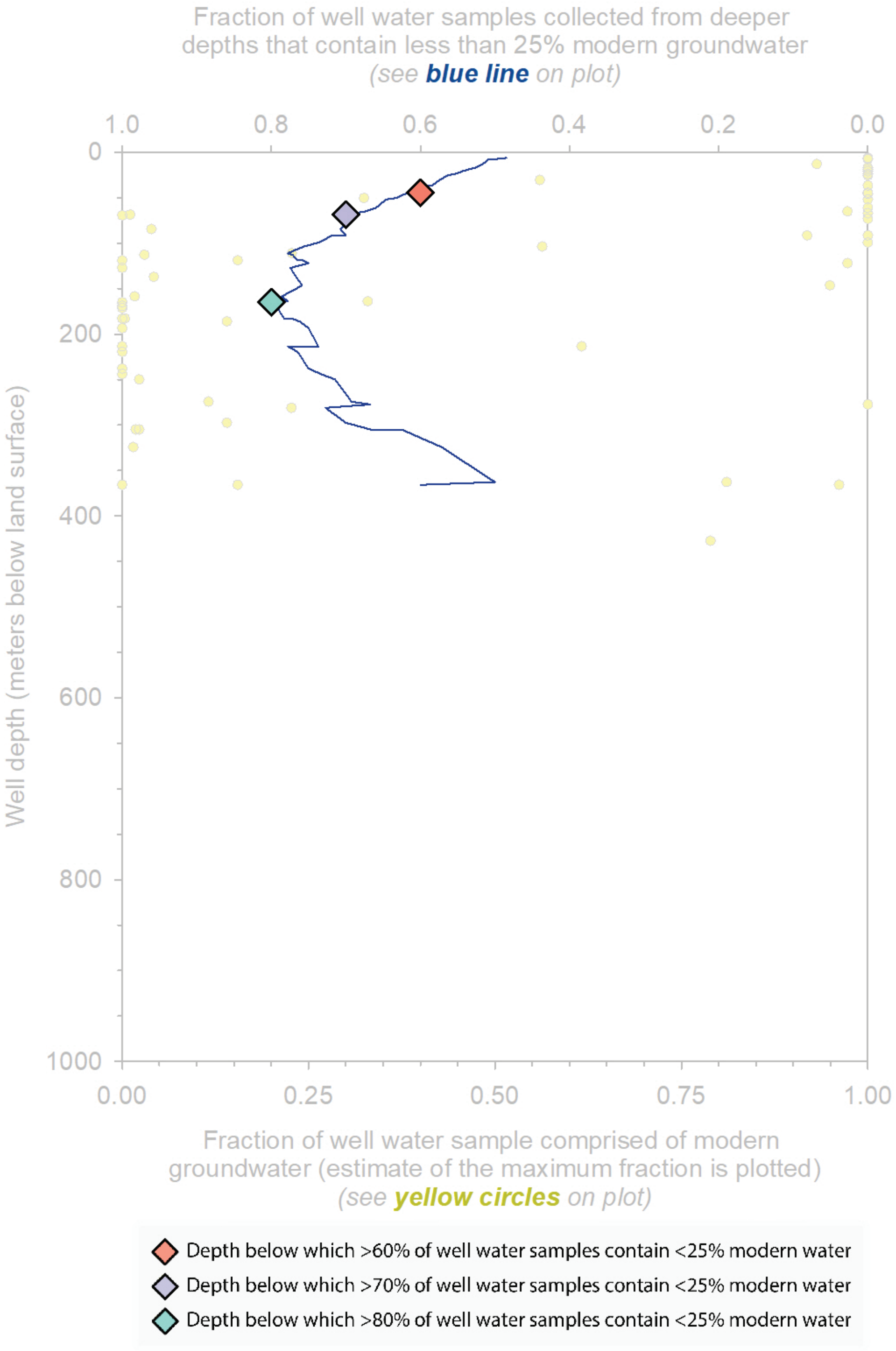

Supplementary Fig. 52. West Salt River Basin modern well water prevalence with depth. For details on symbology see the paragraph at the beginning of Supplementary Note 1.

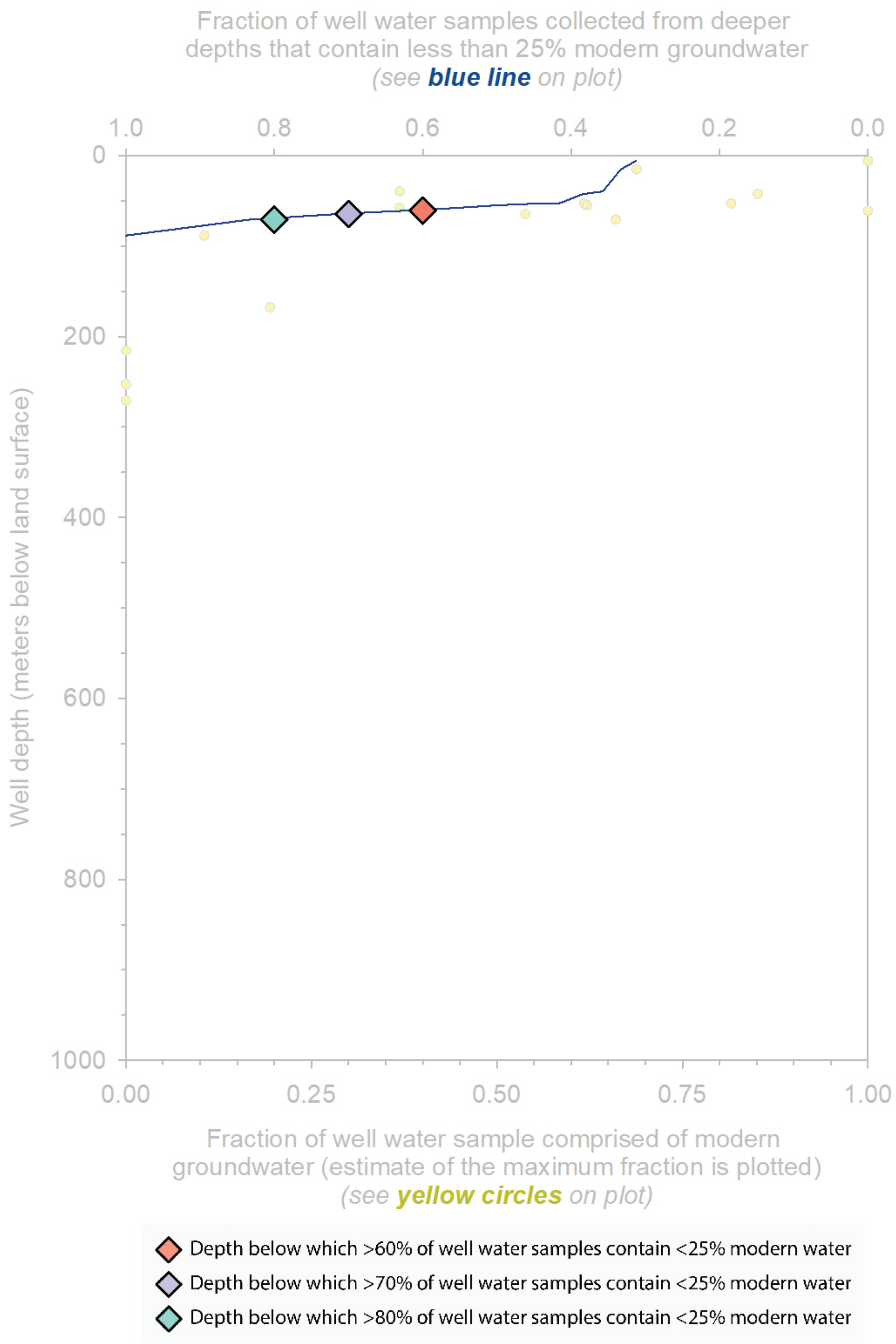

Supplementary Fig. 53. Lower Santa Ynez Valley modern well water prevalence with depth. For details on symbology see the paragraph at the beginning of Supplementary Note 1.

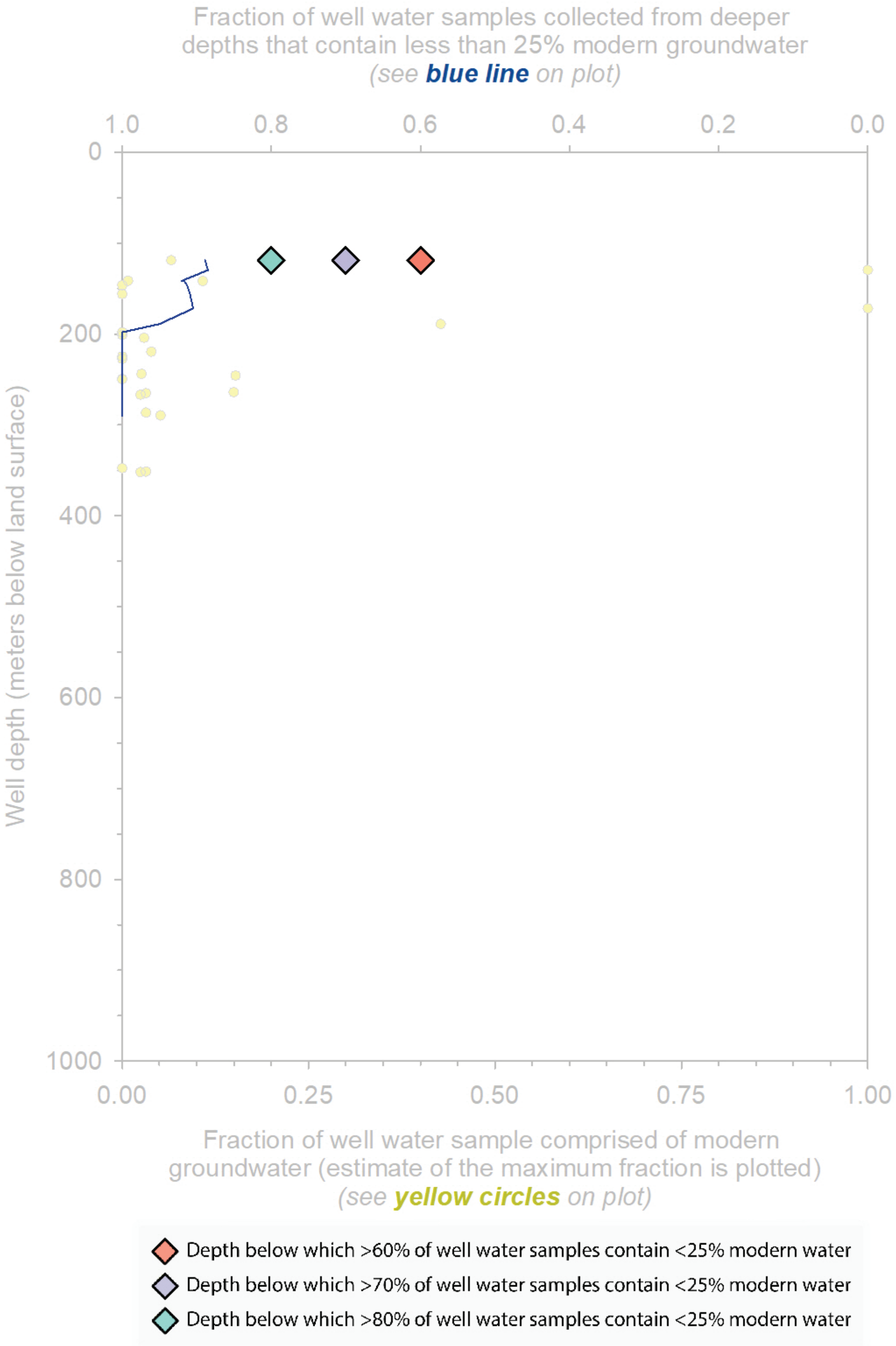

Supplementary Fig. 54. Valle de Juarez and Hueco Bolson modern well water prevalence with depth. For details on symbology see the paragraph at the beginning of Supplementary Note 1.

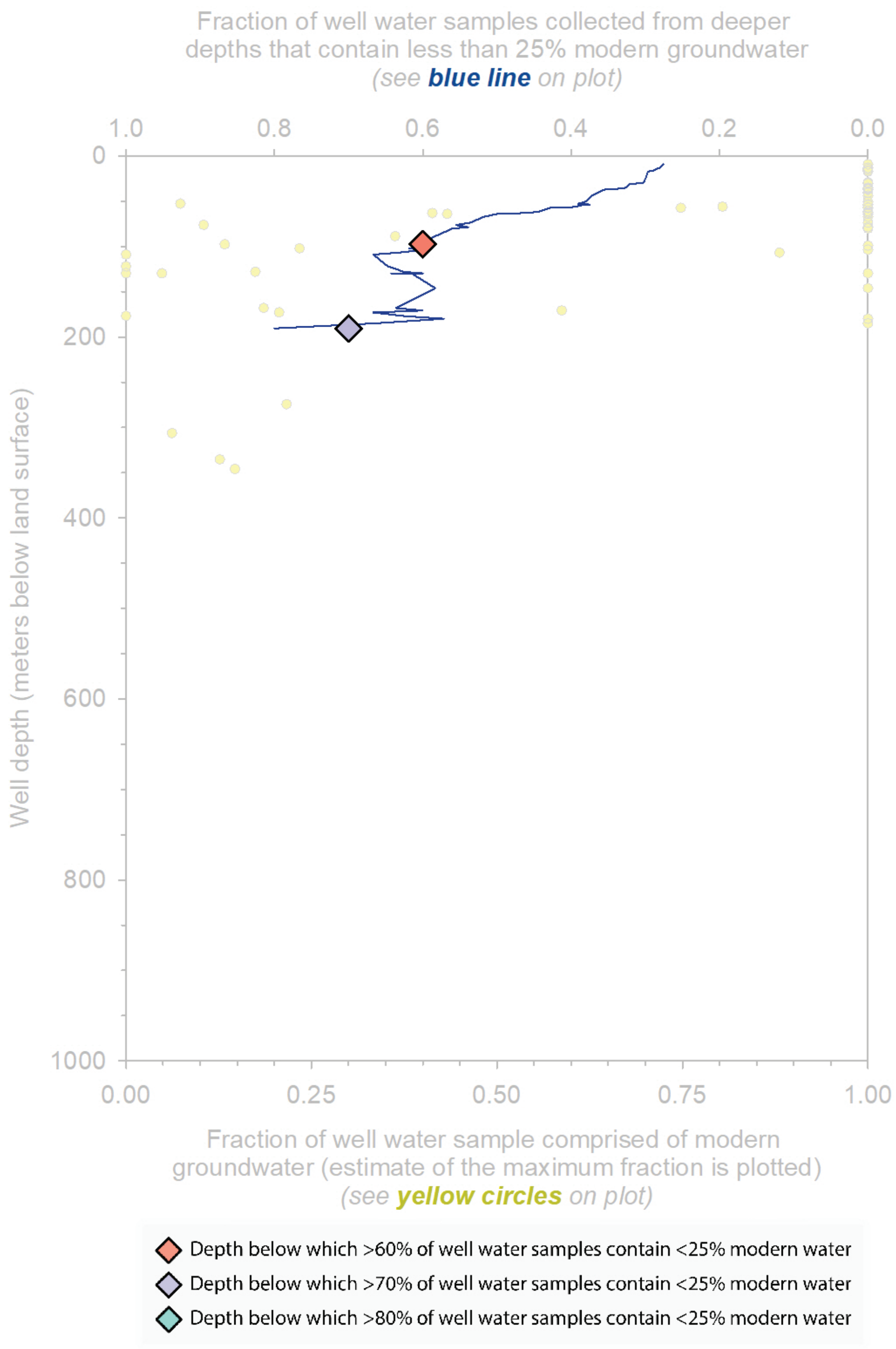

Supplementary Fig. 55. Boise Valley and Homedale Murphy Area modern well water prevalence with depth. For details on symbology see the paragraph at the beginning of Supplementary Note 1.

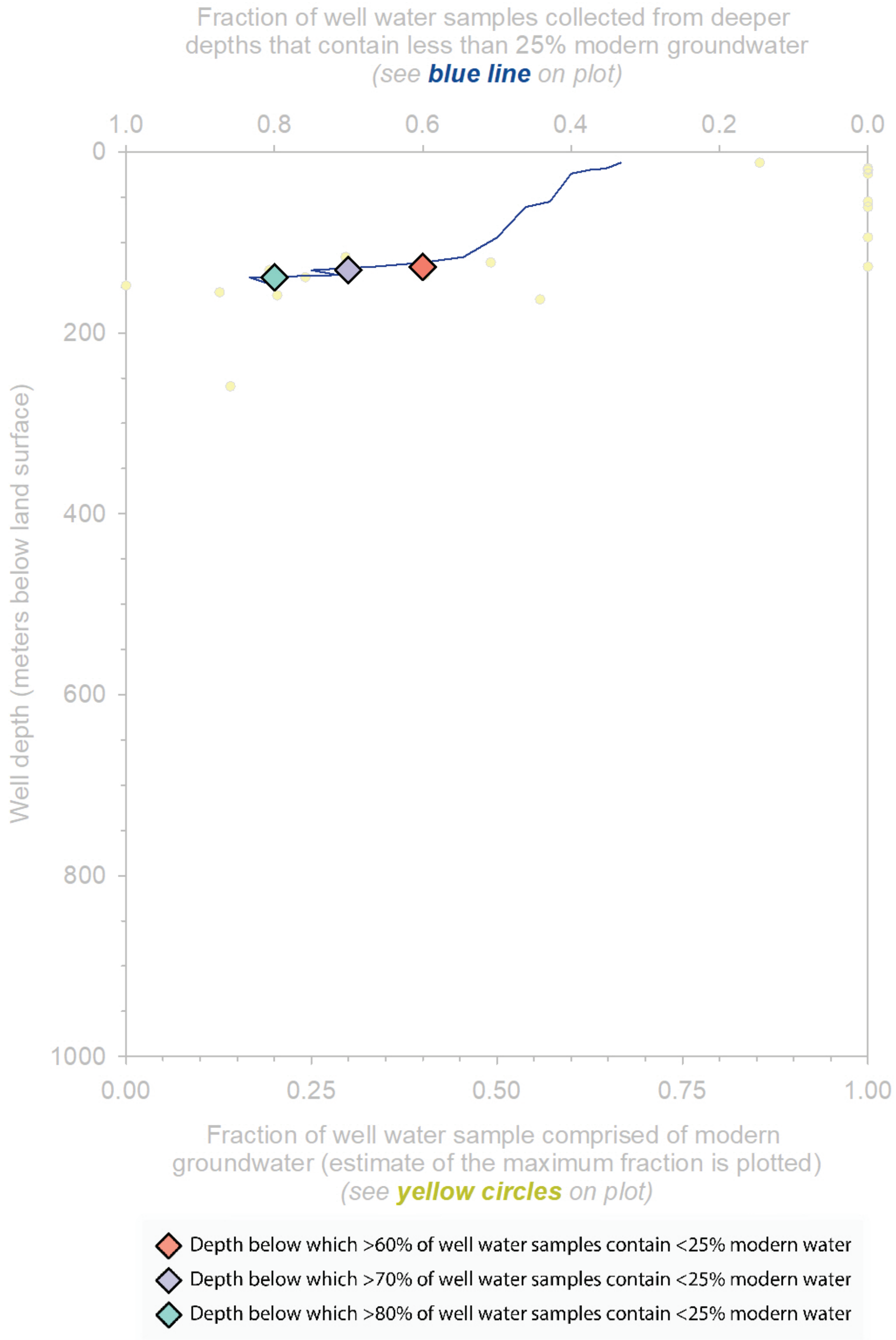

Supplementary Fig. 56. Mountain Home Plateau modern well water prevalence with depth. For details on symbology see the paragraph at the beginning of Supplementary Note 1.

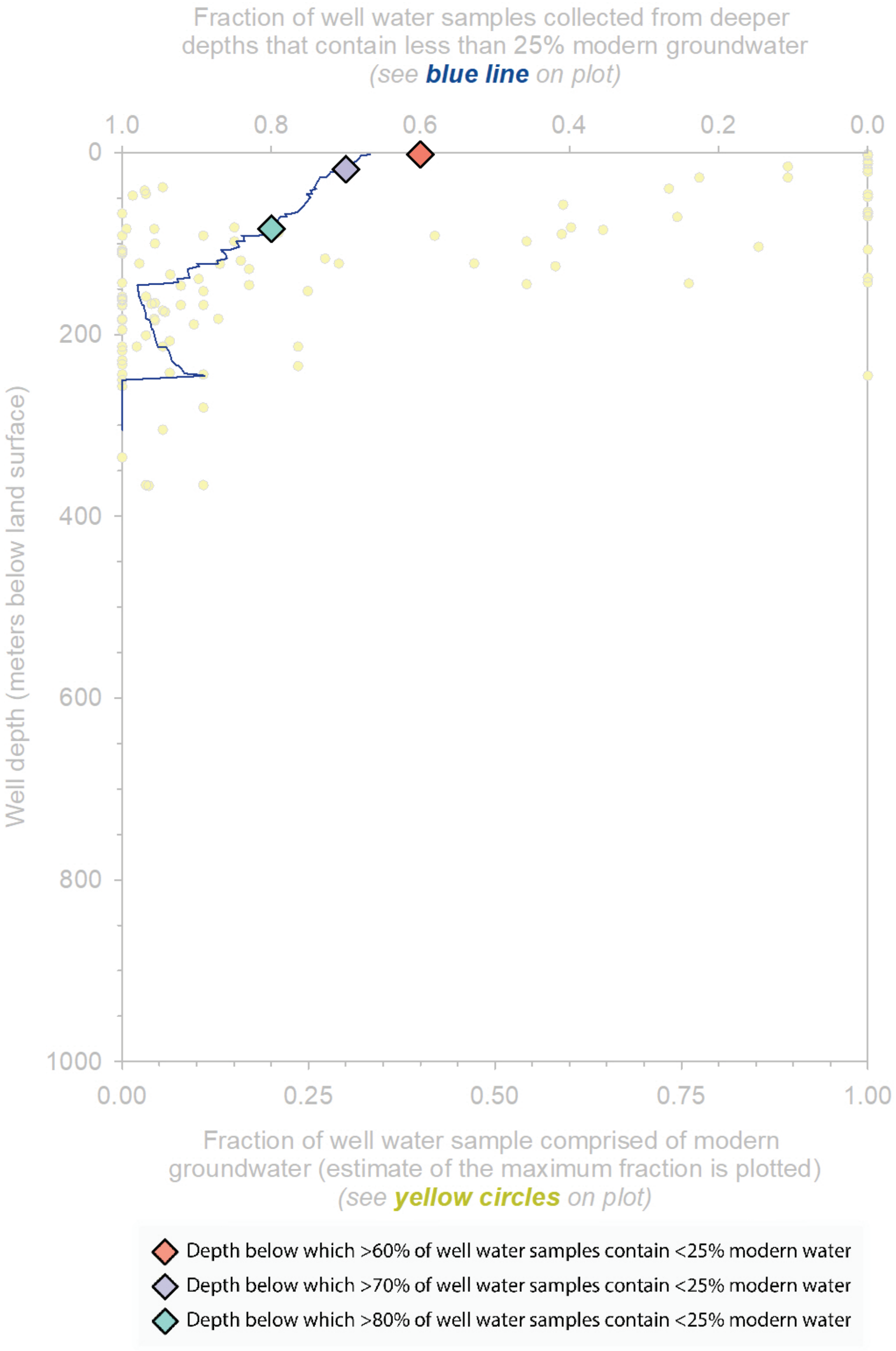

Supplementary Fig. 57. Antelope Valley modern well water prevalence with depth. For details on symbology see the paragraph at the beginning of Supplementary Note 1.

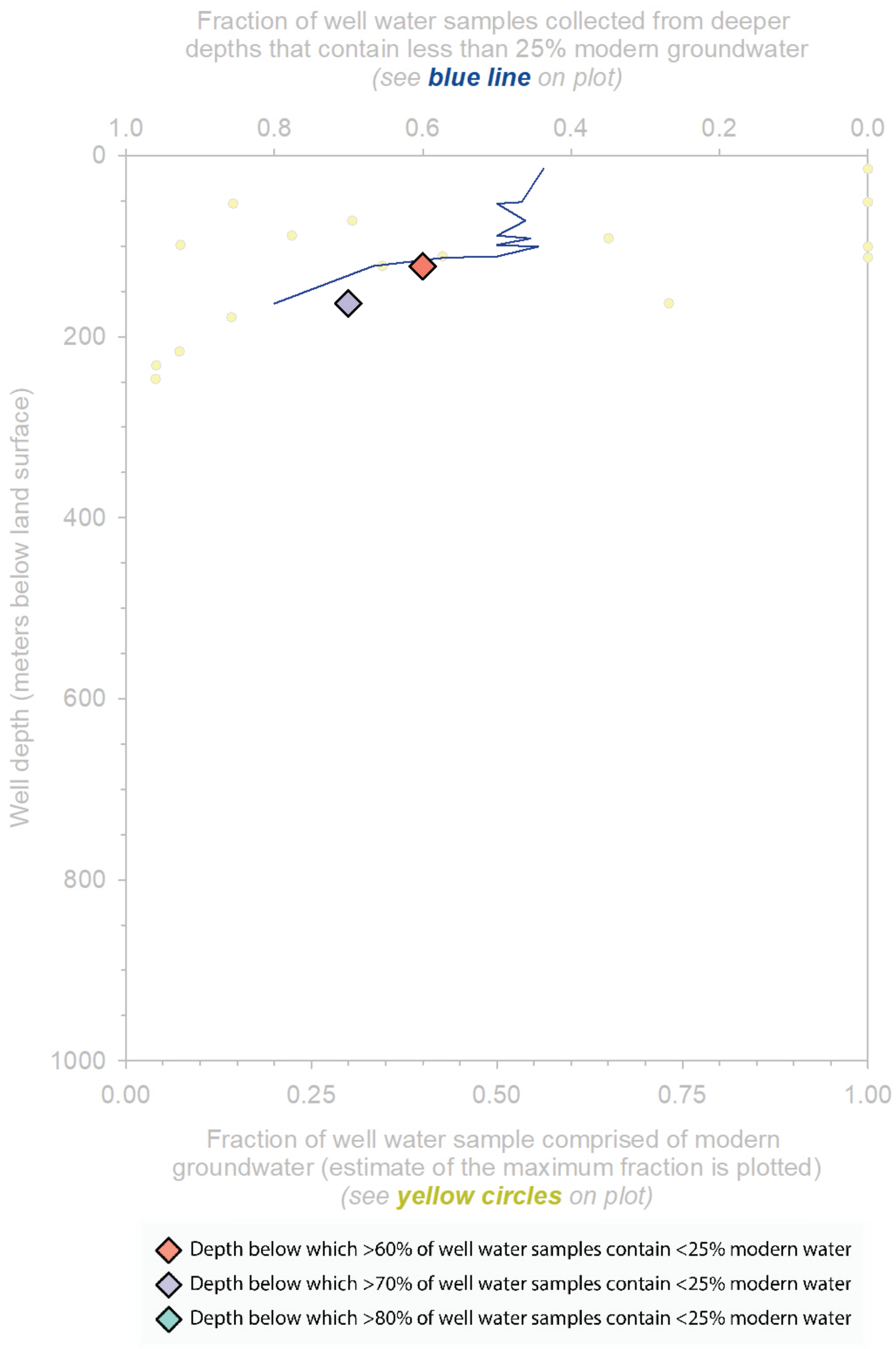

Supplementary Fig. 58. Big Bear Valley modern well water prevalence with depth. For details on symbology see the paragraph at the beginning of Supplementary Note 1.

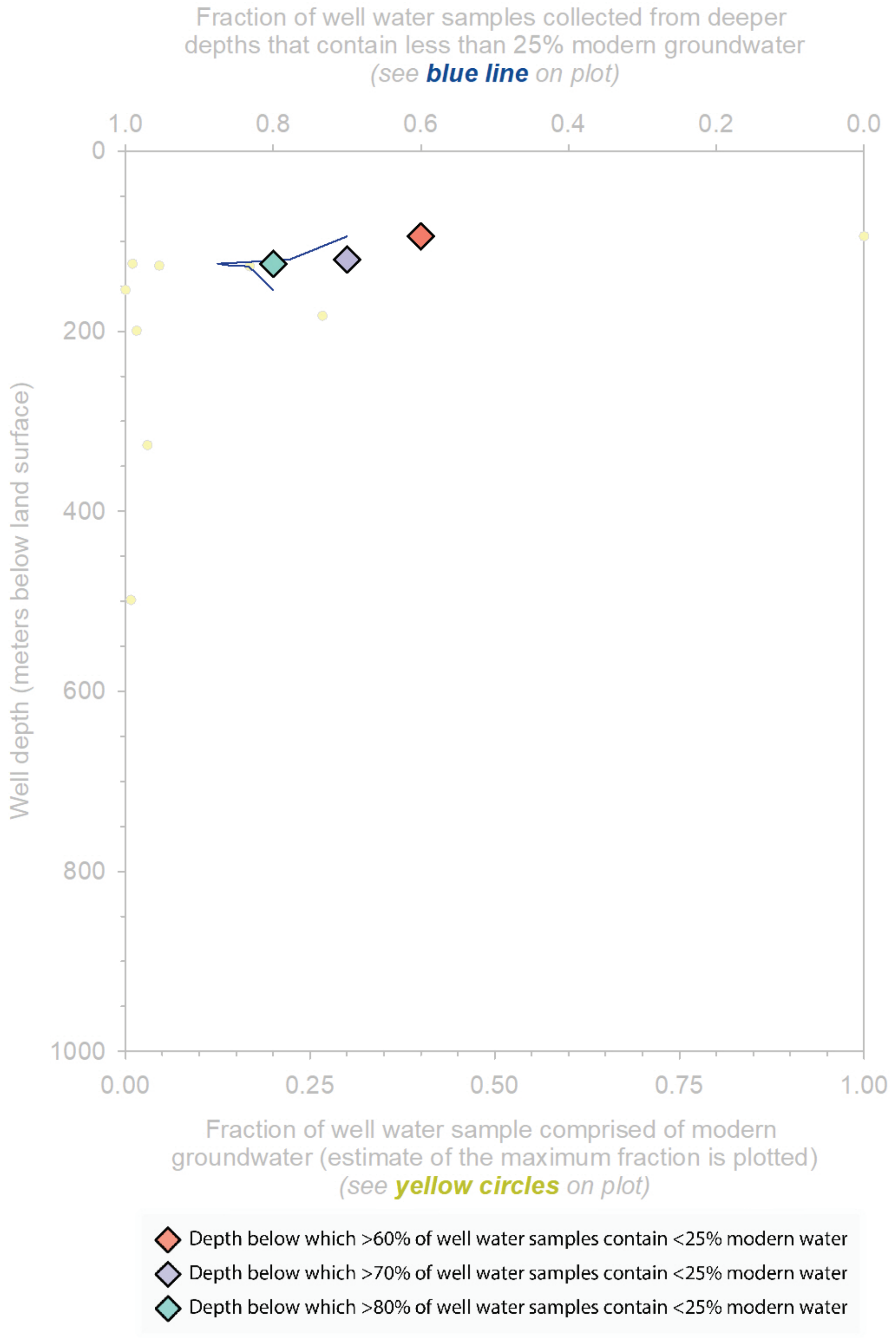

Supplementary Fig. 59. Big Chino Valley modern well water prevalence with depth. For details on symbology see the paragraph at the beginning of Supplementary Note 1.

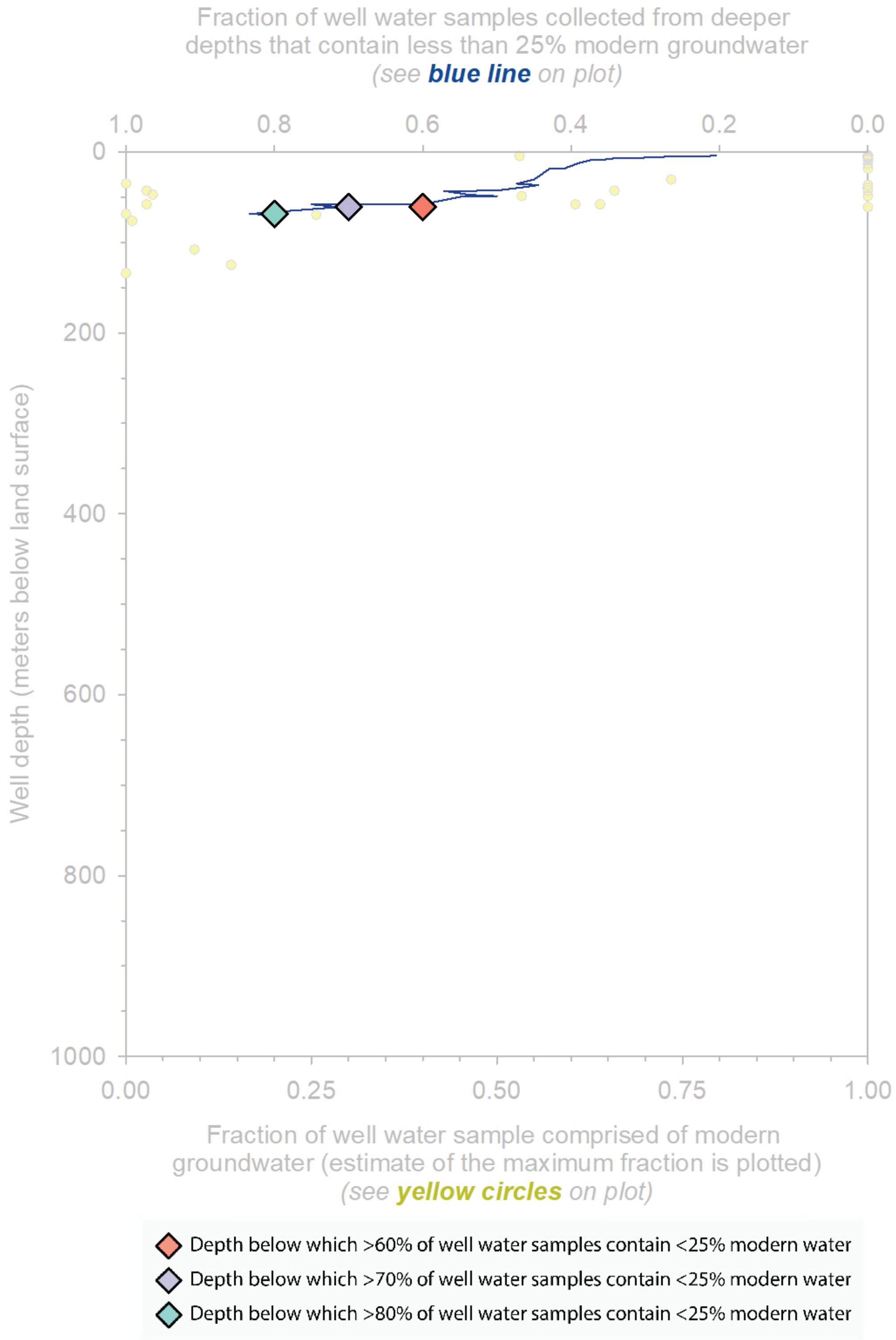

Supplementary Fig. 60. Bighorn Basin modern well water prevalence with depth. For details on symbology see the paragraph at the beginning of Supplementary Note 1.

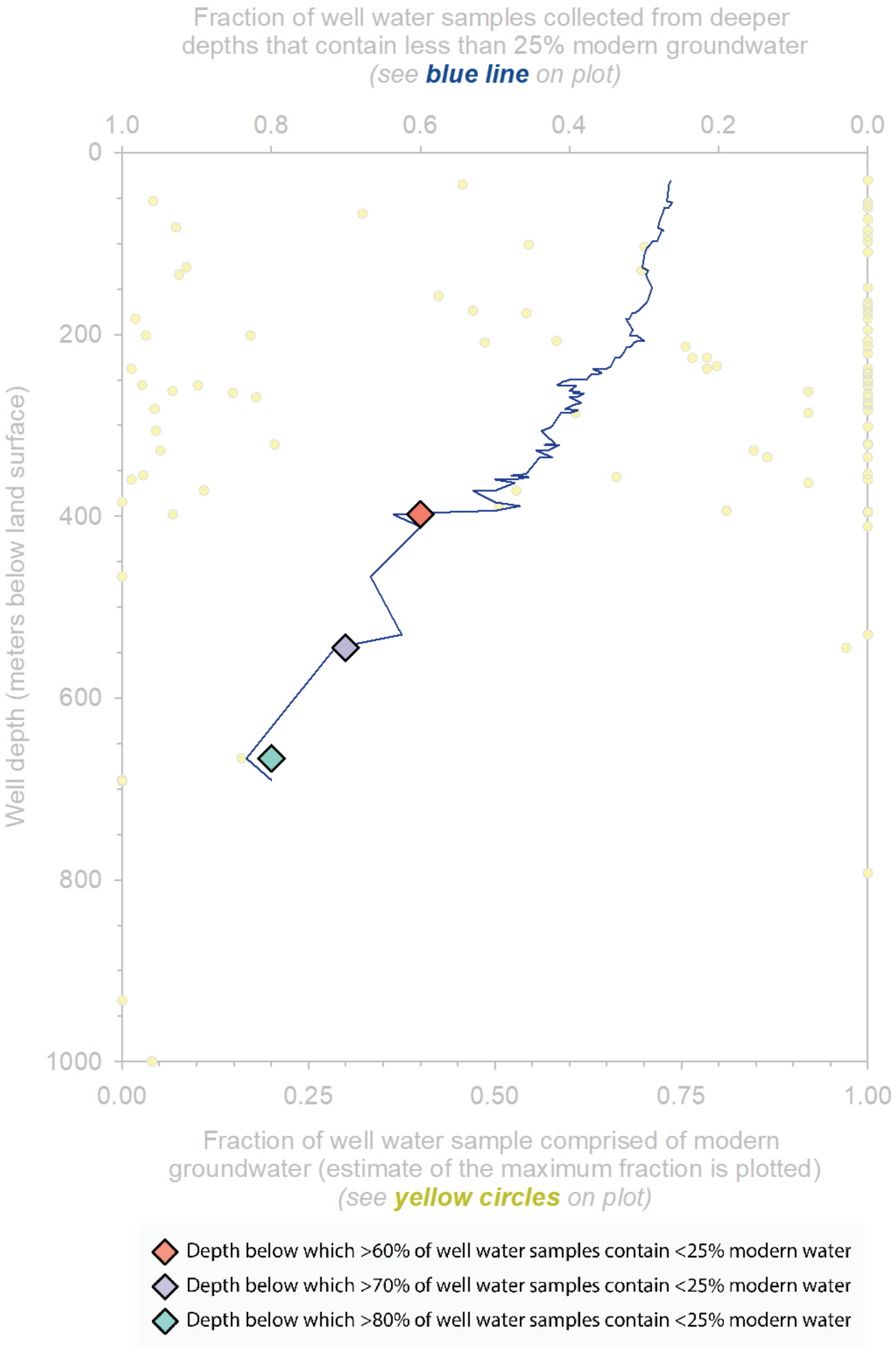

Supplementary Fig. 61. Black Hills Uplift modern well water prevalence with depth. For details on symbology see the paragraph at the beginning of Supplementary Note 1.

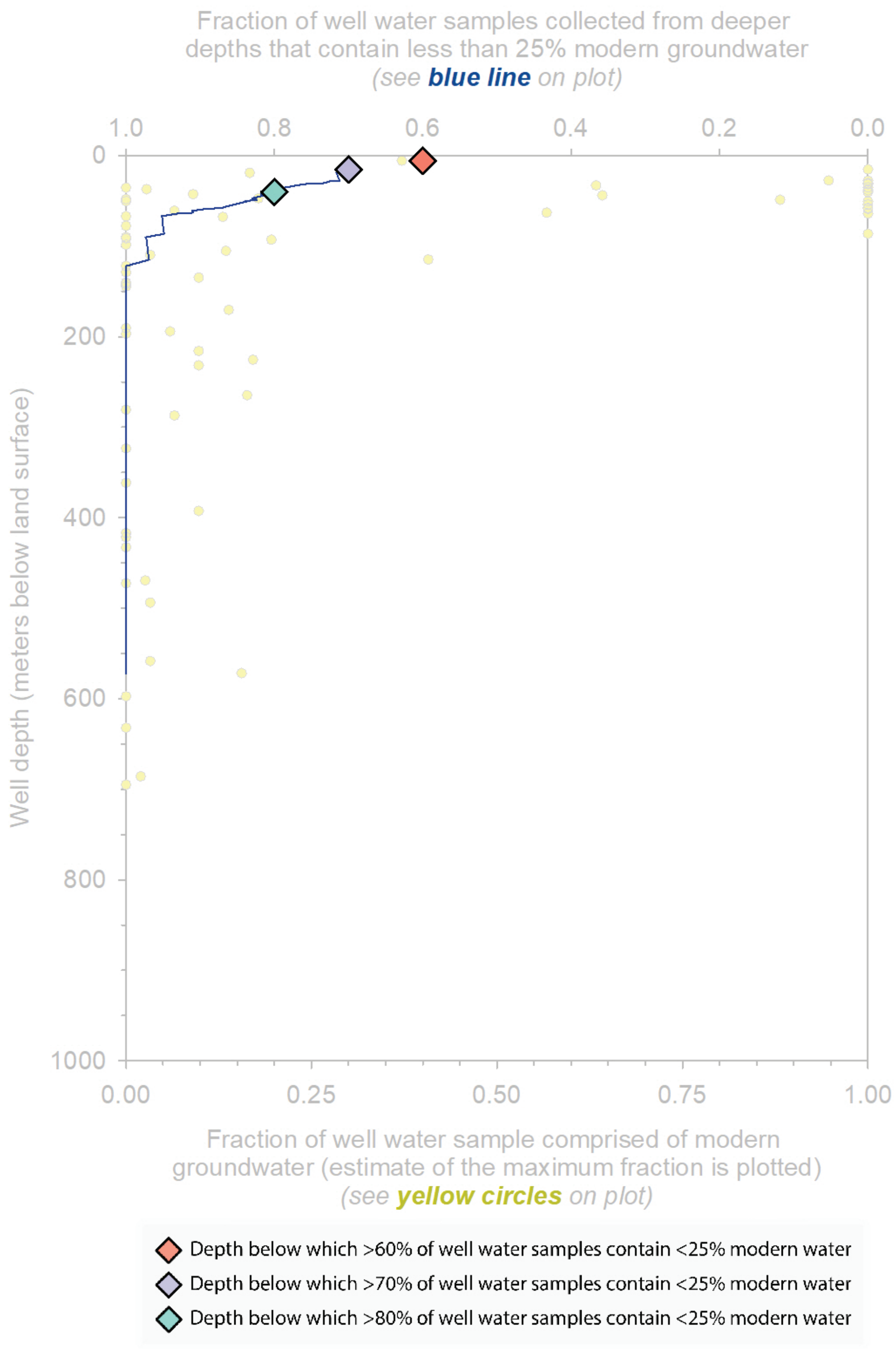

Supplementary Fig. 62. Black Warrior River Aquifer System (Eutaw and McShan Formations and Tuscaloosa Group) modern well water prevalence with depth. For details on symbology see the paragraph at the beginning of Supplementary Note 1.

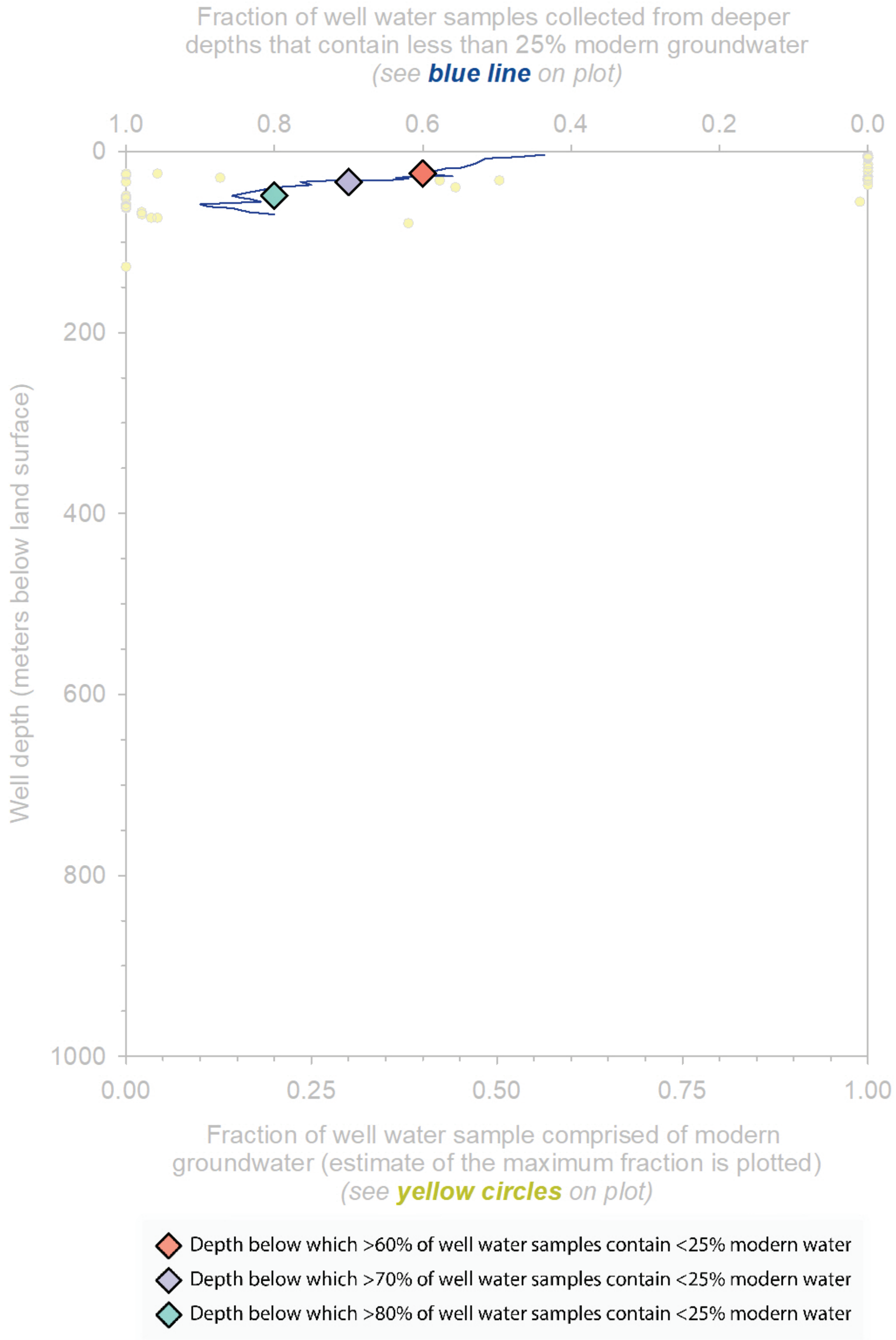

Supplementary Fig. 63. Castle Hayne Aquifer modern well water prevalence with depth.
For details on symbology see the paragraph at the beginning of Supplementary Note 1.

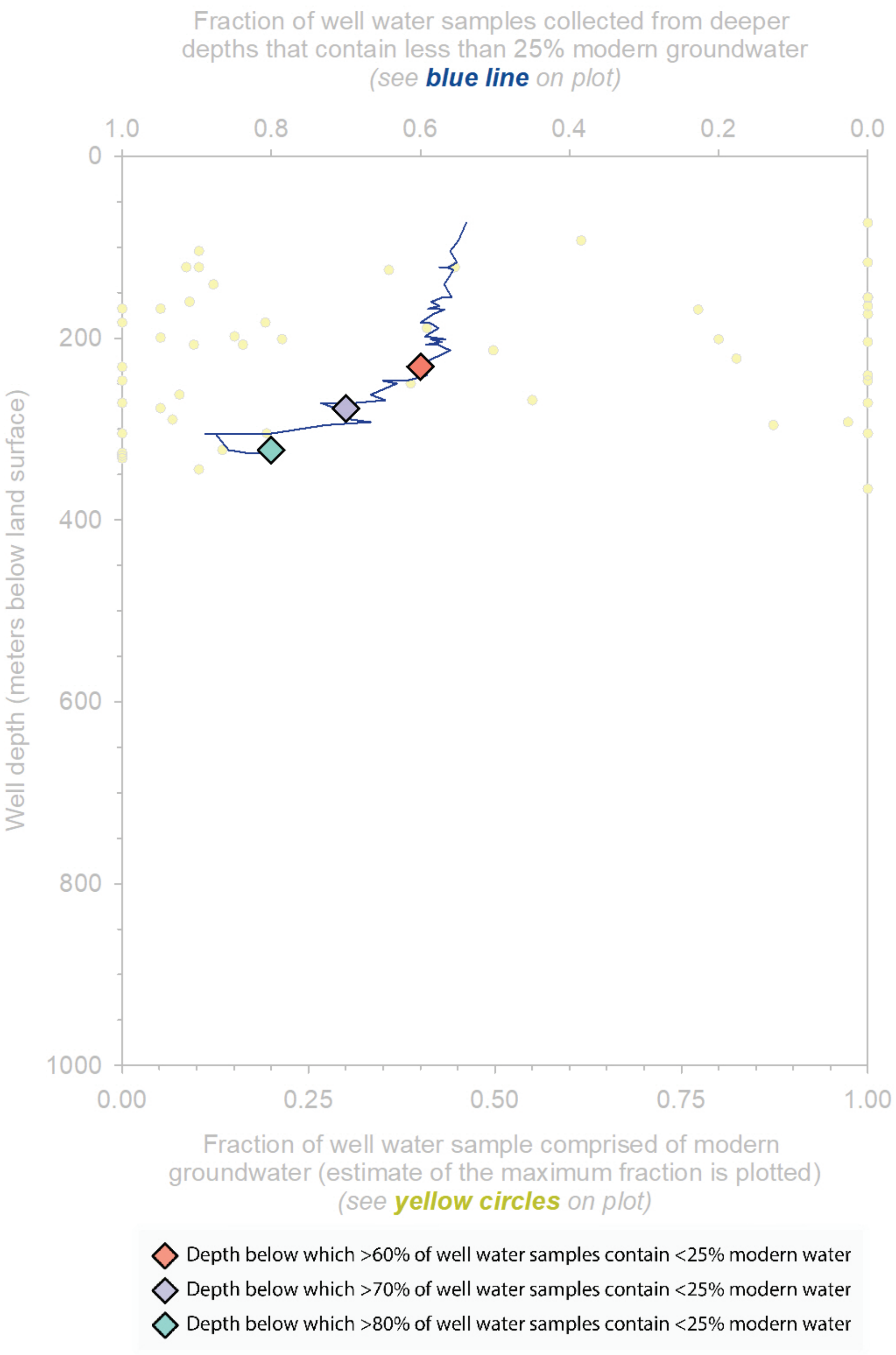

Supplementary Fig. 64. Coachella Valley modern well water prevalence with depth. For details on symbology see the paragraph at the beginning of Supplementary Note 1.

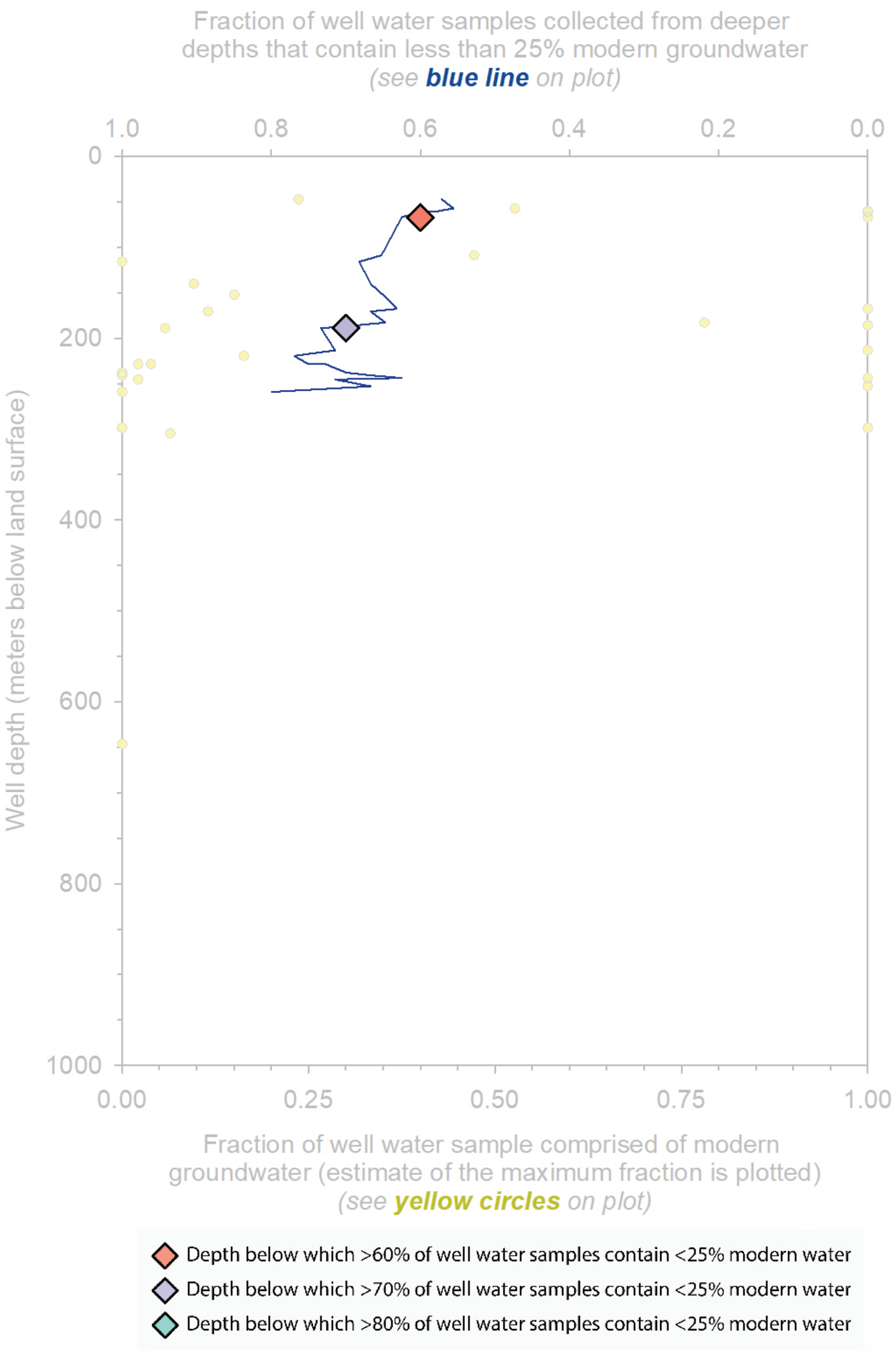

Supplementary Fig. 65. Cuyama Valley modern well water prevalence with depth. For details on symbology see the paragraph at the beginning of Supplementary Note 1.

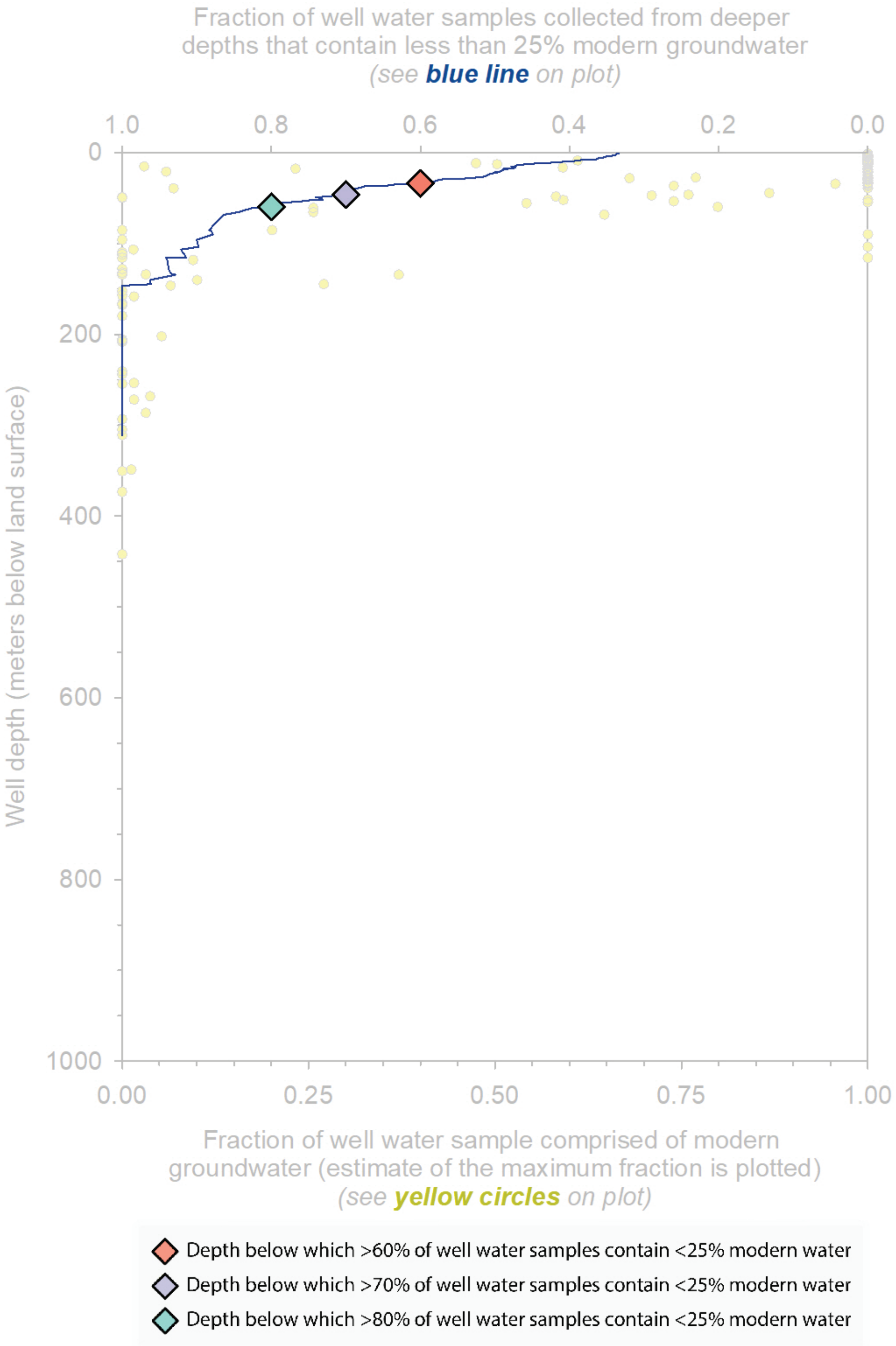

Supplementary Fig. 66. Denver Basin modern well water prevalence with depth. For details on symbology see the paragraph at the beginning of Supplementary Note 1.

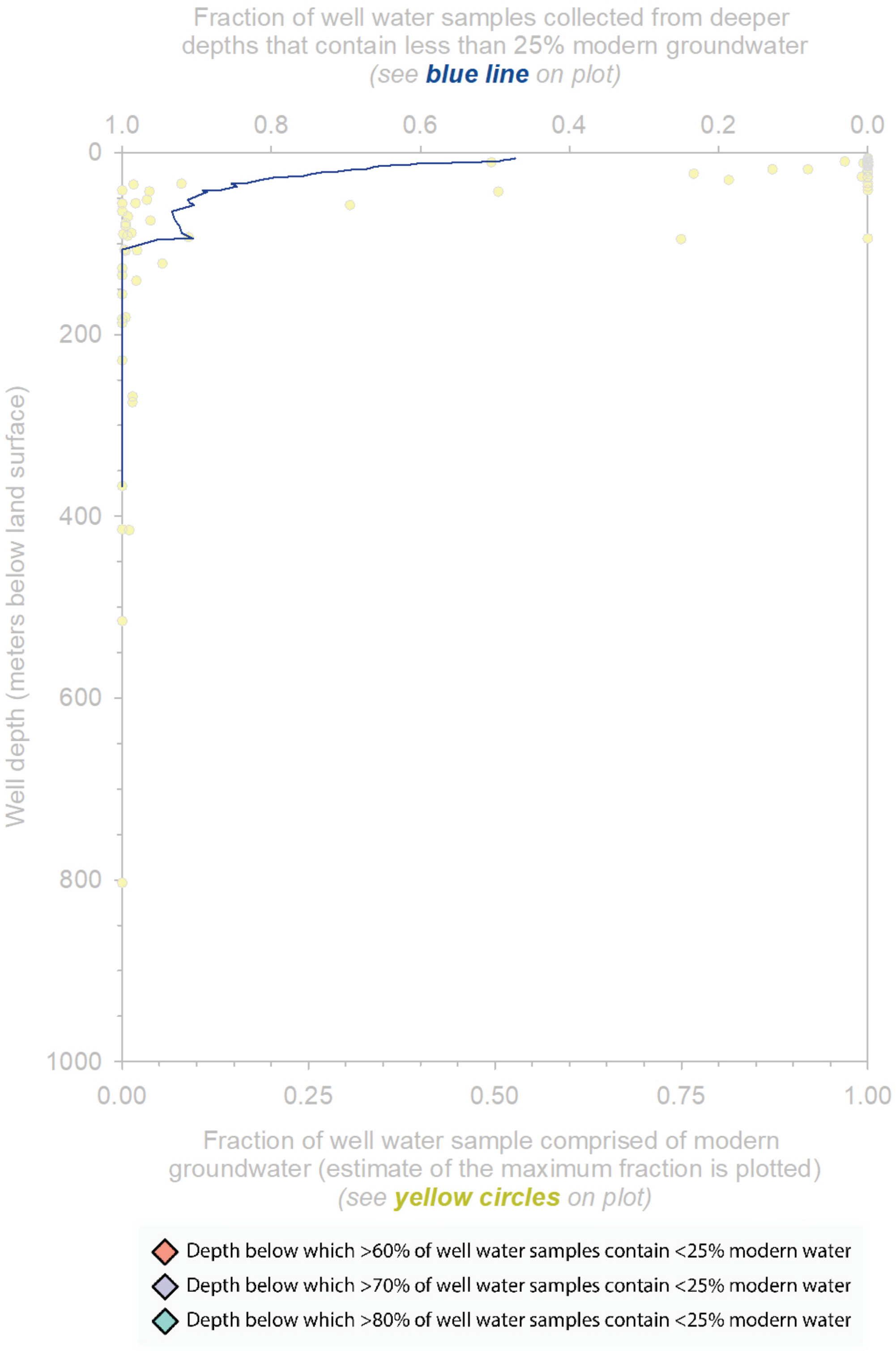

Supplementary Fig. 67. Eastern Dakota Aquifer modern well water prevalence with depth. For details on symbology see the paragraph at the beginning of Supplementary Note 1.

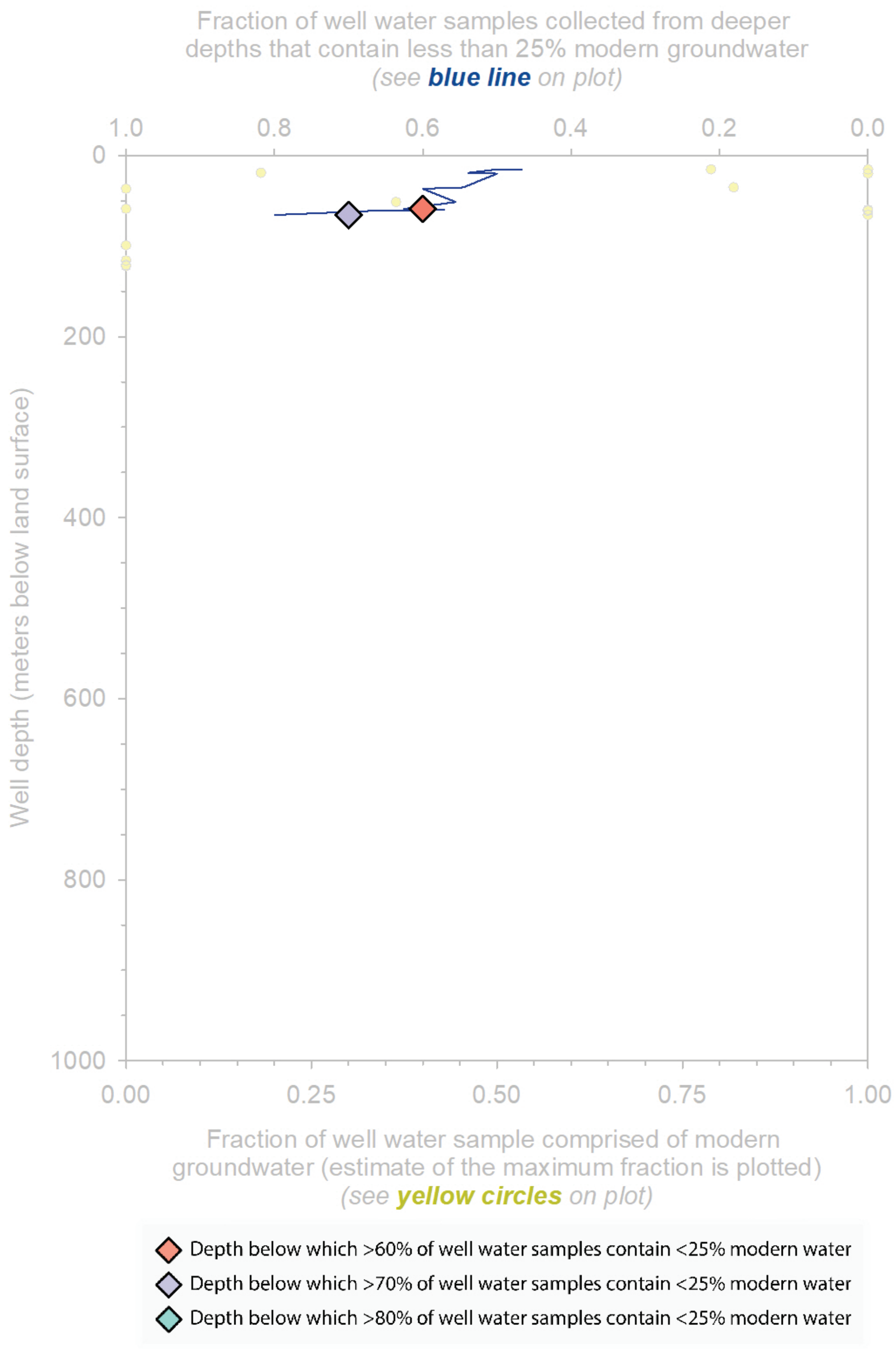

Supplementary Fig. 68. Eureka and Eel River and Mad River Plains modern well water prevalence with depth. For details on symbology see the paragraph at the beginning of Supplementary Note 1.

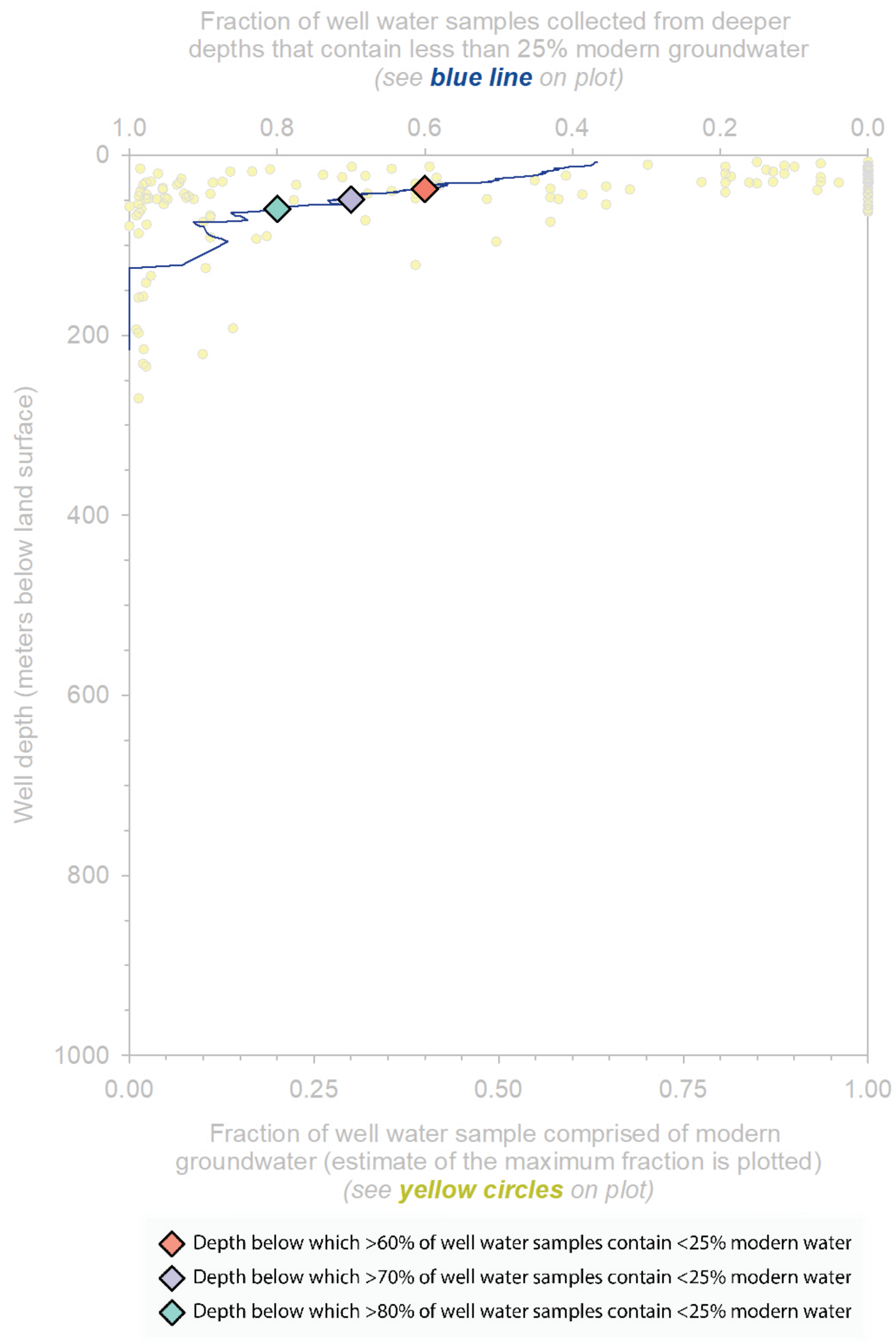

Supplementary Fig. 69. Garber-Wellington Aquifer modern well water prevalence with depth. For details on symbology see the paragraph at the beginning of Supplementary Note 1.

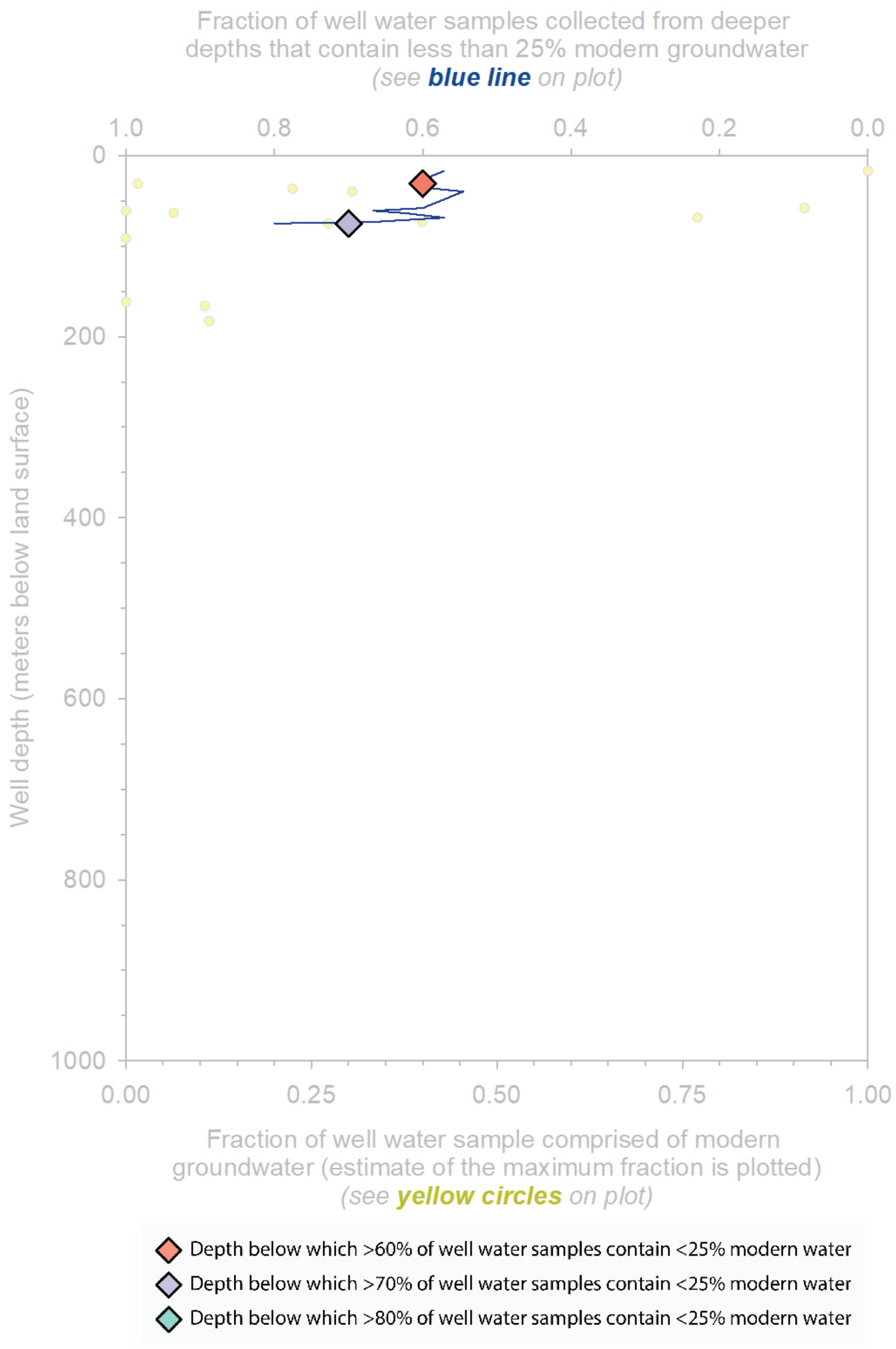

Supplementary Fig. 70. Honey Lake Valley modern well water prevalence with depth. For details on symbology see the paragraph at the beginning of Supplementary Note 1.

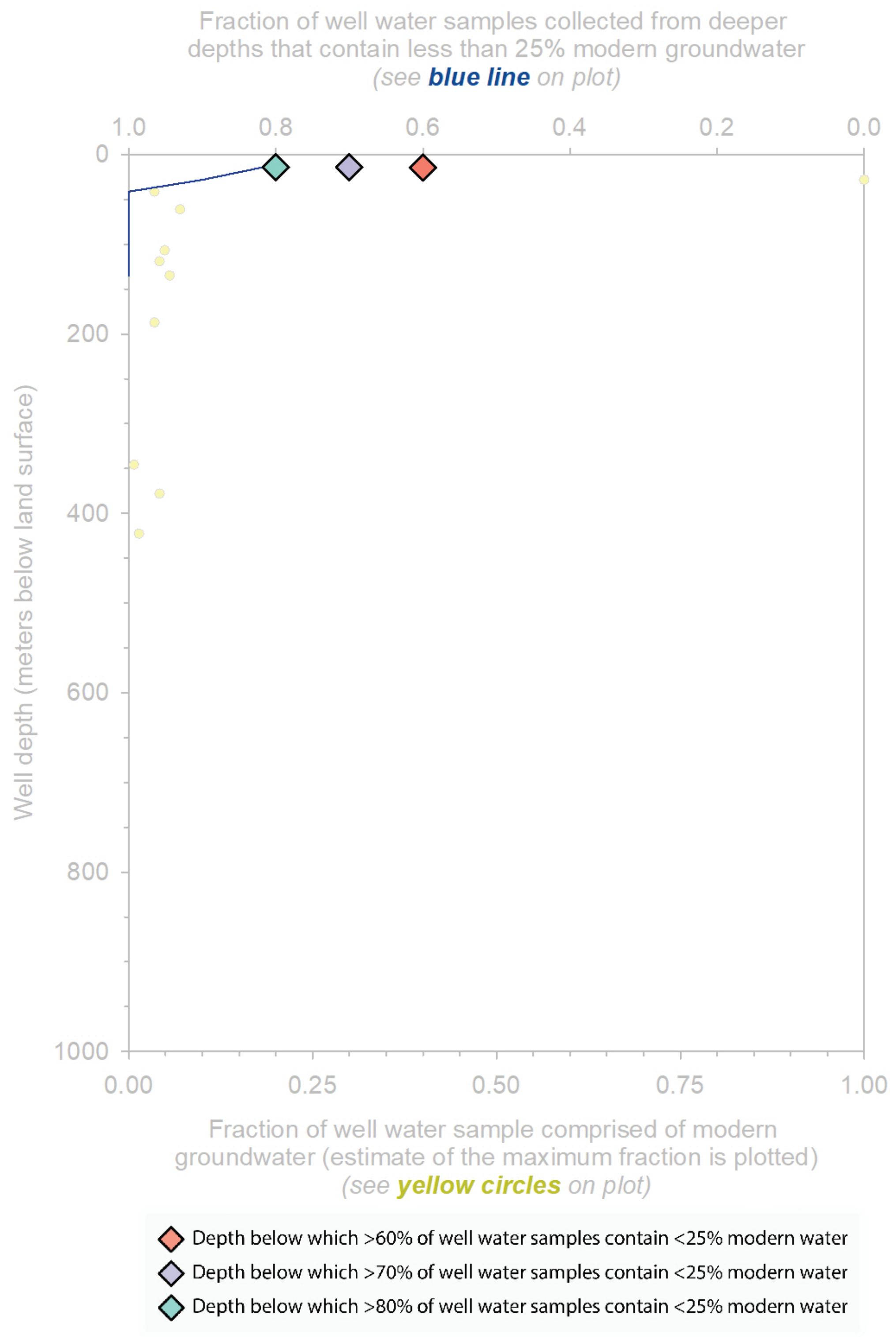

Supplementary Fig. 71. Judith Basin modern well water prevalence with depth. For details on symbology see the paragraph at the beginning of Supplementary Note 1.

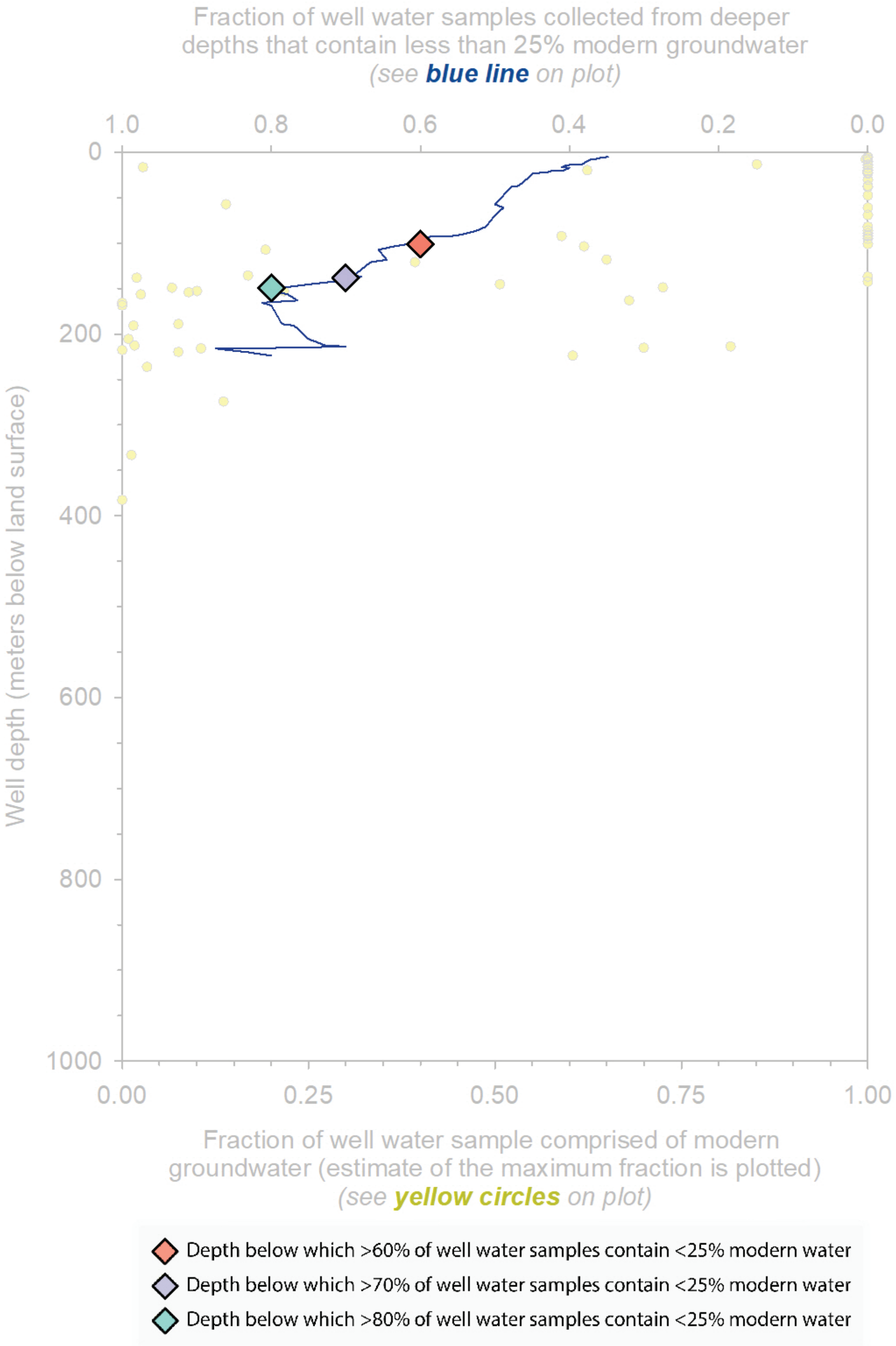

Supplementary Fig. 72. Long Island modern well water prevalence with depth. For details on symbology see the paragraph at the beginning of Supplementary Note 1.

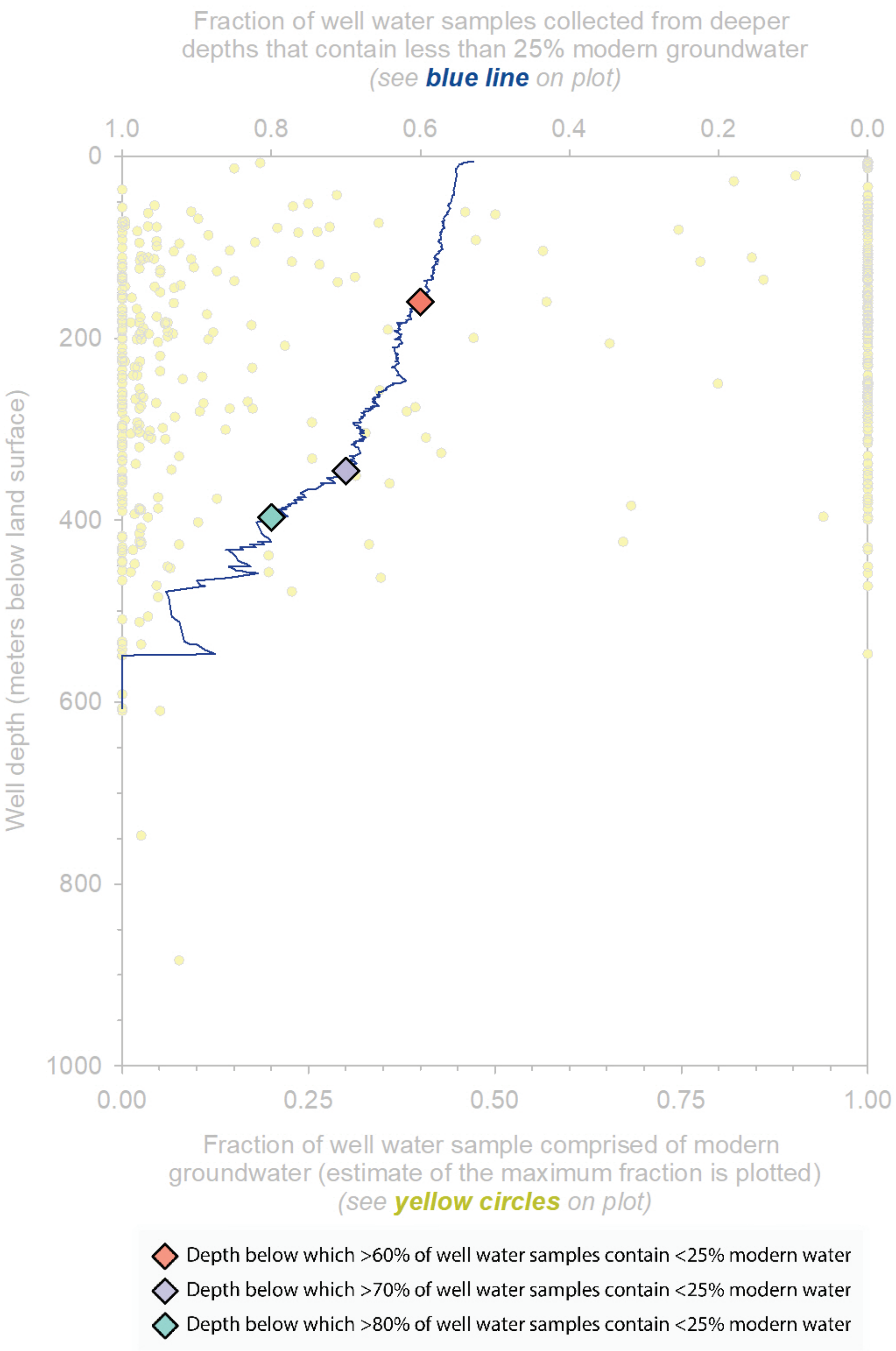

Supplementary Fig. 73. Los Angeles Basin modern well water prevalence with depth. For details on symbology see the paragraph at the beginning of Supplementary Note 1.

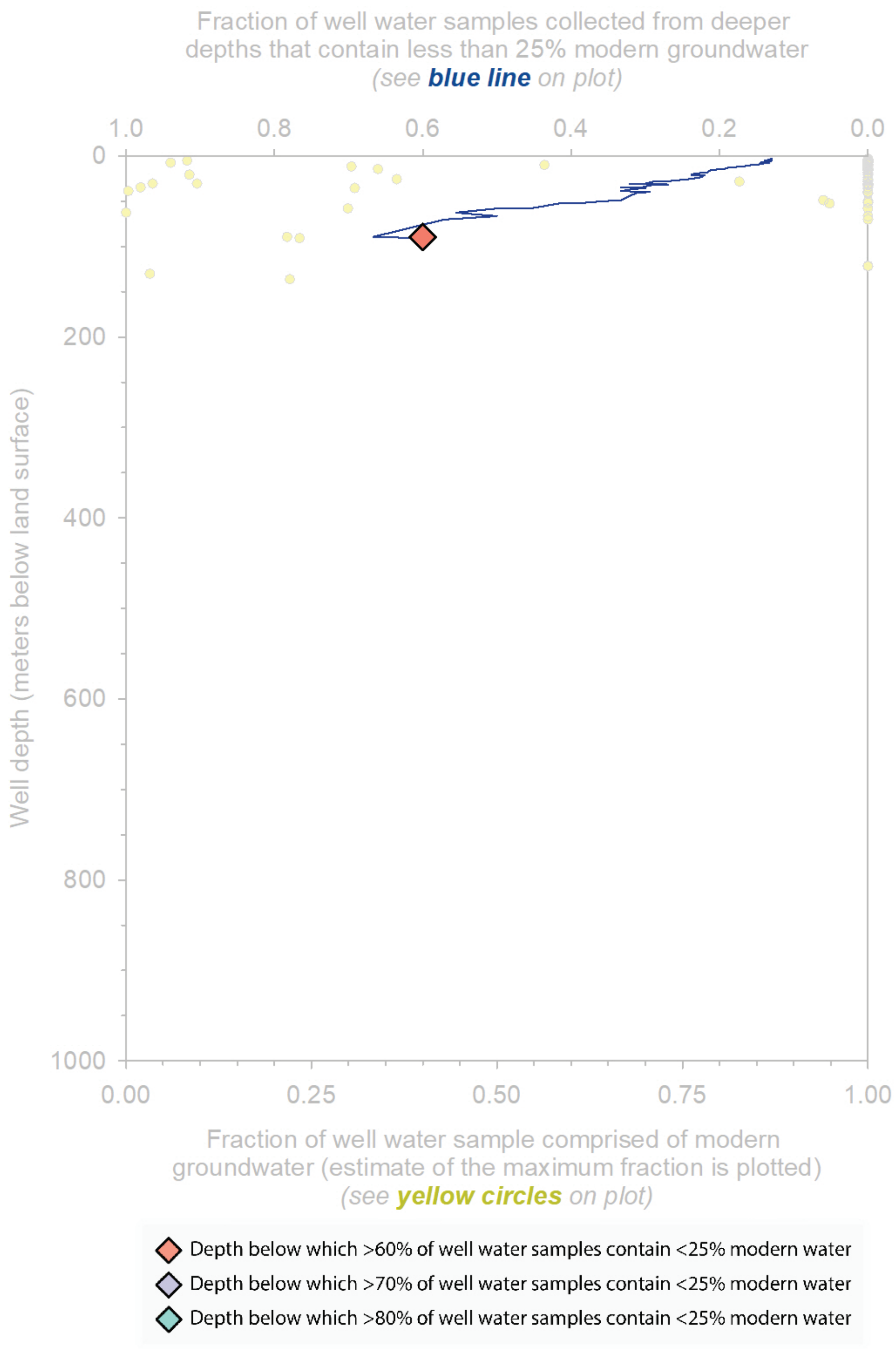

Supplementary Fig. 74. Michigan Basin modern well water prevalence with depth. For details on symbology see the paragraph at the beginning of Supplementary Note 1.

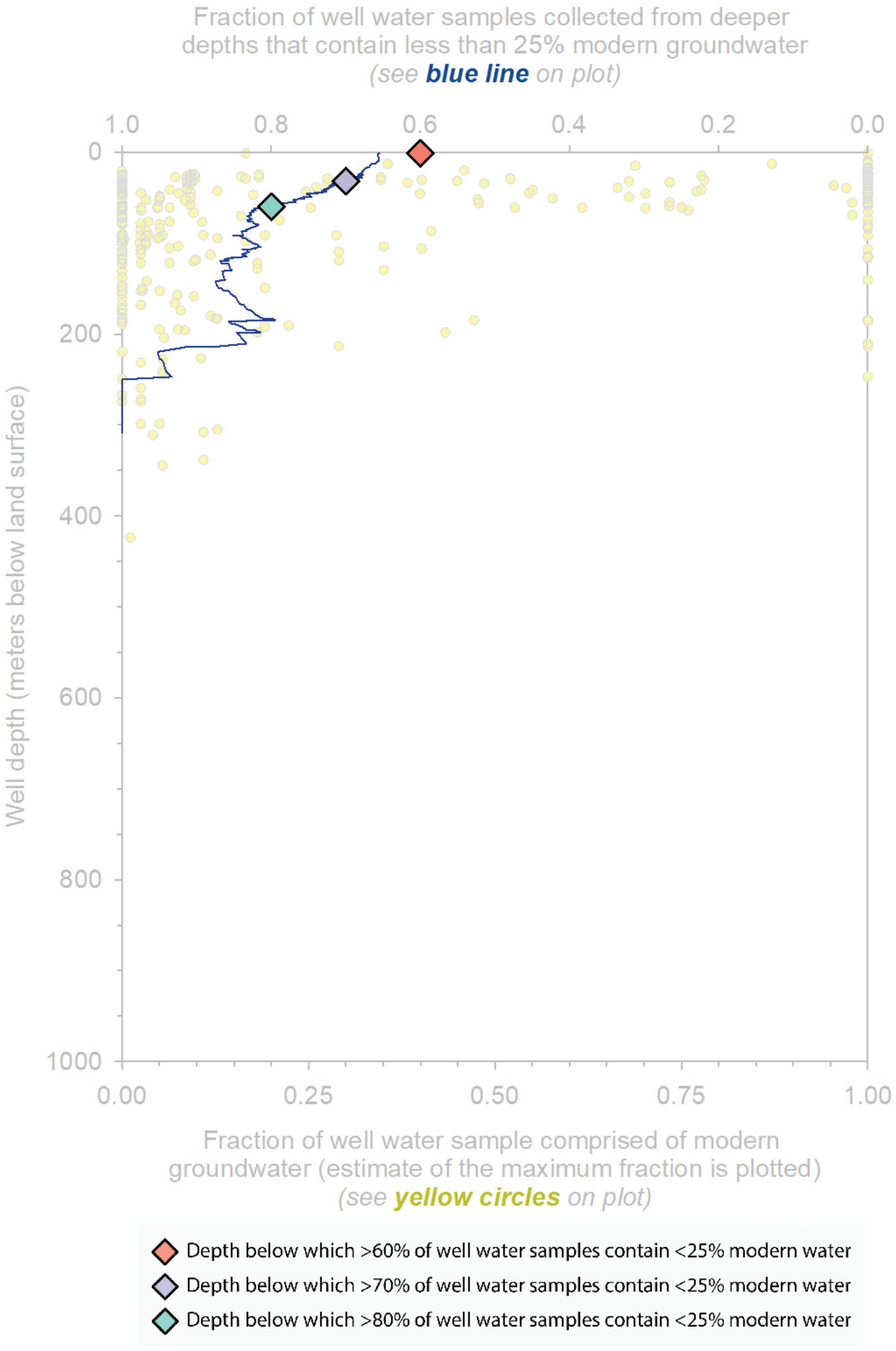

Supplementary Fig. 75. Mojave Basin modern well water prevalence with depth. For details on symbology see the paragraph at the beginning of Supplementary Note 1.

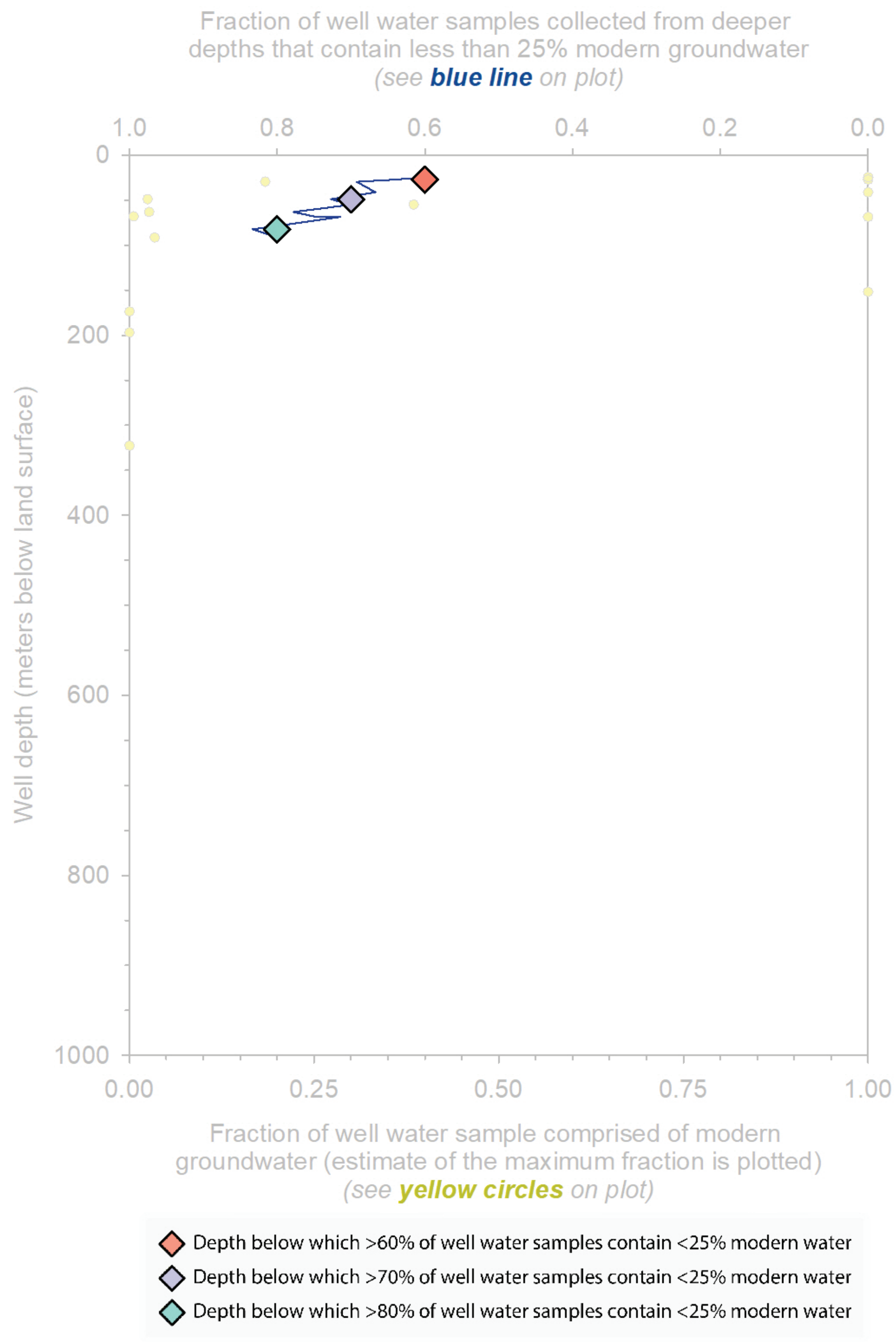

Supplementary Fig. 76. Northern Green River Basin modern well water prevalence with depth. For details on symbology see the paragraph at the beginning of Supplementary Note 1.

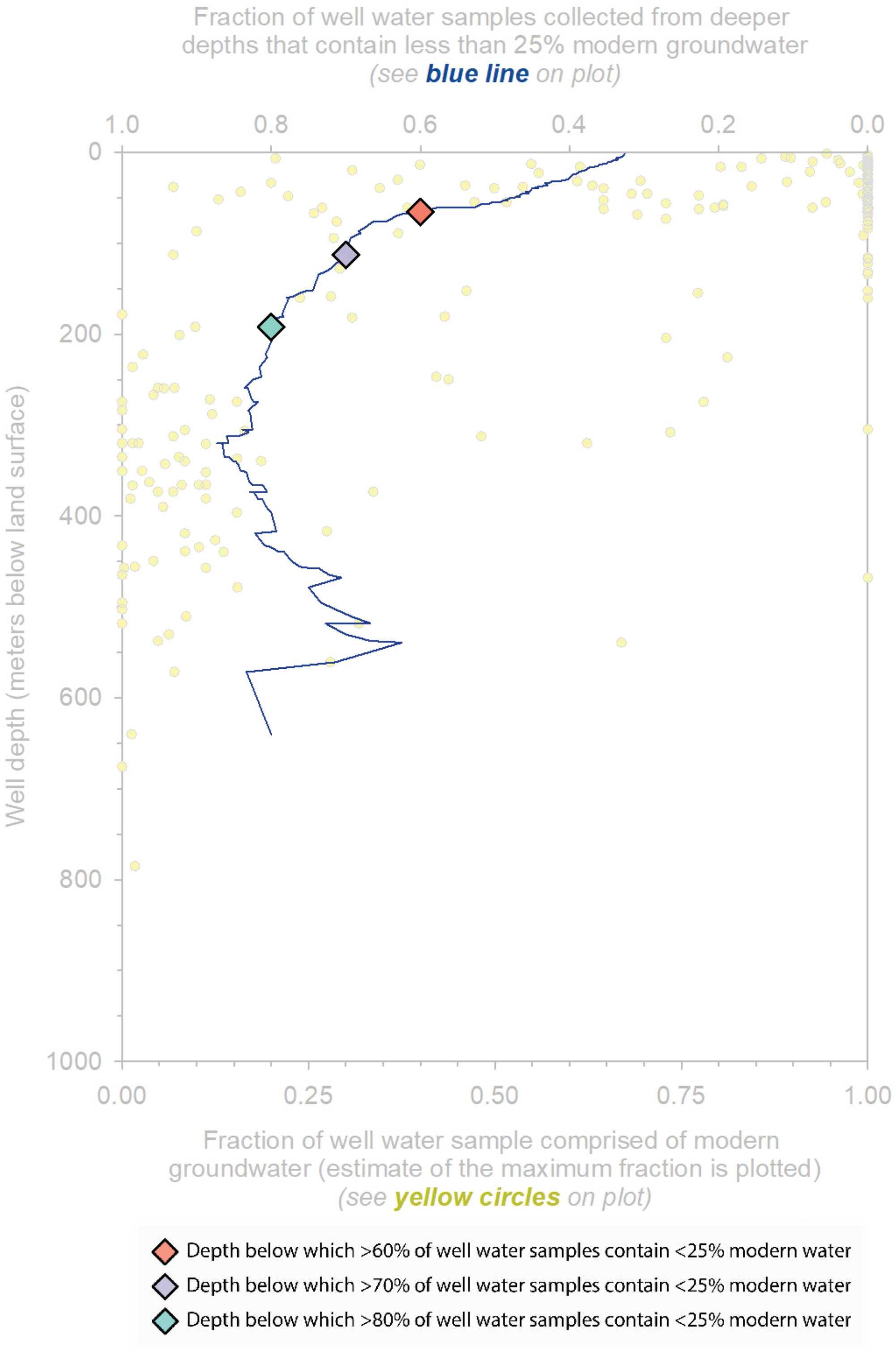

Supplementary Fig. 77. Ozark Plateaus Aquifer System modern well water prevalence with depth. For details on symbology see the paragraph at the beginning of Supplementary Note 1.

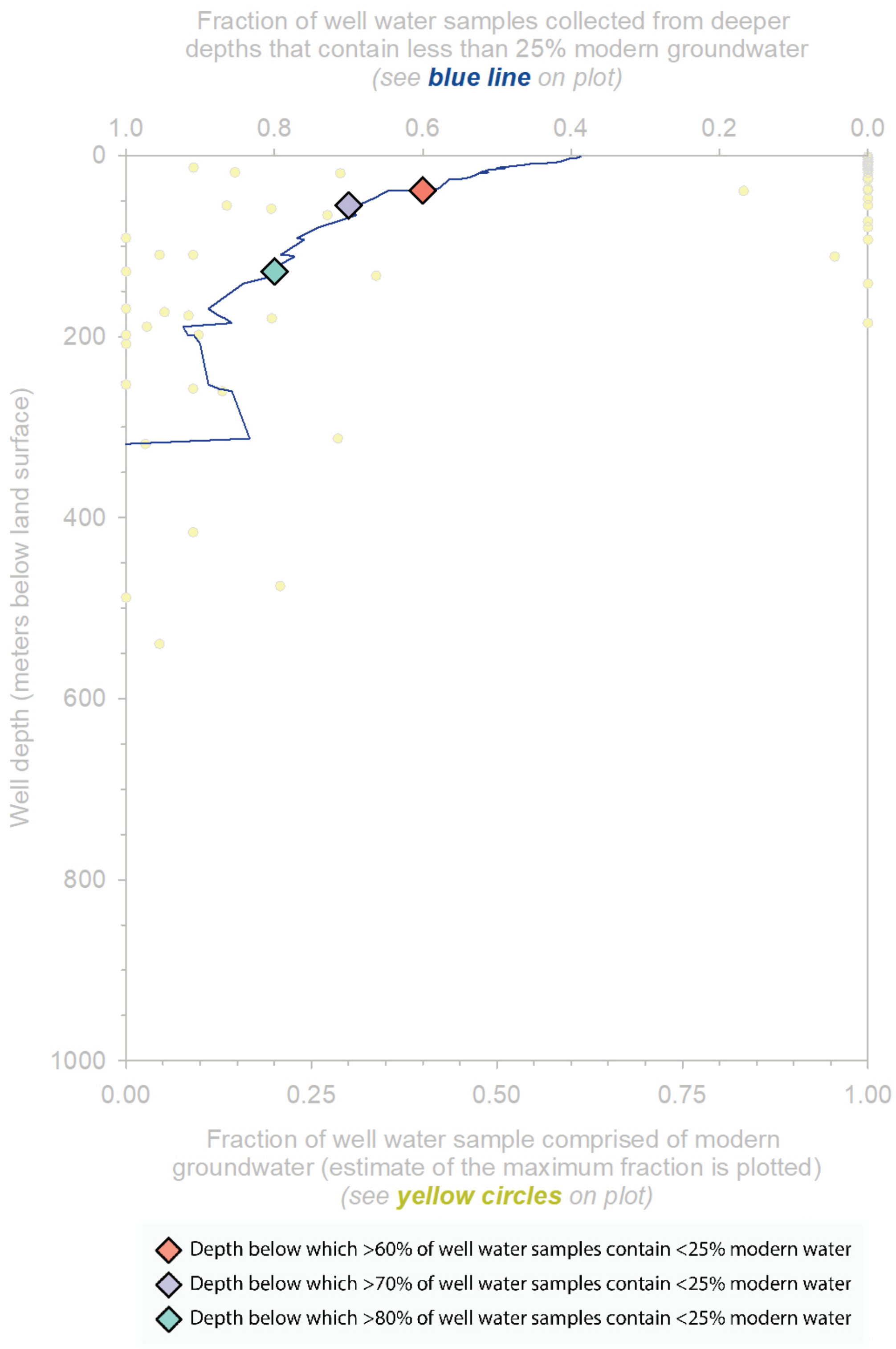

Supplementary Fig. 78. Pearl and Chattahoochee Aquifer System modern well water prevalence with depth. For details on symbology see the paragraph at the beginning of Supplementary Note 1.

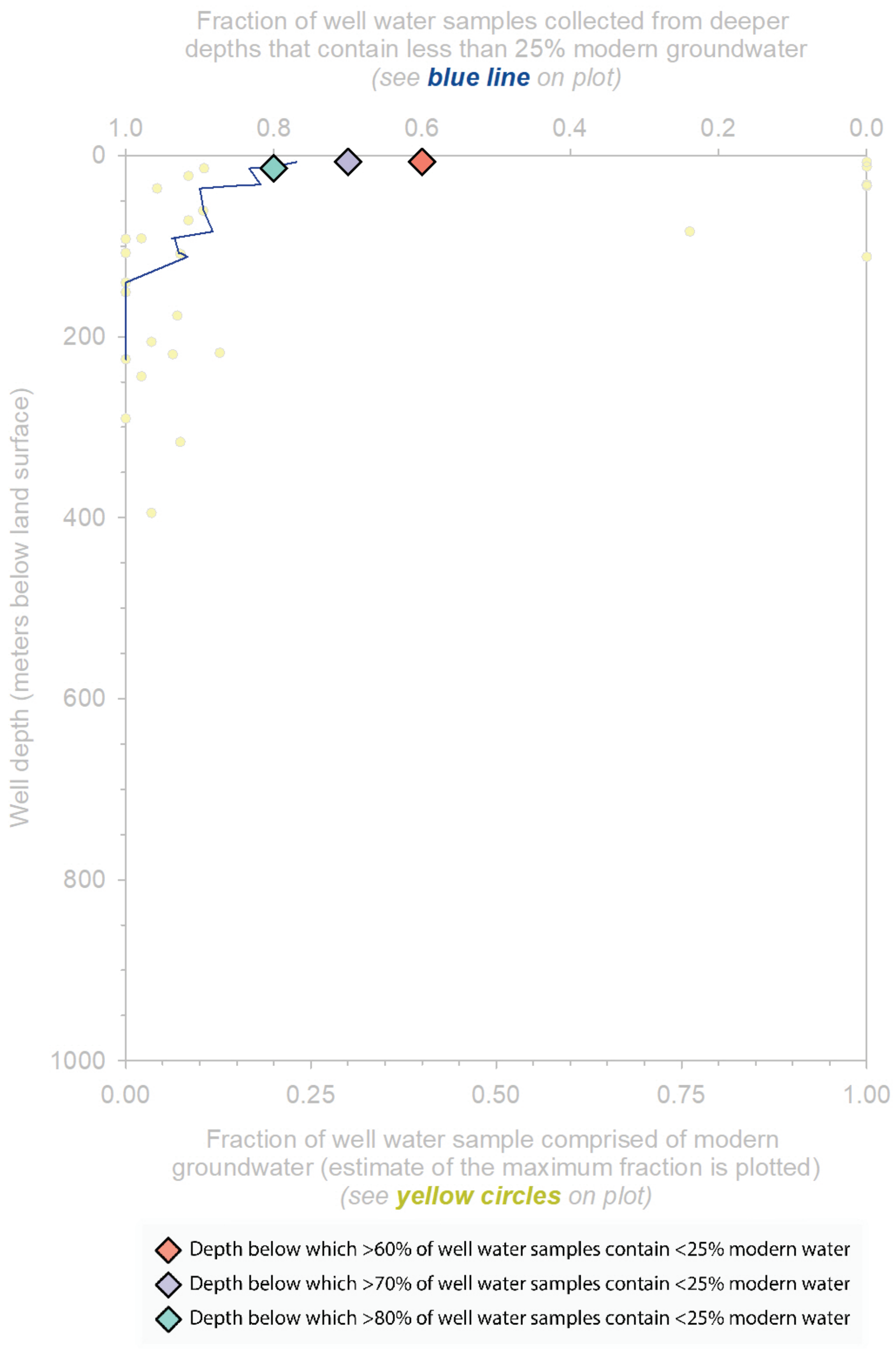

Supplementary Fig. 79. Peedee and Black Creek and Cape Fear Aquifers modern well water prevalence with depth. For details on symbology see the paragraph at the beginning of Supplementary Note 1.

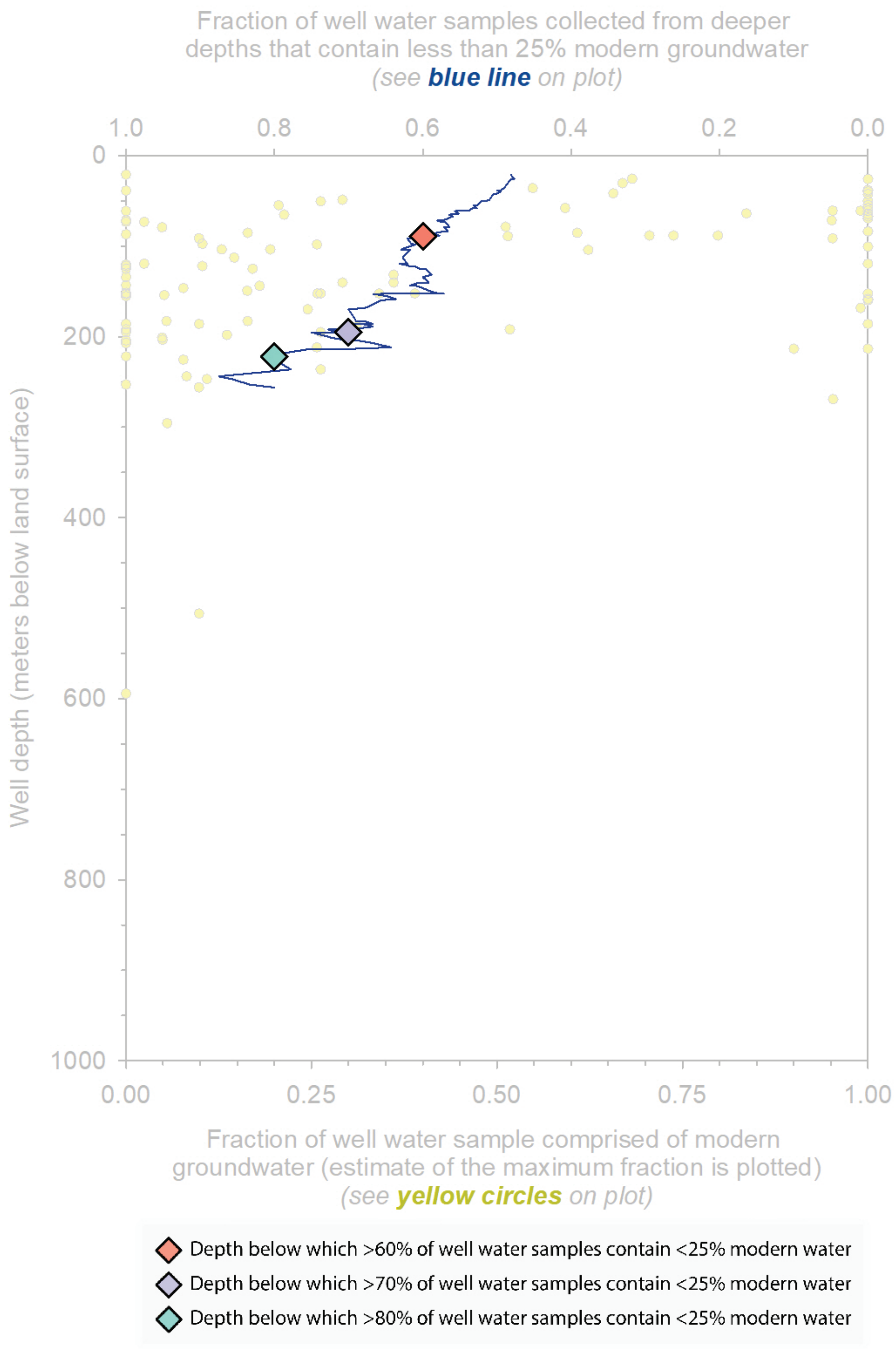

Supplementary Fig. 80. Salinas Valley modern well water prevalence with depth. For details on symbology see the paragraph at the beginning of Supplementary Note 1.

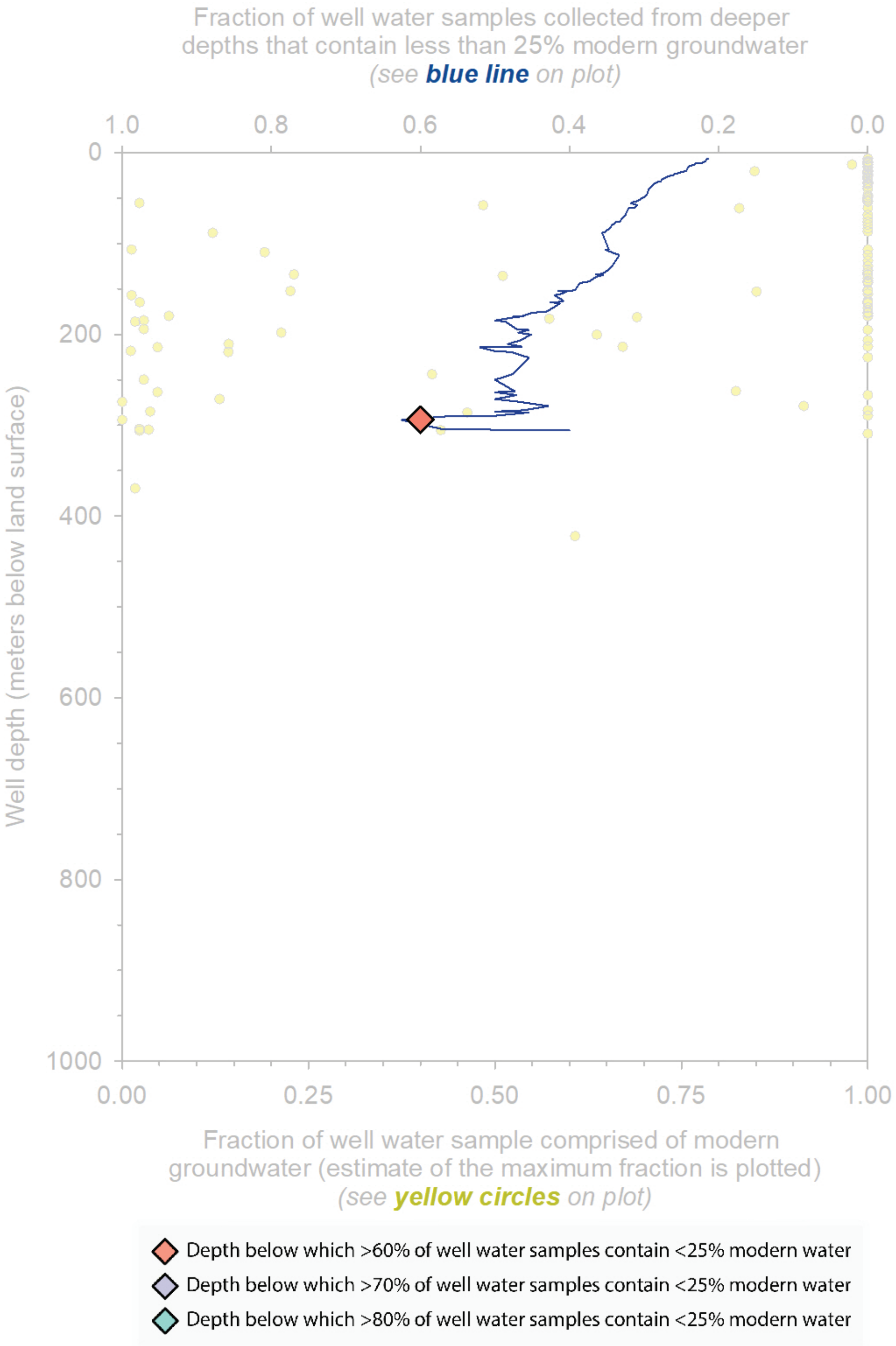

Supplementary Fig. 81. Salt Lake Valley modern well water prevalence with depth. For details on symbology see the paragraph at the beginning of Supplementary Note 1.

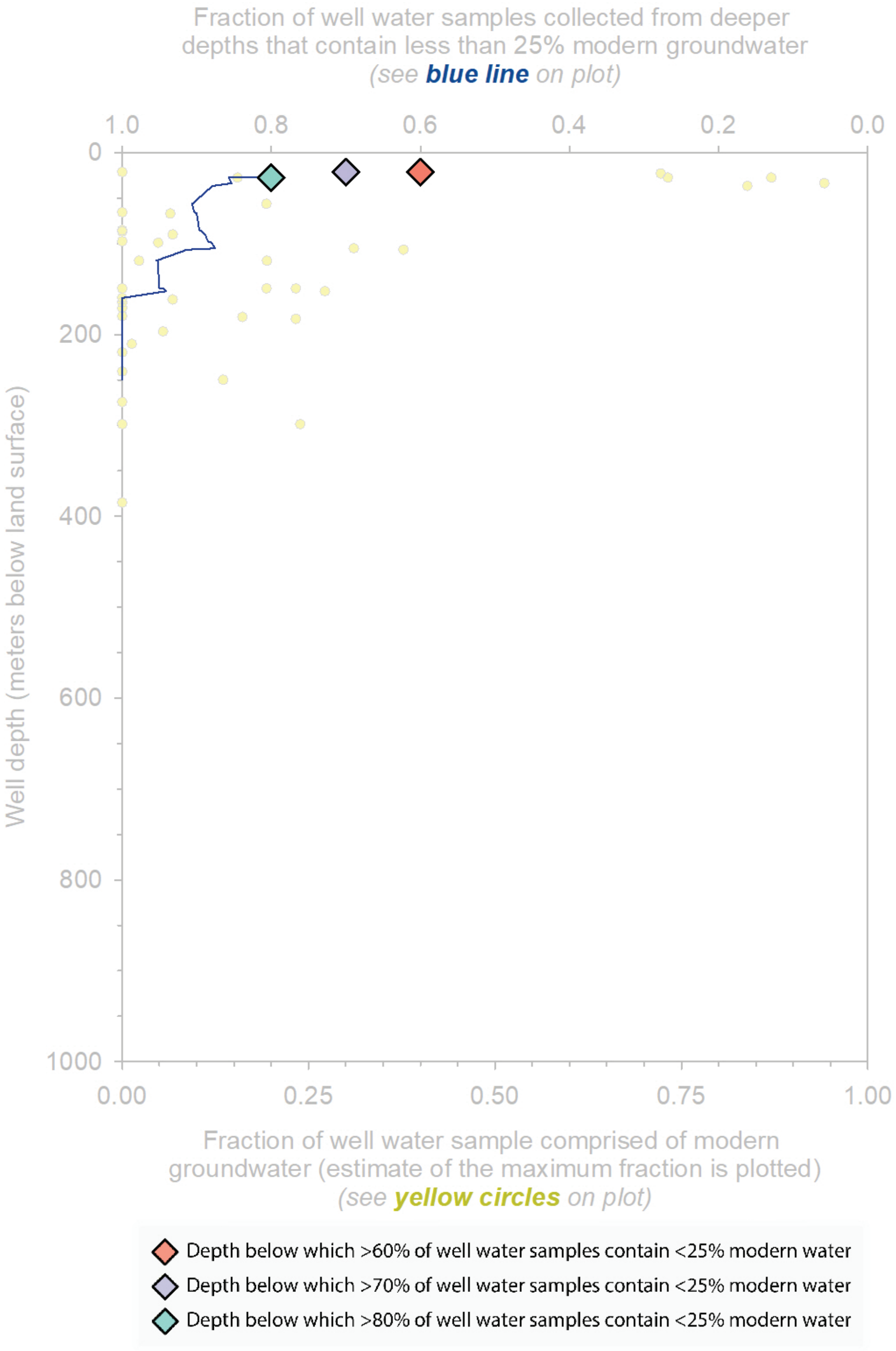

Supplementary Fig. 82. San Antonio Creek Valley modern well water prevalence with depth. For details on symbology see the paragraph at the beginning of Supplementary Note 1.

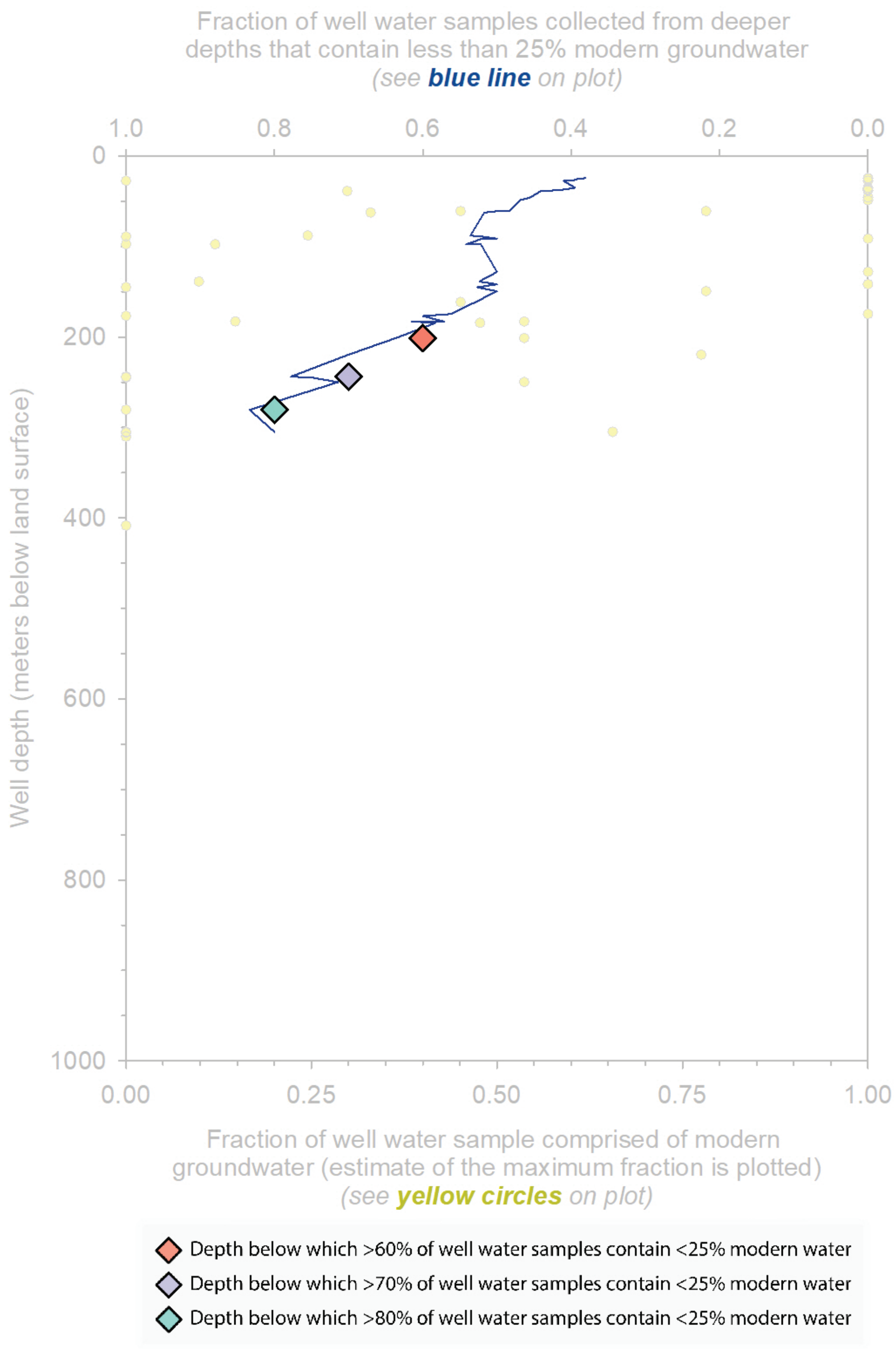

Supplementary Fig. 83. San Pedro Basin modern well water prevalence with depth. For details on symbology see the paragraph at the beginning of Supplementary Note 1.

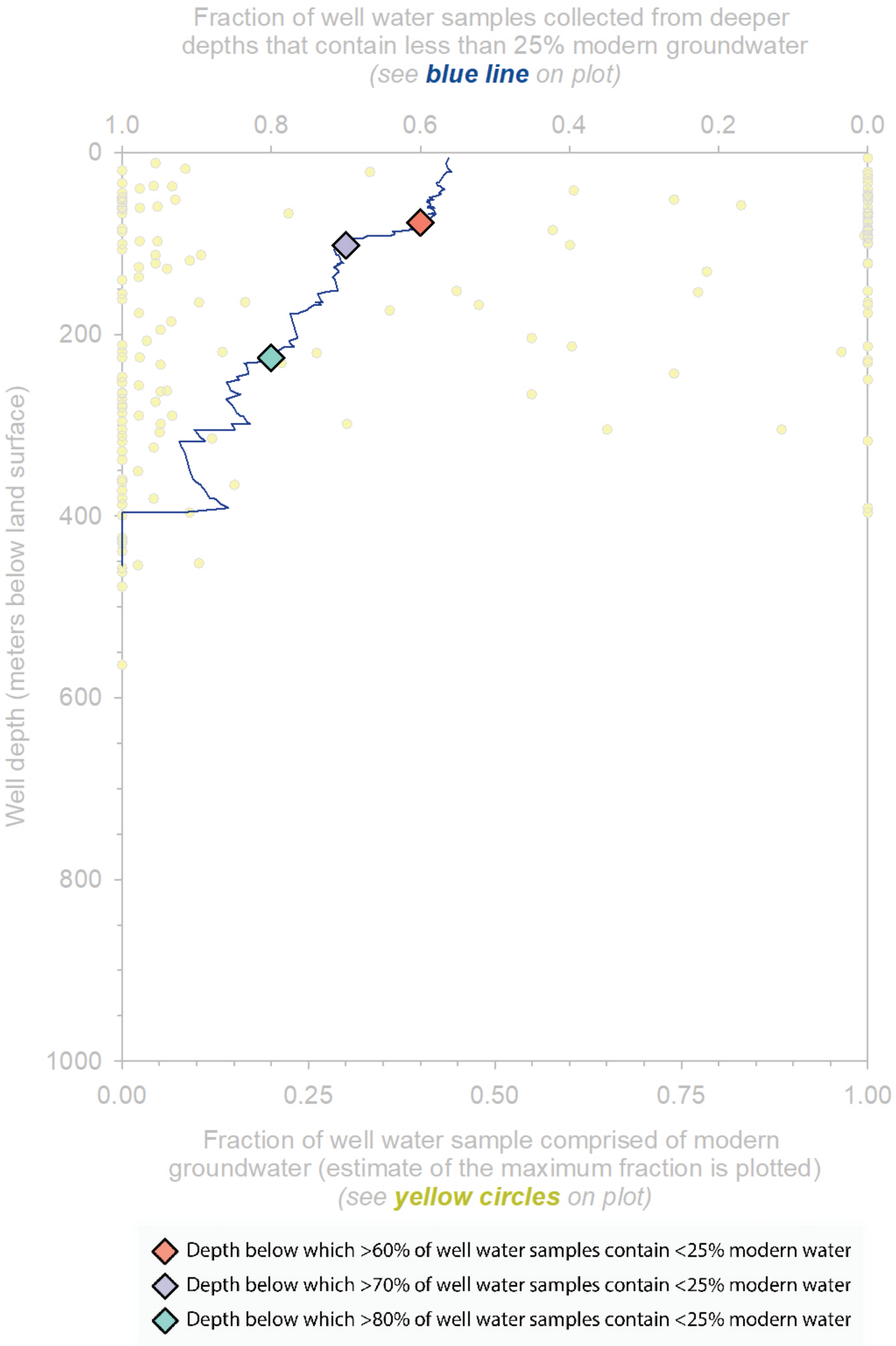

Supplementary Fig. 84. Santa Clara-Calleguas Basin modern well water prevalence with depth. For details on symbology see the paragraph at the beginning of Supplementary Note 1.

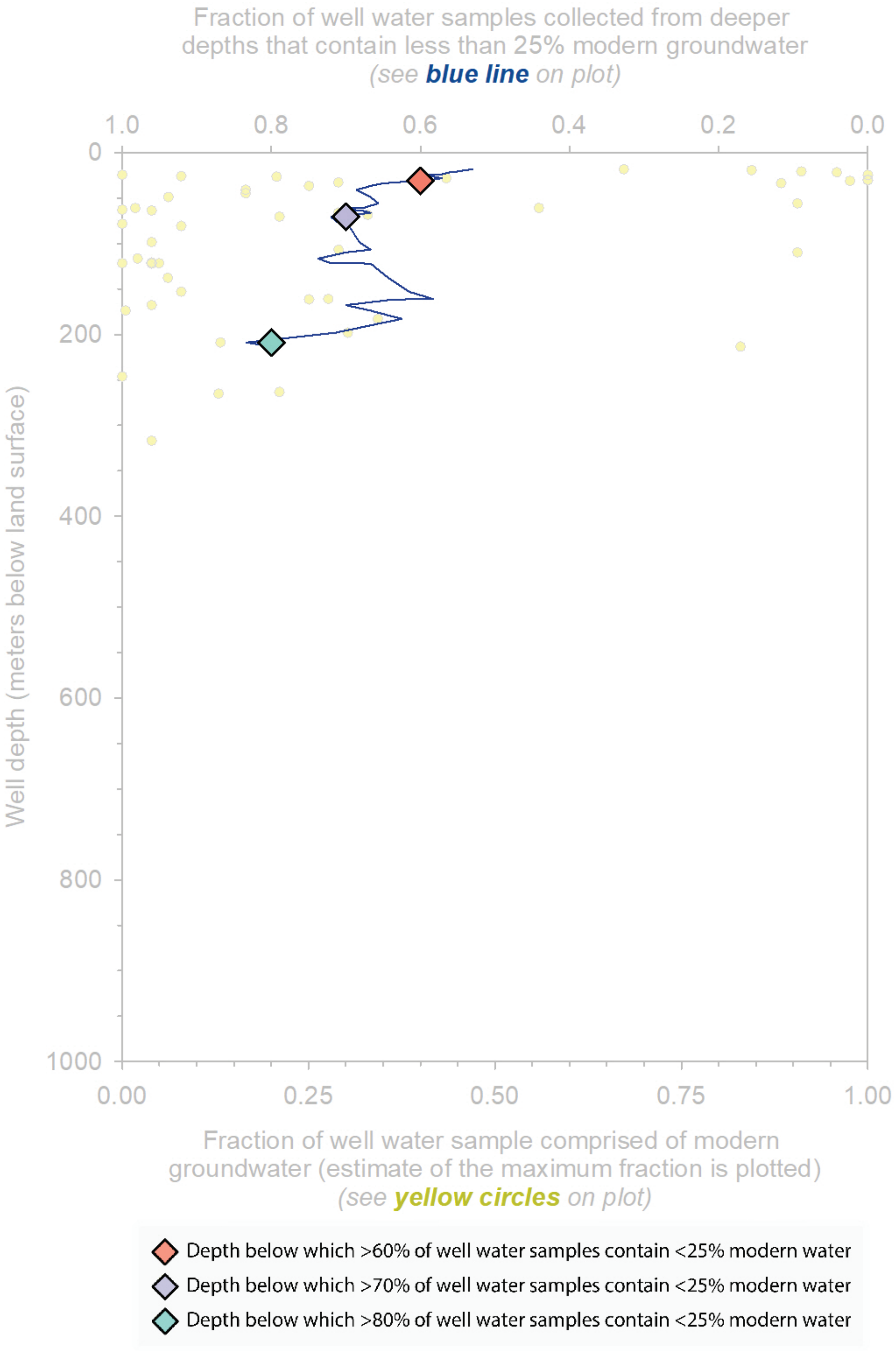

Supplementary Fig. 85. Santa Rosa Valley modern well water prevalence with depth. For details on symbology see the paragraph at the beginning of Supplementary Note 1.

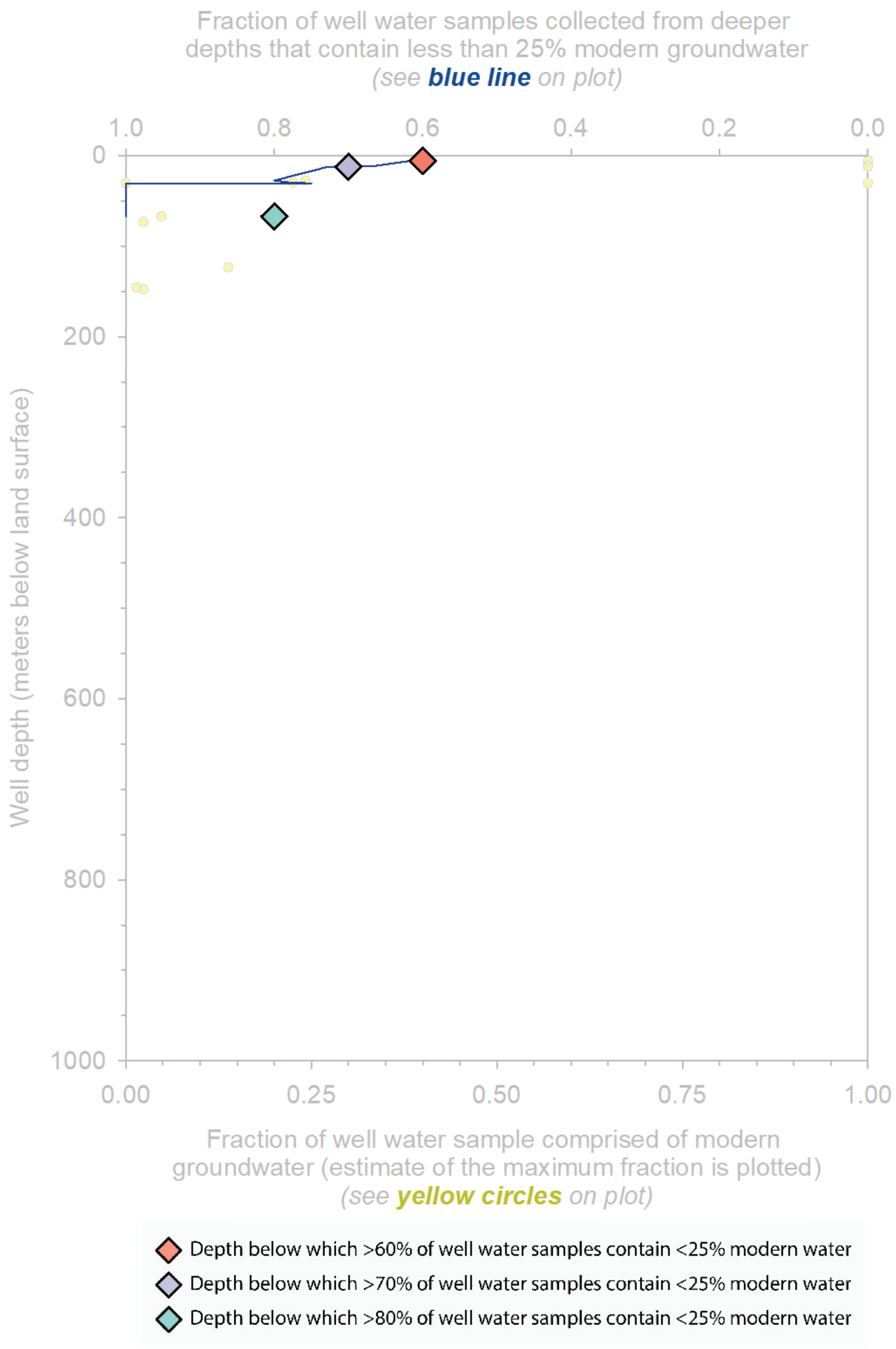

Supplementary Fig. 86. South Park Basin modern well water prevalence with depth. For details on symbology see the paragraph at the beginning of Supplementary Note 1.

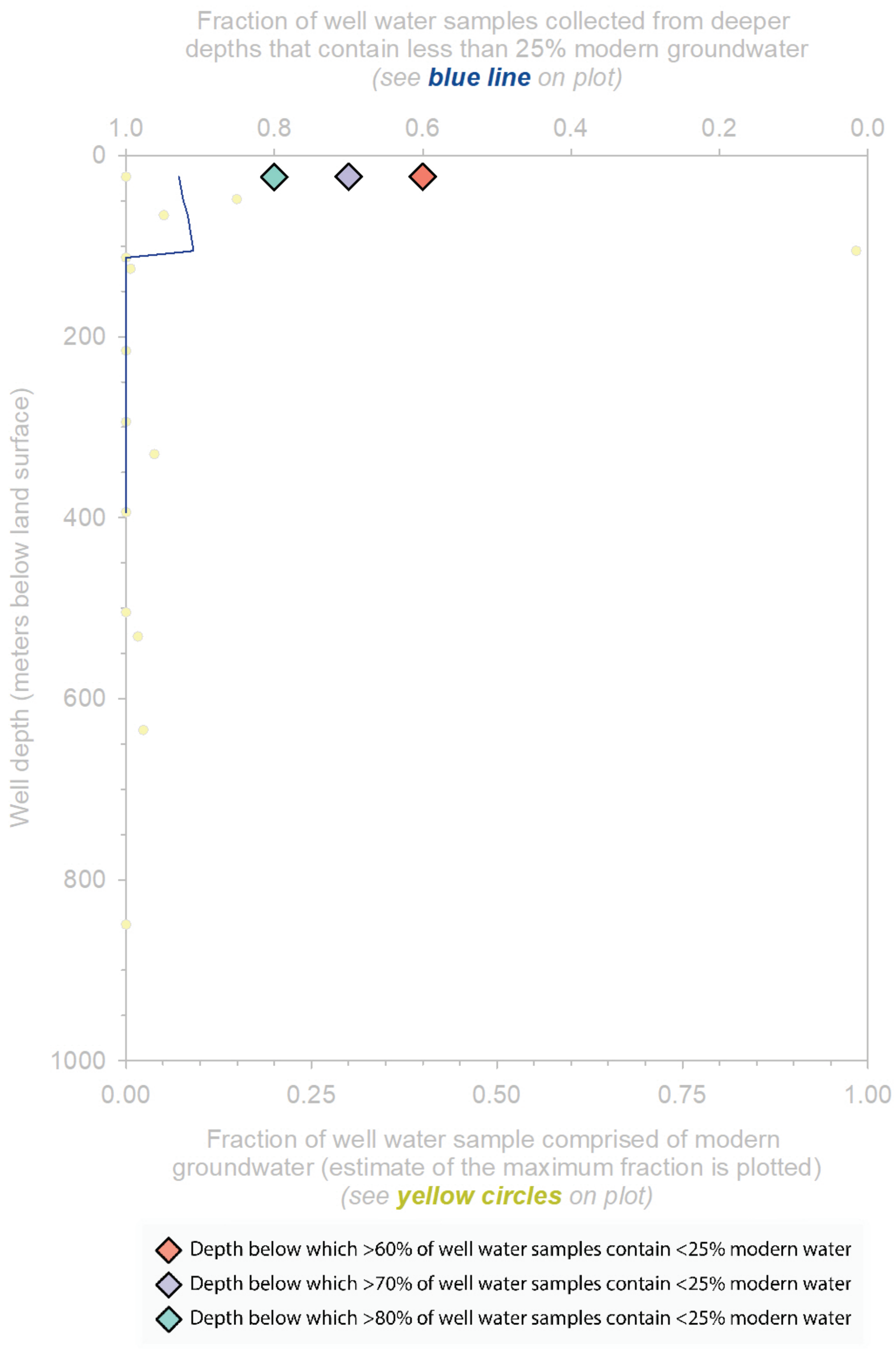

Supplementary Fig. 87. Southern San Juan Basin modern well water prevalence with depth. For details on symbology see the paragraph at the beginning of Supplementary Note 1.

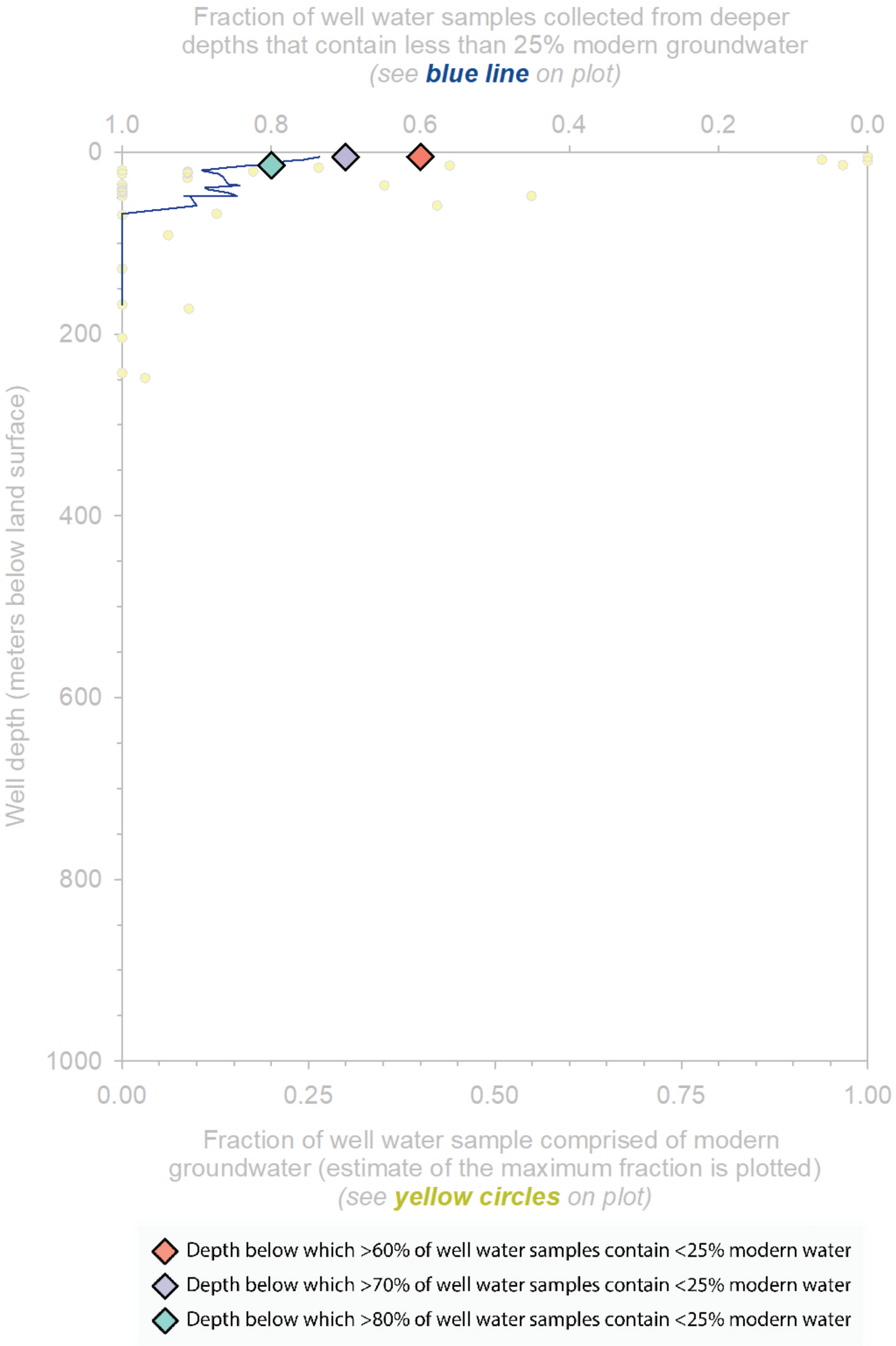

Supplementary Fig. 88. Spanish Springs Valley modern well water prevalence with depth. For details on symbology see the paragraph at the beginning of Supplementary Note 1.

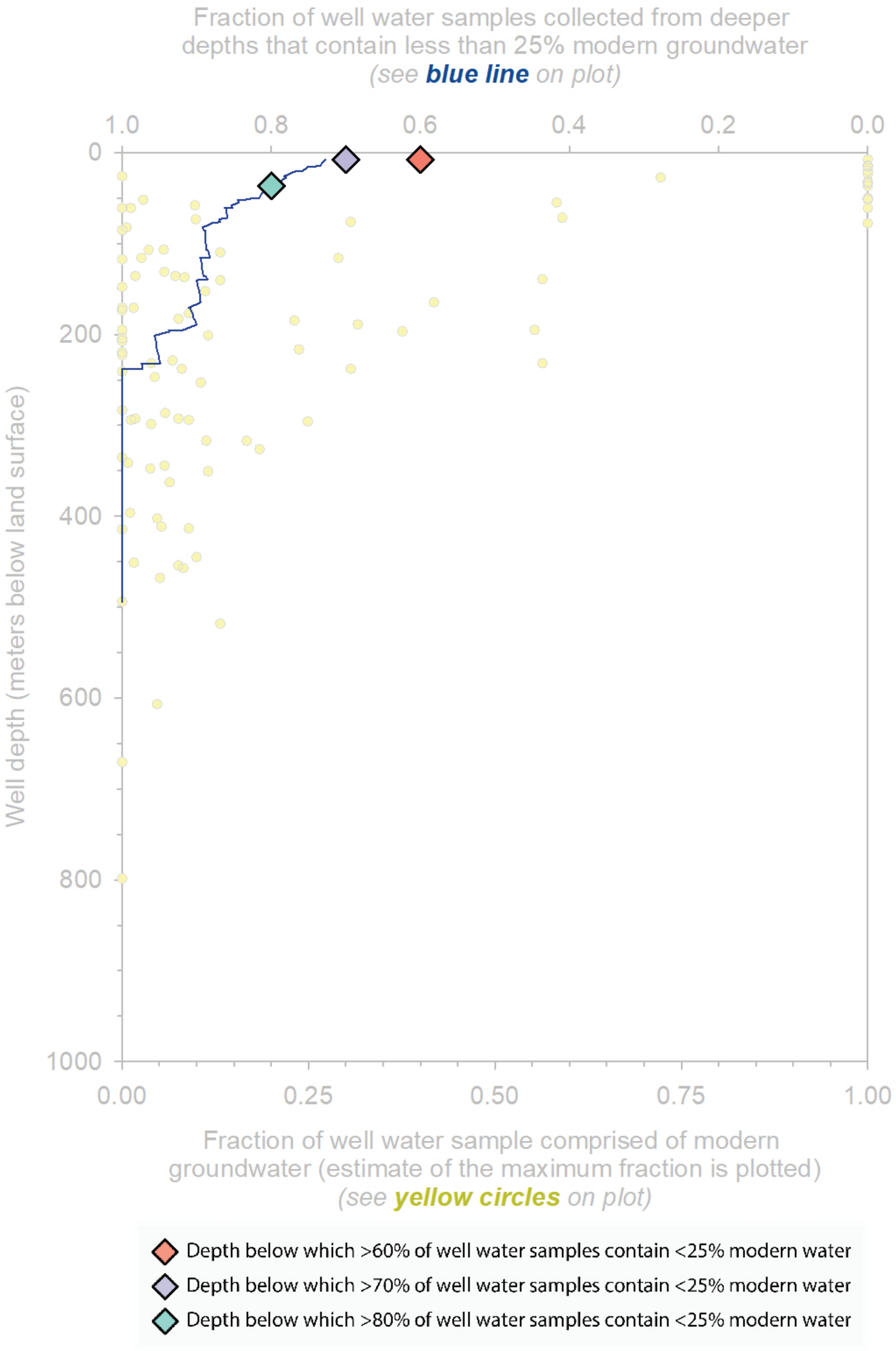

Supplementary Fig. 89. Tijuana-San Diego modern well water prevalence with depth. For details on symbology see the paragraph at the beginning of Supplementary Note 1.

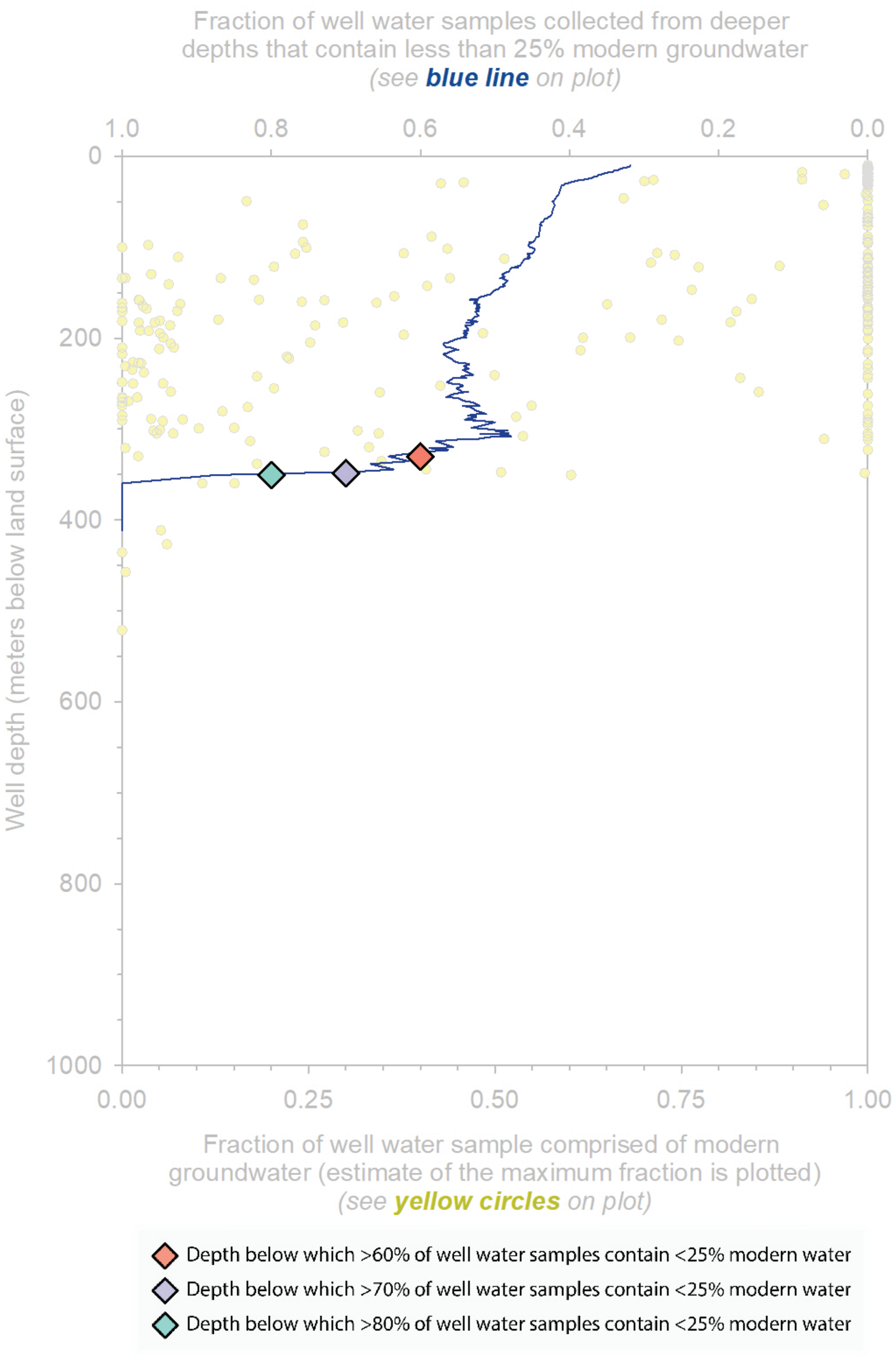

Supplementary Fig. 90. Upper Santa Ana Basin modern well water prevalence with depth. For details on symbology see the paragraph at the beginning of Supplementary Note 1.

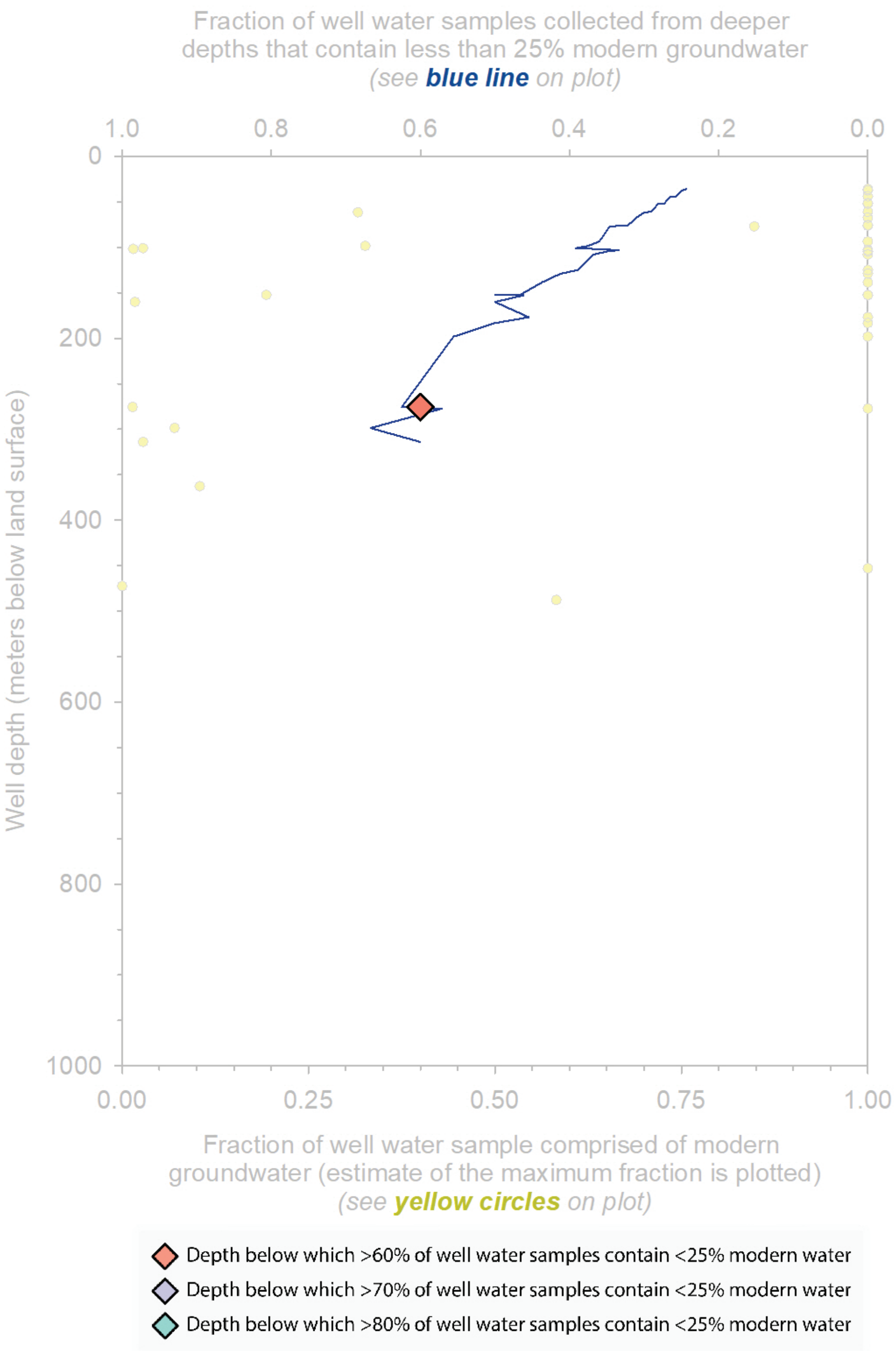

Supplementary Fig. 91. Utah Lake Valley modern well water prevalence with depth. For details on symbology see the paragraph at the beginning of Supplementary Note 1

Supplementary Note 2. Estimating groundwater withdrawals within each study area

We analysed groundwater withdrawal data in order to calculate correlations between the depth to which modern groundwater penetrates and groundwater withdrawals.

We examined the spatial distribution of groundwater withdrawals reported by the USGS for 2015 (see Dieter, C.A., Maupin, M.A., Caldwell, R.R., Harris, M.A., Ivahnenko, T.I., Lovelace, J.K., Barber, N.L., and Linsey, K.S., 2018, Estimated use of water in the United States in 2015: U.S. Geological Survey Circular 1441, 65 p., data from: https://www.sciencebase.gov/catalog/item/get/5af3311be4b0da30c1b245d8; field title: "TO-WGWTo").

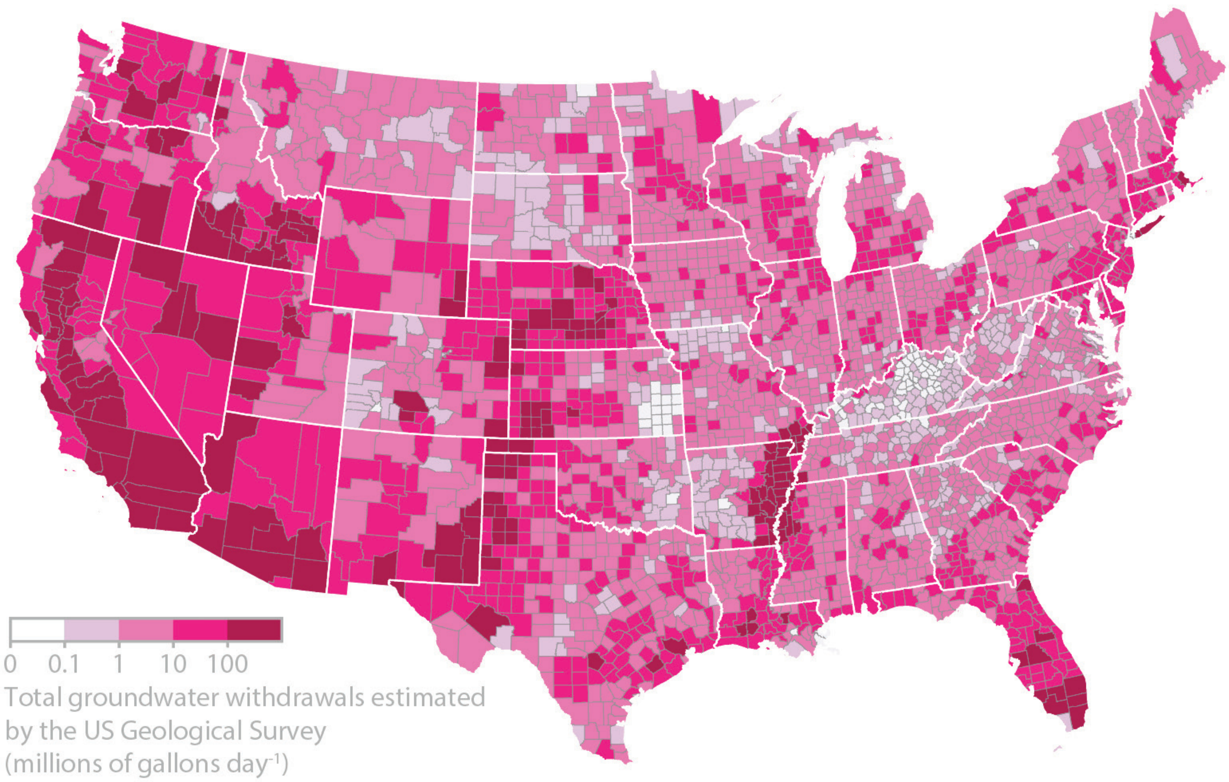

Supplementary Fig. 92. Groundwater withdrawals in countries estimated by Dieter, C.A., Maupin, M.A., Caldwell, R.R., Harris, M.A., Ivahnenko, T.I., Lovelace, J.K., Barber, N.L., and Linsey, K.S., Estimated use of water in the United States in 2015. US Geological Survey Circular 1441, 65 pp. (2018). Accessed May 23, 2022 via https://pubs.er.usgs.gov/publication/cir1441. The spatial distribution of county-scale groundwater withdrawals expressed in millions of gallons per day highlights the wide variation in groundwater withdrawals across the US. However, because some counties are larger than others (e.g., see large counties in southern California), comparing the groundwater withdrawals across counties is challenging.

The raw groundwater withdrawal data (figure above) are reported as a volumetric flux (in units of millions of gallons per day); these units are imperfect for comparison across counties, because some have larger areas than others. For example, the counties in southern California are substantially larger than those in Kentucky, meaning the volumetric fluxes (i.e., groundwater withdrawals in figure on previous page) cannot be straightforwardly compared in their current units. Further, groundwater use within counties is unevenly distributed; for example, some of the expansive counties in the southern portion of California's Central Valley extend from the Sierra Nevada mountains (where groundwater pumping is limited) across the Tulare Basin of the Central Valley (where groundwater extractions can be high). These limitations with county-scale data led us to apply a downscaling method in order to estimate groundwater withdrawals at a finer spatial resolution.

We downscaled these data using two finer-resolution data products: (i) gridded population density data from https://sedac.ciesin.columbia.edu/data/set/gpw-v4-population-density-rev11/data-download (1 km^2 data population density data for the year 2015; the year 2015 was selected to match the water use data, which are also for the year 2015), and (ii) irrigated land areas from https://www.sciencebase.gov/catalog/item/5db08e84e4b0b0c58b56e04f (1 km^2 irrigation data for the year 2017). Specifically, to downscale the spatial distribution of groundwater withdrawals across the US we completed two steps.

(i) First, we downscaled county scale irrigation groundwater withdrawals using MODIS irrigated agriculture grids (total county-level irrigation groundwater withdrawals from Dieter et al. (2018), see field entitled: "IR-WGWFr"). Specifically, we calculated the total number of 1 km by 1 km grids within each county; next total county-level groundwater irrigation withdrawals were distributed spatially according to the relative intensity of irrigation as estimated in the MODIS-based dataset. In this way, the estimated USGS county-scale groundwater withdrawals were assumed to be spatially distributed according to the MODIS-based irrigation data. This approach introduces uncertainty in a number of ways, one of which being that our irrigated land data does not provide information pertaining to the water source for irrigation (i.e., groundwater-fed versus surface-water-fed irrigation). However, we found that this downscaling is preferable to the alternative of analysing groundwater use at county scales, as counties are often too expansive to be suitable for locally relevant analyses. For example, our downscaling approach distributes Kern County irrigation withdrawals to the Central Valley (where groundwater-sourced irrigation water withdrawals are known to be high) and correctly identifies that minimal (i.e., at- or near-zero) irrigation occurs in the higher elevations of the Sierra Nevada.

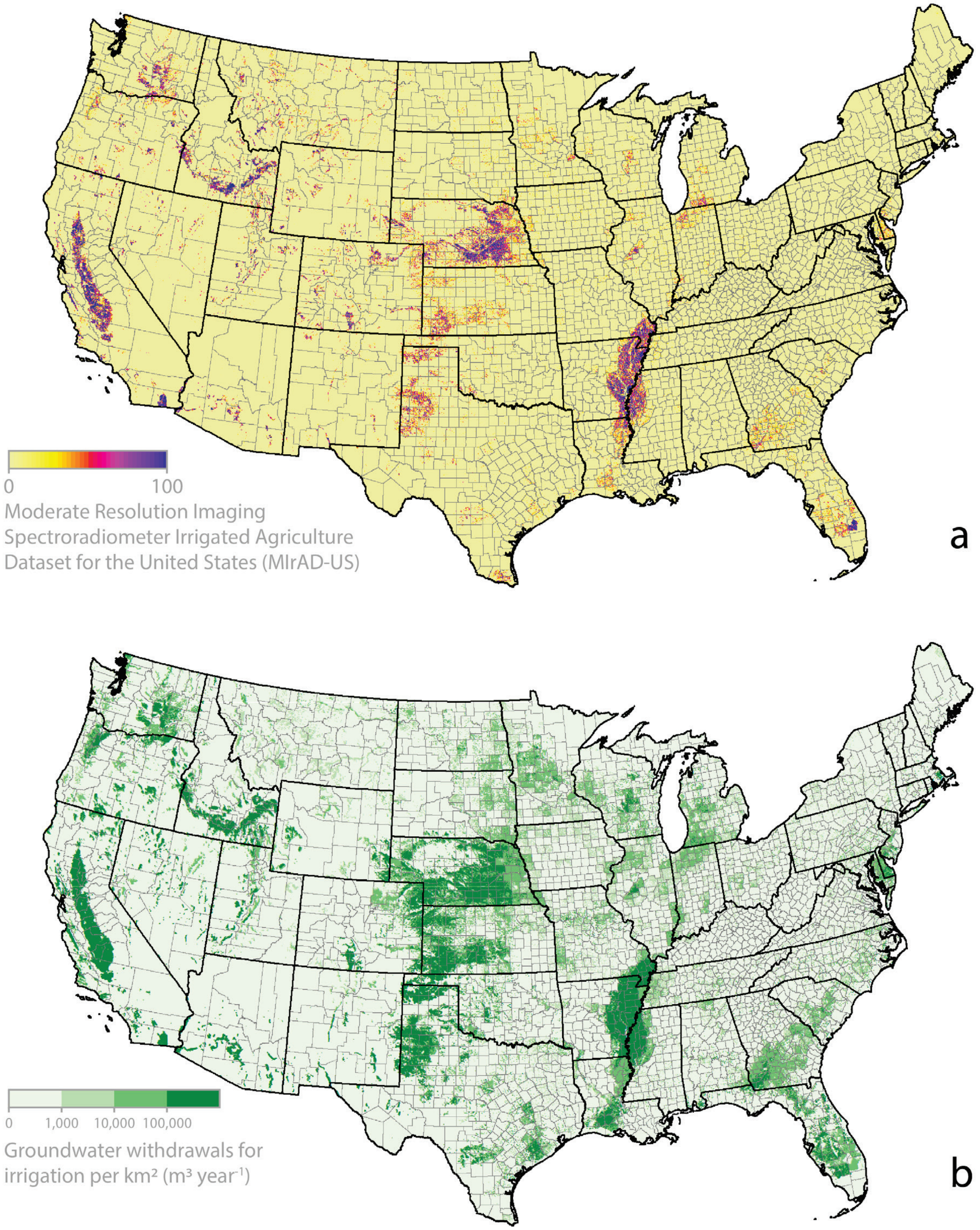

Supplementary Fig. 93. Estimating groundwater withdrawals for irrigated agriculture across the contiguous United States. a) 1 km^2 resolution MODIS-based (i.e., satellite- and land-use-data-based) irrigated lands from https://www.sciencebase.gov/catalog/item/5db08e84e4b0b0c58b56e04f. Irrigation data are for the year 2017 (the closest year to 2015, which is the year for which the groundwater withdrawal data are available for). b) Downscaled irrigation groundwater withdrawals for the year 2015 estimated from county-scale USGS irrigation groundwater withdrawals (from https://www.sciencebase.gov/catalog/item/get/5af3311be4b0da30c1b245d8; field title: “IR-WGWFr”).

(ii) Second, we downscaled all non-irrigation groundwater withdrawals using 1 km^2 population density data from https://sedac.ciesin.columbia.edu/data/set/gpw-v4-population-density-rev11/data-download (1 km^2 data population density data for the year 2015; the year 2015 was selected to match the water use data, which are also for the year 2015). County-scale non-irrigation groundwater withdrawals were calculated by subtracting irrigation groundwater withdrawals (field title: "IR-WGWFr" in https://www.sciencebase.gov/catalog/item/get/5af3311be4b0da30c1b245d8) from total groundwater withdrawals (field title: "TO-WGWTo" https://www.sciencebase.gov/catalog/item/get/5af3311be4b0da30c1b245d8). County-scale non-irrigation groundwater withdrawals were assumed to be distributed across each county according to the relative distribution of population within each county. An assumption of a scaling relationship between population density and groundwater pumping has been applied in previous studies of the portions of the contiguous United States (e.g., Ferguson, G., Gleeson, T. Vulnerability of coastal aquifers to groundwater use and climate change. *Nature Climate Change* **2,** 342-345 (2012)). Like our approach for analysing the spatial patterns of groundwater-fed irrigation (i.e., (i) above), this approach introduces uncertainty into our analyses, as spatial patterns of population density are not necessarily related to spatial patterns of non-irrigation groundwater withdrawals. The estimated spatial distribution of non-irrigation groundwater withdrawals across the United States are displayed in the figure to follow:

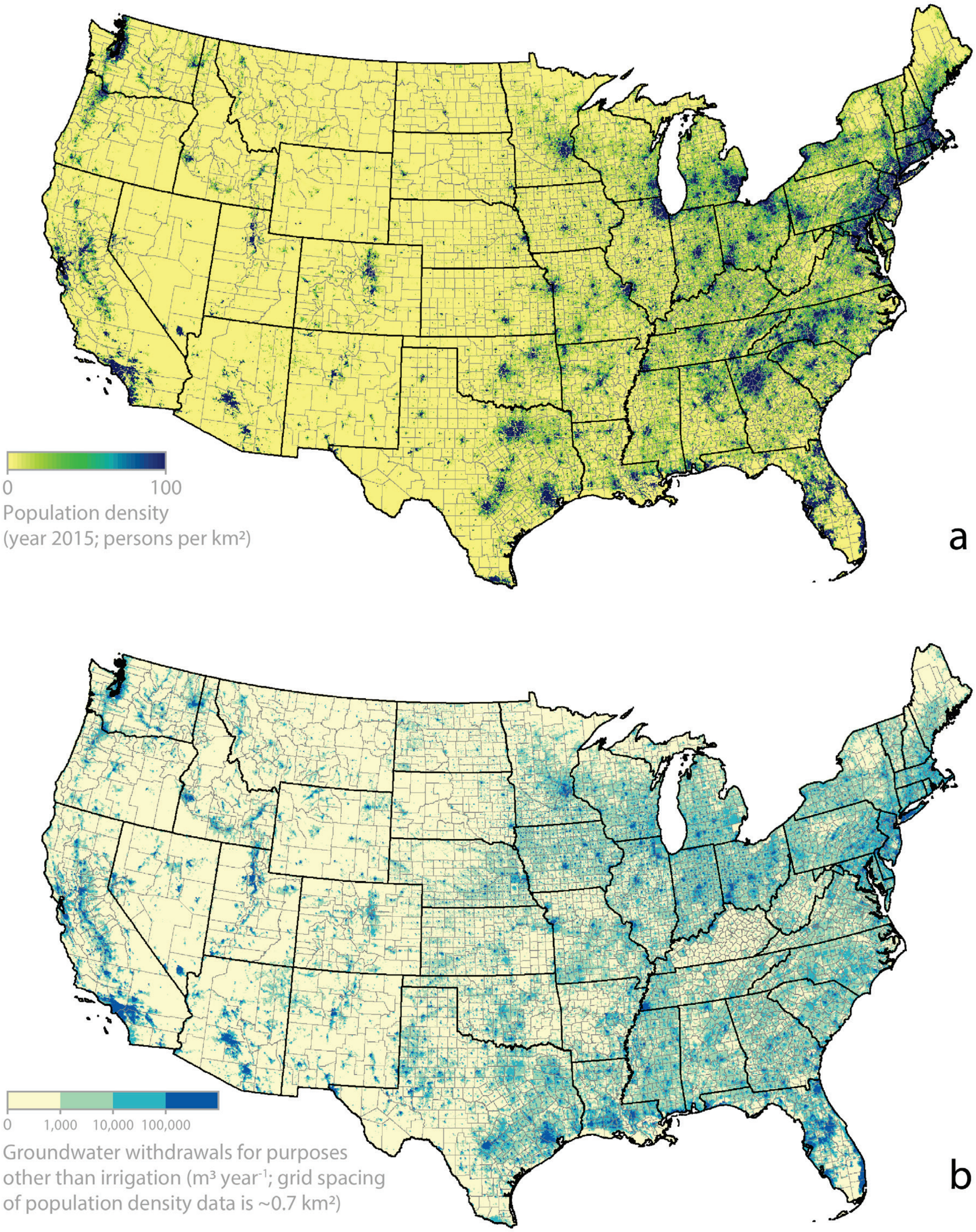

Supplementary Fig. 94. Estimating groundwater withdrawals for purposes other than irrigated agriculture across the contiguous United States. a) Population density from https://sedac.ciesin.columbia.edu/data/set/gpw-v4-population-density-rev11/data-download. b) Downscaled non-irrigation groundwater withdrawals for the year 2015 estimated by downscaling county-scale USGS-reported groundwater withdrawal data using population density data displayed in panel a.

Next, for each of our study aquifer systems, we quantified annual groundwater withdrawals for the year 2015 by calculating the sum of all estimated groundwater withdrawals taking place within the boundaries of the aquifer system. The estimated total annual irrigation and non-irrigation groundwater withdrawals taking place within the boundaries of each of our n=74 study areas are shown in the following table.

Supplementary Table 2. Estimated annual groundwater withdrawals in each study aquifer system

Aquifer system	Annual groundwater withdrawals estimated for the year 2015 (cubic kilometers per year)	Area within aquifer system boundaries (km^2)	Annual groundwater withdrawals estimated for the year 2015 (mm per year)*
Albuquerque Basin	0.142	7057	20.12
Antelope Valley	0.1122	8139	13.78
Big Bear Valley	0.0045	99	45.34
Bighorn Basin	0.0941	20215	4.66
Black Hills Uplift	0.0316	18915	1.67
Black Warrior River Aquifer System	0.2685	50236	5.35
Boise Valley and Homedale Murphy Area	0.64	4569	140.06
Castle Hayne Aquifer	0.1091	9992	10.92
Catahoula Area	0.184	23067	7.98
Central High Plains	5.115	104407	48.99
Central Mississippi Embayment	16.895	89602	188.55
Central Wabash and Bloomington Ridged Plain	0.1947	25798	7.55
Coachella Valley	0.2339	1797	130.12
Cuyama Valley	0.014	483	28.97
Delmarva Peninsula	0.3698	18139	20.39
Denver Basin	0.1385	18087	7.66
Dougherty Plain and Marianna Lowlands	0.4833	18625	25.95
Eagle Valley	0.0115	179	64.27
Eastern Carrizo-Wilcox	0.3279	57255	5.73
Eastern Dakota Aquifer	0.2167	39327	5.51
Eastern Flatwoods Southshores	0.8125	25502	31.86
Eastern Mississippi Embayment	0.6278	60649	10.35
Eastern Silurian-Devonian Aquifers	0.4564	30217	15.1
Eureka and Eel River and Mad River Plains	0.0687	663	103.7
Garber-Wellington Aquifer	0.0726	8114	8.95
Honey Lake Valley	0.0312	2070	15.07
Houston-Galveston Area	0.6642	29036	22.88
Lafayette Area	1.63	65887	24.73

Aquifer system	Annual groundwater withdrawals estimated for the year 2015 (cubic kilometers per year)	Area within aquifer system boundaries (km^2)	Annual groundwater withdrawals estimated for the year 2015 (mm per year)*
Long Island	0.6361	3967	160.36
Los Angeles Basin	0.8256	2097	393.61
Lower Coastal Plain	0.1284	18570	6.91
Lower Santa Ynez Valley	0.016	400	40.03
Maryland Western Shores	0.1647	7008	23.5
Mesilla Valley	0.1843	980	187.97
Michigan Basin	0.4815	62179	7.74
Mississippian-Silurian-Devonian Carbonates	0.6808	87758	7.76
Mojave Basin	0.1017	5974	17.02
Mountain Home Plateau	0.0963	3498	27.53
New Jersey Coastal Plain	0.4955	12541	39.51
North Carolina and Virginia Coastal Plain	0.2901	49130	5.9
Northeast Missouri Carbonates	0.1229	29735	4.13
Northern Cambrian-Ordovician Aquifers	0.3132	20419	15.34
Northern Green River Basin	0.1136	11965	9.5
Northern High Plains	9.413	259022	36.34
Ocala Uplift	0.9025	35703	25.28
Ozark Plateaus Aquifer System	1.003	172532	5.81
Palouse Slope	0.1451	15220	9.53
Pearl and Chattahoochee Aquifer System	0.3231	35620	9.07
Powder River Basin	0.1087	63508	1.71
Sacramento Basin	5.488	16195	338.9
Salinas Valley	0.2515	4317	58.24
Salt Lake Valley	0.1607	1251	128.48
San Joaquin Basin	4.43	14170	312.62
San Luis Valley	0.5581	12662	44.08
San Pedro Basin	0.0299	6717	4.45
Santa Clara-Calleguas Basin	0.1455	852	170.79
Santa Rosa Valley	0.1326	487	272.28
Sea Island	0.5193	22897	22.68
South Park Basin	0.001	2706	0.36
Southern High Plains	4.039	74911	53.92
Southern Hills	0.5163	34222	15.09
Stockton Plateau	0.2408	33280	7.23

Aquifer system	Annual groundwater withdrawals estimated for the year 2015 (cubic kilometers per year)	Area within aquifer system boundaries (km^2)	Annual groundwater withdrawals estimated for the year 2015 (mm per year)*
Tifton Upland	0.2336	14671	15.92
Tijuana-San Diego Basin	0.0122	455	26.78
Trinity Aquifer System	0.2892	67113	4.31
Tulare Basin	6.013	20199	297.71
Umatilla Basin and Horse Heaven Hills	0.4004	15371	26.05
Upper Carbonate Aquifer	0.0437	11771	3.71
Upper Santa Ana Basin	0.5326	2146	248.22
Utah Lake Valley	0.1124	383	293.18
Vidalia Upland	0.2219	23846	9.31
Western Cambrian-Ordovician Aquifers	0.6543	59360	11.02
Williston Basin	0.1737	241595	0.72
Yakima Basin	0.2447	9955	24.58

* determined by dividing the value in column 2 (in units of km^3/year) by the value in column 3 (in units of km^2); next, we multiplied by 10^6 (to convert from units of km/year into units of mm/year)

The maps on the following pages display the downscaled groundwater withdrawals (for irrigation, for purposes other than irrigation, and total groundwater withdrawals for all purposes (i.e., total annual groundwater withdrawals).

We stress that the realism of the estimated irrigation withdrawals presented in the following maps relies on the accuracy of USGS county-scale groundwater withdrawal estimates. It is possible that USGS county-scale groundwater withdrawal estimates are inaccurate in places. For example, there are counties mapped as having high groundwater irrigation withdrawals where relatively small groundwater withdrawal rates occur.

One such example may be the Imperial Valley, where *"100% of the valley's water supply is imported from the Colorado River via the Imperial Dam and the All-American Canal."* (quoting https://www.usbr.gov/watersmart/bsp/docs/finalreport/secalifornia/secabasinstudy.pdf), but the USGS annual irrigation groundwater withdrawal estimate for this county is quite high (Imperial County irrigation groundwater withdrawals (field entitled "IR-WGWFr") estimated as 1303.78 Mgal/d in this dataset: https://www.sciencebase.gov/catalog/item/get/5af3311be4b0da30c1b245d8). This county-scale estimate (1303.78 MGal/d, equivalent to *1,755,000 acre-ft/year*) may be an overestimate of actual groundwater withdrawals; a different, local-scale USGS report quotes *"Groundwater pumping in the Imperial Valley was estimated to be about 25,000 acre-ft/yr (Tompson and others, 2008)"* (quoting https://pubs.usgs.gov/sir/2015/5102/sir20155102.pdf). This local-scale estimate is substantially lower than the national-scale, county-level estimate for groundwater withdrawals, though we acknowledge that Imperial County (for which groundwater withdrawals are estimated to be 1,755,000 acre-ft/year in https://www.sciencebase.gov/catalog/item/get/5af3311be4b0da30c1b245d8) is more expansive than the Imperial Basin (studied in https://pubs.usgs.gov/sir/2015/5102/sir20155102.pdf).

We emphasize that the annual groundwater withdrawal estimates we calculate and analyse via rank regressions for each of our study aquifer systems are imperfect.

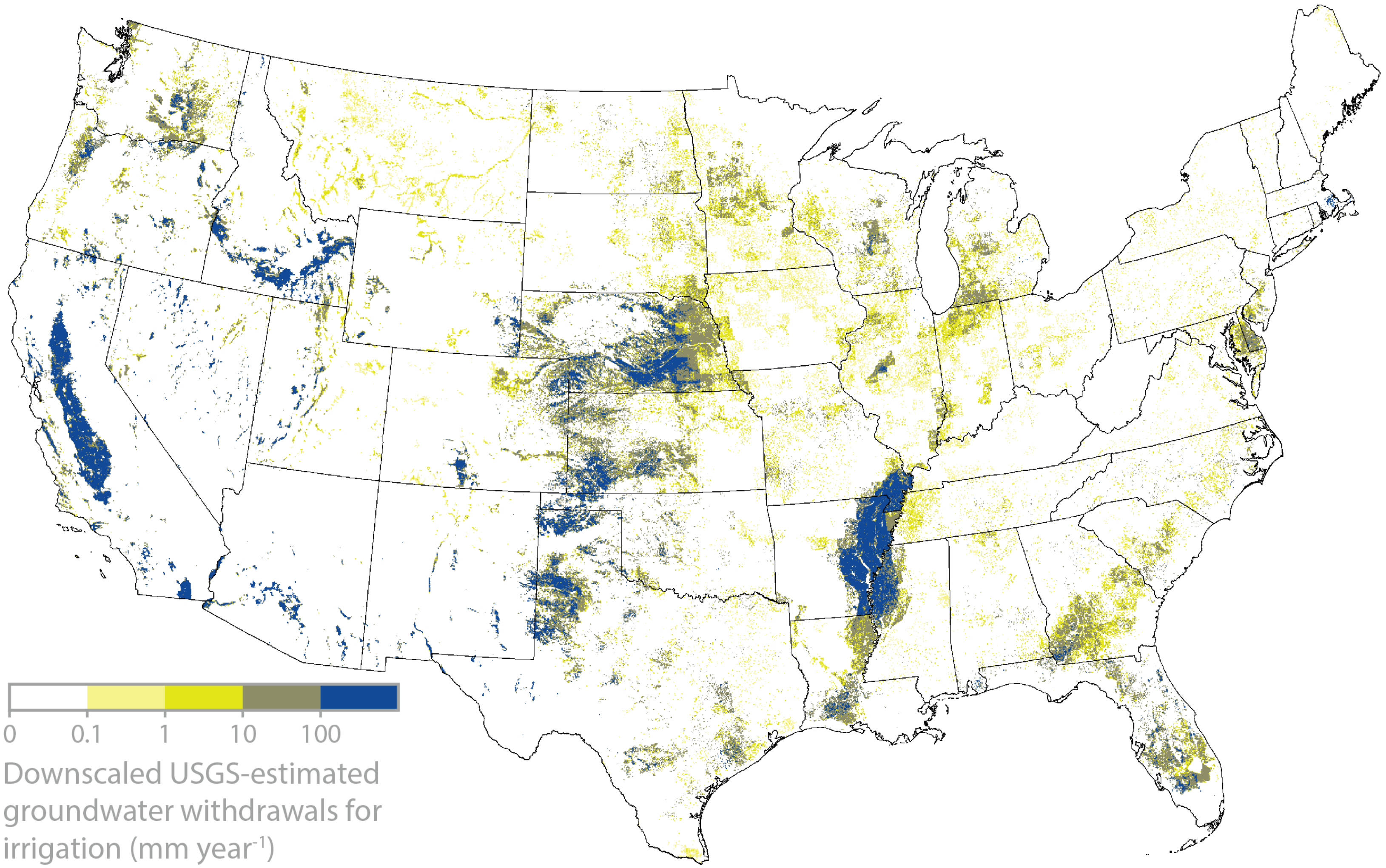

Supplementary Fig. 95. Estimated groundwater withdrawals for irrigation across the contiguous United States. Lighter coloured shades represent lower annual groundwater withdrawals; darker shades represent higher annual groundwater withdrawals Groundwater withdrawals were estimated by downscaling county-level USGS-estimated groundwater withdrawals (for the year 2015; for data see https://www.sciencebase.gov/catalog/item/get/5af3311be4b0da30c1b245d8; field title: “IR-WGWFr”) using 1 km^2 resolution MODIS-based (i.e., satellite- and land-use-data-based) irrigated lands from: https://www.sciencebase.gov/catalog/item/5db08e84e4b0b0c58b56e04f).

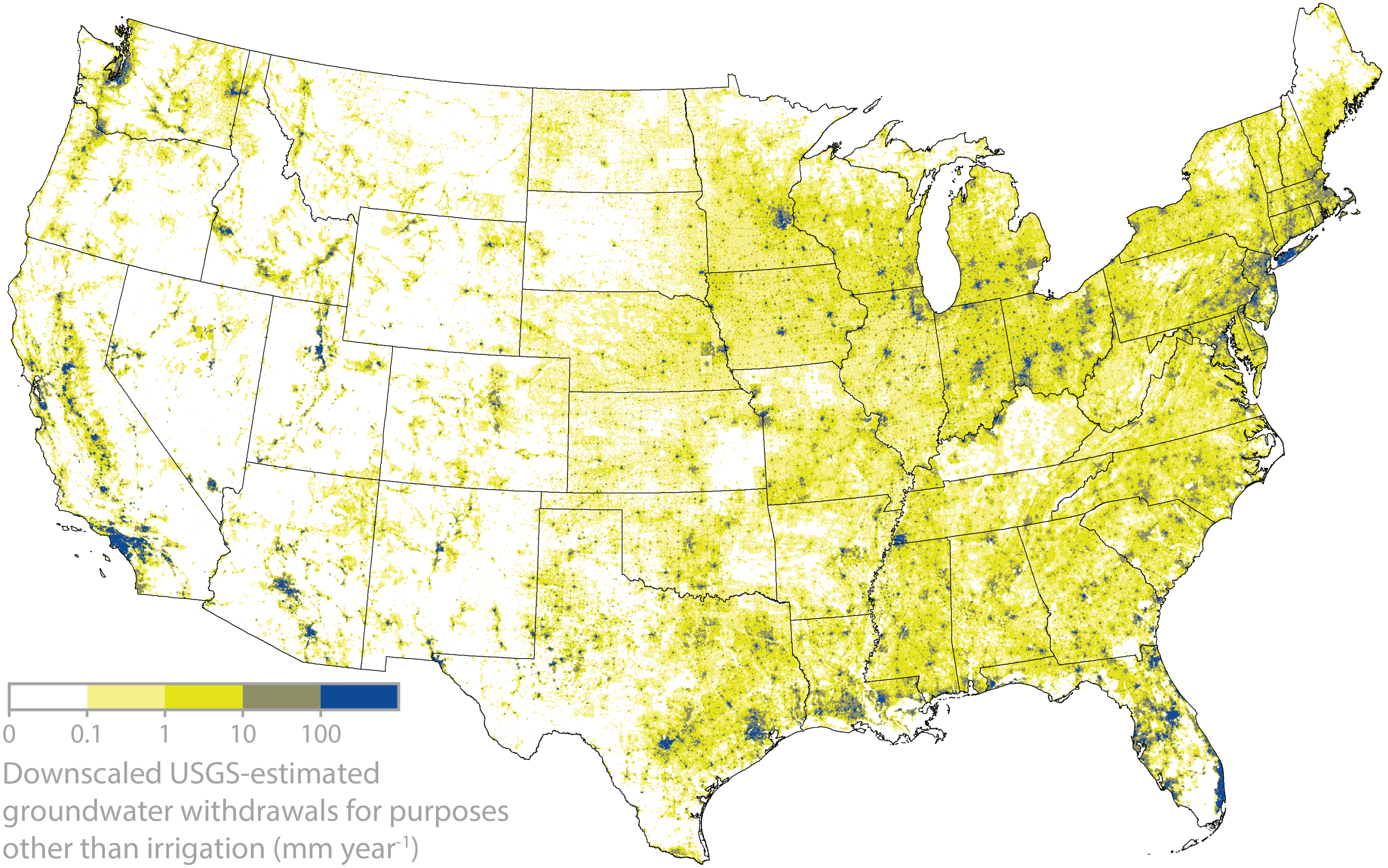

Supplementary Fig. 96. Estimated groundwater withdrawals for purposes other than irrigation across the contiguous United States. Lighter coloured shades represent lower annual groundwater withdrawals; darker shades represent higher annual groundwater withdrawals Groundwater withdrawals were estimated by downscaling county-level USGS-estimated groundwater withdrawals (for the year 2015; for data see https://www.sciencebase.gov/catalog/item/get/5af3311be4b0da30c1b245d8) using population density data (from: https://sedac.ciesin.columbia.edu/data/set/gpw-v4-population-density-rev11/data-download).

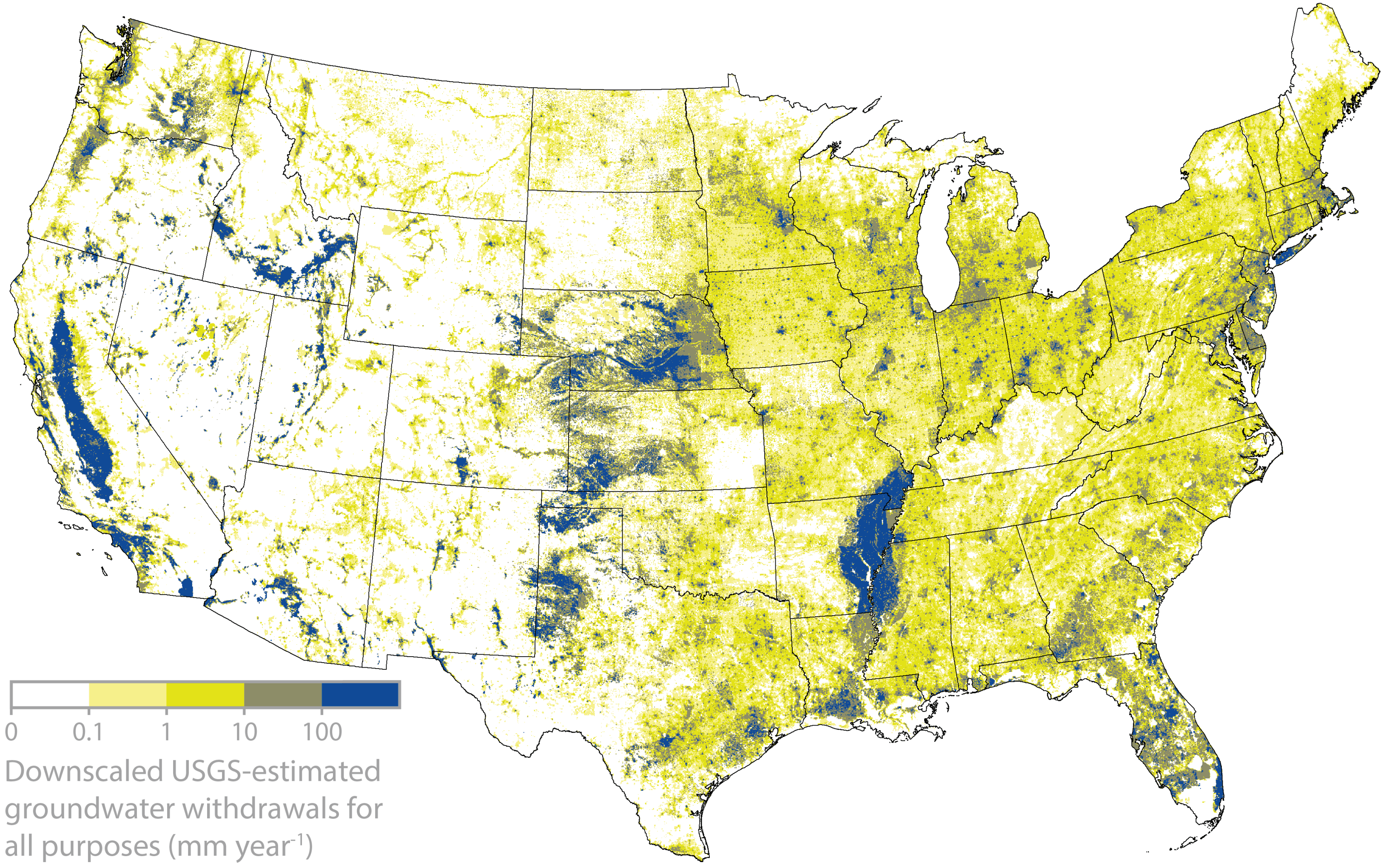

Supplementary Fig. 97. Estimated groundwater withdrawals for all purposes across the contiguous United States. Lighter coloured shades represent lower annual groundwater withdrawals; darker shades represent higher annual groundwater withdrawals Groundwater withdrawals were estimated by downscaling county-level USGS-estimated groundwater withdrawals (for the year 2015; for data see https://www.sciencebase.gov/catalog/item/get/5af3311be4b0da30c1b245d8) using population density data (from: https://sedac.ciesin.columbia.edu/data/set/gpw-v4-population-density-rev11/data-download) and irrigation spatial data (from: https://www.sciencebase.gov/catalog/item/get/5af3311be4b0da30c1b245d8).

Supplementary Note 3. Regional hydrogeology and hydrostratigraphy of study aquifer systems

Here we review local- and regional-scale hydrogeologic investigations for each of our aquifer systems to better understand the hydrostratigraphy of each study area. We provide a unique subsection for each hydrogeologic study area examined in our study (see Supplementary Notes 3.1-3.74). Our review focuses on the range of depths to the uppermost confining unit or endogenous bedrock. Our analysis is based on as many as three data sources:

(i) Analysis of hydrogeologic cross sections created on the basis of a local-scale study (see extent of transparent pink bars on cross sections in subsections to follow)

(ii) Analysis of USGS wells within the boundaries of the study aquifer where the USGS has defined the well as tapping an unconfined or a confined aquifer* *(*note: the USGS data used for this analysis derives from all NWIS wells for which such data are available, even those where tritium data are not available; data downloaded April 22, 2022 from https://waterdata.usgs.gov/nwis/inventory*

(iii) Quotes from local-scale studies that we have reviewed that provide information about the depth to confining units in the study area.

We prioritized use of data source (ii) where sufficient data were available (i.e., if sufficient USGS-defined wells were available to estimate the depth to confined conditions (data source (ii)), we used this estimate instead of data sources (i) and (iii)). Estimated depths to confined conditions for each of our n=74 study areas (i.e., aquifer systems) are summarized in Supplementary Fig. 98 and Supplementary Table 3. Specifically, we review USGS-defined well conditions (i.e., wells in unconfined vs. confined aquifers) for each study area, and we also reconstruct hydrogeologic cross sections for each of our study aquifer systems to better understand the depth to confining units in our study areas. We examine cross sections rather than 3D hydrostratigraphic data because 3D hydrostratigraphic data are unavailable for many of our study aquifers (cf. see digital thickness data for the Mississippi Embayment: https://pubs.er.usgs.gov/publication/sir20085098). We examine confining units in the context of the definition from a USGS Scientific Investigations Report: *"a hydrogeologic unit composed of clay or a series of clays that impedes or obstructs groundwater flow"* (italicized text quoted directly from Griffith, J.M. (2006). Hydrogeologic maps and sections of the "400-Foot," "600-Foot," and "800-Foot" Sands of the Baton Rouge Area and adjacent aquifers in east and west Baton Rouge, East and West Feliciana, and Pointe Coupee Parishes, Louisiana. US Geological Survey Scientific Investigations Report 2006-5072, 23 pp. Accessed April 18, 2022 via https://pubs.usgs.gov/sir/2006/5072/report.pdf); however, if a local-scale report distinguishes another type of confining unit (e.g., shale) we also include these in our reconstructed cross sections.

For a minority of our study areas we lack sufficient USGS-defined wells (i.e., depth distributions of wells that the USGS has classified as tapping either confined or unconfined conditions), we rely on cross sections to estimate the depth to confined conditions. For these study areas where we rely on cross sections to estimate the depth to confined conditions, our estimate is uncertain, as (a) cross sections may not provide adequate representation of the 3D hydrostratigraphy of the broader aquifer system, as they are necessarily constrained to only a portion of each study area, and (b) some cross sections in the primary

literature do not differentiate confining beds within a given geologic unit (e.g., see Supplementary Note 3.57 and our review of Fig. 5 by Mashburn et al. (2014) that displays the Central Oklahoma aquifer as a single hydrogeologic unit depicted as outcropping at the land surface with no overlying confining bed in their cross section, but the authors also highlight that: *"...even though the Central Oklahoma aquifer extends to land surface with a potentiometric surface below the top of the Central Oklahoma aquifer, the groundwater system acts as a confined system due to laterally extensive interbedded mudstone and large contrasts in vertical hydraulic conductivity."* (italicized text quoted directly from Mashburn, S.L., Ryter, D.W., Neel, C.R., Smith, S.J., Correll, J.S., (2014). Hydrogeology and simulation of ground-water flow in the Central Oklahoma (Garber-Wellington) Aquifer, Oklahoma, 1987 to 2009, and simulation of available water in storage, 2010–2059. US Geological Survey Scientific Investigations Report 2013–5219, 92 pp. Accessed May 14, 2021 from https://pubs.usgs.gov/sir/2013/5219/pdf/sir20135219_v2.0.pdf). We highlight that our estimated depths to confined conditions are thus approximations. These estimated depths to confined conditions are presented in Supplementary Figure 98 on the following page, and tabulated in Supplementary Table 3 in the following pages. For a map of the location of each hydrogeologic cross section see Supplementary Note 8 (and Supplementary Fig. 244).

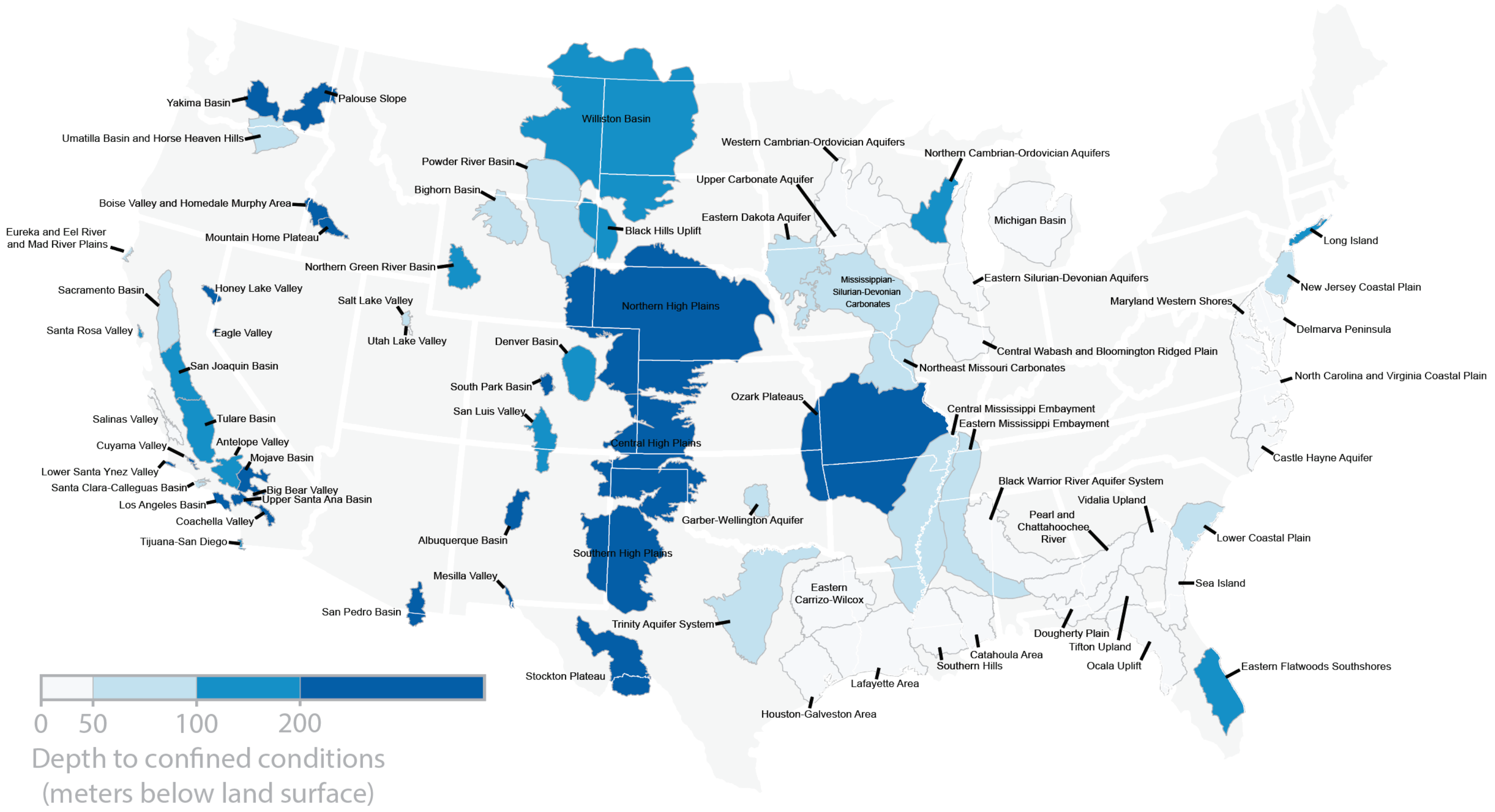

Supplementary Fig. 98. Map of our estimated depth to confined conditions in each of our n=74 study aquifer systems. Each polygon on the map represents one study area. Light blue colours represent shallower depths to confined conditions; darker blue shades represent deeper depths to confined conditions. Each aquifer system title is shown next to (or atop) the polygon. Depths to confined conditions were estimated on the basis of up to three data sources: (i) USGS-defined well conditions (i.e., wells defined as tapping unconfined versus confined conditions by the USGS), (ii) digitization and evaluation of hydrogeologic cross sections derived from local-scale reports, and (iii) quotes within local-scale reports pertaining to the prevalence of confined conditions. For details see Supplementary Notes 3.1-3.74 on the following pages.

Supplementary Table 3. The depth below which * most wells contain minimal (<25%) modern groundwater in our study aquifer systems (columns 3, 4 and 5; see footnote underneath table for quantitative definitions of "most") and physiographic, water use and climate conditions within the boundaries of each aquifer system (i.e., four potential explanatory variables are in rightmost 4 columns)

Aquifer system title	Broader aquifer system title	* 60% (m below land surface)	* 70% (m below land surface)	* 80% (m below land surface)	(i) Median depth to top of uppermost confining unit based on cross section**	(ii) Depth below which most wells have been defined by the USGS as tapping a confined aquifer***	(iii) Information from local-scale study pertaining to confined conditions	Estimated depth to confining units for the aquifer system****
**Sacramento Basin**	California Central Valley	92	114	158	The median depth to granitic and metamorphic rock is >663 meters below land surface; however, the aquifer system transitions to confined conditions at shallower depths, despite the lack of a clear confining unit (see (iii)).	Most (>90%) wells at depths of 90-100 m and at depths exceeding 100 m are defined as tapping a confined aquifer	"Groundwater is typically unconfined to semi-confined in the shallow aquifer system and confined where deeper aquifers are present." quoting Bureau of Reclamation (EIS/EIR) (2003). Environmental Water Account: Draft Environmental Impact Statement Environmental Impact Report. 317 https://usbr.gov/mp/ewa/docs/v1-draft-enviro-impact-statement-environmental-impact-report.pdf	90-100 m (see (ii) to left)
**San Joaquin Basin**	California Central Valley	134	163	203	A hydrogeologic cross section presented in Fig. 5 by Page and Balding (1973) does not depict a clear confining unit within the aquifer system.	Most (>80%) wells at depths of 140-160 m and at depths exceeding 140 m are defined as tapping a confined aquifer.	Page and Balding (1973) state (quote directly): "The confined water body occurs in the unconsolidated deposits that underlie the E-clay (fig. 6). The base of the confined water body probably is at the top of the Mehrten Formation, but in terms of use its base is considered to be the base of fresh water..."	140-160 m (see (ii) to left)
**Tulare Basin**	California Central Valley	126	220	232	A hydrogeologic cross section presented in Plate 1 by Croft (1999) suggests that the aquifer system includes a series of low permeability units, including the "E-clay" (primarily the Corcoran clay unit).	Most (>80%) wells at depths of 160-180 m and wells with depths exceeding 160 m are defined as tapping a confined aquifer.	(Croft (1999) state (quote) "Although the E clay is the principal confining bed in the valley (Davis and other, 1959, p. 87-90; Davis and Poland, 1957, p. 426), water-level data indicate that the A clay also is an effective confining bed throughout most of its extent."	160-180 m (see (ii) to left)
**Eastern Carrizo-Wilcox**	Carrizo-Wilcox	15	15	20	A hydrogeologic cross section presented in Fig. 2.4 by George (2009) depicts a series of confining units including the relatively shallow Reklaw Formation and the Weches Formation.	Most (>80%) wells at depths of 20-30 m and at depths exceeding 20 m are defined as tapping a confined aquifer.	-	20-30 m (see (ii) to left)
**Eagle Valley**	Carson River Basin	12	72	152	As we created a cross section based on a georeferenced map of basin fill thickness (by	Only half of the deepest wells in our dataset (depths exceeding 200 m) are defined as tapping a confined	Thiros et al. (2010; see section 4 by J.M. Huntington) state that (quote): "Unconfined to confined conditions	>200 m (see (ii) to left)

Aquifer system title	Broader aquifer system title	* 60% (m below land surface)	* 70% (m below land surface)	* 80% (m below land surface)	(i) Median depth to top of uppermost confining unit based on cross section**	(ii) Depth below which most wells have been defined by the USGS as tapping a confined aquifer***	(iii) Information from local-scale study pertaining to confined conditions	Estimated depth to confining units for the aquifer system****
					Arteaga, 1986), we did not quantitatively interpret the hydrogeologic cross section (as it was not explicitly based on a cross section from the primary literature).	aquifer; the deepest well in the dataset (381 m) is defined as tapping an unconfined aquifer.	are present in the basin-fill sediments…The degree of confinement varies spatially through the valley due to the clay lenses being discontinuous at different depths." and "Although groundwater exists under both confined and unconfined conditions in Carson Valley, no single confining layer extends across the entire valley… the confining layers occur mainly as scattered, discontinuous clay beds, 30 to 70 ft thick, at a depth of 200 to 300 ft."	
**Central Wabash and Bloomington Ridged Plain**	Central Lowland Till Plain	7	9	10	A hydrogeologic cross section presented in Fig. 2 by Panno et al. (1994) depicts sedimentary aquifers at shallow depths (including the Mahomet Sand member of the Banner Formation) overlying low permeability layers. In some areas carbonate rocks directly underlie the shallow sedimentary aquifers.	Most (>80%) wells at depths of 30-40 m and at depths exceeding 30 m are defined as tapping a confined aquifer.	-	30-40 m (see (ii) to left)
**Palouse Slope**	Columbia Plateau Regional Aquifer System	30	30	78	We examined a hydrogeologic cross section generated using a web application available here: https://or.water.usgs.gov/proj/cpras/index.html. No clear confining unit is depicted on the cross section.	Only half of the deepest wells in our dataset (depths exceeding 200 m) are defined as tapping a confined aquifer; the deepest well in the dataset (276 m) is defined as tapping an unconfined aquifer.	-	>276 m (see (ii) to left)
**Umatilla Basin and Horse Heaven Hills**	Columbia Plateau Regional Aquifer System	16	16	62	A hydrogeologic cross section presented in Fig. 3 by Herrera et al. (2017) suggests that the aquifer system does not have a clear confining unit. However, the aquifer system is confined at depth by basalt flow interiors (see (iii) to the right).	All (100%) wells at depths of 60-70 m and at depths exceeding 60 m are defined as tapping a confined aquifer. No wells in our dataset have depths between 25 m and 69 m; all wells (n=2) with depths of shallower than 25 m are classified as unconfined.	Herrera et al. (2017) state that (quote): "The uppermost part of the CRBG is often permeable and unconfined, and has a good hydraulic connection with the overlying alluvial aquifer and, in some cases, streams. Permeable interflow zones at depth are confined by the flow interiors."	60-70 m (see (ii) to left)
**Yakima Basin**	Columbia Plateau Regional Aquifer System	113	126	130	A hydrogeologic section by Kahle et al. (2011) demonstrates the high degree of topographic and	Three or the four deepest wells in our dataset (depths exceeding 200 m) are defined as tapping a confined	-	280-300 m (see (i) to left)

Aquifer system title	Broader aquifer system title	* 60% (m below land surface)	* 70% (m below land surface)	* 80% (m below land surface)	(i) Median depth to top of uppermost confining unit based on cross section**	(ii) Depth below which most wells have been defined by the USGS as tapping a confined aquifer***	(iii) Information from local-scale study pertaining to confined conditions	Estimated depth to confining units for the aquifer system****
					geologic complexity in the Yakima Basin. Their cross section suggests that the uppermost confining unit is 282 meters below land surface (25th-75th percentile range: 209 m to >529 m meters below land surface)	aquifer; the deepest well in the dataset (291 m, located on the northeast periphery of Grandview) is defined as tapping an unconfined aquifer.		
**Stockton Plateau**	Edwards-Trinity Aquifer System	130	-	-	A hydrogeologic cross section presented in Fig. 6-13 by Meyer et al. (2012) does not depict a clear confining unit within the aquifer system within the uppermost 190-340 m of the aquifer system (median of pink transparent pink bars suggests a lack of a clear confining unit in the uppermost 249 m below the land surface).	(ii) We analysed wells within the study area that the USGS has defined as either unconfined or confined. Only four wells were available in our dataset; they have depths ranging from 30 m to 87 m, and all are classified as tapping unconfined aquifers.	-	>249 m (see (i) to left)
**Trinity Aquifer System**	Edwards-Trinity Aquifer System	62	66	73	A hydrogeologic cross section presented in Fig. 6-38 by Bruun et al. (2016) depicts layered sequences of sedimentary aquifers and aquitards. The uppermost Washita and Fredericksburg Formations are shown as aquitards, but may contain permeable portions that serve as aquifers (see (iii) below).	Most (>80%) wells at depths of 90-100 m and at depths exceeding 90 m are defined as tapping a confined aquifer.	Bruun et al. (2016) state (quote) “Sand distribution and thickness largely controls the productivity of the aquifer. The depositional environment in the Cretaceous Period resulted in a layered system of aquifers and aquitards in the northern Trinity Group.” Mace et al. (1994) state (quote) “Hydrologic parameters for the Washita and Fredericksburg Groups… were estimated on the basis of rock type. These units are composed of approximately 40 percent shale and 60 percent limestone, as indicated by resistivity well logs…”	90-100 m (see (ii) to left)
**Dougherty Plain and Marianna Lowlands**	Floridan Aquifer System	76	113	163	A hydrogeologic cross section presented in Plate 8 by Williams and Kuniansky (2016) depicts a shallow confining unit (Hawthorn Group) underlain by carbonate aquifers and confining units.	Most (>80%) wells at depths of 30-40 m and at depths exceeding 30 m are defined as tapping a confined aquifer.	-	30-40 m (see (ii) to left)
**Eastern Flatwoods Southshores**	Floridan Aquifer System	38	46	61	A hydrogeologic cross section presented in Plate 2 by Williams and Kuniansky (2016) shows	Most (>80%) wells at depths of 100-120 m and at depths exceeding 100 m are defined as tapping a confined	-	100-120 m (see (ii) to left)

Aquifer system title	Broader aquifer system title	* 60% (m below land surface)	* 70% (m below land surface)	* 80% (m below land surface)	(i) Median depth to top of uppermost confining unit based on cross section**	(ii) Depth below which most wells have been defined by the USGS as tapping a confined aquifer***	(iii) Information from local-scale study pertaining to confined conditions	Estimated depth to confining units for the aquifer system****
					undifferentiated sand, silt and clay (~70 m thick) underlain by the Hawthorn Group confining unit.	aquifer.		
**Lower Coastal Plain**	Floridan Aquifer System	9	34	59	A hydrogeologic cross section presented in Fig. 11 by Aucott (1996) depicts a confining unit at depths of ~80 to ~150 m below the land surface.	Most (>80%) wells at depths of 90-100 m and at depths exceeding 90 m are defined as tapping a confined aquifer.	-	90-100 m (see (ii) to left)
**Ocala Uplift**	Floridan Aquifer System	103	-	-	A hydrogeologic cross section presented in Plate 2 by Williams and Kuniansky (2016) depicts the Suwannee and Ocala limestone units near the land surface but, in some places, it is overlain by a shallow confining unit (the Hawthorn Group).	Most (>80%) wells at depths of 30-40 m and at depths exceeding 30 m are defined as tapping a confined aquifer.	-	30-40 m (see (ii) to left)
**Sea Island**	Floridan Aquifer System	6	59	75	A hydrogeologic cross section presented in Plate 1 by Williams and Kuniansky (2016) depicts a shallow layer of undifferentiated sand, silt and clay underlain by a confining unit.	Most (>80%) wells at depths of 30-40 m and at depths exceeding 30 m are defined as tapping a confined aquifer.	-	30-40 m (see (ii) to left)
**Tifton Upland**	Floridan Aquifer System	9	17	52	A hydrogeologic cross section presented in Plate 8 by Williams and Kuniansky (2016) shows the Surficial Aquifer underlain by a relatively shallow confining unit (Hawthorn Group).	Most (>80%) wells at depths of 40-50 m and at depths exceeding 40 m are defined as tapping a confined aquifer.	-	40-50 m (see (ii) to left)
**Vidalia Upland**	Floridan Aquifer System	7	9	17	A hydrogeologic cross section presented in Plate 8 by Williams and Kuniansky (2016) depicts undifferentiated sand, silt and clay (less than ~20 m thick) underlain by the Hawthorn Group confining unit.	Most (>80%) wells at depths of 10-20 m and at depths exceeding 10 m are defined as tapping a confined aquifer.	-	10-20 m (see (ii) to left)
**Catahoula Area**	Gulf Coast Regional Aquifer System	73	78	-	42.8 meters below land surface (25th-75th percentile range: 16-73 meters below land surface)	The available USGS well data (n=3 wells) are insufficient to evaluate the depths at which the aquifer system transitions from unconfined to confined conditions.	"The confining units overlying the upper, middle, and lower Catahoula aquifers are primarily clays within the Catahoula, Pascagoula, and Hattiesburg Formations." quoting Halford, K. J., Barber, N. L. (1995).	40-50 m (see (i) to left)

Aquifer system title	Broader aquifer system title	* 60% (m below land surface)	* 70% (m below land surface)	* 80% (m below land surface)	(i) Median depth to top of uppermost confining unit based on cross section**	(ii) Depth below which most wells have been defined by the USGS as tapping a confined aquifer***	(iii) Information from local-scale study pertaining to confined conditions	Estimated depth to confining units for the aquifer system****
							Analysis of ground-water flow in the Catahoula aquifer system in the vicinity of Laurel and Hattiesburg, Mississippi. U.S. Geological Survey Water-Resources Investigations Report 94-4219, 78 pp. Accessed March 31, 2021 from https://pubs.usgs.gov/wri/1994/4219/report.pdf	
**Houston-Galveston Area**	Gulf Coast Regional Aquifer System	6	10	16	We analysed wells within the study area that the USGS has defined as either unconfined or confined. Most (>80%) wells at depths of 20-30 m and at depths exceeding 20 m are defined as tapping a confined aquifer.	Most (>80%) wells at depths of 20-30 m and at depths exceeding 20 m are defined as tapping a confined aquifer.	-	20-30 m (see (ii) to left)
**Lafayette Area**	Gulf Coast Regional Aquifer System	3	15	23	A hydrogeologic cross section presented in Fig. 7 by Vahdat-Aboueshagh et al. (2021) suggests that a low permeability clay-rich unit exists near to the land surface in the study area.	Most (>80%) wells at depths of 20-30 m and at depths exceeding 20 m are defined as tapping a confined aquifer.	-	20-30 m (see (ii) to left)
**Southern Hills**	Gulf Coast Regional Aquifer System	101	105	178	A hydrogeologic cross section presented in Fig. 4 by USGS (2017) depicts a series of interbedded aquifers and low-permeability units.	Most (>80%) wells at depths of 20-30 m and at depths exceeding 20 m are defined as tapping a confined aquifer.	-	20-30 m (see (ii) to left)
**Central High Plains**	High Plains	35	59	78	A hydrogeologic cross section presented by Macfarlane (1996) suggests that the uppermost confining unit is 43 meters below the land surface (25th-75th percentile range: 0-97 meters below land surface).	Nearly all wells with depths of less than 300 m are defined as tapping unconfined aquifers. All wells (n=3) at depths of 300-340 m are defined as tapping a confined aquifer (Dakota Sandstone aquifer).	-	300-340 m (see (ii) to left)
**Northern High Plains**	High Plains	20	35	64	A hydrogeologic cross section presented in Fig. 2 by Hallum et al. (2019) depicts relatively deep depths (>200 m) to the uppermost relatively low-permeability unit.	Nearly all wells with depths of less than 240 m are defined as tapping unconfined aquifers. One well with a depth of 300 m is defined as tapping a confined aquifer (Dakota Sandstone aquifer).	-	>240 m (see (ii) to left)
**Southern High Plains**	High Plains	89	99	117	A hydrogeologic cross section presented in Fig. 15 by Blandford	Nearly all wells are defined as tapping an unconfined aquifer,	-	>270 m (see (ii) to left)

Aquifer system title	Broader aquifer system title	* 60% (m below land surface)	* 70% (m below land surface)	* 80% (m below land surface)	(i) Median depth to top of uppermost confining unit based on cross section**	(ii) Depth below which most wells have been defined by the USGS as tapping a confined aquifer***	(iii) Information from local-scale study pertaining to confined conditions	Estimated depth to confining units for the aquifer system****
					et al. (2008) suggests that the aquifer system does not depict a clear confining unit within the shallow (<200 m) portion of the aquifer system.	including the deepest well in the dataset (274 m).		
**Albuquerque Basin**	Middle Rio Grande	29	87	117	A hydrogeologic cross section presented in Plate 2 by Connell (2006) depicts thick (>200 m) poorly consolidated aquifers with a high density of faulting.	Nearly all wells are defined as tapping an unconfined aquifer, including the deepest well in the dataset (631 m).	-	>630 m (see (ii) to left)
**San Luis Valley**	Middle Rio Grande	107	-	-	A hydrogeologic cross section presented in Fig. 5 by Leonard and Watts (1989) does not depict a clear confining unit within the aquifer system. However, the hydrogeologic cross section in Plate 6 by Repplier et al. (1981) does depict a shallow confining unit (*note: this cross section is incorrectly labelled in the cited publication as “Raton Basin”; we confirmed that this cross section represents the San Luis Valley (not the Raton Basin) via personal communication with a Senior Hydrogeologist at the Colorado Geological Survey on April 18, 2022). We added a confining unit to the cross section (top left of this page) on the basis of	Most (>80%) wells at depths of 140-160 m and at depths exceeding 140 m are defined as tapping a confined aquifer.	-	140-160 m (see (ii) to left)
**Central Mississippi Embayment**	Mississippi Embayment	33	37	45	A hydrogeologic cross section presented in Figs. 68 and 69 by Renken (1998) suggests that the uppermost confining unit (named the Cook Mountain Formation, in the area where the cross section is located) is typically 75 meters below land surface (median vertical offset of the 20 pink transparent bars in the cross section to the left; the 25th-75th percentile range is 49-94 meters below land surface).	Most (>80%) wells at depths of 70-80 m and at depths exceeding 70 m are defined as tapping a confined aquifer.	-	70-80 m (see (i) and (ii) to left)

Aquifer system title	Broader aquifer system title	* 60% (m below land surface)	* 70% (m below land surface)	* 80% (m below land surface)	(i) Median depth to top of uppermost confining unit based on cross section**	(ii) Depth below which most wells have been defined by the USGS as tapping a confined aquifer***	(iii) Information from local-scale study pertaining to confined conditions	Estimated depth to confining units for the aquifer system****
**Eastern Mississippi Embayment**	Mississippi Embayment	19	29	58	A hydrogeologic cross section presented in Figs. 68 and 69 by Renken (1998) suggests that the uppermost confining unit (named the Cook Mountain Formation, in the area where the cross section is located) is typically 75 meters below land surface (median vertical offset of the 20 pink transparent bars in the cross section to the left; the 25th-75th percentile range is 49-94 meters below land surface).	Most (>80%) wells at depths of 70-80 m and at depths exceeding 70 m are defined as tapping a confined aquifer.	Renken (1998) state that (following text quoted directly): "The alluvial aquifers consist of gravel and sand deposits of Quaternary age and generally contain ground water under unconfined conditions".	70-80 m (see (ii) to left)
**Delmarva Peninsula**	North Atlantic Coastal Plain	15	21	29	A hydrogeologic cross section presented in Fig. 21 of Sanford et al. (2012) depicts a thin (less than ~50 m) shallow aquifer underlain by a layered aquifer system consisting of aquitards and aquifers.	Most (>80%) wells at depths of 30-40 m and at depths exceeding 30 m are defined as tapping a confined aquifer.	-	30-40 m (see (ii) to left)
**Maryland Western Shores**	North Atlantic Coastal Plain	8	26	47	A hydrogeologic cross section presented in Figs. 4-6 by Vroblesky et al. (1991) depicts a shallow geologic formation with both permeable and less-permeable subunits (undivided).	Most (>80%) wells at depths of 30-40 m and at depths exceeding 30 m are defined as tapping a confined aquifer.	-	30-40 m (see (ii) to left)
**New Jersey Coastal Plain**	North Atlantic Coastal Plain	48	53	58	A hydrogeologic cross section presented in Fig. 10 of Masterson et al. (2013) shows a layered aquifer system including clastic sedimentary aquifers and aquitards of varying thicknesses.	Most (>80%) wells at depths of 80-90 m and at depths exceeding 80 m are defined as tapping a confined aquifer.	-	80-90 m (see (ii) to left)
**North Carolina and Virginia Coastal Plain**	North Atlantic Coastal Plain	5	8	13	A hydrogeologic cross section presented in Plate 9 by Winner Jr. and Coble (1989) depicts a shallow suficial aquifer underlain by a confining unit at depths of less than ~50 m below the land surface.	Most (>80%) wells at depths of 30-40 m and at depths exceeding 30 m are defined as tapping a confined aquifer.	-	30-40 m (see (ii) to left)
**Powder River Basin**	Northern Great Plains	4	16	30	A hydrogeologic cross section presented in Fig. 4 by Long et al. (2018) depicts relatively shallow confining units in some areas,	Most (>80%) wells at depths of 60-70 m and at depths exceeding 60 m are defined as tapping a confined aquifer.	-	60-70 m (see (ii) to left)

Aquifer system title	Broader aquifer system title	* 60% (m below land surface)	* 70% (m below land surface)	* 80% (m below land surface)	(i) Median depth to top of uppermost confining unit based on cross section**	(ii) Depth below which most wells have been defined by the USGS as tapping a confined aquifer***	(iii) Information from local-scale study pertaining to confined conditions	Estimated depth to confining units for the aquifer system****
					and deeper depths to the uppermost confining unit in other areas.			
**Williston Basin**	Northern Great Plains	7	16	28	A hydrogeologic cross section presented in Fig. 4 by Long et al. (2018) depicts complex layered sedimentary sequences, with relatively deep depths to the uppermost confining unit in the northeast portion of the hydrogeologic study area.	Most (>80%) wells at depths of 140-160 m and at depths exceeding 140 m are defined as tapping a confined aquifer.	-	140-160 m (see (ii) to left)
**Eastern Silurian-Devonian Aquifers**	Northern Midwest Aquifer System	14	16	29	A hydrogeologic cross section presented in Fig. 20 by Young (1992) shows a drift aquifer atop the Silurian-Devonian carbonates, underlain by the Maquoketa confining unit.	Most (>80%) wells at depths of 30-40 m and at depths exceeding 30 m are defined as tapping a confined aquifer.	-	30-40 m (see (ii) to left)
**Mississippian-Silurian-Devonian Carbonates**	Northern Midwest Aquifer System	14	32	77	A hydrogeologic cross section presented in Fig. 20 by Young (1992) shows Pleistocene deposits overlying Mississippian- to Silurian-aged carboante rocks atop a series of confining units.	Most (>80%) wells at depths of 60-70 m and at depths exceeding 60 m are defined as tapping a confined aquifer.	-	60-70 m (see (ii) to left)
**Northeast Missouri Carbonates**	Northern Midwest Aquifer System	41	70	-	A hydrogeologic cross section presented in Plate 1 by Young (1992) depicts layered clastic and carbonate sedimentary units with relatively shallow confining units in most of the area.	Most (>80%) wells at depths of 50-60 m and at depths exceeding 50 m are defined as tapping a confined aquifer.	-	50-60 m (see (ii) to left)
**Northern Cambrian-Ordovician Aquifers**	Northern Midwest Aquifer System	59	73	105	A hydrogeologic cross section in Plate 1 by Young (1992) depicts sedimentary deposits overlying Precambrian bedrock.	Most (>80%) wells at depths of 160-180 m and at depths exceeding 160 m are defined as tapping a confined aquifer.	-	160-180 m (see (ii) to left)
**Upper Carbonate Aquifer**	Northern Midwest Aquifer System	93	134	140	A hydrogeologic cross section presented in Fig. 4 by Savoca et al. (1999) depicts Quaternary deposits (~50 m thick) overlying a carbonate rock aquifer (~100-150 m thick).	Most (>80%) wells at depths of 20-30 m and at depths exceeding 20 m are defined as tapping a confined aquifer.	Savoca et al. (1999) write (quote) “The Upper carbonate aquifer underlies the northern part of the study area and consists of 250 to 600 ft of Ordovician- and Devonian-age limestone, dolomite, dolomitic limestone, and shale (table 1; fig. 4, hydrogeologic section A-A') of shallow marine origin. The aquifer is	20-30 m (see (ii) to left)

Aquifer system title	Broader aquifer system title	* 60% (m below land surface)	* 70% (m below land surface)	* 80% (m below land surface)	(i) Median depth to top of uppermost confining unit based on cross section**	(ii) Depth below which most wells have been defined by the USGS as tapping a confined aquifer***	(iii) Information from local-scale study pertaining to confined conditions	Estimated depth to confining units for the aquifer system****
							overlain by unconsolidated Quaternary- and Cretaceous-age deposits (sand, gravel, and clay) and is unconfined except in areas where overlying fine-grained deposits produce locally confined conditions”	
**Western Cambrian-Ordovician Aquifers**	Northern Midwest Aquifer System	56	87	103	A hydrogeologic cross section presented in Fig. 5 by Seaberg (2000) depicts layered sedimentary sequences including discontinuous aquitards (Glenwood-Platteville-Decorah units) and more continuous aquitards (e.g., St. Lawrence Confining Bed).	Most (>80%) wells at depths of 30-40 m and at depths exceeding 30 m are defined as tapping a confined aquifer.	-	30-40 m (see (ii) to left)
**Mesilla Valley**	Rincon-Mesilla Valleys	37	64	93	A hydrogeologic cross section presented in Fig. 3 by Robertson et al. (2022) does not depict a clear confining unit within the shallow portion of the aquifer system.	Most (>80%) wells at depths of 240-260 m and at depths exceeding 240 m are defined as tapping a confined aquifer.	-	240-260 m (see (ii) to left)
**Lower Santa Ynez Valley**	Santa Ynez Valley	61	65	71	A hydrogeologic cross section presented in Fig. 2a.2 5a by the Western Management Area Groundwater Sustainability Agency (2022) suggests that the uppermost confining unit is typically 202 meters below land surface (25th-75th percentile range: 56-243 meters below land surface).	All n=4 wells (with depths ranging from 49 m to 117 m) are classified as unconfined. The available USGS well data are insufficient to evaluate the depths at which the aquifer system transitions from unconfined to confined conditions.	Regarding confined conditions, the Western Management Area Groundwater Sustainability Agency (2022) states (quote) “The main zone throughout most of the Lompoc Plain subarea is separated from the middle zone by lenses of silt and clay that result in confined or partially confined conditions in the main zone. However, in the eastern, southern, and northern portions of the Lompoc Plain subarea, the confining deposits are less continuous or absent…”	200-220 m (see (i) and (iii) to left)
**Boise Valley and Homedale Murphy Area**	Western Snake River Plain	98	191	-	A hydrogeologic cross section presented in Fig. 39 by Lindholm (1996) suggests that the upper unit (primarily sand and gravel) is underlain by a confining unit (“Middle unit”).	Two of the three deepest wells in the dataset (depths range from 209 m to 372 m) as classified as unconfined; the deepest well (372 m deep) is classified as confined. The available USGS well data are insufficient to evaluate the depths at which the aquifer system transitions from	-	>260 m (see (ii) to left)

Aquifer system title	Broader aquifer system title	* 60% (m below land surface)	* 70% (m below land surface)	* 80% (m below land surface)	(i) Median depth to top of uppermost confining unit based on cross section**	(ii) Depth below which most wells have been defined by the USGS as tapping a confined aquifer***	(iii) Information from local-scale study pertaining to confined conditions	Estimated depth to confining units for the aquifer system****
						unconfined to confined conditions.		
**Mountain Home Plateau**	Western Snake River Plain	127	131	139	A hydrogeologic cross section presented in Fig. 4 by Norton et al. (1982) for the Mountain Home Plateau does not depict a clear confining unit. The median depth to a confined unit exceeds 336 meters below land surface (median of pink transparent bars in cross section; the 25th-75th percentile range is >336 m to >351 m).	There is only n=1 well (with a depth of 112 m) that the USGS has defined as tapping either a confined or unconfined aquifer; this single data point is insufficient to evaluate the depths at which the aquifer system transitions from unconfined to confined conditions.	-	>336 m (see (ii) to left)
**Antelope Valley**	-	2	18	84	A hydrogeologic cross section presented in Fig. 3 by Duell Jr. (1987) suggests that shallow unconsolidated deposits are underlain by a confining unit comprised of lacustrine clay.	Most (>80%) wells at depths of 120-140 m and at depths exceeding 120 m are defined as tapping a confined aquifer.	-	120-140 m (see (ii) to left)
**Big Bear Valley**	-	122	163	-	282 meters below land surface (25th-75th percentile range: 121-336 meters below land surface)	There are no USGS wells within the study area that have been defined as tapping an aquifer that is either unconfined or confined.	-	280-300 m (see (i) to left)
**Bighorn Basin**	-	61	61	69	A hydrogeologic cross section presented in Plate VI by Tauchen et al. (2012) suggests that the aquifer system does not have a single and continuous confining unit at shallow depths.	Most (>80%) wells at depths of 70-80 m and at depths exceeding 120 m are defined as tapping a confined aquifer.	-	70-80 m (see (ii) to left)
**Black Hills Uplift**	-	398	545	666	A hydrogeologic cross section presented in Fig. 25 by Driscoll et al. (2002) depicts a series of sedimentary rock units dipping to the east, with numerous confining units including the Spearfish confining unit and the Opeche confining unit.	Most (>80%) wells at depths of 180-200 m and at depths exceeding 180 m are defined as tapping a confined aquifer.	-	180-200 m (see (ii) to left)
**Black Warrior River Aquifer System (Eutaw and McShan Formations and**	-	5	15	40	A hydrogeologic cross section presented in Fig. 3 by Strom and Mallory (1995) suggests that the shallowest confining unit is typically 3.5 m below the land surface, but with wide variability	Most (>80%) wells at depths of 0-10 m and at depths exceeding 0 m are defined as tapping a confined aquifer.	-	0-10 m (see (ii) to left)

Aquifer system title	Broader aquifer system title	* 60% (m below land surface)	* 70% (m below land surface)	* 80% (m below land surface)	(i) Median depth to top of uppermost confining unit based on cross section**	(ii) Depth below which most wells have been defined by the USGS as tapping a confined aquifer***	(iii) Information from local-scale study pertaining to confined conditions	Estimated depth to confining units for the aquifer system****
**Tuscaloosa Group)**					along the cross section (25th-75th percentile range of the depth to uppermost confining unit is 0m to 255 m).			
**Castle Hayne Aquifer**	-	24	34	49	A hydrogeologic cross section presented in Plate 6 by Winner Jr. and Coble (1989) depicts shallow low-permeability units including the Castle Hayne Confining Unit, Beaufort Confining Unit, and Peedee Confining Unit.	Most (>80%) wells at depths of 10-20 m and at depths exceeding 10 m are defined as tapping a confined aquifer.	-	10-20 m (see (ii) to left)
**Coachella Valley**	-	232	277	323	A hydrogeologic cross section presented in Fig. 3-3 by MWH (2014) suggests that a relatively continuous aquitard exists in the southeastern portion of the Coachella Valley, but that this unit is absent in the northwestern portion of the area.	(ii) We analysed wells within the study area that the USGS has defined as either unconfined or confined. Nearly all (11 of 12) wells in the Coachella Valley are defined as unconfined, including the deepest wells in the dataset (depths of 323 m and 341 m).	-	>341 m (see (ii) to left)
**Cuyama Valley**	-	67	189	-	The median depth to a confined unit or endogenous bedrock is >722 m.	The available USGS well data (n=2 wells with depths of 71 m and 183 m) are insufficient to evaluate the depths at which the aquifer system transitions from unconfined to confined conditions.	"The aquifer is considered to be continuous and unconfined with the exception of locally perched aquifers resulting from clays in the formations."	>722 m (see (i) and (iii) to left)
**Denver Basin**	-	34	46	60	A hydrogeologic cross section presented in Fig. 3 by Malenda and Penn (2020) suggests the top of the uppermost confining unit (the Laramie Formation) is typically 297 meters below the land surface.	Most (>80%) wells at depths of 160-180 m and at depths exceeding 160 m are defined as tapping a confined aquifer.	Malenda and Penn (2020) highlight that confined conditions can exist in the Denver Formation, which overlies the Laramie Formation; they state that "The three Denver aquifer wells represent confined aquifer conditions". Therefore, we do not rely solely on the depth to the Laramie Formation to estimate the depth to confined conditions, instead drawing information from the wells where the USGS has defined whether the well taps unconfined versus confined conditions.	160-180 m (see (ii) to left)
**Eastern Dakota Aquifer**	-	12	21	30	A hydrogeologic cross section presented in the figure on page 27 by Prior et al. (2003) depicts	Most (>80%) wells at depths of 60-70 m and at depths exceeding 60 m are defined as tapping a confined	-	60-70 m (see (ii) to left)

Aquifer system title	Broader aquifer system title	* 60% (m below land surface)	* 70% (m below land surface)	* 80% (m below land surface)	(i) Median depth to top of uppermost confining unit based on cross section**	(ii) Depth below which most wells have been defined by the USGS as tapping a confined aquifer***	(iii) Information from local-scale study pertaining to confined conditions	Estimated depth to confining units for the aquifer system****
					relatively thick unconsolidated deposits overlying the Dakota Aquifer, which itself overlies dipping sedimentary sequences including several aquitards (e.g., the Upper and Basal Devonian Aquitards).	aquifer.		
**Eureka and Eel River and Mad River Plains**	-	59	66	-	605 meters below land surface (25th-75th percentile range: 421-757 meters below land surface)	There are no USGS wells within the study area that have been defined as tapping an aquifer that is either unconfined or confined.	"The two younger units, the Scotia Bluffs Sandstone and Carlotta Formation, consist dominantly of coarse-grained clastic sediments of marginal marine deposition and may be important aquifers. Water found in these two formations in the Eureka area is generally confined by interbeds of silt and clay of low permeability or by the over-lying, fine-grained sediment in the Hookton Formation" quoting Johnson, M. J. (1975). Ground-water conditions in the Eureka Area, Humboldt County, California. U.S. Geological Survey Water-Resources Investigations 78-127. 51 pp. Accessed March 20, 2021 from https://pubs.usgs.gov/wri/1978/0127/report.pdf	70-80 m [x] (see (iii) to left)
**Garber-Wellington Aquifer**	-	37	49	60	A hydrogeologic cross section presented in Fig. 5 by Mashburn et al. (2014) does not depict a clear confining unit in the central portion of the study area (but see quote in (iii) to the right).	Most (>80%) wells at depths of 80-90 m and at depths exceeding 80 m are defined as tapping a confined aquifer.	Mashburn et al. (2014) state that (following text quoted directly): "…even though the Central Oklahoma aquifer extends to land surface with a potentiometric surface below the top of the Central Oklahoma aquifer, the groundwater system acts as a confined system due to laterally extensive interbedded mudstone and large contrasts in vertical hydraulic conductivity."	80-90 m (see (ii) to left)
**Honey Lake Valley**	-	31	75	-	(i) A hydrogeologic cross section presented in Fig. 2 by Mayo et al. (2010) suggests that the unconfined portion of Honey Lake Valley extends to a typical depth of greater than 683 m meters below land surface	There are no USGS wells within the study area that have been defined as tapping an aquifer that is either unconfined or confined.	"Three groundwater systems have been identified in Honey Lake basin: (1) shallow unconfined and semiconfined (<200 m below ground surface (bgs)), (2) deep confined (>200 m bgs), and (3) geothermal" quoting Mayo, A. L., Henderson, R.	200-220 m based on (iii)

Aquifer system title	Broader aquifer system title	* 60% (m below land surface)	* 70% (m below land surface)	* 80% (m below land surface)	(i) Median depth to top of uppermost confining unit based on cross section**	(ii) Depth below which most wells have been defined by the USGS as tapping a confined aquifer***	(iii) Information from local-scale study pertaining to confined conditions	Estimated depth to confining units for the aquifer system****
					(median of pink bars shown in cross section to the left).		M., Tingey, D., Webber, W. (2010). Chemical evolution of shallow playa groundwater in response to post-pluvial isostatic rebound, Honey Lake Basin, California–Nevada, USA. Hydrogeology Journal, 18, 725-747.	
**Long Island**	-	101	138	149	A hydrogeologic cross section presented by Smolensky et al. (1990) suggests that the aquifer system has two primary confining units, the Gardiners Clay and the Raritan Confining Unit.	Most (>80%) wells at depths of 120-140 m and at depths exceeding 120 m are defined as tapping a confined aquifer.	-	120-140 m (see (ii) to left)
**Los Angeles Basin**	-	160	346	397	A hydrogeologic cross section presented in Fig. 4 by Reichard et al. (2003) does not depict a continuous confining unit in the study area.	We could not identify a depth where most (>80%) wells with the depth range and at deeper depths are defined as tapping a confined aquifer. Three of the four deepest wells in our dataset (depths of 396 m, 399 m, 445 m, and 472 m) are classified as unconfined.	-	>472 m (see (ii) to left)
**Michigan Basin**	-	89	-	-	A hydrogeologic cross section presented in Fig. 7 by Westjohn et al. (1998) depicts a series of stacked confining units underlying relatively thick (>100 m) glacial deposits.	Most (>80%) wells at depths of 40-50 m and at depths exceeding 40 m are defined as tapping a confined aquifer.	-	40-50 m (see (ii) to left)
**Mojave Basin**	-	1	31	60	A hydrogeologic cross section presented in Fig. 2a by Kulongoski et al. (2003) does not depict a clear confining unit within the aquifer system. The median depth to basement igneous and metamorphic rocks as depicted in the cross section is >512 m.	We could not identify a depth where most (>80%) wells with the depth range and at deeper depths are defined as tapping a confined aquifer. The two deepest wells in our dataset (depths of 228 m and 314 m) are classified as unconfined.	Kulongoski et al. (2003) highlight that unconfined conditions prevail in parts of the aquifer system; they state (following text quoted directly): “These deposits (QTa) consist of unconsolidated to moderately consolidated gravel, sand, silt, and clay deposited in the Pleistocene and late Pliocene, and overlie a crystalline complex of igneous and metamorphic rocks (pTb), (California Dept. Of Water Resources, 1967). Waters from this mostly unconfined aquifer…”	>512 m (see (ii) to left)
**Northern Green River**	-	27	49	82	Fig. 7 by Bartos et al. (2015) suggests that the top if the	The available USGS well data are insufficient to evaluate the depths at	Bartos et al. (2015) state, with regards to the presence of confining	180-200 m (see (i) to left)

Aquifer system title	Broader aquifer system title	* 60% (m below land surface)	* 70% (m below land surface)	* 80% (m below land surface)	(i) Median depth to top of uppermost confining unit based on cross section**	(ii) Depth below which most wells have been defined by the USGS as tapping a confined aquifer***	(iii) Information from local-scale study pertaining to confined conditions	Estimated depth to confining units for the aquifer system****
**Basin**					uppermost confining unit is typically 193 meters below land surface (i.e., the median length of the pink transparent bars in cross section; the lower-upper quartile range is 137-225 meters).	which the aquifer system transitions from unconfined to confined conditions. Wells with depths of 335 m and 416 m are classified as confined, where the shallowest wells (all with depths of less than 26 m) are classified as unconfined.	units, that (quote) *"Confining units are the Wilkins Peak and Tipton confining units; where impermeable, the Laney Member of the Green River Formation also is a confining unit."* As the Laney Formation overlies the Wilkins Formation, it is possible that confined conditions prevail at shallower depths than the top of the Wilkins Formation. However, without spatial data defining low-permeability parts of the Laney Formation, we cannot shorten the length of the pink bars depicted on the cross section such that their bottoms are shallower than the top of the Wilkins Peak Formation. Our estimated depth to confined conditions may overestimate actual depths to confined conditions in some areas.	
**Ozark Plateaus Aquifer System**	-	66	113	192	A hydrogeologic cross section presented in Fig. 3 by Clark et al. (2019) shows thick sequences of carbonate rock with interbedded confining units. The St. Francois confining unit exists at deep depths (>300 m) and, in the northwest, a shallower and thin confining unit exists (the Ozark confining unit).	Most (>80%) wells at depths of 940-960 m and at depths exceeding 940 m are defined as tapping a confined aquifer.	-	940-960 m (see (ii) to left)
**Pearl and Chattahoochee Aquifer System**	-	38	55	128	(i) A hydrogeologic cross section presented in Fig. 3 by Kidd (1987) depicts a series of dipping sedimentary formations. Note that the cross section presented by Kidd (1987) lacks a quantitative vertical axis; whereas the cross sections we consulted for the other n=73 aquifer systems have quantative vertical axes. We scaled the vertical scale of the cross section by Kidd (1987) by matching the topography depicted in the figure with an elevation profile along the cross section path. We stress	Most (>80%) wells at depths of 20-30 m and at depths exceeding 20 m are defined as tapping a confined aquifer.	-	20-30 m (see (ii) to left)

Aquifer system title	Broader aquifer system title	* 60% (m below land surface)	* 70% (m below land surface)	* 80% (m below land surface)	(i) Median depth to top of uppermost confining unit based on cross section**	(ii) Depth below which most wells have been defined by the USGS as tapping a confined aquifer***	(iii) Information from local-scale study pertaining to confined conditions	Estimated depth to confining units for the aquifer system****
					that this cross section is more uncertain than others; we did not use the cross section for this study area to estimate the depth to confined conditions (i.e., we use data source (ii) described to the right).			
**Salinas Valley**	-	89	195	222	A hydrogeologic cross section presented in Fig. 3 by Hall (1992) demonstrates widespread confining beds (e.g., the Salinas Valley Aquitard in the northwest of the valley). The median depth to the top of the uppermost confined unit is 31 meters (25th-75th percentile range is 9 meters to 49 meters below land surface; see pink transparent bars in cross section to the left).	There are no USGS wells within the study area that have been defined as tapping an aquifer that is either unconfined or confined.	Hall (1992) states, with respect to confining conditions, (quote): "The blue clay, overlying the 180 foot aquifer, ranges from 25 feet thick at Salinas to more than 100 feet thick at Nashua Road. Is it known as the Salinas Aquitard and is composed mostly of blue marine clays with some silts". A report by Harding ESE (2001) on the hydrostratigraphy area states that (quote): "The lateral extent of the 180-Foot Aquifer is generally defined by the overlying [Salinas Valley Aquitard] clay, which maintains confined conditions throughout much of the study area." Vengosh et al. (2002) state that: "The Salinas Valley has a relatively deep, confined ''400-foot'' aquifer, overlain by a ''180-foot'' aquifer and a shallower perched aquifer, all made up of alluvial sand, gravel and clay deposits"	30-40 m (see (i) to left)
**Salt Lake Valley**	-	294	-	-	A hydrogeologic cross section presented in Fig. 4 by Manning and Solomon (2005) labels a fine-grained confining layer at shallow depths (<100 m) across much of the study area.	Most (>80%) wells at depths of 50-60 m and at depths exceeding 50 m are defined as tapping a confined aquifer.	-	50-60 m (see (ii) to left)
**San Pedro Basin**	-	201	244	280	A hydrogeologic cross section presented in Fig. 2 by Hopkins et al. (2014) depicts a clay layer in the central portion of the basin.	Most (>80%) wells at depths of 300-320 m and at depths exceeding 300 m are defined as tapping a confined aquifer.	-	300-320 m (see (ii) to left)
**Santa Clara-Calleguas Basin**	-	77	102	226	A hydrogeologic cross section presented in Fig. 8 by Hanson et al. (2002) depicts a multi-layered aquifer system with a confining	Most (>80%) wells at depths of 50-60 m and at depths exceeding 50 m are defined as tapping a confined aquifer.	-	50-60 m (see (ii) to left)

Aquifer system title	Broader aquifer system title	* 60% (m below land surface)	* 70% (m below land surface)	* 80% (m below land surface)	(i) Median depth to top of uppermost confining unit based on cross section**	(ii) Depth below which most wells have been defined by the USGS as tapping a confined aquifer***	(iii) Information from local-scale study pertaining to confined conditions	Estimated depth to confining units for the aquifer system****
					unit relatively close to the coast.			
**Santa Rosa Valley**	-	31	70	209	In the hydrogeologic cross section in Fig. 2-9 by Santa Rosa Plain Advisory Panel (2014), the median depth to the top of the Petaluma Formation—which is depicted as low-permeability by Woolfenden and Nishikawa (2014) in their Fig. 5—is 111 m (25th-75th percentile range of the depth to the top of the Petaluma Formation is 68 to 132 meters below land surface).	There are no USGS wells within the study area that have been defined as tapping an aquifer that is either unconfined or confined.	Santa Rosa Plain Advisory Panel (2014) state that (following text quoted directly): "In most parts of the study area, shallow groundwater flow is unconfined, but, at depth, groundwater flow is confined." Further, Woolfenden and Nishikawa (2014) states (quote): "The Petaluma Formation is dominated by fine-grained materials, either in thick beds or as interstitial material in poorly sorted silty and clayey sands or gravels." (see also their conceptual model in Fig. 5 by Woolfenden and Nishikawa (2014) depicting the Petuluma Formation as a low-permeability formation).	100-120 m (see (i) to left)
**South Park Basin**	-	5	12	67	A hydrogeologic cross section presented in Fig. 11 by Barkmann et al. (2013) demonstrates the complexity of the sedimentary rock sequences in the South Park Basin. Among the 20 evenly spaced pink transparent bars (the length of each representing the depth to the uppermost confining unit), the median depth to a confining unit is >576 meters below land surface (25th-75th percentile range: 224 m to >619 m).	All wells (n=52) in the South Park Basin (depths of 0.15 m to 183 m) are classified as unconfined.	-	>576 m (based on (i), consistent with data presented in (ii))
**Tijuana-San Diego Basin**	-	8	8	37	A hydrogeologic cross section presented in a poster by Anders et al. (2012) depicts a series of sedimentary sequences including shallow Quaternary-aged alluvium underlain by Pliocene- to Eocene-aged formations.	Most (>80%) wells at depths of 140-160 m and at depths exceeding 140 m are defined as tapping a confined aquifer.	-	140-160 m (see (ii) to left)
**Upper Santa Ana Basin**	-	330	348	351	A hydrogeologic cross section presented in Fig. 2-11 by Wildermuth (2005) does not depict a clear continuous confining unit in the Upper Santa	We could not identify a depth where most (>80%) wells with the depth range and at deeper depths are defined as tapping a confined aquifer. The three deepest wells in	-	>360 m (see (ii) to left)

Aquifer system title	Broader aquifer system title	* 60% (m below land surface)	* 70% (m below land surface)	* 80% (m below land surface)	(i) Median depth to top of uppermost confining unit based on cross section**	(ii) Depth below which most wells have been defined by the USGS as tapping a confined aquifer***	(iii) Information from local-scale study pertaining to confined conditions	Estimated depth to confining units for the aquifer system****
					Ana Basin.	our dataset (depths of 330 m, 338 m, 360 m) are classified as unconfined.		
**Utah Lake Valley**	-	276	-	-	A hydrogeologic cross section presented in Fig. 47 by Cederberg et al. (2009) depicts a series of shallow (i.e., near surface) confining units.	Most (>80%) wells at depths of 10-20 m and at depths exceeding 10 m are defined as tapping a confined aquifer.	-	10-20 m (see (ii) to left)

* the depth below which most samples contain minimal (<25%) modern water, where the value defining "most" is reported in the column headings (columns 3, 4 or 5) as one of (i) 60%, (ii) 70%, or (iii) 80%
** determined as the median vertical offset between the land surface and the top of the uppermost confining unit or endogenous bedrock as depicted on the cross section (i.e., the median vertical length (see y-axis for scale) of the 20 transparent pink bars on the cross section)
*** determined on the basis of depth profiles of wells that the USGS has categorized as tapping an unconfined (or confined) aquifer
**** determined on the basis of the combined available from information sources (i), (ii) and (iii) (see columns to left); if a specific information source (i.e., categories (i), (ii) or (iii) in the adjacent columns) was used to generate the estimate the roman numeral specifying that information source is shown here

[x] the uncertainty in our estimated depth to confined conditions for the Eureka and Mad River Plains aquifer system is substantial. The depth to confined conditions was approximated as roughly half the maximum thickness of the Hookton Formation on the basis of descriptions by Johnson, M. J. (1975). Ground-water conditions in the Eureka Area, Humboldt County, California. US Geological Survey Water-Resources Investigations 78-127, 51 pp. Accessed March 20, 2021 from https://pubs.usgs.gov/wri/1978/0127/report.pdf

3.1 Sacramento Basin, California Central Valley

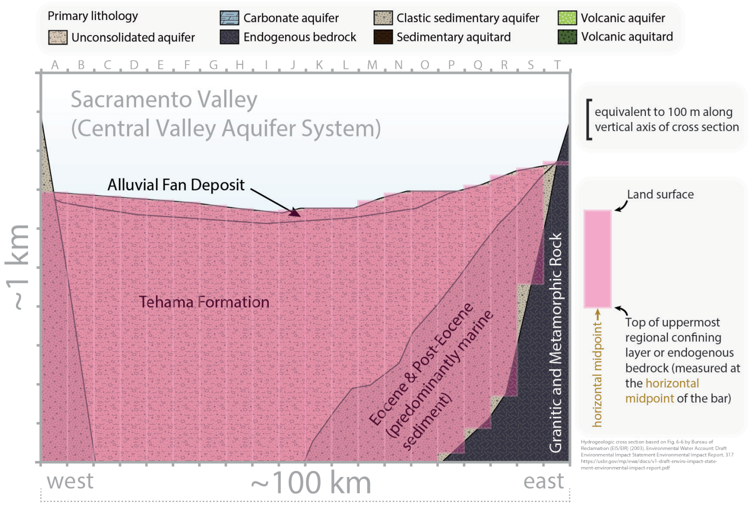

Supplementary Fig. 99. Hydrogeologic cross section. 20 equally spaced transparent pink bars overlie the cross section; each shaded bar depicts the vertical offset from the land surface to the top of the uppermost confining unit or endogenous bedrock.

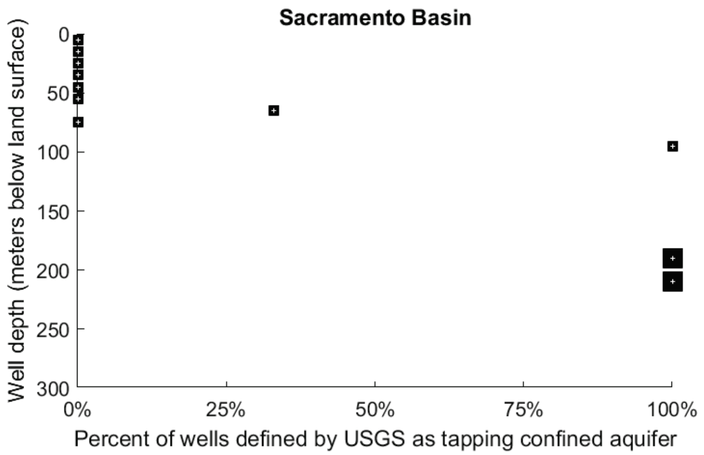

Supplementary Fig. 100. Vertical variations in the prevalence of wells that have been defined as tapping an unconfined or a confined aquifer by the USGS. The smaller squares represent 10 m depth intervals from the land surface to 100 m; the larger squares represent 20 m intervals from 100 m to 300 m below the land surface.

The Sacramento Basin is located in north-central California.

(i) A hydrogeologic cross section presented in Fig. 6-6 by Bureau of Reclamation (2003) does not depict a clear confining unit. The median depth to granitic and metamorphic rock is >663 meters below land surface; however, the aquifer system transitions to confined conditions at shallower depths, despite the lack of a clear confining unit (see (iii)).

(ii) We analysed wells within the study area that the USGS has defined as either unconfined or confined. Most (>80%) wells at depths of 90-100 m or at depths exceeding 90 m are defined as tapping a confined aquifer.

(iii) The Bureau of Reclamation (2003) highlights that deeper depths can be confined (direct quote from report: *"Groundwater is typically unconfined to semi-confined in the shallow aquifer system and confined where deeper aquifers are present"*).

Depth to confined conditions: 90-100 m based on (ii) above

Reference: Bureau of Reclamation (EIS/EIR) (2003). Environmental Water Account: Draft Environmental Impact Statement Environmental Impact Report. 317 https://usbr.gov/mp/ewa/docs/v1-draft-enviro-impact-statement-environmental-impact-report.pdf

The table below presents a series of published quotes (see quotation marks denoting text quoted from another publication, which is cited following the quotation marks with the full reference written in full below the table). The leftmost column lists a title of a hydrogeologic formation depicted in the cross section on the previous page. The rightmost column presents a quote from a hydrogeological study (see base of table for citation). The quote has been annotated with colored text to highlight how we categorized each layer (i.e., see categories in the center column in the table). Specifically: (i) blue text highlights portions of a quote that provide insights into the degree of consolidation of the formation, (ii) red text highlights portions of a quote that categorize the formation as an aquifer or an aquitard (i.e., higher versus lower permeability in the context of local hydrogeologic formations), and (iii) green text highlights portions of a quote that provide information about the lithology of the formation.

Supplementary Table 4. Hydrostratigraphy details for the Sacramento Valley

**Formation name**	**Category**	**Quote**
Alluvial Fan Deposit	Unconsolidated aquifer	"The **alluvial fan** is highly suitable for banking purposes, as it generally consists of **permeable** **river deposits** with high well yields that allow quick recovery." (EWA, 2003)
Tehama Formation	Unconsolidated aquifer	"The Tehama Formation in the western portion of the basin is derived from **Coast Range sediment**. In most of the Sacramento Groundwater Basin, the Tuscan, Mehrten, and Tehama formations are overlain with relatively **thin alluvial deposits**." (EWA, 2003)<br><br>"The groundwater basin west of the Sacramento River is composed of the Tehama Formation, which has exhibited subsidence in Yolo County" (EWA, 2003)<br><br>"Of particular importance to this study is the Plio-Pleistocene Tehama Formation, a productive **aquifer** described in some detail below…" Davisson and Criss (1993)<br><br>"In the subsurface the Tehama deposits generally are water-saturated and **unconsolidated**, except near the base of the section where moderately consolidated gravels occur" Davisson and Criss (1993)<br><br>"The Tehama Formation is a 600-900 m thick fluvial deposit that extends from the Coast Ranges to the axis of the Sacramento Valley, and from Red Bluff in the north to the Montezuma Hills in the eastern Delta region (Olmsted and Davis, 1961). This formation, which has been locally deformed by late Cenozoic uplift and tilting along the western basin margin, consists of detritus derived from the rapidly rising Coast Ranges (Thomasson et al., 1960; Loewen et al., 1992). The deposits are characterized by **yellowish or brownish to blue-green clays interbedded with sands** and gravels." Davisson and Criss (1993)
Eocene and Post-Eocene Continental Deposit	Clastic sedimentary **aquifer** (consolidated or semi-consolidated rock)	"The base of the post-Eocene continental **deposits** is equivalent to the base of the Tehama Formation of Pliocene age which in some places at least may be of late Oligocene and early Miocene age. The post-Eocene deposits contain most of the **fresh ground water** in the valley." Page (1974).<br><br>"The continental sediments consist mostly of **sand and gravel interbedded and mixed with clay and silt deposited by streams and lakes**." Thiros et al. (2010)

Formation name	Category	Quote
Granitic and Metamorphic rocks of the Sierra Nevada	Endogenous bedrock	"**Granitic, volcanic, and metamorphic** rocks that crop out and underlie the eastern part of the valley form an almost **impermeable** boundary for the basin-fill groundwater system." (EWA, 2003)

Page, R. W. (1974). Base and thickness of the post-Eocene continental deposits in the Sacramento Valley, California (No. 45-73). US Geological Survey. https://pubs.usgs.gov/wri/1973/0045/report.pdf

Environmental Water Account ("EWA") (2003). Draft Environmental Impact Statement Environmental Impact Report Volume I Chapters 1-9. https://www.usbr.gov/mp/ewa/docs/v1-draft-enviro-impact-statement-environmental-impact-report.pdf

Thiros, S. A., Bexfield, L. M., Anning, D. W., & Huntington, J. M. (2010). Conceptual understanding and groundwater quality of selected basin-fill aquifers in the southwestern United States (No. 1781). US Geological Survey. https://pubs.usgs.gov/pp/1781/

Davisson, M. L., & Criss, R. E. (1993). Stable isotope imaging of a dynamic groundwater system in the southwestern Sacramento Valley, California, USA. Journal of Hydrology, 144, 213-246.

3.2 San Joaquin Basin, California Central Valley

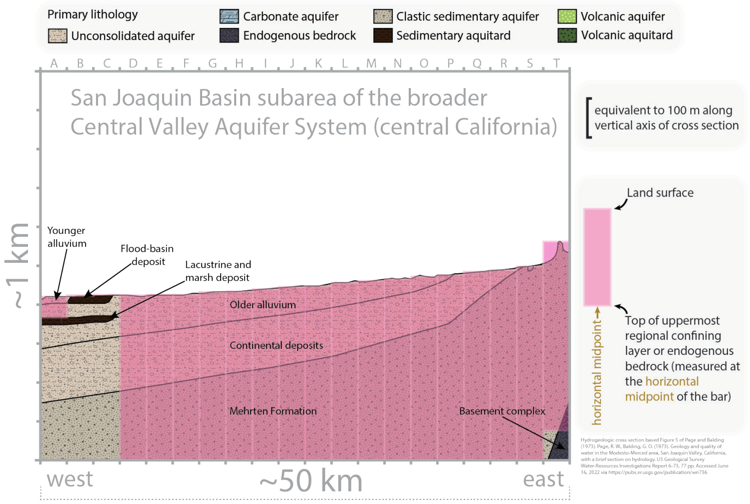

Supplementary Fig. 101. Hydrogeologic cross section. 20 equally spaced transparent pink bars overlie the cross section; each shaded bar depicts the vertical offset from the land surface to the top of the uppermost confining unit or endogenous bedrock.

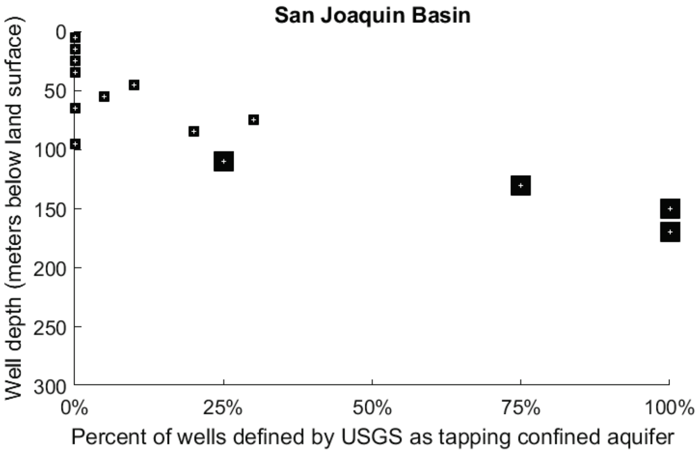

Supplementary Fig. 102. Vertical variations in the prevalence of wells that have been defined as tapping an unconfined or a confined aquifer by the USGS. The smaller squares represent 10 m depth intervals from the land surface to 100 m; the larger squares represent 20 m intervals from 100 m to 300 m below the land surface.

The San Joaquin Basin is located in central California.

(i) A hydrogeologic cross section presented in Fig. 5 by Page and Balding (1973) does not depict a clear confining unit within the aquifer system.

(ii) We analysed wells within the study area that the USGS has defined as either unconfined or confined. Most (>80%) wells at depths of 140-160 m and at depths exceeding 140 m are defined as tapping a confined aquifer.

(iii) Page and Balding (1973) state (quote directly): *"The confined water body occurs in the unconsolidated deposits that underlie the E-clay (fig. 6). The base of the confined water body probably is at the top of the Mehrten Formation, but in terms of use its base is considered to be the base of fresh water..."*

Depth to confined conditions: 140-160 m based on (ii) above

Reference: Page, R. W., Balding, G. O. (1973). Geology and quality of water in the Modesto-Merced area, San Joaquin Valley, California, with a brief section on hydrology. US Geological Survey Water-Resources Investigations Report 73-6, 77 pp. Accessed April 11, 2022 via https://pubs.usgs.gov/wri/1973/0006/report.pdf

The table below presents a series of published quotes (see quotation marks denoting text quoted from another publication, which is cited following the quotation marks with the full reference written in full below the table). The leftmost column lists a title of a hydrogeologic formation depicted in the cross section on the previous page. The rightmost column presents a quote from a hydrogeological study (see base of table for citation). The quote has been annotated with colored text to highlight how we categorized each layer (i.e., see categories in the center column in the table). Specifically: (i) blue text highlights portions of a quote that provide insights into the degree of consolidation of the formation, (ii) red text highlights portions of a quote that categorize the formation as an aquifer or an aquitard (i.e., higher versus lower permeability in the context of local hydrogeologic formations), and (iii) green text highlights portions of a quote that provide information about the lithology of the formation.

Supplementary Table 5. Hydrostratigraphy details for the San Joaquin Basin

Formation name	Category	Quote
Qb – Flood-basin deposits *(unconsolidated aquitard due to high impermeable clay layer)*	Sedimentary **aquitard** (consolidated or semi-consolidated rock)	"They consist **of intercalated lenses of bluish-gray, brown, and reddish-brown fine sand, silt, and clay. In the subsurface, the deposits are interbedded with the younger alluvium and probably in part with the older alluvium**." (Page and Balding, 1973). Figure 5 in Page and Balding, 1973, the explanation shows that "**Flood-basin deposits**" under "**Unconsolidated rocks**". "As indicated on drillers' logs, they range in thickness from 0 to about 100 feet. Because of **their impermeable clayey nature**, the flood-basin deposits **would yield only very small quantities of water to wells**" (Page and Balding, 1973).
Qya – Younger alluvium	Unconsolidated aquifer	"It consists mostly of **fine sand, sand, and gravel with little or no hardpan**." (Page and Balding, 1973). Figure 5 in Page and Balding, 1973, the explanation shows that "**Younger alluvium**" under "**Unconsolidated rocks**". "Because in most places the younger alluvium is not completely saturated, **it probably will yield only moderate quantities of water to wells**." (Page and Balding, 1973).
Q1 – lacustrine and marsh deposits (E-clay)	Sedimentary **aquitard** (consolidated or semi-consolidated rock)	"The **lacustrine and marsh deposits (E-clay)** and the flood-basin deposits **yield little water to wells**" (Page and Balding, 1973). Table 1 in Page and Balding, 1973, Lithologic characteristic: "**Silt, silty clay, and clay, gray and blue**" and Water-bearing character: "**Confining bed, very small hydraulic conductivities**."
Qca – Older alluvium	Unconsolidated aquifer	"It consists of **intercalated beds** of **gravel, sand, silt, and clay, with some hardpan**." (Page and Balding, 1973). Figure 5 in Page and Balding, 1973, the explanation shows that "**Older alluvium**" under "**Unconsolidated rocks**". "The **older alluvium** is the most extensively developed a**quifer in the Modesto-Merced area, yielding water to large numbers of domestic, irrigation, industrial, and public-supply wells**." (Page and Balding, 1973).
QTc – Continental deposits	Unconsolidated aquifer	"They consist of a gently southwestward-dipping **alluvium of poorly sorted gravel, sand, silt, and clay**. And they are generally finer grained than the overlying older alluvium (fig. 12)." (Page and Balding, 1973). Figure 5 in Page and Balding, 1973, the explanation shows that "**Continental deposits**" under "**Unconsolidated rocks**". "**Yields to wells are as large as 281 cfm (2,100 gpm) and specific capacities as large as 3 ft2/min (22 gpm per foot)**." (Page and Badling, 1973).

Formation name	Category	Quote
Tm – Mehrten Formation	Sedimentary **aquifer** (consolidated or semi-consolidated rock)	"The Mehrten Formation, of Miocene and Pliocene age, crops out near the eastern edge of the area (fig. 5). **It consists of fluviatile deposits of sandstone, breccia, conglomerate, tuff, siltstone, and claystone** (Davis and Hall, 1959, p. 9-10; Piper and others, 1939, p. 61-67)." (page and Balding, 1973). "The **Mehrten is one of the important aquifers** in the Modesto-Merced area, and water wells in the eastern part of the area commonly penetrate it (fig. 6)." (Page and Balding, 1973). Figure 5 in page and Balding, 1973, the explanation shows that "**Mehrten Formation**" under "**Consolidated rocks**".
pTb – Basement complex	Endogenous bedrock	"The exposed **basement complex** consists largely of **metasedimentary and 134etavolcanics rocks of pre-Tertiary age** (Bateman and others, 1963, pl. 1)." (Page and Badling, 1973). "Where the **basement complex** occurs at or near the surface, **only small quantities of water are yielded to wells through narrow joints and fractures**." (Page and Balding, 1973).

Page, R. W., Balding, G. O. (1973). Geology and quality of water in the Modesto-Merced area, San Joaquin Valley, California, with a brief section on hydrology. (No.6-73). US Geological Survey Water-Resources Investigations Report 73-6, 77 pp. Accessed June 16, 2022 via https://pubs.er.usgs.gov/publication/wri736

3.3 Tulare Basin, California Central Valley

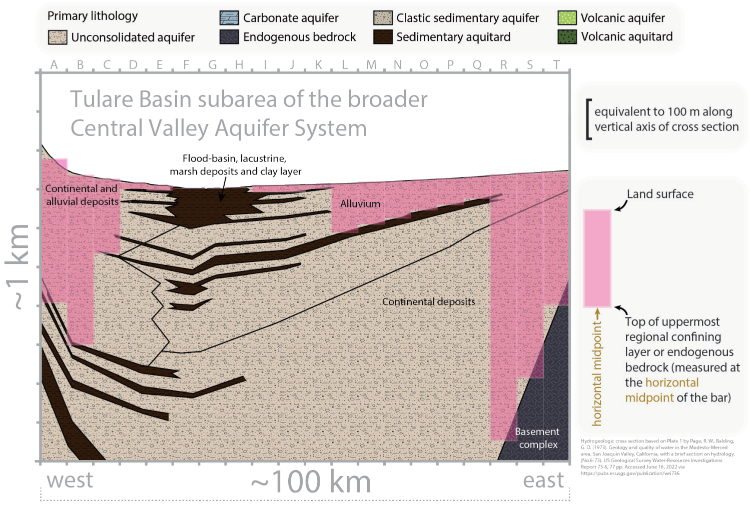

Supplementary Fig. 103. Hydrogeologic cross section. 20 equally spaced transparent pink bars overlie the cross section; each shaded bar depicts the vertical offset from the land surface to the top of the uppermost confining unit or endogenous bedrock.

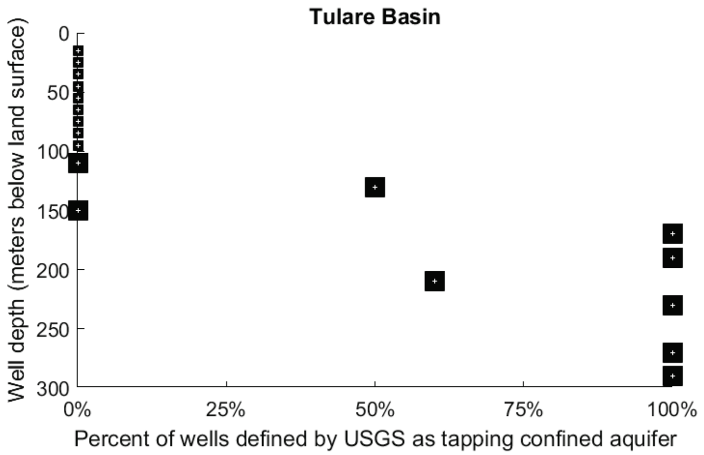

Supplementary Fig. 104. Vertical variations in the prevalence of wells that have been defined as tapping an unconfined or a confined aquifer by the USGS. The smaller squares represent 10 m depth intervals from the land surface to 100 m; the larger squares represent 20 m intervals from 100 m to 300 m below the land surface.

The Tulare Basin is located in south-central California.

(i) A hydrogeologic cross section presented in Plate 1 by Croft (1999) suggests that the aquifer system includes a series of low permeability units, including the "E-clay" (primarily the Corcoran clay unit).

(ii) We analysed wells within the study area that the USGS has defined as either unconfined or confined. Most (>80%) wells at depths of 160-180 m and wells with depths exceeding 160 m are defined as tapping a confined aquifer.

(iii) Croft (1999) state (quote) *"Although the E clay is the principal confining bed in the valley (Davis and other, 1959, p. 87-90; Davis and Poland, 1957, p. 426), water-level data indicate that the A clay also is an effective confining bed throughout most of its extent."*

Depth to confined conditions: 160-180 m based on (ii) above

Reference: Croft, M.G. (1999). Subsurface geology of the late Tertiary and Quaternary water-bearing deposits of the southern part of the San Joaquin Valley, California. U.S. Geological Survey Water-Supply Paper 1999-H, 35 pp. Accessed April 10, 2022 via https://pubs.usgs.gov/wsp/1999h/report.pdf

The table below presents a series of published quotes (see quotation marks denoting text quoted from another publication, which is cited following the quotation marks with the full reference written in full below the table). The leftmost column lists a title of a hydrogeologic formation depicted in the cross section on the previous page. The rightmost column presents a quote from a hydrogeological study (see base of table for citation). The quote has been annotated with colored text to highlight how we categorized each layer (i.e., see categories in the center column in the table). Specifically: (i) blue text highlights portions of a quote that provide insights into the degree of consolidation of the formation, (ii) red text highlights portions of a quote that categorize the formation as an aquifer or an aquitard (i.e., higher versus lower permeability in the context of local hydrogeologic formations), and (iii) green text highlights portions of a quote that provide information about the lithology of the formation.

Supplementary Table 6. Hydrostratigraphy details for the Tulare Basin

**Formation name**	**Category**	**Quote**
Flood-basin, lacustrine, marsh deposits and clay layer	Sedimentary **aquitard** (consolidated or semi-consolidated rock)	"They consist **of intercalated lenses of bluish-gray, brown, and reddish-brown fine sand, silt, and clay. In the subsurface, the deposits are interbedded with the younger alluvium and probably in part with the older alluvium**." (Page and Balding, 1973). Figure 5 in Page and Balding, 1973, the explanation shows that "**Flood-basin deposits**" under "**Unconsolidated rocks**". "As indicated on drillers' logs, they range in thickness from 0 to about 100 feet. Because of **their impermeable clayey nature**, the flood-basin deposits **would yield only very small quantities of water to wells**" (Page and Balding, 1973).
Alluvium	Unconsolidated aquifer	"The reduced deposits of the **alluvium** consist of **moderately permeable bluish-green fine to coarse sand, silt, and clay**." (Croft, 1999).
Continental and alluvial deposits	Unconsolidated aquifer	"**Continental and alluvial deposits** of Tertiary and Quaternary age that were derived from the Coast Ranges consist mainly of **poorly to moderately permeable yellowish-brown gravel, sand, silt, and clay**." (Croft, 1999).
Continental deposits	Unconsolidated aquifer	"They consist of a gently southwestward-dipping **alluvium of poorly sorted gravel, sand, silt, and clay**. And they are generally finer grained than the overlying older alluvium (fig. 12)." (Page and Balding, 1973). Figure 5 in Page and Balding, 1973, the explanation shows that "**Continental deposits**" under "**Unconsolidated rocks**". "**Yields to wells are as large as 281 cfm (2,100 gpm) and specific capacities as large as 3 ft^2/min (22 gpm per foot)**." (Page and Badling, 1973).
Basement complex	Endogenous bedrock	"The basement complex forms much of the southern Sierra Nevada, Tehachapi, and San Emigdio Mountains and **is composed of a mass of plutonic and metamorphic rocks commonly referred to as the Sierra Nevada batholith of pre-Tertiary age**." Croft, M.G. (1999). "**The rocks of the basement complex** are of **little importance as a source of ground water because they are largely impermeable and**

Formation name	Category	Quote
		**generally lie beneath most water wells, except locally along the eastern margin of the valley**." (Croft, 1999).

Page, R. W., Balding, G. O. (1973). Geology and quality of water in the Modesto-Merced area, San Joaquin Valley, California, with a brief section on hydrology. (No.6-73). US Geological Survey Water-Resources Investigations Report 73-6, 77 pp. https://pubs.er.usgs.gov/publication/wri736

Croft, M.G. (1999). Subsurface geology of the late Tertiary and Quaternary water-bearing deposits of the southern part of the San Joaquin Valley, California. U.S. Geological Survey Water-Supply Paper 1999-H, 35 pp. Accessed June 17, 2022 via https://pubs.usgs.gov/wsp/1999h/report.pdf

3.4 Eastern Carrizo-Wilcox, Carrizo-Wilcox

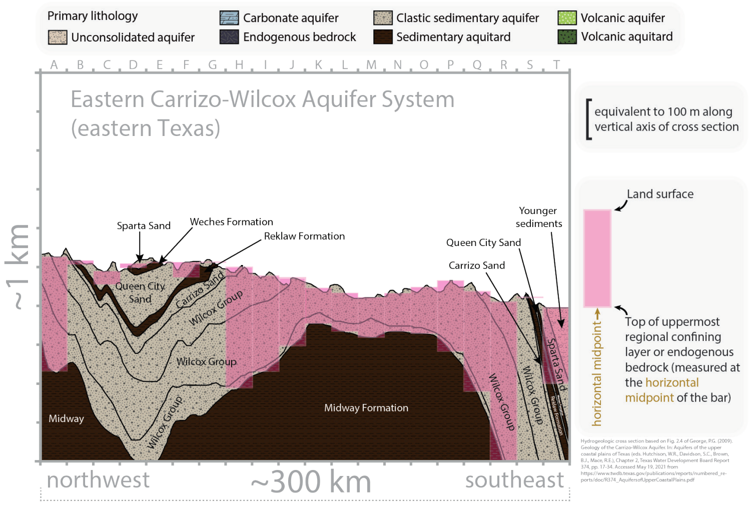

Supplementary Fig. 105. Hydrogeologic cross section. 20 equally spaced transparent pink bars overlie the cross section; each shaded bar depicts the vertical offset from the land surface to the top of the uppermost confining unit or endogenous bedrock.

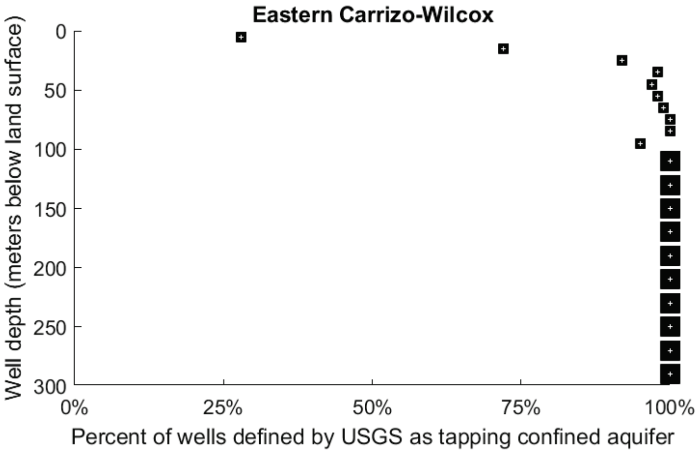

Supplementary Fig. 106. Vertical variations in the prevalence of wells that have been defined as tapping an unconfined or a confined aquifer by the USGS. The smaller squares represent 10 m depth intervals from the land surface to 100 m; the larger squares represent 20 m intervals from 100 m to 300 m below the land surface.

The Eastern Carrizo-Wilcox aquifer system is located in eastern Texas and northwestern Louisiana.

(i) A hydrogeologic cross section presented in Fig. 2.4 by George (2009) depicts a series of confining units including the relatively shallow Reklaw Formation and the Weches Formation.

(ii) We analysed wells within the study area that the USGS has defined as either unconfined or confined. Most (>80%) wells at depths of 20-30 m and at depths exceeding 20 m are defined as tapping a confined aquifer.

Depth to confined conditions: 20-30 m based on (ii) above

Reference: George, P.G. (2009). Geology of the Carrizo-Wilcox Aquifer. In: Aquifers of the upper coastal plains of Texas (eds. Hutchison, W.R., Davidson, S.C., Brown, B.J., Mace, R.E.), Chapter 2, Texas Water Development Board Report 374, pp. 17-34. Accessed May 19, 2021 from https://www.twdb.texas.gov/publications/reports/numbered_reports/doc/R374_AquifersofUpperCoastalPlains.pdf

The table below presents a series of published quotes (see quotation marks denoting text quoted from another publication, which is cited following the quotation marks with the full reference written in full below the table). The leftmost column lists a title of a hydrogeologic formation depicted in the cross section on the previous page. The rightmost column presents a quote from a hydrogeological study (see base of table for citation). The quote has been annotated with colored text to highlight how we categorized each layer (i.e., see categories in the center column in the table). Specifically: (i) blue text highlights portions of a quote that provide insights into the degree of consolidation of the formation, (ii) red text highlights portions of a quote that categorize the formation as an aquifer or an aquitard (i.e., higher versus lower permeability in the context of local hydrogeologic formations), and (iii) green text highlights portions of a quote that provide information about the lithology of the formation.

Supplementary Table 7. Hydrostratigraphy details for the Eastern Carrizo-Wilcox

Formation name	Category	Quote
Younger Formation	Unconsolidated aquifer	"**Sand, silt, clay, and some gravel.**" (Sandeen 1987) (Table 6). "**May yield small quantities of water to shallow dug wells.**" (Sandeen 1987). "**Sand, silt, and clay**" (Sandeen, 1987)
Sparta Sand	Sedimentary **aquifer** (consolidated or semi-consolidated rock)	"**Interbedded sand, clay, and silt**." (Sandeen 1987) (Table 6). "**Feeds springs; may yield some water to dug wells.**" (Sandeen, 1987).
Weches Formation	Sedimentary **aquitard** (consolidated or semi-consolidated rock)	"**Glauconite, glauconitic clay and sand**. **Secondary deposits of limestone in outcrop**." (Sandeen 1987) (Table 6). **Not known to yield water to wells** in Rusk County. (Sandeen, 1987)
Queen City Sand	Sedimentary **aquifer** (consolidated or semi-consolidated rock)	"**Sand, silt, clay, and some lignite**. (Sandeen 1987) (Table 6). "**Yields small to moderate quantities of freshwater**. (Sandeen 1987) "The Queen City Formation is composed of **sand, sandstone, shale, and clay, with lignite** found locally. **The aquifer thickness is less than 500 feet in most places but reaches almost 700 feet in parts** of northeast Texas." (Davidson et al. 2009)
Reklaw Formation	Sedimentary **aquitard** (consolidated or semi-consolidated rock)	"**Glauconitic clay, some sand, weathers to a red clayey soil, limonite seams, iron concretions.**" (Sandeen 1987) (Table 6). "**Yields small quantities of water to wells.**" (Sandeen 1987) "The Carrizo-Wilcox aquifer is separated from the overlying Queen City aquifer by the **Reklaw or Bigford Fm., which is a confining unit** (Fig. 2)." (Huang et al. 2012). "It is separated from the overlying Queen City Aquifer by the relatively **impermeable** Reklaw Formation." (George, 2009).
Carrizo Sand	Sedimentary **aquifer** (consolidated or semi-consolidated rock)	"Grey to white. Often **massive sand, clay lenses**; may be predominantly clayey". (Sandeen 1987) (Table 6). "**Yields large to moderate quantities of freshwater**. In hydrologic continuity with the Wilcox." (Sandeen 1987)
Wilcox Group	Sedimentary **aquifer** (consolidated or semi-consolidated rock)	"Thin, sometimes **massive** beds of **sand; clay and lignite**. Beds often dis- continuous." (Sandeen 1987) (Table 6).

Formation name	Category	Quote
Midway	Sedimentary **aquitard** (consolidated or semi-consolidated rock)	"**Calcareous clay** and minor amounts of **limestone, silt, and glauconitic clay**." (Sandeen 1987) (Table 6). "**Not known to yield water to wells** in Rusk County; upper sand may contain some slightly saline water." (Sandeen 1987)

Sandeen, W. M. (1984). Ground-water resources of Rusk County, Texas. US Geological Survey. (https://pubs.usgs.gov/of/1983/0757/report.pdf)

Huang, Y., Scanlon, B. R., Nicot, J. P., Reedy, R. C., Dutton, A. R., Kelley, V. A., Deeds, N. E. (2012). Sources of groundwater pumpage in a layered aquifer system in the Upper Gulf Coastal Plain, USA. Hydrogeology Journal, 20(4), 783-796.

George, P.G. (2009). Geology of the Carrizo-Wilcox Aquifer. In: Aquifers of the upper coastal plains of Texas (eds. Hutchison, W.R., Davidson, S.C., Brown, B.J., Mace, R.E.), Chapter 2, Texas Water Development Board Report 374, pp. 17-34. Accessed June 14, 2022 from https://www.twdb.texas.gov/publications/reports/numbered_reports/doc/R374_AquifersofUpperCoastalPlains.pdf

Davidson, S.C., Brown, B.J., Mace, R.E. (2009). Geology of the Carrizo-Wilcox Aquifer. In: Aquifers of the upper coastal plains of Texas (eds. Hutchison, W.R., Davidson, S.C., Brown, B.J., Mace, R.E.), Chapter 1, Texas Water Development Board Report 374, pp. 1-16. Accessed June 14, 2022 from https://www.twdb.texas.gov/publications/reports/numbered_reports/doc/R374_AquifersofUpperCoastalPlains.pdf

3.5 Eagle Valley, Carson River Basin

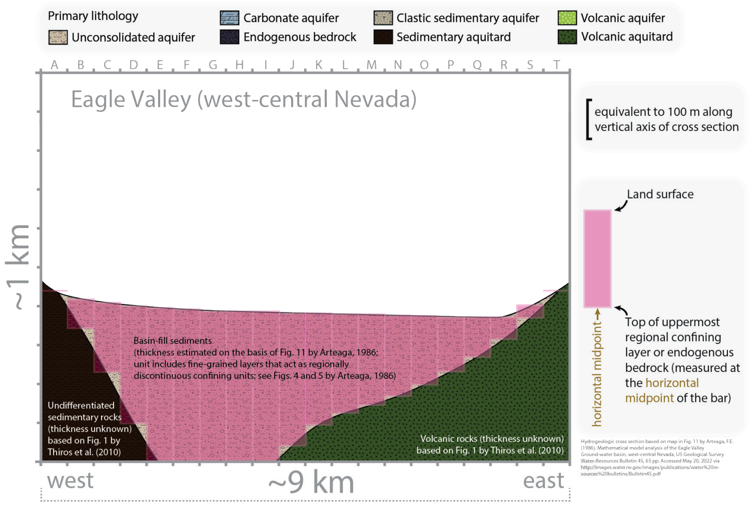

Supplementary Fig. 107. Hydrogeologic cross section. 20 equally spaced transparent pink bars overlie the cross section; each shaded bar depicts the vertical offset from the land surface to the top of the uppermost confining unit or endogenous bedrock.

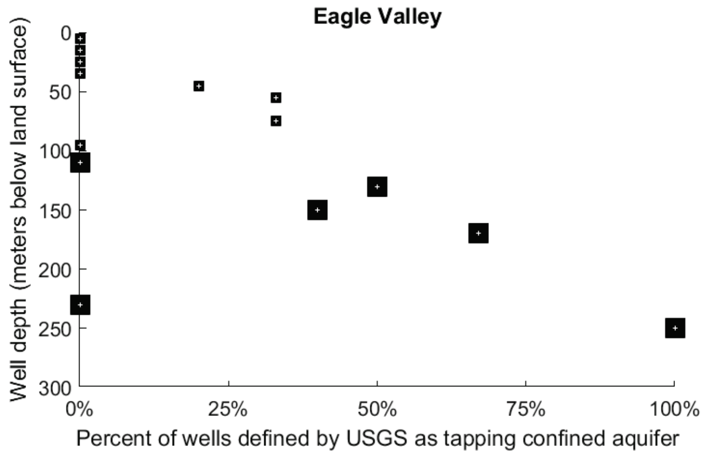

Supplementary Fig. 108. Vertical variations in the prevalence of wells that have been defined as tapping an unconfined or a confined aquifer by the USGS. The smaller squares represent 10 m depth intervals from the land surface to 100 m; the larger squares represent 20 m intervals from 100 m to 300 m below the land surface.

Eagle Valley is located in western Nevada.

(i) To create a hydrogeologic cross section, we georeferenced Fig. 11 (pp. 25) by Arteaga (1986) that displays the (quote) *"(a)pproximate thickness of valley-fill sedimentary deposits".*

(ii) Only half of the deepest wells that the USGS has defined as confined/unconfined (depths exceeding 200 m) are defined as tapping a confined aquifer; the deepest well in the dataset (381 m) is defined as tapping an unconfined aquifer.

(iii) Thiros et al. (2010; see section 4 by J.M. Huntington) state (quote): *"Unconfined to confined conditions are present in the basin-fill sediments…The degree of confinement varies spatially through the valley due to the clay lenses being discontinuous at different depths."* and *"Although groundwater exists under both confined and unconfined conditions in Carson Valley, no single confining layer extends across the entire valley… the confining layers occur mainly as scattered, discontinuous clay beds, 30 to 70 ft thick, at a depth of 200 to 300 ft."*

Depth to confined conditions: >200 m based on (ii)

References: Arteaga, F.E. (1986). Mathematical model analysis of the Eagle Valley Ground-water basin, west-central Nevada, US Geological Survey Water-Resources Bulletin 45, 65 pp. Accessed May 20, 2022 via http://images.water.nv.gov/images/publications/water%20resources%20bulletins/Bulletin45.pdf

Thiros, S.A., Bexfield, L.M., Anning, D.W., and Huntington, J.M., eds., (2010). Conceptual understanding and groundwater quality of selected basin-fill aquifers in the Southwestern United States: US Geological Professional Paper 1781, 288 pp. Accessed May 19, 2022 via https://pubs.usgs.gov/pp/1781/

The table below presents a series of published quotes (see quotation marks denoting text quoted from another publication, which is cited following the quotation marks with the full reference written in full below the table). The leftmost column lists a title of a hydrogeologic formation depicted in the cross section on the previous page. The rightmost column presents a quote from a hydrogeological study (see base of table for citation). The quote has been annotated with colored text to highlight how we categorized each layer (i.e., see categories in the center column in the table). Specifically: (i) blue text highlights portions of a quote that provide insights into the degree of consolidation of the formation, (ii) red text highlights portions of a quote that categorize the formation as an aquifer or an aquitard (i.e., higher versus lower permeability in the context of local hydrogeologic formations), and (iii) green text highlights portions of a quote that provide information about the lithology of the formation.

Supplementary Table 8. Hydrostratigraphy details for the Eagle Valley

Formation name	Category	Quote
Basin-fill sediments	Unconsolidated aquifer	"Quaternary sediments of two ages are present in Eagle Valley. The older sediments form fans at the mouths of deeply incised canyons on the western side of the valley. Small individual fans merge into one wide fan extending as much as 1 mi eastward into the valley from the mountain front and are made up of **partly consolidated to unconsolidated gravel, sand, and silt, with discontinuous clay layers** (Maurer and others, 1996). Similar fans are present at the base of the Virginia Range to the north and Prison Hill to the east (Trexler and others, 1980). The discontinuity of clay layers in the central part of the basin enable a direct hydraulic connection from the land surface to **the basin-fill aquifer** and make the aquifer susceptible to contamination from sources at the surface (Lico, 1998, p. 1). The younger sediments in the valley lowlands consist of fine-grained sands, silty and muddy sands, and clay (Arteaga, 1986; Trexler and others, 1980). Overall, basin-fill sediments are coarse-grained near the base of the mountains and finer grained near the center of the valley. The basin-fill sediments are estimated to be about 1,200 ft thick at a point 1.5 mi west of Lone Mountain, about 400 to 800 ft thick beneath the northeastern and southern parts of Eagle Valley, and about 2,000 ft thick about 1 mi northwest of Prison Hill (Arteaga, 1986). In general, the deepest part of the alluvial basin is in the center of the Eagle Valley (Schaefer and others, 2007)." quoting Thiros et al. (2010)
Volcanic rocks	Volcanic rocks (see Fig. 1 by Thiros et al. (2010) on page 50)	"Mesozoic-age granite and metamorphosed rocks crop out to the north and west of Eagle Valley and near Prison Hill, and most likely underlie most of the valley floor (Moore, 1969). In the Virginia Range, Tertiary sandstone and volcanic rocks consisting mostly of rhyolite, andesite, and basalt flows, flow breccias, and tuffs overlie the granite and metamorphosed rocks (Moore, 1969; Trexler, 1977)." quoting Thiros et al. (2010)
Undifferentiated sedimentary rocks	Sedimentary **aquitard** (consolidated or semi-consolidated rock)	Based on Fig. 1 by Thiros et al. (2010)

Thiros, S.A., Bexfield, L.M., Anning, D.W., and Huntington, J.M., eds., (2010). Conceptual understanding and groundwater quality of selected basin-fill aquifers in the Southwestern United States: US Geological Professional Paper 1781, 288 pp. Accessed May 19, 2022 via https://pubs.usgs.gov/pp/1781/

3.6 Central Wabash and Bloomington Ridged Plain, Central Lowland Till Plain

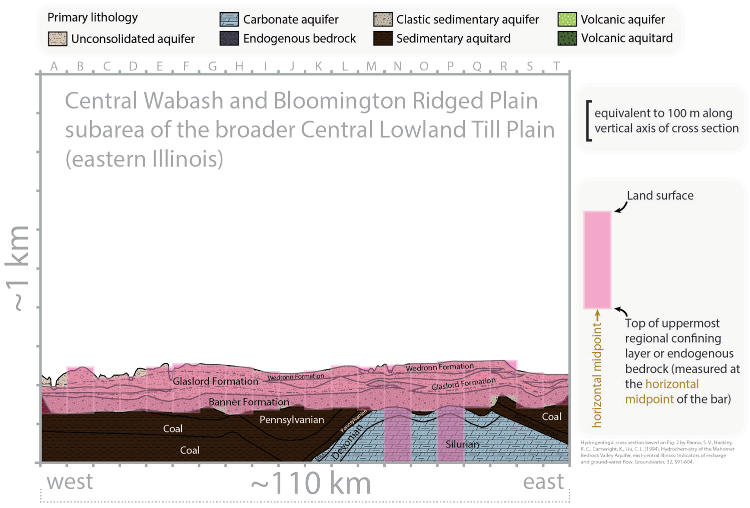

Supplementary Fig. 109. Hydrogeologic cross section. 20 equally spaced transparent pink bars overlie the cross section; each shaded bar depicts the vertical offset from the land surface to the top of the uppermost confining unit or endogenous bedrock.

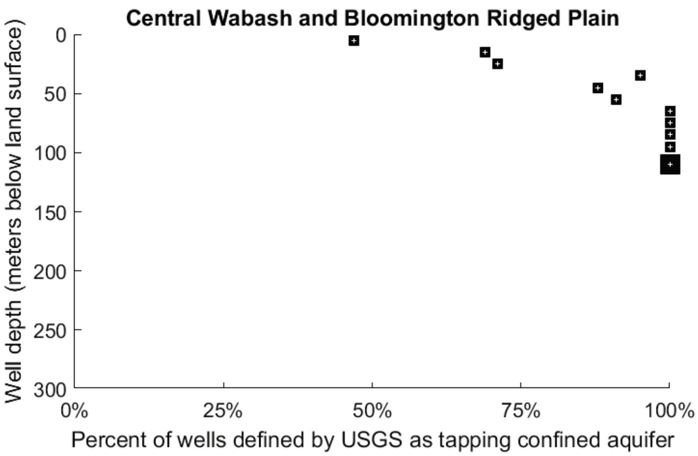

Supplementary Fig. 110. Vertical variations in the prevalence of wells that have been defined as tapping an unconfined or a confined aquifer by the USGS. The smaller squares represent 10 m depth intervals from the land surface to 100 m; the larger squares represent 20 m intervals from 100 m to 300 m below the land surface.

The Central Wabash and Bloomington Ridged Plain is located in eastern Illinois.

(i) A hydrogeologic cross section presented in Fig. 2 by Panno et al. (1994) depicts sedimentary aquifers at shallow depths (including the Mahomet Sand member of the Banner Formation) overlying low permeability layers. In some areas carbonate rocks directly underlie the shallow sedimentary aquifers.

(ii) We analysed wells within the study area that the USGS has defined as either unconfined or confined. Most (>80%) wells at depths of 30-40 m and at depths exceeding 30 m are defined as tapping a confined aquifer.

Depth to confined conditions: 30-40 m (based on (ii) above)

Reference: Panno, S. V., Hackley, K. C., Cartwright, K., Liu, C. L. (1994). Hydrochemistry of the Mahomet Bedrock Valley Aquifer, east-central Illinois: Indicators of recharge and ground-water flow. Groundwater, 32, 591-604.

The table below presents a series of published quotes (see quotation marks denoting text quoted from another publication, which is cited following the quotation marks with the full reference written in full below the table). The leftmost column lists a title of a hydrogeologic formation depicted in the cross section on the previous page. The rightmost column presents a quote from a hydrogeological study (see base of table for citation). The quote has been annotated with colored text to highlight how we categorized each layer (i.e., see categories in the center column in the table). Specifically: (i) blue text highlights portions of a quote that provide insights into the degree of consolidation of the formation, (ii) red text highlights portions of a quote that categorize the formation as an aquifer or an aquitard (i.e., higher versus lower permeability in the context of local hydrogeologic formations), and (iii) green text highlights portions of a quote that provide information about the lithology of the formation.

Supplementary Table 9. Hydrostratigraphy details for the Central Wabash Bloomington Ridge Plain

Formation name	Category	Quote
Wedron Formation	Unconsolidated aquifer	"**Water-bearing sand and gravel deposits** contained within the Wedron Formation occur only as scattered pockets, as the formation consists principally of **glacial till**." Anliker & Sanderson, 1995). "The **chemistry of the ground waters** from the **sands of the Wedron**, Glasford, and Banner Formations is very similar, suggesting that the aquifers of these formations are in hydraulic communication (Figure 2)." (Panno et al., 1994).
Glasford Formation	Unconsolidated aquifer	"The MVA is generally coincident with the Mahomet Sand Member of the Banner Formation, and is overlain by the Glasford and the Wedron Formations (Figure 2). **Sand and gravel outwash deposits** within the overlying **Glasford Formation** are usually most extensive where they are associated with the Vandalia Till Member and constitute a second **productive aquifer** that is locally important." (Panno et al., 1994).
Banner Formation	Sedimentary rock **aquifer** (consolidated or semi-consolidated rock)	"The **chemistry of the ground waters** from the sands of the Wedron, Glasford, and **Banner Formations** is very similar, suggesting that the aquifers of these formations are in hydraulic communication (Figure 2)." (Panno et al., 1994). "The **Mahomet Sand Member** is composed of **glacial outwash sand and gravel laid down** within the confines of the Mahomet Valley Lowland." (Panno et al., 1994).
Pennsylvanian	Sedimentary rock **aquitard** (consolidated or semi-consolidated rock)	"The **bedrock geology** of the study area consists of Pennsylvanian, **Mississippian**, Devonian, and Silurian rocks. The western half of the valley is dominated by Pennsylvanian rocks (typically **shale with thin limestone, sandstone, and coal**). The eastern part of the valley is underlain by **rocks of** the Pennsylvanian, **Mississippian**, and Devonian." (Panno et al., 1994).
Coal	Sedimentary rock **aquitard** (consolidated or semi-consolidated rock)	"The **bedrock geology** of the study area consists of Pennsylvanian, Mississippian, Devonian, and Silurian rocks. The western half of the valley is dominated by Pennsylvanian rocks (typically shale with thin limestone, sandstone, and **coal**). The eastern part of the valley is underlain by rocks of the Pennsylvanian, Mississippian, and Devonian." (Panno et al., 1994). "(Figure 2) consists of a subcrop of **Pennsylvanian coals and associated black shale**. Coals in this area are thin and typically contain several percent S" (Panno et al., 1994).

Formation name	Category	Quote
Mississippian	Carbonate aquifer	"The **bedrock geology** of the study area consists of Pennsylvanian, **Mississippian**, Devonian, and Silurian rocks. The western half of the valley is dominated by Pennsylvanian rocks (typically shale with thin limestone, sandstone, and coal). The eastern part of the valley is underlain by rocks of the Pennsylvanian, Mississippian, and Devonian." (Panno et al., 1994). "**Shale, siltstone, limestone and dolomite; upper part is soft, fissile, and fractured; locally contains siderite nodules, plant macrofossils, and slickenslides**" (Stumpf, 2018).
Devonian	Carbonate aquifer	"The **bedrock geology** of the study area consists of Pennsylvanian, Mississippian, **Devonian**, and Silurian rocks. The western half of the valley is dominated by Pennsylvanian rocks (typically shale with thin limestone, sandstone, and coal). The eastern part of the valley is underlain by **rocks of** the Pennsylvanian, Mississippian, and **Devonian**." (Panno et al., 1994). "The Onarga Valley, a northeastern tributary of the **MVA**, is dominated by **bedrock of Middle Devonian** and Silurian age strata that consists of **dolomite and the Pennsylvanian Caseyville, Abbott, and Spoon Formations that contain thin coals, black shale, and thin limestone** (Willman et al., 1967)." (Panno et al., 1994). "**Mahomet Bedrock Valley Aquifer (MVA)"** (Panno et al., 1994).
Silurian	Carbonate aquifer	"The **bedrock geology** of the study area consists of Pennsylvanian, Mississippian, Devonian, and **Silurian** rocks. The western half of the valley is dominated by Pennsylvanian rocks (typically shale with thin limestone, sandstone, and coal). The eastern part of the valley is underlain by rocks of the Pennsylvanian, Mississippian, and Devonian." (Panno et al., 1994). "**Ground water from most wells** in the **Silurian dolomite** in Iroquois Co. contain relatively low concentrations of $SO4^{-2}$." (Panno et al., 1994).

Panno, S. V., Hackley, K. C., Cartwright, K., & Liu, C. L. (1994). Hydrochemistry of the Mahomet Bedrock Valley Aquifer, east-central Illinois: Indicators of recharge and ground-water flow. Groundwater, 32(4), 591-604.

Anliker, M. A., Sanderson, E. W. (1995). Reconnaissance study of ground-water levels and withdrawls in the vicinity of DeWitt and Piatt counties. *ISWS Contract Report CR 589*. Accessed July 10, 2022 via http://citeseerx.ist.psu.edu/viewdoc/download?doi=10.1.1.542.7107&rep=rep1&type=pdf

Stumpf, A.J. (2018). Geologic cross sections of Quaternary deposits across the Manlove gas storage field area, Champaign County, Illinois: Illinois State Geological Survey, Special Report 6, 7 p.; 2 plates. Accessed July 10, 2022 via https://www2.illinois.gov/epa/Documents/iepa/community-relations/mahomet-aquifer/task-force/Special-Report-6.pdf

3.7 Palouse Slope, Columbia Plateau Regional Aquifer System

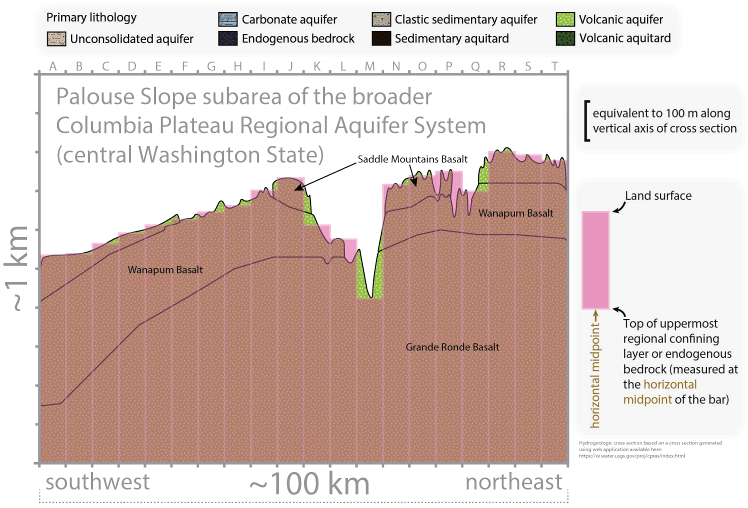

Supplementary Fig. 111. Hydrogeologic cross section. 20 equally spaced transparent pink bars overlie the cross section; each shaded bar depicts the vertical offset from the land surface to the top of the uppermost confining unit or endogenous bedrock.

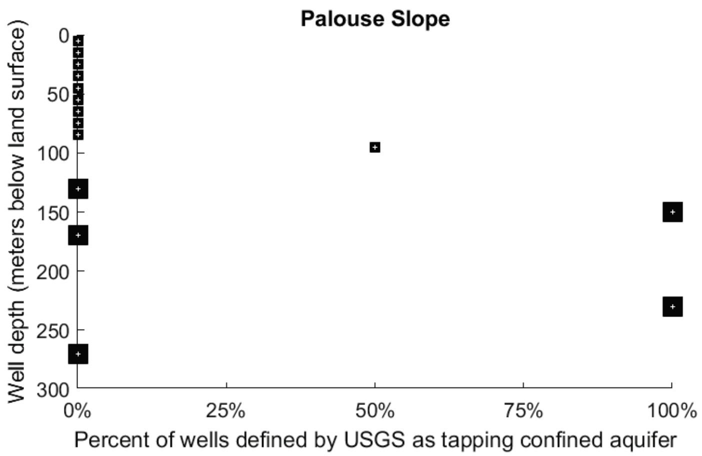

Supplementary Fig. 112. Vertical variations in the prevalence of wells that have been defined as tapping an unconfined or a confined aquifer by the USGS. The smaller squares represent 10 m depth intervals from the land surface to 100 m; the larger squares represent 20 m intervals from 100 m to 300 m below the land surface.

The Palouse Slope is located in central Washington State.

(i) We examined a hydrogeologic cross section generated using a web application available here: https://or.water.usgs.gov/proj/cpras/index.html. No clear confining unit is depicted on the cross section.

(ii) We analysed wells within the study area that the USGS has defined as either unconfined or confined. Only half of the deepest wells in our dataset (depths exceeding 200 m) are defined as tapping a confined aquifer; the deepest well in the dataset (276 m) is defined as tapping an unconfined aquifer.

Depth to confined conditions: >276 m based on (ii) (consistent with hydrogeologic cross section depicted in figure to the left)

The table below presents a series of published quotes (see quotation marks denoting text quoted from another publication, which is cited following the quotation marks with the full reference written in full below the table). The leftmost column lists a title of a hydrogeologic formation depicted in the cross section on the previous page. The rightmost column presents a quote from a hydrogeological study (see base of table for citation). The quote has been annotated with colored text to highlight how we categorized each layer (i.e., see categories in the center column in the table). Specifically: (i) blue text highlights portions of a quote that provide insights into the degree of consolidation of the formation, (ii) red text highlights portions of a quote that categorize the formation as an aquifer or an aquitard (i.e., higher versus lower permeability in the context of local hydrogeologic formations), and (iii) green text highlights portions of a quote that provide information about the lithology of the formation.

Supplementary Table 10. Hydrostratigraphy details for Palouse Slope

Formation name	Category	Quote
Saddle Mountains Basalts	Volcanic aquifer	"The Saddle Mountains unit consists mostly of the **Saddle Mountains Basalt and interbed members**." (Burns et al., 2012). "**The Saddle Mountains unit Kh has been estimated to range from 0.007 to 3,200 ft/d, with a median of about 1 to 2 ft/d**." (Burns et al., 2012).
Wanapum Basalt	Volcanic aquifer	"The Saddle Mountains unit consists mostly of the **Saddle Mountains Basalt and interbed members**." (Burns et al., 2012). "**The Saddle Mountains unit Kh has been estimated to range from 0.007 to 3,200 ft/d, with a median of about 1 to 2 ft/d**." (Burns et al., 2012).
Grande Ronde Basalt	Volcanic aquifer	"The Grande Ronde unit is the **oldest and most extensive of the basalt units**." (Burns et al., 2012). "**The range in Grande Ronde unit Kh values was similar to that for the Wanapum unit, from 0.005 to 5,200 ft/d. Median Kh values for the Grande Ronde unit range from about 0.1 to 5 ft/d (again, excluding value for flow interior)**." (Burns et al., 2012).

Burns, E. R., Snyder, D. T., Haynes, J. V., Waibel, M. S. (2012). Groundwater status and trends for the Columbia Plateau Regional Aquifer System, Washington, Oregon, and Idaho. U.S. Geological Survey Scientific Investigations Report 2012–5261, 52 pp. Accessed February 18, 2021 from http://pubs.er.usgs.gov/publication/sir20125261

3.8 Umatilla Basin and Horse Heaven Hills, Columbia Plateau Regional Aquifer System

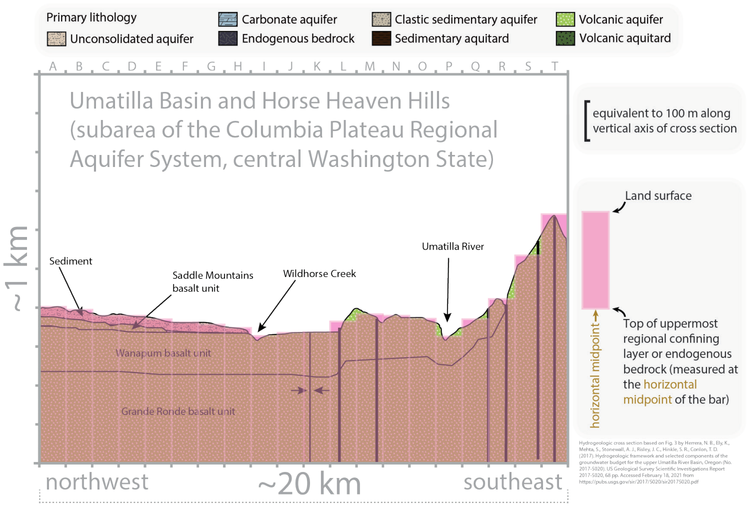

Supplementary Fig. 113. Hydrogeologic cross section. 20 equally spaced transparent pink bars overlie the cross section; each shaded bar depicts the vertical offset from the land surface to the top of the uppermost confining unit or endogenous bedrock.

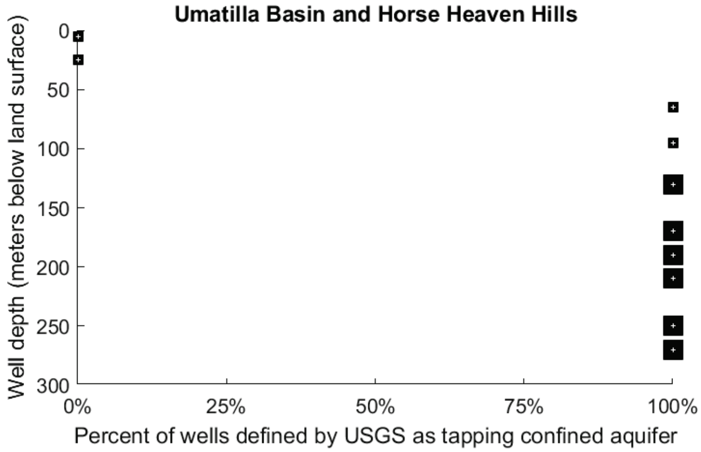

Supplementary Fig. 114. Vertical variations in the prevalence of wells that have been defined as tapping an unconfined or a confined aquifer by the USGS. The smaller squares represent 10 m depth intervals from the land surface to 100 m; the larger squares represent 20 m intervals from 100 m to 300 m below the land surface.

The Umatilla Basin and Horse Heaven Hills are located in southern Washington and northern Oregon.

(i) A hydrogeologic cross section presented in Fig. 3 by Herrera et al. (2017) suggests that the system does not have a clear confining unit. However, the system is confined by basalt flow interiors (see (iii) below).

(ii) We analysed wells within the study area that the USGS has defined as either unconfined or confined. All (100%) wells at depths of 60-70 m and at depths exceeding 60 m are defined as tapping a confined aquifer. No wells in our dataset have depths between 25 m and 69 m; all wells (n=2) with depths of shallower than 25 m are classified as unconfined.

(iii) Herrera et al. (2017) state that (quote): *"The uppermost part of the CRBG is often permeable and unconfined, and has a good hydraulic connection with the overlying alluvial aquifer and, in some cases, streams. Permeable interflow zones at depth are confined by the flow interiors."*

Depth to confined conditions: 60-70 m (based on (ii) above)

Reference: Herrera, N. B., Ely, K., Mehta, S., Stonewall, A. J., Risley, J. C., Hinkle, S. R., Conlon, T. D. (2017). Hydrogeologic framework and selected components of the groundwater budget for the upper Umatilla River Basin, Oregon. US Geological Survey Scientific Investigations Report 2017-5020, 68 pp. Accessed February 18, 2021 from https://pubs.usgs.gov/sir/2017/5020/sir20175020.pdf

The table below presents a series of published quotes (see quotation marks denoting text quoted from another publication, which is cited following the quotation marks with the full reference written in full below the table). The leftmost column lists a title of a hydrogeologic formation depicted in the cross section on the previous page. The rightmost column presents a quote from a hydrogeological study (see base of table for citation). The quote has been annotated with colored text to highlight how we categorized each layer (i.e., see categories in the center column in the table). Specifically: (i) blue text highlights portions of a quote that provide insights into the degree of consolidation of the formation, (ii) red text highlights portions of a quote that categorize the formation as an aquifer or an aquitard (i.e., higher versus lower permeability in the context of local hydrogeologic formations), and (iii) green text highlights portions of a quote that provide information about the lithology of the formation.

Supplementary Table 11. Hydrostratigraphy details for Umatilla Basin and Horse Heaven Hills

Formation name	Category	Quote
Sediment	Unconsolidated aquifer *(large proportion of silt results in low permeability)*	"The **sedimentary deposits** overlying the CRBG consist of four types of Tertiary and Quaternary sediments defined by Hogenson (1964) and refined by Ferns and Ely (2006) (figs. 3 and 6): (1) **recent stream alluvium, including Pleistocene terrace deposits (Qal), (2) Quaternary alluvial fan deposits (Qf), (3) Quaternary landslide deposits (Qls), (4) loess and fine-grained sandstone (QTs) interpreted as late Miocene wind-reworked, fine grained deposits correlative to the McKay Formation, and (5) late-Miocene to early-Pliocene conglomerate of the McKay Formation (Tms)** (fig. 3)." (Herrera et al., 2017). "**Mean hydraulic conductivity and storage coefficient values for the sedimentary unit were determined from well data in the Hermiston-Umatilla area and estimated to be 24,000 ft/d and 0.15, respectively** (Davies-Smith and others, 1988)." (Herrera et al., 2017).
Saddle Mountains	Volcanic aquifer *(upper most part is unconfined)*	"**Conceptually, the CRBG is a series of productive aquifers consisting of relatively high permeability interflow zones separated by the low permeability flow interiors**. The uppermost part of the CRBG is often **permeable and unconfined**, and **has a good hydraulic connection with the overlying alluvial aquifer and, in some cases, streams**." (Herrera et al., 2017). "Mean values of hydraulic conductivity determined from about 1,700 short-duration specific capacity tests in the Umatilla River Basin are 18 ft/d for the **Saddle Mountains basalt** unit, 170 ft/d for the Wanapum basalt unit, and 65 ft/d for the Grande Ronde basalt unit (Davies-Smith and others, 1988)." (Herrera et al., 2017).
Wanapum basalt unit	Volcanic aquifer	"**Conceptually, the CRBG is a series of productive aquifers consisting of relatively high permeability interflow zones separated by the low permeability flow interiors**. The uppermost part of the CRBG is often **permeable and unconfined**, and **has a good hydraulic connection with the overlying alluvial aquifer and, in some cases, streams**." (Herrera et al., 2017). "Mean values of hydraulic conductivity determined from about 1,700 short-duration specific capacity tests in the Umatilla River Basin are 18 ft/d for the Saddle Mountains basalt unit, 170 ft/d for the **Wanapum basalt** unit, and 65 ft/d for the Grande Ronde basalt unit (Davies-Smith and others, 1988)." (Herrera et al., 2017).

Formation name	Category	Quote
Grande Ronde basalt unit	Volcanic aquifer	“**Conceptually, the CRBG is a series of productive aquifers consisting of relatively high permeability interflow zones separated by the low permeability flow interiors**. The uppermost part of the CRBG is often **permeable and unconfined**, and **has a good hydraulic connection with the overlying alluvial aquifer and, in some cases, streams**.” (Herrera et al., 2017). “Mean values of hydraulic conductivity determined from about 1,700 short-duration specific capacity tests in the Umatilla River Basin are 18 ft/d for the Saddle Mountains basalt unit, 170 ft/d for the Wanapum basalt unit, and 65 ft/d for the **Grande Ronde** basalt unit (Davies-Smith and others, 1988).” (Herrera et al., 2017).

Herrera, N.B., Ely, K. Mehta, S., Stonewall, A.J., Risley, J.C., Hinkle, S.R., Conlon, T.D. (2017). Hydrogeologic framework and selected components of the groundwater budget for the upper Umatilla River Basin, Oregon. No. 2017-5020. US Geological Survey. https://pubs.er.usgs.gov/publication/sir20175020

3.9 Yakima Basin, Columbia Plateau Regional Aquifer System

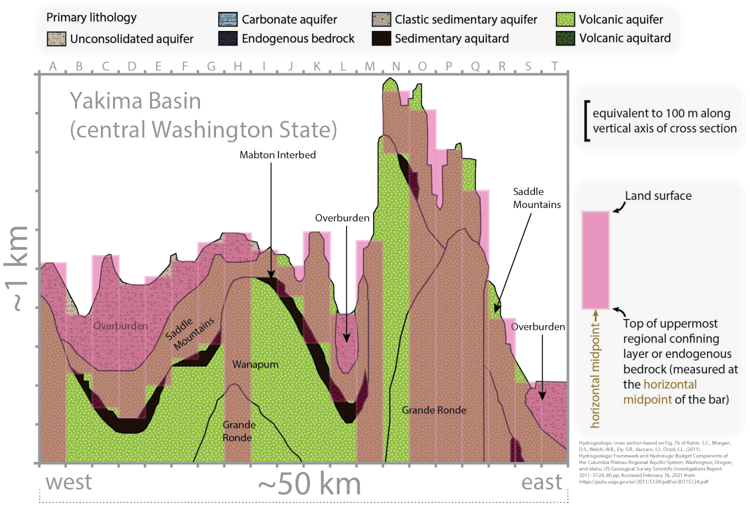

Supplementary Fig. 115. Hydrogeologic cross section. 20 equally spaced transparent pink bars overlie the cross section; each shaded bar depicts the vertical offset from the land surface to the top of the uppermost confining unit or endogenous bedrock.

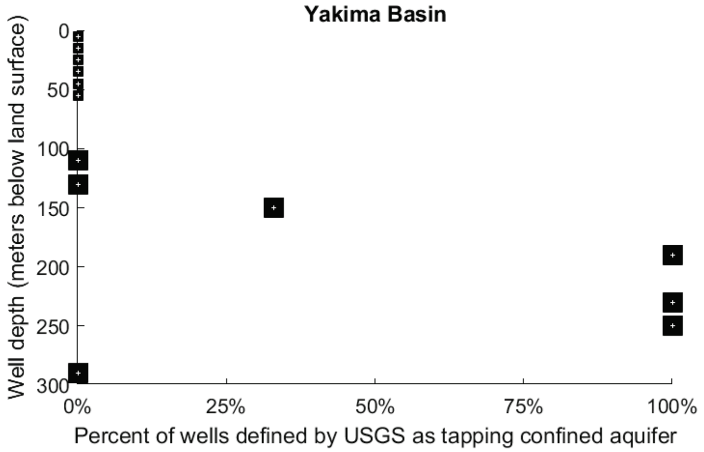

Supplementary Fig. 116. Vertical variations in the prevalence of wells that have been defined as tapping an unconfined or a confined aquifer by the USGS. The smaller squares represent 10 m depth intervals from the land surface to 100 m; the larger squares represent 20 m intervals from 100 m to 300 m below the land surface.

The Yakima Basin is located in central Washington State.

(i) A hydrogeologic section by Kahle et al. (2011) in their Fig. 7b demonstrates the high degree of topographic and geologic complexity in the Yakima Basin. Their cross section suggests that the uppermost confining unit is 282 meters below land surface (25th-75th percentile range: 209 m to >529 m meters below land surface)

(ii) We analysed wells within the study area that the USGS has defined as either unconfined or confined. Three or the four deepest wells in our dataset (depths exceeding 200 m) are defined as tapping a confined aquifer; the deepest well in the dataset (291 m, located on the northeast periphery of Grandview) is defined as tapping an unconfined aquifer.

Depth to confined conditions: 280-300 m (based on (i) above)

Reference: Kahle, S.C., Morgan, D.S., Welch, W.B., Ely, S.R., Vaccaro, J.J., Orzol, L.L. (2011). Hydrogeologic Framework and Hydrologic Budget Components of the Columbia Plateau Regional Aquifer System, Washington, Oregon, and Idaho. US Geological Survey Scientific Investigations Report 2011–5124, 80 pp. Accessed February 16, 2021 from https://pubs.usgs.gov/sir/2011/5124/pdf/sir20115123.pdf

The table below presents a series of published quotes (see quotation marks denoting text quoted from another publication, which is cited following the quotation marks with the full reference written in full below the table). The leftmost column lists a title of a hydrogeologic formation depicted in the cross section on the previous page. The rightmost column presents a quote from a hydrogeological study (see base of table for citation). The quote has been annotated with colored text to highlight how we categorized each layer (i.e., see categories in the center column in the table). Specifically: (i) blue text highlights portions of a quote that provide insights into the degree of consolidation of the formation, (ii) red text highlights portions of a quote that categorize the formation as an aquifer or an aquitard (i.e., higher versus lower permeability in the context of local hydrogeologic formations), and (iii) green text highlights portions of a quote that provide information about the lithology of the formation.

Supplementary Table 12. Hydrostratigraphy details for the Yakima Basin

Formation name	Category	Quote
Overburden	Unconsolidated aquifer *(consolidated aquifer also exists)*	"Overburden deposits are diverse in lithology and, thus, so are their hydraulic characteristics (table 2). The deposits, which consist of **unconsolidated and consolidated material** of **alluvial, glacial, lacustrine, wind-blown, and volcanic origins**, form **important water-bearing units, as well as semiconfining to confining units**." (Burns et al., 2012).
Saddle Mountains	Volcanic aquifer	"The Saddle Mountains unit consists mostly of the **Saddle Mountains Basalt and interbed members**." (Burns et al., 2012). "**The Saddle Mountains unit Kh has been estimated to range from 0.007 to 3,200 ft/d, with a median of about 1 to 2 ft/d.**" (Burns et al., 2012).
Mabton Interbed Unit	Sedimentary **aquitard** (consolidated or semi-consolidated rock) *(laterally extensive but thin)*	"The Mabton unit generally consists of **clay, shale, claystone, clay with basalt, clay with sand, and sandstone**." (Burns et al., 2012). "**The confining units are** equivalent to the Saddle Mountains-Wanapum and Wanapum-Grande Ronde interbeds, referred to in this study as the **Mabton** and Vantage **Interbeds**, respectively." (Burns et al., 2012).
Wanapum	Volcanic aquifer	"The Wanapum unit, **composed mostly of basalt and interbed members of the Wanapum basalt**, is in most of the north-central part of the study area" (Burns et al., 2012). "**The Wanapum unit had a slightly larger range (0.007 to 5,200 ft/d) than the Saddle Mountains unit, and the median reported Kh for the Wanapum unit ranges from about 3 to 11 ft/d (excluding value for flow interior)**." (Burns et al., 2012).
Grande Ronde	Volcanic aquifer	"The Grande Ronde unit is the **oldest and most extensive of the basalt units**." (Burns et al., 2012). "**The range in Grande Ronde unit Kh values was similar to that for the Wanapum unit, from 0.005 to 5,200 ft/d. Median Kh values for the Grande Ronde unit range from about 0.1 to 5 ft/d (again, excluding value for flow interior)**." (Burns et al., 2012).
Older bedrock	Endogenous bedrock *(water may present in the bed rock)*	"The areas bordering and underlying the CPRAS include **metamorphic (crystalline), sedimentary, volcanic, and intrusive and extrusive igneous rocks**. In general, the older bedrock has **lower values of porosity and permeability** than the overburden and CRBG units." (Burns et al., 2012).

Burns, E. R., Snyder, D. T., Haynes, J. V., Waibel, M. S. (2012). Groundwater status and trends for the Columbia Plateau Regional Aquifer System, Washington, Oregon, and Idaho. U.S. Geological Survey Scientific Investigations Report 2012–5261, 52 pp. Accessed February 18, 2021 from http://pubs.er.usgs.gov/publication/sir20125261

3.10 Stockton Plateau, Edwards-Trinity Aquifer System

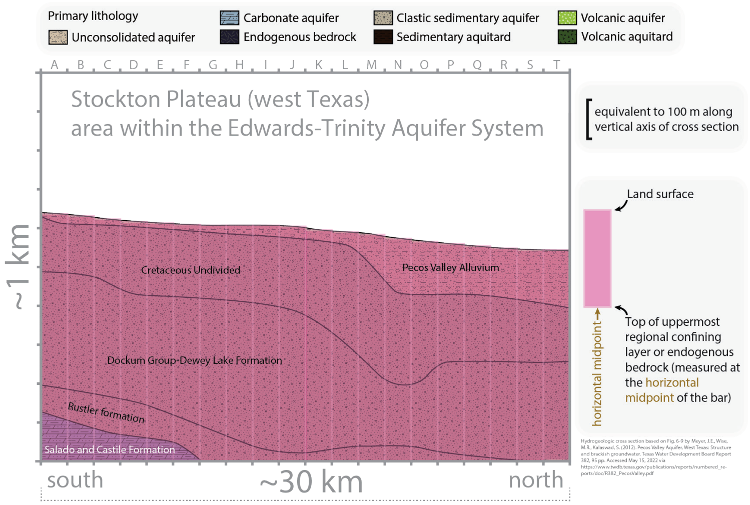

Supplementary Fig. 117. Hydrogeologic cross section. 20 equally spaced transparent pink bars overlie the cross section; each shaded bar depicts the vertical offset from the land surface to the top of the uppermost confining unit or endogenous bedrock.

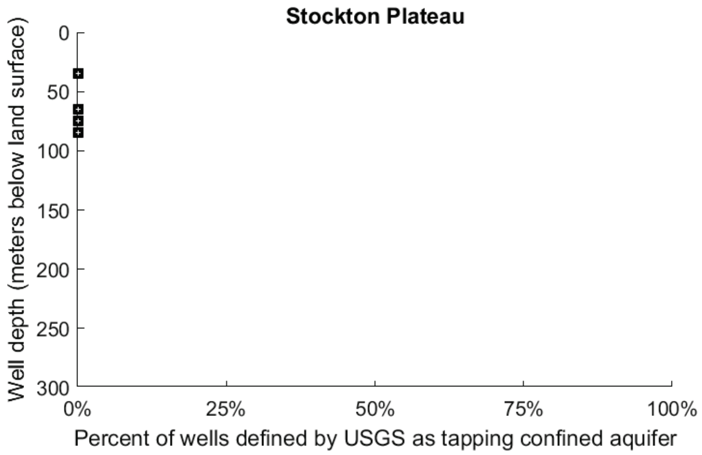

Supplementary Fig. 118. Vertical variations in the prevalence of wells that have been defined as tapping an unconfined or a confined aquifer by the USGS. The smaller squares represent 10 m depth intervals from the land surface to 100 m; the larger squares represent 20 m intervals from 100 m to 300 m below the land surface.

The Stockton Plateau is located in western Texas and forms part of the broader Edwards-Trinity Aquifer System.

(i) A hydrogeologic cross section presented in Fig. 6-9 by Meyer et al. (2012) does not depict a clear confining unit within the aquifer system within the uppermost 190-340 m of the aquifer system (median of pink transparent pink bars suggests a lack of a clear confining unit in the uppermost 249 m below the land surface).

(ii) We analysed wells within the study area that the USGS has defined as either unconfined or confined. Only four wells were available in our dataset; they have depths ranging from 30 m to 87 m, and all are classified as tapping unconfined aquifers.

Depth to confined conditions: >249 meters below land surface (based on (i) above)

Reference: Meyer, J.E., Wise, M.R., Kalaswad, S. (2012). Pecos Valley Aquifer, West Texas: Structure and brackish groundwater. Texas Water Development Board Report 382, 95 pp. Accessed May 15, 2022 via https://www.twdb.texas.gov/publications/reports/numbered_reports/doc/R382_PecosValley.pdf

The table below presents a series of published quotes (see quotation marks denoting text quoted from another publication, which is cited following the quotation marks with the full reference written in full below the table). The leftmost column lists a title of a hydrogeologic formation depicted in the cross section on the previous page. The rightmost column presents a quote from a hydrogeological study (see base of table for citation). The quote has been annotated with colored text to highlight how we categorized each layer (i.e., see categories in the center column in the table). Specifically: (i) blue text highlights portions of a quote that provide insights into the degree of consolidation of the formation, (ii) red text highlights portions of a quote that categorize the formation as an aquifer or an aquitard (i.e., higher versus lower permeability in the context of local hydrogeologic formations), and (iii) green text highlights portions of a quote that provide information about the lithology of the formation.

Supplementary Table 13. Hydrostratigraphy details for Stockton Plateau

Formation name	Category	Quote
Pecos Valley Alluvium	Unconsolidated aquifer *(unconfined aquifer, although deeper sections may have local confining layers)*	"The **Pecos Valley Aquifer**, previously known as the Cenozoic Pecos **Alluvium**, is designated as a major aquifer in Texas (Ashworth and Hopkins, 1995; George and others, 2011)." (Meyer et al., 2012). "The stratigraphic top of the Pecos Valley **Alluvium consists of post-Cretaceous sediments** that are exposed at ground surface in the study area." (Meyer et al., 2012)
Cretaceous Undivided	Clastic sedimentary **aquifer** (consolidated or semi-consolidated rock) *(carbonate rocks can be found)*	"The unit mapped as Cretaceous Undivided consists of **Cretaceous sediments deposited** unconformably on the Triassic Dockum Group or the Permian Dewey Lake Formation (Table 6-1). **The sediments** consist of **clay, sand, and limestone deposited in continental to marine depositional settings at the onset of the marine transgression in West Texas**. The undifferentiated Cretaceous Undivided unit constitutes **the hydrostratigraphic unit for the Edwards-Trinity (Plateau) Aquifer, classified as a major aquifer in Texas** (Ashworth and Hopkins, 1995; George and others, 2011)." (Meyer et al., 2012)
Dockum Group-Dewey Lake Formation	Clastic sedimentary **aquifer** (consolidated or semi-consolidated rock) *(water quality issue due to dissolution of halite)*	"The **sediments** consist of alternating **shale, siltstone, sandstone, and gravel that were deposited in a variety of fluvial, lacustrine, and deltaic environments** (Bradley and Kalaswad, 2003). **The Dockum Group constitutes the hydrostratigraphic unit for the Dockum Aquifer which is classified as a minor aquifer in Texas** (Ashworth and Hopkins, 1995; George and others, 2011)." (Meyer et al., 2012)
Rustler formation	Clastic sedimentary **aquifer** (consolidated or semi-consolidated rock) *(water quality issue due to dissolution of halite)*	"The Rustler Formation consists of Upper Permian **sediments deposited** unconformably on the Permian Salado Formation. The **sediments consist of shale, silt, sandstone, dolomite, and the evaporites, halite and gypsum (anhydrite at depth)**. The **Rustler Formation constitutes the hydrostratigraphic unit for the Rustler Aquifer**, although the areal extent of the formation is much larger than the mapped limits of the aquifer." (Meyer et al., 2012)
Salado and Castile Formation	Carbonate aquifer *(Evaporites exist)*	"**Capitan Reef Complex Aquifer**". (Meyer et al., 2012). "The structurally elevated ridge separating the Pecos and Monument Draw troughs is underlain by the **thickest** sections of **evaporites** of the Salado and Castile formations". (Meyer et al., 2012)

Meyer, J.E., Wise, M.R., Kalaswad, S. (2012). Pecos Valley aquifer, west Texas: structure and brackish groundwater. Texas Water Development Board Report. Accessed August 8, 2022 via https://www.twdb.texas.gov/publications/reports/numbered_reports/doc/R382_PecosValley.pdf

3.11 Trinity Aquifer System, Edwards-Trinity Aquifer System

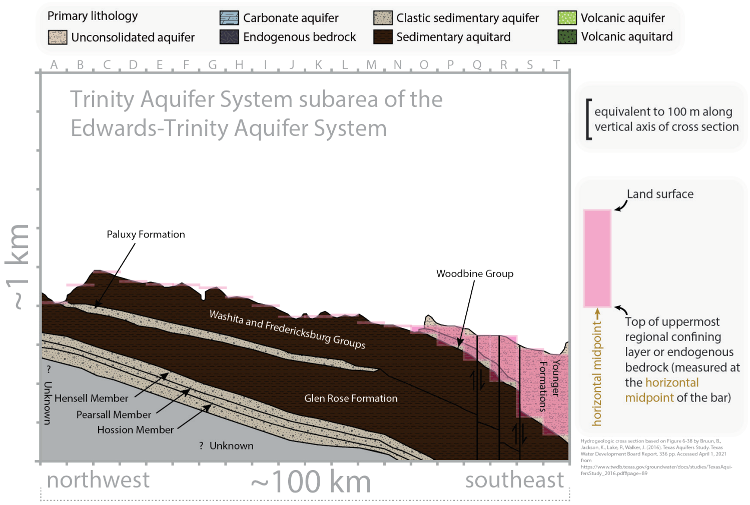

Supplementary Fig. 119. Hydrogeologic cross section. 20 equally spaced transparent pink bars overlie the cross section; each shaded bar depicts the vertical offset from the land surface to the top of the uppermost confining unit or endogenous bedrock.

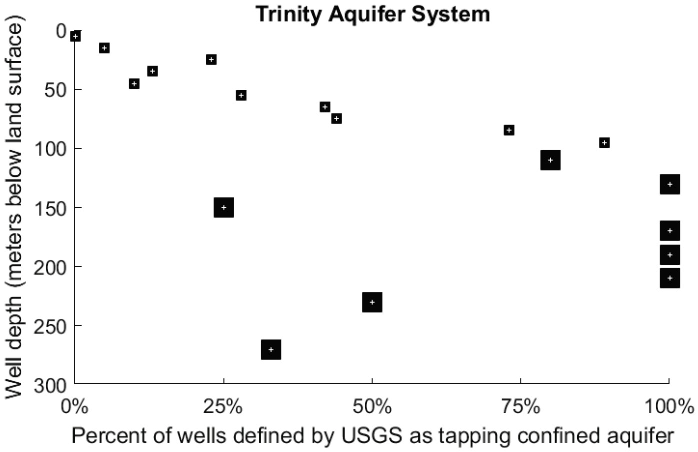

Supplementary Fig. 120. Vertical variations in the prevalence of wells that have been defined as tapping an unconfined or a confined aquifer by the USGS. The smaller squares represent 10 m depth intervals from the land surface to 100 m; the larger squares represent 20 m intervals from 100 m to 300 m below the land surface.

The Trinity Aquifer System is located in central Texas.

(i) A hydrogeologic cross section presented in Fig. 6-38 by Bruun et al. (2016) depicts layered sequences of sedimentary aquifers and aquitards. The uppermost Washita and Fredericksburg Formations are shown as aquitards, but may contain permeable portions that serve as aquifers (see (iii) below).

(ii) We analysed wells within the study area that the USGS has defined as either unconfined or confined. Most (>80%) wells at depths of 90-100 m and at depths exceeding 90 m are defined as tapping a confined aquifer.

(iii) Bruun et al. (2016) state (quote) *"Sand distribution and thickness largely controls the productivity of the aquifer. The depositional environment in the Cretaceous Period resulted in a layered system of aquifers and aquitards in the northern Trinity Group."* Mace et al. (1994) state (quote) *"Hydrologic parameters for the Washita and Fredericksburg Groups… were estimated on the basis of rock type. These units are composed of approximately 40 percent shale and 60 percent limestone…"*

Depth to confined conditions: 90-100 meters below land surface (based on (ii) above)

References: Bruun, B., Jackson, K., Lake, P., Walker, J. (2016). Texas Aquifers Study. Texas Water Development Board Report. 336 pp. Accessed April 1, 2021 from https://www.twdb.texas.gov/groundwater/docs/studies/TexasAquifersStudy_2016.pdf#page=89

Mace, R.E., Nance, H.S., Dutton, A.R. (1996). Geologic and Hydrogeologic Framework of Regional Aquifers in the Twin Mountains, Paluxy, and Woodbine Formations near the SSC Site, North-Central Texas. Topical Report to Taxes National Research Laboratory. https://www.beg.utexas.edu/files/publications/contract-reports/CR1994-Mace-1.pdf

The table below presents a series of published quotes (see quotation marks denoting text quoted from another publication, which is cited following the quotation marks with the full reference written in full below the table). The leftmost column lists a title of a hydrogeologic formation depicted in the cross section on the previous page. The rightmost column presents a quote from a hydrogeological study (see base of table for citation). The quote has been annotated with colored text to highlight how we categorized each layer (i.e., see categories in the center column in the table). Specifically: (i) blue text highlights portions of a quote that provide insights into the degree of consolidation of the formation, (ii) red text highlights portions of a quote that categorize the formation as an aquifer or an aquitard (i.e., higher versus lower permeability in the context of local hydrogeologic formations), and (iii) green text highlights portions of a quote that provide information about the lithology of the formation.

Supplementary Table 14. Hydrostratigraphy details for the Trinity Aquifer System

**Formation name**	**Category**	**Quote**
Younger Formation	Unconfined aquifer	"**Sand, silt, clay and gravel**." (Nordstrom, 1982). "**Yields small to large amount of water to wells along the red river**." (Nordstrom, 1982).
Woodbine group	Sedimentary **aquifer** (consolidated or semi-consolidated rock)	"The Woodbine Group is the only **important aquifer of the Gulf Series** in the area covered by this report. It consists of **sand, sandstone, and clay** and is capable of yielding small to large amounts of water." (Nordstrom, 1982).
Washita and Fredericksburg Groups	Sedimentary **aquitard** (consolidated or semi-consolidated rock)	"Both the Washita and Fredericksburg Groups of the Comanche Series consist predominantly of **limestone**, **shale, clay, and marl** and **yield only small amounts of water to localized areas**." (Nordstrom, 1982).
Paluxy Formation	Sedimentary **aquifer** (consolidated or semi-consolidated rock)	"The Paluxy **is composed predominantly of fine- to coarse-grained, friable, homogeneous, white quartz sand interbedded with sandy, silty, calcareous, or waxy clay and shale**. In general, coarse-grained sand is in the lower part." (Nordstrom, 1982). "**The Paluxy Formation is an important aquifer** in the study region and during 1974, **produced over 10,000 acre-feet (12.3 hm3) of water for municipal and industrial use and provided water to many domestic and livestock wells**." (Nordstrom, 1982).
Glen Rose Formation	Sedimentary **aquitard** (consolidated or semi-consolidated rock)	"The Glen Rose is predominantly a **limestone** and **yields small quantities of water only to localized areas**." (Nordstrom, 1982). "A middle section of Antlers contains considerably more clay beds than the upper or lower sections, and to the south, near the updip limit of the **Glen Rose Formation, limestone beds also occur**." (Nordstrom, 1982).
Travis Peak/Twin Mountain Formation includes:<br>Hensell , Pearsall Member, and Hossion Member	Sedimentary **aquifer** (consolidated or semi-consolidated rock)	"Originally the **basal Cretaceous bed** was named the Travis Peak Formation, but the name was changed to the Twin Mountains Formation in north-central Texas (Fisher and Rodda, 1966)." (Nordstrom, 1982). "The Twin Mountains consists of **a basal conglomerate of chert and quartz, grading upward into coarse- to fine-grained sand interspersed with varicolored shale**." (Nordstrom, 1982). "**The Twin Mountains Formation is the most important source of ground water for a**

Formation name	Category	Quote
		**large part of the study region ant yields moderate to large quantities of fresh to slightly saline water to municipal and industrial wells**." (Nordstrom, 1982).

Nordstrom, P.L., 1982, Occurrence, availability, and chemical quality of ground water in the Cretaceous aquifers of northcentral Texas: Texas Department of Water Resources Report 269, v. 1, 109 p., and v. 2, 387 p. Accessed on June 15, 2022 via
https://www.twdb.texas.gov/publications/reports/numbered_reports/doc/R269/R269v1/R269v1.pdf

3.12 Dougherty Plain and Marianna Lowlands, Floridan Aquifer System

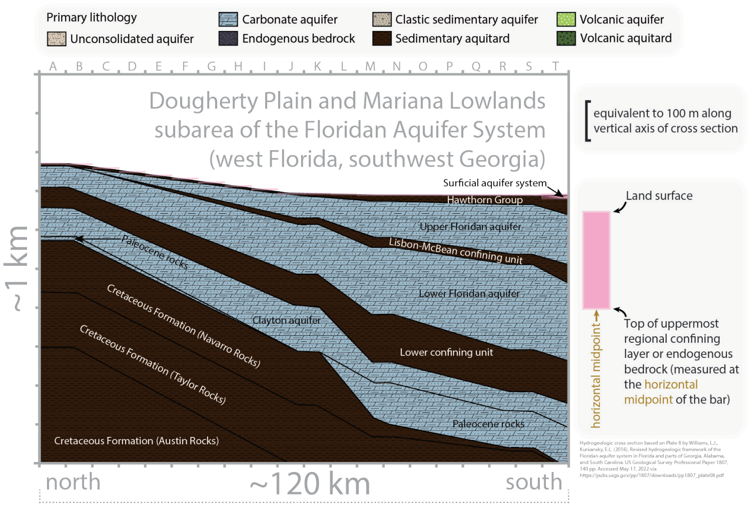

Supplementary Fig. 121. Hydrogeologic cross section. 20 equally spaced transparent pink bars overlie the cross section; each shaded bar depicts the vertical offset from the land surface to the top of the uppermost confining unit or endogenous bedrock.

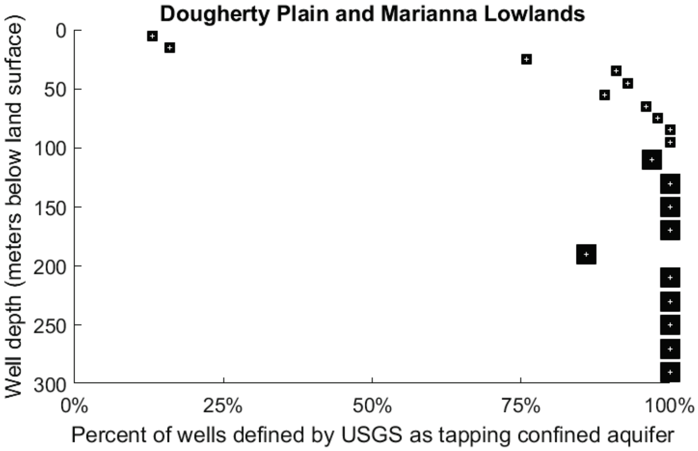

Supplementary Fig. 122. Vertical variations in the prevalence of wells that have been defined as tapping an unconfined or a confined aquifer by the USGS. The smaller squares represent 10 m depth intervals from the land surface to 100 m; the larger squares represent 20 m intervals from 100 m to 300 m below the land surface.

The Dougherty Plain and Marianna Lowlands are located in southwestern Georgia, southeastern Alabama, and western Florida.

(i) A hydrogeologic cross section presented in Plate 8 by Williams and Kuniansky (2016) depicts a shallow confining unit (Hawthorn Group) underlain by carbonate aquifers and confining units.

(ii) We analysed wells within the study area that the USGS has defined as either unconfined or confined. Most (>80%) wells at depths of 30-40 m and at depths exceeding 30 m are defined as tapping a confined aquifer.

Depth to confined conditions: 30-40 meters below land surface (based on (ii) above)

Reference: Williams, L.J., Kuniansky, E.L. (2016). Revised hydrogeologic framework of the Floridan aquifer system in Florida and parts of Georgia, Alabama, and South Carolina. US Geological Survey Professional Paper 1807, 140 pp. Accessed March 31, 2021 from https://pubs.usgs.gov/pp/1807/pdf/pp1807.pdf

The table below presents a series of published quotes (see quotation marks denoting text quoted from another publication, which is cited following the quotation marks with the full reference written in full below the table). The leftmost column lists a title of a hydrogeologic formation depicted in the cross section on the previous page. The rightmost column presents a quote from a hydrogeological study (see base of table for citation). The quote has been annotated with colored text to highlight how we categorized each layer (i.e., see categories in the center column in the table). Specifically: (i) blue text highlights portions of a quote that provide insights into the degree of consolidation of the formation, (ii) red text highlights portions of a quote that categorize the formation as an aquifer or an aquitard (i.e., higher versus lower permeability in the context of local hydrogeologic formations), and (iii) green text highlights portions of a quote that provide information about the lithology of the formation.

Supplementary Table 15. Hydrostratigraphy details for the Dougherty Plain and Marianna Lowlands

Formation name	Category	Quote
Undifferentiated Sand, Silt and Clay	Unconsolidated aquifer	"The **surficial aquifer system** consists mostly of **sand and locally contains gravel and sandy lime stone** of Pliocene to Holocene age. Where these **sediments are thick and highly permeable,**" (Williams, and Kuniansky, 2016). "The surficial aquifer system forms **a thin irregular blanket of terrace and alluvial sands** that can act as an important source sink layer for temporary storage of groundwater that may **ultimately recharge the underlying Floridan aquifer system**. (Williams and Kuniansky, 2016)
Hawthorn Group	Sedimentary **aquitard** (consolidated or semi-consolidated rock)	"The **thickest and most extensive Miocene unit** in the study area is the **Hawthorn Group**. The lithology of the Hawthorn varies greatly between areas, but mostly consists of **phosphatic clay, silt, and sand that range in color from cream or gray to green to brown**." (Williams and Kuniansky, 2016). "The entire formation **forms a thick, generally clastic**, **highly variable sequence of lower permeability** rock that, where present, is **considered to be the upper confining unit** of **the Floridan aquifer system**." (Williams and Kuniansky, 2016).
Upper Floridan aquifer	Carbonate aquifer	"The Upper Floridan aquifer includes the **uppermost or shallowest permeable zones** in the Floridan aquifer system." (Williams, and Kuniansky, 2016) | "The Floridan aquifer system includes the vertically continuous **carbonate-rock system** described by Miller (1986)". (Williams, and Kuniansky, 2016). "the **carbonate rocks** of the Upper Floridan aquifer may directly overlie clastic rocks of the Lisbon and Claiborne aquifers;" (Williams, and Kuniansky, 2016). "Over a large part of the Floridan aquifer system, the top is marked **by Oligocene rocks (Suwannee Limestone or equivalent)** where such rocks are **permeable and in hydraulic connection with the main part of the system**." (Williams and Kuniansky, 2016). "the **Upper Floridan** aquifer consists mostly of **Oligocene Suwannee Limestone (if present) and late-Eocene Ocala Limestone**." (Williams and Kuniansky, 2016).
Lisbon-McBean confining unit	Sedimentary **aquitard** (consolidated or semi-consolidated rock)	"The composite unit also has been identified by Faye and Mayer (1997) as the **Lisbon-McBean confining unit**." (Williams and Kuniansky, 2016). Table 7 of (Williams and Kuniansky, 2016), under the "Equivalent hydrogeologic unit": "Lisbon-McBean confining unit" and "Lithology" as "**Clay,**

Formation name	Category	Quote
		**sand, argillaceous limestone**", under the "Water-bearing properites" "**Mostly confining**, may vary laterally depend on lithology".
Lower Floridan aquifer	Carbonate aquifer	"The **Lower Floridan aquifer** includes **all permeable and less-permeable zones** below (1) the MAPCU in peninsular Florida, (2) the BCCU in the western Florida panhandle and contiguous areas in southwestern Alabama, and (3) the LISAPCU in southeastern Alabama, Georgia, western South Carolina and northern Florida." (Williams and Kuniansky, 2016).
Lower confining unit	Sedimentary **aquitard** (consolidated or semi-consolidated rock)	"The Floridan is **underlain everywhere by low-permeability rocks** called **the lower confining unit**, which separates the Floridan aquifer system from older, deeper aquifers of the Southeastern Coastal Plain aquifer system." (Williams and Kuniansky, 2016). "The **base of the Floridan aquifer system** is marked by the **lower confining unit**, consisting of **predominantly low-permeability late Paleocene to middle Eocene rocks**." (Williams and Kuniansky, 2016).
Clayton aquifer *(sometimes, sand can dominate)*	Carbonate aquifer	"The **limestone** of **the Clayton aquifer** is not hydraulically connected to other Tertiary limestones of the Floridan aquifer system." (Williams and Kuniansky, 2016). "In western Georgia, the uppermost part of the Clayton is a **hard, sandy, fossiliferous limestone** that constitutes **an important aquifer** (Clarke and others, 1984)." (Williams and Kuniansky, 2016).
Paleocene rocks	Carbonate aquifer *(though it has lower permeability, we categorize this unit as a carbonate rock unit as this is the depiction in Fig. Plate 8 by Williams and Kuniansky, 2016)*	"**Paleocene rocks** in the study area can be categorized into three groups. The first group, which is the most important to this study, primarily consists of **interbedded dolomite and anhydrite** of the **Cedar Keys Formation**, which underlies part of southeastern Georgia and all of peninsular Florida." (Williams and Kuniansky, 2016). "**Interbedded dolomite and anhydrite, both being of lower permeability**, compose the lower two-thirds of the Cedar Keys Formation of peninsular Florida and southeastern Georgia. This **carbonate-evaporite sequence forms the base of the Floridan aquifer system**." (Williams and Kuniansky, 2016).
Cretaceous formation	Sedimentary **aquitard** (consolidated or semi-consolidated rock)	"South of Brunswick, Ga., the base of the system drops several hundred feet into **late Cretaceous rocks** consisting of **soft, friable, possibly Tayloran age, limestone with the permeable, late Cretaceous, Navarroan age Lawson Limestone above included as part of the Lower Floridan aquifer** (Miller, 1986)." (Williams and Kuniansky, 2016). Figure 2 of (Williams and Kuniansky, 2016) under "Hydrogeology" in "Florida" "**Confining unit**". (Williams et al. 2016) under the "Hydrogeology" in "Florida" "**confining unit**".

Williams, L.J., Kuniansky, E.L. (2016). Revised hydrogeologic framework of the Floridan aquifer system in Florida and parts of Georgia, Alabama, and South Carolina (ver. 1.1, March 2016): US Geological Survey Professional Paper 1807, 140 p., Accessed August 8, 2022 via https://pubs.er.usgs.gov/publication/pp1807

Williams, L.J., Raines, J.E., Lanning, A.E. (2016). Geophysical log database for the Floridan aquifer system and southeastern Coastal Plain aquifer system in Florida and parts of Georgia, Alabama, and South Carolina (ver. 1.1, December 2016): U.S. Geological Survey Data Series 760, 12 pp. Accessed August 8, 2022 via http://pubs.usgs.gov/ds/760/

3.13 Eastern Flatwoods Southshores, Floridan Aquifer System

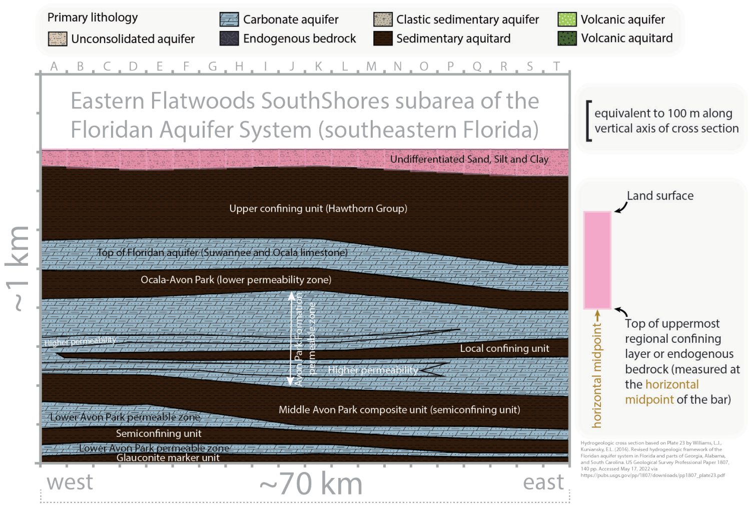

Supplementary Fig. 123. Hydrogeologic cross section. 20 equally spaced transparent pink bars overlie the cross section; each shaded bar depicts the vertical offset from the land surface to the top of the uppermost confining unit or endogenous bedrock.

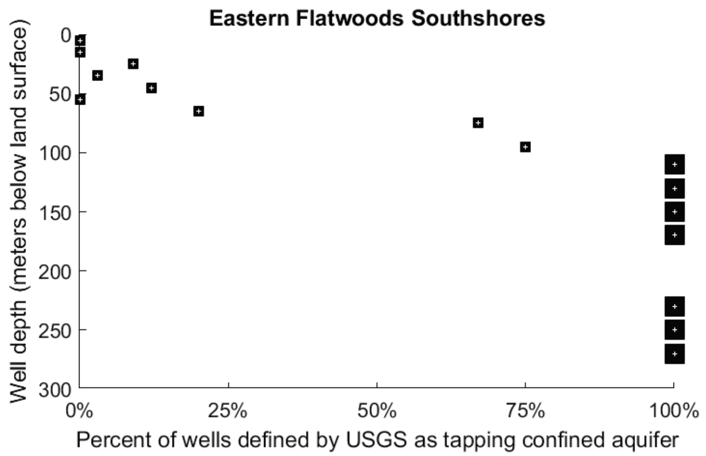

Supplementary Fig. 124. Vertical variations in the prevalence of wells that have been defined as tapping an unconfined or a confined aquifer by the USGS. The smaller squares represent 10 m depth intervals from the land surface to 100 m; the larger squares represent 20 m intervals from 100 m to 300 m below the land surface.

The Eastern Flatwoods Southshores is located in southeastern Florida.

(i) A hydrogeologic cross section presented in Plate 2 by Williams and Kuniansky (2016) shows undifferentiated sand, silt and clay (~70 m thick) underlain by the Hawthorn Group confining unit.

(ii) We analysed wells within the study area that the USGS has defined as either unconfined or confined. Most (>80%) wells at depths of 100-120 m and at depths exceeding 100 m are defined as tapping a confined aquifer.

Depth to confined conditions: 100-120 meters below land surface (based on (ii) above)

Reference: Williams, L.J., Kuniansky, E.L. (2016). Revised hydrogeologic framework of the Floridan aquifer system in Florida and parts of Georgia, Alabama, and South Carolina. US Geological Survey Professional Paper 1807, 140 pp. Accessed March 31, 2021 from https://pubs.usgs.gov/pp/1807/pdf/pp1807.pdf

The table below presents a series of published quotes (see quotation marks denoting text quoted from another publication, which is cited following the quotation marks with the full reference written in full below the table). The leftmost column lists a title of a hydrogeologic formation depicted in the cross section on the previous page. The rightmost column presents a quote from a hydrogeological study (see base of table for citation). The quote has been annotated with colored text to highlight how we categorized each layer (i.e., see categories in the center column in the table). Specifically: (i) blue text highlights portions of a quote that provide insights into the degree of consolidation of the formation, (ii) red text highlights portions of a quote that categorize the formation as an aquifer or an aquitard (i.e., higher versus lower permeability in the context of local hydrogeologic formations), and (iii) green text highlights portions of a quote that provide information about the lithology of the formation.

Supplementary Table 16. Hydrostratigraphy details for the Eastern Flatwoods Southshores

Formation name	Category	Quote
Undifferentiated Sand, Silt and Clay	Unconsolidated aquifer	"The **surficial aquifer system** consists mostly of **sand and locally contains gravel and sandy lime stone** of Pliocene to Holocene age. Where these **sediments are thick and highly permeable,**" (Williams, and Kuniansky, 2016). "The surficial aquifer system forms **a thin irregular blanket of terrace and alluvial sands** that can act as an important source sink layer for temporary storage of groundwater that may **ultimately recharge the underlying Floridan aquifer system**. (Williams, and Kuniansky, 2016)
Upper confining unit (Hawthorn Group)	Sedimentary **aquitard** (consolidated or semi-consolidated rock)	"The **thickest and most extensive Miocene unit** in the study area is the **Hawthorn Group**. The lithology of the Hawthorn varies greatly between areas, but mostly consists of **phosphatic clay, silt, and sand that range in color from cream or gray to green to brown**." (Williams and Kuniansky, 2016). "The entire formation **forms a thick, generally clastic, highly variable sequence of lower permeability** rock that, where present, is **considered to be the upper confining unit** of **the Floridan aquifer system**." (Williams and Kuniansky, 2016).
Top of Floridan aquifer (Suwannee and Ocala limestone)	Carbonate aquifer	"The Upper Floridan aquifer includes the **uppermost or shallowest permeable zones** in the Floridan aquifer system." (Williams, and Kuniansky, 2016) | "The Floridan aquifer system includes the vertically continuous **carbonate-rock system** described by Miller (1986)". (Williams, and Kuniansky, 2016). "the **carbonate rocks** of the Upper Floridan aquifer may directly overlie clastic rocks of the Lisbon and Claiborne aquifers;" (Williams, and Kuniansky, 2016). "Over a large part of the Floridan aquifer system, the top is marked **by Oligocene rocks (Suwannee Limestone or equivalent)** where such rocks are **permeable and in hydraulic connection with the main part of the system**." (Williams and Kuniansky, 2016). "the **Upper Floridan** aquifer consists mostly of **Oligocene Suwannee Limestone (if present) and late-Eocene Ocala Limestone**." (Williams and Kuniansky, 2016).
Ocala-Avon Park lower permeability zone *(layers of semiconfined unit exists)*	Sedimentary **aquitard** (consolidated or semi-consolidated rock)	"The aggregate Avon Park permeable zone is overlain everywhere by a **less-permeable carbonate zone** named by others the "**Ocala-Avon Park lower permeability zone**" (**a lower permeability zone within the Upper Floridan aquifer**) and underlain by lower permeability confining to semiconfining evaporitic and non-evaporitic rocks of the newly

Formation name	Category	Quote
		mapped middle Avon Park composite unit (previously all or parts of MCUI–III and VI).” (Williams and Kuniansky, 2016).
Aggregate Avon Park permeable zone *(Upper dolomite sand unit is leaky aquifer)*	Carbonate aquifer	“Within the **Upper Floridan aquifer** of central and southern Florida, a subregionally e**xtensive, highly fractured and cavernous interval** called the **Avon Park permeable zone** is mapped as an aggregate of **several permeable zones in the upper part of the Avon Park Formation**.” (Williams and Kuniansky, 2016).
Middle Avon Park composite unit (semiconfining)	Sedimentary **aquitard** (consolidated or semi-consolidated rock)	“The aggregate Avon Park permeable zone is overlain everywhere by a less-permeable carbonate zone named by others the “Ocala-Avon Park lower permeability zone” (a lower permeability zone within the Upper Floridan aquifer) and underlain by **lower permeability confining to semiconfining evaporitic and non-evaporitic rocks** of the newly mapped **middle Avon Park composite unit** (**previously all or parts of MCUI–III and VI**).” (Williams and Kuniansky, 2016).
Lower Avon Park permeable zone *(this aquifer unit has thin layer of semiconfining unit).*	Carbonate aquifer	“Below the MAPCU, **permeable zones** in the lower Avon Park Formation form the **lower Avon Park permeable zone (LAPPZ)**. The LAPPZ is mapped as a relatively thick zone of **higher permeability with some lower permeability rocks** that lie between the MAPCU and the glauconite marker unit. **Freshwater parts of the LAPPZ are used for water supply in east-central Florida, where the zone is part of the Lower Floridan aquifer**.” (Williams and Kuniansky, 2016).
Glauconite marker unit	Sedimentary rock **aquitard** (consolidated or semi-consolidated rock)	“***Glauconite Marker Unit***: *Peninsular Florida*—Over much of peninsular Florida, a distinctive **lower permeability unit** lies deep within the **Floridan aquifer system near its base**.” (Williams and Kuniansky, 2016).

Williams, L.J., Kuniansky, E.L. (2016). Revised hydrogeologic framework of the Floridan aquifer system in Florida and parts of Georgia, Alabama, and South Carolina (ver. 1.1, March 2016): U.S. Geological Survey Professional Paper 1807, 140 p., 23 pls., http://dx.doi.org/10.3133/pp1807

3.14 Lower Coastal Plain, Floridan Aquifer System

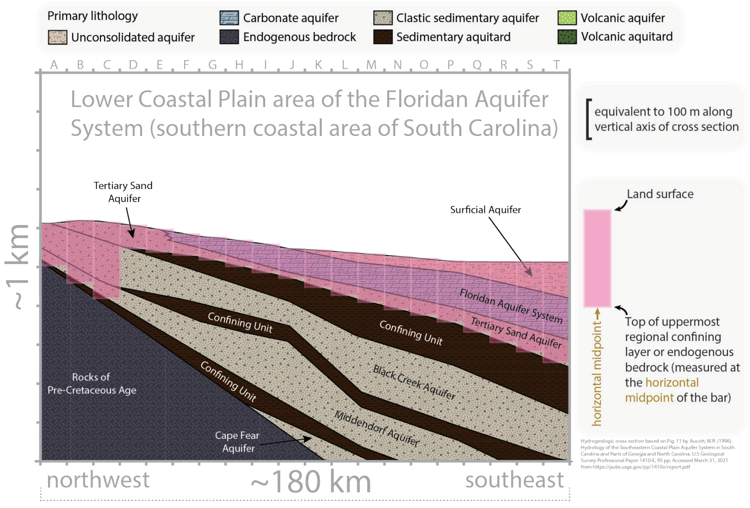

Supplementary Fig. 125. Hydrogeologic cross section. 20 equally spaced transparent pink bars overlie the cross section; each shaded bar depicts the vertical offset from the land surface to the top of the uppermost confining unit or endogenous bedrock.

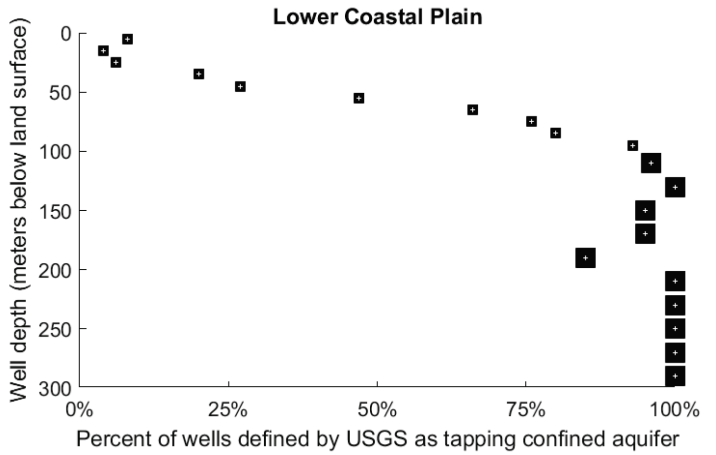

Supplementary Fig. 126. Vertical variations in the prevalence of wells that have been defined as tapping an unconfined or a confined aquifer by the USGS. The smaller squares represent 10 m depth intervals from the land surface to 100 m; the larger squares represent 20 m intervals from 100 m to 300 m below the land surface.

The Lower Coastal Plain is located in the southern coastal region of South Carolina.

(i) A hydrogeologic cross section presented in Fig. 11 by Aucott (1996) depicts a confining unit at depths of ~80 to ~150 m below the land surface.

(ii) We analysed wells within the study area that the USGS has defined as either unconfined or confined. Most (>80%) wells at depths of 90-100 m and at depths exceeding 90 m are defined as tapping a confined aquifer.

Reference: Aucott, W.R. (1996). Hydrology of the Southeastern Coastal Plain Aquifer System in South Carolina and Parts of Georgia and North Carolina. U.S Geological Survey Professional Paper 1410-E, 95 pp. Accessed March 31, 2021 from https://pubs.usgs.gov/pp/1410e/report.pdf

The table below presents a series of published quotes (see quotation marks denoting text quoted from another publication, which is cited following the quotation marks with the full reference written in full below the table). The leftmost column lists a title of a hydrogeologic formation depicted in the cross section on the previous page. The rightmost column presents a quote from a hydrogeological study (see base of table for citation). The quote has been annotated with colored text to highlight how we categorized each layer (i.e., see categories in the center column in the table). Specifically: (i) blue text highlights portions of a quote that provide insights into the degree of consolidation of the formation, (ii) red text highlights portions of a quote that categorize the formation as an aquifer or an aquitard (i.e., higher versus lower permeability in the context of local hydrogeologic formations), and (iii) green text highlights portions of a quote that provide information about the lithology of the formation.

Supplementary Table 17. Hydrostratigraphy details for the Lower Coastal Plain

Formation name	Category	Quote
Surficial aquifer	Unconsolidated aquifer	"The **surficial aquifer** consists of **marine terrace deposits**. These sediments are generally less than 40 ft thick and **consist primarily of sand, shell, and clay that were deposited during a series of transgressions and regressions of the sea** during the Pleistocene Epoch (Siple, 1946)." (Aucott, 1999).
Floridan aquifer system	Carbonate aquifer	"The **Floridan aquifer system** in South Carolina generally consists of **white to creamy-yellow limestone** of late to middle Eocene age. The sediments composing this system are parts of **the Cooper Group, the Ocala Limestone (where present), and the underlying Santee Limestone** (table 1)." (Aucott, 1999).
Tertiary Sand aquifer	Sedimentary **aquifer** (consolidated or semi-consolidated rock)	"The **Tertiary sand aquifer** partly underlies the carbonate rocks composing the Floridan aquifer system and partly is the clastic facies equivalent of the carbonate rocks." (Aucott, 1999). "**Sediments** composing this aquifer include the Barnwell, McBean, and Congaree Formations and the upper part of the Black Mingo Formation (table 1); they consist of **fine to medium sand and clay, commonly light greenish yellow to orange**." (Aucott, 1999).
Confining unit	Sedimentary **aquitard** (consolidated or semi-consolidated rock)	"Vertical movement across a **confining unit** is usually controlled by **the least permeable layer** within the confining unit, which is typically a tight, **marine clay** in the Coastal Plain sediments of South Carolina. All of the confining units identified **allow limited vertical movement of water through them**." (Aucott, 1999).
Black Creek aquifer	Sedimentary **aquifer** (consolidated or semi-consolidated rock)	"**Sediments** composing the Black Creek Formation are principally thin, **laminated layers** of **gray, fine to medium, micaceous sand and dark-gray to black clay**. The **coarseness of the sands and the clay content vary areally**. The **Black Creek aquifer** is the uppermost regional aquifer consisting of sediments of Cretaceous age." (Aucott, 1999).
Confining unit	Sedimentary **aquitard** (consolidated or semi-consolidated rock)	"The **confining unit** between the Black Creek aquifer and the Middendorf aquifer primarily consists of **sandy clay** in the lower part of the Black Creek Formation." (Aucott, 1999).
Middendorf aquifer	Sedimentary **aquifer** (consolidated or	"In the lower Coastal Plain, the sediments of the **Middendorf aquifer** are lithologically similar to those of the Black Creek

Formation name	Category	Quote
	semi-consolidated rock)	aquifer and consist of **thin, laminated layers of fine to medium sand and clay**." (Aucott, 1999).
Confining unit	Sedimentary **aquitard** (consolidated or semi-consolidated rock)	"The **confining unit** between the Middendorf and Cape Fear aquifers is **very effective in separating the flow systems of these aquifers** in the eastern part of the study area." (Aucott, 1999).
Cape Fear aquifer	Sedimentary **aquifer** (consolidated or semi-consolidated rock)	"The **Cape Fear aquifer** consists of the lower part of the Cape Fear Formation and is the **basal aquifer** in the Coastal Plain aquifer system of South Carolina." (Aucott, 1999). "The **Cape Fear aquifer** consists predominantly of **sand, silt, and gravel** separated by **relatively thick silt and clay layers**." (Aucott, 1999).
Rocks of pre-Cretaceous age	Endogenous bedrock (some of these rocks may be sedimentary in nature – see quote to right)	"Underlying the **sediments** that compose the Coastal Plain aquifer system are **pre-Cretaceous igneous, metamorphic, and consolidated sedimentary rocks**, all of **low permeability**. The **flow of water within these pre-Cretaceous rocks and between them** and the overlying Coastal Plain aquifers **is considerably less than** flow within the Coastal Plain aquifers." (Aucott, 1999).

Aucott, W.R. (1996). Hydrology of the Southeastern Coastal Plain Aquifer System in South Carolina and Parts of Georgia and North Carolina. U.S Geological Survey Professional Paper 1410-E, 95 pp. Accessed March 31, 2021 from https://pubs.usgs.gov/pp/1410e/report.pdf

3.15 Ocala Uplift, Floridan Aquifer System

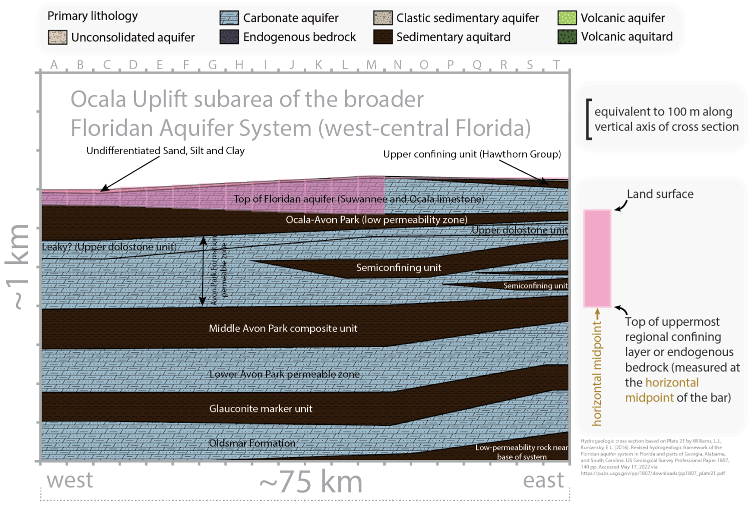

Supplementary Fig. 127. Hydrogeologic cross section. 20 equally spaced transparent pink bars overlie the cross section; each shaded bar depicts the vertical offset from the land surface to the top of the uppermost confining unit or endogenous bedrock.

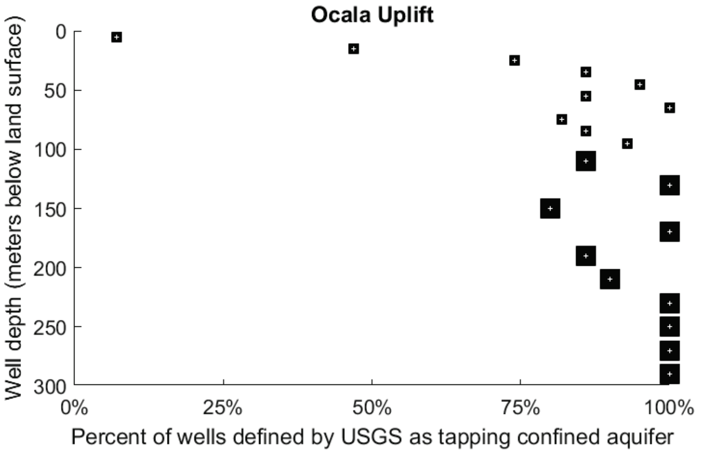

Supplementary Fig. 128. Vertical variations in the prevalence of wells that have been defined as tapping an unconfined or a confined aquifer by the USGS. The smaller squares represent 10 m depth intervals from the land surface to 100 m; the larger squares represent 20 m intervals from 100 m to 300 m below the land surface.

The Ocala Uplift portion of the Floridan Aquifer System is located in western Florida.

(i) A hydrogeologic cross section presented in Plate 2 by Williams and Kuniansky (2016) depicts the Suwannee and Ocala limestone units near the land surface but, in some places, it is overlain by a shallow confining unit (the Hawthorn Group).

(ii) We analysed wells within the study area that the USGS has defined as either unconfined or confined. Most (>80%) wells at depths of 30-40 m and at depths exceeding 30 m are defined as tapping a confined aquifer.

Depth to confined conditions: 30-40 meters below land surface (based on (ii) above)

Reference: Williams, L.J., Kuniansky, E.L. (2016). Revised hydrogeologic framework of the Floridan aquifer system in Florida and parts of Georgia, Alabama, and South Carolina. US Geological Survey Professional Paper 1807, 140 pp. Accessed March 31, 2021 from https://pubs.usgs.gov/pp/1807/pdf/pp1807.pdf

The table below presents a series of published quotes (see quotation marks denoting text quoted from another publication, which is cited following the quotation marks with the full reference written in full below the table). The leftmost column lists a title of a hydrogeologic formation depicted in the cross section on the previous page. The rightmost column presents a quote from a hydrogeological study (see base of table for citation). The quote has been annotated with colored text to highlight how we categorized each layer (i.e., see categories in the center column in the table). Specifically: (i) blue text highlights portions of a quote that provide insights into the degree of consolidation of the formation, (ii) red text highlights portions of a quote that categorize the formation as an aquifer or an aquitard (i.e., higher versus lower permeability in the context of local hydrogeologic formations), and (iii) green text highlights portions of a quote that provide information about the lithology of the formation.

Supplementary Table 18. Hydrostratigraphy details for the Ocala Uplift

**Formation name**	**Category**	**Quote**
Undifferentiated Sand, Silt and Clay	Unconsolidated aquifer	"The **surficial aquifer system** consists mostly of **sand and locally contains gravel and sandy lime stone** of Pliocene to Holocene age. Where these **sediments are thick and highly permeable,**" (Williams, and Kuniansky, 2016). "The surficial aquifer system forms **a thin irregular blanket of terrace and alluvial sands** that can act as an important source sink layer for temporary storage of groundwater that may **ultimately recharge the underlying Floridan aquifer system**. (Williams, and Kuniansky, 2016)
Upper confining unit (Hawthorn Group)	Sedimentary **aquitard** (consolidated or semi-consolidated rock)	"The **thickest and most extensive Miocene unit** in the study area is the **Hawthorn Group**. The lithology of the Hawthorn varies greatly between areas, but mostly consists of **phosphatic clay, silt, and sand that range in color from cream or gray to green to brown**." (Williams and Kuniansky, 2016). "The entire formation **forms a thick, generally clastic**, **highly variable sequence of lower permeability** rock that, where present, is **considered to be the upper confining unit** of **the Floridan aquifer system**." (Williams and Kuniansky, 2016).
Top of Floridan aquifer system (Suwannee and Ocala limestone)	Carbonate aquifer	"The Upper Floridan aquifer includes the **uppermost or shallowest permeable zones** in the Floridan aquifer system." (Williams, and Kuniansky, 2016) | "The Floridan aquifer system includes the vertically continuous **carbonate-rock system** described by Miller (1986)". (Williams, and Kuniansky, 2016). "the **carbonate rocks** of the Upper Floridan aquifer may directly overlie clastic rocks of the Lisbon and Claiborne aquifers;" (Williams, and Kuniansky, 2016). "Over a large part of the Floridan aquifer system, the top is marked **by Oligocene rocks (Suwannee Limestone or equivalent)** where such rocks are **permeable and in hydraulic connection with the main part of the system**." (Williams and Kuniansky, 2016). "the **Upper Floridan** aquifer consists mostly of **Oligocene Suwannee Limestone (if present) and late-Eocene Ocala Limestone**." (Williams and Kuniansky, 2016).
Ocala-Avon Park lower permeability zone *(layers of semiconfined unit exists)*	Sedimentary **aquitard** (consolidated or semi-consolidated rock)	"The aggregate Avon Park permeable zone is overlain everywhere by a **less-permeable carbonate zone** named by others the "**Ocala-Avon Park lower permeability zone**" (**a lower permeability zone within the Upper Floridan aquifer**) and underlain by lower permeability confining to semiconfining evaporitic and non-evaporitic rocks of the newly

		mapped middle Avon Park composite unit (previously all or parts of MCUI–III and VI).” (Williams and Kuniansky, 2016).
Aggregate Avon Park permeable zone *(Upper dolomite sand unit is leaky aquifer)*	Carbonate aquifer	“Within the **Upper Floridan aquifer** of central and southern Florida, a subregionally e**xtensive, highly fractured and cavernous interval** called the **Avon Park permeable zone** is mapped as an aggregate of **several permeable zones in the upper part of the Avon Park Formation**.” (Williams and Kuniansky, 2016).
Middle Avon park composite unit (semiconfining)	Sedimentary **aquitard** (consolidated or semi-consolidated rock)	“The aggregate Avon Park permeable zone is overlain everywhere by a less-permeable carbonate zone named by others the “Ocala-Avon Park lower permeability zone” (a lower permeability zone within the Upper Floridan aquifer) and underlain by **lower permeability confining to semiconfining evaporitic and non-evaporitic rocks** of the newly mapped **middle Avon Park composite unit** (**previously all or parts of MCUI–III and VI**).” (Williams and Kuniansky, 2016).
Lower Avon Park permeable zone	Carbonate aquifer	“Below the MAPCU, **permeable zones** in the lower Avon Park Formation form the **lower Avon Park permeable zone (LAPPZ)**. The LAPPZ is mapped as a relatively thick zone of **higher permeability with some lower permeability rocks** that lie between the MAPCU and the glauconite marker unit. **Freshwater parts of the LAPPZ are used for water supply in east-central Florida, where the zone is part of the Lower Floridan aquifer**.” (Williams and Kuniansky, 2016).
Glauconite marker unit	Sedimentary **aquitard** (consolidated or semi-consolidated rock)	“***Glauconite Marker Unit***: *Peninsular Florida*—Over much of peninsular Florida, a distinctive **lower permeability unit** lies deep within the **Floridan aquifer system near its base**.” (Williams and Kuniansky, 2016).
Oldsmar Formation	Carbonate aquifer	“Unit is in the **Oldsmar Formation** consisting almost entirely of **yellowish brown, well indurated, cryptocrystalline to microcrystalline dolostone**; in part **calcareous**, highly altered, vuggy with associated iron staining.” (Williams and Kuniansky, 2016). “The Oldsmar permeable zone includes all extremely **permeable zones and otherwise less permeable zones** that lie between the glauconite marker unit **and base of the massive dolostone unit in the Oldsmar Formation**.” (Williams and Kuniansky, 2016).
Low permeability rock near base of system	Sedimentary **aquitard** (consolidated or semi-consolidated rock)	“The Floridan is **underlain everywhere by low-permeability rocks** called **the lower confining unit**, which separates the Floridan aquifer system from older, deeper aquifers of the Southeastern Coastal Plain aquifer system.” (Williams and Kuniansky, 2016). “The **base of the Floridan aquifer system** is marked by the **lower confining unit**, consisting of **predominantly low-permeability late Paleocene to middle Eocene rocks**.” (Williams and Kuniansky, 2016).

Williams, L.J., and Kuniansky, E.L. (2016). Revised hydrogeologic framework of the Floridan aquifer system in Florida and parts of Georgia, Alabama, and South Carolina (ver. 1.1, March 2016): U.S. Geological Survey Professional Paper 1807, 140 p., 23 pls., http://dx.doi.org/10.3133/pp1807

3.16 Sea Island, Floridan Aquifer System

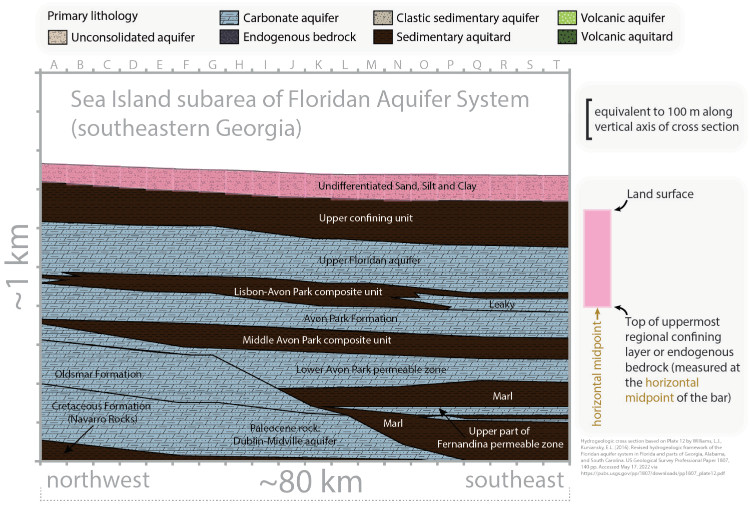

Supplementary Fig. 129. Hydrogeologic cross section. 20 equally spaced transparent pink bars overlie the cross section; each shaded bar depicts the vertical offset from the land surface to the top of the uppermost confining unit or endogenous bedrock.

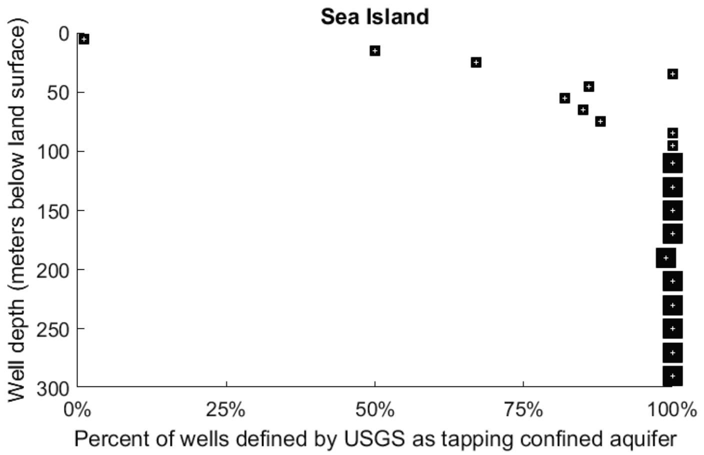

Supplementary Fig. 130. Vertical variations in the prevalence of wells that have been defined as tapping an unconfined or a confined aquifer by the USGS. The smaller squares represent 10 m depth intervals from the land surface to 100 m; the larger squares represent 20 m intervals from 100 m to 300 m below the land surface.

The Sea Island area of the Floridan Aquifer System is located in eastern Georgia.

(i) A hydrogeologic cross section presented in Plate 1 by Williams and Kuniansky (2016) depicts a shallow layer of undifferentiated sand, silt and clay underlain by a confining unit.

(ii) We analysed wells within the study area that the USGS has defined as either unconfined or confined. Most (>80%) wells at depths of 30-40 m and at depths exceeding 30 m are defined as tapping a confined aquifer.

Depth to confined conditions: 30-40 meters below land surface (based on (ii) above)

Reference: Williams, L.J., Kuniansky, E.L. (2016). Revised hydrogeologic framework of the Floridan aquifer system in Florida and parts of Georgia, Alabama, and South Carolina. US Geological Survey Professional Paper 1807, 140 pp. Accessed March 31, 2021 from https://pubs.usgs.gov/pp/1807/pdf/pp1807.pdf

The table below presents a series of published quotes (see quotation marks denoting text quoted from another publication, which is cited following the quotation marks with the full reference written in full below the table). The leftmost column lists a title of a hydrogeologic formation depicted in the cross section on the previous page. The rightmost column presents a quote from a hydrogeological study (see base of table for citation). The quote has been annotated with colored text to highlight how we categorized each layer (i.e., see categories in the center column in the table). Specifically: (i) blue text highlights portions of a quote that provide insights into the degree of consolidation of the formation, (ii) red text highlights portions of a quote that categorize the formation as an aquifer or an aquitard (i.e., higher versus lower permeability in the context of local hydrogeologic formations), and (iii) green text highlights portions of a quote that provide information about the lithology of the formation.

Supplementary Table 19. Hydrostratigraphy details for the Sea Island

Formation name	Category	Quote
Undifferentiated Sand, Silt and clay	Unconsolidated aquifer	"The **surficial aquifer system** consists mostly of **sand and locally contains gravel and sandy lime stone** of Pliocene to Holocene age. Where these **sediments are thick and highly permeable,**" (Williams, and Kuniansky, 2016). "The surficial aquifer system forms **a thin irregular blanket of terrace and alluvial sands** that can act as an important source sink layer for temporary storage of groundwater that may **ultimately recharge the underlying Floridan aquifer system**. (Williams, and Kuniansky, 2016)
Upper confining unit	Sedimentary **aquitard** (consolidated or semi-consolidated rock)	"The **thickest and most extensive Miocene unit** in the study area is the **Hawthorn Group**. The lithology of the Hawthorn varies greatly between areas, but mostly consists of **phosphatic clay, silt, and sand that range in color from cream or gray to green to brown**." (Williams and Kuniansky, 2016). "The entire formation **forms a thick, generally clastic**, **highly variable sequence of lower permeability** rock that, where present, is **considered to be the upper confining unit** of **the Floridan aquifer system**." (Williams and Kuniansky, 2016).
Upper Floridan aquifer	Carbonate aquifer	"The Upper Floridan aquifer includes the **uppermost or shallowest permeable zones** in the Floridan aquifer system." (Williams, and Kuniansky, 2016) | "The Floridan aquifer system includes the vertically continuous **carbonate-rock system** described by Miller (1986)". (Williams, and Kuniansky, 2016). "the **carbonate rocks** of the Upper Floridan aquifer may directly overlie clastic rocks of the Lisbon and Claiborne aquifers;" (Williams, and Kuniansky, 2016).
Lisbon-Avon Park composite unit *(A leaky to non-leaky(?), semiconfining unit locally gypsiferous limestone, dolomitic limestone, & dolostone in the middle part of the Avon Park Formation)*	Sedimentary **aquitard** (consolidated or semi-consolidated rock)	"As shown in figure 6, the Upper and Lower Floridan aquifers are separated by the **discontinuous numbered middle confining units** that are now grouped within two composite units: (1) **the Lisbon-Avon Park composite unit** (LISAPCU), consisting **of lower permeability clastic and higher to lower permeability carbonate rocks in** the northern part of the study area; and (2) **the middle Avon Park composite unit** (MAPCU), consisting of **lower permeability evaporite- and non-evaporite-bearing carbonate rocks to moderately permeable carbonate rocks** in the central and southern parts of the study area." (Williams and Kuniansky, 2016).

Formation name	Category	Quote
Avon Park Formation	Carbonate aquifer	"In the northern coastal areas of Georgia and South Carolina, the strata that compose the **Lower Floridan aquifer** include **limestone, dolomitic limestone, and dolomite** that lie within the **middle to lower part of the Avon Park Formation** or equivalent middle Eocene formations (pl. 2)." (Williams and Kuniansky, 2016).
Middle Avon Park composite unit	Sedimentary **aquitard** (consolidated or semi-consolidated rock)	"**Middle Avon Park Composite Unit**, *Central and Southern Florida*—The MAPCU consists of **lower permeability rocks** in both **evaporitic and non-evaporitic** facies within the middle (or approximate middle) part of the Avon Park Formation (table 8, figs. 37 and 38). Because **its permeability is generally lower than that of other less-permeable zones within the Floridan aquifer system**, the MAPCU is considered **the principal confining to semiconfining unit** in peninsular Florida (fig. 38)." (Williams and Kuniansky, 2016).
Lower Avon Park permeable zone	Carbonate aquifer	"Below the MAPCU, **permeable zones** in the lower Avon Park Formation form the **lower Avon Park permeable zone (LAPPZ)**. The LAPPZ is mapped as a relatively thick zone of **higher permeability with some lower permeability rocks** that lie between the MAPCU and the glauconite marker unit. **Freshwater parts of the LAPPZ are used for water supply in east-central Florida, where the zone is part of the Lower Floridan aquifer**." (Williams and Kuniansky, 2016).
Marl	Sedimentary **aquitard** (consolidated or semi-consolidated rock)	"In the northern coastal region of Georgia and South Carolina, the **base of the Floridan aquifer system** is formed by **low-permeability middle Eocene marl** (fig. 23)." (Williams and Kuniansky, 2016).
Oldsmar Formation	Carbonate aquifer	"Unit is in the **Oldsmar Formation** consisting almost entirely of **yellowish brown, well indurated, cryptocrystalline to microcrystalline dolostone**; in part **calcareous**, highly altered, vuggy with associated iron staining." (Williams and Kuniansky, 2016). "The Oldsmar permeable zone includes all extremely **permeable zones and otherwise less permeable zones** that lie between the glauconite marker unit **and base of the massive dolostone unit in the Oldsmar Formation**." (Williams and Kuniansky, 2016).
Upper Part of Fernandina permeable zone	Carbonate aquifer	"For example, the **Fernandina permeable zone** (Miller, 1986) is a cavernous interval within the **Lower Floridan aquifer** that **has much higher hydraulic conductivity than the surrounding rock**." (Williams and Kuniansky, 2016).
Paleocene rock: Dublin-Midville aquifer	Carbonate aquifer	"**Paleocene rocks** in the study area can be categorized into three groups. The first group, which is the most important to this study, primarily consists of **interbedded dolomite and anhydrite** of the **Cedar Keys Formation**, which underlies part of southeastern Georgia and all of peninsular Florida." (Williams and Kuniansky, 2016). "**Interbedded dolomite and anhydrite, both being of lower permeability**, compose the lower two-thirds of the Cedar Keys Formation of peninsular Florida and southeastern Georgia. This **carbonate-evaporite sequence forms the base of the Floridan aquifer system**." (Williams and Kuniansky, 2016).

Formation name	Category	Quote
Cretaceous Formation (Navarro rocks)	Sedimentary **aquitard** (consolidated or semi-consolidated rock)	"South of Brunswick, Ga., the base of the system drops several hundred feet into **late Cretaceous rocks** consisting of **soft, friable, possibly Tayloran age, limestone with the permeable, late Cretaceous, Navarroan age Lawson Limestone above included as part of the Lower Floridan aquifer** (Miller, 1986)." (Williams and Kuniansky, 2016). Figure 2 of (Williams and Kuniansky, 2016) under "Hydrogeology" in "Florida" "**Confining unit**". (William, Raines, Lanning, 2016) under the "Hydrogeology" in "Florida" "**confining unit**".

Williams, L.J., and Kuniansky, E.L. (2016). Revised hydrogeologic framework of the Floridan aquifer system in Florida and parts of Georgia, Alabama, and South Carolina (ver. 1.1, March 2016): U.S. Geological Survey Professional Paper 1807, 140 p., 23 pls., http://dx.doi.org/10.3133/pp1807

3.17 Tifton Upland, Floridan Aquifer System

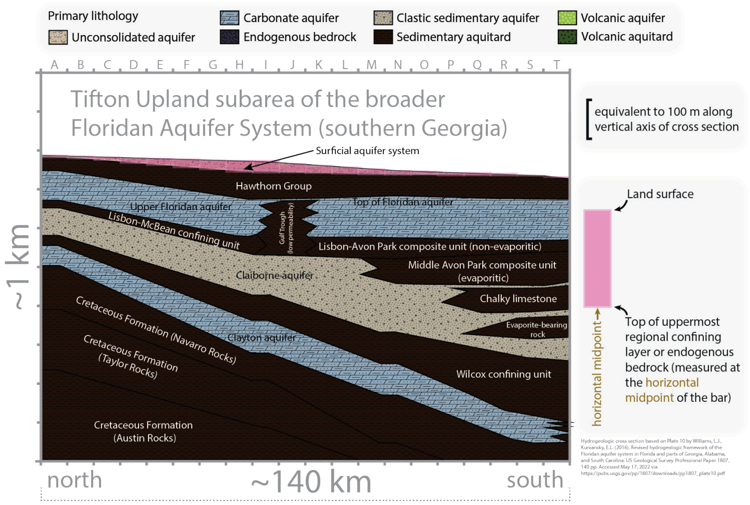

Supplementary Fig. 131. Hydrogeologic cross section. 20 equally spaced transparent pink bars overlie the cross section; each shaded bar depicts the vertical offset from the land surface to the top of the uppermost confining unit or endogenous bedrock.

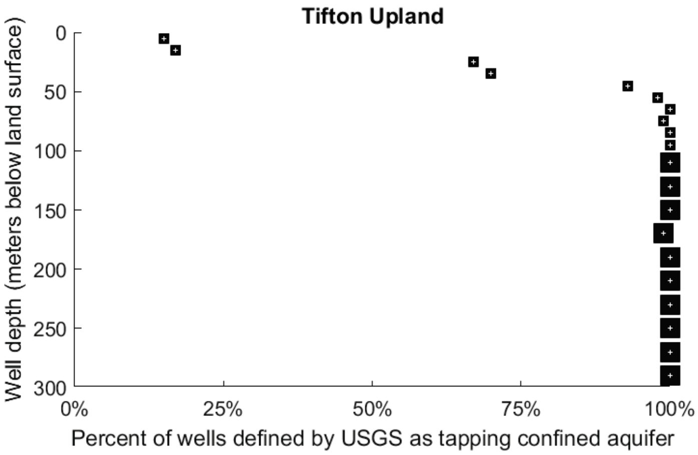

Supplementary Fig. 132. Vertical variations in the prevalence of wells that have been defined as tapping an unconfined or a confined aquifer by the USGS. The smaller squares represent 10 m depth intervals from the land surface to 100 m; the larger squares represent 20 m intervals from 100 m to 300 m below the land surface.

The Tifton Upland portion of the Floridan Aquifer System is located in south-central Georgia.

(i) A hydrogeologic cross section presented in Plate 8 by Williams and Kuniansky (2016) shows the Surficial Aquifer underlain by a relatively shallow confining unit (Hawthorn Group).

(ii) We analysed wells within the study area that the USGS has defined as either unconfined or confined. Most (>80%) wells at depths of 40-50 m and at depths exceeding 40 m are defined as tapping a confined aquifer.

Depth to confined conditions: 40-50 meters below land surface (based on (ii) above)

Reference: Williams, L.J., Kuniansky, E.L. (2016). Revised hydrogeologic framework of the Floridan aquifer system in Florida and parts of Georgia, Alabama, and South Carolina. US Geological Survey Professional Paper 1807, 140 pp. Accessed March 31, 2021 from https://pubs.usgs.gov/pp/1807/pdf/pp1807.pdf

The table below presents a series of published quotes (see quotation marks denoting text quoted from another publication, which is cited following the quotation marks with the full reference written in full below the table). The leftmost column lists a title of a hydrogeologic formation depicted in the cross section on the previous page. The rightmost column presents a quote from a hydrogeological study (see base of table for citation). The quote has been annotated with colored text to highlight how we categorized each layer (i.e., see categories in the center column in the table). Specifically: (i) blue text highlights portions of a quote that provide insights into the degree of consolidation of the formation, (ii) red text highlights portions of a quote that categorize the formation as an aquifer or an aquitard (i.e., higher versus lower permeability in the context of local hydrogeologic formations), and (iii) green text highlights portions of a quote that provide information about the lithology of the formation.

Supplementary Table 20. Hydrostratigraphy details for the Tifton Upland

Formation name	Category	Quote
Surficial aquifer system	Unconsolidated aquifer	"The **surficial aquifer system** consists mostly of **sand and locally contains gravel and sandy lime stone** of Pliocene to Holocene age. Where these **sediments are thick and highly permeable,**" (Williams, and Kuniansky, 2016). "The surficial aquifer system forms **a thin irregular blanket of terrace and alluvial sands** that can act as an important source sink layer for temporary storage of groundwater that may **ultimately recharge the underlying Floridan aquifer system**. (Williams, and Kuniansky, 2016)
Hawthorn Group	Sedimentary **aquitard** (consolidated or semi-consolidated rock)	"The **thickest and most extensive Miocene unit** in the study area is the **Hawthorn Group**. The lithology of the Hawthorn varies greatly between areas, but mostly consists of **phosphatic clay, silt, and sand that range in color from cream or gray to green to brown**." (Williams and Kuniansky, 2016). "The entire formation **forms a thick, generally clastic**, **highly variable sequence of lower permeability** rock that, where present, is **considered to be the upper confining unit** of **the Floridan aquifer system**." (Williams and Kuniansky, 2016).
Upper Floridan aquifer	Carbonate aquifer	"The Upper Floridan aquifer includes the **uppermost or shallowest permeable zones** in the Floridan aquifer system." (Williams, and Kuniansky, 2016) | "The Floridan aquifer system includes the vertically continuous **carbonate-rock system** described by Miller (1986)". (Williams, and Kuniansky, 2016). "the **carbonate rocks** of the Upper Floridan aquifer may directly overlie clastic rocks of the Lisbon and Claiborne aquifers;" (Williams, and Kuniansky, 2016). "In Georgia, the **Gulf Trough forms a narrow, distinct structural low at the top of the Floridan aquifer system** (figs. 10 and 22). (Williams and Kuniansky, 2016).
Lisbon-McBean confining	Sedimentary **aquitard** (consolidated or semi-consolidated rock	"The composite unit also has been identified by Faye and Mayer (1997) as the **Lisbon-McBean confining unit**." (Williams and Kuniansky, 2016). Table 7 of (Williams and Kuniansky, 2016), under the "Equivalent hydrogeologic unit": "Lisbon-McBean confining unit" and "Lithology" as "**Clay, sand, argillaceous limestone**", under the "Water-bearing properites" "**Mostly confining**, may vary laterally depend on lithology". (quoting Williams and Kuniansky, 2016)
Claiborne aquifer	Sedimentary **aquifer** (consolidated or semi-	"In this area, and farther north toward the outcrop area, **clastic rocks** of the **Claiborne aquifer** (McFadden and Perriello, 1983) are **hydraulically connected to the Upper Floridan aquifer**." (Williams and Kuniansky, 2016). "**Sands of the Tallahatta and**

Formation name	Category	Quote
	consolidated rock)	**Huber Formations were included in the Gordon aquifer system** (Brooks and others, 1985) and **equivalent beds** in southwestern Georgia were called the **Claiborne aquifer** (McFadden and Perriello, 1983). Like the **Lisbon aquifer, the Gordon and Claiborne aquifers** are considered part of the **Floridan aquifer system** in this report." (Williams and Kuniansky, 2016). "In Georgia, the **Gordon aquifer** consists of middle Eocene **interbedded sand, silt, and clay** and is equivalent to the Hatchetigbee and Tallahatta Formations and the lower part of the Lisbon Formation in western Georgia (Brooks and others, 1985)." (Williams and Kuniansky, 2016).
Lisbon-Avon Park composite unit (non-evaporitic), Middle Avon Park composite unit *(some can be moderately permeable).*	Sedimentary **aquitard** (consolidated or semi-consolidated rock)	"As shown in figure 6, the Upper and Lower Floridan aquifers are separated by the **discontinuous numbered middle confining units** that are now grouped within two composite units: (1) **the Lisbon-Avon Park composite unit** (LISAPCU), consisting **of lower permeability clastic and higher to lower permeability carbonate rocks in** the northern part of the study area; and (2) **the middle Avon Park composite unit** (MAPCU), consisting of **lower permeability evaporite-and non-evaporite-bearing carbonate rocks to moderately permeable carbonate rocks** in the central and southern parts of the study area." (Williams and Kuniansky, 2016).
Wilcox confining unit	Sedimentary **aquitard** (consolidated or semi-consolidated rock)	"In Georgia, the base is identified as the **Wilcox confining zone**, which separates the overlying **Lower Floridan and Claiborne aquifers** from the underlying Clayton aquifer (Clarke and others, 1984)." (Williams and Kuniansky, 2016). "The Floridan is **underlain everywhere by low-permeability rocks** called **the lower confining unit**, which separates the Floridan aquifer system from older, deeper aquifers of the Southeastern Coastal Plain aquifer system." (Williams and Kuniansky, 2016). "The **base of the Floridan aquifer system** is marked by the **lower confining unit**, consisting of **predominantly low-permeability late Paleocene to middle Eocene rocks**." (Williams and Kuniansky, 2016).
Clayton aquifer *(sometimes, sand can dominate the limestone presence)*	Carbonate aquifer	"The **limestone** of **the Clayton aquifer** is not hydraulically connected to other Tertiary limestones of the Floridan aquifer system." (Williams and Kuniansky, 2016). "In western Georgia, the uppermost part of the Clayton is a **hard, sandy, fossiliferous limestone** that constitutes **an important aquifer** (Clarke and others, 1984)." (Williams and Kuniansky, 2016).
Cretaceous formation	Sedimentary rock **aquitard** (consolidated or semi-consolidated rock)	"South of Brunswick, Ga., the base of the system drops several hundred feet into **late Cretaceous rocks** consisting of **soft, friable, possibly Tayloran age, limestone with the permeable, late Cretaceous, Navarroan age Lawson Limestone above included as part of the Lower Floridan aquifer** (Miller, 1986)." (Williams and Kuniansky, 2016). Figure 2 of (Williams and Kuniansky, 2016) under "Hydrogeology" in "Florida" "**Confining unit**". (William, Raines, Lanning, 2016) under the "Hydrogeology" in "Florida" "**confining unit**".

Williams, L.J., and Kuniansky, E.L. (2016). Revised hydrogeologic framework of the Floridan aquifer system in Florida and parts of Georgia, Alabama, and South Carolina (ver. 1.1, March 2016): U.S. Geological Survey Professional Paper 1807, 140 p., 23 pls., http://dx.doi.org/10.3133/pp1807

3.18 Vidalia Upland, Floridan Aquifer System

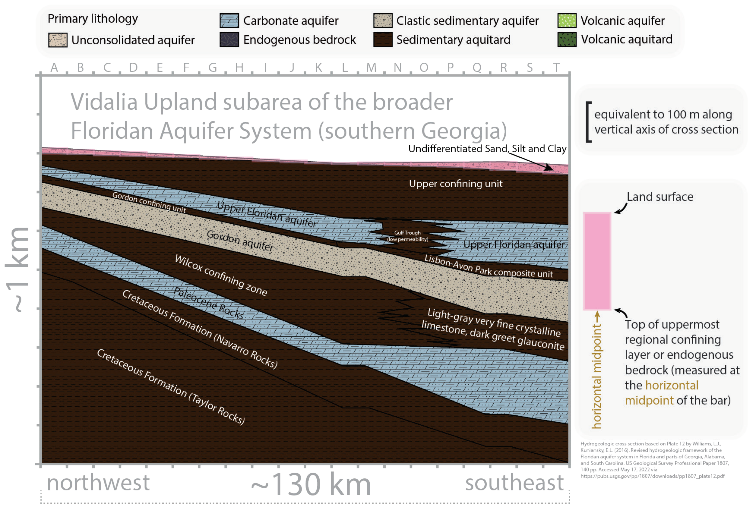

Supplementary Fig. 133. Hydrogeologic cross section. 20 equally spaced transparent pink bars overlie the cross section; each shaded bar depicts the vertical offset from the land surface to the top of the uppermost confining unit or endogenous bedrock.

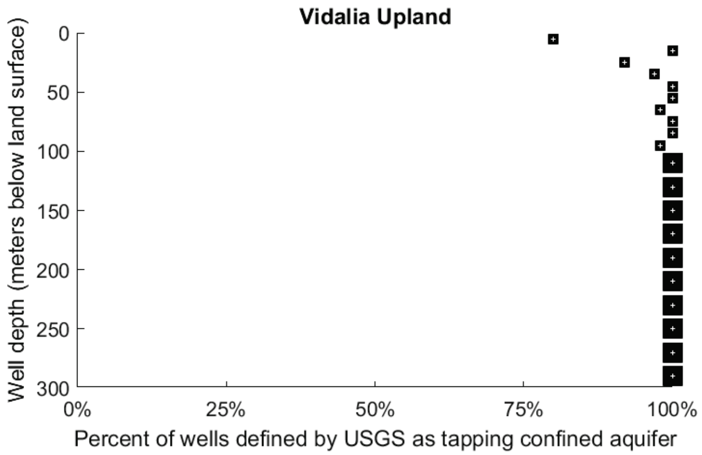

Supplementary Fig. 134. Vertical variations in the prevalence of wells that have been defined as tapping an unconfined or a confined aquifer by the USGS. The smaller squares represent 10 m depth intervals from the land surface to 100 m; the larger squares represent 20 m intervals from 100 m to 300 m below the land surface.

The Vidalia Upland area of the Floridan Aquifer System is located in central and eastern Georgia.

(i) A hydrogeologic cross section presented in Plate 8 by Williams and Kuniansky (2016) depicts undifferentiated sand, silt and clay (less than ~20 m thick) underlain by the Hawthorn Group confining unit.

(ii) We analysed wells within the study area that the USGS has defined as either unconfined or confined. Most (>80%) wells at depths of 10-20 m and at depths exceeding 10 m are defined as tapping a confined aquifer.

Depth to confined conditions: 10-20 meters below land surface (based on (ii) above)

Reference: Williams, L.J., Kuniansky, E.L. (2016). Revised hydrogeologic framework of the Floridan aquifer system in Florida and parts of Georgia, Alabama, and South Carolina. US Geological Survey Professional Paper 1807, 140 pp. Accessed March 31, 2021 from https://pubs.usgs.gov/pp/1807/pdf/pp1807.pdf

The table below presents a series of published quotes (see quotation marks denoting text quoted from another publication, which is cited following the quotation marks with the full reference written in full below the table). The leftmost column lists a title of a hydrogeologic formation depicted in the cross section on the previous page. The rightmost column presents a quote from a hydrogeological study (see base of table for citation). The quote has been annotated with colored text to highlight how we categorized each layer (i.e., see categories in the center column in the table). Specifically: (i) blue text highlights portions of a quote that provide insights into the degree of consolidation of the formation, (ii) red text highlights portions of a quote that categorize the formation as an aquifer or an aquitard (i.e., higher versus lower permeability in the context of local hydrogeologic formations), and (iii) green text highlights portions of a quote that provide information about the lithology of the formation.

Supplementary Table 21. Hydrostratigraphy details for the Vidalia Upland

Formation name	Category	Quote
Undifferentiated Sand, Silt and clay	Unconsolidated aquifer	"The **surficial aquifer system** consists mostly of **sand and locally contains gravel and sandy lime stone** of Pliocene to Holocene age. Where these **sediments are thick and highly permeable,**" (Williams, and Kuniansky, 2016). "The surficial aquifer system forms **a thin irregular blanket of terrace and alluvial sands** that can act as an important source sink layer for temporary storage of groundwater that may **ultimately recharge the underlying Floridan aquifer system**. (Williams, and Kuniansky, 2016)
Upper confining unit	Sedimentary **aquitard** (consolidated or semi-consolidated rock)	"The **thickest and most extensive Miocene unit** in the study area is the **Hawthorn Group**. The lithology of the Hawthorn varies greatly between areas, but mostly consists of **phosphatic clay, silt, and sand that range in color from cream or gray to green to brown**." (Williams and Kuniansky, 2016). "The entire formation **forms a thick, generally clastic**, **highly variable sequence of lower permeability** rock that, where present, is **considered to be the upper confining unit** of **the Floridan aquifer system**." (Williams and Kuniansky, 2016).
Upper Floridan aquifer	Carbonate aquifer	"The Upper Floridan aquifer includes the **uppermost or shallowest permeable zones** in the Floridan aquifer system." (Williams, and Kuniansky, 2016) | "The Floridan aquifer system includes the vertically continuous **carbonate-rock system** described by Miller (1986)". (Williams, and Kuniansky, 2016). "the **carbonate rocks** of the Upper Floridan aquifer may directly overlie clastic rocks of the Lisbon and Claiborne aquifers;" (Williams, and Kuniansky, 2016). "In Georgia, the **Gulf Trough forms a narrow, distinct structural low at the top of the Floridan aquifer system** (figs. 10 and 22). (Williams and Kuniansky, 2016).
Gordon confining unit	Sedimentary **aquitard** (consolidated or semi-consolidated rock)	"The Gordon aquifer is overlain and **confined** by the late-middle Eocene **Lisbon-McBean confining unit** and is equivalent to the Lisbon Formation in western and central Georgia, where it generally consists of **massive glauconitic marl interbedded with calcareous clayey sand and fossiliferous limestone** over most of its extent (Brooks and others, 1985)." (Williams and Kuniansky, 2016).
Lisbon-Avon Park composite unit	Sedimentary **aquitard** (consolidated or	"As shown in figure 6, the Upper and Lower Floridan aquifers are separated by the **discontinuous numbered middle confining units** that are now grouped within two composite units: (1) **the Lisbon-Avon Park composite unit** (LISAPCU),

Formation name	Category	Quote
	semi-consolidated rock)	consisting **of lower permeability clastic and higher to lower permeability carbonate rocks in** the northern part of the study area; and (2) **the middle Avon Park composite unit** (MAPCU), consisting of **lower permeability evaporite- and non-evaporite-bearing carbonate rocks to moderately permeable carbonate rocks** in the central and southern parts of the study area." (Williams and Kuniansky, 2016).
Gordon aquifer	Sedimentary **aquifer** (consolidated or semi-consolidated rock)	"In Georgia, the **Gordon aquifer** consists of middle Eocene **interbedded sand, silt, and clay** and is equivalent to the Hatchetigbee and Tallahatta Formations and the lower part of the Lisbon Formation in western Georgia (Brooks and others, 1985)." (Williams and Kuniansky, 2016).
Wilcox confining zone	Sedimentary **aquitard** (consolidated or semi-consolidated rock)	"In Georgia, the base is identified as the **Wilcox confining zone**, which separates the overlying **Lower Floridan and Claiborne aquifers** from the underlying Clayton aquifer (Clarke and others, 1984)." (Williams and Kuniansky, 2016). "The Floridan is **underlain everywhere by low-permeability rocks** called **the lower confining unit**, which separates the Floridan aquifer system from older, deeper aquifers of the Southeastern Coastal Plain aquifer system." (Williams and Kuniansky, 2016). "The **base of the Floridan aquifer system** is marked by the **lower confining unit**, consisting of **predominantly low-permeability late Paleocene to middle Eocene rocks**." (Williams and Kuniansky, 2016).
Light-gray very fine crystalline limestone, dark greet glauconite	Sedimentary **aquitard** (consolidated or semi-consolidated rock)	"***Glauconite Marker Unit***: *Peninsular Florida*—Over much of peninsular Florida, a distinctive **lower permeability unit** lies deep within the **Floridan aquifer system near its base**." (Williams and Kuniansky, 2016).
Paleocene rocks	Carbonate aquifer	"**Paleocene rocks** in the study area can be categorized into three groups. The first group, which is the most important to this study, primarily consists of **interbedded dolomite and anhydrite** of the **Cedar Keys Formation**, which underlies part of southeastern Georgia and all of peninsular Florida." (Williams and Kuniansky, 2016). "**Interbedded dolomite and anhydrite, both being of lower permeability**, compose the lower two-thirds of the Cedar Keys Formation of peninsular Florida and southeastern Georgia. This **carbonate-evaporite sequence forms the base of the Floridan aquifer system**." (Williams and Kuniansky, 2016).
Cretaceous Formation (Navarro and Taylor rocks)	Sedimentary rock **aquitard** (consolidated or semi-consolidated rock)	"South of Brunswick, Ga., the base of the system drops several hundred feet into **late Cretaceous rocks** consisting of **soft, friable, possibly Tayloran age, limestone with the permeable, late Cretaceous, Navarroan age Lawson Limestone above included as part of the Lower Floridan aquifer** (Miller, 1986)." (Williams and Kuniansky, 2016). Figure 2 of (Williams and Kuniansky, 2016) under "Hydrogeology" in "Florida" "**Confining unit**". (William, Raines, Lanning, 2016) under the "Hydrogeology" in "Florida" "**confining unit**".

Williams, L.J., and Kuniansky, E.L. (2016). Revised hydrogeologic framework of the Floridan aquifer system in Florida and parts of Georgia, Alabama, and South Carolina (ver. 1.1, March 2016): U.S. Geological Survey Professional Paper 1807, 140 p., 23 pls., http://dx.doi.org/10.3133/pp1807

3.19 Catahoula Area, Gulf Coast Regional Aquifer System

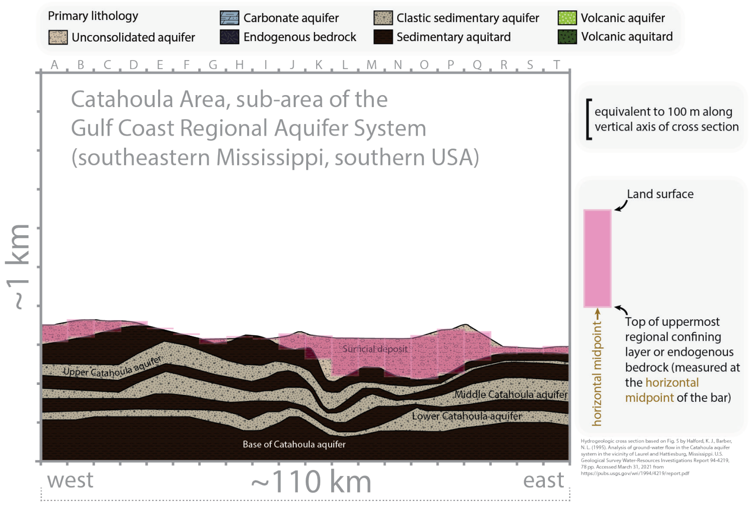

Supplementary Fig. 135. Hydrogeologic cross section. 20 equally spaced transparent pink bars overlie the cross section; each shaded bar depicts the vertical offset from the land surface to the top of the uppermost confining unit or endogenous bedrock.

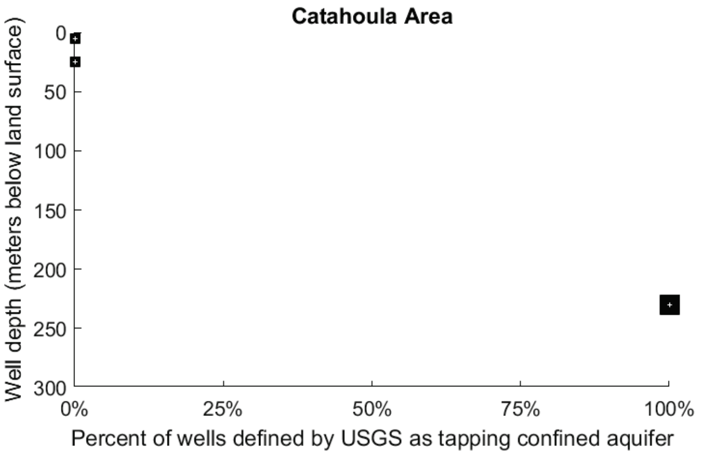

Supplementary Fig. 136. Vertical variations in the prevalence of wells that have been defined as tapping an unconfined or a confined aquifer by the USGS. The smaller squares represent 10 m depth intervals from the land surface to 100 m; the larger squares represent 20 m intervals from 100 m to 300 m below the land surface.

The Catahoula Area of the broader Gulf Coast Regional Aquifer System is located in southeastern Mississippi.

(i) A hydrogeologic cross section (based on Fig. 5 by Halford and Barber, 1995) suggests that the median depth to the uppermost sedimentary aquitard is 40-50 meters below land surface (see pink transparent bars in cross section to the left).

(ii) We analysed wells within the study area that the USGS has defined as either unconfined or confined. The available USGS well data (n=3 wells) are insufficient to evaluate the depths at which the aquifer system transitions from unconfined to confined conditions.

(iii) In a regional-scale hydrogeologic study, Halford and Barber (1995) comment on the presence of confined conditions in the Catahoula formations; they state (quote): *"The confining units overlying the upper, middle, and lower Catahoula aquifers are primarily clays within the Catahoula, Pascagoula, and Hattiesburg Formations."*

Depth to confined conditions: 40-50 m (see (i) above)

Reference: Halford, K. J., Barber, N. L. (1995). U.S. Geological Survey Water-Resources Investigations Report 94-4219, 78 pp. Accessed March-2021 from https://pubs.usgs.gov/wri/1994/4219/report.pdf

The table below presents a series of published quotes (see quotation marks denoting text quoted from another publication, which is cited following the quotation marks with the full reference written in full below the table). The leftmost column lists a title of a hydrogeologic formation depicted in the cross section on the previous page. The rightmost column presents a quote from a hydrogeological study (see base of table for citation). The quote has been annotated with colored text to highlight how we categorized each layer (i.e., see categories in the center column in the table). Specifically: (i) blue text highlights portions of a quote that provide insights into the degree of consolidation of the formation, (ii) red text highlights portions of a quote that categorize the formation as an aquifer or an aquitard (i.e., higher versus lower permeability in the context of local hydrogeologic formations), and (iii) green text highlights portions of a quote that provide information about the lithology of the formation.

Supplementary Table 22. Hydrostratigraphy details for Catahoula Area

Formation name	Category	Quote
Land surface (surficial deposit)	Unconsolidated aquifer *(In some area this aquifer is confined aquifer)*	"Geologic units at land surface in the study area are **sediments of Miocene** age consisting of a complex series of alternating and **lenticular beds of sand and clay, and other sediments** of Pliocene and younger age." (Halford & Barber, 1995). "Thin surficial deposits of sand and gravel of the Citronelle Formation overlie the Pascagoula and Hattiesburg Formations in the immediate vicinity of the dome (fig. 23). **Freshwater sands of the Pascagoula and Hattiesburg overlie the Catahoula Sandstone**, the basal unit of the sedimentary beds that extend across the dome." (Spiers & Gandl, 1980).
Confining layer	Sedimentary **aquitard** (consolidated or semi-consolidated rock)	"The **confining units overlying** the upper, middle, and lower Catahoula aquifers are primarily **clays** within the Catahoula, Pascagoula, and Hattiesburg Formations. These **clays vary in thickness from nearly zero to several hundred feet** (figs. 9-11)." (Halford and Barber, 1995)
Upper Catahoula aquifer	Sedimentary **aquifer** (consolidated or semi-consolidated rock)	"The **principal sources of ground water** in the Laurel and Hattiesburg areas are **sands** within the Catahoula Formation which compose the Catahoula aquifer system. Generally the Catahoula Formation **contains a thick water-bearing sand** near the base of the formation and two other sands higher in the sequence; however the sands generally cannot be correlated regionally. Between and within the sands are units of clay that vary in thickness and areal extent. Following Boswell and others (1987, p. 18), **the sands** in this report are referred to as **the lower, middle, and upper Catahoula aquifers**." (Halford and Barber, 1995)
Confining layer	Sedimentary **aquitard** (consolidated or semi-consolidated rock)	"The **confining units overlying** the upper, middle, and lower Catahoula aquifers are primarily **clays** within the Catahoula, Pascagoula, and Hattiesburg Formations. These **clays vary in thickness from nearly zero to several hundred feet** (figs. 9-11)." (Halford and Barber, 1995)
Middle Catahoula aquifer	Sedimentary **aquifer** (consolidated or semi-consolidated rock)	"The **principal sources of ground water** in the Laurel and Hattiesburg areas are **sands** within the Catahoula Formation which compose the Catahoula aquifer system. Generally the Catahoula Formation **contains a thick water-bearing sand** near the base of the formation and two other sands higher in the sequence; however the sands generally cannot be correlated regionally. Between and within the sands are units of clay that vary in thickness and areal extent. Following Boswell and others (1987, p. 18), **the sands** in this report are

Formation name	Category	Quote
		referred to as **the lower, middle, and upper Catahoula aquifers**." (Halford and Barber, 1995)
Confining layer	Sedimentary **aquitard** (consolidated or semi-consolidated rock)	"The **confining units overlying** the upper, middle, and lower Catahoula aquifers are primarily **clays** within the Catahoula, Pascagoula, and Hattiesburg Formations. These **clays vary in thickness from nearly zero to several hundred feet** (figs. 9-11)." (Halford and Barber, 1995)
Lower Catahoula aquifer	Sedimentary **aquifer** (consolidated or semi-consolidated rock)	"The **principal sources of ground water** in the Laurel and Hattiesburg areas are **sands** within the Catahoula Formation which compose the Catahoula aquifer system. Generally the Catahoula Formation **contains a thick water-bearing sand** near the base of the formation and two other sands higher in the sequence; however the sands generally cannot be correlated regionally. Between and within the sands are units of clay that vary in thickness and areal extent. Following Boswell and others (1987, p. 18), **the sands** in this report are referred to as **the lower, middle, and upper Catahoula aquifers**." (Halford and Barber, 1995)
Base of Catahoula aquifer	Sedimentary **aquitard** (consolidated or semi-consolidated rock)	"The base of the lower Catahoula aquifer system in the study area was identified using borehole geophysical logs and the Glendon Formation, a **highly resistive limestone** unit within the Vicksburg Group, as the primary **marker bed**." (Halford and Barber, 1995)

Halford, K. J., Barber, N. L. (1995). Analysis of ground-water flow in the Catahoula aquifer system in the vicinity of Laurel and Hattiesburg, Mississippi (Vol. 94, No. 4219). US Department of the Interior, US Geological Survey. Accessed August 8, 2022 via https://pubs.usgs.gov/wri/1994/4219/report.pdf

Spiers, C. A., Gandl, L. A. (1980). *A preliminary report of the geohydrology of the Mississippi salt-dome basin* (Vol. 80, No. 595). US Geological Survey. https://pubs.er.usgs.gov/publication/ofr80595

3.20 Houston-Galveston Area, Gulf Coast Regional Aquifer System

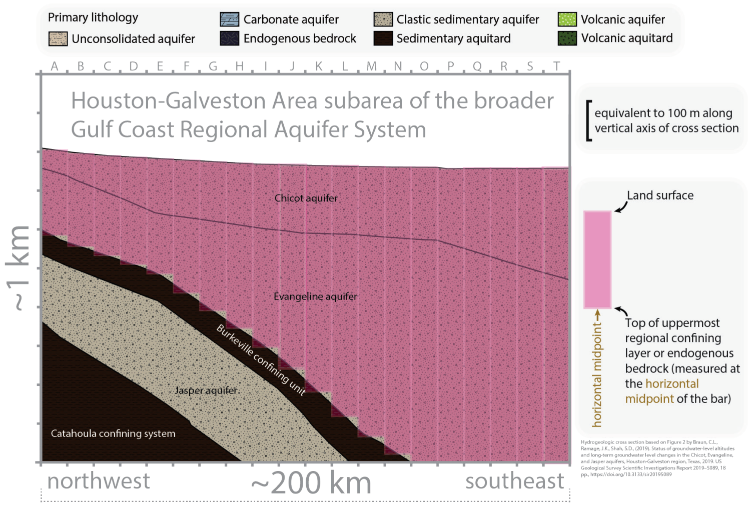

Supplementary Fig. 137. Hydrogeologic cross section. 20 equally spaced transparent pink bars overlie the cross section; each shaded bar depicts the vertical offset from the land surface to the top of the uppermost confining unit or endogenous bedrock.

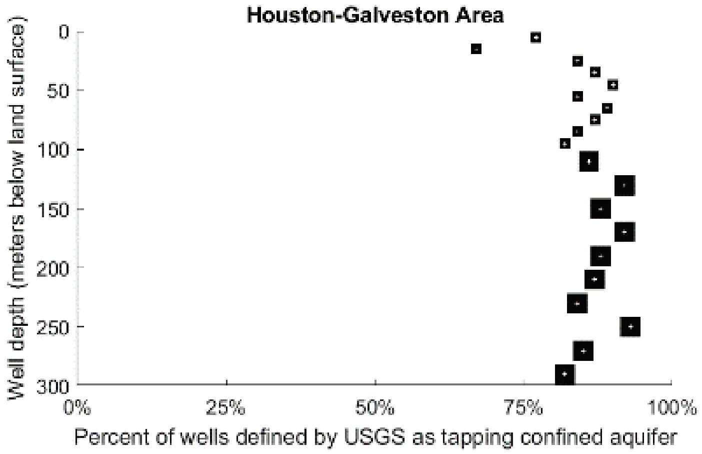

Supplementary Fig. 138. Vertical variations in the prevalence of wells that have been defined as tapping an unconfined or a confined aquifer by the USGS. The smaller squares represent 10 m depth intervals from the land surface to 100 m; the larger squares represent 20 m intervals from 100 m to 300 m below the land surface.

The Houston-Galveston area is located near the central coast of Texas.

(i) A hydrogeologic cross section presented Fig. 2 by Braun et al. (2019) does not depict a clear confining unit within the aquifer system. However, the uppermost Chicot formation includes low-permeability units that together serve as confining units, leading to confined conditions in some wells screened within the Chicot Aquifer (see (ii) below).

(ii) We analysed wells within the study area that the USGS has defined as either unconfined or confined. Most (>80%) wells at depths of 20-30 m and at depths exceeding 20 m are defined as tapping a confined aquifer.

Depth to confined conditions: 20-30 m (see (ii) above)

Reference: Braun, C.L., Ramage, J.K., Shah, S.D., (2019). Status of groundwater-level altitudes and long-term groundwater level changes in the Chicot, Evangeline, and Jasper aquifers, Houston-Galveston region, Texas, 2019. US Geological Survey Scientific Investigations Report 2019–5089, 18 pp., https://doi.org/10.3133/sir20195089

The table below presents a series of published quotes (see quotation marks denoting text quoted from another publication, which is cited following the quotation marks with the full reference written in full below the table). The leftmost column lists a title of a hydrogeologic formation depicted in the cross section on the previous page. The rightmost column presents a quote from a hydrogeological study (see base of table for citation). The quote has been annotated with colored text to highlight how we categorized each layer (i.e., see categories in the center column in the table). Specifically: (i) blue text highlights portions of a quote that provide insights into the degree of consolidation of the formation, (ii) red text highlights portions of a quote that categorize the formation as an aquifer or an aquitard (i.e., higher versus lower permeability in the context of local hydrogeologic formations), and (iii) green text highlights portions of a quote that provide information about the lithology of the formation.

Supplementary Table 23. Hydrostratigraphy details for the Houston Galveston Area

Formation name	Category	Quote
Chicot aquifer	Sedimentary **aquifer** (consolidated or semi-consolidated rock)	"It consists of several **aquifers**, including the Jasper, Evangeline, and **Chicot aquifers**, which are composed of **discontinuous sand, silt, clay, and gravel beds of Miocene to Holocene age** (Figure 6-17)." (Bruun et al., 2017).
Evangeline aquifer	Sedimentary **aquifer** (consolidated or semi-consolidated rock)	"It consists of several **aquifers**, including the Jasper, **Evangeline**, and Chicot **aquifers**, which are composed of **discontinuous sand, silt, clay, and gravel beds of Miocene to Holocene age** (Figure 6-17)." (Bruun et al., 2017).
Burkeville confining unit	Sedimentary **aquitard** (consolidated or semi-consolidated rock)	"The Oligocene Catahoula tuff forms a leaky confining layer at the base of the aquifer, and the **Burkeville confining unit** separates the Jasper Aquifer from the Evangeline Aquifer. All of the **sedimentary units** thicken toward the Gulf of Mexico." (Bruun et al., 2017). "The updated **Burkeville Confining Unit** consists primarily of the **clay-rich deposits comprising the Upper, Middle, and Lower Lagarto formations** delineated by Young and others (2010, 2012)." (Young, 2020)
Jasper aquifer	Sedimentary **aquifer** (consolidated or semi-consolidated rock)	"It consists of several **aquifers**, including the **Jasper**, Evangeline, and Chicot **aquifers**, which are composed of **discontinuous sand, silt, clay, and gravel beds of Miocene to Holocene age** (Figure 6-17)." (Bruun et al., 2017).
Catahoula confining system	Sedimentary **aquitard** (consolidated or semi-consolidated rock)	"The Oligocene Catahoula **tuff** forms **a leaky confining layer** at the base of the aquifer, and the Burkeville confining unit separates the Jasper Aquifer from the Evangeline Aquifer. All of the **sedimentary units** thicken toward the Gulf of Mexico." (Bruun et al., 2017). "one for each of the hydrogeologic units of the aquifer system except the **Catahoula confining system, the assumed no-flow base of the system.**" Kasmarek (2012)

Bruun, B., Jackson, K., Lake, P., Walker, J. (2016). Texas Aquifers Study. Texas Water Development Board Report. 336 pp. Accessed June 14, 2022 from https://www.twdb.texas.gov/groundwater/docs/studies/TexasAquifersStudy_2016.pdf#page=89

Young C.S. (2020). The delineation of the Burkeville confining unit and the base of the Chicot aquifer to support the development of the Gulf 2023 groundwater model. INTERA Incorporated. Accessed June 14, 2022 from https://hgsubsidence.org/wp-content/uploads/2021/06/Final_HGSD_FBSD_Burkeville_Report_final.pdf

Kasmarek, M.C. (2012). Hydrogeology and simulation of groundwater flow and land-surface subsidence in the northern part of the Gulf Coast aquifer system, Texas, 1891–2009 (ver. 1.1, November 2013): U.S. Geological Survey Scientific Investigations Report 2012–5154, 55 p., http://pubs.usgs.gov/sir/2012/5154/. Accessed June 14, 2022 from https://www.twdb.texas.gov/groundwater/models/gam/glfc_n/HAGM.SIR.Version1.1.November2013.pdf

Braun, C.L., Ramage, J.K., Shah, S.D. (2019). Status of groundwater-level altitudes and long-term groundwaterlevel changes in the Chicot, Evangeline, and Jasper aquifers, Houston-Galveston region, Texas, 2019: U.S. Geological Survey Scientific Investigations Report 2019–5089, 18 p., https://doi.org/10.3133/sir20195089

3.21 Lafayette Area, Gulf Coast Regional Aquifer System

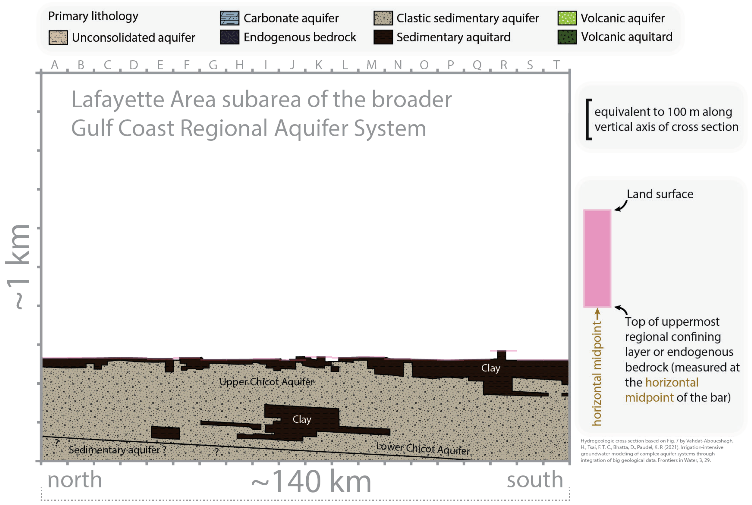

Supplementary Fig. 139. Hydrogeologic cross section. 20 equally spaced transparent pink bars overlie the cross section; each shaded bar depicts the vertical offset from the land surface to the top of the uppermost confining unit or endogenous bedrock.

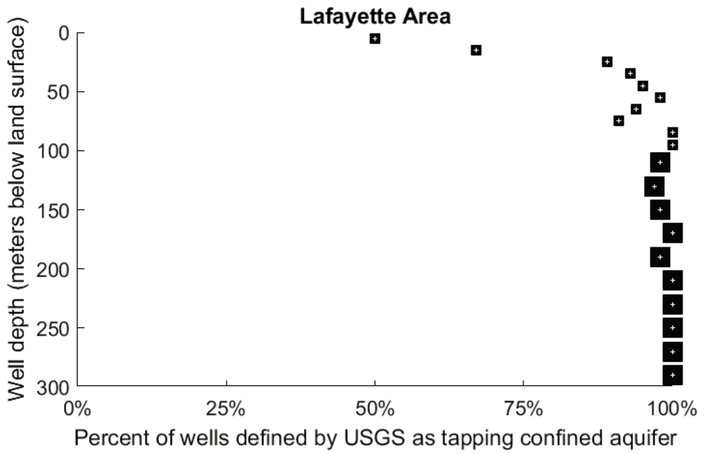

Supplementary Fig. 140. Vertical variations in the prevalence of wells that have been defined as tapping an unconfined or a confined aquifer by the USGS. The smaller squares represent 10 m depth intervals from the land surface to 100 m; the larger squares represent 20 m intervals from 100 m to 300 m below the land surface.

The Lafayette Area is located in southwestern Louisiana.

(i) A hydrogeologic cross section presented in Fig. 7 by Vahdat-Aboueshagh et al. (2021) suggests that a low permeability clay-rich unit exists near to the land surface in the study area.

(ii) We analysed wells within the study area that the USGS has defined as either unconfined or confined. Most (>80%) wells at depths of 20-30 m and at depths exceeding 20 m are defined as tapping a confined aquifer.

Depth to confined conditions: 20-30 m (see (ii) above)

Reference: Vahdat-Aboueshagh, H., Tsai, F. T. C., Bhatta, D., Paudel, K. P. (2021). Irrigation-intensive groundwater modeling of complex aquifer systems through integration of big geological data. Frontiers in Water, 3, 623476.

The table below presents a series of published quotes (see quotation marks denoting text quoted from another publication, which is cited following the quotation marks with the full reference written in full below the table). The leftmost column lists a title of a hydrogeologic formation depicted in the cross section on the previous page. The rightmost column presents a quote from a hydrogeological study (see base of table for citation). The quote has been annotated with colored text to highlight how we categorized each layer (i.e., see categories in the center column in the table). Specifically: (i) blue text highlights portions of a quote that provide insights into the degree of consolidation of the formation, (ii) red text highlights portions of a quote that categorize the formation as an aquifer or an aquitard (i.e., higher versus lower permeability in the context of local hydrogeologic formations), and (iii) green text highlights portions of a quote that provide information about the lithology of the formation.

Supplementary Table 24. Hydrostratigraphy details for the Lafayette Area

Formation name	Category	Quote
Confining clay units	Sedimentary **aquitard** (consolidated or semi-consolidated rock)	"The Pleistocene Upper Chicot and Lower Chicot aquifers are separated by **clay beds as thick as 30m (100 ft) in a few areas**. The **clay beds** are not extensive (Williams and Duex, 1995). In general, sands in the north are relatively shallow and often **interbedded with clays**." (Vahdat-Aboueshagh et al., 2021).
Upper Chicot aquifer	Sedimentary **aquifer** (consolidated or semi-consolidated rock)	"The **Chicot aquifer system** encompasses seven aquifers (Nyman et al., 1990): **the Upper Chicot** and the Lower Chicot **aquifers** in the east, the Undifferentiated sand in the central area, and the Shallow sand, the "200-foot" sand, the "500-foot" sand, and the "700-foot" sand in the west." (Vahdat-Aboueshagh et al., 2021). "The Chicot is comprised of a series of **unconsolidated sands, gravels, silts and clays** of Holocene through Pliocene-age that were generated by the ancestral Mississippi River to the east and the smaller Sabine and Calcasieu Rivers to the west (Prakken, 2003, Tollett et al., 2003, Lovelace et al., 2004; Fig. 1)." (Borrok et al., 2016).
Lower Chicot aquifer	Sedimentary **aquifer** (consolidated or semi-consolidated rock)	"The **Chicot aquifer system** encompasses seven aquifers (Nyman et al., 1990): the Upper Chicot and **the Lower Chicot aquifers** in the east, the Undifferentiated sand in the central area, and the Shallow sand, the "200-foot" sand, the "500-foot" sand, and the "700-foot" sand in the west." (Vahdat-Aboueshagh et al., 2021). "The Chicot is comprised of a series of **unconsolidated sands, gravels, silts and clays** of Holocene through Pliocene-age that were generated by the ancestral Mississippi River to the east and the smaller Sabine and Calcasieu Rivers to the west (Prakken, 2003, Tollett et al., 2003, Lovelace et al., 2004; Fig. 1)." (Borrok et al., 2016).
Sedimentary aquifer (maybe the Evangeline aquifer)	Sedimentary **aquifer** (consolidated or semi-consolidated rock)	"Pumping at a rate that is greater than can be supported by the Chicot in this region likely resulted in the capture of Na–HCO_3-rich waters from the underlying Evangeline aquifer." (Borrok et al., 2016). "The Chicot, **Evangeline**, and Jasper equivalent **aquifer systems** extend across most of southeastern

Formation name	Category	Quote
		Louisiana and generally consist of **silt, sand, and gravel separated by discontinuous layers of clay and sandy clay**." USGS (2017).

Vahdat-Aboueshagh, H., Tsai, F. T. C., Bhatta, D., Paudel, K. P. (2021). Irrigation-Intensive groundwater modeling of complex aquifer systems through integration of big geological data. *Frontiers in Water*, **3,** 623476.

Borrok, D. M., Broussard III, W. P. (2016). Long-term geochemical evaluation of the coastal Chicot aquifer system, Louisiana, USA. *Journal of Hydrology*, *533*, 320-331.

USGS (2017). Water Resources of the Southern Hills Regional Aquifer System, Southeastern Louisiana. US Geological Survey Fact Sheet Fact Sheet 2017–3010, 6 pp. Accessed April 12, 2022 via https://pubs.usgs.gov/fs/2017/3010/fs20173010.pdf

3.22 Southern Hills, Gulf Coast Regional Aquifer System

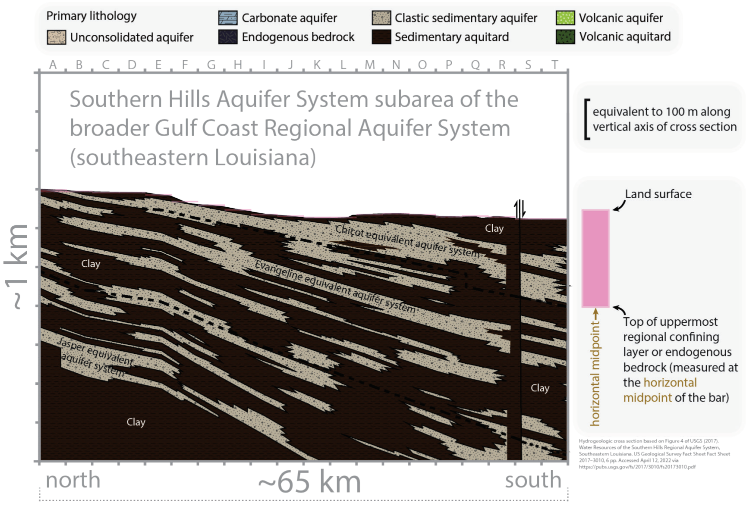

Supplementary Fig. 141. Hydrogeologic cross section. 20 equally spaced transparent pink bars overlie the cross section; each shaded bar depicts the vertical offset from the land surface to the top of the uppermost confining unit or endogenous bedrock.

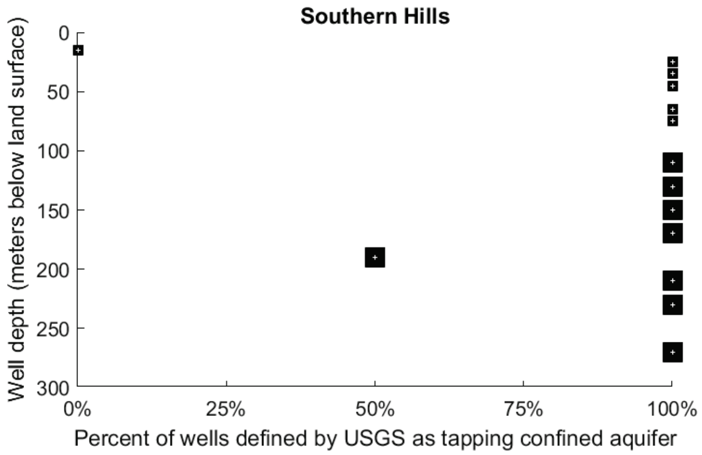

Supplementary Fig. 142. Vertical variations in the prevalence of wells that have been defined as tapping an unconfined or a confined aquifer by the USGS. The smaller squares represent 10 m depth intervals from the land surface to 100 m; the larger squares represent 20 m intervals from 100 m to 300 m below the land surface.

The Southern Hills subarea of the broader Gulf Coast Regional Aquifer System is located in southeastern Louisiana.

(i) A hydrogeologic cross section presented in Fig. 4 by USGS (2017) depicts a series of interbedded aquifers and low-permeability units.

(ii) We analysed wells within the study area that the USGS has defined as either unconfined or confined. Most (>80%) wells at depths of 20-30 m and at depths exceeding 20 m are defined as tapping a confined aquifer.

Depth to confined conditions: 20-30 m (see (ii) above)

Reference: USGS (2017). Water Resources of the Southern Hills Regional Aquifer System, Southeastern Louisiana. US Geological Survey Fact Sheet Fact Sheet 2017–3010, 6 pp. Accessed April 12, 2022 via https://pubs.usgs.gov/fs/2017/3010/fs20173010.pdf

The table below presents a series of published quotes (see quotation marks denoting text quoted from another publication, which is cited following the quotation marks with the full reference written in full below the table). The leftmost column lists a title of a hydrogeologic formation depicted in the cross section on the previous page. The rightmost column presents a quote from a hydrogeological study (see base of table for citation). The quote has been annotated with colored text to highlight how we categorized each layer (i.e., see categories in the center column in the table). Specifically: (i) blue text highlights portions of a quote that provide insights into the degree of consolidation of the formation, (ii) red text highlights portions of a quote that categorize the formation as an aquifer or an aquitard (i.e., higher versus lower permeability in the context of local hydrogeologic formations), and (iii) green text highlights portions of a quote that provide information about the lithology of the formation.

Supplementary Table 25. Hydrostratigraphy details for the Southern Hills aquifer

Formation name	Category	Quote
Chicot equivalent aquifer system	Sedimentary **aquifer** (consolidated or semi-consolidated rock)	"The **Chicot**, Evangeline, and Jasper equivalent **aquifer systems** extend across most of southeastern Louisiana and generally consist of **silt, sand, and gravel separated by discontinuous layers of clay and sandy clay**." USGS (2017). "" USGS (2017). "**The primary aquifers composing the Chicot equivalent aquifer system** in the western part of the 10-parish area, from shallowest to deepest**, are the shallow sands, Upland terrace aquifer, and the "400-foot" and "600-foot" sands** of the Baton Rouge area (Griffith, 2003; fig. 4)." USGS (2017).
Evangeline equivalent aquifer system	Sedimentary **aquifer** (consolidated or semi-consolidated rock)	"The Chicot, **Evangeline**, and Jasper equivalent **aquifer systems** extend across most of southeastern Louisiana and generally consist of **silt, sand, and gravel separated by discontinuous layers of clay and sandy clay**." USGS (2017). "**The primary aquifers composing the Evangeline equivalent aquifer system**, from shallowest to deepest, in the western part of the 10-parish area are **the "800-foot," "1,000-foot," "1,200-foot," "1,500-foot," and "1,700-foot" sands** of the Baton Rouge area (Griffith, 2003; fig. 4)." USGS (2017).
Jasper equivalent aquifer system	Sedimentary **aquifer** (consolidated or semi-consolidated rock)	"The Chicot, Evangeline, and **Jasper** equivalent **aquifer systems** extend across most of southeastern Louisiana and generally consist of **silt, sand, and gravel separated by discontinuous layers of clay and sandy clay**." USGS (2017). "" USGS (2017). "The **primary aquifers composing the Jasper equivalent aquifer system**, from shallowest to deepest, in the western part of the 10-parish area are the **"2,000-foot," "2,400-foot," and "2,800-foot" sands** of the Baton Rouge area (Griffith, 2003; fig. 4)." USGS (2017).

USGS (2017). Water Resources of the Southern Hills Regional Aquifer System, Southeastern Louisiana. US Geological Survey Fact Sheet Fact Sheet 2017–3010, 6 pp. Accessed April 12, 2022 via https://pubs.usgs.gov/fs/2017/3010/fs20173010.pdf

3.23 Central High Plains, High Plains

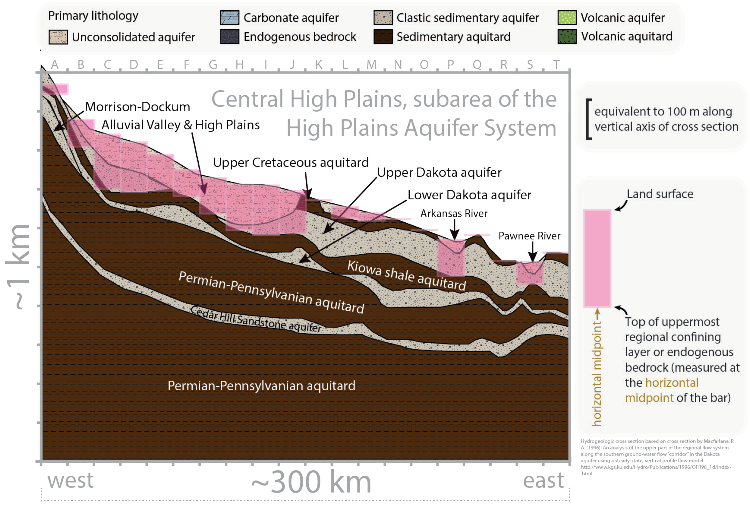

Supplementary Fig. 143. Hydrogeologic cross section. 20 equally spaced transparent pink bars overlie the cross section; each shaded bar depicts the vertical offset from the land surface to the top of the uppermost confining unit or endogenous bedrock.

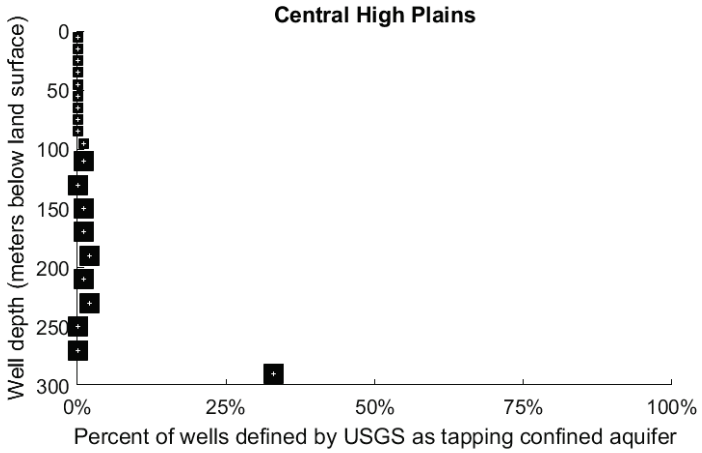

Supplementary Fig. 144. Vertical variations in the prevalence of wells that have been defined as tapping an unconfined or a confined aquifer by the USGS. The smaller squares represent 10 m depth intervals from the land surface to 100 m; the larger squares represent 20 m intervals from 100 m to 300 m below the land surface.

The Central High Plains aquifer system is located in northwest Texas, southwest Kansas, western Oklahoma, northwest New Mexico, and southeast Colorado.

(i) A hydrogeologic cross section presented by Macfarlane (1996) suggests that the uppermost confining unit is 43 meters below the land surface (25th-75th percentile range: 0-97 meters below land surface).

(ii) We analysed wells within the study area that the USGS has defined as either unconfined or confined. Nearly all wells with depths of less than 300 m are defined as tapping unconfined aquifers. All wells (n=3) at depths of 300-340 m are defined as tapping a confined aquifer (Dakota Sandstone aquifer).

Depth to confined conditions: 300-340 m (see (ii) above)

References: Macfarlane, P. A. (1996). An analysis of the upper part of the regional flow system along the southern ground-water flow “corridor” in the Dakota aquifer using a steady-state, vertical profile flow model. http://www.kgs.ku.edu/Hydro/Publications/1996/OFR96_1d/index.html

The table below presents a series of published quotes (see quotation marks denoting text quoted from another publication, which is cited following the quotation marks with the full reference written in full below the table). The leftmost column lists a title of a hydrogeologic formation depicted in the cross section on the previous page. The rightmost column presents a quote from a hydrogeological study (see base of table for citation). The quote has been annotated with colored text to highlight how we categorized each layer (i.e., see categories in the center column in the table). Specifically: (i) blue text highlights portions of a quote that provide insights into the degree of consolidation of the formation, (ii) red text highlights portions of a quote that categorize the formation as an aquifer or an aquitard (i.e., higher versus lower permeability in the context of local hydrogeologic formations), and (iii) green text highlights portions of a quote that provide information about the lithology of the formation.

Supplementary Table 26. Hydrostratigraphy details for the Central High Plains

**Formation name**	**Category**	**Quote**
Alluvial Valley & High Plains aquifer	Unconsolidated aquifer	"**Unconsolidated alluvial and eolian deposits**" (Macfarlane 1996) | "Alluvial Valley & High Plains **aquifer**" (Macfarlane 1996)
Upper Cretaceous aquitard	Sedimentary **aquitard** (consolidated or semi-consolidated rock)	**"Pierre Shale, Niobrara Chalk, Carlile Shale, Greenhorn Limestone, Graneros Shale"** Macfarlane, P. A. (1996) **| "Upper Cretaceous aquitard"** (Macfarlane 1996)
Upper Dakota aquifer	Clastic sedimentary **aquifer** (consolidated or semi-consolidated rock)	"Buff to light-brown, fine to medium grained **sandstone with interbedded shale**." (Luckey and Becker, 1999) | "**Upper Dakota aquifer**" (Macfarlane 1996)
Kiowa shale aquitard	Sedimentary **aquitard** (consolidated or semi-consolidated rock)	"**Gray to black shale** with some **fine-grained sandstone in upper part**." (Luckey and Becker, 1999) | "Kiowa shale **aquitard**" (Macfarlane 1996)
Lower Dakota aquifer	Clastic sedimentary **aquifer** (consolidated or semi-consolidated rock)	"Longford member" Macfarlane, P. A. (1996) "Cheyenne **Sandstone**" Macfarlane, P. A. (1996) | "**White to buff, fine to medium-grained sandstone** with some **interbedded shales**. Unit contains some conglomerate in lower part." (Luckey and Becker, 1999) I "Lower Dakota **aquifer**" (Macfarlane 1996)
Morrison-Dockum aquifer	Clastic sedimentary **aquifer** (consolidated or semi-consolidated rock)	"**Varicolored shale, sandstone, limestone, dolostone, and conglomerate**." (Luckey and Becker, 1999) "The Dockum Group is composed of **sandstone** with **interbedded shales grading upward** to a **shaly sandstone or siltstone"** (Luckey and Becker, 1999) "The Dakota Sandstone, the Lytle Sandstone, and the Dockum Group **all provide sufficient water** for stock and domestic use and **may provide sufficient water for irrigation, particularly when combined with the High Plains aquifer or with each other**." (Luckey and Becker, 1999). "Morrison-Dockum **aquifer**" (Macfarlane 1996)
Permian Aquitard	Sedimentary **aquitard** (consolidated or semi-consolidated rock)	"Undifferentiated Permian" (Macfarlane 1996) "Undifferentiated **red beds**" (Luckey and Becker, 1999) "**Predominately red or orange, shale, mudstone, siltstone, sandstone, dolostone, and anhydrite with some gypsum, limestone and halite**." (Luckey and Becker, 1999) | "Permian **Aquitard**" (Macfarlane 1996)

Formation name	Category	Quote
Cedar Hills Sandstone	Clastic sedimentary **aquifer** (consolidated or semi-consolidated rock)	"Cedar Hill **Sandstone**" Macfarlane, P. A. (1996)<br>"Cedar Hills **Sandstone** **aquifer**" Macfarlane, P. A. (1996)

Luckey, R.L., Becker, M.F. (1999). Hydrogeology, water use, and simulation of flow in the High Plains aquifer in northwestern Oklahoma, southeastern Colorado, southwestern Kansas, northeastern New Mexico, and northwestern Texas. US Geological Survey Water-Resources Investigations Report 99-4104, 73 pp. Accessed February 21, 2022 from https://pubs.usgs.gov/wri/wri994104/pdf/wri994104.pdf

Macfarlane, P.A. (1996). An analysis of the upper part of the regional flow system along the southern ground-water flow "corridor" in the Dakota aquifer using a steady-state, vertical profile flow model. Open-File Report. http://www.kgs.ku.edu/Hydro/Publications/1996/OFR96_1d/OFR96-1d.pdf

3.24 Northern High Plains, High Plains

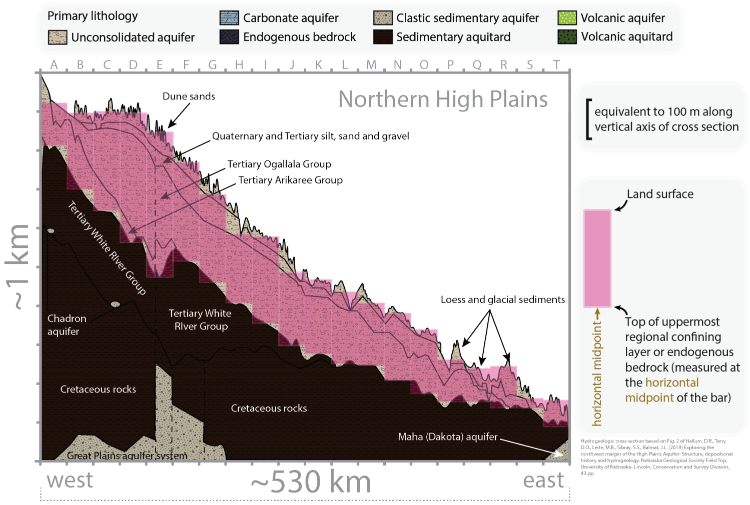

Supplementary Fig. 145. Hydrogeologic cross section. 20 equally spaced transparent pink bars overlie the cross section; each shaded bar depicts the vertical offset from the land surface to the top of the uppermost confining unit or endogenous bedrock.

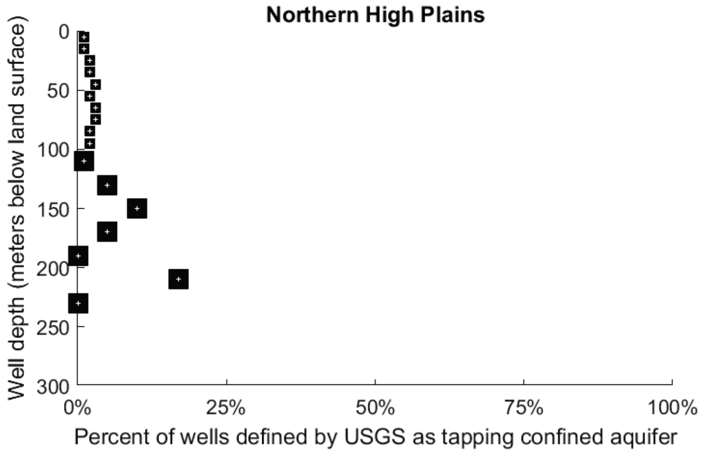

Supplementary Fig. 146. Vertical variations in the prevalence of wells that have been defined as tapping an unconfined or a confined aquifer by the USGS. The smaller squares represent 10 m depth intervals from the land surface to 100 m; the larger squares represent 20 m intervals from 100 m to 300 m below the land surface.

The Northern High Plains aquifer system is located, predominantly, in Nebraska.

(i) A hydrogeologic cross section presented in Fig. 2 by Hallum et al. (2019) depicts relatively deep depths (>200 m) to the uppermost relatively low-permeability unit.

(ii) We analysed wells within the study area that the USGS has defined as either unconfined or confined. Nearly all wells with depths of less than 240 m are defined as tapping unconfined aquifers. One well with a depth of 300 m is defined as tapping a confined aquifer (Dakota Sandstone aquifer).

Depth to confined conditions: >240 m (see (ii) above)

Reference: Hallum, D.R., Terry D.O., Leite, M.B., Sibray, S.S., Balmat, J.L. (2019) Exploring the northwest margin of the High Plains Aquifer: Structure, depositional history and hydrogeology. Nebraska Geological Society Field Trip, University of Nebraska–Lincoln, Conservation and Survey Division, 43 pp.

The table below presents a series of published quotes (see quotation marks denoting text quoted from another publication, which is cited following the quotation marks with the full reference written in full below the table). The leftmost column lists a title of a hydrogeologic formation depicted in the cross section on the previous page. The rightmost column presents a quote from a hydrogeological study (see base of table for citation). The quote has been annotated with colored text to highlight how we categorized each layer (i.e., see categories in the center column in the table). Specifically: (i) blue text highlights portions of a quote that provide insights into the degree of consolidation of the formation, (ii) red text highlights portions of a quote that categorize the formation as an aquifer or an aquitard (i.e., higher versus lower permeability in the context of local hydrogeologic formations), and (iii) green text highlights portions of a quote that provide information about the lithology of the formation.

Supplementary Table 27. Hydrostratigraphy details for the Northern High Plains

**Formation name**	**Category**	**Quote**
Quaternary-age deposit	Unconsolidated aquifer	"Quaternary-age deposits include alluvial, valley-fill, dune sand, and glacial deposits (fig. 6)." (Peterson et al., 2020) | Valley-fill deposits: "**Stream-laid deposits of gravel, sand, silt, and clay** associated with the **most recent cycle of erosion and deposition along present streams**. Usually hydraulically connected to underlying Quaternary and Tertiary deposits." (Peterson et al., 2020). Dune sand: "**Fine to medium sand with small amounts of clay, silt, and coarse sand formed into hills and ridges by the wind**." (Peterson et al., 2020) Loess: "**Silt with lesser amounts of very fine sand and clay deposited as windblown dust**. Where thickest, generally only the lowest 100 feet is below the water table." (Peterson et al., 2020). "Unconsolidated alluvial deposit: **Stream-laid deposits of gravel, sand, silt, and clay locally cemented by calcium carbonate into caliche or mortar beds**." (Peterson et al., 2020). "**Usually hydraulically connected to underlying Quaternary and Tertiary deposits**." (Peterson et al., 2020)
Ogallala Formation	Unconsolidated aquifer	"**Poorly sorted clay, silt, sand, and gravel** generally **unconsolidated**; forms caliche layers or mortar beds when cemented by calcium carbonate. Most of the saturated thickness of the Northern High Plains aquifer, though absent from the western and eastern ends." (Peterson et al., 2020) | "**The Ogallala Formation composes most of the Northern High Plains aquifer (fig. 6, table 2; Gutentag and others, 1984)**." (Peterson et al., 2020)
Arikaree Group	Clastic sedimentary **aquifer** (consolidated or semi-consolidated rock)	"Predominantly **massive very fine to fine-grained sandstone with localized beds of volcanic ash, silty sand, siltstone, claystone, sandy clay, limestone, marl, and mortar beds.**" (Peterson et al., 2020) **|** "**Ogallala and Arikaree Group strata that comprise the High Plains Aquifer**." (Hallum et al., 2019).
White River Group	Sedimentary **aquitard** (consolidated or semi-consolidated rock) *(may be aquifer where secondary permeability resulting*	"Upper unit, Brule Formation, predominantly **massive siltstone** containing **sandstone beds and channel deposits of sandstone**. Included where it contains saturated sandstones or interconnected fractures, mainly limited to western Nebraska. Otherwise, the top of the Brule Formation is considered the base of the Northern High Plains aquifer." (Peterson et al., 2020) | "**The Chadron and Brule Formations of the White River Group, together with the younger Arikaree Group, are generally fine-grained, low-**

Formation name	Category	Quote
	interconnected fractures - (Peterson et al., 2020, Gutentag et al., 1984).	**permeability units except for a few areas of high permeability and areas where permeability has been increased by fractures**." (Peterson et al., 2020)
Cretaceous rock (undifferentiated)	Sedimentary **aquitard** (consolidated or semi-consolidated rock)	"**Shales, chalks, limestones**, or **other poorly permeable deposits**, the top of which form the base of the Northern High Plains aquifer. **Most often, Pierre Shale, Niobrara Chalk, or Niobrara Shale**." (Peterson et al., 2020)
Maha (Dakota) aquifer	Clastic sedimentary **aquifer** (consolidated or semi-consolidated rock)	"The **upper aquifer of the Great Plains aquifer system is called the Maha aquifer**. This aquifer was formerly called the Dakota aquifer from the Dakota **Sandstone**, which is a prominent part of the aquifer." (Miller & Appel, 1997). "The Maha and the Apishapa aquifers consist of **loosely cemented, medium- to fine-grained sandstone**." (Miller & Appel, 1997). "**The Maha aquifer is more extensive** than the Apishapa aquifer (fig. 76);" (Miller & Appel, 1997).
Great Plains aquifer system	Clastic sedimentary **aquifer** (consolidated or semi-consolidated rock)	"**The Great Plains aquifer system** is exposed at the land surface in a band that extends from south-central Kansas to northeastern Nebraska (fig. 5). This aquifer system consists of **two sandstone aquifers in Cretaceous rocks, separated by a shale confining unit**." (Miller & Appel, 1997). "**Water in the Great Plains aquifer system is under confined conditions in most places**." (Miller & Appel, 1997). "**Sand bodies are typically linear, lenticular, or sinuous, which indicates that they were deposited in deltaic, shoreline, or fluvial environments**."

Hallum, D.R., Terry D.O., Leite, M.B., Sibray, S.S., Balmat, J.L. (2019). Exploring the northwest margin of the High Plains Aquifer: Structure, depositional history and hydrogeology. Nebraska Geological Society Field Trip, University of Nebraska–Lincoln, Conservation and Survey Division, 43 pp.

Peterson, S.M., Traylor, J.P., Guira, M. (2020). Groundwater Availability of the Northern High Plains Aquifer in Colorado, Kansas, Nebraska, South Dakota, and Wyoming. U.S. Geological Survey Professional Paper 1864, 57 p., Accessed November 28, 2021 from https://pubs.usgs.gov/pp/1864/pp1864.pdf

Gutentag, E.D., Heimes, F.J., Krothe, N. C., Luckey, R. R., Weeks, J. B. (1984). Geohydrology of the High Plains aquifer in parts of Colorado, Kansas, Nebraska, New Mexico, Oklahoma, South Dakota, Texas, and Wyoming (No. 1400-B). Accessed February 10, 2021 from https://pubs.usgs.gov/pp/1400b/report.pdf

Miller, J.A., Appel, C.L. (1997). Ground water atlas of the United States: Segment 3, Kansas, Missouri, Nebraska (No. 730-D, pp. D1-D24). US Geological Survey. Accessed April 22, 2022 from https://pubs.er.usgs.gov/publication/ha730D

3.25 Southern High Plains, High Plains

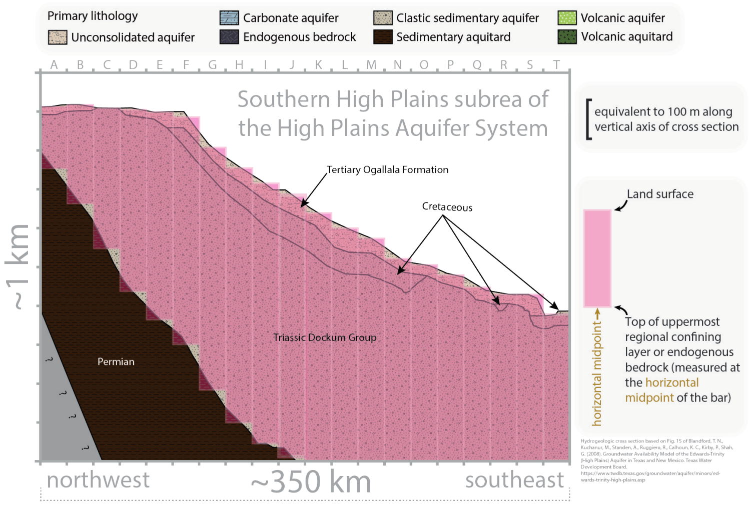

Supplementary Fig. 147. Hydrogeologic cross section. 20 equally spaced transparent pink bars overlie the cross section; each shaded bar depicts the vertical offset from the land surface to the top of the uppermost confining unit or endogenous bedrock.

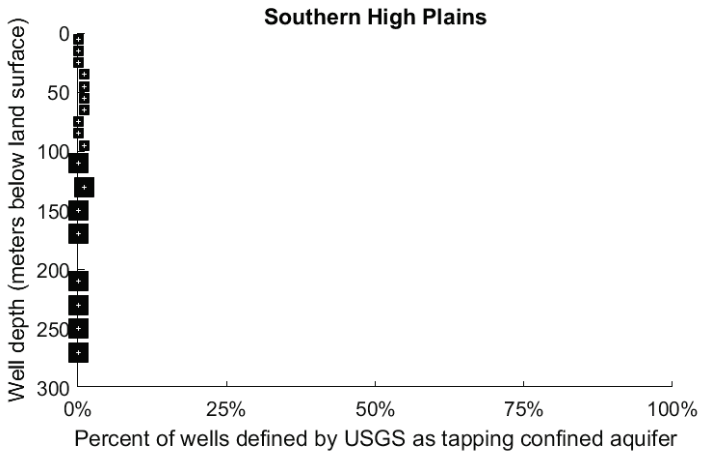

Supplementary Fig. 148. Vertical variations in the prevalence of wells that have been defined as tapping an unconfined or a confined aquifer by the USGS. The smaller squares represent 10 m depth intervals from the land surface to 100 m; the larger squares represent 20 m intervals from 100 m to 300 m below the land surface.

The Southern High Plains aquifer system is located in eastern New Mexico and western Texas.

(i) A hydrogeologic cross section presented in Fig. 15 by Blandford et al. (2008) suggests that the aquifer system does not depict a clear confining unit within the shallow (<200 m) portion of the aquifer system.

(ii) We analysed wells within the study area that the USGS has defined as either unconfined or confined. Nearly all wells are defined as tapping an unconfined aquifer, including the deepest well in the dataset (274 m).

Depth to confined conditions: >270 m (see (ii) above)

Reference: Blandford, T. N., Kuchanur, M., Standen, A., Ruggiero, R., Calhoun, K. C., Kirby, P., Shah, G. (2008). Groundwater Availability Model of the Edwards-Trinity (High Plains) Aquifer in Texas and New Mexico. Texas Water Development Board. https://www.twdb.texas.gov/groundwater/aquifer/minors/edwards-trinity-high-plains.asp

The table below presents a series of published quotes (see quotation marks denoting text quoted from another publication, which is cited following the quotation marks with the full reference written in full below the table). The leftmost column lists a title of a hydrogeologic formation depicted in the cross section on the previous page. The rightmost column presents a quote from a hydrogeological study (see base of table for citation). The quote has been annotated with colored text to highlight how we categorized each layer (i.e., see categories in the center column in the table). Specifically: (i) blue text highlights portions of a quote that provide insights into the degree of consolidation of the formation, (ii) red text highlights portions of a quote that categorize the formation as an aquifer or an aquitard (i.e., higher versus lower permeability in the context of local hydrogeologic formations), and (iii) green text highlights portions of a quote that provide information about the lithology of the formation.

Supplementary Table 28. Hydrostratigraphy details for the Southern High Plains

Formation name	Category	Quote
Tertiary Ogallala Formation	Unconsolidated aquifer	“**Tan, yellow, and reddish brown silt, clay, sand, and gravel.** Caliche layers common near the surface.” (Table 1, Blandford, 2008). “**Yields moderate to large amounts of water to wells across Southern High Plains**.” (Table 1, Blandford, 2008). “Depositional environments of the Ogallala Formation have been interpreted as including **coalescing alluvial fans or alluvial aprons** (Johnson, 1901; Frye and Leonard, 1964; Seni, 1980; Reeves, 1984) or **fluvial-dominated valley fill sequences confined within paleovalleys** (Gustavson, 1996).” (Blandford et al., 2008)
Cretaceous *(Duck Creek and Kiamichi Formation are aquitard; Carbonate aquifer exists in Edwards and Comanche Peak Formations)*	Clastic sedimentary **aquifer** (consolidated or semi-consolidated rock)	Edwards Formation: “Light gray to yellowish gray, **thick to massive bedded**, **fine to coarse-grained limestone**.” (Table 1, Blandford et al., 2008). Comanche Peak Formation: “Light gray to yellowish brown, **irregularly bedded argillaceous limestone with thin interbeds of light gray shale**.” For both Edwards and Comanche Peak Formation: “Generally **yields fairly small amounts of water to wells beneath Southern High Plains, but may yield large amounts of water locally due to fractures and solution cavities**.” (Table 1, Blandford et al., 2008).<br><br>Cretaceous Trinity Group, Antlers Formation: “**White, gray, yellowish brown to purple, argillaceous, loosely cemented sand, sandstone, and conglomerate with interbeds of siltstone and clay**.” (Table 1, Blandford et al., 2008). “**Yields small to moderate amounts of water to wells**.” (Table 1, Blandford et al., 2008). Cretaceous System, Hydrogeologic Units: “**Edwards-Trinity (High Plains) Aquifer**” (Table 1, Blandford et al., 2008).
Triassic Dockum Group	Clastic sedimentary **aquifer** (consolidated or semi-consolidated rock)	“The Triassic section can be as **much as 2,000 feet thick, and its low-permeability sediments** in the upper portion of the section separate groundwater in the Southern Ogallala and Edwards- Trinity (High Plains) Aquifers from groundwater in Triassic sandstone units, referred to as the **Dockum Aquifer**.” (Blandford et al., 2008). “**Multi-colored fine- to coarse grained micaceous sandstone with some claystone and shale interbeds**.” (Table 1, Blandford et al., 2008).

Formation name	Category	Quote
Permian *(evaporite beds exists)*	Sedimentary **aquitard** (consolidated or semi-consolidated rock)	“buried Permian **salt beds**” (Blandford et al., 2008). “**Bedrock units** in contact with the High Plains aquifer range in age from Permian to Tertiary.” (Gutentag et al., 1984).

Blandford, T. N., Kuchanur, M., Standen, A., Ruggiero, R., Calhoun, K. C., Kirby, P., Shah, G. (2008, December). Groundwater Availability Model of the Edwards-Trinity (High Plains) Aquifer in Texas and New Mexico. Texas Water Development Board. Accessed June 6, 2022 via https://www.twdb.texas.gov/groundwater/aquifer/minors/edwards-trinity-high-plains.asp

Gutentag, E.D., Heimes, F.J., Krothe, N.C., Luckey, R.R., Weeks, J.B. (1984). Geohydrology of the High Plains aquifer in parts of Colorado, Kansas, Nebraska, New Mexico, Oklahoma, South Dakota, Texas, and Wyoming (No. 1400-B). Accessed June 6, 2022 via https://pubs.er.usgs.gov/publication/pp1400B

3.26 Albuquerque Basin, Middle Rio Grande

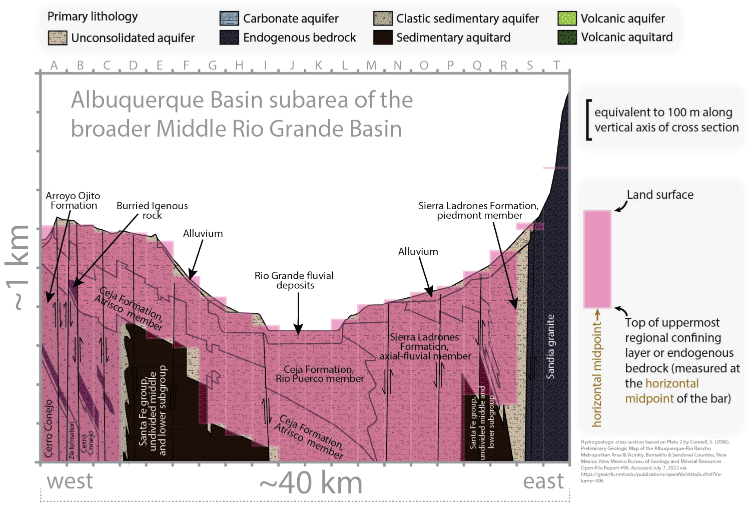

Supplementary Fig. 149. Hydrogeologic cross section. 20 equally spaced transparent pink bars overlie the cross section; each shaded bar depicts the vertical offset from the land surface to the top of the uppermost confining unit or endogenous bedrock.

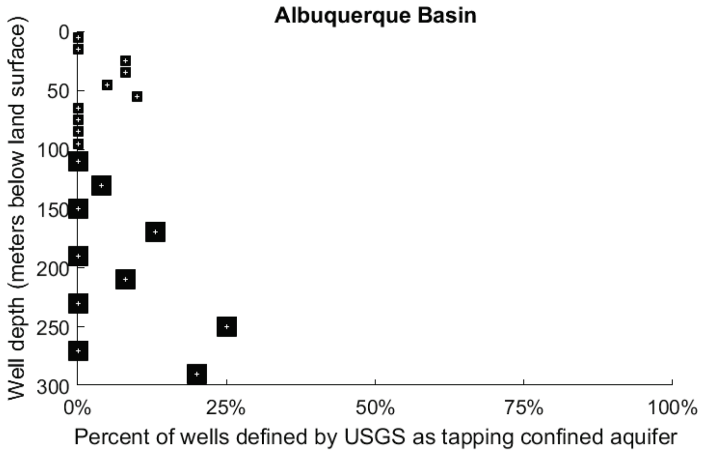

Supplementary Fig. 150. Vertical variations in the prevalence of wells that have been defined as tapping an unconfined or a confined aquifer by the USGS. The smaller squares represent 10 m depth intervals from the land surface to 100 m; the larger squares represent 20 m intervals from 100 m to 300 m below the land surface.

The Albuquerque Basin is located in central New Mexico.

(i) A hydrogeologic cross section presented in Plate 2 by Connell (2006) depicts thick (>200 m) poorly consolidated aquifers with a high density of faulting.

(ii) We analysed wells within the study area that the USGS has defined as either unconfined or confined. Nearly all wells in the study area are defined in the USGS dataset as tapping an unconfined aquifer, including the deepest well in the dataset (631 m).

Depth to confined conditions: >630 m (see (ii) above)

Reference: Connell, S. (2006). Preliminary Geologic Map of the Albuquerque-Rio Rancho Metropolitan Area & Vicinity, Bernalillo & Sandoval Counties, New Mexico. New Mexico Bureau of Geology and Mineral Resources Open-File Report 496. Accessed July 7, 2022 via https://geoinfo.nmt.edu/publications/openfile/details.cfml?Volume=496

The table below presents a series of published quotes (see quotation marks denoting text quoted from another publication, which is cited following the quotation marks with the full reference written in full below the table). The leftmost column lists a title of a hydrogeologic formation depicted in the cross section on the previous page. The rightmost column presents a quote from a hydrogeological study (see base of table for citation). The quote has been annotated with colored text to highlight how we categorized each layer (i.e., see categories in the center column in the table). Specifically: (i) blue text highlights portions of a quote that provide insights into the degree of consolidation of the formation, (ii) red text highlights portions of a quote that categorize the formation as an aquifer or an aquitard (i.e., higher versus lower permeability in the context of local hydrogeologic formations), and (iii) green text highlights portions of a quote that provide information about the lithology of the formation.

Supplementary Table 29. Hydrostratigraphy details for the Albuquerque

Formation name	Category	Quote
Qu — Alluvium, undivided	Unconsolidated aquifer	"**River alluvium; channel, floodplain, and lower terraces deposits** of inner Rio Grande and Puerco valleys; as much as 120 ft thick. Map unit "Qf' (Kelley 1977). Forms upper part of the "**shallow aquifer**" system. Hydrogeologic (lithofacies) subdivision Iv•. *Age: Holocene to late Pleistocene."* (Hawley et al., 1995).
Qr — Rio Grande fluvial deposits, undivided	Unconsolidated aquifer	From (Connel, 2006), Plate 1, Explanation of Map Units " **Las Padillas Formation (Qrp, historic-upper Holocene) — fluvial deposits of the Rio Grande**; **pinkish-gray to grayish-brown sand and pebbly sand with lenses of reddish-brown silt and clay; contains paleochannel, point-bar, and overbank levee deposits**; underlies modern (inner) valley of the Rio Grande; very weak to no soil development; base not exposed; **15-34 m thick in wells**; proposed by Connell and Love (2001); divided into 8 units based largely on surface morphology (Kelson et al., 1999): ". "The other major hydrostratigraphic unit (RA) comprises Rio Grande and Puerco deposited of late Quaternary age (<15,000) that form the upper part of the **regional shallow-aquifer system**." (Hawley and Haase, 1992).
Ts — Santa Fe Group, undivided middle and lower subgroup	Sedimentary **aquitard** (consolidated or semi-consolidated rock)	"The ***lower and middle parts of the Santa Fe Group*** are locally well indurated and contain a **large amount of fine- to medium-grained elastic material** (**clay, silt, and fine sand**)." (Hawley et al., 1994). "With the exception of locally extensive and thick eolian sand deposits (e.g., Zia Fm in the lower Santa Fe Group), **lower and middle Santa Fe units do not produce significant amounts of good quality groundwater** even though they constitute the bulk of the basin-fill sequence." (Hawley et al., 1995).
QTsp — Sierra Ladrones Formation, piedmont member	Unconsolidated aquifer	From (Connel, 2006), Plate 1, Explanation of Map Units "**Sierra Ladrones Formation, piedmont member (QTsp, Miocene(?), Pliocene-lower Pleistocene) — reddish- to yellowish-brown conglomerate, sandstone, and minor mudstone; contains weakly developed paleosols and upward-fining sequences of gravel, sand, and mud; contains limestone, sandstone and granite clasts; sparse hypabyssal intrusive clasts present at northeastern end of map area; generally poorly consolidated; interfingers with axial-fluvial member (QTsa); 0-600(?) m thick**.". "Unit includes Ceja Member of Kelley (1977), and **Sierra Ladrones Formation** of Machette (1978a,b) and Lozinsky and Tedford (1991). Forms **lower part of "shallow aquifer" below river-floodplain areas, and upper part of basin-fill aquifer** in western part of NE and SE Albuquerque well fields." (Hawley and Haase, 1992).
QTsa — Sierra	Unconsolidated aquifer	From (Connel, 2006), Plate 1, Explanation of Map Units "**Sierra Ladrones Formation, axial — fluvial member (QTsa, Miocene(?), Pliocene —**

Formation name	Category	Quote
Ladrones Formation, axial-fluvial member		**lower Pleistocene) — light — gray to yellowish — brown sand, pebbly to cobbly sand, and sparse interbedded mud; clasts dominated by rounded othoquartzite and volcanic rocks**; **deposits associated with the ancestral Rio Grande; interfingers with piedmont member (QTsp); contains early Irvingtonian mammalian fossils (*SB-1*, Table 1); base not exposed; estimated thickness more than 300-800 m**." "Sand, pebbly sand and silty sand beds in facies III form a major part of the **basin-fill aquifer** in the central Albuquerque Basin." (Hawley and Haase, 1992). "Unit includes Ceja Member of Kelley (1977), and **Sierra Ladrones Formation** of Machette (1978a,b) and Lozinsky and Tedford (1991). Forms **lower part of "shallow aquifer" below river-floodplain areas, and upper part of basin-fill aquifer** in western part of NE and SE Albuquerque well fields." (Hawley and Haase, 1992).
Tcrp — Ceja Formation, Rio Puerco Member	Unconsolidated aquifer	From (Connel, 2006), Plate 1, Explanation of Map Units " **Ceja Formation (Tc, Tcrg, Tcau, Tca, Tcs, Pliocene-lowest Pleistocene(?)) — sand, gravel, and mud derived from western and northwestern Albuquerque basin**; disconformably overlies Arroyo Ojito Formation (**Ton** and **Top**) and generally coarsens upsection; defined by Kelley (1977); contains Blancan mammalian fossils (*SB-2*, Table 1); **20-700(?) m thick**; divided into three members and one subunit:". "The other major hydrostratigraphic unit (RA) comprises Rio Grande and Puerco deposited of late Quaternary age (<15,000) that form the upper part of the **regional shallow-aquifer system**." (Hawley and Haase, 1992).
Tca — Ceja Formation, Atrisco Members	Unconsolidated aquifer	From (Connel, 2006), Plate 1, Explanation of Map Units "**Atrisco Member (Tca, Pliocene) — pink to yellowish-brown sandstone, pebbly sandstone, and mudstone**; interpreted to **interfinger with Sierra Ladrones Formation (QTsa) to east; rests on Arroyo Ojito Formation (Ton, Top) and *Rincones paleosurface*; locally subdivided into upper sandy subunit (Tcau)**; defined by Connell et al. (1998a); **20-600(?) m thick**.". "Unit includes **Ceja Member** of Kelley (1977), and Sierra Ladrones Formation of Machette (1978a,b) and Lozinsky and Tedford (1991). Forms **lower part of "shallow aquifer" below river-floodplain areas, and upper part of basin-fill aquifer** in western part of NE and SE Albuquerque well fields." (Hawley and Haase, 1992).
To — Arroyo Ojito Formation, undivided	Sedimentary **aquifer** (consolidated or semi-consolidated rock)	From (Connel, 2006), Plate 1, Explanation of Map Units "**Arroyo Ojito Formation (1999) (To, Ton, Tob, Top, upper Miocene) — gravel-bearing fluvial deposits derived from north and northwest of the Albuquerque basin**; defined by Connell et al. (1999) and modified by Connell (*in preparation*); **437-456 m thick at type section in Arroyo Ojito; divided into three conformable members**:". "Buried **arroyo-channel deposits** of a **large alluvial fan** that spread out from the mouth of Ti.jeras Canyon (facies Vd) **form another major hydrogeologic unit** (middle and upper Santa Fe; MSF-1 and USF-1) **that has greater than average aquifer potential**." (Hawley and Haase, 1992).
Tcc — Cerro Conejo Formation	Sedimentary **aquifer** (consolidated or semi-consolidated rock)	From (Connel, 2006), Plate 1, Explanation of Map Units "**Cerro Conejo Formation (Tcc, middle-upper Miocene) — pink to very pale-brown tabular and cross-stratified sandstone with thin to medium bedded mudstone; contains sandstone concretions and volcanic fallout ashes (11.3-10.8, *SA-25* through *SA28*, Table 1), and late Barstovian mammalian fossils** (*SB-3, SB-4*, Table 1); **base is probably disconformable with the Zia Formation; top may be disconformable with Arroyo Ojito Formation to west, but interfingers with Navajo Draw Member to east; originally defined as Cerro Conejo Member**

Formation name	Category	Quote
		(**Zia Formation**) by Connell et al. (1999), but elevated to formation rank based on mappability; **245-316 m thick**.". "**Eolian (Zia) and facies could be at least a local (future) source of groundwater** in the far northwestern part of the basin (west and northwest of Rio Rancho)." (Hawley and Haase, 1992).
Tz — Zia Formation, undivided	Sedimentary **aquifer** (consolidated or semi-consolidated rock)	From (Connel, 2006), Plate 1, Explanation of Map Units "**Zia Formation (Tz, lower-middle Miocene) — cross-stratified sandstone and mudstone**; unconformably overlies Galisteo and Menefee Formations (**Tg, Kvm**) and unit of Isleta well #2 (**Tis**, in subsurface, see Plate 2); defined by Galusha (1966) and Gawne (1981) and contains late Arikareean through Hemmingfordian mammalian fossils (*SB-5*, Table 1; Galusha, 1966) and divided into three members: **Cañada Pilares Member (Tzr, middle Miocene) — red mudstone and sandstone; discontinuously exposed; defined by Gawne (1981); 8-75 m thick. Chamisa Mesa Member (Tzm, middle Miocene) — pale-brown to light reddish-brown cross-stratified fluvial and eolian sandstone and mudstone; 30-200 m thick. Piedra Parada Member (Tzp, lower-middle Miocene) — whitish-gray to pinkish-gray cross-stratified sandstone, eolian in origin; 70-122 m thick.".** "**Eolian (Zia) and facies could be at least a local (future) source of groundwater** in the far northwestern part of the basin (west and northwest of Rio Rancho)." (Hawley and Haase, 1992).
Tvi — Buried Igneous rocks	Endogenous bedrock	From (Connel, 2008), under the Description of Map and Cross Section Units, "**Buried Igneous rocks** (Pliocene – Oligocene) **Igneous rocks**, locally encountered in boreholes".
Ys — Sandia granite	Endogenous bedrock	From (Connel, 2006), Plate 1, Explanation of Map Units " **Sandia granite (Ys, Mesoproterozoic) — pink megacrystic biotite monzogranite and granodiorite; includes zones of sheared megacrystic biotite monzogranite and granodiorite of the Seven Springs shear zone (Yss) just north of trace of Tijeras fault zone**; U-Pb dates on zircon indicate **age of crystallization between 1455±12 Ma and 1446±26 Ma.**". "The predominant **granitic material** is most likely derived from the **Sandia Granite**." (Hawley and Haase, 1992).

Hawley, J.W., Haase, C.S. (1992) Hydrogeologic framework of the northern Albuquerque Basin. Open-File Report 387:74 New Mexico Bureau of Mines and Mineral Resources, Socorro, NM. Accessed July 9, 2022 via https://geoinfo.nmt.edu/publications/openfile/downloads/300-399/387/ofr_387.pdf

Hawley, J.W., Haase, C. S., Lozinsky, R. P. (1995). An underground view of the Albuquerque Basin. In The Water Future of Albuquerque and Middle Rio Grande Basin: Proceedings of the 39th Annual New Mexico Water Conference: November 3-4, 1994, Albuquerque, New Mexico (No. 290, p. 37). New Mexico Water Resources Research Institute, New Mexico State University. Accessed July 10, 2022 via https://nmwrri.nmsu.edu/wp-content/uploads/2015/watcon/proc39/Hawley.pdf

Connell, S.D. (2006). Preliminary geologic map of the Albuquerque–Rio Rancho metropolitan area and vicinity. *Bernalillo and Sandoval Counties, New Mexico: New Mexico Bureau of Geology and Mineral Resources, Open-file Report*, *496*(2). Plate 1 and Plate 2 accessed July 9, 2022 via https://geoinfo.nmt.edu/publications/openfile/details.cfml?Volume=496

Connell, S.D. (2008). *Geologic Map of the Albuquerque-Rio Rancho Metropolitan Area and Vicinity: Bernalillo and Sandoval Counties, New Mexico*. New Mexico Bureau of Geology and Mineral Resources. Accessed July 9, 2022 via https://geoinfo.nmt.edu/publications/openfile/downloads/400-499/496/OFR-496_Plate2_xsects.pdf

3.27 San Luis Valley, Middle Rio Grande

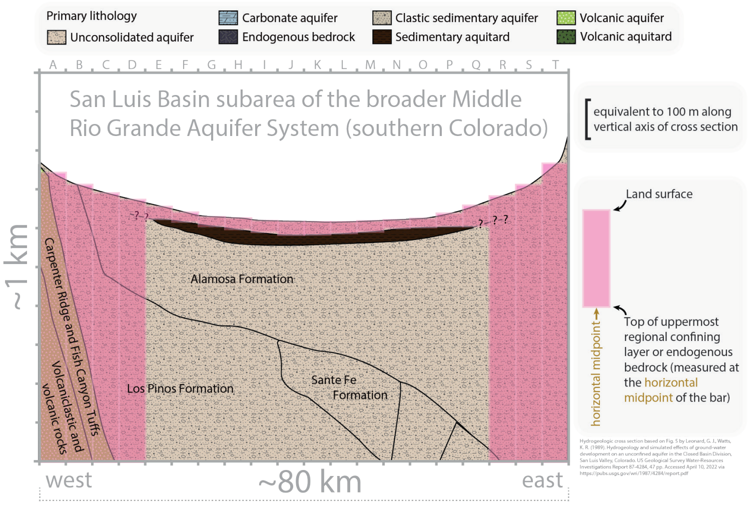

Supplementary Fig. 151. Hydrogeologic cross section. 20 equally spaced transparent pink bars overlie the cross section; each shaded bar depicts the vertical offset from the land surface to the top of the uppermost confining unit or endogenous bedrock.

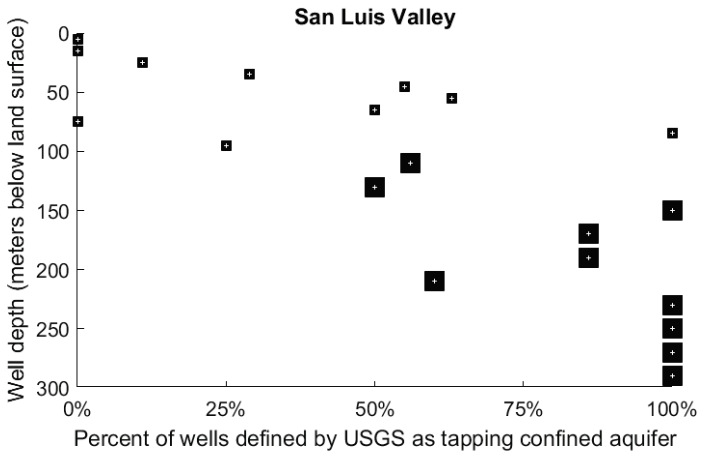

Supplementary Fig. 152. Vertical variations in the prevalence of wells that have been defined as tapping an unconfined or a confined aquifer by the USGS. The smaller squares represent 10 m depth intervals from the land surface to 100 m; the larger squares represent 20 m intervals from 100 m to 300 m below the land surface.

The San Luis Valley is located in southern Colorado.

(i) A hydrogeologic cross section presented in Fig. 5 by Leonard and Watts (1989) does not depict a clear confining unit within the aquifer system. However, the hydrogeologic cross section in Plate 6 by Repplier et al. (1981) does depict a shallow confining unit (*note: this cross section is incorrectly labelled in the cited publication as “Raton Basin”; we confirmed that this cross section represents the San Luis Valley (not the Raton Basin) via personal communication with a Senior Hydrogeologist at the Colorado Geological Survey on April 18, 2022). We added a confining unit to the cross section (top left of this page) on the basis of that work.

(ii) We analysed wells within the study area that the USGS has defined as either unconfined or confined. Most (>80%) wells at depths of 140-160 m and at depths exceeding 140 m are defined as tapping a confined aquifer.

Depth to confined conditions: 140-160 m (see (ii) above)

Reference: Leonard, G.J., Watts, K.R. (1989). Hydrogeology and simulated effects of ground-water development on an unconfined aquifer in the Closed Basin Division, San Luis Valley, Colorado. US Geological Survey Water-Resources Investigations Report 87-4284, 47 pp. Accessed April 10, 2022 via https://pubs.usgs.gov/wri/1987/4284/report.pdf

Repplier, F.N., Healy, F.C., Longmire, P.A. (1981). Atlas of Ground Water Quality in Colorado. Map Series 16, https://coloradogeologicalsurvey.org/publications/atlas-ground-water-quality-colorado/

The table below presents a series of published quotes (see quotation marks denoting text quoted from another publication, which is cited following the quotation marks with the full reference written in full below the table). The leftmost column lists a title of a hydrogeologic formation depicted in the cross section on the previous page. The rightmost column presents a quote from a hydrogeological study (see base of table for citation). The quote has been annotated with colored text to highlight how we categorized each layer (i.e., see categories in the center column in the table). Specifically: (i) blue text highlights portions of a quote that provide insights into the degree of consolidation of the formation, (ii) red text highlights portions of a quote that categorize the formation as an aquifer or an aquitard (i.e., higher versus lower permeability in the context of local hydrogeologic formations), and (iii) green text highlights portions of a quote that provide information about the lithology of the formation.

Supplementary Table 30. Hydrostratigraphy details for the San Luis Valley

**Formation name**	**Category**	**Quote**
Alamosa Formation *(Clay layer exists)*	Unconsolidated aquifer	"The **Alamosa Formation and overlying deposits** consist of **discontinuous beds** of **clay, silt, sand, and gravel**." (Leonard and Watts, 1987). "Alamosa Formation has a **maximum thickness of about 2,050 ft** in the topographic low (Burroughs, 1981)." Leonard and Watts (1987). "The **valley-fill deposits** of the San Luis Valley **form aquifers that contain ground water**." (Leonard and Watts, 1987).
Santa Fe Formation	Unconsolidated aquifer	"The Santa Fe Formation of Miocene and Pliocene age consists of **buff to pinkish-orange clays with interbedded, poorly to moderately sorted silty sands**." (Leonard and Watts (1987). "all 'of the **Santa Fe**, Los Pinos, Oligocene (?) volcaniclastics, Vallejo and older vo'lcanics and volcaniclastics are lumped into **a single aquifer, the "confined."**" (Burroughs, 1981).
Los Pinos Formation	Unconsolidated aquifer	"the **Los Pinos Formation** consists of **sandy gravel with interbedded volcaniclastic sandstone and tuffaceous material** that was deposited as an eastward thickening wedge along the eastern border of the San Juan Mountains." Leonard and Watts (1987) "all 'of the Santa Fe, **Los Pinos**, Oligocene (?) volcaniclastics, Vallejo and older vo'lcanics and volcaniclastics are lumped into **a single aquifer, the "confined."**" (Burroughs, 1981).
Carpenter Ridge and Fish Canyon Tuffs	Volcanic aquifer	"The upper boundary of these volcaniclastics has been placed at the base of the **Fish Canyon-Carpenter Ridge ash flow tuffs**. These tuffs are at the base of and are **interbedded, with the Los Pinos sands** (Lipman, 1975)." (Burroughs, 1981). "**The Fish Canyon-Carpenter Ridge ash flow tuffs** and the Oligocene (?) volcaniclastics should be the **main reservoir rocks for geothermal water** in the Monte Vista graben." (Burroughs, 1981).
Volcaniclastic and volcanic rocks	Volcanic aquifer	"The basal interval consists of **older volcanic and volcaniclastic rocks**" "The Fish Canyon-Carpenter Ridge ash flow tuffs and **the Oligocene (?) volcaniclastics** should be the **main reservoir rocks for geothermal water** in the Monte Vista graben." (Burroughs, 1981).

Leonard, G.J., Watts, K.R. (1989). Hydrogeology and simulated effects of ground-water development on an unconfined aquifer in the Closed Basin Division, San Luis Valley, Colorado. US Geological Survey No. 87-4284. Accessed June 9, 2022 via https://pubs.usgs.gov/wri/1987/4284/report.pdf

Burroughs, R L. (1981). Summary of the geology of the San Luis Basin, Colorado-New Mexico with emphasis on the geothermal potential for the Monte Vista Graben. Special Publication 17. United States doi:10.2172/6763319.

3.28 Central Mississippi Embayment, Mississippi Embayment

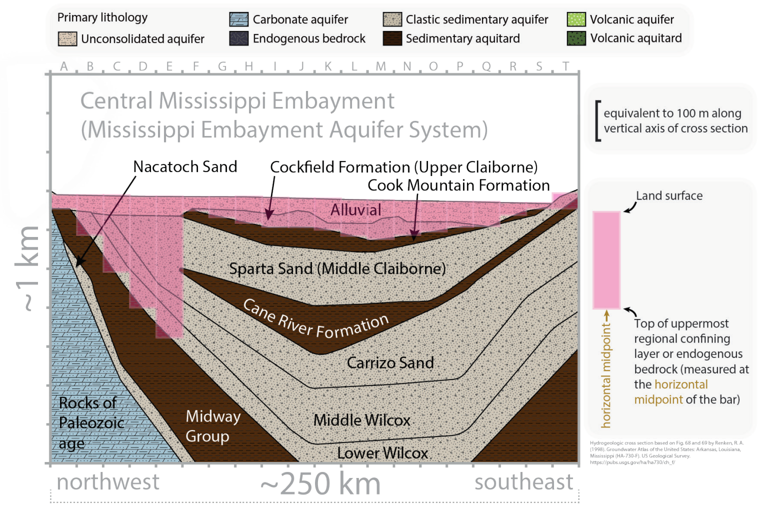

Supplementary Fig. 153. Hydrogeologic cross section. 20 equally spaced transparent pink bars overlie the cross section; each shaded bar depicts the vertical offset from the land surface to the top of the uppermost confining unit or endogenous bedrock.

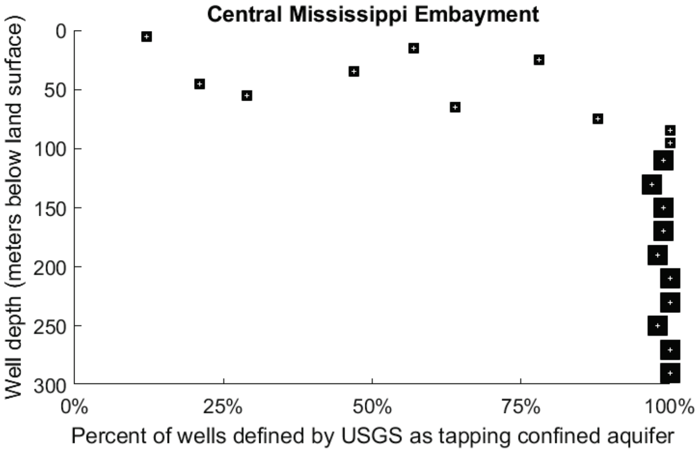

Supplementary Fig. 154. Vertical variations in the prevalence of wells that have been defined as tapping an unconfined or a confined aquifer by the USGS. The smaller squares represent 10 m depth intervals from the land surface to 100 m; the larger squares represent 20 m intervals from 100 m to 300 m below the land surface.

The Central Mississippi Embayment is located near the Mississippi River in Arkansas, Louisiana, Mississippi and Tennessee.

(i) A hydrogeologic cross section presented in Figs. 68 and 69 by Renken (1998) suggests that the uppermost confining unit (named the Cook Mountain Formation, in the area where the cross section is located) is typically 75 meters below land surface (median vertical offset of the 20 pink transparent bars in the cross section to the left; the 25th-75th percentile range is 49-94 meters below land surface).

(ii) We analysed wells within the study area that the USGS has defined as either unconfined or confined. Most (>80%) wells at depths of 70-80 m and at depths exceeding 70 m are defined as tapping a confined aquifer. This estimate is consistent with the median depth to the top of the uppermost confining layer shown in the hydrogeologic cross section (see (i) above).

(iii) Renken (1998) state that (following text quoted directly): *“The alluvial aquifers consist of gravel and sand deposits of Quaternary age and generally contain ground water under unconfined conditions”.*

Depth to confined conditions: 70-80 m (see (i) and (ii) above)

References: Renken, R. A. (1998). Groundwater Atlas of the United States: Arkansas, Louisiana, Mississippi (HA-730-F). US Geological Survey. https://pubs.usgs.gov/ha/ha730/ch_f/

The table below presents a series of published quotes (see quotation marks denoting text quoted from another publication, which is cited following the quotation marks with the full reference written in full below the table). The leftmost column lists a title of a hydrogeologic formation depicted in the cross section on the previous page. The rightmost column presents a quote from a hydrogeological study (see base of table for citation). The quote has been annotated with colored text to highlight how we categorized each layer (i.e., see categories in the center column in the table). Specifically: (i) blue text highlights portions of a quote that provide insights into the degree of consolidation of the formation, (ii) red text highlights portions of a quote that categorize the formation as an aquifer or an aquitard (i.e., higher versus lower permeability in the context of local hydrogeologic formations), and (iii) green text highlights portions of a quote that provide information about the lithology of the formation.

Supplementary Table 31. Hydrostratigraphy details for the Mississippi Embayment Aquifer System

Formation name	Category	Quote
Mississippi River Valley alluvial aquifer	Unconsolidated aquifer	The **alluvial deposits** that make up the aquifer are mostly of Holocene age." (Hosman, & Weiss, (1991) | "The materials constituting the aquifer range in **size from coarse gravel to clay**. They commonly grade downward from **fine sand, silt, and clay at the top to coarse sand or gravel at the base**." (Hosman & Weiss 1991)
Upper Claiborne aquifer	Clastic sedimentary **aquifer** (consolidated or semi-consolidated rock)	"The **aquifer** consists of **interbedded fine sand, silt, and clay with common occurrences of lignite**." (Hosman & Weiss 1991)
Cook Mountain Formation	Sedimentary rock **aquitard** (consolidated or semi-consolidated rock)	"The **clay**, which is mostly the Cook Mountain Formation (included in the Laredo Formation in part of southern Texas), underlies about 92,000 mi^2 of the area." (Hosman, & Weiss, (1991) | "the Cook Mountain generally constitutes a **confining bed**" (Hosman & Weiss 1991)
Middle Claiborne aquifer (Sparta Sand)	Clastic sedimentary **aquifer** (consolidated or semi-consolidated rock)	"This **aquifer** underlies about 136,000 mi^2 and consists primarily of the **Sparta Sand**, which is present in most of the study area as a continentally derived **sand with clay interbeds of varying thickness and extent.**" (Hosman & Weiss 1991)
Cane River Formation	Sedimentary rock **aquitard** (consolidated or semi-consolidated rock)	"In Texas, three formations are equivalent to the Cane River Formation. They are, in ascending order, the Reklaw Formation, the Queen City Sand, and the Weches Formation (except in extreme southern Texas, where the Bigford Formation and El Pico **Clay** represent these units). The Reklaw Formation, which **is mostly clay**, is virtually the entire lower Claiborne **confining unit** east of the Sabine uplift." (Hosman & Weiss 1991)
Lower Claiborne-Upper Wilcox aquifer	Clastic sedimentary **aquifer** (consolidated or semi-consolidated rock)	"Most of the **aquifer** is the Carrizo Sand and its equivalent, the Meridian Sand Member of the Tallahatta Formation (table 1). The Carrizo or Meridian is an extensive **sand**, commonly **massive and unbroken by clay beds, that represents the basal unit** of the Claiborne Group." (Hosman & Weiss 1991)
Middle Wilcox aquifer	Clastic sedimentary **aquifer** (consolidated or semi-consolidated rock)	"Because the middle Wilcox **aquifer** is composed chiefly of **thin interbedded sand, silt, and clay**, it has **water-bearing characteristics different from those of typical massive and aquifers**." (Hosman & Weiss 1991)
Lower Wilcox aquifer	Clastic sedimentary **aquifer**	"This **aquifer** only occurs in the Mississippi embayment aquifer system. In the northern part of the Mississippi

Formation name	Category	Quote
	(consolidated or semi-consolidated rock)	embayment, a **massive sand** aquifer, the Fort Pillow Sand of Tennessee, Arkansas, and Missouri (Moore and Brown, 1969), occurs in the lower to middle part of the Wilcox deposits." (Hosman & Weiss 1991)
Midway Group	Sedimentary **aquitard** (consolidated or semi-consolidated rock)	"The Midway (Paleocene) **confining unit** (pi. 18) is a **thick confining layer** that is the base of the flow system for Tertiary aquifers in most of the study area." (Hosman, & Weiss, (1991) | "The Midway consists mostly of **dense marine clays, with lesser amounts of calcareous materials in the lower part**." (Hosman & Weiss 1991)
Nacatoch Sand	Clastic sedimentary **aquifer** (consolidated or semi-consolidated rock)	"**An important aquifer** in the northeastern part of the study area, it is composed of **sand beds** in the Nacatoch Sand in Arkansas; the McNairy Sand in Missouri, Illinois, Kentucky, and Tennessee; and the McNairy **Sand** Member of the Ripley Formation of northern Mississippi." Hosman & Weiss (1991)
Ozark Plateaus aquifer system	Carbonate aquifer	"**Flat-lying** to southward-dipping **limestone, dolomite, and sandstone** comprise the **principal aquifers** of the Ozark Plateaus aquifer system" Renken (1998)

Hosman, R. L., Weiss, J. S. (1991). Geohydrologic units of the Mississippi embayment and Texas coastal uplands aquifer systems, south-central United States (No. 1416-B). US Government Printing Office. https://pubs.er.usgs.gov/publication/pp1416B

Renken, R. A. (1998). Ground Water Atlas of the United States: Segment 5, Arkansas, Louisiana, Mississippi (No. 730-F, pp. F1-F28). US Geological Survey. https://pubs.er.usgs.gov/publication/ha730F

3.29 Eastern Mississippi Embayment, Mississippi Embayment

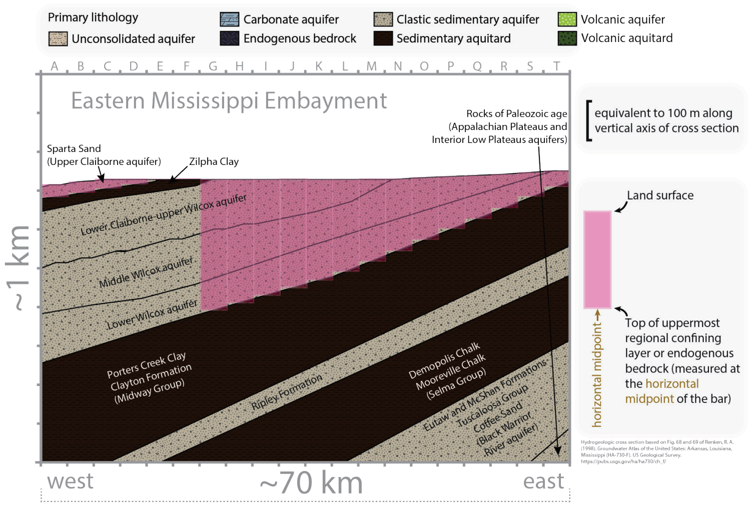

Supplementary Fig. 155. Hydrogeologic cross section. 20 equally spaced transparent pink bars overlie the cross section; each shaded bar depicts the vertical offset from the land surface to the top of the uppermost confining unit or endogenous bedrock.

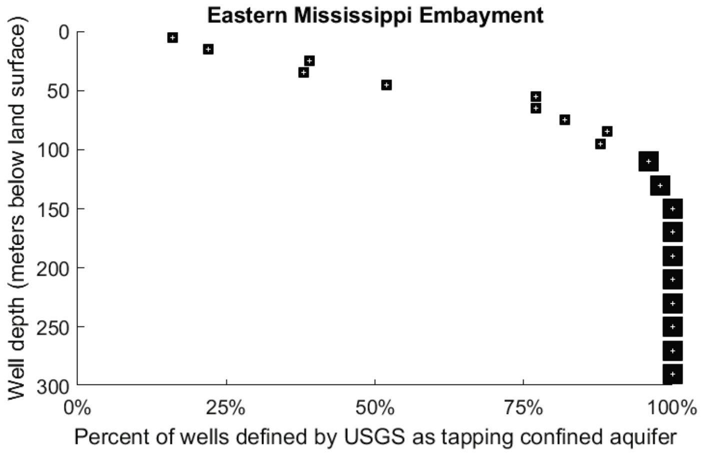

Supplementary Fig. 156. Vertical variations in the prevalence of wells that have been defined as tapping an unconfined or a confined aquifer by the USGS. The smaller squares represent 10 m depth intervals from the land surface to 100 m; the larger squares represent 20 m intervals from 100 m to 300 m below the land surface.

The Eastern Mississippi Embayment is located in central Mississippi and western Tennessee.

(i) A hydrogeologic cross section presented in Figs. 68 and 69 by Renken (1998) depicts dipping sedimentary sequences including layers classified as aquifers and layers classified as aquitards.

(ii) We analysed wells within the study area that the USGS has defined as either unconfined or confined. Most (>80%) wells at depths of 70-80 m and at depths exceeding 70 m are defined as tapping a confined aquifer.

Depth to confined conditions: 70-80 m (see (ii) above)

Reference: Renken, R. A. (1998). Groundwater Atlas of the United States: Arkansas, Louisiana, Mississippi (HA-730-F). US Geological Survey. https://pubs.usgs.gov/ha/ha730/ch_f/

The table below presents a series of published quotes (see quotation marks denoting text quoted from another publication, which is cited following the quotation marks with the full reference written in full below the table). The leftmost column lists a title of a hydrogeologic formation depicted in the cross section on the previous page. The rightmost column presents a quote from a hydrogeological study (see base of table for citation). The quote has been annotated with colored text to highlight how we categorized each layer (i.e., see categories in the center column in the table). Specifically: (i) blue text highlights portions of a quote that provide insights into the degree of consolidation of the formation, (ii) red text highlights portions of a quote that categorize the formation as an aquifer or an aquitard (i.e., higher versus lower permeability in the context of local hydrogeologic formations), and (iii) green text highlights portions of a quote that provide information about the lithology of the formation.

Supplementary Table 32. Hydrostratigraphy details for the Eastern Mississippi Embayment

Formation name	Category	Quote
Sparta Sand	Clastic sedimentary **aquifer** (consolidated or semi-consolidated rock)	"This **aquifer** underlies about 136,000 mi^2 and consists primarily of the **Sparta Sand**, which is present in most of the study area as a continentally derived **sand with clay interbeds of varying thickness and extent.**" (Hosman & Weiss 1991)
Zilpha Clay	Clastic sedimentary **aquitard** (consolidated or semi-consolidated rock)	"In much of the downdip area toward the axis of the Mississippi embayment, the Cane River Formation or its equivalents, the Tallahatta Formation (excluding the Meridian Sand Member), Winona Sand, and **Zilpha Clay,** constitute all or most of the **lower Claiborne confining unit** (table 1)." (Hosman & Weiss 1991)
Lower Claiborne-Upper Wilcox aquifer	Clastic sedimentary **aquifer** (consolidated or semi-consolidated rock)	"Most of the **aquifer** is the Carrizo Sand and its equivalent, the Meridian Sand Member of the Tallahatta Formation (table 1). The Carrizo or Meridian is an extensive **sand**, commonly **massive and unbroken by clay beds, that represents the basal unit** of the Claiborne Group." (Hosman & Weiss 1991)
Middle Wilcox aquifer	Clastic sedimentary **aquifer** (consolidated or semi-consolidated rock)	"Because the middle Wilcox **aquifer** is composed chiefly of **thin interbedded sand, silt, and clay**, it has **water-bearing characteristics different from those of typical massive and aquifers**." (Hosman & Weiss 1991)
Lower Wilcox aquifer	Clastic sedimentary **aquifer** (consolidated or semi-consolidated rock)	"This **aquifer** only occurs in the Mississippi embayment aquifer system. In the northern part of the Mississippi embayment, a **massive sand** aquifer, the Fort Pillow Sand of Tennessee, Arkansas, and Missouri (Moore and Brown, 1969), occurs in the lower to middle part of the Wilcox deposits." (Hosman & Weiss 1991)
Midway Group	Sedimentary **aquitard** (consolidated or semi-consolidated rock)	"The Midway (Paleocene) **confining unit** (pi. 18) is a **thick confining layer** that is the base of the flow system for Tertiary aquifers in most of the study area." (Hosman, & Weiss, (1991) | "The Midway consists mostly of **dense marine clays, with lesser amounts of calcareous materials in the lower part**." (Hosman & Weiss 1991)
Ripley aquifer (part of Selma Group)	Clastic sedimentary **aquifer** (consolidated or semi-consolidated rock)	"An important **aquifer** in Tippah, eastern Benton, and northern Union Counties. The McNoiry sand member ranges up to 250 feet thick and is the unit which most of the wells utilize in the Ripley. Quality is good except for hardness." (Shows, 1970) | "The Ripley Formation in Alabama and Mississippi has a maximum thickness of about 500 ft. The formation typically consists of **clay, sandy clay, sand, and thin beds of sandstone**." Mallory (1993)

Formation name	Category	Quote
Demopolis Chalk and Mooreville Chalk (part of Selma Group; *"Coffee sand" formation may be an aquifer in areas)*	Sedimentary **aquitard** (consolidated or semi-consolidated rock)	"These formations in western Alabama and eastern Mississippi, in ascending order, are the **Mooreville Chalk, the Demopolis Chalk, the Ripley Formation, and the Prairie Bluff Chalk**." Cushing et al., (1964) "**Not an aquifer.**" (referring to Demopolis **Chalk** and Mooreville **chalk**) (Shows, 1970)
Eutaw Formation	Clastic sedimentary **aquifer** (consolidated or semi-consolidated rock)	"The main body of the formation is composed of **gray clay interbedded** with fine glauconitic sand. Thin beds of fine to medium glauconitic sand are common and are fairly persistent near the base of the formation, which is normally marked by a thin bed of fine gravel. The sands are **commonly cross bedded or show distinct stratification**." Cushing et al., (1964) | "Thin beds of fine to medium glauconitic **sand** within the Eutaw and McShan Formations make up the bulk of the upper Black Warrior River regional aquifer, locally known as the **Eutaw-McShan aquifer**." Mallory (1993)
Paleozoic rocks	Sedimentary rock **aquitard** (consolidated or semi-consolidated rock)	"The lower Black Warrior River aquifer is the lowest aquifer in the Southeastern Coastal Plain aquifer system. In the northern part of the study area, the basal **confining unit** of the Southeastern Coastal Plain aquifer system consists of Paleozoic rocks. These **consolidated shales, sandstones, limestones, and dolomites** have **much smaller permeability** than the overlying Cretaceous sediments." Mallory (1993)

Renken, R. A. (1998). Ground Water Atlas of the United States: Segment 5, Arkansas, Louisiana, Mississippi (No. 730-F, pp. F1-F28). US Geological Survey. https://pubs.er.usgs.gov/publication/ha730F

Hosman, R. L., Weiss, J. S. (1991). Geohydrologic units of the Mississippi embayment and Texas coastal uplands aquifer systems, south-central United States (No. 1416-B). US Government Printing Office. https://pubs.er.usgs.gov/publication/pp1416B

Mallory, M. J. (1993). Hydrogeology of the Southeastern Coastal Plain Aquifer System in Parts of Eastern Mississippi and Western Alabama. Regional aquifer-system analysis. Southeastern coastal plain. US Geological Survey Professional Paper 1410-G, 66 pp. Accessed February 21, 2022 from https://pubs.usgs.gov/pp/1410g/report.pdf

Shows, T.N. (1970). Water resources of Mississippi. Mississippi Geological, Economic and Topographical Survey Bulletin 113, 182 pp. Accessed February 21, 2022 from https://www.mdeq.ms.gov/wp-content/uploads/2017/06/Bulletin-113.pdf

3.30 Delmarva Peninsula, North Atlantic Coastal Plain

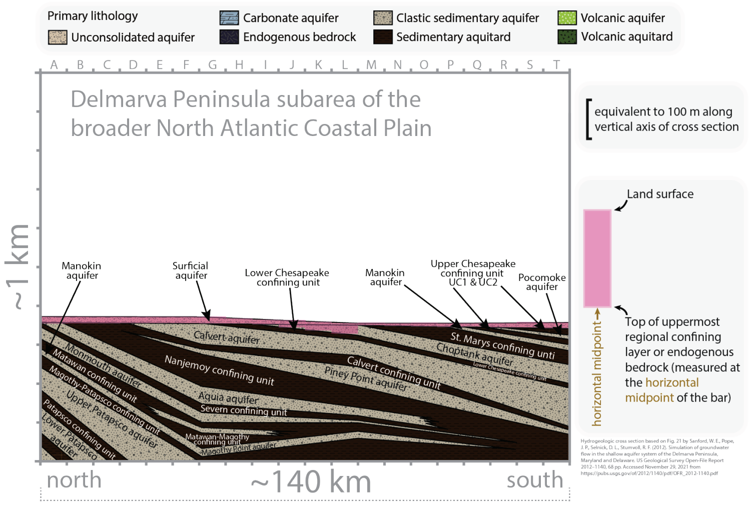

Supplementary Fig. 157. Hydrogeologic cross section. 20 equally spaced transparent pink bars overlie the cross section; each shaded bar depicts the vertical offset from the land surface to the top of the uppermost confining unit or endogenous bedrock.

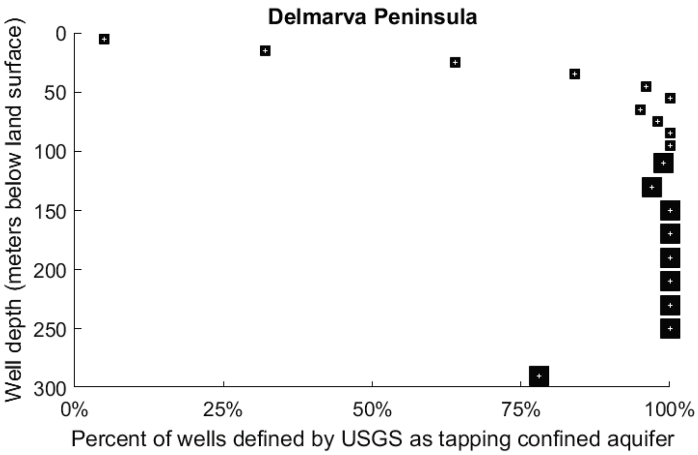

Supplementary Fig. 158. Vertical variations in the prevalence of wells that have been defined as tapping an unconfined or a confined aquifer by the USGS. The smaller squares represent 10 m depth intervals from the land surface to 100 m; the larger squares represent 20 m intervals from 100 m to 300 m below the land surface.

The Delmarva Peninsula is located in eastern Maryland and Delaware.

(i) A hydrogeologic cross section presented in Fig. 21 of Sanford et al. (2012) depicts a thin (less than ~50 m) shallow aquifer underlain by a layered aquifer system consisting of aquitards and aquifers.

(ii) We analysed wells within the study area that the USGS has defined as either unconfined or confined. Most (>80%) wells at depths of 30-40 m and at depths exceeding 30 m are defined as tapping a confined aquifer.

Depth to confined conditions: 30-40 meters below land surface (based on (ii) above)

Reference: Sanford, W. E., Pope, J. P., Selnick, D. L., Stumvoll, R. F. (2012). Simulation of groundwater flow in the shallow aquifer system of the Delmarva Peninsula, Maryland and Delaware. US Geological Survey Open-File Report 2012–1140, 68 pp. Accessed November 29, 2021 from https://pubs.usgs.gov/of/2012/1140/pdf/OFR_2012-1140.pdf

The table below presents a series of published quotes (see quotation marks denoting text quoted from another publication, which is cited following the quotation marks with the full reference written in full below the table). The leftmost column lists a title of a hydrogeologic formation depicted in the cross section on the previous page. The rightmost column presents a quote from a hydrogeological study (see base of table for citation). The quote has been annotated with colored text to highlight how we categorized each layer (i.e., see categories in the center column in the table). Specifically: (i) blue text highlights portions of a quote that provide insights into the degree of consolidation of the formation, (ii) red text highlights portions of a quote that categorize the formation as an aquifer or an aquitard (i.e., higher versus lower permeability in the context of local hydrogeologic formations), and (iii) green text highlights portions of a quote that provide information about the lithology of the formation.

Supplementary Table 33. Hydrostratigraphy details for the Delmarva Peninsula

Formation name	Category	Quote
Surficial aquifer	Unconsolidated aquifer	"The **surficial aquifer** is composed of a veneer of Upper Miocene to Holocene age **sediments** that mantle Cretaceous and **older Tertiary sediment** in Maryland and Delaware." (Vroblesky and Fleck, 1991). "*Otton* (1955, p.104) has divided the lowland deposits into **three lithologic units a basal sand and gravel, an intermediate tough clay, and an upper bed or beds of sandy clay or clayey gravel. Diatoms, marine shells, plant debris, and vivianite are common in the clay**." (Vroblesky and Fleck, 1991).
Upper Chesapeake confining unit UC1	Sedimentary **aquitard** (consolidated or semi-consolidated rock)	*The **upper Chesapeake confining unit** – The* **upper Chesapeake confining unit** (fig. 27), in the uppermost part of the Chesapeake Group, is a **discontinuous unit of lenticular silt, clay, and fine sand** separating the Upper Chesapeake aquifer from the overlying surficial aquifer downdip from the subcrop area." (Vroblesky and Fleck, 1991).
Pocomoke aquifer	Sedimentary **aquifer** (consolidated or semi-consolidated rock)	"The **upper Chesapeake aquifer** contains three major **sand bodies**. They are, from lowermost to **uppermost, the Pocomoke aquifer**, the Ocean City aquifer, and the Manokin aquifer (Weigle, 1974, p. 31-33; Hansen, 1981b)." (Vroblesky and Fleck, 1991). "The **Pocomoke aquifer** consists **of gray, fine- to medium-grained sand** and **some interbedded silt and clay**." (Vroblesky and Fleck, 1991).
Upper Chesapeake confining unit UC2	Sedimentary **aquitard** (consolidated or semi-consolidated rock)	*The **upper Chesapeake confining unit** – The* **upper Chesapeake confining unit** (fig. 27), in the uppermost part of the Chesapeake Group, is a **discontinuous unit of lenticular silt, clay, and fine sand** separating the Upper Chesapeake aquifer from the overlying surficial aquifer downdip from the subcrop area." (Vroblesky and Fleck, 1991)
Manokin aquifer	Sedimentary **aquifer** (consolidated or semi-consolidated rock)	" The **Manokin aquifer** is composed of the same general material and may contain **coarse sand and pea-sized gravel in basal units**. The Pocomoke and Manokin aquifers are separated by a sequence of **clay, silt, and fine sand**, ranging in thickness from 20 to 50 ft in Delaware (Miller, 1971, p. 14, 16)" (Vroblesky and Fleck, 1991).
St. Marys confining unit	Sedimentary **aquitard** (consolidated or semi-consolidated rock)	"The **regional confining unit** overlying the Lower Chesapeake aquifer includes **an unnamed confining unit** in New Jersey, the **Saint Marys confining unit** in Delaware, Maryland, and Virginia, and the Pungo River confining unit in North Carolina. This Miocene unit primarily **composes silt and clay** but is **diatomaceous** in New Jersey **and silty and**

Formation name	Category	Quote
		**shelly** in Delaware, Maryland, and Virginia (Trapp, 1992)." (Masterson et al., 2015).
Choptank aquifer	Sedimentary **aquifer** (consolidated or semi-consolidated rock)	"*The* **lower Chesapeake aquifer** (fig. 24) is that part of the Miocene Calvert and **Choptank Formations** in the Chesapeake Group that is **sandy** enough to **function as an aquifer**." (Vroblesky and Fleck, 1991). "The **sediments** of the lower Chesapeake aquifer consist of **medium to coarse silty sand and clay having locally abundant shells**." (Vroblesky and Fleck, 1991).
Lower Chesapeake confining unit	Sedimentary **aquitard** (consolidated or semi-consolidated rock)	"The **lower Chesapeake confining unit** consists of the **silt, clay, fine sand, and diatomaceous** earth between the Piney Point-Nanjemoy aquifer and the overlying lower Chesapeake aquifer. Point-Nanjemoy and Aquia-Rancocas aquifers beyond the updip limit of the lower Chesapeake aquifer. In eastern Maryland, the confining unit typically consists of clayey beds of the lowermost part of the Miocene Chesapeake Group (Otton, 1955, p. 90-95). " (Vroblesky and Fleck, 1991).
Calvert aquifer	Sedimentary **aquifer** (consolidated or semi-consolidated rock)	"The Lower Chesapeake regional **aquifer** includes the Lower Kirkwood-Cohansey aquifer system in New Jersey, the Milford, Frederica, Federalsburg, and Cheswold local aquifers in Delaware, the Choptank and **Calvert local aquifers** in Maryland, the Saint Marys aquifer in Virginia, and the Pungo River aquifer in North Carolina." (Masterson et al., 2015). "**The Lower Chesapeake aquifer** consists primarily of **marine sands ranging in age from Oligocene to Pliocene**. **In New Jersey, the aquifer includes interbedded sand and gravel** (Trapp, 1992)." (Masterson et al., 2015).
Calvert confining unit	Sedimentary **aquitard** (consolidated or semi-consolidated rock)	"It consists primarily of **marine clay and sandy clay** of Miocene age, and its thickness increases northward." (Masterson et al., 2015). "The **confining unit** overlying the Piney Point regional aquifer and separating it from the Lower Chesapeake aquifer above includes the Basal Kirkwood confining unit in New Jersey, the **Calvert confining unit** in Delaware, Maryland, and Virginia, and the Castle Hayne confining unit in North Carolina. It consists primarily of **marine clay and sandy clay of Miocene age**, and its thickness increases northward from less than 50 ft in North Carolina to a range of 100 to 250 ft over the northern half of the study area (Trapp, 1992)." (Masterson et al., 2015).
Piney Point aquifer	Sedimentary **aquifer** (consolidated or semi-consolidated rock)	"The Piney Point aquifer consists of **marine sediments** of mostly Eocene to Oligocene age, though it also may include sediments of Miocene age in some locations" (Masterson et al., 2015) | "a productive section of the Piney Point **aquifer** north of the James River and south of the Potomac River in Virginia composes **calcite-cemented sands and moldic limestone**." (Masterson et al., 2015)
Nanjemoy confining unit	Sedimentary **aquitard** (consolidated or semi-consolidated rock)	"A rapid marine transgression followed and is marked by the basal **clay and silt beds** of the Eocene Nanjemoy Formation." (Vroblesky and Fleck, 1991). "*The Nanjemoy-Marlboro confining unit.* ***The*** **Nanjemoy-Marlboro confining unit** is typically the **clayey** material between the sharp upper contact of the Aquia- Rancocas aquifer and the gradational

Formation name	Category	Quote
		lower contact of the Piney Point-Nanjemoy aquifer." (Vroblesky and Fleck, 1991).
Aquia aquifer	Sedimentary **aquifer** (consolidated or semi-consolidated rock)	"The Aquia **aquifer** composes permeable **marine sediments** of Paleocene age and consists primarily of medium- **to coarse grained glauconitic and fossiliferous quartz sands**." (Masterson et al., 2015)
Severn confining unit	Sedimentary **aquitard** (consolidated or semi-consolidated rock)	"The northern section of the Monmouth-Mount Laurel regional aquifer is separated from the Aquia regional aquifer above by an overlying **confining unit** that includes the Navesink-Hornerstown confining unit in New Jersey and the Severn confining unit in Delaware and Maryland. The **confining unit** consists of **marine silt, clay, and silty and clayey glauconitic sand of primarily Cretaceous age**." (Masterson et al., 2015).
Monmouth aquifer	Sedimentary **aquifer** (consolidated or semi-consolidated rock)	"The **Monmouth-Mount Laurel regional aquifer** (previously referred to by Trapp and Meisler (1992) as the Peedee-Severn aquifer) includes the Wenonah-Mount Laurel aquifer in New Jersey, the Mount Laurel aquifer in Delaware, and the **Monmouth aquifer** in Maryland (fig. 9)." (Masterson et al., 2015). "The **Monmouth-Mount Laurel aquifer** is the **uppermost regional aquifer of Late Cretaceous** age in the study area. It **includes permeable parts of the marine Mount Laurel Formation**, which is the lower part of the Monmouth Group (Andreasen and others, 2013). This aquifer consists of **very fine to coarse, slightly glauconitic sand** in New Jersey and **fine glauconitic sand** in Delaware and Maryland and ranges in thickness from about 10 to 120 ft in Delaware and Maryland and in southern New Jersey (Zapecza, 1989; Andreasen and others, 2013)." (Masterson et al., 2015).
Matawan confining unit	Sedimentary **aquifer** (consolidated or semi-consolidated rock)	"**The Matawan confining unit** – The Magothy aquifer grades upward into the **glauconitic clay and silt** of the **Matawan confining unit**." (Vroblesky and Fleck, 1991). "The **Patapsco confining unit** – The Patapsco aquifer is overlain by the **red, plastic clay** of the **Patapsco confining unit** in the upper part of the Patapsco Formation." (Vroblesky and Fleck, 1991).
Matawan-Magothy confining unit	Sedimentary **aquitard** (consolidated or semi-consolidated rock)	"Except on Long Island, the Magothy regional aquifer is overlain by **a confining unit** that separates it from the overlying Matawan aquifer. This **regional confining unit** includes the Merchantville-Woodbury confining unit in New Jersey, consisting of glauconitic and micaceous clay and silt, and the **Matawan-Magothy confining unit** in Delaware and Maryland, consisting of **silt and clay of the Magothy Formation and part of the overlying Matawan Group or Matawan Formation** (Trapp, 1992; Andreasen and others, 2013)." (Masterson et al., 2015)
Magothy aquifer	Sedimentary **aquifer** (consolidated or semi-consolidated rock)	"The Magothy **aquifer** composes **primarily sandy parts of the Magothy Formation**, which were **deposited in a transitional fluvial-marine environment during the Late Cretaceous**". (Masterson et al., 2015)

Formation name	Category	Quote
Magothy-Patapsco confining unit	Sedimentary **aquitard** (consolidated or semi-consolidated rock)	"This unit is referred to as the Raritan **Clay confining unit** in New York and New Jersey, the Magothy-Patapsco **confining unit** in Delaware and Maryland." (Masterson et al., 2015)
Upper Patapsco aquifer	Sedimentary **aquifer** (consolidated or semi-consolidated rock)	"The **Potomac-Patapsco regional aquifer** includes the Lloyd aquifer in New York (Long Island), the middle aquifer of the Potomac-Raritan-Magothy aquifer system in New Jersey, the **Upper and Lower Patapsco aquifers** of Delaware and Maryland, the undifferentiated Potomac aquifer of Virginia, and the Lower Cretaceous aquifer in North Carolina (fig. 9)." (Masterson et al., 2015). "The **Potomac-Patapsco aquifer** is similar to the underlying Potomac-Patuxent aquifer in that it consists **primarily of lenses of medium- to coarse-grained quartz sand with some gravel, interbedded with lenses of clay and silt**.". (Masterson et al., 2015). "It is **composed of fluvial-deltaic sediments** of primarily Early Cretaceous age in Maryland and Delaware, and Late Cretaceous age in New Jersey and New York." (Masterson et al., 2015). "The top of the Potomac aquifer in Virginia now correlates with the top of the Upper Patapsco **aquifer** in Maryland" (Masterson et al., 2015). "It is **composed of fluvial-deltaic sediments** of primarily Early Cretaceous age in Maryland and Delaware, and Late Cretaceous age in New Jersey and New York." (Masterson et al., 2015).
Patapsco confining unit	Sedimentary **aquitard** (consolidated or semi-consolidated rock)	"The **Patapsco confining unit** – The Patapsco aquifer is overlain by the **red, plastic clay** of the **Patapsco confining unit** in the upper part of the Patapsco Formation." (Vroblesky and Fleck, 1991).
Lower Patapsco aquifer	Sedimentary **aquifer** (consolidated or semi-consolidated rock)	"The **Potomac-Patapsco regional aquifer** includes the Lloyd aquifer in New York (Long Island), the middle aquifer of the Potomac-Raritan-Magothy aquifer system in New Jersey, the **Upper and Lower Patapsco aquifers** of Delaware and Maryland, the undifferentiated Potomac aquifer of Virginia, and the Lower Cretaceous aquifer in North Carolina (fig. 9)." (Masterson et al., 2015). "The **Potomac-Patapsco aquifer** is similar to the underlying Potomac-Patuxent aquifer in that it consists **primarily of lenses of medium- to coarse-grained quartz sand with some gravel, interbedded with lenses of clay and silt**.". (Masterson et al., 2015). "It is **composed of fluvial-deltaic sediments** of primarily Early Cretaceous age in Maryland and Delaware, and Late Cretaceous age in New Jersey and New York." (Masterson et al., 2015).

Masterson, J. P., Pope, J. P., Monti Jr, J., Nardi, M. R., Finkelstein, J. S., McCoy, K. J. (2013). Hydrogeology and hydrologic conditions of the Northern Atlantic Coastal Plain aquifer system from Long Island, New York, to North Carolina (No. 2013-5133). US Geological Survey. Accessed July 6, 2022 via https://pubs.er.usgs.gov/publication/sir20135133

Vroblesky, D.A., Fleck, W.B. (1991). Hydrogeologic Framework of the Coastal Plain of Maryland, Delaware, and the District of Columbia. U.S. Geological Survey Professional Paper 1404-E, 52 pp. Accessed April 1, 2021 from https://pubs.usgs.gov/pp/1404e/report.pdf

3.31 Maryland Western Shores, North Atlantic Coastal Plain

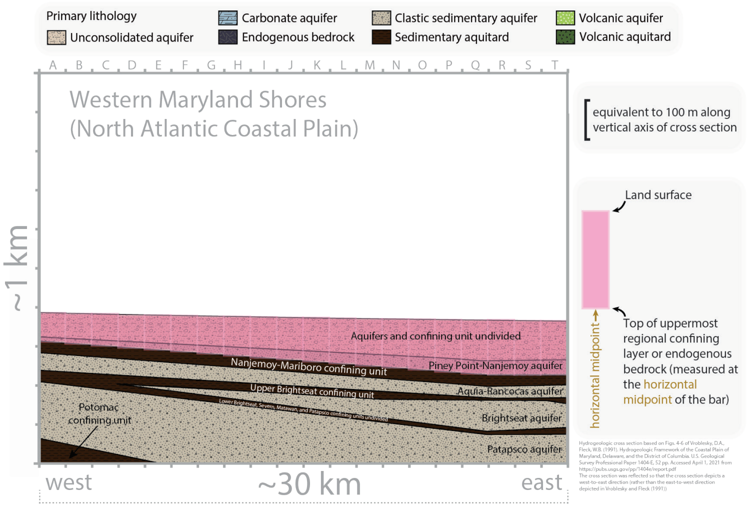

Supplementary Fig. 159. Hydrogeologic cross section. 20 equally spaced transparent pink bars overlie the cross section; each shaded bar depicts the vertical offset from the land surface to the top of the uppermost confining unit or endogenous bedrock.

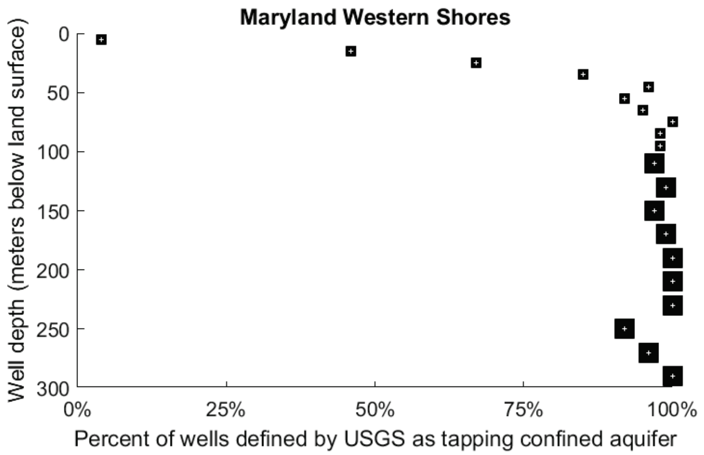

Supplementary Fig. 160. Vertical variations in the prevalence of wells that have been defined as tapping an unconfined or a confined aquifer by the USGS. The smaller squares represent 10 m depth intervals from the land surface to 100 m; the larger squares represent 20 m intervals from 100 m to 300 m below the land surface.

The Maryland Western Shores area is located in central Maryland.

(i) A hydrogeologic cross section presented in Figs. 4-6 by Vroblesky et al. (1991) depicts a shallow geologic formation with both permeable and less-permeable subunits (undivided).

(ii) We analysed wells within the study area that the USGS has defined as either unconfined or confined. Most (>80%) wells at depths of 30-40 m and at depths exceeding 30 m are defined as tapping a confined aquifer.

Depth to confined conditions: 30-40 meters below land surface (based on (ii) above)

Reference: Vroblesky, D.A., Fleck, W.B. (1991). Hydrogeologic Framework of the Coastal Plain of Maryland, Delaware, and the District of Columbia. U.S. Geological Survey Professional Paper 1404-E, 52 pp. Accessed April 1, 2021 from https://pubs.usgs.gov/pp/1404e/report.pdf

The table below presents a series of published quotes (see quotation marks denoting text quoted from another publication, which is cited following the quotation marks with the full reference written in full below the table). The leftmost column lists a title of a hydrogeologic formation depicted in the cross section on the previous page. The rightmost column presents a quote from a hydrogeological study (see base of table for citation). The quote has been annotated with colored text to highlight how we categorized each layer (i.e., see categories in the center column in the table). Specifically: (i) blue text highlights portions of a quote that provide insights into the degree of consolidation of the formation, (ii) red text highlights portions of a quote that categorize the formation as an aquifer or an aquitard (i.e., higher versus lower permeability in the context of local hydrogeologic formations), and (iii) green text highlights portions of a quote that provide information about the lithology of the formation.

Supplementary Table 34. Hydrostratigraphy details for the Maryland Western Shores

Formation name	Category	Quote
Aquifers and confining unit undivided	Unconsolidated aquifer	“The **surficial aquifer** is composed of a veneer **of Upper Miocene to Holocene age sediments** that mantle Cretaceous and older Tertiary sediment in Maryland and Delaware.” (Vroblesky and Fleck, 1991). “Otton (1955, p. 104) has divided the **lowland deposits** into **three lithologic units a basal sand and gravel, an intermediate tough clay, and an upper bed or beds of sandy clay or clayey gravel**.” (Vroblesky and Fleck, 1991). “The **St. Marys confining unit** (fig. 25), which overlies the lower Chesapeake aquifer, is composed of **gray clay, clayey silt, and very fine sand of the Miocene** St. **Marys Formation** in the middle part of the Chesapeake Group.” (Vroblesky and Fleck, 1991). “The **upper Chesapeake aquifer** contains **three major sand bodies**.” (Vroblesky and Fleck, 1991). “The **lower Chesapeake aquifer** is a multilayer aquifer.” (Vroblesky and Fleck, 1991). “it is difficult to distinguish the **lower Chesapeake aquifer** from the overlying **surficial aquifer** in subcrop areas because of similar lithologies, although the lower Chesapeake aquifer sediments have been reported to be **grayer and better sorted** (Sundstrom and Pickett, 1969, p. 17-20).” (Vroblesky and Fleck, 1991).
Piney Point-Nanjemoy aquifer	Sedimentary **aquifer** (consolidated or semi-consolidated rock)	“The Piney Point aquifer consists of **marine sediments** of mostly Eocene to Oligocene age, though it also may include sediments of Miocene age in some locations” (Masterson et al., 2015) | “a productive section of the Piney Point **aquifer** north of the James River and south of the Potomac River in Virginia composes **calcite-cemented sands and moldic limestone**.” (Masterson et al., 2015)
Nanjemoy-Mariboro confining unit	Sedimentary **aquitard** (consolidated or semi-consolidated rock)	“The regional **confining unit** includes the Vincentown Manasquan confining unit in New Jersey; the **Nanjemoy-Marlboro Clay confining unit** in Delaware, Maryland, and Virginia; and the Beaufort confining unit in North Carolina.” (Masterson et al., 2015) | “The **confining unit** overlaying the Aquia aquifer is made of **marine silt, clay, and sandy clay** ranging from a thickness of 50 ft in North Carolina to more than 900 ft in New Jersey (Trapp, 1992).” (Masterson et al., 2015)
Aquia-Rancocas aquifer	Sedimentary **aquifer** (consolidated or semi-consolidated rock)	“The **Aquia-Rancocas aquifer** is composed of the **sandy portions of the Paleocene Aquia Formation** in Maryland and the Paleocene Rancocas Group in Delaware. In places, it may include **sandy** portions of the underlying Brightseat Formation.” (Vroblesky and Fleck, 1991). “The **Aquia-Rancocas aquifer** is predominantly **glauconitic and quartzose, medium- to coarse-grained, and medium- to well-sorted sand** (Chapelle and Drummond, 1983, p. 7).” (Vroblesky and Fleck, 1991).
Upper Brightseat	Sedimentary **aquitard**	“The **upper Brightseat confining unit** – **The upper Brightseat confining unit**, in what may be the upper part of the Brightseat

Formation name	Category	Quote
confining unit	(consolidated or semi-consolidated rock)	Formation (Bennett and Collins, 1952), overlies the Brightseat aquifer." (Vroblesky and Fleck, 1991). "The **confining unit** is composed of **greenish-gray to black, glauconitic silt and clay, having interbedded glauconitic, fine-grained sand** (Weigle and Webb, 1970, p. 32). (Vroblesky and Fleck, 1991).
Brightseat aquifer	Sedimentary **aquifer** (consolidated or semi-consolidated rock)	"The **Brightseat aquifer** is restricted to the southernmost part of the study area. It includes **sediments that are located primarily in St. Marys County, Md.,** that earlier studies assigned to the Magothy aquifer (Hansen, 1972, p. 47; Weigle and Webb, 1970, p. 32)." (Vroblesky and Fleck, 1991). "In St. Marys County, the **aquifer** is composed of **very fine to fine, light-gray to yellowish or purple quartzose sand and muscovite, lignite, and minor glauconite** (Weigle and Webb, 1970, p 32)." (Vroblesky and Fleck, 1991).
Lower Brightseat, Severn, Matawan, and Patapsco confining units undivided	Sedimentary **aquitard** (consolidated or semi-consolidated rock)	"The **lower Brightseat confining unit** – **The lower Brightseat confining unit** typically **consists of the silt and clay** between the Severn aquifer and the overlying Aquia-Rancocas aquifer." (Vroblesky and Fleck, 1991). "The **Severn confining unit** – The **Severn confining unit**, in the lower part of the Upper Cretaceous Severn Formation in Maryland, is composed of the **clay and silt** between the Matawan aquifer and the overlying Severn aquifer." (Vroblesky and Fleck, 1991). "**The Matawan confining unit** – The Magothy aquifer grades upward into the **glauconitic clay and silt** of the **Matawan confining unit**." (Vroblesky and Fleck, 1991). "The **Patapsco confining unit** – The Patapsco aquifer is overlain by the **red, plastic clay** of the **Patapsco confining unit** in the upper part of the Patapsco Formation." (Vroblesky and Fleck, 1991).
Patapsco aquifer	Sedimentary **aquifer** (consolidated or semi-consolidated rock)	"The sediments of the **Patapsco aquifer** are typically **white to yellow, crossbedded, fine to medium, clayey sand and subordinate amounts of gravel**. **Associated clay is dense, massive or laminated, and variegated in shades of red, gray, brown, and purple** (Glaser, 1969, p. 9). The **aquifer** is predominantly sand in Anne Arundel County." (Vroblesky and Fleck, 1991). "The top of the Potomac aquifer in Virginia now correlates with the top of the Upper Patapsco **aquifer** in Maryland" (Masterson et al., 2015). "It is **composed of fluvial-deltaic sediments** of primarily Early Cretaceous age in Maryland and Delaware, and Late Cretaceous age in New Jersey and New York." (Masterson et al., 2015).
Potomac confining unit	Sedimentary **aquitard** (consolidated or semi-consolidated rock)	"The **Potomac confining unit** – The **Potomac confining unit** overlies the Patuxent aquifer. (Vroblesky and Fleck, 1991). "**Potomac confining unit generally** corresponds to a zone of **clay and sand lenses** separating two predominantly sandy zones in the Potomac Group (or Formation), as described by Sundstrom and others (1967, p. 21). (Vroblesky and Fleck, 1991).

Masterson, J. P., Pope, J. P., Monti Jr, J., Nardi, M. R., Finkelstein, J. S., McCoy, K. J. (2015). Hydrogeology and hydrologic conditions of the Northern Atlantic Coastal Plain aquifer system from Long Island, New York, to North Carolina (No. 2013-5133). US Geological Survey. Accessed August 10, 2022 via https://pubs.usgs.gov/sir/2013/5133/pdf/sir20135133.pdf

Vroblesky, D.A., Fleck, W.B. (1991). Hydrogeologic Framework of the Coastal Plain of Maryland, Delaware, and the District of Columbia. U.S. Geological Survey Professional Paper 1404-E, 52 pp. Accessed April 1, 2021 from https://pubs.usgs.gov/pp/1404e/report.pdf

3.32 New Jersey Coastal Plain, North Atlantic Coastal Plain

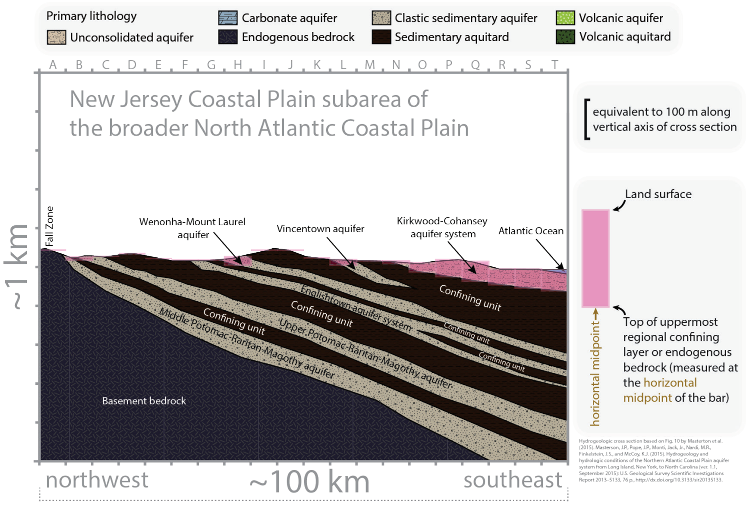

Supplementary Fig. 161. Hydrogeologic cross section. 20 equally spaced transparent pink bars overlie the cross section; each shaded bar depicts the vertical offset from the land surface to the top of the uppermost confining unit or endogenous bedrock.

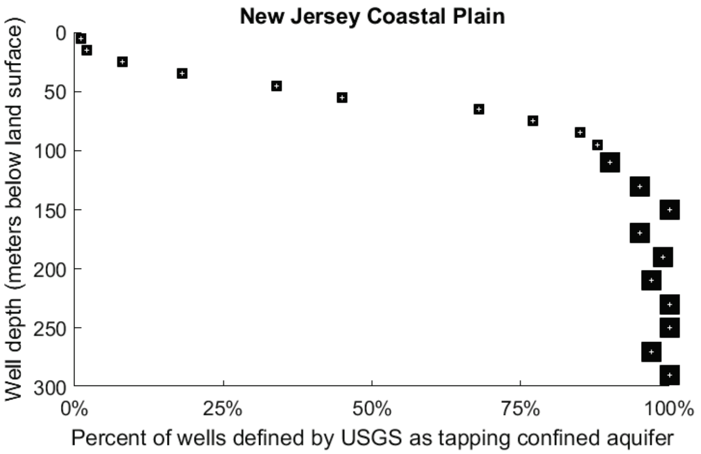

Supplementary Fig. 162. Vertical variations in the prevalence of wells that have been defined as tapping an unconfined or a confined aquifer by the USGS. The smaller squares represent 10 m depth intervals from the land surface to 100 m; the larger squares represent 20 m intervals from 100 m to 300 m below the land surface.

The New Jersey Coastal Plain is located in eastern New Jersey.

(i) A hydrogeologic cross section presented in Fig. 10 of Masterson et al. (2013) shows a layered aquifer system including clastic sedimentary aquifers and aquitards of varying thicknesses.

(ii) We analysed wells within the study area that the USGS has defined as either unconfined or confined. Most (>80%) wells at depths of 80-90 m and at depths exceeding 80 m are defined as tapping a confined aquifer.

Depth to confined conditions: 80-90 meters below land surface (based on (ii) above)

Reference: Masterson, J.P., Pope, J.P., Monti, Jack, Jr., Nardi, M.R., Finkelstein, J.S., McCoy, K.J. (2013). Hydrogeology and hydrologic conditions of the Northern Atlantic Coastal Plain aquifer system from Long Island, New York, to North Carolina. US Geological Survey Scientific Investigations Report 2013–5133, 88 pp. Accessed June 13, 2022 via http://dx.doi.org/10.3133/sir20135133

The table below presents a series of published quotes (see quotation marks denoting text quoted from another publication, which is cited following the quotation marks with the full reference written in full below the table). The leftmost column lists a title of a hydrogeologic formation depicted in the cross section on the previous page. The rightmost column presents a quote from a hydrogeological study (see base of table for citation). The quote has been annotated with colored text to highlight how we categorized each layer (i.e., see categories in the center column in the table). Specifically: (i) blue text highlights portions of a quote that provide insights into the degree of consolidation of the formation, (ii) red text highlights portions of a quote that categorize the formation as an aquifer or an aquitard (i.e., higher versus lower permeability in the context of local hydrogeologic formations), and (iii) green text highlights portions of a quote that provide information about the lithology of the formation.

Supplementary Table 35. Hydrostratigraphy details for the New Jersey Coastal Plain

Formation name	Category	Quote
Kirkwood-Cohansey aquifer system	Unconsolidated aquifer	"**unconfined Kirkwood-Cohansey aquifer** is hydrologically equivalent to the **Surficial aquifer** as described in other States, and therefore **could be grouped with the regional Surficial regional aquifer**." (Masterson et al., 2015). "The **Upper Chesapeake aquifer includes the Upper Kirkwood-Cohansey aquifer** in New Jersey" (Masterson et al., 2015). "This aquifer consists of **permeable sediments** of the upper part of the Miocene to Pliocene-age Chesapeake Group. The aquifer composes primarily **sands of marine origin** in North Carolina and Virginia but transitions northward to New Jersey into **coarser sands and gravels of fluvial origin** (Trapp, 1992)." (Masterson et al., 2015). "The Lower Chesapeake **regional aquifer** includes the **Lower Kirkwood-Cohansey aquifer** system in New Jersey". (Masterson et al., 2015). "The Lower Chesapeake aquifer consists primarily of **marine sands ranging in age from Oligocene to Pliocene. In New Jersey, the aquifer includes interbedded sand and gravel** (Trapp, 1992)." (Masterson et al., 2015).
Confining unit	Sedimentary **aquitard** (consolidated or semi-consolidated rock)	"The **regional confining unit** overlying the Lower Chesapeake aquifer includes **an unnamed confining unit** in New Jersey, the Saint Marys confining unit in Delaware, Maryland, and Virginia, and the Pungo River confining unit in North Carolina. This Miocene unit primarily **composes silt and clay** but is **diatomaceous** in New Jersey **and silty and shelly** in Delaware, Maryland, and Virginia (Trapp, 1992)." (Masterson et al., 2015).
Vincentown aquifer	Sedimentary **aquifer** (consolidated or semi-consolidated rock)	"The **Aquia regional aquifer** (previously referred to by Trapp and Meisler (1992) as the Beaufort-Aquia aquifer) includes the **Vincentown aquifer** in New Jersey, the Rancocas aquifer in Delaware, the Aquia aquifer in Maryland and Virginia, and the Beaufort aquifer in North Carolina (fig. 9). ". (Masterson et al., 2015). " The **Aquia aquifer** composes **permeable marine sediments** of Paleocene age and consists primarily of **medium- to coarse-grained glauconitic and fossiliferous quartz sands** (Trapp, 1992)." (Masterson et al., 2015).
Confining unit	Sedimentary **aquitard** (consolidated or semi-consolidated rock)	"The **regional confining unit** overlying the Lower Chesapeake aquifer includes **an unnamed confining unit** in New Jersey, the Saint Marys confining unit in Delaware, Maryland, and Virginia, and the Pungo River confining unit in North Carolina. This Miocene unit primarily **composes silt and clay** but is **diatomaceous** in New Jersey **and silty and**

Formation name	Category	Quote
		**shelly** in Delaware, Maryland, and Virginia (Trapp, 1992)." (Masterson et al., 2015).
Wenonah-Mount Laurel aquifer	Sedimentary **aquifer** (consolidated or semi-consolidated rock)	"The **Monmouth-Mount Laurel regional aquifer** (previously referred to by Trapp and Meisler (1992) as the Peedee-Severn aquifer) includes the **Wenonah-Mount Laurel aquifer** in New Jersey, the Mount Laurel aquifer in Delaware, and the Monmouth aquifer in Maryland (fig. 9). ". (Masterson et al., 2015). " The **Monmouth-Mount Laurel aquifer** is the uppermost regional aquifer of Late Cretaceous age in the study area. It includes **permeable parts of the marine Mount Laurel Formation**, which is the lower part of the Monmouth Group (Andreasen and others, 2013). This aquifer consists of **very fine to coarse, slightly glauconitic sand** in New Jersey". (Masterson et al., 2015).
Confining unit	Sedimentary **aquitard** (consolidated or semi-consolidated rock)	"The **regional confining unit** overlying the Lower Chesapeake aquifer includes **an unnamed confining unit** in New Jersey, the Saint Marys confining unit in Delaware, Maryland, and Virginia, and the Pungo River confining unit in North Carolina. This Miocene unit primarily **composes silt and clay** but is **diatomaceous** in New Jersey **and silty and shelly** in Delaware, Maryland, and Virginia (Trapp, 1992)." (Masterson et al., 2015).
Englishtown aquifer system	Sedimentary **aquifer** (consolidated or semi-consolidated rock)	" The northern section of the **Matawan aquifer** includes the **Englishtown aquifer** in New Jersey " (Masterson et al., 2015). " The **Matawan aquifer** primarily consists of **sands deposited by a marine transgression during the Late Cretaceous,** including **fine to medium quartz sand in New Jersey and fine silty to clayey sand** in Delaware and Maryland. ". (Masterson et al., 2015).
Confining unit	Sedimentary **aquitard** (consolidated or semi-consolidated rock)	"The **regional confining unit** overlying the Lower Chesapeake aquifer includes **an unnamed confining unit** in New Jersey, the Saint Marys confining unit in Delaware, Maryland, and Virginia, and the Pungo River confining unit in North Carolina. This Miocene unit primarily **composes silt and clay** but is **diatomaceous** in New Jersey **and silty and shelly** in Delaware, Maryland, and Virginia (Trapp, 1992)." (Masterson et al., 2015).
Upper Potomac-Raritan-Magothy aquifer *(The aquifer is dominantly confined aquifer)*	Sedimentary **aquifer** (consolidated or semi-consolidated rock)	"The local aquifer previously considered part of the **Upper Potomac aquifer** now are part of the regional **Magothy aquifer**. These units include the **Upper Potomac-Raritan-Magothy** aquifer in New Jersey". (Masterson et al., 2015). " The **Magothy aquifer** composes **primarily sandy parts of the Magothy Formation**, which were **deposited in a transitional fluvial-marine environment during the Late Cretaceous** (Andreasen and others, 2013). The **Magothy aquifer** consists of **very fine to medium quartz sand, with discontinuous layers of carbonaceous clayey silt; it also contains coarse to very coarse sand and gravel**, particularly in the thicker parts (Trapp, 1992).". (Masterson et al., 2015).
Confining unit	Sedimentary **aquitard** (consolidated or	"The **regional confining unit** overlying the Lower Chesapeake aquifer includes **an unnamed confining unit** in New Jersey, the Saint Marys confining unit in Delaware, Maryland, and Virginia, and the Pungo River confining unit in

Formation name	Category	Quote
	semi-consolidated rock)	North Carolina. This Miocene unit primarily **composes silt and clay** but is **diatomaceous** in New Jersey **and silty and shelly** in Delaware, Maryland, and Virginia (Trapp, 1992)." (Masterson et al., 2015).
Middle Potomac-Raritan-Magothy aquifer	Sedimentary **aquifer** (consolidated or semi-consolidated rock)	" In New Jersey, the **Potomac aquifer system** is subdivided into the **Middle Potomac-Raritan-Magothy** and Lower Potomac-Raritan-Magothy aquifers " (Masterson et al., 2015). "**Unconsolidated Cretaceous-age sediments** of **fluvial-deltaic origin** comprise an aquifer system that is the thickest, deepest, and most important **source of groundwater** throughout the NACP. This **Potomac aquifer system** (Potomac-Patuxent and Potomac-Patapsco aquifers, fig. 9) overlies basement bedrock". (Masterson et al., 2015).
Basement bedrock	Endogenous rock	"In the inner part of the crater, the Exmore clast **confining unit** directly overlies the altered **bedrock basement** and reaches its **maximum thickness of more than 4,500 ft.**". (Masterson et al., 2015).

Masterson, J.P., Pope, J.P., Monti, Jack, Jr., Nardi, M.R., Finkelstein, J.S., McCoy, K.J. (2015). Hydrogeology and hydrologic conditions of the Northern Atlantic Coastal Plain aquifer system from Long Island, New York, to North Carolina. U.S. Geological Survey Scientific Investigations Report 2013–5133, 76 p., http://dx.doi.org/10.3133/sir20135133

3.33 North Carolina and Virginia Coastal Plain, North Atlantic Coastal Plain

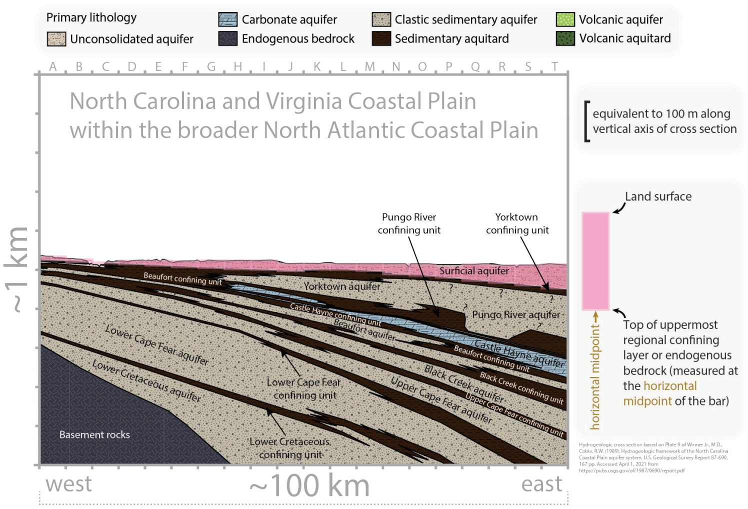

Supplementary Fig. 163. Hydrogeologic cross section. 20 equally spaced transparent pink bars overlie the cross section; each shaded bar depicts the vertical offset from the land surface to the top of the uppermost confining unit or endogenous bedrock.

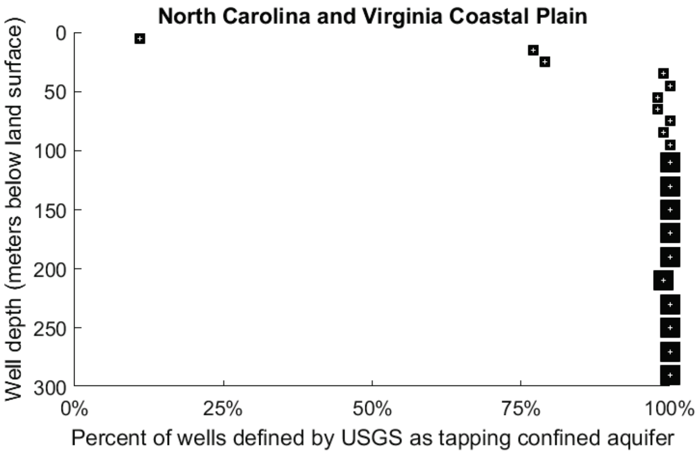

Supplementary Fig. 164. Vertical variations in the prevalence of wells that have been defined as tapping an unconfined or a confined aquifer by the USGS. The smaller squares represent 10 m depth intervals from the land surface to 100 m; the larger squares represent 20 m intervals from 100 m to 300 m below the land surface.

The North Carolina and Virginia Coastal Plain subarea of the broader North Atlantic Coastal Plain is located in northeastern North Carolina and southeastern Virginia.

(i) A hydrogeologic cross section presented in Plate 9 by Winner Jr. and Coble (1989) depicts a shallow suficial aquifer underlain by a confining unit at depths of less than ~50 m below the land surface.

(ii) We analysed wells within the study area that the USGS has defined as either unconfined or confined. Most (>80%) wells at depths of 30-40 m and at depths exceeding 30 m are defined as tapping a confined aquifer.

Depth to confined conditions: 30-40 meters below land surface (based on (ii) above)

Reference: Winner Jr., M.D., Coble, R.W. (1989). Hydrogeologic framework of the North Carolina Coastal Plain aquifer system. U.S. Geological Survey Report 87-690, 167 pp. Accessed April 1, 2021 from https://pubs.usgs.gov/of/1987/0690/report.pdf

The table below presents a series of published quotes (see quotation marks denoting text quoted from another publication, which is cited following the quotation marks with the full reference written in full below the table). The leftmost column lists a title of a hydrogeologic formation depicted in the cross section on the previous page. The rightmost column presents a quote from a hydrogeological study (see base of table for citation). The quote has been annotated with colored text to highlight how we categorized each layer (i.e., see categories in the center column in the table). Specifically: (i) blue text highlights portions of a quote that provide insights into the degree of consolidation of the formation, (ii) red text highlights portions of a quote that categorize the formation as an aquifer or an aquitard (i.e., higher versus lower permeability in the context of local hydrogeologic formations), and (iii) green text highlights portions of a quote that provide information about the lithology of the formation.

Supplementary Table 36. Hydrostratigraphy details for the North Carolina and Virginia Coastal Plain

Formation name	Category	Quote
Surficial aquifer	Unconsolidated aquifer	"**The surficial aquifer** (A10) overlies all of the North Carolina Coastal Plain (fig. 1) and consists of **fine sand, silt, clay, shell, and peat beds**. **Scattered deposits of coarser-grained sediments in the unit occur in relict beach ridges or in alluvium**." (Giese et al., 1997)
Yorktown confining unit	Sedimentary **aquitard** (consolidated or semi-consolidated rock)	"The **Yorktown confining unit** (CU9) overlying the Yorktown aquifer is comprised of the **youngest clay beds** of the Yorktown Formation in most places, but locally may **include clay beds** of Pleistocene or Holocene age. Its **thickness averages about 25 ft,** ranging from less than 10 up to 50 ft thick. It is composed largely **of clay and sandy clay** that locally includes beds of fine sand or shell." (Giese et al., 1997)
Yorktown aquifer	Sedimentary **aquifer** (consolidated or semi-consolidated rock)	"**The Yorktown aquifer** largely consists of **fine sand, silty and clayey sand, sand with shells and shell beds, some limestone, and** some **coarse sand beds**." (Giese et al., 1997)
Pungo River confining unit	Sedimentary **aquitard** (consolidated or semi-consolidated rock)	"The **Pungo River confining unit (CU8)** is formed **by the upper clay beds of the Pungo River Formation and contiguous clays of the lowermost Yorktown Formation**. The **confining unit** ranges in thickness from less than 10 ft near the western margin to about 150 ft beneath Currituck County, with an average thickness of nearly 55 ft." (Giese et al., 1997)
Pungo River aquifer	Sedimentary **aquifer** (consolidated or semi-consolidated rock)	"The **Pungo River aquifer** is composed of **fine to medium marine sands** having considerable phosphate content.". (Giese et al., 1997). "The Pungo River aquifer (A8) is thinnest near its western and northern limits, **where its thickness averages about 15 ft**. The aquifer dips eastward and **thickens to more than 200 ft in the vicinity of the** Outer Banks, **where the top is deeper than 700 ft below sea level**." (Giese et al., 1997)
Castle Hayne confining unit	Sedimentary **aquitard** (consolidated or semi-consolidated rock)	"The thickness of the **Castle Hayne confining unit (CU7) averages only about 10 ft; it exceeds 25 ft only** in Gates County along the Virginia border, in eastern Pamlico and Carteret Counties, and in two small areas along the western limit of the Castle Hayne aquifer (A7). **The confining unit** is composed of **beds of clay, sandy clay, and clay with sandy streaks that are part of the Purgo River Formation, the**

Formation name	Category	Quote
		**Yorktown Formation, or younger clays**." (Giese et al., 1997)
Castle Hayne aquifer	Carbonate aquifer	"The **Castle Hayne aquifer** (A7) consists of **limestone, sand, and minor amounts of clay deposited under marine conditions**. **Limestone may occur as shell limestone, dolomitic limestone, and sandy limestone ranging from loosely consolidated to hard and recrystallized**." (Giese et al., 1997)
Beaufort confining unit,	Sedimentary **aquitard** (consolidated or semi-consolidated rock)	"**The Beaufort confining unit (CU6**) consists of the uppermost sediments of the Beaufort Formation and possibly **some younger clay, silt, and sandy clay**. Over most of the area, the confining unit shows a **gradation** from **sandy clay to clay**, but contains **distinct clay beds interlayered** with **fine sand or silt**." (Giese et al., 1997)
Beaufort aquifer	Sedimentary **aquifer** (consolidated or semi-consolidated rock)	"The **Beaufort aquifer** (A6) consists of **fine to medium glauconitic sands, clayey sands, and clay beds of marine origin**. **Shell and limestone beds** are present but are less than 6 ft thick." (Giese et al., 1997)
Black Creek confining unit	Sedimentary **aquitard** (consolidated or semi-consolidated rock)	"The **Black Creek confining unit** (CU4) is primarily composed of the uppermost **beds** of the **Black Creek Formation and consists of clay, silty clay, and sandy clay**." (Giese et al., 1997)
Black Creek aquifer	Sedimentary **aquifer** (consolidated or semi-consolidated rock)	"**The Black Creek aquifer** (A4) **contains Upper Cretaceous sediments** of both the Black Creek and underlying Middendorf Formations (Winner and Coble, 1989, 1996). **The Black Creek Formation consists mainly of thinly laminated gray to black clay, interbedded with gray to tan sands**. **Outcrops also exhibit sand- or clay-dominated lenses**." (Giese et al., 1997)
Upper Cape Fear confining unit	Sedimentary **aquitard** (consolidated or semi-consolidated rock)	"As described by Winner and Coble (1989), the **upper Cape Fear confining unit** (CU3) **consists of nearly continuous clay**, **silty clay, and sandy clay beds belonging** either to the Middendorf Formation in the Sand Hills area or to the Black Creek Formation." (Giese et al., 1997)
Upper Cape Fear aquifer	Sedimentary **aquifer** (consolidated or semi-consolidated rock)	"The **sediments** of the **upper Cape Fear aquifer** (A3), (fig. 18) are alternating **beds of sand and clay**." (Giese et al., 1997)
Lower Cape Fear confining unit	Sedimentary **aquitard** (consolidated or semi-consolidated rock)	"The **lower Cape Fear confining unit (CU2)** is composed **of clay and sandy-clay beds that belong largely to the Cape Fear Formation**. **The average thickness of the confining unit is about 50 ft**." (Giese et al., 1997)
Lower Cape Fear aquifer	Sedimentary **aquifer** (consolidated or semi-consolidated rock)	"The **lower Cape Fear aquifer** (A2) strikes northeast and dips southwest at a rate of 15 to 35 ft/mi. Its extent is shown in figure 20. Its **thickness ranges from a few feet along its western margin to more than 400 ft** in the north- eastern North Carolina Coastal Plain." (Giese et al., 1997). "The **Cape Fear aquifer consists predominantly of sand, silt, and gravel separated by relatively thick silt and clay layers**." (Aucott, 1996)

Formation name	Category	Quote
Lower Cretaceous confining unit	Sedimentary **aquitard** (consolidated or semi-consolidated rock)	"The **Lower Cretaceous confining unit** (CU1**) consists of clay and sandyclay beds** that belong to either **sediments of Early Cretaceous or Late Cretaceous age**. The thickness of the unit averages about 46 ft but is nearly 70 ft in Camden and Currituck Counties. The Lower Cretaceous aquifer and **confining unit** are overlain everywhere by the lower Cape Fear aquifer (A2) and underlain everywhere by crystalline basement rocks (Winner and Coble, 1989)." (Giese et al., 1991).
Lower Cretaceous aquifer	Sedimentary **aquifer** (consolidated or semi-consolidated rock)	"Various investigators have established that the updip beds of the **Lower Cretaceous aquifer** are largely **nonmarine in origin**, but the incidence of **beds of marine origin increases** downdip toward the coast. **The non- marine beds are shales, sands, and gravel. Marine beds are chiefly limestones that may be sandy or dolomitic**." (Giese et al., 1997).
Basement Rocks	Endogenous bedrock	"The Lower Cretaceous aquifer and confining unit are overlain everywhere by the lower Cape Fear aquifer (A2) and are underlain everywhere by **crystalline basement rocks**". (Giese et al., 1997). "The unconsolidated Coastal Plain aquifer system is underlain by **crystalline basement** rocks of **low permeability**." (Giese et al., 1991).

Giese G.L., Eimers J.L., Coble R.W. (1997). Simulation of ground water flow in the Coastal Plain aquifer system of North Carolina. US Geological Survey Professional Paper 1404-M. 142 p. accessed on 3/29/2022 via https://pubs.usgs.gov/pp/1404m/report.pdf

Aucott, W.R. (1996). *Hydrology of the Southeastern Coastal Plain aquifer system in South Carolina and parts of Georgia and North Carolina* (No. 1410-E). US Geological Survey. https://pubs.er.usgs.gov/publication/pp1410E

Giese, G.L., Eimers, J.L., Coble, R.W. (1991). *Simulation of ground-water flow in the Coastal Plain aquifer system of North Carolina* (Vol. 1404). US Government Printing Office. Accessed on 3/29/2022 via https://pubs.er.usgs.gov/publication/ofr90372

3.34 Powder River Basin, Northern Great Plains

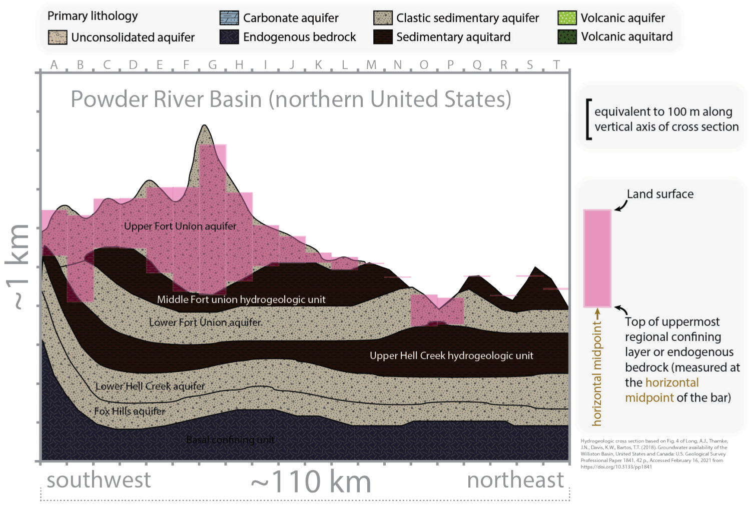

Supplementary Fig. 165. Hydrogeologic cross section. 20 equally spaced transparent pink bars overlie the cross section; each shaded bar depicts the vertical offset from the land surface to the top of the uppermost confining unit or endogenous bedrock.

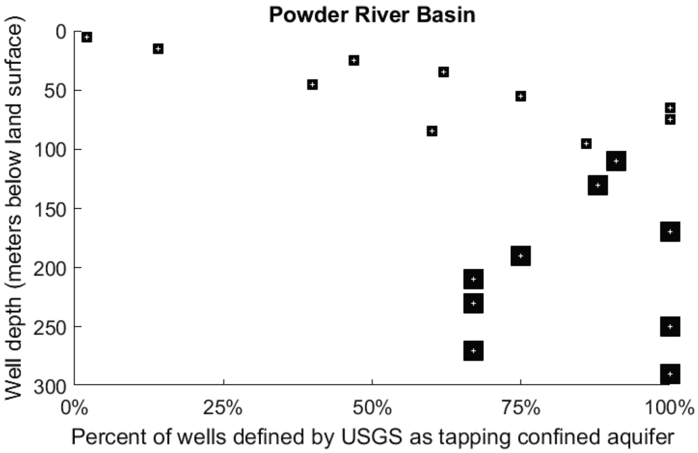

Supplementary Fig. 166. Vertical variations in the prevalence of wells that have been defined as tapping an unconfined or a confined aquifer by the USGS. The smaller squares represent 10 m depth intervals from the land surface to 100 m; the larger squares represent 20 m intervals from 100 m to 300 m below the land surface.

The Powder River Basin is located in northeastern Wyoming and southeastern Montana.

(i) A hydrogeologic cross section presented in Fig. 4 by Long et al. (2018) depicts relatively shallow confining units in some areas, and deeper depths to the uppermost confining unit in other areas.

(ii) We analysed wells within the study area that the USGS has defined as either unconfined or confined. Most (>80%) wells at depths of 60-70 m and at depths exceeding 60 m are defined as tapping a confined aquifer.

Depth to confined conditions: 60-70 m (see (ii) above)

Reference: Long, A.J., Thamke, J.N., Davis, K.W., Bartos, T.T. (2018). Groundwater availability of the Williston Basin, United States and Canada: U.S. Geological Survey Professional Paper 1841, 42 p., Accessed February 16, 2021 from https://doi.org/10.3133/pp1841

The table below presents a series of published quotes (see quotation marks denoting text quoted from another publication, which is cited following the quotation marks with the full reference written in full below the table). The leftmost column lists a title of a hydrogeologic formation depicted in the cross section on the previous page. The rightmost column presents a quote from a hydrogeological study (see base of table for citation). The quote has been annotated with colored text to highlight how we categorized each layer (i.e., see categories in the center column in the table). Specifically: (i) blue text highlights portions of a quote that provide insights into the degree of consolidation of the formation, (ii) red text highlights portions of a quote that categorize the formation as an aquifer or an aquitard (i.e., higher versus lower permeability in the context of local hydrogeologic formations), and (iii) green text highlights portions of a quote that provide information about the lithology of the formation.

Supplementary Table 37. Hydrostratigraphy details for the Powder River Basin

Formation name	Category	Quote
Upper Fort Union aquifer	Clastic sedimentary **aquifer** (consolidated or semi-consolidated rock)	"The **upper Fort Union aquifer** is **as thick as 1,920 ft and composed of crossbedded light-yellow to light-yellow-gray sandstone, sandy mudstone, gray shale, carbonaceous shale, and thick coal beds and associated clinker deposits** (permeable rocks created by the natural burning of coal beds)." (Long et al., 2018).
Middle Fort union hydrogeologic unit *(in some areas can be aquifer)*	Sedimentary **aquitard** (consolidated or semi-consolidated rock)	"**Composed of as much as 520 ft of thickness of alternating beds** of **sandstone, siltstone, mudstone, claystone, and lignite, rocks in the middle Fort Union hydrogeologic unit generally are finer-grained and darker-colored than the overlying upper Fort Union aquifer and underlying lower Fort Union aquifer**. Because of spatially variable lithology**, the middle Fort Union hydrogeologic unit may act as a confining unit** in some areas and as an aquifer in other areas." (Long et al., 2018).
Lower Fort Union aquifer	Clastic sedimentary **aquifer** (consolidated or semi-consolidated rock)	"The **lower Fort Union aquifer** is composed of **as much as 670 ft of thickness of yellow-weathering sandstones and light-gray-weathering sandy mudstones interfingering with alternating brown and gray beds of sandstone, siltstone, claystone, mudstone, and lignite deposited in continental and marine environments**." (Long et al., 2018).
Upper Hell Creek hydrogeologic unit *(in some areas can be aquifer)*	Sedimentary **aquitard** (consolidated or semi-consolidated rock)	"The upper Hell Creek hydrogeologic unit **is composed of as much as 740 ft of thickness of alternating layers of gray and brown mudstone, siltstone, sandstone, and sparse lignite beds deposited by meandering streams with point bars and channel plugs**. Because of spatial variability, this lithology may act as **a confining unit in some areas** and as an aquifer in other areas." (Long et al., 2018).
Lower Hell Creek aquifer	Clastic sedimentary **aquifer** (consolidated or semi-consolidated rock)	"The **general lithology** of the **lower Hell Creek aquifer is similar to the upper Hell Creek hydrogeologic unit**, **except that the latter has a smaller percentage of sandstone**." (Long et al., 2018).
Fox Hills aquifer	Clastic sedimentary **aquifer** (consolidated or semi-consolidated rock)	"The **Fox Hills aquifer** is **the most areally extensive of the units**, with as much as **420 ft of thickness of interbedded sandstone, siltstone, and mudstone**." (Long et al., 2018).
Basal confining unit	Sedimentary **aquitard** (consolidated or	"In the Williston Basin, these three aquifer systems are as deep as 2,850 ft below land surface and **overlie 800–2,000 ft of relatively impermeable Upper Cretaceous marine shale**

Formation name	Category	Quote
	semi-consolidated rock)	that serves as **a basal confining unit that impedes groundwater flow** (Anna, 1986; Downey, 1986; Downey and Dinwiddie, 1988; Thamke and others, 2014).” (Long et al., 2018).

Long, A.J., Thamke, J.N., Davis, K.W., Bartos, T.T. (2018). Groundwater availability of the Williston Basin, United States and Canada: U.S. Geological Survey Professional Paper 1841, 42 p., https://doi.org/10.3133/pp1841

3.35 Williston Basin, Northern Great Plains

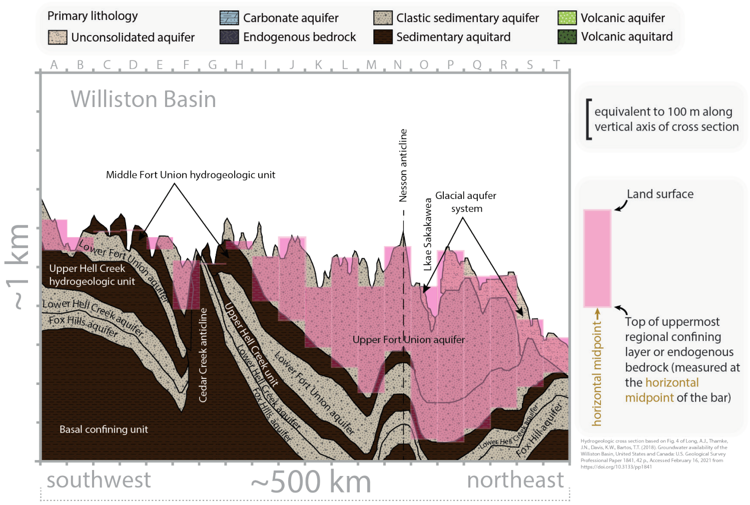

Supplementary Fig. 167. Hydrogeologic cross section. 20 equally spaced transparent pink bars overlie the cross section; each shaded bar depicts the vertical offset from the land surface to the top of the uppermost confining unit or endogenous bedrock.

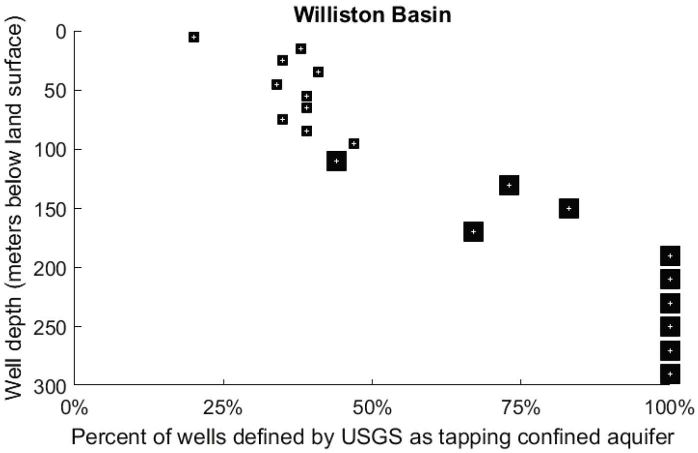

Supplementary Fig. 168. Vertical variations in the prevalence of wells that have been defined as tapping an unconfined or a confined aquifer by the USGS. The smaller squares represent 10 m depth intervals from the land surface to 100 m; the larger squares represent 20 m intervals from 100 m to 300 m below the land surface.

The Williston Basin is located in eastern Montana, western North Dakota, and northwestern South Dakota.

(i) A hydrogeologic cross section presented in Fig. 4 by Long et al. (2018) depicts complex layered sedimentary sequences, with relatively deep depths to the uppermost confining unit in the northeast portion of the hydrogeologic study area.

(ii) We analysed wells within the study area that the USGS has defined as either unconfined or confined. Most (>80%) wells at depths of 140-160 m and at depths exceeding 140 m are defined as tapping a confined aquifer.

Depth to confined conditions: 140-160 m (see (ii) above)

Reference: Long, A.J., Thamke, J.N., Davis, K.W., Bartos, T.T. (2018). Groundwater availability of the Williston Basin, United States and Canada: U.S. Geological Survey Professional Paper 1841, 42 p., Accessed February 16, 2021 from https://doi.org/10.3133/pp1841

The table below presents a series of published quotes (see quotation marks denoting text quoted from another publication, which is cited following the quotation marks with the full reference written in full below the table). The leftmost column lists a title of a hydrogeologic formation depicted in the cross section on the previous page. The rightmost column presents a quote from a hydrogeological study (see base of table for citation). The quote has been annotated with colored text to highlight how we categorized each layer (i.e., see categories in the center column in the table). Specifically: (i) blue text highlights portions of a quote that provide insights into the degree of consolidation of the formation, (ii) red text highlights portions of a quote that categorize the formation as an aquifer or an aquitard (i.e., higher versus lower permeability in the context of local hydrogeologic formations), and (iii) green text highlights portions of a quote that provide information about the lithology of the formation.

Supplementary Table 38. Hydrostratigraphy details for the Williston Basin

Formation name	Category	Quote
Glacial aquifer system	Unconsolidated aquifer	"The **glacial aquifer system** consists of **Quaternary-age unconsolidated till, silt, clay, outwash sand and gravel, and occasional cobbles and boulders**." (Long et al., 2018).
Upper Fort Union aquifer	Clastic sedimentary **aquifer** (consolidated or semi-consolidated rock)	"The **upper Fort Union aquifer** is **as thick as 1,920 ft and composed of crossbedded light-yellow to light-yellow-gray sandstone, sandy mudstone, gray shale, carbonaceous shale, and thick coal beds and associated clinker deposits** (permeable rocks created by the natural burning of coal beds)." (Long et al., 2018).
Middle Fort union hydrogeologic unit *(in some areas can be aquifer)*	Sedimentary **aquitard** (consolidated or semi-consolidated rock)	"**Composed of as much as 520 ft of thickness of alternating beds** of **sandstone, siltstone, mudstone, claystone, and lignite, rocks in the middle Fort Union hydrogeologic unit generally are finer-grained and darker-colored than the overlying upper Fort Union aquifer and underlying lower Fort Union aquifer**. Because of spatially variable lithology**, the middle Fort Union hydrogeologic unit may act as a confining unit** in some areas and as an aquifer in other areas." (Long et al., 2018).
Lower Fort Union aquifer	Clastic sedimentary **aquifer** (consolidated or semi-consolidated rock)	"The **lower Fort Union aquifer** is composed of **as much as 670 ft of thickness of yellow-weathering sandstones and light-gray-weathering sandy mudstones interfingering with alternating brown and gray beds of sandstone, siltstone, claystone, mudstone, and lignite deposited in continental and marine environments**." (Long et al., 2018).
Upper Hell Creek hydrogeologic unit *(in some areas can be aquifer)*	Sedimentary **aquitard** (consolidated or semi-consolidated rock)	"The upper Hell Creek hydrogeologic unit **is composed of as much as 740 ft of thickness of alternating layers of gray and brown mudstone, siltstone, sandstone, and sparse lignite beds deposited by meandering streams with point bars and channel plugs**. Because of spatial variability, this lithology may act as **a confining unit in some areas** and as an aquifer in other areas." (Long et al., 2018).
Lower Hell Creek aquifer	Clastic sedimentary **aquifer** (consolidated or semi-consolidated rock)	"The **general lithology** of the **lower Hell Creek aquifer is similar to the upper Hell Creek hydrogeologic unit, except that the latter has a smaller percentage of sandstone**." (Long et al., 2018).

Formation name	Category	Quote
Fox Hills aquifer	Clastic sedimentary **aquifer** (consolidated or semi-consolidated rock)	"The **Fox Hills aquifer** is **the most areally extensive of the units**, with as much as **420 ft of thickness of interbedded sandstone, siltstone, and mudstone**." (Long et al., 2018).
Basal confining unit	Sedimentary **aquitard** (consolidated or semi-consolidated rock)	"In the Williston Basin, these three aquifer systems are as deep as 2,850 ft below land surface and **overlie 800–2,000 ft of relatively impermeable Upper Cretaceous marine shale** that serves as **a basal confining unit that impedes groundwater flow** (Anna, 1986; Downey, 1986; Downey and Dinwiddie, 1988; Thamke and others, 2014)." (Long et al., 2018).

Long, A.J., Thamke, J.N., Davis, K.W., Bartos, T.T. (2018). Groundwater availability of the Williston Basin, United States and Canada: U.S. Geological Survey Professional Paper 1841, 42 p., https://doi.org/10.3133/pp1841

3.36 Eastern Silurian-Devonian Aquifers, Northern Midwest Aquifer System

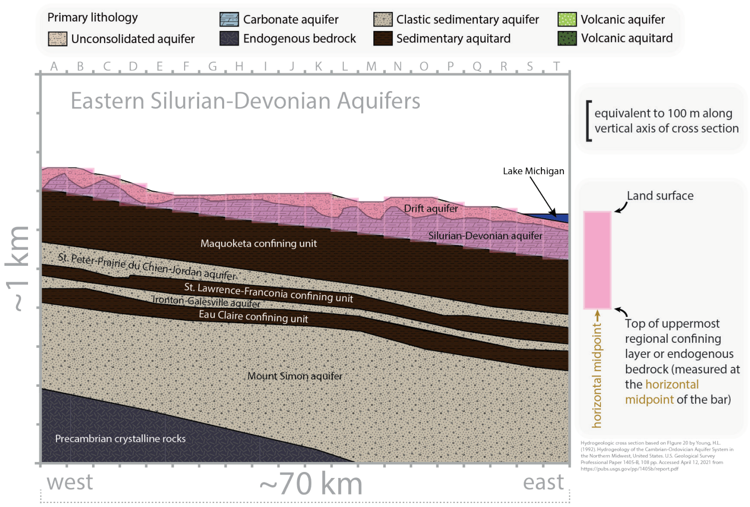

Supplementary Fig. 169. Hydrogeologic cross section. 20 equally spaced transparent pink bars overlie the cross section; each shaded bar depicts the vertical offset from the land surface to the top of the uppermost confining unit or endogenous bedrock.

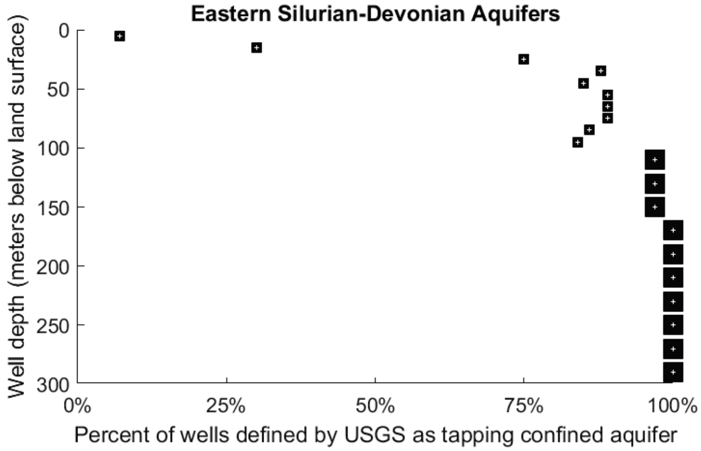

Supplementary Fig. 170. Vertical variations in the prevalence of wells that have been defined as tapping an unconfined or a confined aquifer by the USGS. The smaller squares represent 10 m depth intervals from the land surface to 100 m; the larger squares represent 20 m intervals from 100 m to 300 m below the land surface.

The Eastern Silurian-Devonian Aquifers subarea of the broader Northern Midwest Aquifer System is located in eastern Illinois, west of Lake Michigan.

(i) A hydrogeologic cross section presented in Fig. 20 by Young (1992) shows a drift aquifer atop the Silurian-Devonian carbonates, underlain by the Maquoketa confining unit.

(ii) We analysed wells within the study area that the USGS has defined as either unconfined or confined. Most (>80%) wells at depths of 30-40 m and at depths exceeding 30 m are defined as tapping a confined aquifer.

Depth to confined conditions: 30-40 m (see (ii) above)

Reference: Young, H.L. (1992). Hydrogeology of the Cambrian-Ordovician Aquifer System in the Northern Midwest, United States. U.S. Geological Survey Professional Paper 1405-B, 108 pp. Accessed April 12, 2021 from https://pubs.usgs.gov/pp/1405b/report.pdf

The table below presents a series of published quotes (see quotation marks denoting text quoted from another publication, which is cited following the quotation marks with the full reference written in full below the table). The leftmost column lists a title of a hydrogeologic formation depicted in the cross section on the previous page. The rightmost column presents a quote from a hydrogeological study (see base of table for citation). The quote has been annotated with colored text to highlight how we categorized each layer (i.e., see categories in the center column in the table). Specifically: (i) blue text highlights portions of a quote that provide insights into the degree of consolidation of the formation, (ii) red text highlights portions of a quote that categorize the formation as an aquifer or an aquitard (i.e., higher versus lower permeability in the context of local hydrogeologic formations), and (iii) green text highlights portions of a quote that provide information about the lithology of the formation.

Supplementary Table 39. Hydrostratigraphy details for the Eastern Silurian Devonian

Formation name	Category	Quote
Drift aquifer	Unconsolidated aquifer	"**Aquifer layer**" Young, H.L. (1992) | "Because glacial drift is almost universally present in the study area and **is the shallowest permeable rock material, it provides a ready source of ground water**. **Ground-water availability from the drift** is directly proportional to the amount of **permeable**, **well-sorted sand and gravel within the drift**. The large variety of sediment types in the drift have a wide range of sorting and depositional form" Young (1992). | "**Sand and gravel is less abundant, present as discontinuous lenses within or beneath moraine or as more extensive outwash and ice-contact deposits**." Young (1992)
Silurian-Devonian aquifer	Carbonate aquifer	"**Aquifer layer**" Young, H.L. (1992) | "**Dolomite and limestone** constitute most of the Silurian through Middle Devonian rocks in the study area, which are collectively termed the **Silurian-Devonian aquifer** in this study. The rock is relatively **fine grained and dense, and its permeability is primarily dependent on the extent and. degree of intersection of' fractures and joints within** it and on the subsequent solutional enlargement of these openings by weathering action and ground-water movement." Young (1992)
Maquoketa confining unit	Sedimentary **aquitard** (consolidated or semi-consolidated rock)	"**Confining layer**" Young, H.L. (1992) | "This **confining unit** consists of the **Maquoketa Shale, the Galena Dolomite, and the Decorah, Platteville, and Glenwood Formations or equivalents**." Young (1992)
St. Peter-Prairie du Chien-Jordan aquifer	Clastic sedimentary **aquifer** (consolidated or semi-consolidated rock)	"**Aquifer layer**" Young, H.L. (1992) | "Although this **multiunit** aquifer may be the least uniform of the bedrock aquifer units in **the northern Midwest (fig. 18), it is a major source of ground water in Iowa and Minnesota**. In these States, the Jordan **Sandstone** and all or parts of the overlying Prairie du Chien Group are in direct hydraulic connection, resulting in a highly productive aquifer." Young (1992)
St. Lawrence-Franconia confining unit	Sedimentary **aquitard** (consolidated or semi-consolidated rock)	"**Confining layer**" Young (1992) | "The St. Lawrence and Franconia Formations **form an important regional confining unit** over the Ironton-Galesville aquifer. Although these formations are **dominantly sandstone in the northern part of the area, they are very silty and shaly, fine grained, poorly sorted, and dolomitic**. Thus**, the units are anisotropic and restrict vertical movement of ground water**." Young (1992)

Formation name	Category	Quote
Ironton-Galesville aquifer	Clastic sedimentary **aquifer** (consolidated or semi-consolidated rock)	"**Aquifer layer**" Young, H.L. (1992) | "The Ironton and Galesville **Sandstones** form the most important **aquifer** of the Cambrian-Ordovician aquifer system in the east-central part of the study area, although they generally are not the only rock units open to deep wells." Young, H.L. (1992). | "The aquifer terminates to the west, south, and east (fig. 10) **as the sandstones grade into carbonate rocks, primarily dolomite**." Young (1992)
Eau Claire confining unit	Sedimentary **aquitard** (consolidated or semi-consolidated rock)	"**Confining layer**" Young (1992) | "The Eau Claire Formation and its partial equivalent to the southwest, the Bonneterre Formation, form an extensive **confining unit** above the Mount Simon aquifer. The effectiveness of the Eau Claire as a **confining unit** depends on the relative abundance of **shale, siltstone, dolomite, and sandstone in the formation**." Young (1992)
Mount Simon aquifer	Clastic sedimentary **aquifer** (consolidated or semi-consolidated rock)	"**Aquifer layer**" Young (1992) | "The lowermost **aquifer** of the Cambrian-Ordovician aquifer system is composed primarily of the Mount Simon **Sandstone** and equivalent strata, the Lamotte Sandstone, in Missouri. In Minnesota, the Mount Simon is underlain by Precambrian sedimentary rocks-the Hinckley Sandstone and the older Fond du Lac Formation." Young (1992)
Precambrian crystalline rocks (confining unit)	Endogenous bedrock	Young, 1992 Figure 4, System: "**Precambrian**"; major rock type: "**Igneous and metamorphic crystalline rocks**". "**The dense crystalline rocks of the Precambrian basement** beneath the Cambrian-Ordovician aquifer system are **a very effective confining unit** whose upper surface marks the lower limit of the Cambrian-Ordovician aquifer system." Young 1(992)

Young, H.L. (1992). Hydrogeology of the Cambrian-Ordovician Aquifer System in the Northern Midwest, United States. U.S. Geological Survey Professional Paper 1405-B, 108 pp. Accessed April 12, 2021 from https://pubs.usgs.gov/pp/1405b/report.pdf

3.37 Mississippian-Silurian-Devonian Carbonates, Northern Midwest Aquifer System

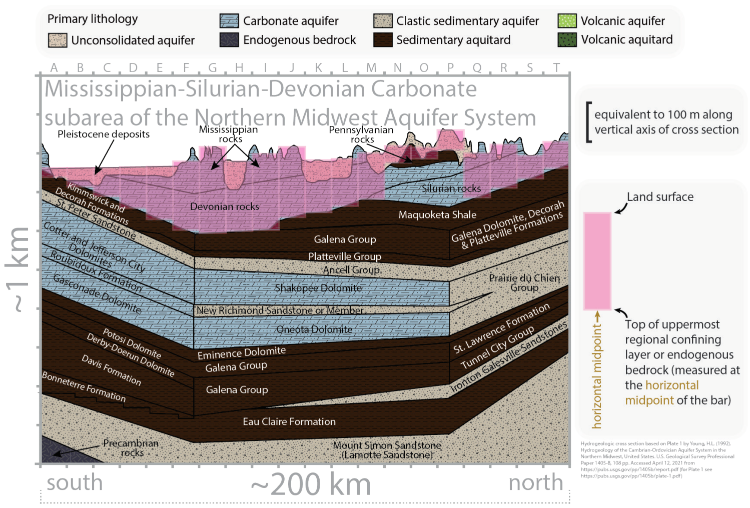

Supplementary Fig. 171. Hydrogeologic cross section. 20 equally spaced transparent pink bars overlie the cross section; each shaded bar depicts the vertical offset from the land surface to the top of the uppermost confining unit or endogenous bedrock.

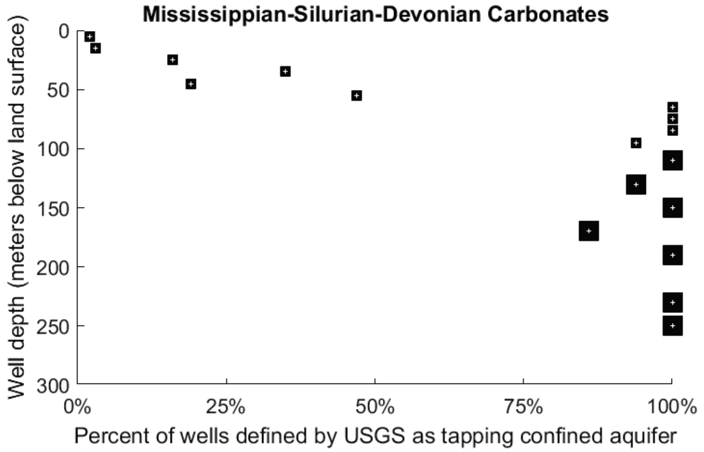

Supplementary Fig. 172. Vertical variations in the prevalence of wells that have been defined as tapping an unconfined or a confined aquifer by the USGS. The smaller squares represent 10 m depth intervals from the land surface to 100 m; the larger squares represent 20 m intervals from 100 m to 300 m below the land surface.

The Mississippian-Silurian-Devonian Carbonates subarea of the broader Northern Midwest Aquifer System is located in eastern Illinois, west of Lake Michigan.

(i) A hydrogeologic cross section presented in Fig. 20 by Young (1992) shows Pleistocene deposits overlying Mississippian- to Silurian-aged carboante rocks atop a series of confining units.

(ii) We analysed wells within the study area that the USGS has defined as either unconfined or confined. Most (>80%) wells at depths of 60-70 m and at depths exceeding 60 m are defined as tapping a confined aquifer.

Depth to confined conditions: 60-70 m (see (ii) above)

Reference: Young, H.L. (1992). Hydrogeology of the Cambrian-Ordovician Aquifer System in the Northern Midwest, United States. U.S. Geological Survey Professional Paper 1405-B, 108 pp. Accessed April 12, 2021 from https://pubs.usgs.gov/pp/1405b/report.pdf

The table below presents a series of published quotes (see quotation marks denoting text quoted from another publication, which is cited following the quotation marks with the full reference written in full below the table). The leftmost column lists a title of a hydrogeologic formation depicted in the cross section on the previous page. The rightmost column presents a quote from a hydrogeological study (see base of table for citation). The quote has been annotated with colored text to highlight how we categorized each layer (i.e., see categories in the center column in the table). Specifically: (i) blue text highlights portions of a quote that provide insights into the degree of consolidation of the formation, (ii) red text highlights portions of a quote that categorize the formation as an aquifer or an aquitard (i.e., higher versus lower permeability in the context of local hydrogeologic formations), and (iii) green text highlights portions of a quote that provide information about the lithology of the formation.

Supplementary Table 40. Hydrostratigraphy details for the Mississippian-Silurian-Devonian

Formation name	Category	Quote
Q – Pleistocene deposits	Unconsolidated aquifer	"**unconsolidated rocks** (**glacial drift and alluvial deposits**) and bedrock aquifers that **store large quantities of ground water**." (Young, 1992). Young, 1992 Figure 4, "**Unconsolidated deposits** of **clay, silt, sand, gravel, and boulders**; degree of sorting variable"
IP – Pennsylvanian rocks	Sedimentary **aquitard** (consolidated or semi-consolidated rock)	Figure 4 of (Young, 1992) under major rock units "**Limestone and shale**" in Iowa and in Northern Illinois "**Limestone, sandstone, siltstone, limestone, coal and clay**". Figure 18 of (Young, 1992), "**Confining layer**" or "**Confining beds**"
M – Mississippian rocks *(It is absent in Wisconsin, and aquifer in Iowa and northern Illinois)*	Carbonate aquifer	Young, 1992 Figure 4, System: "Mississippian"; major rock type in Northern Missouri: "**Limestone, sandstone, siltstone, and shale**". "The Kinderhookian Series is mainly **shale and siltstone** in Illinois and northern Missouri, but the upper part contains **some limestone**. The **limestone** increases in abundance northwestward." (Young, 1992). "The uppermost series in the Mississippian, the Chesterian Series, is present only in the southern parts of Missouri, Illinois, and Indiana. It consists of **repetitive beds of sandstone, shale, and limestone** that are classified into 20 formations in southern Illinois." (Young, 1992). Young, 1992 Figure 18, Hydrogeologic unit: **Pennsylvanian-Mississippian-Devonian confining unit**", Layer number in digital flow model: "**Confining layer**".
D – Devonian rocks *(In Northern Illinois: it is part of the "Upper Confining Unit".*	Carbonate aquifer	Young, 1992 Figure 4, System: "Devonian"; major rock type in Iowa: "**Limestone and shale**", Northern Illinois: "**Dolomite and limestone**". Young, 1992 Figure 18, Iowa: "**Silurian-Devonian aquifer**", and Northern Illinois: "**Devonian or Upper confining bed**".
S – Silurian rocks	Carbonate aquifer	Young, 1992 Figure 4, System: "Silurian" major rocks in Iowa and Northern Illinois: "**Dolomite**" and "**Dolomite and limestone**" respectively. "Rocks of Middle Devonian through Silurian age are mainly **dolomite and limestone** and are termed the **Silurian-Devonian aquifer**." (Young, 1992).
Om – Maquoketa Shale	Sedimentary **aquitard** (consolidated or semi-consolidated rock)	Young, 1992 Figure 18, Rock stratigraphic unit: "**Maquoketa shale**". Wisconsin, Iowa and Northern Illinois: "**Confining bed**". "This **confining unit** consists of the Maquoketa Shale" (Young, 1992).

Formation name	Category	Quote
Okd – Kimmswick and Decorah Formations *(Kimmswick can be aquifer in other states)*	Sedimentary **aquitard** (consolidated or semi-consolidated rock)	"The **Galena Dolomite** of Wisconsin is the rock stratigraphic equivalent of the **Kimmswick Formation** of Missouri." (Young, 1992). "The **Galena Dolomite** is primarily **carbonate, either dolomitic limestone or interbedded limestone and dolomite**." (Young, 1992). Young, 1992 Figure 18, Rock stratigraphic unit: "**Maquoketa shale**". Wisconsin, Iowa and Northern Illinois: "**Confining bed**". "This **confining unit** consists of the Maquoketa Shale" (Young, 1992). Young, 1992 Figure 18 "**confining unit**" in Iowa and "**Galena-Platteville unit**" in Northern Illinois.
Og – Galena Group – Consists of Dubuque, Wise Lake, and Dunleith Formations and Decorah Formation in Iowa or Guttenberg and Spechts Ferry Formations in Illinois.	Sedimentary **aquitard** (consolidated or semi-consolidated rock)	"The **Galena Dolomite** is primarily **carbonate, either dolomitic limestone or interbedded limestone and dolomite**." (Young, 1992). Young, 1992 Figure 18, Rock stratigraphic unit: "**Maquoketa shale**". Wisconsin, Iowa and Northern Illinois: "**Confining bed**". "This **confining unit** consists of the Maquoketa Shale" (Young, 1992). Young, 1992 Figure 18 "**confining unit**" in Iowa and "**Galena-Platteville unit**" in Northern Illinois.
Op – Platteville Group – Consists of Quimbys Mill, Nachusa, Grand Detour, Mifflin, and Pecatonica Formations in Illinois or Platteville Formation in Iowa	Sedimentary **aquitard** (consolidated or semi-consolidated rock)	"The Maquoketa Shale is its main component, but where the Maquoketa overlies **dolomite and shale** of the **Galena Dolomite and the Decorah, Platteville, and Glenwood Formations**, these units also are an important part of the **confining unit**." (Young, 1992). **Platteville Formation**: The lowermost member of the **Platteville**, the Pecatonica Member, **is mainly a yellowish- to grayish-brown, fine- to medium-grained, medium-bedded to massive dolomite that commonly contains some sand** near its base." (Young, 1992). "This **confining unit** consists of the Maquoketa Shale, the **Galena Dolomite**, and the **Decorah, Platteville**, and Glenwood **Formations** or equivalents." (Young, 1992).
Ogp – Galena Dolomite, Decorah and Platteville Formations	Sedimentary **aquitard** (consolidated or semi-consolidated rock)	"The **Galena Dolomite** is primarily **carbonate, either dolomitic limestone or interbedded limestone and dolomite**." (Young, 1992). Young, 1992 Figure 18, Rock stratigraphic unit: "**Maquoketa shale**". Wisconsin, Iowa and Northern Illinois: "**Confining bed**". "This **confining unit** consists of the Maquoketa Shale" (Young, 1992). Young, 1992 Figure 18 "**confining unit**" in Iowa and "**Galena-Platteville unit**" in Northern Illinois.
**Osp** – St. Peter Sandstone	Sedimentary **aquifer** (consolidated or semi-consolidated rock)	Young, 1992 Figure 18, Iowa: "**St. Peter Ss**", rock stratigraphic unit and "**water bearing**". "A prominent exception to the carbonate facies is the **St. Peter Sandstone**–a **very well sorted, pure quartzose sandstone** that is very extensive and uniform throughout the northern Midwest." (Young, 1992).
Oa – Ancell Group – Consists of Glenwood Formation, St. Peter Sandstone	Sedimentary **aquifer** (consolidated or semi-consolidated rock)	Young, 1992 Figure 18, Northern Illinois: "**St. Peter Ss**", rock stratigraphic unit and "**Ancell aquifer**". "A prominent exception to the carbonate facies is the **St. Peter Sandstone**–a **very well sorted, pure quartzose sandstone** that is very extensive and uniform throughout the northern Midwest." (Young, 1992).

Formation name	Category	Quote
in Iowa, Illinois, and Wisconsin		
Ocj – Cotter and Jefferson City Dolomites	Carbonate aquifer	"In northern Missouri, the Jefferson City and **Cotter Dolomites**, equivalent to the Willow River Member, **consist primarily of fine- to medium-grained dolomite with variable amounts of chert and thin beds of shale and fine-grained sandstone**." (Young, 1992). "The upper most Prairie du Chien, the **Willow River Member** of the **Shakopee Formation**, and the basal beds of the St. Peter locally confine the Prairie du Chien and Jordan. Equivalent rocks to the Prairie du Chien in northern Missouri, the Roubidoux Formation and Gasconade Dolomite, are mainly carbonate, but they also contain enough sandstone to make them productive and important aquifers." (Young, 1992). Young, 1992 Figure 18, both in Iowa and Northern Illinois this unit is part of "**Cambrian-Ordovician aquifer**"
Or – Roubidoux Formation	Carbonate aquifer	Young, 1992 Figure 18, Northern Missouri: "Roubidoux Fm., Gasconade Dol., Eminence Dol., Potosi Dol. (**good aquifer**)". "The **Roubidoux Formation** in Missouri, equivalent to the **New Richmond Sandstone** Member, **consists of fine·- to medium-grained, white, partly dolomitic, quartzose sandstone and fine-grained dolomite with beds of chert**. The **Roubidoux ranges in thickness from 100 to 250 ft and is thinnest in** northeastern Missouri." (Young, 1992).
Ogd – Gasconade Dolomite	Carbonate aquifer	Young, 1992 Figure 18, Northern Missouri: "Roubidoux Fm**.,** **Gasconade Dol.**, Eminence Dol., Potosi Dol. (**good aquifer**)". "The **Gasconade Dolomite** in Missouri, equivalent to the Oneota, is **coarse grained and very cherty in the lower part and fine grained and much less cherty** in the upper part." (Young, 1992).
Opc – Prairie du Chien Group – Consists of Shakopee, New Richmond, and Oneota rock units of various rank in Wisconsin, Iowa, and Illinois, plus the Gunter Sandstone in Illinois	Sedimentary **aquifer** (consolidated or semi-consolidated rock)	"The upper most Prairie du Chien, the **Willow River Member** of the **Shakopee Formation**, and the basal beds of the St. Peter locally confine the Prairie du Chien and Jordan. Equivalent rocks to the Prairie du Chien in northern Missouri, the Roubidoux Formation and Gasconade Dolomite, are mainly carbonate, but they also contain enough sandstone to make them productive and important aquifers." (Young, 1992). Young, 1992 Figure 18, both in Iowa and Northern Illinois this unit is part of "**Cambrian-Ordovician aquifer**"
Os – Shakopee Dolomite *(portion of it might be aquitard in Northern Illinois, Young, 1992 of Figure 18).*	Carbonate aquifer	"The major part of the **Shakopee Formation** consists of the Willow River Member, generally **a very fine grained to fine-grained, sandy, light gray to tan or buff dolomite**." (Young, 1992). Young, 1992 Figure 18, in Iowa "Cambrian-Ordovician aquifer system" and in Northern Illinois". "The **New Richmond Sandstone** Member of the **Shakopee Formation** of the Prairie du Chien Group is somewhat similar to the Jordan, but it is thinner and more variable. In some. areas, the New Richmond **probably contributes significantly to the yield of wells**." (Young, 1992).

Formation name	Category	Quote
Onr – New Richmond Sandstone or Member	Sedimentary **aquifer** (consolidated or semi-consolidated rock)	"The New Richmond Sandstone Member generally consists of three lithologies: **a lower fine- to medium grained, white to light gray or tan, quartzose sandstone; a transitional fine-grained, sandy dolomite; and an upper very thin (1 ft or less), blue to green shale or sandy shale** (M.E. Ostrom·, Wisconsin Geological and Natural History Survey, written commun., 1987)." (Young, 1992). "In some. areas, the New Richmond **probably contributes significantly to the yield of wells**." (Young, 1992).
Oo – Oneota Dolomite	Carbonate aquifer	"The **Oneota Dolomite** is **fine to coarse grained, tan to light gray, with variable amounts of chert and sand**. It is typically sandy in the lower part in Wisconsin, but in Illinois the lower part is very cherty with very little sand." (Young, 1992). Young, 1992). Young, 1992 Figure 18, both in Iowa and Northern Illinois this unit is part of "**Cambrian-Ordovician aquifer**".
Ee – Eminence Dolomite	Sedimentary **aquitard** (consolidated or semi-consolidated rock)	Young, 1992 Figure 18, Northern Illinois: "**Eminence Dol.**, Potosi Dol" are part of "**Middle confining units**". "In northern Missouri-the Roubidoux Formation and the Gasconade and **Eminence Dolomites**-are mainly **carbonate rocks that are somewhat permeable and contain some sandstone**." (Young, 1992).
Ep – Potosi Dolomite	Sedimentary **aquitard** (consolidated or semi-consolidated rock)	Young, 1992 Figure 18, Northern Illinois: "Eminence Dol., **Potosi Dol**" are part of "**Middle confining units**". "**The most productive parts of the aquifer system** in this area are the Roubidoux Formation and Gasconade, Eminence, and **Potosi Dolomites** (fig. 18)" (Young, 1992).
Esl – St. Lawrence Formation	Sedimentary **aquitard** (consolidated or semi-consolidated rock)	"**Middle Confining unit**" (Young, 1992) in Northern Illinois. "**Confining bed**" (Young, 1992) in Iowa. "Potosi **Dolomite** (equivalent to **St. Lawrence Formation** elsewhere)" (Young, 1992). "Both are **fine- to medium-crystalline dolomites** that contain algal material." (Young, 1992).
Edd – Derby-Doerun Dolomite	Sedimentary **aquitard** (consolidated or semi-consolidated rock)	Young, 1992 Figure 18, Northern Missouri: "**Derby-Doerun Dol.** (**poor aquifer**)". "In Missouri, Imes (1985) includes the Potosi and **Derby-Doerun in his Cambrian Ordovician aquifer** (fig. 18), although the **Derby-Doerun is not considered to be very productive**." (Young, 1992). "In the southern and eastern parts of the area, the Potosi and **Derby-Doerun Dolomites** and the **upper part of the Davis Formation are the equivalent rocks (mainly carbonate** rocks)." (Young, 1992).
Ef – Franconia Formation	Sedimentary **aquitard** (consolidated or semi-consolidated rock)	"**Middle Confining unit**" (Young, 1992). "St. Lawrence-**Franconia confining unit**-The two formations that compose this unit generally are **very silty and shaly, fine grained, poorly sorted, dolomitic sandstones**." (Young, 1992).
Etc – Tunnel City Group – Consists of Lone Rock and Mazomanie Formations in Wisconsin and Iowa	Sedimentary **aquitard** (consolidated or semi-consolidated rock	Young 1992, Figure 6: Geologic unit: "Tunnel City group", the rock type is "**Shaly sandstone** and/or **shale**". "**The Franconia, whose Mazomanie Member** is coarse grained, has been included with the Ironton-Galesville aquifer in Minnesota by some investigators (Kanivetsky, 1978; Adolphson and others, 1981). However, natural-gamma well logs show the Franconia there to be primarily a **confining unit** (Woodward, 1986)." (Young, 1992).

Formation name	Category	Quote
Ed – Davis Formation	Sedimentary **aquitard** (consolidated or semi-consolidated rock)	Young, 1992 Figure 18, Northern Missouri: "**The Davis Formation**, **Lower confining bed**". "**The Davis Formation** in Missouri consists of **silty to sandy shale and limestone or dolomite, with beds of flat-pebble conglomerate**." (Young, 1992). "The Davis also is a dolomite but contains as much as **50 percent shale and silt**." (Young, 1992)
Eb – Bonneterre Formation	Sedimentary **aquitard** (consolidated or semi-consolidated rock)	Young, 1992 Figure 18, Northern Missouri: "Bonneterre Fm. (little information)". "the **Bonneterre Formation**, form an extensive **confining unit** above the Mount Simon aquifer." (Young, 1992). "The Bonneterre generally is **a fine- to coarse-grained**, **shaly, dolomitized calcarenite with much oolitic and stromatolitic material**". "" (Young, 1992).
El – Lamotte Sandstone	Sedimentary **aquifer** (consolidated or semi-consolidated rock)	Young, 1992 Figure 18, Northern Missouri: "**Lamotte Ss.** **(probable aquifer)**". "The **lowermost aquifer of the Cambrian-Ordovician aquifer system** is composed primarily of the Mount Simon Sandstone and equivalent strata, the **Lamotte Sandstone**, in Missouri." (Young, 1992). "It consists mainly of the **Mount Simon Sandstone** in the north and its equivalent, the **Lamotte Sandstone**, in northern Missouri" (Young, 1992).
Eig – Ironton Galesville Sandstones	Sedimentary **aquifer** (consolidated or semi-consolidated rock)	"The Ironton and Galesville **Sandstones** form **the most important aquifer of the Cambrian-Ordovician aquifer system** in the east-central part of the study area" (Young, 1992). "It consists of the **moderately sorted, quartzose Ironton and Galesville Sandstones** and generally is 50 to 150 ft thick." (Young, 1992).
Eec – Eau Claire Formation	Sedimentary **aquitard** (consolidated or semi-consolidated rock)	Young, 1992 Figure 18, Northern Missouri: "**Eau Claire confining unit**". "**The Eau Claire Formation** and its partial equivalent to the southwest, the Bonneterre Formation, **form an extensive confining unit** above the Mount Simon aquifer." (Young, 1992). "The effectiveness of the Eau Claire as a **confining unit** depends ·on the relative abundance of **shale, siltstone, dolomite, and sandstone in the formation**." (Young, 1992).
Ems – Mount Simon Sandstone	Sedimentary **aquifer** (consolidated or semi-consolidated rock)	Young, 1992 Figure 18, Hydrogeologic unit: "**Mount Simon aquifer**". "The **Mount Simon Sandstone** generally is a **medium to coarse-grained, poorly to moderately sorted, sometimes pebbly, white to gray, quartzose sandstone**." (Young, 1992).
pE – Precambrian rocks, undivided	Endogenous bedrock	Young, 1992 Figure 4, System: "**Precambrian**"; major rock type in Northern Missouri: "**Igneous and metamorphic crystalline rocks**". "**The dense crystalline rocks of the Precambrian basement** beneath the Cambrian-Ordovician aquifer system are **a very effective confining unit** whose upper surface marks the lower limit of the Cambrian-Ordovician aquifer system." (Young, 1992).

Young, H.L. (1992). Hydrogeology of the Cambrian-Ordovician Aquifer System in the Northern Midwest, United States. U.S. Geological Survey Professional Paper 1405-B, 108 pp. Accessed June 14, 2022 via https://pubs.er.usgs.gov/publication/pp1405B

3.38 Northeast Missouri Carbonates, Northern Midwest Aquifer System

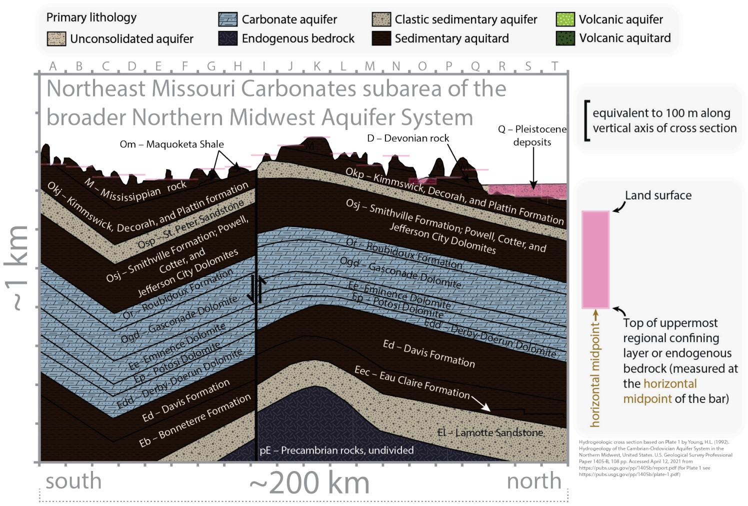

Supplementary Fig. 173. Hydrogeologic cross section. 20 equally spaced transparent pink bars overlie the cross section; each shaded bar depicts the vertical offset from the land surface to the top of the uppermost confining unit or endogenous bedrock.

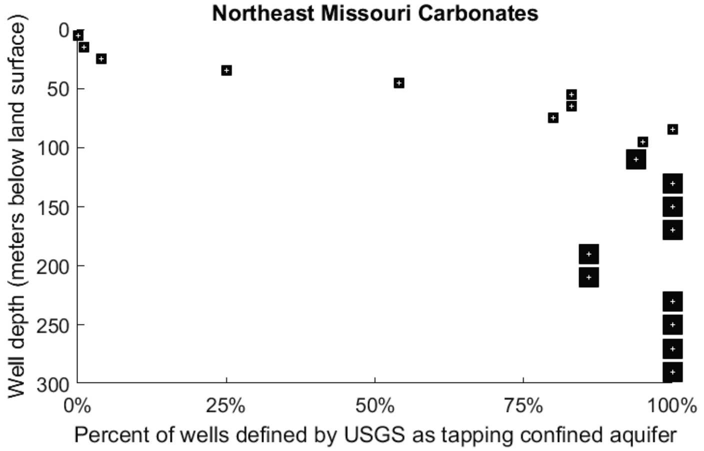

Supplementary Fig. 174. Vertical variations in the prevalence of wells that have been defined as tapping an unconfined or a confined aquifer by the USGS. The smaller squares represent 10 m depth intervals from the land surface to 100 m; the larger squares represent 20 m intervals from 100 m to 300 m below the land surface.

The Northeast Missouri Carbonates subarea is located in northeastern Missouri.

(i) A hydrogeologic cross section presented in Plate 1 by Young (1992) depicts layered clastic and carbonate sedimentary units with relatively shallow confining units in most of the area.

(ii) We analysed wells within the study area that the USGS has defined as either unconfined or confined. Most (>80%) wells at depths of 50-60 m and at depths exceeding 50 m are defined as tapping a confined aquifer.

Depth to confined conditions: 50-60 m (see (ii) above)

Reference: Young, H.L. (1992). Hydrogeology of the Cambrian-Ordovician Aquifer System in the Northern Midwest, United States. U.S. Geological Survey Professional Paper 1405-B, 108 pp. Accessed April 12, 2021 from https://pubs.usgs.gov/pp/1405b/report.pdf (for Plate 1 see https://pubs.usgs.gov/pp/1405b/plate-1.pdf)

The table below presents a series of published quotes (see quotation marks denoting text quoted from another publication, which is cited following the quotation marks with the full reference written in full below the table). The leftmost column lists a title of a hydrogeologic formation depicted in the cross section on the previous page. The rightmost column presents a quote from a hydrogeological study (see base of table for citation). The quote has been annotated with colored text to highlight how we categorized each layer (i.e., see categories in the center column in the table). Specifically: (i) blue text highlights portions of a quote that provide insights into the degree of consolidation of the formation, (ii) red text highlights portions of a quote that categorize the formation as an aquifer or an aquitard (i.e., higher versus lower permeability in the context of local hydrogeologic formations), and (iii) green text highlights portions of a quote that provide information about the lithology of the formation.

Supplementary Table 41. Hydrostratigraphy details for the Northeast Missouri Carbonate

Formation name	Category	Quote
**Q** – Pleistocene deposits	Unconsolidated aquifer	"**unconsolidated rocks** (**glacial drift and alluvial deposits**) and bedrock aquifers that **store large quantities of ground water**." (Young, 1992). Young, 1992 Figure 4, "**Unconsolidated deposits** of **clay, silt, sand, gravel, and boulders**; degree of sorting variable"
**M** – Mississippian rocks *(Keokuk-Burlington is an aquifer and St. Louis-Salem are poor aquifer)*	Sedimentary **aquitard** (consolidated or semi-consolidated rock)	Young, 1992 Figure 4, System: "Mississippian"; major rock type in Northern Missouri: "**Limestone, sandstone, siltstone, and shale**". "The Kinderhookian Series is mainly **shale and siltstone** in Illinois and northern Missouri, but the upper part contains **some limestone**. The **limestone** increases in abundance northwestward." (Young, 1992). "The uppermost series in the Mississippian, the Chesterian Series, is present only in the southern parts of Missouri, Illinois, and Indiana. It consists of **repetitive beds of sandstone, shale, and limestone** that are classified into 20 formations in southern Illinois." (Young, 1992). Young, 1992 Figure 18, Hydrogeologic unit: **Pennsylvanian-Mississippian-Devonian confining unit**", Layer number in digital flow model: "**Confining layer**".
**D** – Devonian rocks	Sedimentary rock **aquitard** (consolidated or semi-consolidated rock)	Young, 1992 Figure 4, System: "Devonian"; major rock type in Northern Missouri: "**Limestone and shale**". Young, 1992 Figure 18, Northern Missouri: "**Confining bed**". Young, 1992 Figure 18, Northern Missouri: "**Confining bed**".
**Om** – Maquoketa Shale	Sedimentary **aquitard** (consolidated or semi-consolidated rock)	Young, 1992 Figure 18, Rock stratigraphic unit: "**Maquoketa shale**". Northern Missouri: "**Confining bed**". ""This **confining unit** consists of the Maquoketa Shale" (Young, 1992).
**Okj (Okp)** – Kimmswick, Decorah, and Plattin Formation and (Joachim) Dolomite *(Kimmswick formation is a minor aquifer)*	Sedimentary **aquitard** (consolidated or semi-consolidated rock)	Young, 1992 Figure 18, Northern Missouri: "**Decorah Fm., Plattin Fm., Joachim Dol.,** (**confining bed**)". "The Galena Dolomite of Wisconsin is the rock stratigraphic equivalent of the Kimmswick Formation of Missouri." (Young, 1992). "In northern Missouri, the equivalent Decorah and Plattin Formations and **the Joachim Dolomite** (**mainly carbonate rocks**) contain dolomite with various amounts of **interbedded shale** that **restricts vertical ground-water flow** (Imes, 1985)." (Young, 1992).
**Osp** – St. Peter Sandstone	Sedimentary **aquifer** (consolidated or	Young, 1992 Figure 18, Northern Missouri: "**St. Peter Ss**, Everton Dol. (**moderate aquifer**)". "A prominent exception to the carbonate facies is the **St. Peter Sandstone**–a **very well**

Formation name	Category	Quote
	semi-consolidated rock)	**sorted, pure quartzose sandstone** that is very extensive and uniform throughout the northern Midwest." (Young, 1992).
**Osj** – Smithville Formation; Powell, Cotter, and Jefferson City Dolomites	Sedimentary **aquitard** (consolidated or semi-consolidated rock)	"The younger **Powell Dolomite and Smithville Formation** in Missouri may have no equivalent to the north, presumably because of pre-St. Peter Sandstone erosion." (Young, 1992). "In northern Missouri, **the Jefferson City and Cotter Dolomites**, equivalent to the Willow River Member, consist primarily of **fine- to medium-grained dolomite** with variable amounts of **chert and thin beds of shale and fine-grained sandstone**." (Young, 1992). Young, 1992 Figure 18, Northern Missouri: "**Confining bed**".
**Or** – Roubidoux Formation	Carbonate aquifer	Young, 1992 Figure 18, Northern Missouri: "Roubidoux Fm., Gasconade Dol., Eminence Dol., Potosi Dol. (**good aquifer**)". "The **Roubidoux Formation** in Missouri, equivalent to the **New Richmond Sandstone** Member, **consists of fine·- to medium-grained, white, partly dolomitic, quartzose sandstone and fine-grained dolomite with beds of chert**. The **Roubidoux ranges in thickness from 100 to 250 ft and is thinnest in** northeastern Missouri." (Young, 1992).
**Ogd** – Gasconade Dolomite	Carbonate aquifer	Young, 1992 Figure 18, Northern Missouri: "Roubidoux Fm**.**, **Gasconade Dol.**, Eminence Dol., Potosi Dol. (**good aquifer**)". "The **Gasconade Dolomite** in Missouri, equivalent to the Oneota, is **coarse grained and very cherty in the lower part and fine grained and much less cherty** in the upper part." (Young, 1992).
**Ee** -Eminence Dolomite	Carbonate aquifer	Young, 1992 Figure 18, Northern Missouri: "Roubidoux Fm., Gasconade Dol., **Eminence Dol.**, Potosi Dol. (**good aquifer**)". "In northern Missouri-the Roubidoux Formation and the Gasconade and **Eminence Dolomites**-are mainly **carbonate rocks that are somewhat permeable and contain some sandstone**." (Young, 1992).
**Ep** – Potosi Dolomite	Carbonate aquifer	Young, 1992 Figure 18, Northern Missouri: "Roubidoux Fm., Gasconade Dol., Eminence Dol., **Potosi Dol.** (**good aquifer**)". "**The most productive parts of the aquifer system** in this area are the Roubidoux Formation and Gasconade, Eminence, and **Potosi Dolomites** (fig. 18)" (Young, 1992).
Edd – Derby-Doerun Dolomite *(poor aquifer)*	Carbonate aquifer	Young, 1992 Figure 18, Northern Missouri: "**Derby-Doerun Dol.** (**poor aquifer**)". "In Missouri, Imes (1985) includes the Potosi and **Derby-Doerun in his Cambrian Ordovician aquifer** (fig. 18), although the **Derby-Doerun is not considered to be very productive**." (Young, 1992). "In the southern and eastern parts of the area, the Potosi and **Derby-Doerun Dolomites** and the **upper part of the Davis Formation are the equivalent rocks (mainly carbonate** rocks)." (Young, 1992).
Ed – Davis Formation	Sedimentary **aquitard** (consolidated or semi-consolidated rock)	Young, 1992 Figure 18, Northern Missouri: "**The Davis Formation**, **Lower confining bed**". "**The Davis Formation** in Missouri consists of **silty to sandy shale and limestone or dolomite, with beds of flat-pebble conglomerate**." (Young, 1992). "The Davis also is a dolomite but contains as much as **50 percent shale and silt**." (Young, 1992)

Formation name	Category	Quote
Eb – Bonneterre Formation	Sedimentary **aquitard** (consolidated or semi-consolidated rock)	Young, 1992 Figure 18, Northern Missouri: “Bonneterre Fm. (little information)”. “the **Bonneterre Formation**, form an extensive **confining unit** above the Mount Simon aquifer.” (Young, 1992). “The Bonneterre generally is **a fine- to coarse-grained**, **shaly, dolomitized calcarenite with much oolitic and stromatolitic material**”. “” (Young, 1992).
Eec – Eau Claire Formation	Sedimentary **aquitard** (consolidated or semi-consolidated rock)	Young, 1992 Figure 18, Northern Missouri: “**Eau Claire confining unit**”. “**The Eau Claire Formation** and its partial equivalent to the southwest, the Bonneterre Formation, **form an extensive confining unit** above the Mount Simon aquifer.” (Young, 1992). “The effectiveness of the Eau Claire as a **confining unit** depends ·on the relative abundance of **shale, siltstone, dolomite, and sandstone in the formation**.” (Young, 1992).
El – Lamotte Sandstone	Sedimentary **aquifer** (consolidated or semi-consolidated rock)	Young, 1992 Figure 18, Northern Missouri: “**Lamotte Ss. (probable aquifer)**”. “The **lowermost aquifer of the Cambrian-Ordovician aquifer system** is composed primarily of the Mount Simon Sandstone and equivalent strata, the **Lamotte Sandstone**, in Missouri.” (Young, 1992). “It consists mainly of the **Mount Simon Sandstone** in the north and its equivalent, the **Lamotte Sandstone**, in northern Missouri” (Young, 1992).
pE Precambrian rocks, undivided	Endogenous bedrock	Young, 1992 Figure 4, System: “**Precambrian**”; major rock type in Northern Missouri: “**Igneous and metamorphic crystalline rocks**”. “**The dense crystalline rocks of the Precambrian basement** beneath the Cambrian-Ordovician aquifer system are **a very effective confining unit** whose upper surface marks the lower limit of the Cambrian-Ordovician aquifer system.” (Young, 1992).

Young, H.L. (1992). Hydrogeology of the Cambrian-Ordovician Aquifer System in the Northern Midwest, United States. U.S. Geological Survey Professional Paper 1405-B, 108 pp. Accessed June 14, 2022 via https://pubs.er.usgs.gov/publication/pp1405B

3.39 Northern Cambrian-Ordovician Aquifers, Northern Midwest Aquifer System

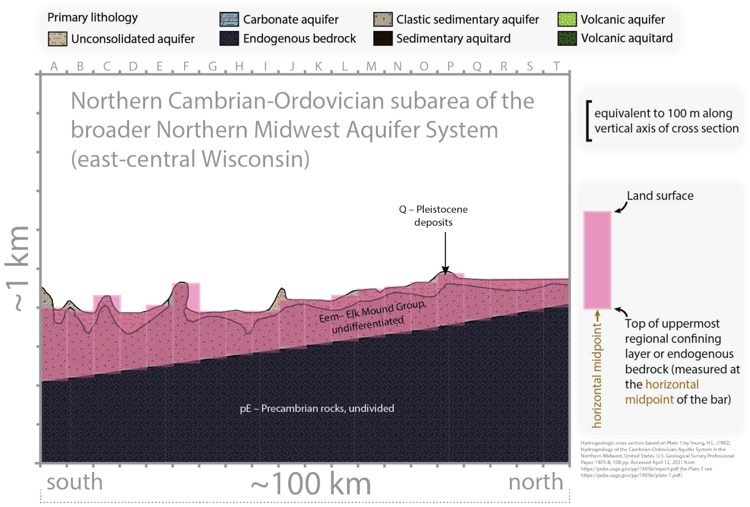

Supplementary Fig. 175. Hydrogeologic cross section. 20 equally spaced transparent pink bars overlie the cross section; each shaded bar depicts the vertical offset from the land surface to the top of the uppermost confining unit or endogenous bedrock.

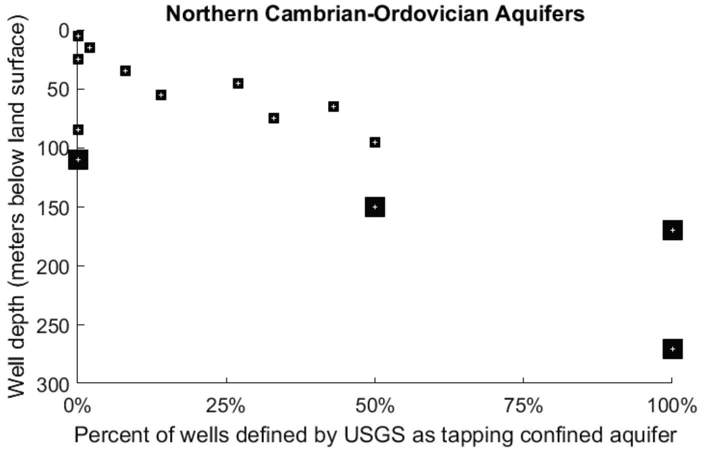

Supplementary Fig. 176. Vertical variations in the prevalence of wells that have been defined as tapping an unconfined or a confined aquifer by the USGS. The smaller squares represent 10 m depth intervals from the land surface to 100 m; the larger squares represent 20 m intervals from 100 m to 300 m below the land surface.

The Northern Cambrian-Ordovician Aquifers subarea is located in east-central Wisconsin.

(i) A hydrogeologic cross section in Plate 1 by Young (1992) depicts sedimentary deposits overlying Precambrian bedrock.

(ii) We analysed wells within the study area that the USGS has defined as either unconfined or confined. Most (>80%) wells at depths of 160-180 m and at depths exceeding 160 m are defined as tapping a confined aquifer.

Depth to confined conditions: 160-180 m (see (ii) above)

Reference: Young, H.L. (1992). Hydrogeology of the Cambrian-Ordovician Aquifer System in the Northern Midwest, United States. US Geological Survey Professional Paper 1405-B, 108 pp. Accessed April 12, 2021 from https://pubs.usgs.gov/pp/1405b/report.pdf (for Plate 1 see https://pubs.usgs.gov/pp/1405b/plate-1.pdf)

The table below presents a series of published quotes (see quotation marks denoting text quoted from another publication, which is cited following the quotation marks with the full reference written in full below the table). The leftmost column lists a title of a hydrogeologic formation depicted in the cross section on the previous page. The rightmost column presents a quote from a hydrogeological study (see base of table for citation). The quote has been annotated with colored text to highlight how we categorized each layer (i.e., see categories in the center column in the table). Specifically: (i) blue text highlights portions of a quote that provide insights into the degree of consolidation of the formation, (ii) red text highlights portions of a quote that categorize the formation as an aquifer or an aquitard (i.e., higher versus lower permeability in the context of local hydrogeologic formations), and (iii) green text highlights portions of a quote that provide information about the lithology of the formation.

Supplementary Table 42. Hydrostratigraphy details for the Northern Cambrian Ordovician

Formation name	Category	Quote
Q – Pleistocene deposits	Unconsolidated aquifer	"**unconsolidated rocks** (**glacial drift and alluvial deposits**) and bedrock aquifers that **store large quantities of ground water**." (Young, 1992). Young, 1992 Figure 4, "**Unconsolidated deposits** of **clay, silt, sand, gravel, and boulders**; degree of sorting variable"
Eem– Elk Mound Group, undifferentiated	Sedimentary rock **aquitard** (consolidated or semi-consolidated rock)	"Thus he proposed the term **Elk Mound Group**, which consists of the **Mount Simon Sandstone, the Eau Claire Formation, and the Wonewoc Sandstone**." (Young, 1992). "**The aquifer is generally between 50 and 150 ft thick but exceeds 200 ft on the northeastern edge of the Illinois basin**. The **thickness of the aquifer is uncertain north of Milwaukee, where the Ironton and Galesville Sandstones and the other sandstones of the Elk Mound Group** generally cannot be differentiated in the subsurface from drill cuttings or borehole geophysical logs." (Young, 1992).
pE – Precambrian rocks, undivided	Endogenous bedrock	Young, 1992 Figure 4, System: "**Precambrian**"; major rock type in Northern Missouri: "**Igneous and metamorphic crystalline rocks**". "**The dense crystalline rocks of the Precambrian basement** beneath the Cambrian-Ordovician aquifer system are **a very effective confining unit** whose upper surface marks the lower limit of the Cambrian-Ordovician aquifer system." (Young, 1992).

Young, H.L. (1992). Hydrogeology of the Cambrian-Ordovician Aquifer System in the Northern Midwest, United States. U.S. Geological Survey Professional Paper 1405-B, 108 pp. Accessed June 14, 2022 via https://pubs.er.usgs.gov/publication/pp1405B

3.40 Upper Carbonate Aquifer, Northern Midwest Aquifer System

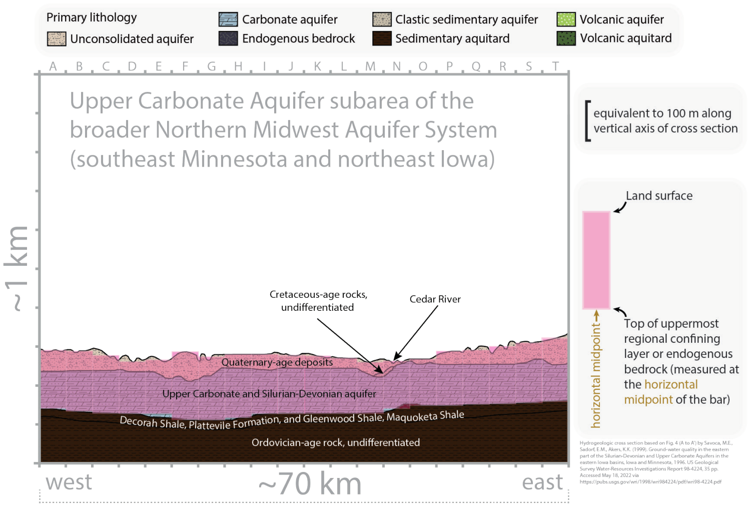

Supplementary Fig. 177. Hydrogeologic cross section. 20 equally spaced transparent pink bars overlie the cross section; each shaded bar depicts the vertical offset from the land surface to the top of the uppermost confining unit or endogenous bedrock.

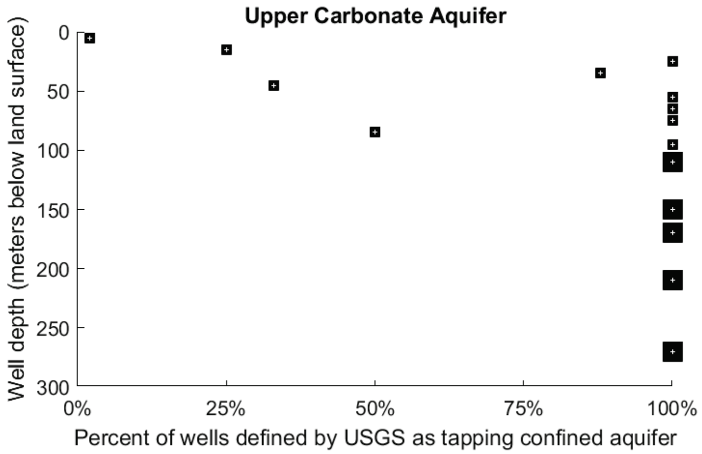

Supplementary Fig. 178. Vertical variations in the prevalence of wells that have been defined as tapping an unconfined or a confined aquifer by the USGS. The smaller squares represent 10 m depth intervals from the land surface to 100 m; the larger squares represent 20 m intervals from 100 m to 300 m below the land surface.

The Upper Carbonate Aquifer subarea is located in southeastern Minnesota and northeastern Iowa.

(i) A hydrogeologic cross section presented in Fig. 4 by Savoca et al. (1999) depicts Quaternary deposits (~50 m thick) overlying a carbonate rock aquifer (~100-150 m thick).

(ii) We analysed wells within the study area that the USGS has defined as either unconfined or confined. Most (>80%) wells at depths of 20-30 m and at depths exceeding 20 m are defined as tapping a confined aquifer.

(iii) Savoca et al. (1999) write (quote) *"The Upper carbonate aquifer underlies the northern part of the study area and consists of 250 to 600 ft of Ordovician- and Devonian-age limestone, dolomite, dolomitic limestone, and shale (table 1; fig. 4, hydrogeologic section A-A') of shallow marine origin. The aquifer is overlain by unconsolidated Quaternary- and Cretaceous-age deposits (sand, gravel, and clay) and is unconfined except in areas where overlying fine-grained deposits produce locally confined conditions"*

Depth to confined conditions: 20-30 m (see (ii) above)

Reference: Savoca, M.E., Sadorf, E.M., Akers, K.K. (1999). Ground-water quality in the eastern part of the Silurian-Devonian and Upper Carbonate Aquifers in the eastern Iowa basins, Iowa and Minnesota, 1996. US Geological Survey Water-Resources Investigations Report 98-4224, 35 pp. Accessed May 18, 2022 via https://pubs.usgs.gov/wri/1998/wri984224/pdf/wri98-4224.pdf

The table below presents a series of published quotes (see quotation marks denoting text quoted from another publication, which is cited following the quotation marks with the full reference written in full below the table). The leftmost column lists a title of a hydrogeologic formation depicted in the cross section on the previous page. The rightmost column presents a quote from a hydrogeological study (see base of table for citation). The quote has been annotated with colored text to highlight how we categorized each layer (i.e., see categories in the center column in the table). Specifically: (i) blue text highlights portions of a quote that provide insights into the degree of consolidation of the formation, (ii) red text highlights portions of a quote that categorize the formation as an aquifer or an aquitard (i.e., higher versus lower permeability in the context of local hydrogeologic formations), and (iii) green text highlights portions of a quote that provide information about the lithology of the formation.

Supplementary Table 43. Hydrostratigraphy details for the Upper Carbonate

Formation name	Category	Quote
Quaternary-age deposits	Unconsolidated aquifer	"**Unconsolidated Quaternary-age deposits**, which commonly contain **low permeability glacial deposits**, cover most of the study area (fig. 6)." (Savoca et al., 1999). "**Sand, gravel, silt, and clay**" (Table 1 Savoca et al., 1999). "**Surficial aquifer**" (Table 1, Savoca et al., 1999).
Cretaceous-age rocks, undifferentiated	Clastic sedimentary **aquifer** (consolidated or semi-consolidated rock)	"**Cretaceous-age deposits** (**sand, gravel, and clay**)" (Savoca et al., 1999). "**Dakota Formation**" (Table 1 Savoca et al., 1999). "Isolated, **water-bearing units**" (Table 1, Savoca et al., 1999).
Upper Carbonate and Silurian-Devonian aquifer	Carbonate aquifer	"**Limestone and dolomite** are the dominant lithologies in the **Silurian-Devonian and Upper Carbonate aquifers**." (Savoca et al., 1999). "The **Upper Carbonate aquifer** underlies thenorthern part of the study area and consists of 250 to 600 ft of **Ordovician- and Devonian-age limestone, dolomite, dolomitic limestone, and shale** (table 1; fig. 4, hydrogeologic section A-A') of shallow marine origin." Savoca et al., 1999).
Decorah Shale, Plattevile Formation, and Gleenwood Shale, Maquoketa Shale	Sedimentary **aquitard** (consolidated or semi-consolidated rock)	"The Upper Carbonate aquifer is **underlain by a confining unit** consisting of the **Ordovician-age Decorah Shale, Platteville Formation, and Glenwood Shale**." (Savoca et al., 1999). "**Shale, dolomitic limestone, and limestone**" & "**Confining unit**" (Table 1, Savoca et al., 1999).
Ordovician-age rock, undifferentiated	Sedimentary **aquitard** (consolidated or semi-consolidated rock)	"The Silurian-Devonian aquifer is underlain by a **confining unit** consisting of the **Ordovician-age Maquoketa Shale**." (Savoca et al., 1999). "**Shale, dolomitic limestone, and limestone**" & "**Confining unit**" (Table 1, Savoca et al., 1999).

Savoca, M.E., Sadorf, E.M., Akers, K.K. (1999). Ground-Water quality in the eastern part of the Silurian-Devonian and Upper Carbonate Aquifers in the eastern Iowa basins, Iowa and Minnesota, 1996. US Geological Survey Water-Resources Investigations Report 98-4224, 35 pp. Accessed June 5, 2022 via https://pubs.er.usgs.gov/publication/wri984224

3.41 Western Cambrian-Ordovician Aquifers, Northern Midwest Aquifer System

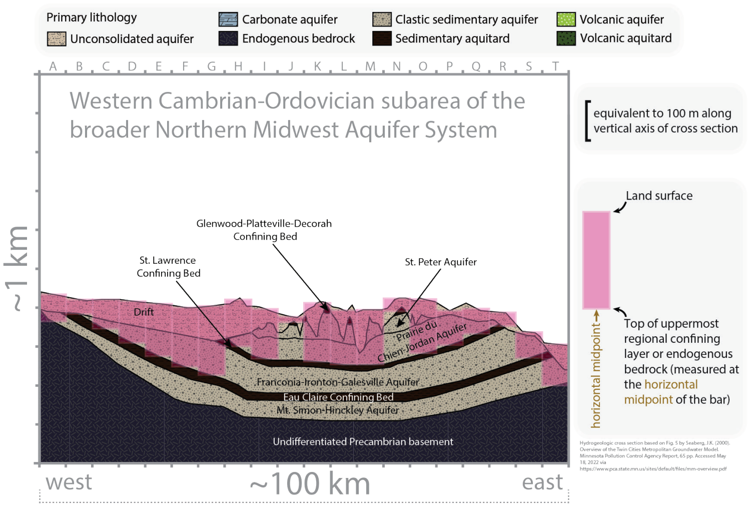

Supplementary Fig. 179. Hydrogeologic cross section. 20 equally spaced transparent pink bars overlie the cross section; each shaded bar depicts the vertical offset from the land surface to the top of the uppermost confining unit or endogenous bedrock.

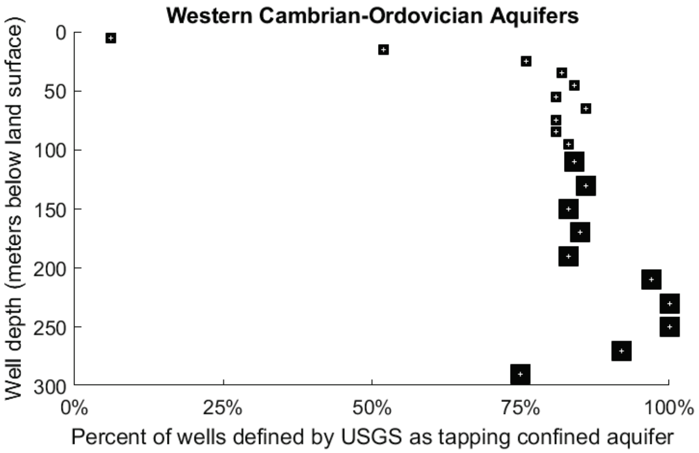

Supplementary Fig. 180. Vertical variations in the prevalence of wells that have been defined as tapping an unconfined or a confined aquifer by the USGS. The smaller squares represent 10 m depth intervals from the land surface to 100 m; the larger squares represent 20 m intervals from 100 m to 300 m below the land surface.

The Western Cambrian-Ordovician Aquifers area is situated in southeastern Minnesota and western Wisconsin.

(i) A hydrogeologic cross section presented in Fig. 5 by Seaberg (2000) depicts layered sedimentary sequences including discontinuous aquitards (Glenwood-Platteville-Decorah units) and more continuous aquitards (e.g., St. Lawrence Confining Bed).

(ii) We analysed wells within the study area that the USGS has defined as either unconfined or confined. Most (>80%) wells at depths of 30-40 m and at depths exceeding 30 m are defined as tapping a confined aquifer.

Depth to confined conditions: 30-40 m (see (ii) above)

Reference: Seaberg, J.K. (2000). Overview of the Twin Cities Metropolitan Groundwater Model. Minnesota Pollution Control Agency Report, 65 pp. Accessed May 18, 2022 via https://www.pca.state.mn.us/sites/default/files/mm-overview.pdf

The table below presents a series of published quotes (see quotation marks denoting text quoted from another publication, which is cited following the quotation marks with the full reference written in full below the table). The leftmost column lists a title of a hydrogeologic formation depicted in the cross section on the previous page. The rightmost column presents a quote from a hydrogeological study (see base of table for citation). The quote has been annotated with colored text to highlight how we categorized each layer (i.e., see categories in the center column in the table). Specifically: (i) blue text highlights portions of a quote that provide insights into the degree of consolidation of the formation, (ii) red text highlights portions of a quote that categorize the formation as an aquifer or an aquitard (i.e., higher versus lower permeability in the context of local hydrogeologic formations), and (iii) green text highlights portions of a quote that provide information about the lithology of the formation.

Supplementary Table 44. Hydrostratigraphy details for the Western Cambrian-Ordovician

Formation name	Category	Quote
Drift	Unconsolidated aquifer	**Aquifer layer**" Young, H.L. (1992) | "Because glacial drift is almost universally present in the study area and **is the shallowest permeable rock material, it provides a ready source of ground water. Ground-water availability from the drift** is directly proportional to the amount of permeable, **well-sorted sand and gravel within the drift**. The large variety of sediment types in the drift have a wide range of sorting and depositional form" Young, H.L. (1992). | "**Sand and gravel is less abundant, present as discontinuous lenses within or beneath moraine or as more extensive outwash and ice-contact deposits**." Young, H.L. (1992)
Glenwood-Platteville-Decorah confining bed	Sedimentary **aquitard** (consolidated or semi-consolidated rock)	"**Confining layer**" Young, H.L. (1992) | "This **confining unit** consists of the **Maquoketa Shale, the Galena Dolomite, and the Decorah, Platteville, and Glenwood Formations or equivalents**." Young, H.L. (1992).
St. Peter aquifer	Sedimentary **aquifer** (consolidated or semi-consolidated rock)	"Underlying the Glenwood Shale is the **St. Peter Sandstone**, **a fine to medium-grained, well-sorted, white to buff quartz sandstone** occupying approximately 1,760 square kilometers (680 square miles) of the seven-county metropolitan area, as determined from Mossler and Tipping (2000)." (Seaberg, 2000). "**Aquifer layer**" Young, H.L. (1992)
Prairie du Chien-Jordan aquifer	Sedimentary **aquifer** (consolidated or semi-consolidated rock)	"**Aquifer layer**" Young, H.L. (1992) | "Although this **multiunit** aquifer may be the least uniform of the bedrock aquifer units in **the northern Midwest (fig. 18), it is a major source of ground water in Iowa and Minnesota**. In these States, the Jordan **Sandstone** and all or parts of the overlying Prairie du Chien Group are in direct hydraulic connection, resulting in a highly productive aquifer." Young, H.L. (1992).
St. Lawrence confining bed	Sedimentary **aquitard** (consolidated or semi-consolidated rock)	"**Confining layer**" Young, H.L. (1992) | "The St. Lawrence and Franconia Formations **form an important regional confining unit** over the Ironton-Galesville aquifer. Although these formations are **dominantly sandstone in the northern part of the area, they are very silty and shaly, fine grained, poorly sorted, and dolomitic**. Thus, **the units are anisotropic and restrict vertical movement of ground water**." Young, H.L. (1992).
Franconia-Ironton-Galesville aquifer	Sedimentary **aquifer** (consolidated or	"**Aquifer layer**" Young, H.L. (1992) | "The Ironton and Galesville **Sandstones** form the most important **aquifer** of the Cambrian-Ordovician aquifer system in the east-central part of the study area, although they generally are not the only rock

Formation name	Category	Quote
	semi-consolidated rock)	units open to deep wells." Young, H.L. (1992). | "The aquifer terminates to the west, south, and east (fig. 10) **as the sandstones grade into carbonate rocks, primarily dolomite**." Young, H.L. (1992). Franconian: "not generally regarded as an aquifer by itself" (Seaberg, 2000).
Eau Claire confining bed	Sedimentary **aquitard** (consolidated or semi-consolidated rock)	"**Confining layer**" Young, H.L. (1992) | "The Eau Claire Formation and its partial equivalent to the southwest, the Bonneterre Formation, form an extensive **confining unit** above the Mount Simon aquifer. The effectiveness of the Eau Claire as a **confining unit** depends on the relative abundance of **shale, siltstone, dolomite, and sandstone in the formation**." Young, H.L. (1992).
Mt. Simon-Hinckley aquifer	Sedimentary **aquifer** (consolidated or semi-consolidated rock)	"**Aquifer layer**" Young, H.L. (1992) | "The lowermost **aquifer** of the Cambrian-Ordovician aquifer system is composed primarily of the Mount Simon **Sandstone** and equivalent **strata**, the Lamotte Sandstone, in Missouri. In Minnesota, the Mount Simon is underlain by Precambrian sedimentary rocks-the Hinckley Sandstone and the older Fond du Lac Formation." Young, H.L. (1992).
Undifferentiated Precambrian bedrock	Endogenous bedrock	"**Impermeable boundary**" Young, H.L. (1992) | "The lowermost hydrogeologic unit is a **confining unit** that consists of generally very **low permeability crystalline rocks of the Precambrian basement** and, in Minnesota, most of the Precambrian sedimentary rocks below the Hinckley Sandstone." Young, H.L. (1992).

Young, H.L. (1992). Hydrogeology of the Cambrian-Ordovician Aquifer System in the Northern Midwest, United States. U.S. Geological Survey Professional Paper 1405-B, 108 pp. Accessed June 17, 2022 via https://pubs.usgs.gov/pp/1405b/report.pdf

Seaberg, J.K. (2000). Overview of the Twin Cities Metropolitan Groundwater Model. Minnesota Pollution Control Agency Report, 65 pp. Accessed May 18, 2022 via https://www.pca.state.mn.us/sites/default/files/mm-overview.pdf

3.42 Mesilla Valley, Rincon-Mesilla Valleys

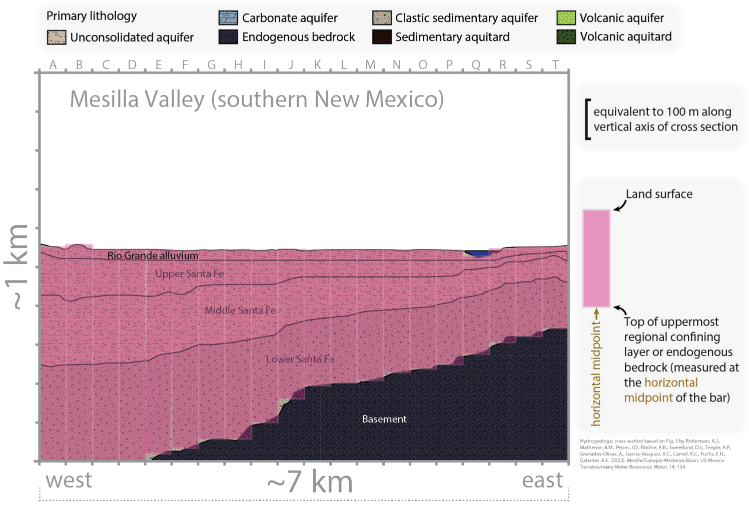

Supplementary Fig. 181. Hydrogeologic cross section. 20 equally spaced transparent pink bars overlie the cross section; each shaded bar depicts the vertical offset from the land surface to the top of the uppermost confining unit or endogenous bedrock.

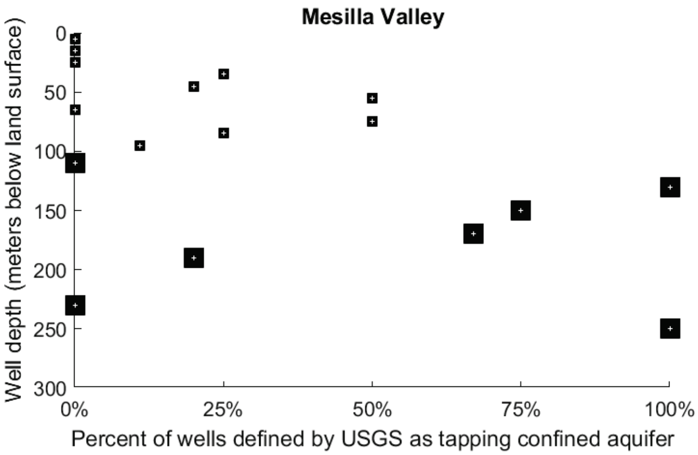

Supplementary Fig. 182. Vertical variations in the prevalence of wells that have been defined as tapping an unconfined or a confined aquifer by the USGS. The smaller squares represent 10 m depth intervals from the land surface to 100 m; the larger squares represent 20 m intervals from 100 m to 300 m below the land surface.

The Mesilla Valley is located in southern New Mexico.

(i) A hydrogeologic cross section presented in Fig. 3 by Robertson et al. (2022) does not depict a clear confining unit within the shallow portion of the aquifer system.

(ii) We analysed wells within the study area that the USGS has defined as either unconfined or confined. Most (>80%) wells at depths of 240-260 m and at depths exceeding 240 m are defined as tapping a confined aquifer.

Depth to confined conditions: 240-260 m (see (ii) above)

Reference: Robertson, A.J., Matherne, A.M., Pepin, J.D., Ritchie, A.B., Sweetkind, D.S., Teeple, A.P., Granados-Olivas, A., García-Vásquez, A.C., Carroll, K.C., Fuchs, E.H., Galanter, A.E. (2022). Mesilla/Conejos-Médanos Basin: US-Mexico Transboundary Water Resources. Water, 14, 134.

The table below presents a series of published quotes (see quotation marks denoting text quoted from another publication, which is cited following the quotation marks with the full reference written in full below the table). The leftmost column lists a title of a hydrogeologic formation depicted in the cross section on the previous page. The rightmost column presents a quote from a hydrogeological study (see base of table for citation). The quote has been annotated with colored text to highlight how we categorized each layer (i.e., see categories in the center column in the table). Specifically: (i) blue text highlights portions of a quote that provide insights into the degree of consolidation of the formation, (ii) red text highlights portions of a quote that categorize the formation as an aquifer or an aquitard (i.e., higher versus lower permeability in the context of local hydrogeologic formations), and (iii) green text highlights portions of a quote that provide information about the lithology of the formation.

Supplementary Table 45. Hydrostratigraphy details for the Mesilla Valley

Formation name	Category	Quote
Rio Grande alluvium	Unconsolidated aquifer	"**A thin layer (< 25 m) of unconsolidated Quaternary alluvial and fluvial deposits**, known as the Rio Grande alluvium, overlies the Santa Fe" Robertson et al., 2022). "The Rio Grande alluvium is composed **of river-channel and overbank depositional facies** ranging in texture from **sand and gravel to silt and clay** that are generally **15 to 38 m thick**, respectively [11]." Robertson et al., 2022). "**Groundwater levels in the Rio Grande alluvium are shallow and unconfined and generally decrease from north to south at an average gradient of about 0.8 to 1.1 m/km [38], closely following the topographic gradient**." (Robertson et al., 2022).
Upper Santa Fe	Unconsolidated aquifer	"The upper Santa Fe unit is about 3 to 4 Ma and consists of **fluvial deposits from a large, braided river of the ancestral Rio Grande**, with **channel sands and gravels** from as far north as the mountains in southern Colorado and alluvial fan deposits derived from basin-bounding highlands (Figure 1) [8]." Robertson et al., 2022). "Hawley and Kennedy (2004) estimated **the "most productive" portion of the aquifer system** (the Rio Grande alluvium, **upper Santa Fe unit**, and middle Santa Fe unit) to hold about 17,300 hm3 of available freshwater (<1000 mg/L DS) [8]." (Robertson et al., 2022).
Middle Santa Fe	Unconsolidated aquifer	"The middle Santa Fe unit is composed of **eolian dune sequences** up to 610 m thick that **intertongue with alluvial deposits** near the bounding mountains and **fluvial and playa-lake deposits** in the inner Basin [8]." (Robertson et al., 2022). "Hawley and Kennedy (2004) estimated **the "most productive" portion of the aquifer system** (the Rio Grande alluvium, upper Santa Fe unit, and **middle Santa Fe unit**) to hold about 17,300 hm3 of available freshwater (<1000 mg/L DS) [8]." (Robertson et al., 2022).
Lower Santa Fe	Clastic sedimentary **aquifer** (consolidated or semi-consolidated rock)	"The lower Santa Fe unit is **primarily fine-grained and partly consolidated** with some **calcium-sulfate and sodium-sulfate evaporites** and cementation, which was deposited in a closed basin." (Robertson et al., 2022). "specific capacity estimates of the middle Santa Fe are usually less than 8 L/s/m and between **0.2 to 2 L/s/m for the lower Santa Fe** [8]." (Robertson et al., 2022).

Formation name	Category	Quote
Basement	Endogenous bedrock	"The pre-Santa Fe Group **rocks are deformed and faulted**" (Hanson et al., 2020). "**The basement units** in the TRG were grouped into seven groups that represent the **bedrock units, Tertiary sediments, intrusive rocks, and volcanics** (table 4; Sweetkind, 2017, fig. 23)" (Hanson et al., 2020).

Robertson, A.J., Matherne, A.M., Pepin, J.D., Ritchie, A.B., Sweetkind, D.S., Teeple, A.P., Granados-Olivas, A., García-Vásquez, A.C., Carroll, K.C., Fuchs, E.H., Galanter, A.E. (2022). Mesilla/Conejos-Médanos Basin: US-Mexico Transboundary Water Resources. Water, 14, 134.

Hanson, R.T.; Ritchie, A.B.; Boyce, S.E.; Galanter, A.E.; Ferguson, I.A.; Flint, L.E., Henson, W.R. (2020). Rio Grande transboundary integrated hydrologic model and water-availability analysis, New Mexico and Texas, United States, and Northern Chihuahua, Mexico. In US Geological Survey Scientific Investigations Report 2019-5120; U.S. Geological Survey, Accessed June 5, 2022 via https://pubs.er.usgs.gov/publication/sir20195120

3.43 Lower Santa Ynez Valley, Santa Ynez Valley

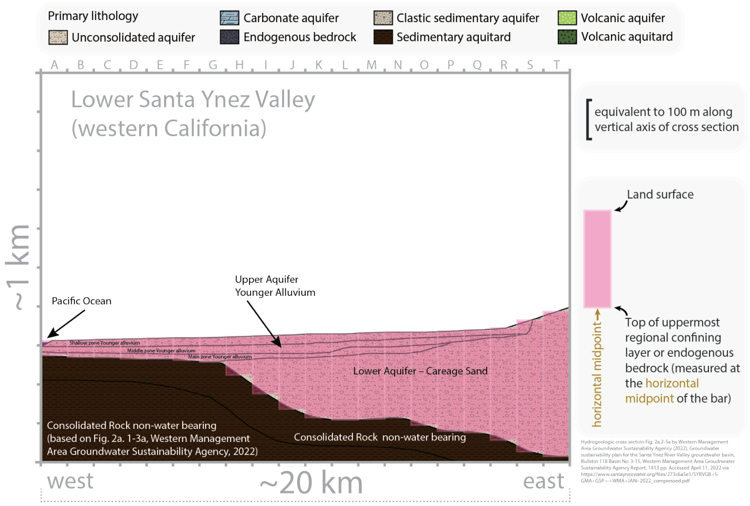

Supplementary Fig. 183. Hydrogeologic cross section. 20 equally spaced transparent pink bars overlie the cross section; each shaded bar depicts the vertical offset from the land surface to the top of the uppermost confining unit or endogenous bedrock.

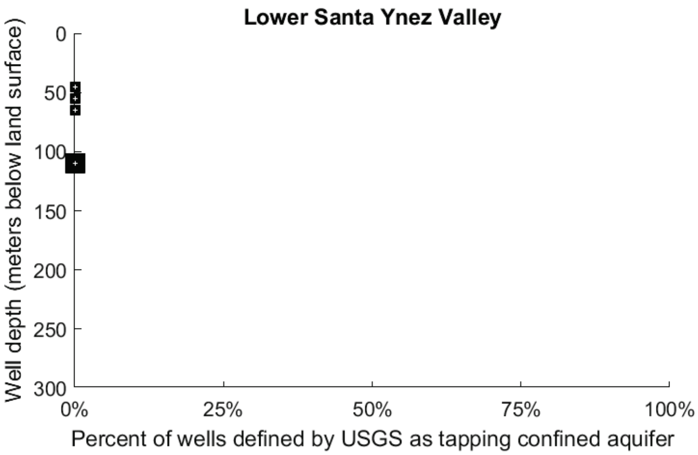

Supplementary Fig. 184. Vertical variations in the prevalence of wells that have been defined as tapping an unconfined or a confined aquifer by the USGS. The smaller squares represent 10 m depth intervals from the land surface to 100 m; the larger squares represent 20 m intervals from 100 m to 300 m below the land surface.

The Lower Santa Ynez Valley is located in western California.

(i) A hydrogeologic cross section presented in Fig. 2a.2-5a by the Western Management Area Groundwater Sustainability Agency (2022) suggests that the uppermost confining unit is typically 202 meters below land surface (25th-75th percentile range: 56-243 meters below land surface).

(ii) We analysed wells within the study area that the USGS has defined as either unconfined or confined. All n=4 wells (with depths ranging from 49 m to 117 m) are classified as unconfined.

(iii) Regarding confined conditions, the Western Management Area Groundwater Sustainability Agency (2022) states (quote) *"The main zone throughout most of the Lompoc Plain subarea is separated from the middle zone by lenses of silt and clay that result in confined or partially confined conditions in the main zone. However, in the eastern, southern, and northern portions of the Lompoc Plain subarea, the confining deposits are less continuous or absent…"*

Depth to confined conditions: 200-220 m based on (i) and (iii)

References: Western Management Area Groundwater Sustainability Agency (2022). Groundwater sustainability plan for the Santa Ynez River Valley groundwater basin, Bulletin 118 Basin No. 3-15, Western Management Area Groundwater Sustainability Agency Report, 1413 pp. Accessed April 11, 2022 via https://www.santaynezwater.org/files/273c6a5e1/SYRVGB+SGMA+GSP+-+WMA+JAN+2022_compressed.pdf

The table below presents a series of published quotes (see quotation marks denoting text quoted from another publication, which is cited following the quotation marks with the full reference written in full below the table). The leftmost column lists a title of a hydrogeologic formation depicted in the cross section on the previous page. The rightmost column presents a quote from a hydrogeological study (see base of table for citation). The quote has been annotated with colored text to highlight how we categorized each layer (i.e., see categories in the center column in the table). Specifically: (i) blue text highlights portions of a quote that provide insights into the degree of consolidation of the formation, (ii) red text highlights portions of a quote that categorize the formation as an aquifer or an aquitard (i.e., higher versus lower permeability in the context of local hydrogeologic formations), and (iii) green text highlights portions of a quote that provide information about the lithology of the formation.

Supplementary Table 46. Hydrostratigraphy details for the Lower Santa Ynez Valley

Formation name	Category	Quote
Upper Aquifer-Younger Alluvium	Unconsolidated aquifer	"**Groundwater occurs** in **thin, unconsolidated** sedimentary layers of younger **alluvium** directly over non-water-bearing, consolidated geologic units (Section 2a.2)." (WMAGSA, 2020). "**an Upper Aquifer**, consisting of younger **alluvial sediments that are primarily associated with river and surface water geomorphic processes**," (WMAGSA, 2020). "a **Lower Aquifer**, which is more extensive throughout the Basin and consists of **older geologic depositions**." (WMAGSA, 2020).
Lower Aquifer – Careage Sand Tca	Unconsolidated aquifer *(unconfined aquifer, and semi-confined aquifer)*	"For groundwater management purposes, two principal aquifers were defined based on the Lompoc Plain location: the Upper Aquifer, which consists of alluvial sediments, and the **Lower Aquifer**, which consists of the **water-bearing Careaga Sand and Paso Robles Formation**." (WMAGSA, 2020). "The Santa Rita Upland contains **unconsolidated water-bearing principal aquifer units of the Lower Aquifer** within an east/west-trending geologic syncline fold." (WMAGSA, 2020).
Consolidated Rock-non-water bearing	Clastic sedimentary rock **aquitard** (consolidated or semi-consolidated rock)	"**Non-water-bearing consolidated geologic units** also form the lateral boundaries as exposed bedrock in this area." (WMAGSA, 2020). "Tertiary-Mesozoic Rocks are **consolidated non-water bearing units, all of marine origin. They consist of the near-shore marine Foxen (Tf), Sisquoc (Tsq), and Monterey (Tm) Formations**." (WMAGSA, 2020).

Western Management Area Groundwater Sustainability Agency (2020). Groundwater sustainability plan for the Santa Ynez River Valley groundwater basin bulletin 118 basin no. 3-15 western management area groundwater sustainability agency.
https://www.santaynezwater.org/files/273c6a5e1/SYRVGB+SGMA+GSP+-+WMA+JAN+2022_compressed.pdf

3.44 Boise Valley and Homedale Murphy Area, Western Snake River Plain

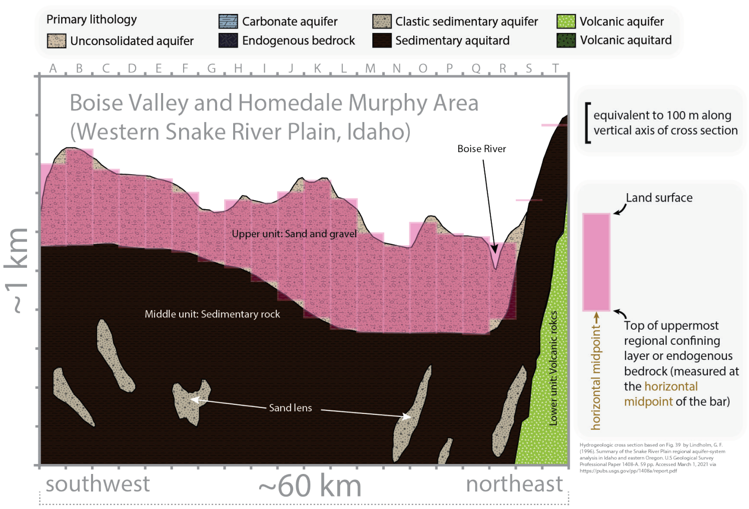

Supplementary Fig. 185. Hydrogeologic cross section. 20 equally spaced transparent pink bars overlie the cross section; each shaded bar depicts the vertical offset from the land surface to the top of the uppermost confining unit or endogenous bedrock.

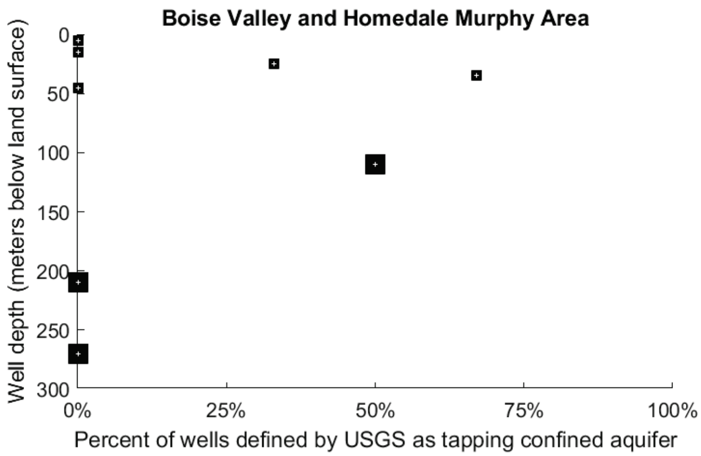

Supplementary Fig. 186. Vertical variations in the prevalence of wells that have been defined as tapping an unconfined or a confined aquifer by the USGS. The smaller squares represent 10 m depth intervals from the land surface to 100 m; the larger squares represent 20 m intervals from 100 m to 300 m below the land surface.

The Boise Valley and Homedale Murphy Area is located in Idaho.

(i) A hydrogeologic cross section presented in Fig. 39 by Lindholm (1996) suggests that the upper unit (primarily sand and gravel) is underlain by a confining unit ("Middle unit").

(ii) We analysed wells within the study area that the USGS has defined as either unconfined or confined. Two of the three deepest wells in the dataset (depths range from 209 m to 372 m) as classified as unconfined; the deepest well (372 m deep) is classified as confined. The available USGS well data are insufficient to evaluate the depths at which the aquifer system transitions from unconfined to confined conditions.

Depth to confined conditions: >260 m (see (ii) above)

Reference: Lindholm, G. F. (1996). Summary of the Snake River Plain regional aquifer-system analysis in Idaho and eastern Oregon. U.S Geological Survey Professional Paper 1408-A. 59 pp. Accessed March 1, 2021 via https://pubs.usgs.gov/pp/1408a/report.pdf

The table below presents a series of published quotes (see quotation marks denoting text quoted from another publication, which is cited following the quotation marks with the full reference written in full below the table). The leftmost column lists a title of a hydrogeologic formation depicted in the cross section on the previous page. The rightmost column presents a quote from a hydrogeological study (see base of table for citation). The quote has been annotated with colored text to highlight how we categorized each layer (i.e., see categories in the center column in the table). Specifically: (i) blue text highlights portions of a quote that provide insights into the degree of consolidation of the formation, (ii) red text highlights portions of a quote that categorize the formation as an aquifer or an aquitard (i.e., higher versus lower permeability in the context of local hydrogeologic formations), and (iii) green text highlights portions of a quote that provide information about the lithology of the formation.

Supplementary Table 47. Hydrostratigraphy details for Boise Valley and Homedale Murphy Area

**Formation name**	**Category**	**Quote**
Upper unit: Sand and gravel	Unconsolidated aquifer	"The **most productive aquifers** in the 4,800-square-mile western plain are **alluvial sand and gravel** in the Boise River valley." (Lindholm, 1996). "**Alluvium** is thickest (several hundred feet) and the percentage of **sand and gravel largest** in the Boise River valley." (Lindholm, 1996).
Middle unit: Sedimentary Unit	Clastic sedimentary **aquitard** (consolidated or semi-consolidated rock)	"Pre-Tertiary **sedimentary rocks** in the eastern part of the basin (fig. 8) are generally a **complex of shale, argillite, sandstone, and limestone**." (Lindholm, 1996). "Temporal changes **in ground-water solute concentrations** in the Snake River Plain are poorly defined. Low (1987) noted that concentrations of dissolved solids and chloride are generally greatest in areas of **fine-grained sedimentary rocks** and in intensively irrigated areas." (Lindholm, 1996).
Lower Unit: Volcanic rock	Volcanic aquifer	"**Tertiary volcanic rocks** east of the batholith range from **rhyodacite to basalt**." (Lindholm, 1996). "**Very low; confining unit High; may be extremely high Horizontal: low to moderate, depends on vesicularity and degree of fracturing Vertical: depends on degree of fracturing; commonly several orders of magnitude lower than horizontal conductivity**" (Lindholm, 1996).

Lindholm, G. F. (1996). Summary of the Snake River Plain regional aquifer-system analysis in Idaho and eastern Oregon. U.S Geological Survey Professional Paper 1408-A. 59 pp. Accessed March 1, 2021 via https://pubs.usgs.gov/pp/1408a/report.pdf

3.45 Mountain Home Plateau, Western Snake River Plain

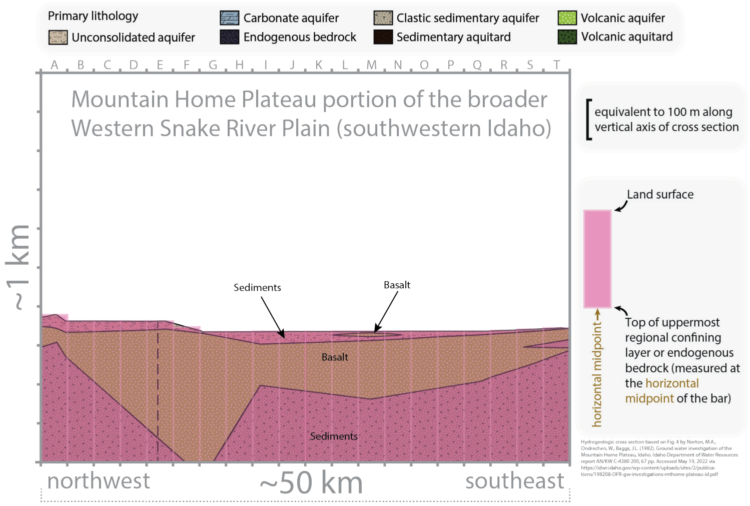

Supplementary Fig. 187. Hydrogeologic cross section. 20 equally spaced transparent pink bars overlie the cross section; each shaded bar depicts the vertical offset from the land surface to the top of the uppermost confining unit or endogenous bedrock.

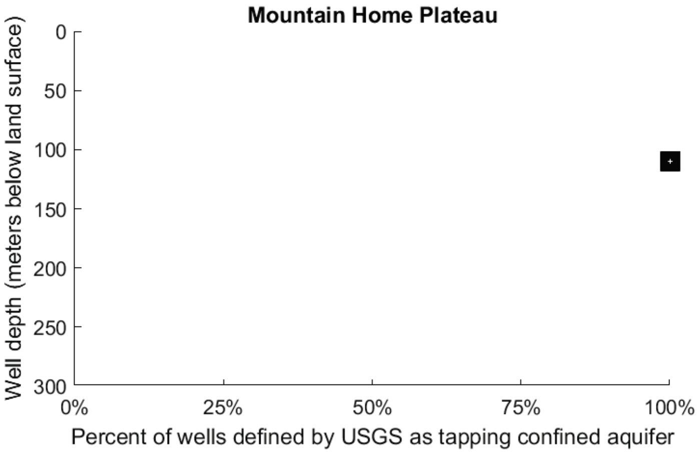

Supplementary Fig. 188. Vertical variations in the prevalence of wells that have been defined as tapping an unconfined or a confined aquifer by the USGS. The smaller squares represent 10 m depth intervals from the land surface to 100 m; the larger squares represent 20 m intervals from 100 m to 300 m below the land surface.

The Mountain Home Plateau subarea of the broader Western Snake River Plain is located in southwestern Idaho.

(i) A hydrogeologic cross section presented in Fig. 4 by Norton et al. (1982) for the Mountain Home Plateau does not depict a clear confining unit. The median depth to a confined unit exceeds 336 meters below land surface (median of pink transparent bars in cross section; the 25th-75th percentile range is >336 m to >351 m).

(ii) There is only n=1 well (with a depth of 112 m) that the USGS has defined as tapping either a confined or unconfined aquifer; this single data point is insufficient to evaluate the depths at which the aquifer system transitions from unconfined to confined conditions.

Depth to confined conditions: >336 m (based on (i))

Reference: Norton, M.A., Ondrechen, W., Baggs, J.L. (1982). Ground water investigation of the Mountain Home Plateau, Idaho. Idaho Department of Water Resources report AN/KW C-4380 200, 67 pp. Accessed May 19, 2022 via https://idwr.idaho.gov/wp-content/uploads/sites/2/publications/198208-OFR-gw-investigations-mthome-plateau-id.pdf

The table below presents a series of published quotes (see quotation marks denoting text quoted from another publication, which is cited following the quotation marks with the full reference written in full below the table). The leftmost column lists a title of a hydrogeologic formation depicted in the cross section on the previous page. The rightmost column presents a quote from a hydrogeological study (see base of table for citation). The quote has been annotated with colored text to highlight how we categorized each layer (i.e., see categories in the center column in the table). Specifically: (i) blue text highlights portions of a quote that provide insights into the degree of consolidation of the formation, (ii) red text highlights portions of a quote that categorize the formation as an aquifer or an aquitard (i.e., higher versus lower permeability in the context of local hydrogeologic formations), and (iii) green text highlights portions of a quote that provide information about the lithology of the formation.

Supplementary Table 48. Hydrostratigraphy details for the Mountain Home Plateau

**Formation name**	**Category**	**Quote**
Sediment	Unconsolidated aquifer	"**alluvium and younger terrace gravels**" (Norton et al., 1982). "**Unconsolidated clay, silt, sand, and gravel occurring** beneath flood plains of Boise and Snake Rivers." (Norton et al., 1982). "**Hydraulic conductivity generally high**: however, because of thinness and irregularity of beds, yields to wells are generally small to moderate." (Norton et al., 1982).
Basalt (assumed to be Basalt of Snake River Group from Table 1 by Norton et al. 1982)	Volcanic aquifer	"Vesicular olivine **basalt**, light to dark gray, irregular to columnar jointing." "Hydraulic conductivity variable. Where saturated, reported **well yields range fran 20 to 3,100 gal/min**" (Norton et al., 1982).
Sediment (assumed to be Older Terrace Gravel from Table 1 by Norton et al. 1982)	Sedimentary rock **aquifer** (consolidated or semi-consolidated rock)	"**Unconsolidated clay, sand, and fine to coarse gravel.**" (Norton et al., 1982). "**Hydraulic conductivity generally high**." (Norton et al., 1982).

Norton, M.A., Ondrechen, W., Baggs, J.L. (1982). Ground water investigation of the Mountain Home Plateau, Idaho. Idaho Department of Water Resources report AN/KW C-4380 200, 67 pp. Accessed May 19, 2022 via https://idwr.idaho.gov/wp-content/uploads/sites/2/publications/198208-OFR-gw-investigations-mthome-plateau-id.pdf

3.46 Antelope Valley

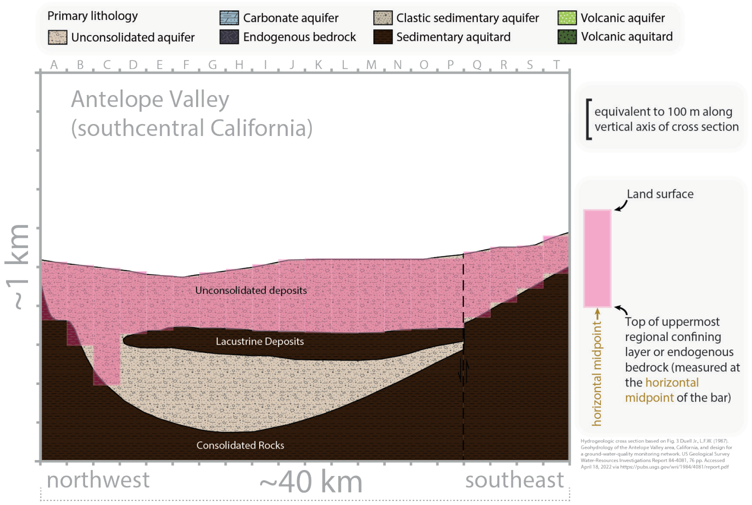

Supplementary Fig. 189. Hydrogeologic cross section. 20 equally spaced transparent pink bars overlie the cross section; each shaded bar depicts the vertical offset from the land surface to the top of the uppermost confining unit or endogenous bedrock.

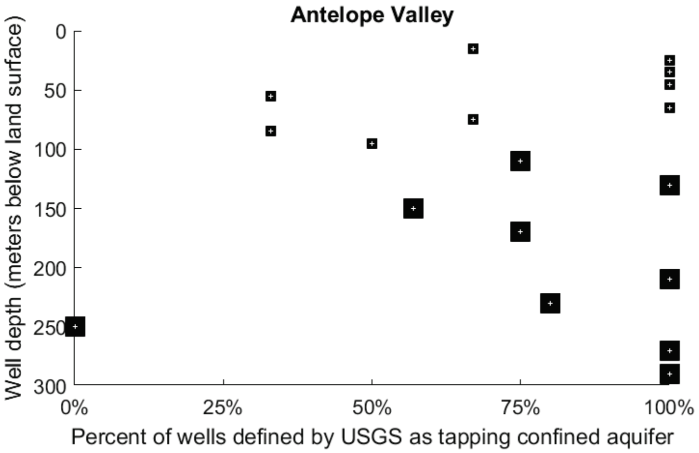

Supplementary Fig. 190. Vertical variations in the prevalence of wells that have been defined as tapping an unconfined or a confined aquifer by the USGS. The smaller squares represent 10 m depth intervals from the land surface to 100 m; the larger squares represent 20 m intervals from 100 m to 300 m below the land surface.

The Antelope Valley is located in southcentral California.

(i) A hydrogeologic cross section presented in Fig. 3 by Duell Jr. (1987) suggests that shallow unconsolidated deposits are underlain by a confining unit comprised of lacustrine clay.

(ii) We analysed wells within the study area that the USGS has defined as either unconfined or confined. Most (>80%) wells at depths of x-y m and at depths exceeding y m are defined as tapping a confined aquifer.

Depth to confined conditions: 120-140 m (see (ii) above)

Reference: Duell Jr., L.F.W. (1987). Geohydrology of the Antelope Valley area, California, and design for a ground-water-quality monitoring network. US Geological Survey Water-Resources Investigations Report 84-4081, 76 pp. Accessed April 18, 2022 via https://pubs.usgs.gov/wri/1984/4081/report.pdf

The table below presents a series of published quotes (see quotation marks denoting text quoted from another publication, which is cited following the quotation marks with the full reference written in full below the table). The leftmost column lists a title of a hydrogeologic formation depicted in the cross section on the previous page. The rightmost column presents a quote from a hydrogeological study (see base of table for citation). The quote has been annotated with colored text to highlight how we categorized each layer (i.e., see categories in the center column in the table). Specifically: (i) blue text highlights portions of a quote that provide insights into the degree of consolidation of the formation, (ii) red text highlights portions of a quote that categorize the formation as an aquifer or an aquitard (i.e., higher versus lower permeability in the context of local hydrogeologic formations), and (iii) green text highlights portions of a quote that provide information about the lithology of the formation.

Supplementary Table 49. Hydrostratigraphy details for the Antelope Valley

Formation name	Category	Quote
Unconsolidated deposits	Unconsolidated aquifer	"**The unconsolidated deposits that** underlie Antelope Valley (pi. 1) include **younger and older alluvium, older fan deposits, windblown dune sand, and playa deposits**. **These deposits comprise the aquifers of the area**." Duell, (1987). "The older alluvium is **porous and permeable** and **yields water freely, and is the most important water-bearing unit**." Duell (1987).
Lacustrine deposits	Sedimentary **aquitard** (consolidated or semi-consolidated rock)	"**Playa or lacustrine deposits** of Pliocene through Holocene age are composed of **siltstone, clay, and marl**. During pluvial periods, or times of relatively heavy precipitation, massive beds of blue clay formed in deep, perennial lakes. Individual **clay beds are locally as much as 400 feet thick**. **These beds are interbedded with lenses of coarser material as much as 20 feet thick**. **The clay yields virtually no water to wells, but interbedded materials supply some water to wells**." Duell (1987).
Consolidated rocks	Sedimentary rock **aquitard** (consolidated or semi-consolidated rock)	"**Consolidated rocks** surround Antelope Valley and form the sides and bottom of the ground-water basin (pi. 1)." Duell, (1987). "**Consolidated sedimentary rocks of Tertiary age yield little water, if any**." Duell (1987).

Duell, L. F. (1987). Geohydrology of the Antelope Valley Area, California and design for a ground-water-quality monitoring network (Vol. 84, No. 4081). Department of the Interior, US Geological Survey. https://pubs.er.usgs.gov/publication/wri844081

3.47 Big Bear Valley

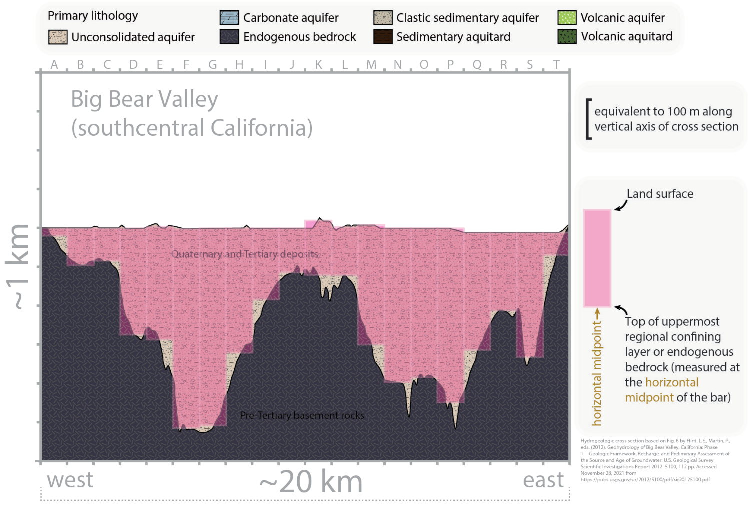

Supplementary Fig. 191. Hydrogeologic cross section. 20 equally spaced transparent pink bars overlie the cross section; each shaded bar depicts the vertical offset from the land surface to the top of the uppermost confining unit or endogenous bedrock.

Big Bear Valley is located in southcentral California.

(i) A hydrogeologic cross section presented in Fig. 6 by Flint et al. (2012) suggests that the aquifer system does not host a clear confining unit within the aquifer system. Pre-Tertiary basement rocks underlie the Quaternary and Tertiary deposits; the typical (i.e., median) depth to these basement rocks as depicted in the cross section is 282 meters below the land surface (see pink shaded bars in the cross section to the left).

(ii) We did not identify sufficient wells within the valley for which the USGS has defined the well as tapping an unconfined or confined aquifer.

Depth to confined conditions: 280-300 m (see (i) above)

Reference: Flint, L.E., Martin, P., eds. (2012). Geohydrology of Big Bear Valley, California: Phase 1—Geologic Framework, Recharge, and Preliminary Assessment of the Source and Age of Groundwater: U.S. Geological Survey Scientific Investigations Report 2012–5100, 112 pp. Accessed November 28, 2021 from https://pubs.usgs.gov/sir/2012/5100/pdf/sir20125100.pdf

The table below presents a series of published quotes (see quotation marks denoting text quoted from another publication, which is cited following the quotation marks with the full reference written in full below the table). The leftmost column lists a title of a hydrogeologic formation depicted in the cross section on the previous page. The rightmost column presents a quote from a hydrogeological study (see base of table for citation). The quote has been annotated with colored text to highlight how we categorized each layer (i.e., see categories in the center column in the table). Specifically: (i) blue text highlights portions of a quote that provide insights into the degree of consolidation of the formation, (ii) red text highlights portions of a quote that categorize the formation as an aquifer or an aquitard (i.e., higher versus lower permeability in the context of local hydrogeologic formations), and (iii) green text highlights portions of a quote that provide information about the lithology of the formation.

Supplementary Table 50. Hydrostratigraphy details for the Big Bear Valley

**Formation name**	**Category**	**Quote**
Quaternary and Tertiary deposits	Unconsolidated aquifer	"**Most of the water supply** for the Big Bear area is **pumped** from the **unconsolidated Quaternary alluvial deposits** in the groundwater basin. **The water-bearing deposits in the groundwater basin have been classified into upper, middle, and lower aquifers (GeoScience Support Services, Inc., 1999), and the upper and middle aquifers are the primary water producers**. Inspection of geologic logs from wells drilled in the Baldwin Lake area indicates that the **deposits consist of sands and gravels with interbedded clays.**" (Flint et al., 2012)
Pre-Tertiary basement rocks	Endogenous bedrock	"**The basement rocks are dominated by (1) large Cretaceous granitic bodies ranging in composition from monzogranite to gabbro, (2) metamorphosed sedimentary rocks ranging in age from late Paleozoic to late Proterozoic, and (3) Middle Proterozoic gneiss** (Miller, 2004). These rocks are complexly deformed by normal, reverse, and thrust faults, and are tightly folded in some places (Miller, 2004). **In general, the basement rocks are of low permeability** and **are not considered a major water-bearing unit except in fractures and weathered zones that can create shallow perched groundwater zones**." (Flint et al., 2012)

Flint, L.E., Martin, P., eds., with contributions by Brandt, J., Christensen, A.H., Flint, A.L., Flint, L.E., Hevesi, J.A., Jachens, R., Kulongoski, J.T., Martin, P., Sneed, M. (2012). Geohydrology of Big Bear Valley, California: Phase 1—Geologic Framework, Recharge, and Preliminary Assessment of the Source and Age of Groundwater: U.S. Geological Survey Scientific Investigations Report 2012–5100, 112 p.

3.48 Bighorn Basin

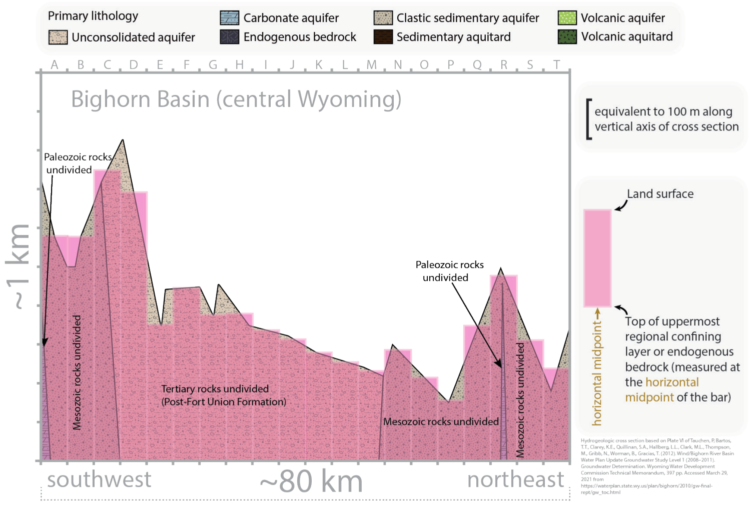

Supplementary Fig. 192. Hydrogeologic cross section. 20 equally spaced transparent pink bars overlie the cross section; each shaded bar depicts the vertical offset from the land surface to the top of the uppermost confining unit or endogenous bedrock.

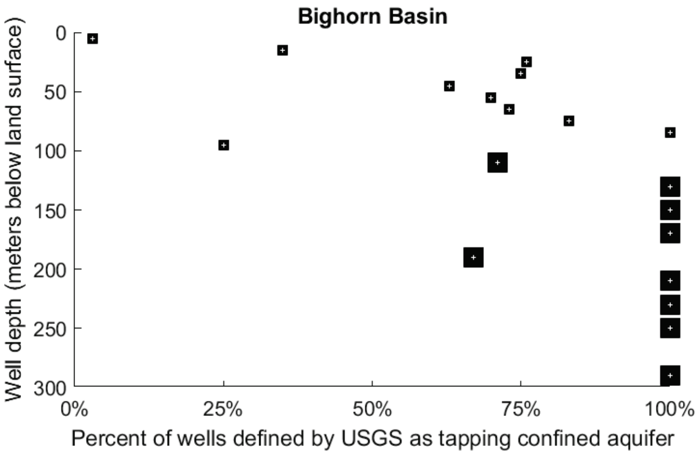

Supplementary Fig. 193. Vertical variations in the prevalence of wells that have been defined as tapping an unconfined or a confined aquifer by the USGS. The smaller squares represent 10 m depth intervals from the land surface to 100 m; the larger squares represent 20 m intervals from 100 m to 300 m below the land surface.

The Bighorn Basin is located in central Wyoming.

(i) A hydrogeologic cross section presented in Plate VI by Tauchen et al. (2012) suggests that the aquifer system does not have a single and continuous confining unit at shallow depths.

(ii) We analysed wells within the study area that the USGS has defined as either unconfined or confined. Most (>80%) wells at depths of 70-80 m and at depths exceeding 120 m are defined as tapping a confined aquifer.

Depth to confined conditions: 70-80 m (see (ii) above)

Reference: Tauchen, P, Bartos, T.T., Clarey, K.E., Quillinan, S.A., Hallberg, L.L., Clark, M.L., Thompson, M., Gribb, N., Worman, B., Gracias, T. (2012). Wind/Bighorn River Basin Water Plan Update Groundwater Study Level 1 (2008–2011). Groundwater Determination. Wyoming Water Development Commission Technical Memorandum, 397 pp. Accessed March 29, 2021 from https://waterplan.state.wy.us/plan/bighorn/2010/gw-finalrept/gw_toc.html

The table below presents a series of published quotes (see quotation marks denoting text quoted from another publication, which is cited following the quotation marks with the full reference written in full below the table). The leftmost column lists a title of a hydrogeologic formation depicted in the cross section on the previous page. The rightmost column presents a quote from a hydrogeological study (see base of table for citation). The quote has been annotated with colored text to highlight how we categorized each layer (i.e., see categories in the center column in the table). Specifically: (i) blue text highlights portions of a quote that provide insights into the degree of consolidation of the formation, (ii) red text highlights portions of a quote that categorize the formation as an aquifer or an aquitard (i.e., higher versus lower permeability in the context of local hydrogeologic formations), and (iii) green text highlights portions of a quote that provide information about the lithology of the formation.

Supplementary Table 51. Hydrostratigraphy details for the Bighorn Basin

Formation name	Category	Quote
Tu (Tertiary rocks undivided (Post-Fort Union Formation) *(confining unit exists)*	Clastic sedimentary **aquifer** (consolidated or semi-consolidated rock)	"Below the major alluvial aquifer at the land surface and above the thick, widespread Upper Cretaceous ***Cody*** *[Major]* ***confining unit*** are three Tertiary or Upper Cretaceous major (**mostly sandstone**) aquifers (Fort Union, Lance, Mesaverde) and one major confining unit (Meeteetse), all components of the **lower Tertiary/Upper Cretaceous aquifer system**; the aquifers are generally accessible in the central basins." Tauchen et al. (2012) "**Aquifers** within the system are **lenticular, discontinuous sandstone bodies** that are hydraulically isolated to various degrees by interbedded fine-grained confining units" Tauchen et al. (2012).
Mzu (Mesozoic rocks undivided) *(confining unit exists)*	Clastic sedimentary **aquifer** (consolidated or semi-consolidated rock)	"The early Mesozoic Era was a time **of shallow seas with deposition of interbedded layers (in decreasing abundance) of sandstone, siltstone, shale, carbonates, and evaporates**. An emergent transition to a terrestrial environment during the Late Triassic and Early Jurassic Epochs **resulted in the deposition of marginal marine, eolian, fluvial, and paludal sandstones and shales**." Tauchen et al. (2012) "The **Paleozoic aquifer system** comprises Permian through Ordovician **carbonate and sandstone** hydrogeologic units." Tauchen et al. (2012) "Immediately below the Cody [Major] confining unit, **the lower and middle Mesozoic aquifers** and confining units system comprises Cretaceous through Jurassic hydrogeologic units." Tauchen et al. (2012).
Pzu (Paleozoic rocks undivided)	Carbonate aquifer	"Paleozoic **strata** in the WBRB were **deposited in marine and nonmarine transgressive/regressive environments**. **Marine limestones and dolomites are the dominant lithologies of the Paleozoic sequence, with less extensive sandstones and shales** that represent beach and near-shore environments." Tauchen et al. (2012) "The **Paleozoic aquifer system** comprises Permian through Ordovician **carbonate and sandstone** hydrogeologic units." Tauchen et al. (2012) "Immediately below the Cody [Major] confining unit, **the lower and middle Mesozoic aquifers** and confining units system comprises Cretaceous through Jurassic hydrogeologic units." Tauchen et al. (2012).

Tauchen, P, Bartos, T.T., Clarey, K.E., Quillinan, S.A., Hallberg, L.L., Clark, M.L., Thompson, M., Gribb, N., Worman, B., Gracias, T. (2012). Wind/Bighorn River Basin Water Plan Update Groundwater Study Level 1 (2008–2011). Groundwater Determination. Wyoming Water Development Commission Technical Memorandum, 397 pp. Accessed June 5, 2022 from https://waterplan.state.wy.us/plan/bighorn/2010/gw-finalrept/gw-finalrept.pdf

3.49 Black Hills Uplift

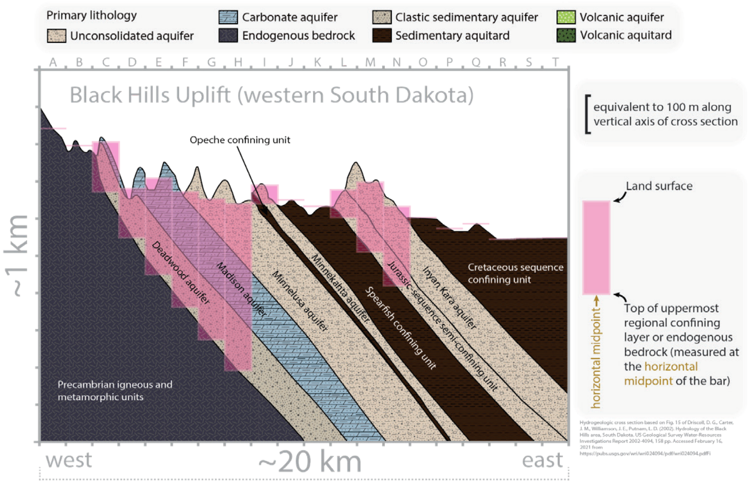

Supplementary Fig. 194. Hydrogeologic cross section. 20 equally spaced transparent pink bars overlie the cross section; each shaded bar depicts the vertical offset from the land surface to the top of the uppermost confining unit or endogenous bedrock.

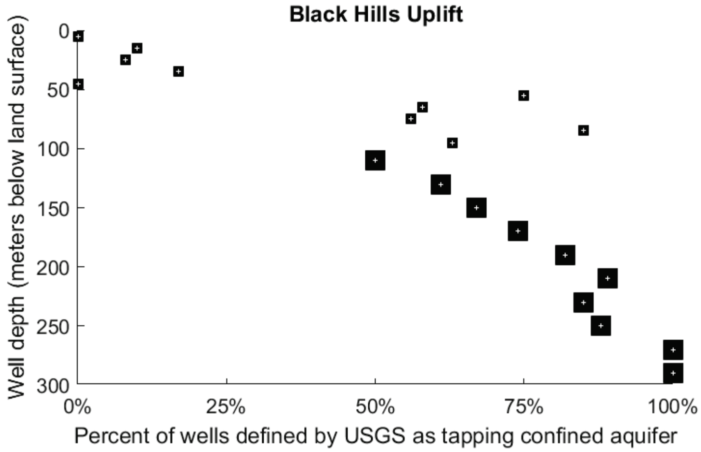

Supplementary Fig. 195. Vertical variations in the prevalence of wells that have been defined as tapping an unconfined or a confined aquifer by the USGS. The smaller squares represent 10 m depth intervals from the land surface to 100 m; the larger squares represent 20 m intervals from 100 m to 300 m below the land surface.

The Black Hills Uplift is located in western South Dakota.

(i) A hydrogeologic cross section presented in Fig. 25 by Driscoll et al. (2002) depicts a series of sedimentary rock units dipping to the east, with numerous confining units including the Spearfish confining unit and the Opeche confining unit.

(ii) We analysed wells within the study area that the USGS has defined as either unconfined or confined. Most (>80%) wells at depths of 180-200 m and at depths exceeding 180 m are defined as tapping a confined aquifer.

Depth to confined conditions: 180-200 m (see (ii) above)

Reference: Driscoll, D. G., Carter, J. M., Williamson, J. E., Putnam, L. D. (2002). Hydrology of the Black Hills area, South Dakota. US Geological Survey Water-Resources Investigations Report 2002-4094, 158 pp. Accessed February 16, 2021 from https://pubs.usgs.gov/wri/wri024094/pdf/wri024094.pdfFi

The table below presents a series of published quotes (see quotation marks denoting text quoted from another publication, which is cited following the quotation marks with the full reference written in full below the table). The leftmost column lists a title of a hydrogeologic formation depicted in the cross section on the previous page. The rightmost column presents a quote from a hydrogeological study (see base of table for citation). The quote has been annotated with colored text to highlight how we categorized each layer (i.e., see categories in the center column in the table). Specifically: (i) blue text highlights portions of a quote that provide insights into the degree of consolidation of the formation, (ii) red text highlights portions of a quote that categorize the formation as an aquifer or an aquitard (i.e., higher versus lower permeability in the context of local hydrogeologic formations), and (iii) green text highlights portions of a quote that provide information about the lithology of the formation.

Supplementary Table 52. Hydrostratigraphy details for the Black Hill Uplift

**Formation name**	**Category**	**Quote**
Unconsolidated units	Unconsolidated aquifer	"**Unconsolidated deposits** of Tertiary or Quaternary age, including **alluvium, colluvium, and wind-blown deposits**, all have the potential to be **local aquifers where they are saturated**." (Driscoll et al., 2002).
Cretaceous sequence confining unit *(this unit is predominantly aquitard)*	Sedimentary rock **aquitard** (consolidated or semi-consolidated rock)	"The **Cretaceous-sequence confining unit** mainly includes **shales of low permeability, such as the Pierre Shale**; local aquifers in the **Pierre Shale** are referred to as the **Pierre aquifer** in this report." (Driscoll et al., 2002).
Inyan Kara aquifer	Clastic sedimentary rock **aquifer** (consolidated or semi-consolidated rock)	"The Cretaceous-age Inyan Kara Group consists of the **Lakota Formation and overlying Fall River Formation**. The Lakota Formation consists of the **Chilson, Minnewaste Limestone, and Fuson Shale members. The Lakota Formation consists of yellow, brown, and reddish-brown massive to thinly bedded sandstone, pebble conglomerate, siltstone, and claystone of fluvial origin** (Gott and others, 1974); **locally there are lenses of limestone and coal**. The Fall River Formation is **a brown to reddish-brown, fine-grained sandstone, thin bedded at the top and massive at the bottom** (Strobel and others, 1999)." (Driscoll et al., 2002). "The **Inyan Kara aquifer generally has the highest effective porosity of the major aquifers**." (Driscoll et al., 2002).
Jurassic-sequence semi-confining unit *(this unit is predominantly aquitard)*	Sedimentary **aquitard** (consolidated or semi-consolidated rock)	"The **Jurassic-sequence semiconfining unit** consists of **shales and sandstones**. Overall, **this unit is semiconfining because of the low permeability of the interbedded shales**" (Driscoll et al., 2002).
Spearfish confining unit *(this unit is predominantly aquitard)*	Sedimentary **aquitard** (consolidated or semi-consolidated rock)	"**Red silty shale, soft red sandstone and siltstone with gypsum and thin limestone layers**. **Gypsum locally near the base**." (Driscoll et al., 2002). "**Spearfish confining unit**" (Driscoll et al., 2002).
Minnekahta aquifer	Carbonate aquifer	"**The Permian-age Minnekahta Limestone is a fine-grained, purple to gray laminated limestone** (Strobel and others, 1999), **which ranges in thickness from 25 to 65 ft** in the study area." (Driscoll et al., 2002). "**Transmissivity and hydraulic conductivity**

Formation name	Category	Quote
		**also may be high in the Minnelusa aquifer**." (Driscoll et al., 2002).
Opeche confining unit	Sedimentary **aquitard** (consolidated or semi-consolidated rock)	"**Red shale and sandstone**." (Driscoll et al., 2002). "**Opeche confining unit**" (Driscoll et al., 2002).
Minnelusa aquifer	Clastic sedimentary **aquifer** (consolidated or semi-consolidated rock)	"The Pennsylvanian- and Permian-age Minnelusa Formation consists mostly of yellow to red **crossstratified sandstone, limestone, dolomite, and shale** (Strobel and others, 1999)." (Driscoll et al., 2002). "**The Pennsylvanian (or Minnelusa) aquifer** is contained within the **sandstones and limestones of the Minnelusa Formation, Tensleep Sandstone, Amsden Formation, and equivalents of Pennsylvanian age** (fig. 12)." (Driscoll et al., 2002).
Madison aquifer	Carbonate aquifer	The **Mississippian (or Madison) aquifer** is contained within the **limestones, siltstones, sandstones, and dolomite of the Madison Limestone or Group**. Generally, water in the Mississippian aquifer is confined except in outcrop areas." (Driscoll et al., 2002).
Deadwood aquifer	Clastic sedimentary **aquifer** (consolidated or semi-consolidated rock)	"The **Cambrian-Ordovician (or Deadwood) aquifer** is contained within the **sandstones of Cambrian age (Deadwood Formation and equivalents) and limestones of Ordovician age** (Red River Formation and equivalents) (fig. 12)." (Driscoll et al., 2002).
Precambrian igneous and metamorphic units	Endogenous bedrock	"**Schist, slate, quartzite, and arkosic grit. Intruded by diorite, metamorphosed to amphibolite, and by granite and pegmatite**." (Driscoll et al., 2002).

Driscoll, D. G., Carter, J. M., Williamson, J. E., Putnam, L. D. (2002). Hydrology of the Black Hills area, South Dakota. US Geological Survey Water-Resources Investigations Report 2002-4094, 158 pp. Accessed May 31, 2022 from https://pubs.usgs.gov/wri/wri024094/pdf/wri024094.pdf

3.50 Black Warrior River Aquifer System (Eutaw and McShan Formations and Tuscaloosa Group)

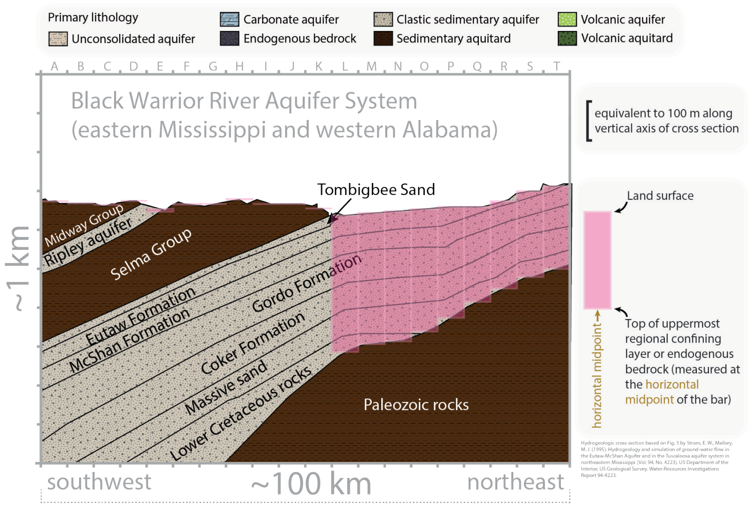

Supplementary Fig. 196. Hydrogeologic cross section. 20 equally spaced transparent pink bars overlie the cross section; each shaded bar depicts the vertical offset from the land surface to the top of the uppermost confining unit or endogenous bedrock.

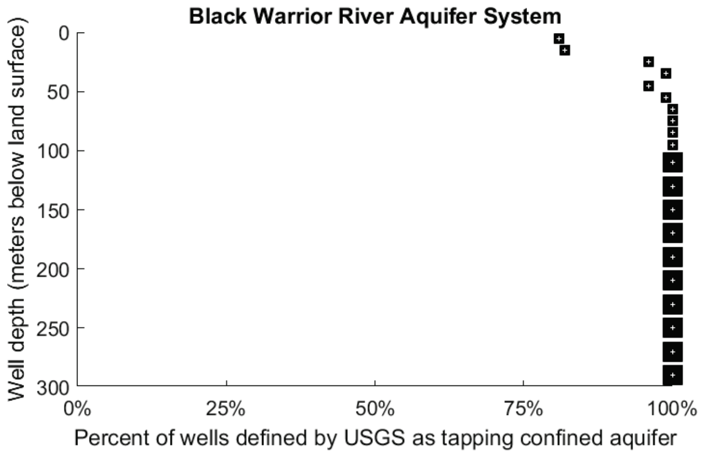

Supplementary Fig. 197. Vertical variations in the prevalence of wells that have been defined as tapping an unconfined or a confined aquifer by the USGS. The smaller squares represent 10 m depth intervals from the land surface to 100 m; the larger squares represent 20 m intervals from 100 m to 300 m below the land surface.

The Black Warrior River Aquifer System is located in eastern Mississippi and western Alabama.

(i) A hydrogeologic cross section presented in Fig. 3 by Strom and Mallory (1995) suggests that the shallowest confining unit is typically 3.5 m below the land surface, but with wide variability along the cross section (25th-75th percentile range of the depth to uppermost confining unit is 0m to 255 m).

(ii) We analysed wells within the study area that the USGS has defined as either unconfined or confined. Most (>80%) wells at depths of 0-10 m and at depths exceeding 0 m are defined as tapping a confined aquifer.

Depth to confined conditions: 0-10 m (based on (ii) above)

References: Strom, E.W., Mallory, M.J. (1995). Hydrogeology and simulation of ground-water flow in the Eutaw-McShan Aquifer and in the Tuscaloosa aquifer system in northeastern Mississippi. US Geological Survey Water-Resources Investigations Report 94-4223, 89 pp. Accessed November 29, 2021 from https://pubs.usgs.gov/wri/1994/4223/report.pdf

The table below presents a series of published quotes (see quotation marks denoting text quoted from another publication, which is cited following the quotation marks with the full reference written in full below the table). The leftmost column lists a title of a hydrogeologic formation depicted in the cross section on the previous page. The rightmost column presents a quote from a hydrogeological study (see base of table for citation). The quote has been annotated with colored text to highlight how we categorized each layer (i.e., see categories in the center column in the table). Specifically: (i) blue text highlights portions of a quote that provide insights into the degree of consolidation of the formation, (ii) red text highlights portions of a quote that categorize the formation as an aquifer or an aquitard (i.e., higher versus lower permeability in the context of local hydrogeologic formations), and (iii) green text highlights portions of a quote that provide information about the lithology of the formation.

Supplementary Table 53. Hydrostratigraphy details for the Black Warrior River Aquifer System

Formation name	Category	Quote
Midway Group	Sedimentary rock **aquitard** (consolidated or semi-consolidated rock)	"South and west of the Ripley Cuesta is another belt of lowlands, the Flatwoods, underlain by the Paleocene Porters Creek **Clay** of the Midway Group." Mallory (1993) | "The Midway Group is composed predominantly of marine **clay and shale but includes subordinate sand and limestone beds**." Mallory (1993)
Ripley aquifer (part of Selma Group)	Clastic sedimentary **aquifer** (consolidated or semi-consolidated rock)	"An important **aquifer** in Tippah, eastern Benton, and northern Union Counties. The McNoiry sand member ranges up to 250 feet thick and is the unit which most of the wells utilize in the Ripley. Quality is good except for hardness." (Shows, 1970) | "The Ripley Formation in Alabama and Mississippi has a maximum thickness of about 500 ft. The formation typically consists of **clay, sandy clay, sand, and thin beds of sandstone**." Mallory (1993)
Demopolis Chalk and Mooreville Chalk (part of Selma Group; *"Coffee sand" formation may be an aquifer in areas)*	Sedimentary **aquitard** (consolidated or semi-consolidated rock)	"These formations in western Alabama and eastern Mississippi, in ascending order, are the **Mooreville Chalk, the Demopolis Chalk, the Ripley Formation, and the Prairie Bluff Chalk**." Cushing et al., (1964) "**Not an aquifer.**" (referring to Demopolis **Chalk** and Mooreville **chalk**) (Shows, 1970)
Tombigbee Sand Member	Clastic sedimentary **aquifer** (consolidated or semi-consolidated rock)	"A persistent **sand** at the top of the formation, known as the Tombigbee Sand Member (Hilgard, 1860, p. 61), is **massive, highly glauconitic, calcareous, and fossiliferous** in the upper part." Cushing et al., (1964) | "An important **aquifer** in most of Tishomingo and all of Alcorn and Prentiss Counties." In Table 3. (Shows, 1970).
Eutaw Formation	Clastic sedimentary **aquifer** (consolidated or semi-consolidated rock)	"The main body of the formation is composed of **gray clay interbedded** with fine glauconitic sand. Thin beds of fine to medium glauconitic sand are common and are fairly persistent near the base of the formation, which is normally marked by a thin bed of fine gravel. The sands are **commonly cross bedded or show distinct stratification**." Cushing et al., (1964) | "Thin beds of fine to medium glauconitic **sand** within the Eutaw and McShan Formations make up the bulk of the upper Black Warrior River regional aquifer, locally known as the **Eutaw-McShan aquifer**." Mallory (1993)
McShan Formation	Clastic sedimentary **aquifer** (consolidated or	"It consists of **laminated micaceous glauconitic gray clay, fine sand, and lenticular beds of fine to medium glauconitic sand**." Cushing et al., (1964) | "Thin beds of fine to medium

Formation name	Category	Quote
	semi-consolidated rock)	glauconitic **sand** within the Eutaw and McShan Formations make up the bulk of the upper Black Warrior River regional aquifer, locally know as the **Eutaw-McShan aquifer**." Mallory (1993)
Gordo Formation	Clastic sedimentary **aquifer** (consolidated or semi-consolidated rock)	"It is **composed of thick beds** of **sand** containing gravel in the lower part and multicolored clay and shale interbedded with sand in the upper part." Cushing et al., (1964) | "The Gordo Formation is an important **aquifer** in Alabama and Mississippi." Cushing et al., (1964)
Coker Formation	Clastic sedimentary **aquifer** (consolidated or semi-consolidated rock)	"In Alabama, the Coker has been subdivided into the Eoline Member and an upper unnamed member." Cushing et al., (1964)<br><br>"The Eoline Member (Monroe and others, 1946, p. 194-197) consists of **thin-bedded clay, sandy clay, shale, and sand, mostly of marine origin**; subordinate beds of **sand** occur throughout the unit." Cushing et al., (1964) | "**Permeable sand and gravel beds** in the Coker and Gordo Formations of the Tuscaloosa Group **make up the lower Black Warrior River aquifer**." Mallory (1993)
Massive sand	Clastic sedimentary **aquifer** (consolidated or semi-consolidated rock)	"The **massive sand** is "a series of **medium- to coarse grained sands** * * * " (according to McGlothlin, 1944, p. 40). Interbedded shale and clay occur in the thick beds of coarse sand, chert, and quartz gravel which compose the main body of the unit." Cushing et al., (1964) | "Although the massive sand is not generally used as a source of ground water, it is potentially one of the most **important aquifers** in the embayment." Mallory (1993)
Lower Cretaceous rocks	Clastic sedimentary **aquifer** (consolidated or semi-consolidated rock)	"Lower Cretaceous rocks do not crop out on the eastern side of the embayment. However, in Mississippi and Alabama they occur in the subsurface as **thick sands, clays, and shales**." Cushing et al., (1964) | "In places, some of the uppermost beds of the Lower Cretaceous series are lithologically very similar to the basal beds of the overlying Coker Formation and are included in this **aquifer**." Mallory (1993)
Paleozoic rocks	Sedimentary **aquitard** (consolidated or semi-consolidated rock)	"The lower Black Warrior River aquifer is the lowest aquifer in the Southeastern Coastal Plain aquifer system. In the northern part of the study area, the basal **confining unit** of the Southeastern Coastal Plain aquifer system consists of Paleozoic rocks. These **consolidated shales, sandstones, limestones, and dolomites** have **much smaller permeability** than the overlying Cretaceous sediments." Mallory (1993)

Cushing, E. M., Boswell, E. H., Hosman, R. L. (1964). General geology of the Mississippi Embayment. US Geological Survey Professional Paper 448-B, 32 pp. Accessed February 21, 2022 from https://pubs.usgs.gov/pp/0448b/report.pdf

Mallory, M. J. (1993). Hydrogeology of the Southeastern Coastal Plain Aquifer System in Parts of Eastern Mississippi and Western Alabama. Regional aquifer-system analysis. Southeastern coastal plain. US Geological Survey Professional Paper 1410-G, 66 pp. Accessed February 21, 2022 from https://pubs.usgs.gov/pp/1410g/report.pdf

Shows, T.N. (1970). Water resources of Mississippi. Mississippi Geological, Economic and Topographical Survey Bulletin 113, 182 pp. Accessed February 21, 2022 from https://www.mdeq.ms.gov/wp-content/uploads/2017/06/Bulletin-113.pdf

3.51 Castle Hayne Aquifer

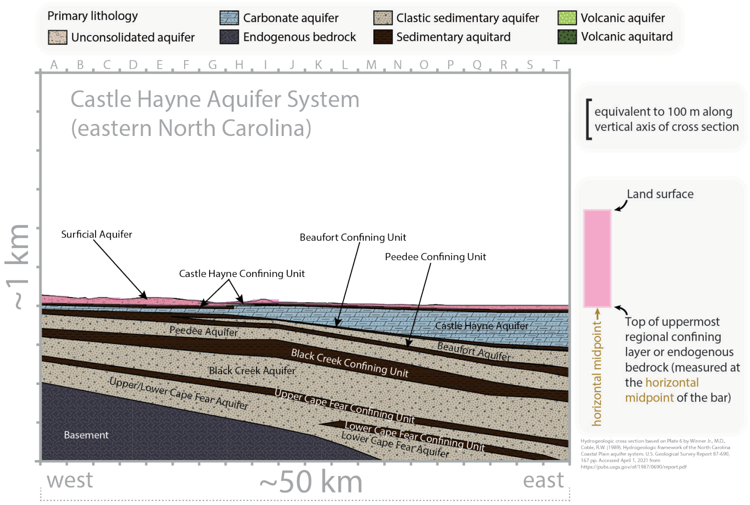

Supplementary Fig. 198. Hydrogeologic cross section. 20 equally spaced transparent pink bars overlie the cross section; each shaded bar depicts the vertical offset from the land surface to the top of the uppermost confining unit or endogenous bedrock.

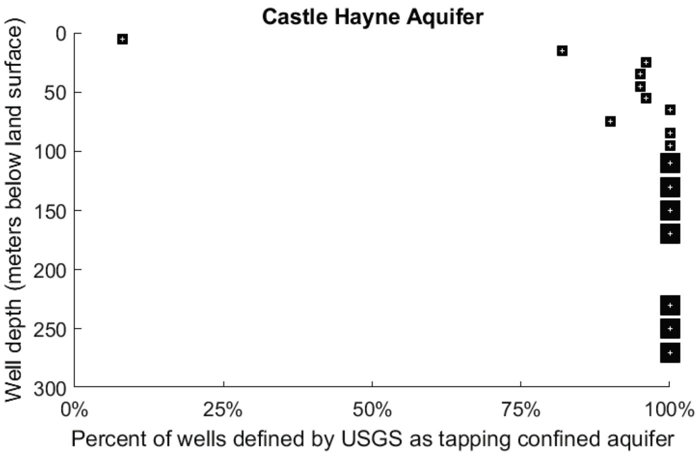

Supplementary Fig. 199. Vertical variations in the prevalence of wells that have been defined as tapping an unconfined or a confined aquifer by the USGS. The smaller squares represent 10 m depth intervals from the land surface to 100 m; the larger squares represent 20 m intervals from 100 m to 300 m below the land surface.

The Castle Hayne Aquifer System is located in the eastern portion of North Carolina.

(i) A hydrogeologic cross section presented in Plate 6 by Winner Jr. and Coble (1989) depicts shallow low-permeability units including the Castle Hayne Confining Unit, Beaufort Confining Unit, and Peedee Confining Unit.

(ii) We analysed wells within the study area that the USGS has defined as either unconfined or confined. Most (>80%) wells at depths of 10-20 m and at depths exceeding 10 m are defined as tapping a confined aquifer.

Depth to confined conditions: 10-20 m (based on (ii) above)

Reference: Winner Jr., M.D., Coble, R.W. (1989). Hydrogeologic framework of the North Carolina Coastal Plain aquifer system. US Geological Survey Report 87-690, 167 pp. Accessed April 1, 2021 from https://pubs.usgs.gov/of/1987/0690/report.pdf

The table below presents a series of published quotes (see quotation marks denoting text quoted from another publication, which is cited following the quotation marks with the full reference written in full below the table). The leftmost column lists a title of a hydrogeologic formation depicted in the cross section on the previous page. The rightmost column presents a quote from a hydrogeological study (see base of table for citation). The quote has been annotated with colored text to highlight how we categorized each layer (i.e., see categories in the center column in the table). Specifically: (i) blue text highlights portions of a quote that provide insights into the degree of consolidation of the formation, (ii) red text highlights portions of a quote that categorize the formation as an aquifer or an aquitard (i.e., higher versus lower permeability in the context of local hydrogeologic formations), and (iii) green text highlights portions of a quote that provide information about the lithology of the formation.

Supplementary Table 54. Hydrostratigraphy details for the Castle Hayne Aquifer

Formation name	Category	Quote
Surficial	Unconsolidated aquifer	"**The surficial aquifer** (A10) overlies all of the North Carolina Coastal Plain (fig. 1) and consists of **fine sand, silt, clay, shell, and peat beds**. **Scattered deposits of coarser-grained sediments in the unit occur in relict beach ridges or in alluvium**." (Giese et al., 1997)
Yorktown Confining unit	Sedimentary **aquitard** (consolidated or semi-consolidated rock)	"The **Yorktown confining unit** (CU9) overlying the Yorktown aquifer is comprised of the **youngest clay beds** of the Yorktown Formation in most places, but locally may **include clay beds** of Pleistocene or Holocene age. Its **thickness averages about 25 ft,** ranging from less than 10 up to 50 ft thick. It is composed largely **of clay and sandy clay** that locally includes beds of fine sand or shell." (Giese et al., 1997)
Yorktown	Clastic sedimentary **aquifer** (consolidated or semi-consolidated rock)	"**The Yorktown aquifer** largely consists of **fine sand, silty and clayey sand, sand with shells and shell beds, some limestone, and** some **coarse sand beds**.". (Giese et al., 1997).
Pungo River Confining unit	Sedimentary **aquitard** (consolidated or semi-consolidated rock)	"The **Pungo River confining unit (CU8)** is formed **by the upper clay beds of the Pungo River Formation and contiguous clays of the lowermost Yorktown Formation**. The **confining unit** ranges in thickness from less than 10 ft near the western margin to about 150 ft beneath Currituck County, with an average thickness of nearly 55 ft.". (Giese et al., 1997)
Pungo River	Clastic sedimentary **aquifer** (consolidated or semi-consolidated rock)	"The **Pungo River aquifer** is composed of **fine to medium marine sands** having considerable phosphate content.". (Giese et al., 1997). "The Pungo River aquifer (A8) is thinnest near its western and northern limits, **where its thickness averages about 15 ft**. The aquifer dips eastward and **thickens to more than 200 ft in the vicinity of the** Outer Banks, **where the top is deeper than 700 ft below sea level**." (Giese et al., 1997)
Castle Hayne Confining unit	Sedimentary **aquitard** (consolidated or semi-consolidated rock)	"The thickness of the **Castle Hayne confining unit (CU7) averages only about 10 ft; it exceeds 25 ft only** in Gates County along the Virginia border, in eastern Pamlico and Carteret Counties, and in two small areas along the western limit of the Castle Hayne aquifer (A7). **The confining unit** is composed of **beds of clay, sandy clay, and clay with sandy streaks that are part of the Purgo River Formation, the Yorktown Formation, or younger clays**." (Giese et al., 1997)

Formation name	Category	Quote
Castle Hayne	Carbonate aquifer	"The **Castle Hayne aquifer** (A7) consists of **limestone, sand, and minor amounts of clay deposited under marine conditions**. **Limestone may occur as shell limestone, dolomitic limestone, and sandy limestone ranging from loosely consolidated to hard and recrystallized**.". (Giese et al., 1997).
Beaufort Confining unit	Sedimentary **aquitard** (consolidated or semi-consolidated rock)	"**The Beaufort confining unit (CU6**) consists of the uppermost sediments of the Beaufort Formation and possibly **some younger clay, silt, and sandy clay**. Over most of the area, the confining unit shows a **gradation** from **sandy clay to clay**, but contains **distinct clay beds interlayered** with **fine sand or silt**.". (Giese et al., 1997)
Beaufort aquifer	Clastic sedimentary **aquifer** (consolidated or semi-consolidated rock)	"The **Beaufort aquifer** (A6) consists of **fine to medium glauconitic sands, clayey sands, and clay beds of marine origin**. **Shell and limestone beds** are present but are less than 6 ft thick.". (Giese et al., 1997).
Peedee Confining unit	Sedimentary **aquitard** (consolidated or semi-consolidated rock)	"The **Peedee confining unit (CU5)** is composed of **clay, silty clay, and sandy clay**. Winner and Coble (1989) **did not identify the confining unit** with a particular geologic unit, but **the unit is composed primarily of sediments at the Cenozoic-Mesozoic boundary**." (Giese et al., 1997)
Peedee aquifer	Clastic sedimentary **aquifer** (consolidated or semi-consolidated rock)	"The **Peedee aquifer (A5)** consists of **fine to medium sands interbedded with clays and silts**. **Thin beds of consolidated calcareous sandstone and impure limestone are interlayered among the sands in some places**, particularly in the southeastern North Carolina Coastal Plain area." (Giese et al., 1997).
Black Creek Confining unit	Sedimentary **aquitard** (consolidated or semi-consolidated rock)	"The **Black Creek confining unit** (CU4) is primarily composed of the uppermost **beds** of the **Black Creek Formation and consists of clay, silty clay, and sandy clay**.". (Giese et al., 1997).
Black Creek aquifer	Clastic sedimentary **aquifer** (consolidated or semi-consolidated rock)	"**The Black Creek aquifer** (A4) **contains Upper Cretaceous sediments** of both the Black Creek and underlying Middendorf Formations (Winner and Coble, 1989, 1996). **The Black Creek Formation consists mainly of thinly laminated gray to black clay, interbedded with gray to tan sands**. **Outcrops also exhibit sand- or clay-dominated lenses**." (Giese et al., 1997)
Upper Cape Fear Creek Confining unit	Sedimentary **aquitard** (consolidated or semi-consolidated rock)	"**the upper Cape Fear confining unit (CU3)** consists of nearly **continuous clay, silty clay, and sandy clay beds** belonging either to the Middendorf Formation in the Sand Hills area or to the Black Creek Formation. **The thickness of the confining unit averages about 48 ft (Winner and Coble, 1989) but may exceed 100 ft**" (Giese et al., 1997)
Upper Cape Fear aquifer	Clastic sedimentary **aquifer** (consolidated or semi-consolidated rock)	"The **upper Cape Fear aquifer** (A3) varies in thick- ness from about 10 ft along its western edge to nearly 500 ft in central Tyrrell County. The **average thickness** of the aquifer is slightly more than 100 ft.". (Giese et al., 1997). "The **Cape Fear aquifer consists predominantly of sand, silt, and gravel separated by relatively thick silt and clay layers**." (Aucott, 1996).

Formation name	Category	Quote
Lower Cape Fear Confining unit	Sedimentary **aquitard** (consolidated or semi-consolidated rock)	“The **lower Cape Fear confining unit (CU2)** is composed **of clay and sandy-clay beds that belong largely to the Cape Fear Formation**. **The average thickness of the confining unit is about 50 ft**.” (Giese et al., 1997)
Lower Cape Fear aquifer	Clastic sedimentary **aquifer** (consolidated or semi-consolidated rock)	“The **lower Cape Fear aquifer** (A2) strikes northeast and dips southwest at a rate of 15 to 35 ft/mi. Its extent is shown in figure 20. Its **thickness ranges from a few feet along its western margin to more than 400 ft** in the north- eastern North Carolina Coastal Plain.”. (Giese et al., 1997). “The **Cape Fear aquifer consists predominantly of sand, silt, and gravel separated by relatively thick silt and clay layers**.” (Aucott, 1996).
Lower Cretaceous aquifer	Clastic sedimentary **aquifer** (consolidated or semi-consolidated rock)	“Various investigators have established that the updip beds of the **Lower Cretaceous aquifer** are largely **nonmarine in origin**, but the incidence of **beds of marine origin increases** downdip toward the coast. **The non- marine beds are shales, sands, and gravel. Marine beds are chiefly limestones that may be sandy or dolomitic**.”. (Giese et al., 1997).
Crystalline Basement rock	Endogenous bedrock	“The Lower Cretaceous aquifer and confining unit are overlain everywhere by the lower Cape Fear aquifer (A2) and are underlain everywhere by **crystalline basement rocks**”. (Giese et al., 1997). “The unconsolidated Coastal Plain aquifer system is underlain by **crystalline basement** rocks of **low permeability**.” (Giese et al., 1991).

Giese, G.L., Eimers, J.L., Coble, R.W. (1997). Simulation of ground water flow in the Coastal Plain aquifer system of North Carolina. US Geological Survey Professional Paper 1404-M. 142 p. accessed on 3/29/2022 via https://pubs.usgs.gov/pp/1404m/report.pdf

Aucott, W.R. (1996). Hydrology of the Southeastern Coastal Plain aquifer system in South Carolina and parts of Georgia and North Carolina (No. 1410-E). US Geological Survey. https://pubs.er.usgs.gov/publication/pp1410E

Giese, G.L., Eimers, J.L., Coble, R.W. (1991). Simulation of ground-water flow in the Coastal Plain aquifer system of North Carolina (Vol. 1404). US Government Printing Office. Accessed on March 29, 2022 via https://pubs.er.usgs.gov/publication/ofr90372

3.52 Coachella Valley

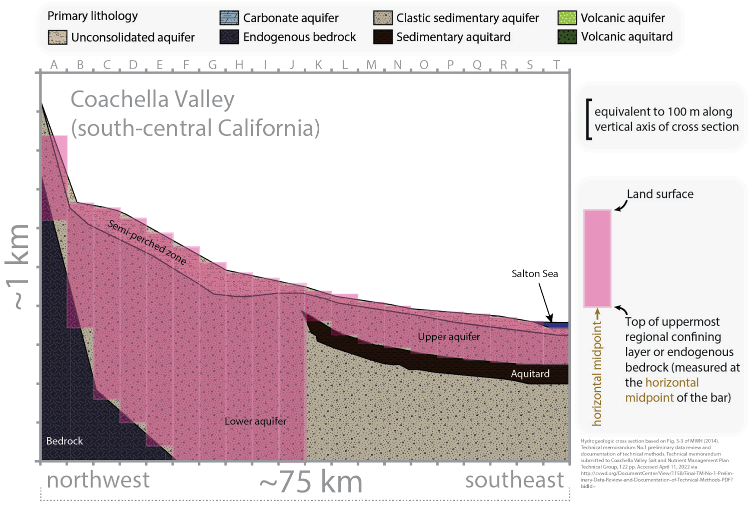

Supplementary Fig. 200. Hydrogeologic cross section. 20 equally spaced transparent pink bars overlie the cross section; each shaded bar depicts the vertical offset from the land surface to the top of the uppermost confining unit or endogenous bedrock.

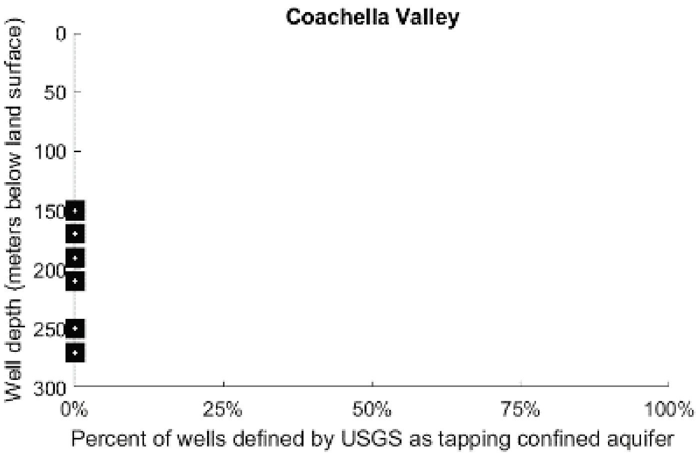

Supplementary Fig. 201. Vertical variations in the prevalence of wells that have been defined as tapping an unconfined or a confined aquifer by the USGS. The smaller squares represent 10 m depth intervals from the land surface to 100 m; the larger squares represent 20 m intervals from 100 m to 300 m below the land surface.

Coachella Valley is located in southcentral California.

(i) A hydrogeologic cross section presented in Fig. 3-3 by MWH (2014) suggests that a relatively continuous aquitard exists in the southeastern portion of the Coachella Valley, but that this unit is absent in the northwestern portion of the area.

(ii) We analysed wells within the study area that the USGS has defined as either unconfined or confined. Nearly all (11 of 12) wells in the Coachella Valley are defined as unconfined, including the deepest wells in the dataset (depths of 323 m and 341 m).

Depth to confined conditions: >341 m (based on (ii) above)

Reference: MWH (2014). Technical memorandum No.1 preliminary data review and documentation of technical methods. Technical memorandum submitted to Coachella Valley Salt and Nutrient Management Plan Technical Group, 122 pp. Accessed April 11, 2022 via http://cvwd.org/DocumentCenter/View/1158/Final-TM-No-1-Preliminary-Data-Review-and-Documentation-of-Technical-Methods-PDF?bidId=

The table below presents a series of published quotes (see quotation marks denoting text quoted from another publication, which is cited following the quotation marks with the full reference written in full below the table). The leftmost column lists a title of a hydrogeologic formation depicted in the cross section on the previous page. The rightmost column presents a quote from a hydrogeological study (see base of table for citation). The quote has been annotated with colored text to highlight how we categorized each layer (i.e., see categories in the center column in the table). Specifically: (i) blue text highlights portions of a quote that provide insights into the degree of consolidation of the formation, (ii) red text highlights portions of a quote that categorize the formation as an aquifer or an aquitard (i.e., higher versus lower permeability in the context of local hydrogeologic formations), and (iii) green text highlights portions of a quote that provide information about the lithology of the formation.

Supplementary Table 55. Hydrostratigraphy details for the Coachella Valley

Formation name	Category	Quote
Semi-perched zone	Unconsolidated aquifer	"The **semi-perched aquifer** is characterized by **fine-grained Holocene and Recent lake deposits and alluvium** that form an effective barrier to the deep percolation of surface runoff and applied water within the central portion of the East Valley where present." MWH (2014). "The **semi-perched aquifer** consists of **interbedded layers of fine sand and clay** and is separated from the underlying upper aquifer by a laterally discontinuous clay zone (DWR, 1964)." MWH (2014).
Upper aquifer	Unconsolidated aquifer	"Based on DWR (1964), **the upper aquifer**, which is formed of **Upper Pleistocene alluvium**, underlies the semi-perched aquifer. The upper aquifer typically **consists of coarse sand and gravel with discontinuous clay lenses in the West Valley and the northern part of the East Valley. Finer sand and sandy clay dominate in the southern part of the East Valley**. The upper aquifer is believed to **be unconfined or semi-confined** in most of the West Valley, and is confined in most of the East Valley by the semi-perched aquifer and a discontinuous clay layer (referred to as the aquitard)." MWH (2014).
Aquitard	Sedimentary **aquitard** (consolidated or semi-consolidated rock)	"The **aquitard** typically consists of **clay and sandy clay with discontinuous sand lenses** having **low permeability**." MWH (2014).
Lower aquifer	Unconsolidated aquifer	**The lower aquifer zone**, composed in part of **the Ocotillo conglomerate**, consists of **silty sands and gravels with interbeds of silt and clay**. It is the most **important source of groundwater** in the Whitewater River Subbasin." MWH (2014).
Bedrock	Endogenous bedrock	"DWR (1964) inferred the depth to **bedrock** was in excess of 12,000 feet below ground surface based on gravity survey data." MWH (2014). "**basement bedrock**" MWH (2014).

MWH (2014). Technical memorandum No.1 preliminary data review and documentation of technical methods. Technical memorandum submitted to Coachella Valley Salt and Nutrient Management Plan Technical Group, 122 pp. Accessed June 2, 2022 via http://cvwd.org/DocumentCenter/View/1158/Final-TM-No-1-Preliminary-Data-Review-and-Documentation-of-Technical-Methods-PDF?bidId=

3.53 Cuyama Valley

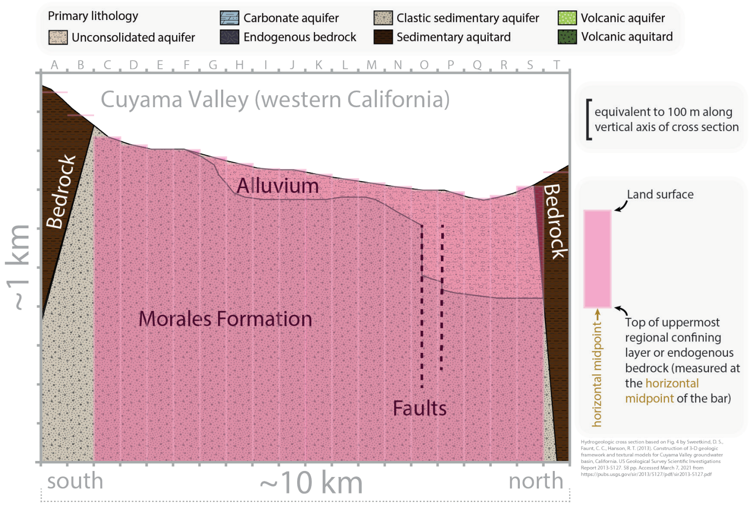

Supplementary Fig. 202. Hydrogeologic cross section. 20 equally spaced transparent pink bars overlie the cross section; each shaded bar depicts the vertical offset from the land surface to the top of the uppermost confining unit or endogenous bedrock.

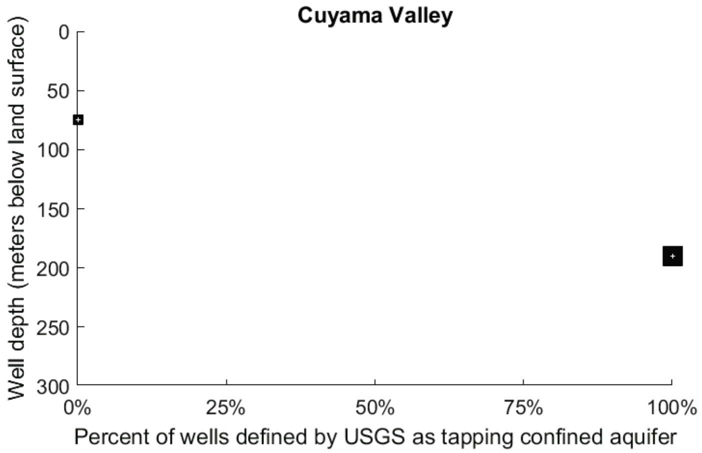

Supplementary Fig. 203. Vertical variations in the prevalence of wells that have been defined as tapping an unconfined or a confined aquifer by the USGS. The smaller squares represent 10 m depth intervals from the land surface to 100 m; the larger squares represent 20 m intervals from 100 m to 300 m below the land surface.

The Cuyama Valley is located in western California.

(i) A hydrogeologic cross section presented in Fig. 2 by Sweetkind et al. (2013) does not depict a clear confining unit (see cross section to the left). The median depth to a confined unit or endogenous bedrock is >722 m.

(ii) We analysed wells within the study area that the USGS has defined as either unconfined or confined. The available USGS well data (n=2 wells with depths of 71 m and 183 m) are insufficient to evaluate the depths at which the valley transitions from unconfined to confined conditions.

(iii) In their 2021 Groundwater Sustainability Plan, Woodard and Curran report (direct quote): *"The aquifer is considered to be continuous and unconfined with the exception of locally perched aquifers resulting from clays in the formations."*

Depth to confined conditions: >722 m based on (i) and (iii)

References: Woodard and Curran (2021). Cuyama Basin Groundwater Sustainability Plan, Chapter 2, Basin Settings. 168 pp. Accessed May 16, 2022 via https://cuyamabasin.org/assets/pdf/public-final-gsp/Cuyama-Final-GSP-Chapter-2.pdf

Sweetkind, D.S., Faunt, C.C., Hanson, R.T. (2013). Construction of 3-D geologic framework and textural models for Cuyama Valley groundwater basin, California. US Geological Survey Scientific Investigations Report 2013-5127. 58 pp. Accessed March 7, 2021 from https://pubs.usgs.gov/sir/2013/5127/pdf/sir2013-5127.pdf

The table below presents a series of published quotes (see quotation marks denoting text quoted from another publication, which is cited following the quotation marks with the full reference written in full below the table). The leftmost column lists a title of a hydrogeologic formation depicted in the cross section on the previous page. The rightmost column presents a quote from a hydrogeological study (see base of table for citation). The quote has been annotated with colored text to highlight how we categorized each layer (i.e., see categories in the center column in the table). Specifically: (i) blue text highlights portions of a quote that provide insights into the degree of consolidation of the formation, (ii) red text highlights portions of a quote that categorize the formation as an aquifer or an aquitard (i.e., higher versus lower permeability in the context of local hydrogeologic formations), and (iii) green text highlights portions of a quote that provide information about the lithology of the formation.

Supplementary Table 56. Hydrostratigraphy details for the Cuyama Valley

**Formation name**	**Category**	**Quote**
Alluvial aquifers	Unconsolidated aquifer	"Geologic maps of the Cuyama Valley (Vedder and Repenning, 1975; Kellogg and others, 2008) show two units of Holocene and Pleistocene-aged **alluvial deposits**, termed younger and older alluvium, underlying the three monitoring well sites." Everett et al., 2013. "The **main water-bearing deposits** in the study area are the saturated portions of the younger and older **alluvium** and the Morales Formation." (Everett et al. 2013)
Morales formation aquifer	Clastic sedimentary **aquifer** (consolidated or semi-consolidated rock)	"The Morales Formation (QTm, fig. 2) is a Pliocene Pleistocene fluvial deposit that is up to 5,000 feet thick and consists of massive- to thick-bedded, **partly consolidated** deposits of clay, silt, sand, and gravel (Hill and others, 1958; Ellis and others, 1993).". **Everett** et al., 2013. "The **main water-bearing deposits** in the study area are the saturated portions of the younger and older **alluvium** and the Morales Formation." (Everett et al. 2013)
Bedrock	Sedimentary rock **aquitard** (consolidated or semi-consolidated rock) OR Endogenous bedrock *(*for display purposes, we show a sedimentary rock aquitard in Fig. 1)*	"**non-water-bearing rocks**—**the crystalline granitic rocks** and **all consolidated sedimentary rocks** older than the Morales Formation". **Everett** et al., 2013. "All rocks that are older than the Morales Formation were considered by previous investigators to be **non-water-bearing**" (Everett et al., 2013)

Everett, R. R., Gibbs, D. R., Hanson, R. T., Sweetkind, D. S., Brandt, J. T., Falk, S. E., Harich, C. R. (2013). Geology, water-quality, hydrology, and geomechanics of the Cuyama Valley groundwater basin, California, 2008-12. US US Geological Survey Scientific Investigations Report 2013–5108, 76 pp. Accessed February 21, 2022 from https://pubs.usgs.gov/sir/2013/5108/pdf/sir2013-5108.pdf

3.54 Denver Basin

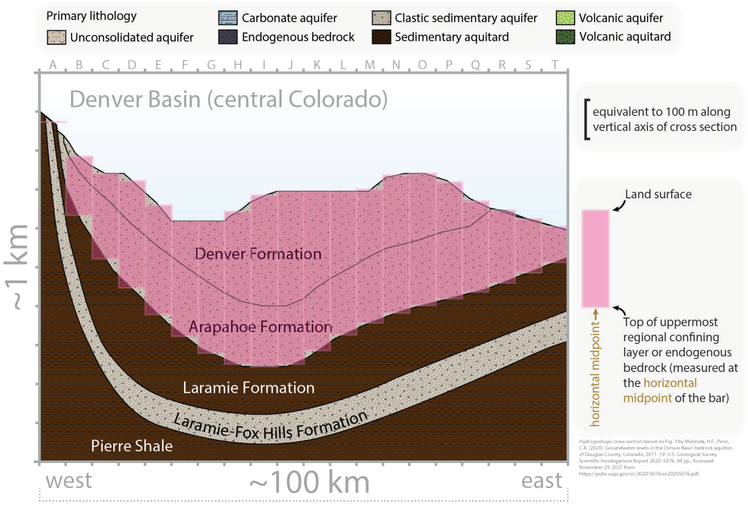

Supplementary Fig. 204. Hydrogeologic cross section. 20 equally spaced transparent pink bars overlie the cross section; each shaded bar depicts the vertical offset from the land surface to the top of the uppermost confining unit or endogenous bedrock.

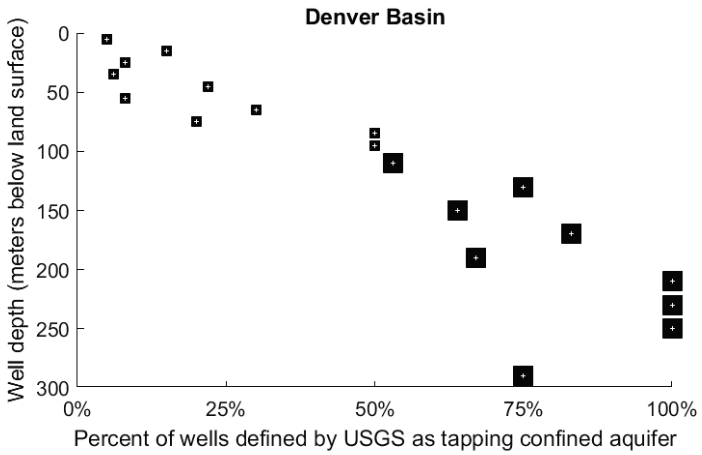

Supplementary Fig. 205. Vertical variations in the prevalence of wells that have been defined as tapping an unconfined or a confined aquifer by the USGS. The smaller squares represent 10 m depth intervals from the land surface to 100 m; the larger squares represent 20 m intervals from 100 m to 300 m below the land surface.

The Denver Basin is located in the central United States.

(i) A hydrogeologic cross section presented in Fig. 3 by Malenda and Penn (2020) suggests the top of the uppermost confining unit (the Laramie Formation) is typically 297 meters below the land surface (i.e. median of pink bars in cross section to left).

(ii) We analysed wells within the study area that the USGS has defined as either unconfined or confined. Most (>80%) wells at depths of 160-180 m and at depths exceeding 160 m are defined as tapping a confined aquifer.

(iii) Malenda and Penn (2020) highlight that confined conditions can exist in the Denver Formation, which overlies the Laramie Formation; they state that “The three Denver aquifer wells represent confined aquifer conditions”. Therefore, we do not rely solely on the depth to the Laramie Formation to estimate the depth to confined conditions, instead drawing information from the wells where the USGS has defined whether the well taps unconfined versus confined conditions.

Depth to confined conditions: 160-180 m (based on (ii) above)

Reference: Malenda, H.F., Penn, C.A. (2020). Groundwater levels in the Denver Basin bedrock aquifers of Douglas County, Colorado, 2011–19. U.S. Geological Survey Scientific Investigations Report 2020–5076, 44 pp., Accessed November 29, 2021 from https://pubs.usgs.gov/sir/2020/5076/sir20205076.pdf

The table below presents a series of published quotes (see quotation marks denoting text quoted from another publication, which is cited following the quotation marks with the full reference written in full below the table). The leftmost column lists a title of a hydrogeologic formation depicted in the cross section on the previous page. The rightmost column presents a quote from a hydrogeological study (see base of table for citation). The quote has been annotated with colored text to highlight how we categorized each layer (i.e., see categories in the center column in the table). Specifically: (i) blue text highlights portions of a quote that provide insights into the degree of consolidation of the formation, (ii) red text highlights portions of a quote that categorize the formation as an aquifer or an aquitard (i.e., higher versus lower permeability in the context of local hydrogeologic formations), and (iii) green text highlights portions of a quote that provide information about the lithology of the formation.

Supplementary Table 57. Hydrostratigraphy details for the Denver Basin

**Formation name**	**Category**	**Quote**
Denver Formation	Clastic sedimentary **aquifer** (consolidated or semi-consolidated rock)	"**Alluvial fan**, swamp, overbank deposits; andesitic fluvial **sandstone with volcanic ash deposits, coal, lignite, mudstone/claystone**; Fe-rich sediments; sediments source of Se and U to **groundwater**." (Musgrove et al., 2014) "**Confined to unconfined aquifer**" (Musgrove et al., 2014). "Confined in central part. Contains a **water table only near out-crops. Moderately permeable. May yield as much as 200 gallons per minute**." (Robson & Banta, 1995)
Arapahoe Formation	Clastic sedimentary **aquifer** (consolidated or semi-consolidated rock)	"**Fluvial environment, alluvial fan deposits near mountain front; conglomerates**, **sandstone, siltstone, shale; pebbles and cobbles with granite, chert, metamorphic rocks, and quartze; shale** more prevalent in northern part of basin." (Musgrove et al., 2014). "**Productive confined aquifer**"- (Musgrove et al., 2014).
Laramie Formation	Sedimentary **aquitard** (consolidated or semi-consolidated rock)	"**Upper part shale, silty shale, silt stone,** and **interbedded** fine **sandstone**." (Robson & Banta, 1995). "**Shale is impermeable**". (Robson & Banta, 1995). See figure 83 from Ref (Robson & Banta, 1995)
Laramie-Fox Hill Formation	Clastic sedimentary **aquifer** (consolidated or semi-consolidated rock)	"Laramie Formation: **swamps, deltas, overbank deposits; claystone, coal, fluvial channel sandstone**; contains coal and lignite beds. Fox Hills Sandstone: **marine beach and delta-front environment**; **sandstone, thin siltstone and claystone beds; contains marine fossils**." (Musgrove et al., 2014) "**Productive confined to unconfined aquifer**" – (Musgrove et al., 2014)
Pierre shale	Sedimentary rock **aquitard** (consolidated or semi-consolidated rock)	"**A thick unit** of **low-permeability** Cretaceous age Pierre **Shale** underlies the Laramie-Fox Hills aquifer and forms the base of the aquifer system" – (Musgrove et al., 2014)

Musgrove, M., Beck, J. A., Paschke, S. S., Bauch, N. J., Mashburn, S. L. (2014). Quality of Groundwater in the Denver Basin Aquifer System, Colorado, 2003-5. US Geological Survey Scientific Investigations Report 2014-5051, 123 pp. Accessed February 21, 2022 from https://pubs.usgs.gov/sir/2014/5051/pdf/sir2014-5051.pdf

Robson, S. G., Banta, E. R. (1995). Ground water atlas of the United States: Segment 2, Arizona, Colorado, New Mexico, Utah. US Geological Survey Hydrologic Investigations Atlas 730-C, Segment 2, 34 pp. Accessed February 21, 2022 from https://pubs.usgs.gov/ha/730c/report.pdf

3.55 Eastern Dakota Aquifer

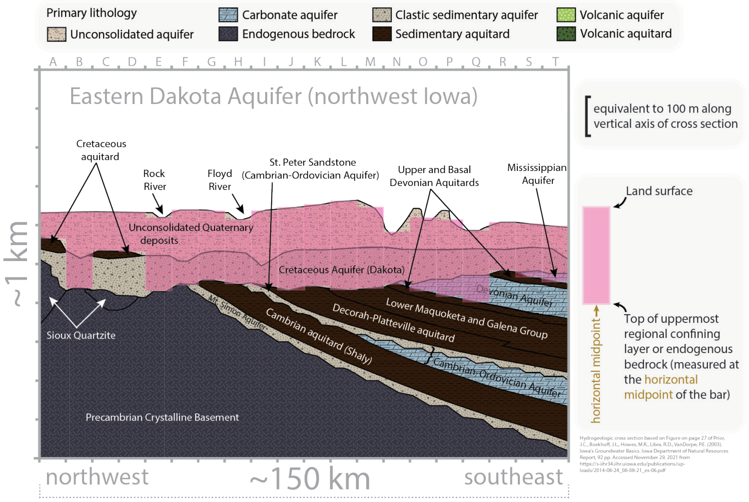

Supplementary Fig. 206. Hydrogeologic cross section. 20 equally spaced transparent pink bars overlie the cross section; each shaded bar depicts the vertical offset from the land surface to the top of the uppermost confining unit or endogenous bedrock.

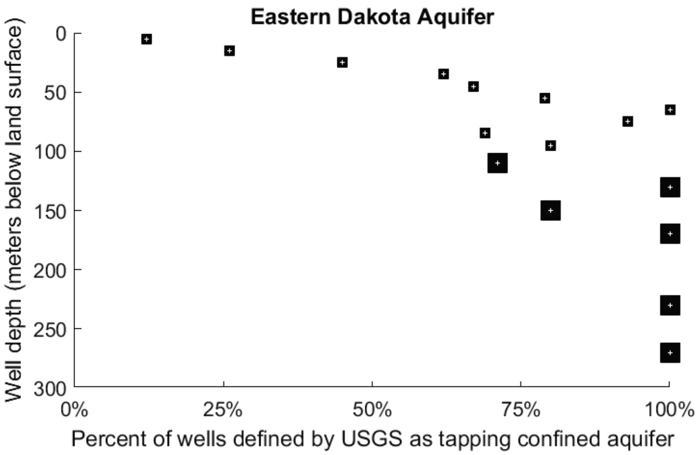

Supplementary Fig. 207. Vertical variations in the prevalence of wells that have been defined as tapping an unconfined or a confined aquifer by the USGS. The smaller squares represent 10 m depth intervals from the land surface to 100 m; the larger squares represent 20 m intervals from 100 m to 300 m below the land surface.

The Eastern Dakota Aquifer is located in northwest Iowa.

(i) A hydrogeologic cross section presented in the figure on page 27 by Prior et al. (2003) depicts relatively thick unconsolidated deposits overlying the Dakota Aquifer, which itself overlies dipping sedimentary sequences including several aquitards (e.g., the Upper and Basal Devonian Aquitards).

(ii) We analysed wells within the study area that the USGS has defined as either unconfined or confined. Most (>80%) wells at depths of 60-70 m and at depths exceeding 60 m are defined as tapping a confined aquifer.

Depth to confined conditions: 60-70 m (based on (ii) above)

Reference: Prior, J.C., Boekhoff, J.L., Howes, M.R., Libra, R.D., VanDorpe, P.E. (2003). Iowa's Groundwater Basics. Iowa Department of Natural Resources Report, 92 pp. Accessed November 29, 2021 from https://s-iihr34.iihr.uiowa.edu/publications/uploads/2014-08-24_08-08-21_es-06.pdf

The table below presents a series of published quotes (see quotation marks denoting text quoted from another publication, which is cited following the quotation marks with the full reference written in full below the table). The leftmost column lists a title of a hydrogeologic formation depicted in the cross section on the previous page. The rightmost column presents a quote from a hydrogeological study (see base of table for citation). The quote has been annotated with colored text to highlight how we categorized each layer (i.e., see categories in the center column in the table). Specifically: (i) blue text highlights portions of a quote that provide insights into the degree of consolidation of the formation, (ii) red text highlights portions of a quote that categorize the formation as an aquifer or an aquitard (i.e., higher versus lower permeability in the context of local hydrogeologic formations), and (iii) green text highlights portions of a quote that provide information about the lithology of the formation.

Supplementary Table 58. Hydrostratigraphy details for the Eastern Dakota Aquifer

**Formation name**	**Category**	**Quote**
Unconsolidated Quaternary deposits	Unconsolidated aquifer	From Prior et al. (2003) page 14: Hydrogeologic units: **"Alluvium", "Glacial drift", and "Buried Valley".** Dominant geologic material: **"Sand, gravel, silt, clay", "Pebbly clay, silt, sand & gravel", and "Sand & gravel"**. Hydrologic condition: **"Local to regional aquifers", "local sand & gravel aquifers", and "local to regional aquifers"** respectively.
Cretaceous aquitard	Sedimentary **aquitard** (consolidated or semi-consolidated rock)	From Prior et al. (2003) page 14: Hydrogeologic units: "**Cretaceous confining units**" Dominant geologic material: "**Shale, limestone**". Hydrologic condition: "**Confining beds; aquitard**".
Cretaceous Aquifer (Dakota)	Clastic sedimentary rock **aquifer** (consolidated or semi-consolidated rock)	From Prior et al. (2003) page 14: Hydrogeologic units: "**Dakota aquifer**" Dominant geologic material: "**Sandstone**". Dominant geologic material: "**Sandstone**". Hydrologic condition: "**Regional aquifer**". "**The two uppermost hydrogeologic units, the drift and the Cretaceous aquifers, generally are unconfined and are the top layer of the regional flow model** (Mandie and Kontis, in press)." (Young, 1992).
Mississippian Aquifer	Carbonate aquifer	From Prior et al. (2003) page 14: Hydrogeologic units: "**Mississippian aquifer**". Dominant geologic material: **"limestone, sandstone, shale", "dolomite, shale, limestone, chert"**, and "**dolomite, limestone, chert**". hydrologic condition: "local aquitard" & "regional aquifer". "The **Mississippian aquifer** is one of the **most dependable sources of groundwater in north-central Iowa**. **Wells drilled into the Mississippian aquifer supply large volumes of water** to livestock, industries, and municipalities." (Gannon & McKay, 2013).
Upper Devonian Shale aquitard *(local aquifers exist)*	Sedimentary rock **aquitard** (consolidated or semi-consolidated rock)	From Prior et al. (2003) page 14: Hydrogeologic units: "**Devonian confining unit**" Dominant geologic material: "**shale, siltstone, dolomite**", & "**shale, dolomite, limestone**". Hydrologic condition: "**confining beds; aquitard", & "confining beds; aquitard, local aquifers**".
Devonian aquifer	Carbonate aquifer	From Prior et al. (2003) page 14: Hydrogeologic units: "**Devonian aquifer**" Dominant geologic material: "**dolomite, chert, limestone**". Hydrologic condition: "**regional aquifer**".
Basal Devonian Aquitard	Sedimentary rock **aquitard** (consolidated	From Prior et al. (2003) page 14: Hydrogeologic units: "Devonian aquifer" Dominant geologic material: "**limestone,**

Formation name	Category	Quote
(evaporite, shale)	or semi-consolidated rock)	**dolomite**, **shale**, **gypsum**". Hydrologic condition: "**local aquitard**".
Lower Maquoketa and Galena Group	Sedimentary rock **aquitard** (consolidated or semi-consolidated rock)	From Prior et al. (2003) page 14: Hydrogeologic units: "**Ordovician confining units**" Dominant geologic material: "**shale, dolomite, chert**", & "**dolomite, limestone, chert**". Hydrologic condition: "**confining beds, aquitard, local aquifer** in northeastern", & "**confining beds, aquitard, local aquifer** in northeastern" respectively.
Decorah-Platteville aquitard	Sedimentary rock **aquitard** (consolidated or semi-consolidated rock)	From Prior et al. (2003) page 14: Hydrogeologic units: "**Ordovician confining units**" Dominant geologic material: "**shale, limestone, sandstone**". Hydrologic condition: "**confining beds, aquitard**".
Cambrian-Ordovician Aquifer	St. Peter Sandstone & Jordan Sandstone are (Clastic sedimentary rock **aquifer** (consolidated or semi-consolidated rock)) and Prairie du Chen Group & St. Lawrence Formation are (Carbonate aquifer)	From Prior et al. (2003) page 14: Hydrogeologic units: "**Cambrian-Ordovician aquifer ("Jordan aquifer")**" Dominant geologic material: "**sandstone**", "**dolomite, sandstone, chert**", "**sandstone**", & "**dolomite**". Hydrologic condition: "**regional aquifer**", **regional aquifer**", **regional aquifer**", & **regional aquifer**" respectively.
Cambrian aquitard (Shaly)	Sedimentary rock **aquitard** (consolidated or semi-consolidated rock)	From Prior et al. (2003) page 14: Hydrogeologic units: "**Cambrian confining unit**" Dominant geologic material: "**shale, siltstone, sandstone**". Hydrologic condition: "**confining beds; aquitard**".
Mt. Simon Aquitard	Clastic sedimentary rock **aquifer** (consolidated or semi-consolidated rock)	From Prior et al. (2003) page 14: Hydrogeologic units: "**Dresbach aquifer**" Dominant geologic material: "**sandstone, shale, dolomite**". Hydrologic condition: "**regional aquifer**".
Precambrian Crystalline Basement	Endogenous bedrock	From Prior et al. (2003) page 14: Dominant geologic material: "**igneous & metamorphic rocks, sandstone, shale**". Hydrologic condition: "**unknown**". "Cambrian and Ordovician rocks form the dominant aquifer system in the study area. They are bounded below by **Precambrian basement rocks**, which have **very low permeability**" (Young, 1992).

Prior, J. C., Boekhoff, J. L., Howes, M. R., Libra, R. D., VanDorpe, P. E. (2003). Iowa's Groundwater Basics: A geological guide to the occurence, use, and vulnerability of Iowa's aquifers. Accessed June 11, 2022 via https://s-iihr34.iihr.uiowa.edu/publications/uploads/2014-08-24_08-08-21_es-06.pdf

Young, H. L. (1992). *Summary of ground-water hydrology of the Cambrian-Ordovician aquifer system in the northern Midwest, United States* (Vol. 1405). US Department of the Interior, US Geological Survey. Accessed June 11, 2022 via https://pubs.er.usgs.gov/publication/pp1405B

Gannon, J. M., McKay, R. M. (2013). *Groundwater Availability Modeling of the Mississippian Aquifer North-Central Iowa* (Vol. 8). University of Iowa. Accessed June 11, 2022 via https://iro.uiowa.edu/esploro/outputs/report/Groundwater-Availability-Modeling-of-the-Mississippian/9984109923302771

3.56 Eureka and Eel River and Mad River Plains

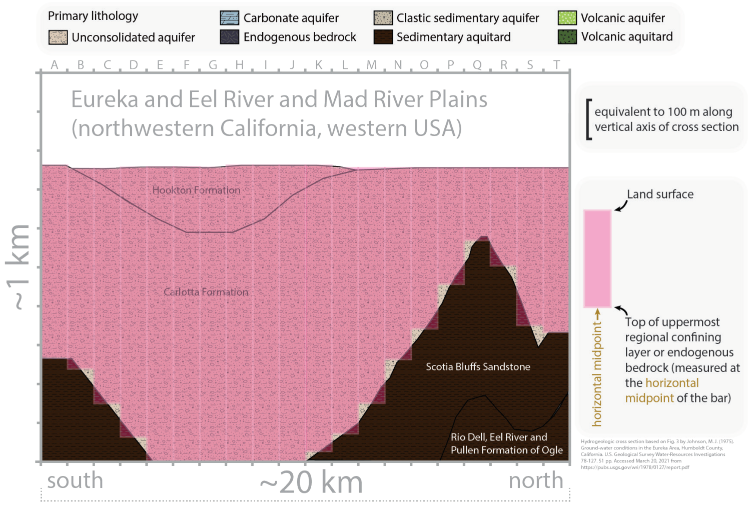

Supplementary Fig. 208. Hydrogeologic cross section. 20 equally spaced transparent pink bars overlie the cross section; each shaded bar depicts the vertical offset from the land surface to the top of the uppermost confining unit or endogenous bedrock.

The Eureka and Eel River and Mad River Plains are located in coastal northwest California.

(i) A hydrogeologic cross section presented in Fig. 3 by Johnson (1975) suggests that the aquifer system does not depict a clear confining unit within the aquifer system (see cross section to the left); however, fine-grained interbedded layers lead to confined conditions in the lower portion of the Hookton Formation and Carlotta Formation (see quotes from Johnson (1975) in “(iii)” below).

(ii) We analysed wells within the study area that the USGS has defined as either unconfined or confined. There are no USGS wells within the study area that have been defined as tapping an aquifer that is either unconfined or confined.

(iii) With regards to the presence of confined conditions, a local-scale study by Johnson (1975) states that (quote) “*“The two younger units, the Scotia Bluffs Sandstone and Carlotta Formation, consist dominantly of coarse-grained clastic sediments of marginal marine deposition and may be important aquifers. Water found in these two formations in the Eureka area is generally confined by interbeds of silt and clay of low permeability or by the over-lying, fine-grained sediment in the Hookton Formation".* and, with regards to the Hookton Formation), Johnson (1975) states (quote) *“The Hookton Formation supplies water to many domestic wells in the Dows Prairie-McKinleyville area and on the hills and terraces of the study area. At lower altitudes and under the river valleys, water in the lower parts of the Hookton is in part confined by the overlying material”.* Because the lower portion of the Hookton Formation is referred to as confined, we estimate the depth to confined conditions to be approximately half the maximum thickness of the Hookton Formation (maximum thickness is ~150 m; half the maximum thickness is ~75 m) as depicted on the cross section by Johnson (1975) (see figure in upper left of this page). We highlight that this approximation is uncertain.

Depth to confined conditions: 70-80 m (see (iii) above)

Reference: Johnson, M. J. (1975). Ground-water conditions in the Eureka Area, Humboldt County, California. U.S. Geological Survey Water-Resources Investigations 78-127. 51 pp. Accessed March 20, 2021 from https://pubs.usgs.gov/wri/1978/0127/report.pdf

The table below presents a series of published quotes (see quotation marks denoting text quoted from another publication, which is cited following the quotation marks with the full reference written in full below the table). The leftmost column lists a title of a hydrogeologic formation depicted in the cross section on the previous page. The rightmost column presents a quote from a hydrogeological study (see base of table for citation). The quote has been annotated with colored text to highlight how we categorized each layer (i.e., see categories in the center column in the table). Specifically: (i) blue text highlights portions of a quote that provide insights into the degree of consolidation of the formation, (ii) red text highlights portions of a quote that categorize the formation as an aquifer or an aquitard (i.e., higher versus lower permeability in the context of local hydrogeologic formations), and (iii) green text highlights portions of a quote that provide information about the lithology of the formation.

Supplementary Table 59. Hydrostratigraphy details for Eureka and Eel River and Mad River Plains

**Formation name**	**Category**	**Quote**
Hookton Formation	Unconsolidated aquifer *(In some area this aquifer is confined aquifer)*	"**Poorly consolidated**; **yields water to wells in small to moderate amounts** from **sand and gravel strata**. **Confined aquifers south of Arcata.**" (Johnson, 1978).
Carlotta Formation	Unconsolidated aquifer *(In some area this aquifer is confined aquifer)*	"**Poorly consolidated**; locally **yields water to wells in moderate to large amounts** from **confined sand and gravel**" (Johnson, 1978).
Scotia Bluffs Sandstone	Sedimentary rock **aquitard** (consolidated or semi-consolidated rock)	"**Massive, fine-grained sandstone with mudstone member in lower part and fluvial sand and gravel in upper part, predominantly of shallow marine origin.**" (Johnson, 1978). "**Semi-consolidated to poorly consolidated**. **Not tapped by water wells**, **potential yield unknown**." (Johnson, 1978).
Rio Dell, Eel River and Pullen Formation of Ogle	Sedimentary rock **aquitard** (consolidated or semi-consolidated rock)	"**Compact mudstone, claystone, siltstone, and some sandstone, predominantly of marine origin**." (Johnson, 1978). "**Semi-consolidated**; **not tapped by wells, probably poor aquifer**." (Johnson, 1978).

Johnson, M. J. (1978). Ground-water conditions in the Eureka area, Humboldt County, California, 1975 (Vol. 78). US Geological Survey, Water Resources Division. Accessed August 10, 2022 via https://pubs.usgs.gov/wri/1978/0127/report.pdf

3.57 Garber-Wellington Aquifer

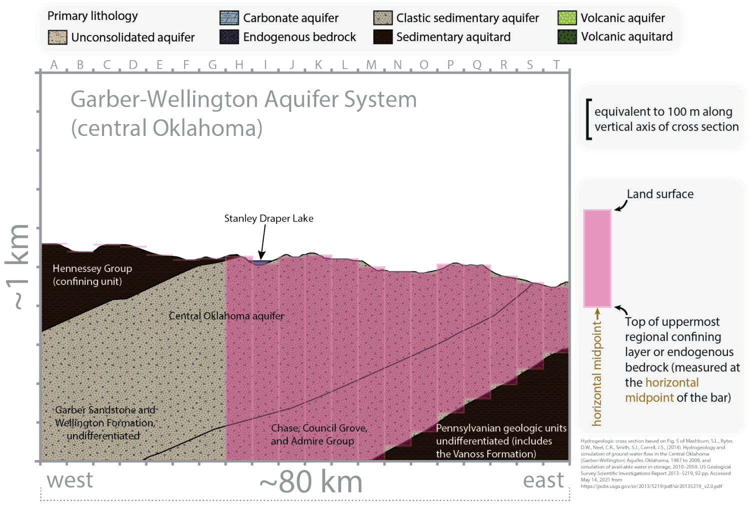

Supplementary Fig. 209. Hydrogeologic cross section. 20 equally spaced transparent pink bars overlie the cross section; each shaded bar depicts the vertical offset from the land surface to the top of the uppermost confining unit or endogenous bedrock.

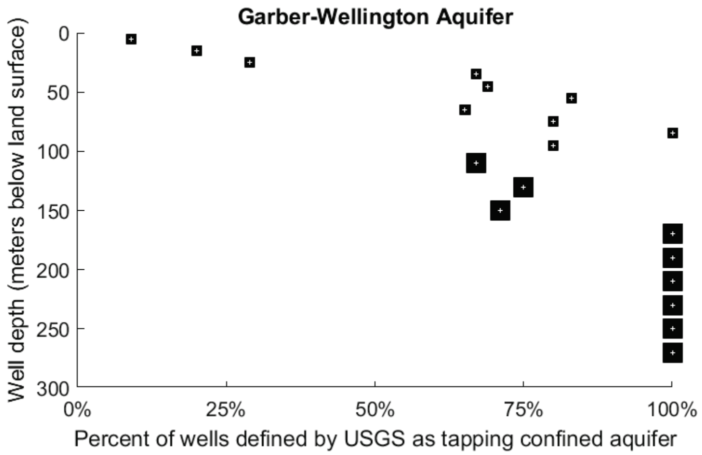

Supplementary Fig. 210. Vertical variations in the prevalence of wells that have been defined as tapping an unconfined or a confined aquifer by the USGS. The smaller squares represent 10 m depth intervals from the land surface to 100 m; the larger squares represent 20 m intervals from 100 m to 300 m below the land surface.

The Garber-Wellington aquifer system is located in central Oklahoma.

(i) A hydrogeologic cross section presented in Fig. 5 by Mashburn et al. (2014) does not depict a clear confining unit in the central portion of the study area (but see quote in (iii) below).

(ii) We analysed wells within the study area that the USGS has defined as either unconfined or confined. Most (>80%) wells at depths of 80-90 m and at depths exceeding 80 m are defined as tapping a confined aquifer.

(iii) Mashburn et al. (2014) state that (following text quoted directly): *"…even though the Central Oklahoma aquifer extends to land surface with a potentiometric surface below the top of the Central Oklahoma aquifer, the groundwater system acts as a confined system due to laterally extensive interbedded mudstone and large contrasts in vertical hydraulic conductivity."*

Depth to confined conditions: 80-90 m (see (ii) above)

Reference: Mashburn, S.L., Ryter, D.W., Neel, C.R., Smith, S.J., Correll, J.S., (2014). Hydrogeology and simulation of ground-water flow in the Central Oklahoma (Garber-Wellington) Aquifer, Oklahoma, 1987 to 2009, and simulation of available water in storage, 2010–2059. US Geological Survey Scientific Investigations Report 2013–5219, 92 pp. Accessed May 14, 2021 from https://pubs.usgs.gov/sir/2013/5219/pdf/sir20135219_v2.0.pdf

The table below presents a series of published quotes (see quotation marks denoting text quoted from another publication, which is cited following the quotation marks with the full reference written in full below the table). The leftmost column lists a title of a hydrogeologic formation depicted in the cross section on the previous page. The rightmost column presents a quote from a hydrogeological study (see base of table for citation). The quote has been annotated with colored text to highlight how we categorized each layer (i.e., see categories in the center column in the table). Specifically: (i) blue text highlights portions of a quote that provide insights into the degree of consolidation of the formation, (ii) red text highlights portions of a quote that categorize the formation as an aquifer or an aquitard (i.e., higher versus lower permeability in the context of local hydrogeologic formations), and (iii) green text highlights portions of a quote that provide information about the lithology of the formation.

Supplementary Table 60. Hydrostratigraphy details for the Garber-Wellington aquifer

Formation name	Category	Quote
Hennessey Group (confining unit) *(some small-yield wells exist)*	Sedimentary **aquitard** (consolidated or semi-consolidated rock)	"The **Permian-age Hennessey Group** overlies the western part of the Central Oklahoma aquifer and **consists of interbedded red shale, clay, and some siltstone or fine-grained sandstone** (Parkhurst and others, 1996)." (Mashburn, et al., 2014). "the Hennessey Group outcrops in the western one-third of the aquifer area and acts as a **confining layer** because of its **small transmissivity**." (Mashburn, et al., 2014).
Central Oklahoma aquifer *(referred locally as the Garber-Wellington aquifer. Quaternary deposit is often unconsolidated)*	Clastic sedimentary **aquifer** (consolidated or semi-consolidated rock)<br><br>Quaternary deposit of the Central Oklahoma aquifer is unconsolidated.	"The **Garber Sandstone and Wellington Formation** consist of **cross-bedded, fine-grained sandstone with interbedded shale or mudstone** (Bingham and Moore, 1975; Parkhurst and others, 1996; Breit, 1998)." (Mashburn, et al., 2014). "The **Central Oklahoma aquifer** consists of **Quaternary-age alluvium and terrace deposits and Permian-age geologic units** (figs. 4 and 5 and table 2) (Christenson and others, 1990). **Groundwater flows between these geologic units and both the Quaternary-age and Permian-age units are used as a source of groundwater**." (Mashburn, et al., 2014).
Chase, Council Grove and Admire Group	Clastic sedimentary **aquifer** (consolidated or semi-consolidated rock)	"The Chase, Council Grove, and Admire Groups are composed of **cross-bedded, fine-grained sandstone, shale, and thin limestone** (Bingham and Moore, 1975)." (Mashburn, et al., 2014). "**Groundwater-flow** in the Central Oklahoma aquifer is slowest in the confined part of the Garber Sandstone and Wellington Formation and in the **less transmissive parts of the unconfined flow system**, which includes part of the Chase, Council Grove, and Admire Groups." (Mashburn, et al., 2014).
Pennsylvanian geologic units, undifferentiated (includes the Vanoss Formation)	Sedimentary **aquitard** (consolidated or semi-consolidated rock)	"the **Vanoss Formation** is considered to be **a hydrologic boundary of little to no flow**." (Mashburn, et al., 2014). "The Vanoss Formation consists of **red-brown to grey shale and intermittent thin limestone and sandstone beds** that range in **total thickness from 250 to 490 feet** (Bingham and Moore, 1975). This formation acts as a **lower confining unit** to the Central Oklahoma aquifer, **limiting vertical groundwater movement**." (Mashburn, et al., 2014).

Mashburn, S.L., Ryter, D.W., Neel, C.R., Smith, S.J., Correll, J.S., (2014). Hydrogeology and simulation of ground-water flow in the Central Oklahoma (Garber-Wellington) Aquifer, Oklahoma, 1987 to 2009, and simulation of avail-able water in storage, 2010–2059. US Geological Survey Scientific Investigations Report 2013–5219, 92 pp. Accessed June 5, 2022 from https://pubs.er.usgs.gov/publication/sir20135219

3.58 Honey Lake Valley

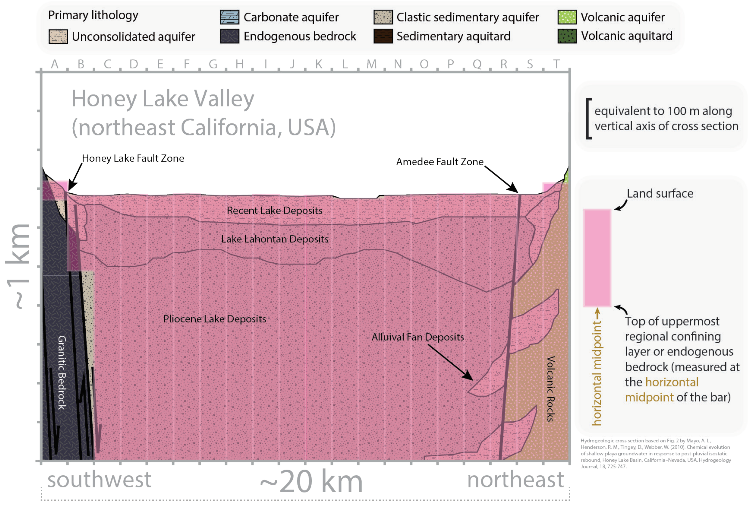

Supplementary Fig. 211. Hydrogeologic cross section. 20 equally spaced transparent pink bars overlie the cross section; each shaded bar depicts the vertical offset from the land surface to the top of the uppermost confining unit or endogenous bedrock.

Honey Lake Valley is located in northeastern California.

(i) A hydrogeologic cross section presented in Fig. 2 by Mayo et al. (2010) suggests that the unconfined portion of Honey Lake Valley extends to a typical depth of greater than 683 m meters below land surface (median of pink bars shown in cross section to the left).

(ii) We could not identify a sufficient number of USGS wells that have been defined as tapping either unconfined or confined conditions.

(iii) In a local-scale study, Mayo et al. (2010) state that (quote) *"Three groundwater systems have been identified in Honey Lake basin: (1) shallow unconfined and semiconfined (<200 m below ground surface (bgs)), (2) deep confined (>200 m bgs), and (3) geothermal".* This quote is consistent with the approximate depth to the top of the Pliocene Lake Deposits presented in the cross section (which exist at a median depth of 162.5 meters below land surface).

Depth to confined conditions: 200-220 m based on (iii)

Reference: Mayo, A. L., Henderson, R. M., Tingey, D., Webber, W. (2010). Chemical evolution of shallow playa groundwater in response to post-pluvial isostatic rebound, Honey Lake Basin, California–Nevada, USA. Hydrogeology Journal, 18, 725-747.

The table below presents a series of published quotes (see quotation marks denoting text quoted from another publication, which is cited following the quotation marks with the full reference written in full below the table). The leftmost column lists a title of a hydrogeologic formation depicted in the cross section on the previous page. The rightmost column presents a quote from a hydrogeological study (see base of table for citation). The quote has been annotated with colored text to highlight how we categorized each layer (i.e., see categories in the center column in the table). Specifically: (i) blue text highlights portions of a quote that provide insights into the degree of consolidation of the formation, (ii) red text highlights portions of a quote that categorize the formation as an aquifer or an aquitard (i.e., higher versus lower permeability in the context of local hydrogeologic formations), and (iii) green text highlights portions of a quote that provide information about the lithology of the formation.

Supplementary Table 61. Hydrostratigraphy details for Honey Lake Valley

**Formation name**	**Category**	**Quote**
Recent Lake and Lake Lahontan Deposits	Unconsolidated aquifer *(unconfined aquifer, and semi-confined aquifer)*	"Honey Lake basin is **filled** with up to 1,700 m of Pliocene and **younger volcanic tuff and ash, and terrestrial, fluvial, and lacustrine sand, silt and clay** (Fig. 2; Handman et al. 1990; California Department of Water Resources 1963, 2003)" (Mayo et al., 2010). "The uppermost 300 m of basin sediments **are pluvial (Pleistocene Lake Lahontan), fluvial and deltaic deposits** (Bonham 1969; Grose et al. 1990; Handman et al. 1990)." (Mayo et al., 2010). "**The pluvial lake and basin margin sediments** support the **shallow groundwater** system described in this investigation." (Mayo et al., 2010).
Pliocene Lake Deposits	Clastic sedimentary **aquifer** (consolidated or semi-consolidated rock)	"Honey Lake basin is **filled** with up to 1,700 m of Pliocene and **younger volcanic tuff and ash, and terrestrial, fluvial, and lacustrine sand, silt and clay** (Fig. 2; Handman et al. 1990; California Department of Water Resources 1963, 2003)" (Mayo et al., 2010). "The uppermost 300 m of basin sediments **are pluvial (Pleistocene Lake Lahontan), fluvial and deltaic deposits** (Bonham 1969; Grose et al. 1990; Handman et al. 1990)." (Mayo et al., 2010). "**The pluvial lake and basin margin sediments** support the **shallow groundwater** system described in this investigation." (Mayo et al., 2010).
Alluvial Fan Deposits	Unconsolidated aquifer *(unconfined aquifer, and semi-confined aquifer)*	"Elsewhere **poorly sorted, highly permeable alluvial fan deposits**, derived from **granodiorite and volcanic terrains, flank the basin and interfinger with Lake Lahontan and younger basin deposits** (California Department of Water Resources 2003; Handman et al. 1990)." (Mayo et al., 2010)
Volcanic Rocks	Volcanic aquifer *(local permeable region exists)*	"Elsewhere volcanic rocks consist of **locally transmissive lava flows (rhyolite, andesite, and basalt), tuff, flow breccia, and volcanic breccia and conglomerate**." (Mayo et al., 2010).
Granitic Bedrock	Endogenous bedrock *(thermal water and local permeable region can exist, discharge to spring)*	"The granitic terrains are locally overlain by **Pliocene–Miocene age volcanic rocks (rhyolite, dacite, andesite, and tuff) that contain phenocrysts of plagioclase (An50–70), pyroxene, hornblende, and olivine in varying proportions (Grose 1984, 1993; Grose et al. 1990, 1993)**." (Mayo et al., 2010). "**Granitic Bedrock**". (Mayo et al., 2010).

Mayo, A. L., Henderson, R. M., Tingey, D., & Webber, W. (2010). Chemical evolution of shallow playa groundwater in response to post-pluvial isostatic rebound, Honey Lake Basin, California–Nevada, USA. *Hydrogeology Journal*, **18**, 725-747.

3.59 Long Island

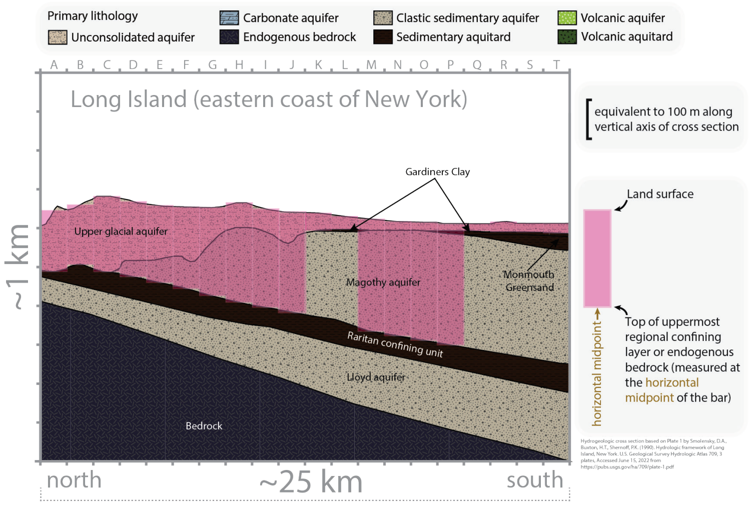

Supplementary Fig. 212. Hydrogeologic cross section. 20 equally spaced transparent pink bars overlie the cross section; each shaded bar depicts the vertical offset from the land surface to the top of the uppermost confining unit or endogenous bedrock.

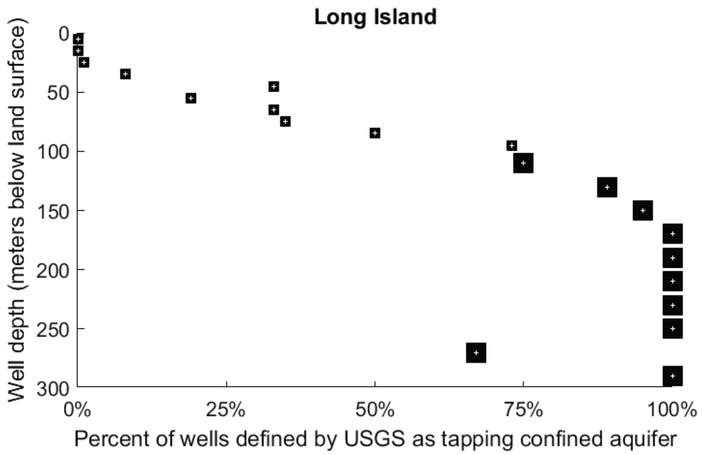

Supplementary Fig. 213. Vertical variations in the prevalence of wells that have been defined as tapping an unconfined or a confined aquifer by the USGS. The smaller squares represent 10 m depth intervals from the land surface to 100 m; the larger squares represent 20 m intervals from 100 m to 300 m below the land surface.

Long Island is located in eastern New York.

(i) A hydrogeologic cross section presented in Plate 1 by Smolensky et al. (1990) suggests that the aquifer system has two primary confining units, the Gardiners Clay and the Raritan Confining Unit.

(ii) We analysed wells within the study area that the USGS has defined as either unconfined or confined. Most (>80%) wells at depths of 120-140 m and at depths exceeding 120 m are defined as tapping a confined aquifer.

Depth to confined conditions: 120-140 m (see (ii) above)

Reference: Smolensky, D.A., Buxton, H.T., Shernoff, P.K. (1990). Hydrologic framework of Long Island, New York. U.S. Geological Survey Hydrologic Atlas 709, 3 plates, Accessed June 15, 2022 from https://pubs.usgs.gov/ha/709/plate-1.pdf

The table below presents a series of published quotes (see quotation marks denoting text quoted from another publication, which is cited following the quotation marks with the full reference written in full below the table). The leftmost column lists a title of a hydrogeologic formation depicted in the cross section on the previous page. The rightmost column presents a quote from a hydrogeological study (see base of table for citation). The quote has been annotated with colored text to highlight how we categorized each layer (i.e., see categories in the center column in the table). Specifically: (i) blue text highlights portions of a quote that provide insights into the degree of consolidation of the formation, (ii) red text highlights portions of a quote that categorize the formation as an aquifer or an aquitard (i.e., higher versus lower permeability in the context of local hydrogeologic formations), and (iii) green text highlights portions of a quote that provide information about the lithology of the formation.

Supplementary Table 62. Hydrostratigraphy details for the Long Island

Formation name	Category	Quote
UG-Upper glacial aquifer	Unconsolidated aquifer	Smolensky et al., 1990 Plate 1, table 1 "**Till composed of clay, sand, gravel, and boulders, forms Harbor Hill and Ronkonkoma terminal moraines. Outwash deposits consist of quartzose sand, fine to very coarse, and gravel, pebble to boulder sized. Also contains lacustrine, marine, and reworked deposits**. Local units are Port Washington aquifer and confining unit, "20-foot clay, and clay at Smithtown.". Smolensky et al., 1990 Plate 1, table 1 "**Till is poorly permeable. Outwash deposits are moderately to highly permeable. Glaciolacustrine and marine clay deposits are mostly poorly permeable but locally have thin, moderately permeable layers of sand and gravel**. **Average horizontal hydraulic conductivity is approximately 270 ft/d**; conductivity of **morainal material** is approximately 50 percent of outwash deposits, anisotropy is approximately 10:1.".
Gc – Gardiners Clay	Sedimentary **aquitard** (consolidated or semi-consolidated rock)	Smolensky et al., 1990 Plate 1, table 1 "**Clay, silt, and few layers of sand**. Colors are grayish green and brown. **Contains marine shells and glauconite**.". Smolensky et al., 1990 Plate 1, table 1 "**Poorly permeable**; constitutes **a confining layer** for underlying aquifer. **Some sand lenses may be permeable Average vertical hydraulic conductivity is approximately 0.001 ft/d**.".
Mg – Monmouth greensand	Sedimentary **aquitard** (consolidated or semi-consolidated rock)	Smolensky et al., 1990 Plate 1, table 1 "**Interbedded marine deposits of clay, silt, and sand. Dark-greenish gray. Greenish-black, greenish, dark-gray. And black, containing much glauconite**.". Smolensky et al., 1990 Plate 1, table 1 "**Poorly permeable**: primarily a confining unit for underlying Magothy aquifer. **Average vertical hydraulic conductivity is approximately 0.001 ft/d**.".
M – Magothy aquifer	Sedimentary **aquifer** (consolidated or semi-consolidated rock)	Smolensky et al., 1990 Plate 1, table 1 "**Sand, fine to medium clayey in part: interbedded with lenses and layers of coarse sand and sandy and solid clay. Gravel is common in basal zone, Sand and gravel are quartzose. Lignite, pyrite, and iron oxide concretions are common**. Colors are gray, white, red brown, and yellow.". Smolensky et al., 1990 Plate 1, table 1 "**Most layers are poorly to moderately permeable**; **some are highly permeable locally. Water is unconfined in uppermost parts, elsewhere is confined**. **Constitutes principal aquifer for**

Formation name	Category	Quote
		**public supply. Average horizontal hydraulic conductivity is 50 ft/d**; anistrophy is approximately 100:1.”.
Rc – Raritan confining unit	Sedimentary **aquitard** (consolidated or semi-consolidated rock)	Smolensky et al., 1990 Plate 1, table 1 “**Clay, solid and silty; few lenses and layers of sand. Lignite and pyrite are common**. Colors are gray, red, and white, commonly variegated.”. Smolensky et al., 1990 Plate 1, table 1 “**Poorly to very poorly permeable; constitutes confining layer** for underlying Lloyd aquifer. **Average vertical hydraulic conductivity is approximately 0.001 ft/d**.”.
L – Lloyd aquifer	Sedimentary **aquifer** (consolidated or semi-consolidated rock)	Smolensky et al., 1990 Plate 1, table 1 “**Sand, fine to coarse, and gravel, commonly with clayey matrix; some lenses and layers of solid and silty clay; locally contains thin lignite layers. Sand and most of gravel are quartzose**. Colors are yellow, gray, and white; clay is red locally.”. Smolensky et al., 1990 Plate 1, table 1 “**Poorly to moderately permeable**. Water is **confined by overlying Raritan clay**. **Average horizontal hydraulic conductivity is 40ft/d**; anisotropy is approximately 10:1.”.
Br – Bedrock	Endogenous bedrock	Smolensky et al., 1990 Plate 1, table 1 “**Crystalline metamorphic and igneous rocks; muscovite-biotite schist, gneiss, and granite. A soft, clayey zone of weathered bedrock locally is more than 70 ft thick.**”. Smolensky et al., 1990 Plate 1, table 1 “**Poorly permeable to virtually impermeable; constitutes lower boundary of ground-water reservoir. Some hard fresh water is contained in joints and fractures but is impractical to develop at most places**.”.

Smolensky, D.A., Buxton, H.T., Shernoff, P.K. (1990). Hydrologic framework of Long Island, New York. U.S. Geological Survey Hydrologic Atlas 709, 3 plates, Accessed June 15, 2022 from https://pubs.usgs.gov/ha/709/plate-1.pdf

3.60 Los Angeles Basin

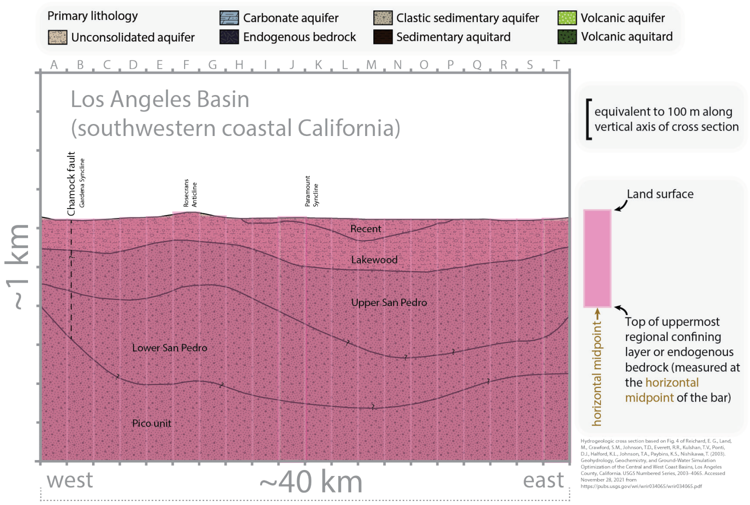

Supplementary Fig. 214. Hydrogeologic cross section. 20 equally spaced transparent pink bars overlie the cross section; each shaded bar depicts the vertical offset from the land surface to the top of the uppermost confining unit or endogenous bedrock.

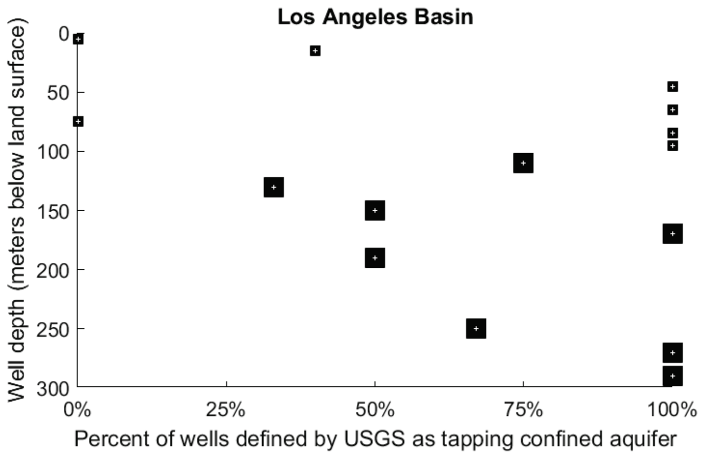

Supplementary Fig. 215. Vertical variations in the prevalence of wells that have been defined as tapping an unconfined or a confined aquifer by the USGS. The smaller squares represent 10 m depth intervals from the land surface to 100 m; the larger squares represent 20 m intervals from 100 m to 300 m below the land surface.

The Los Angeles Basin is located in coastal southern California.

(i) A hydrogeologic cross section presented in Fig. 4 by Reichard et al. (2003) does not depict a continuous confining unit in the study area.

(ii) We analysed wells within the study area that the USGS has defined as either unconfined or confined. We could not identify a depth where most (>80%) wells with the depth range and at deeper depths are defined as tapping a confined aquifer. Three of the four deepest wells in our dataset (depths of 396 m, 399 m, 445 m, and 472 m) are classified as unconfined.

Depth to confined conditions: >472 m (see (ii) above)

Reference: Reichard, E. G., Land, M., Crawford, S.M., Johnson, T.D., Everett, R.R., Kulshan, T.V., Ponti, D.J., Halford, K.L., Johnson, T.A., Paybins, K.S., Nishikawa, T. (2003). Geohydrology, Geochemistry, and Ground-Water Simulation Optimization of the Central and West Coast Basins, Los Angeles County, California. USGS Numbered Series, 2003–4065. Accessed November 28, 2021 from https://pubs.usgs.gov/wri/wrir034065/wrir034065.pdf

The table below presents a series of published quotes (see quotation marks denoting text quoted from another publication, which is cited following the quotation marks with the full reference written in full below the table). The leftmost column lists a title of a hydrogeologic formation depicted in the cross section on the previous page. The rightmost column presents a quote from a hydrogeological study (see base of table for citation). The quote has been annotated with colored text to highlight how we categorized each layer (i.e., see categories in the center column in the table). Specifically: (i) blue text highlights portions of a quote that provide insights into the degree of consolidation of the formation, (ii) red text highlights portions of a quote that categorize the formation as an aquifer or an aquitard (i.e., higher versus lower permeability in the context of local hydrogeologic formations), and (iii) green text highlights portions of a quote that provide information about the lithology of the formation.

Supplementary Table 63. Hydrostratigraphy details for Los Angeles Basin

Formation name	Category	Quote
Recent	Unconsolidated aquifer	"The geohydrologic units that compose the Holocene (Recent) age deposits of the Recent aquifer system include the **semiperched aquifer, the Bellflower aquiclude, the Gaspur aquifer, and the Ballona aquifer** (California Department of Water Resources, 1961). Although these geohydrologic units are referred to in this report as consisting of **Holocene-age deposits**, some of these units consist of deposits of Pleistocene age. The semiperched aquifer is a relatively **thin layer of coarse sand and gravel near the land surface; it consists of alluvial sediments and, in parts of the West Coast Basin, marine deposits that may include the late Pleistocene Palos Verdes Sand**." (Reichard et al., 2003).
Lakewood	Unconsolidated aquifer	"Generally, the **Lakewood aquifer system** is a heterogeneous unit dominated **by sandy silts and silty sands interbedded with sands that become coarser and thicker** near the **base of the aquifer system**." (Reichard et al., 2003).
Upper San Pedro	Clastic sedimentary **aquifer** (consolidated or semi-consolidated rock)	"The **Upper San Pedro aquifer system incorporates the Hollydale, Jefferson, Lynwood, and Silverado aquifers** (fig. 3)." (Reichard et al.,2003). "Overall, the aquifer system appears to be of mixed origin, **with nonmarine deposits consisting of sand and gravel that are interbedded with silt and clay, and marine deposits characterized by blue-gray sand, gravel, silt, and clay, along with shells and wood fragments**." (Reichard et al., 2003).
Lower San Pedro	Clastic sedimentary **aquifer** (consolidated or semi-consolidated rock)	"**The Lower San Pedro aquifer system includes the Sunnyside aquifer (also referred to as the Lower San Pedro aquifer)**. The upper part of this system tends to be characterized by alternating **fine-grained and coarse-grained zones**. The fine-grained zones tend to pinch out or disappear near the forebay margins, such as at USGS Pico Rivera-1 (2S/11W-18C4–7) and 1S/13W-34F (fig. 4*A*,*B*). The **coarsest part of the aquifer system generally is at the base and is as much as 100 ft thick**." (Reichard et al., 2003).

Formation name	Category	Quote
Pico unit	Clastic sedimentary **aquifer** (consolidated or semi-consolidated rock)	"The **Pico aquifer**, which underlies the entire area from Bolsa Chica Mesa to Newport Mesa, ranges in thickness from about **1,200 feet at Huntington Beach Mesa to less than 1 foot** at Newport Mesa. The **permeable zones** in the aquifer are generally of **fine to medium sand with permeability ranging from 200 to 300 gpd / ft 2** (gallons per day per square foot)." (Moreland and Singer, 1969).

Reichard, E. G., Land, M., Crawford, S.M., Johnson, T.D., Everett, R.R., Kulshan, T.V., Ponti, D.J., Halford, K.L., Johnson, T.A., Paybins, K.S., Nishikawa, T. (2003). Geohydrology, Geochemistry, and Ground-Water Simulation Optimization of the Central and West Coast Basins, Los Angeles County, California. USGS Numbered Series, 2003–4065. Accessed June 10, 2022 via https://pubs.usgs.gov/wri/wrir034065/wrir034065.pdf

Moreland, J. A., Singer, J. A. (1969). A study of deep aquifers underlying coastal Orange County, California. US Geological Survey, Water Resources Division. Accessed June 10, 2022 via https://pubs.er.usgs.gov/publication/70047681

3.61 Michigan Basin

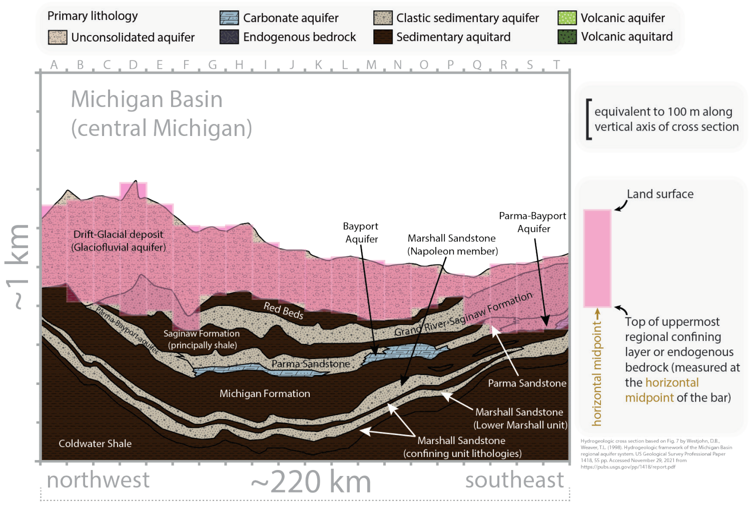

Supplementary Fig. 216. Hydrogeologic cross section. 20 equally spaced transparent pink bars overlie the cross section; each shaded bar depicts the vertical offset from the land surface to the top of the uppermost confining unit or endogenous bedrock.

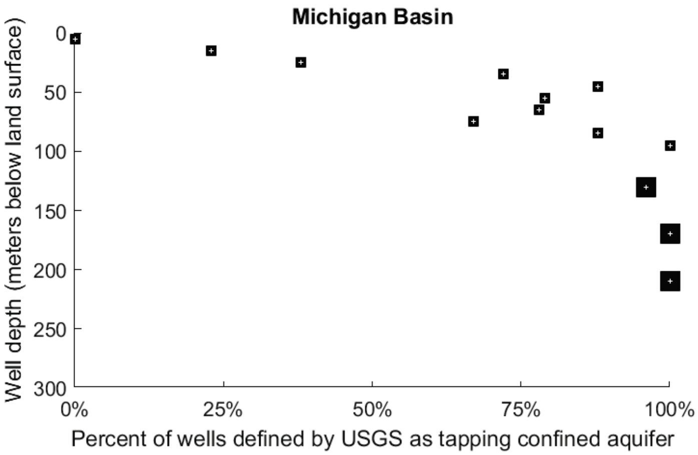

Supplementary Fig. 217. Vertical variations in the prevalence of wells that have been defined as tapping an unconfined or a confined aquifer by the USGS. The smaller squares represent 10 m depth intervals from the land surface to 100 m; the larger squares represent 20 m intervals from 100 m to 300 m below the land surface.

The Michigan Basin is located in central Michigan.

(i) A hydrogeologic cross section presented in Fig. 7 by Westjohn et al. (1998) depicts a series of stacked confining units underlying relatively thick (>100 m) glacial deposits.

(ii) We analysed wells within the study area that the USGS has defined as either unconfined or confined. Most (>80%) wells at depths of 40-50 m and at depths exceeding 40 m are defined as tapping a confined aquifer.

Depth to confined conditions: 40-50 m (see (ii) above)

Reference: Westjohn, D.B., Weaver, T.L. (1998). Hydrogeologic framework of the Michigan Basin regional aquifer system. US Geological Survey Professional Paper 1418, 55 pp. Accessed November 29, 2021 from https://pubs.usgs.gov/pp/1418/report.pdf

The table below presents a series of published quotes (see quotation marks denoting text quoted from another publication, which is cited following the quotation marks with the full reference written in full below the table). The leftmost column lists a title of a hydrogeologic formation depicted in the cross section on the previous page. The rightmost column presents a quote from a hydrogeological study (see base of table for citation). The quote has been annotated with colored text to highlight how we categorized each layer (i.e., see categories in the center column in the table). Specifically: (i) blue text highlights portions of a quote that provide insights into the degree of consolidation of the formation, (ii) red text highlights portions of a quote that categorize the formation as an aquifer or an aquitard (i.e., higher versus lower permeability in the context of local hydrogeologic formations), and (iii) green text highlights portions of a quote that provide information about the lithology of the formation.

Supplementary Table 64. Hydrostratigraphy details for the Michigan Basin

Formation name	Category	Quote
Drift – Glacial deposits (undifferentiated) *(fine grained glacial lacustrine and till are assumed to function as confining units.)*	Unconsolidated aquifer	"Glacial deposits in the study area can be separated into three general provinces (fig. 10): (1) **glacial deposits in the southern part are primarily recessional moraines and outwash deposits that formed at the front of retreating ice lobes; (2) surficial deposits in the Saginaw Lowlands are primarily basal-lodgment tills and fine grained lacustrine sediments that were deposited in former proglacial lakes; and (3) glacial deposits of the northern half of the study area are primarily glaciofluvial deposits and some coarse-textured till** (Farrand and Bell, 1982)." (Westjohn &Weaver, 1998). "**Glaciofluvial deposits** are the **largest source of fresh ground water** in Michigan and in the RASA study area." (Westjohn &Weaver, 1998).
Red Beds	Sedimentary **aquitard** (consolidated or semi-consolidated rock)	"Shaffer (1969) indicates that predominant lithologies of **the "red beds" sequence** are **red clay, mudstone, siltstone, and sandstone; as well as gray-green shale, mudstone, and gypsum**.". (Westjohn &Weaver, 1998). "On the basis of data from geophysical logs, **permeable sandstone within Jurassic "red beds"** is **not volumetrically important and most of the sequence is probably of low permeability**." (Westjohn & Weaver, 1998).
Grand River – Saginaw Formation	Sedimentary **aquifer** (consolidated or semi-consolidated rock)	"The **Grand River Formation** is reported to consist predominantly of **sandstone, although Kelly (1936, p. 209) indicates that a conglomerate bed** at the type locality separates the Grand River Formation from the underlying Saginaw Formation." (Westjohn & Weaver, 1998). "Hydrogeologic units that include all or parts of two stratigraphic units are the **Saginaw aquifer** (**sandstones** of the **Grand River Formation and the Saginaw Formation**), the Parma-Bayport aquifer (sandstones and permeable carbonates of the Parma Sandstone and the Bayport Limestone), and the Marshall aquifer (composite of stratigraphically continuous, permeable sandstones of the Michigan Formation and the Marshall Sandstone)." (Westjohn & Weaver, 1998).
Saginaw Formation (principally shale) *(underlying Saginaw shale*	Sedimentary **aquitard** (consolidated or semi-consolidated rock)	"The **Saginaw confining unit** separates the Saginaw aquifer from the underlying Parma-Bayport aquifer in most of the study area (fig. 5). **This confining unit** consists **mostly of shale; the rest of the sequence consists of thin beds of sandstone, siltstone, coal, and limestone**." (Westjohn & Weaver, 1998).

Formation name	Category	Quote
dominated unit is confining layer)		
Parma Sandstone	Sedimentary **aquifer** (consolidated or semi-consolidated rock)	"The **Parma Sandstone**, which consists of **medium- to coarse-grained sandstone**, is typically less than 100 ft thick (Cohee and others, 1951).". (Westjohn &Weaver, 1998). "Geophysical logs show that these units consist mostly of **permeable sandstones and carbonates** and that the formations are hydraulically connected throughout the area of the regional aquifer system. For characterization of hydrogeologic setting and computer simulation of ground-water flow, these units are combined as the **Parma-Bayport aquifer**." (Westjohn & Weaver, 1998).
Bayport Limestone	Carbonate aquifer	"The **Bayport Limestone** of Late Mississippian age consists of sparsely **fossiliferous to highly fossiliferous limestone, dolostone, sandy limestone, cherty limestone, and sandstone** (Bacon, 1971; Ciner, 1988; Lasemi, 1975; Tyler, 1980).". (Westjohn &Weaver, 1998). "Hydrogeologic units that include all or parts of two stratigraphic units are the Saginaw aquifer (sandstones of the Grand River Formation and the Saginaw Formation), the **Parma-Bayport aquifer** (sandstones and permeable carbonates of the Parma Sandstone and the **Bayport Limestone**), and the Marshall aquifer (composite of stratigraphically continuous, permeable sandstones of the Michigan Formation and the Marshall Sandstone)." (Westjohn & Weaver, 1998).
Michigan Formation	Sedimentary **aquitard** (consolidated or semi-consolidated rock)	"The **Michigan confining unit** is composed of **all low permeability** lithologies of the Michigan Formation, and does not include stratigraphically continuous sandstones at or near the base of the formation. This confining unit separates the Parma-Bayport aquifer from the Marshall aquifer (fig. 5). The **Michigan confining unit** consists **of shale, carbonate, evaporite, and thin, laterally discontinuous siltstone and sandstone lenses**." (Westjohn & Weaver, 1998).
Marshall Sandstone (Napoleon member, lower Marshall unit, and confining unit lithologies) *(sand matrix can limit groundwater flow)*	Sedimentary **aquifer** (consolidated or semi-consolidated rock)	"**Sandstone** forms only part of the formation. **Limestone, dolomite, siltstone, and shale** are **interbedded with sandstones** of the Marshall sedimentary sequence in different parts of the basin." (Westjohn & Weaver, 1998). "The **Marshall aquifer** consists of one or more blanket type **sandstones** of Mississippian age that RASA investigators assume are hydraulically connected at the scale of the regional aquifer system." (Westjohn & Weaver, 1998).
Coldwater Shale	Sedimentary **aquitard** (consolidated or semi-consolidated rock)	"The **Coldwater confining unit** is the base of the regional aquifer system. This basal **confining unit consists** mostly of **shale**. **Siltstone, sandstone, limestone, and dolomite** are part of this hydrogeologic unit in some areas of the basin.". (Westjohn & Weaver, 1998).

Westjohn, D.B., Weaver, T.L. (1998). Hydrogeologic framework of the Michigan Basin regional aquifer system. US Geological Survey Professional Paper 1418, 55 pp. Accessed November 29, 2021 from https://pubs.usgs.gov/pp/1418/report.pdf

3.62 Mojave Basin

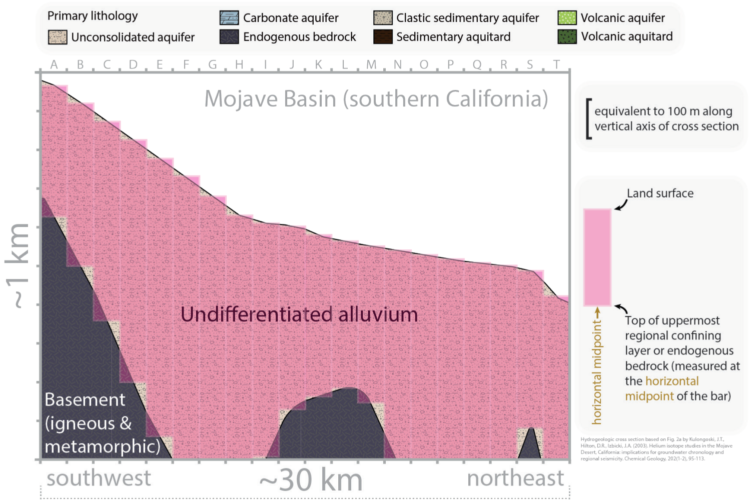

Supplementary Fig. 218. Hydrogeologic cross section. 20 equally spaced transparent pink bars overlie the cross section; each shaded bar depicts the vertical offset from the land surface to the top of the uppermost confining unit or endogenous bedrock.

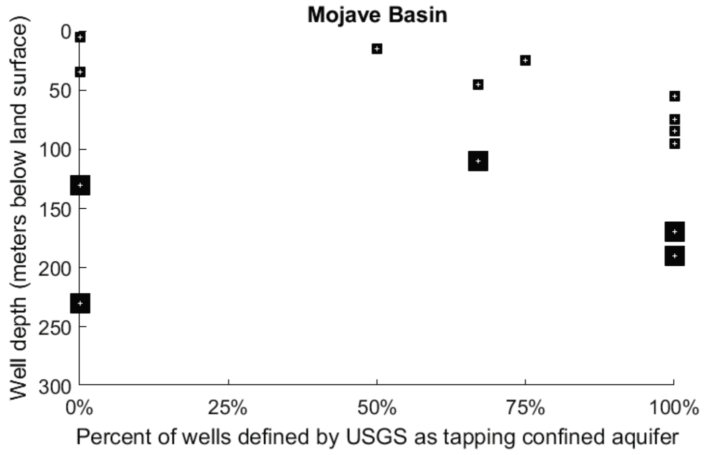

Supplementary Fig. 219. Vertical variations in the prevalence of wells that have been defined as tapping an unconfined or a confined aquifer by the USGS. The smaller squares represent 10 m depth intervals from the land surface to 100 m; the larger squares represent 20 m intervals from 100 m to 300 m below the land surface.

The Mojave Basin is located in southern California.

(i) A hydrogeologic cross section presented in Fig. 2a by Kulongoski et al. (2003) does not depict a clear confining unit within the aquifer system. The median depth to basement igneous and metamorphic rocks as depicted in the cross section is >512 m.

(ii) We could not identify a depth where most (>80%) wells with the depth range and at deeper depths are defined as tapping a confined aquifer. The two deepest wells in our dataset (depths of 228 m and 314 m) are classified as unconfined.

(iii) Kulongoski et al. (2003) highlight that unconfined conditions prevail in parts of the aquifer system; they state (following text quoted directly): *“These deposits (QTa) consist of unconsolidated to moderately consolidated gravel, sand, silt, and clay deposited in the Pleistocene and late Pliocene, and overlie a crystalline complex of igneous and metamorphic rocks (pTb), (California Dept. Of Water Resources, 1967). Waters from this mostly unconfined aquifer…”*

Depth to confined conditions: >512 m (see (i) and (ii) above)

Reference: Kulongoski, J.T., Hilton, D.R., Izbicki, J.A. (2003). Helium isotope studies in the Mojave Desert, California: implications for groundwater chronology and regional seismicity. Chemical Geology, 202, 95-113.

The table below presents a series of published quotes (see quotation marks denoting text quoted from another publication, which is cited following the quotation marks with the full reference written in full below the table). The leftmost column lists a title of a hydrogeologic formation depicted in the cross section on the previous page. The rightmost column presents a quote from a hydrogeological study (see base of table for citation). The quote has been annotated with colored text to highlight how we categorized each layer (i.e., see categories in the center column in the table). Specifically: (i) blue text highlights portions of a quote that provide insights into the degree of consolidation of the formation, (ii) red text highlights portions of a quote that categorize the formation as an aquifer or an aquitard (i.e., higher versus lower permeability in the context of local hydrogeologic formations), and (iii) green text highlights portions of a quote that provide information about the lithology of the formation.

Supplementary Table 65. Hydrostratigraphy details for the Mojave Basin

Formation name	Category	Quote
Undifferentiated alluvium	Unconsolidated aquifer	"The **undifferentiated alluvium** (QTa), which forms the regional **aquifer**, is more than 1000 m thick at some locations (Subsurface Surveys, 1990), and consists of **alluvial and basin-fill deposits**." Kulongoski et al. (2003)
Igneous and metamorphic basement complex	Endogenous bedrock	"The pre-Tertiary **basement complex** typically has **low porosity and permeability**, yielding **only small quantities of water to wells**; however, where the basement complex is intensely fractured, as along major faults, the bedrock is more permeable." Stamos et al. (2001)

Kulongoski, J.T., Hilton, D.R., Izbicki, J.A. (2003). Helium isotope studies in the Mojave Desert, California: implications for groundwater chronology and regional seismicity. Chemical Geology, 202(1-2), 95-113.

Stamos, C.L., Martin, P., Nishikawa, T., Cox, B.F. (2001). Simulation of ground-water flow in the Mojave River Basin, California. US Geological Survey Water-Resources Investigations Report, 01-4002.

3.63 Northern Green River Basin

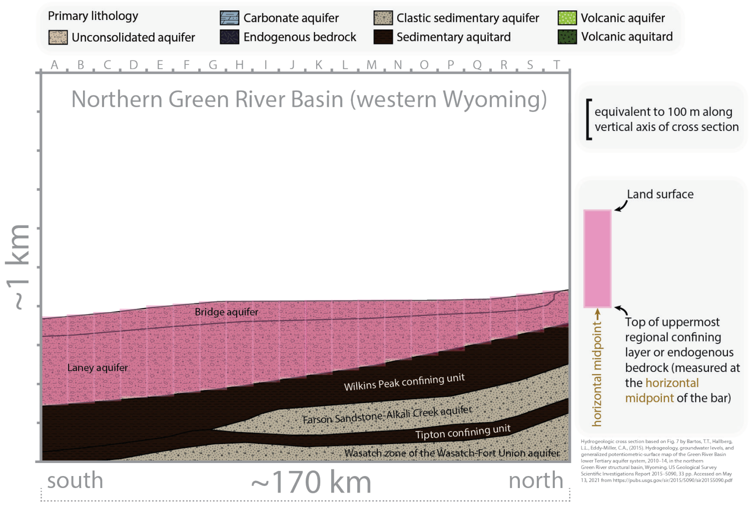

Supplementary Fig. 220. Hydrogeologic cross section. 20 equally spaced transparent pink bars overlie the cross section; each shaded bar depicts the vertical offset from the land surface to the top of the uppermost confining unit or endogenous bedrock.

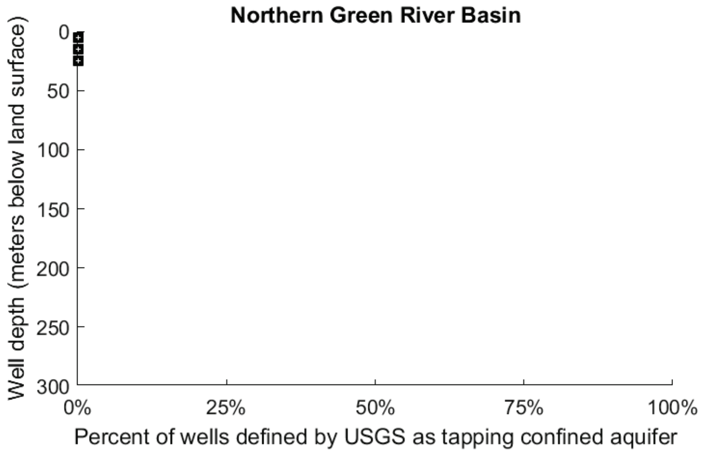

Supplementary Fig. 221. Vertical variations in the prevalence of wells that have been defined as tapping an unconfined or a confined aquifer by the USGS. The smaller squares represent 10 m depth intervals from the land surface to 100 m; the larger squares represent 20 m intervals from 100 m to 300 m below the land surface.

The Northern Green River Basin is located in western Wyoming.

(i) Fig. 7 by Bartos et al. (2015) suggests that the top if the uppermost confining unit is typically 193 meters below land surface (i.e., the median length of the pink transparent bars in cross section; the lower-upper quartile range is 137-225 meters).

(ii) The available USGS well data are insufficient to evaluate the depths at which the aquifer system transitions from unconfined to confined conditions. Wells with depths of 335 m and 416 m are classified as confined, where the shallowest wells (all with depths of less than 26 m) are classified as unconfined.

(iii) Bartos et al. (2015) state, with regards to the presence of confining units, that (quote) *"Confining units are the Wilkins Peak and Tipton confining units; where impermeable, the Laney Member of the Green River Formation also is a confining unit."* As the Laney Formation overlies the Wilkins Formation, it is possible that confined conditions prevail at shallower depths than the top of the Wilkins Formation. Our estimated depth to confined conditions may overestimate actual depths to confined conditions in some areas.

Depth to confined conditions: 180-200 meters based on (i)

Reference: Bartos, T.T., Hallberg, L.L., Eddy-Miller, C.A., (2015). Hydrogeology, groundwater levels, and generalized potentiometric-surface map of the Green River Basin Lower Tertiary aquifer system, 2010–14, in the northern Green River structural basin, Wyoming. US Geological Survey Scientific Investigations Report 2015–5090, 33 pp. Accessed on May 13, 2021 from https://pubs.usgs.gov/sir/2015/5090/sir20155090.pdf

The table below presents a series of published quotes (see quotation marks denoting text quoted from another publication, which is cited following the quotation marks with the full reference written in full below the table). The leftmost column lists a title of a hydrogeologic formation depicted in the cross section on the previous page. The rightmost column presents a quote from a hydrogeological study (see base of table for citation). The quote has been annotated with colored text to highlight how we categorized each layer (i.e., see categories in the center column in the table). Specifically: (i) blue text highlights portions of a quote that provide insights into the degree of consolidation of the formation, (ii) red text highlights portions of a quote that categorize the formation as an aquifer or an aquitard (i.e., higher versus lower permeability in the context of local hydrogeologic formations), and (iii) green text highlights portions of a quote that provide information about the lithology of the formation.

Supplementary Table 66. Hydrostratigraphy details for Northern Green River Basin

**Formation name**	**Category**	**Quote**
Bridge aquifer	Unconsolidated aquifer	"**Aquifers**, from top to bottom, are the **Bridger**, Laney, Farson Sandstone-Alkali Creek, and Wasatch-Fort Union aquifers (fig. 6)." (Bartos et al., 2015). "Complex **intertonguing fluvial and lacustrine sediments**. The Wasatch Formation, the **Fowkes/Bridger Formations in the southwest Overthrust** and Green River Basins near outcrop, as well as Ft Union in Great Divide, Washakie, and Little Snake Basins are major aquifers." States West Water Resources Corporation (2001)
Laney aquifer	Unconsolidated aquifer *(it could be confining unit where impermeable, Groundwater in the sandstone aquifers is under unconfined (water table) and confined (artesian) conditions.)*	"**Aquifers**, from top to bottom, are the Bridger, **Laney**, Farson Sandstone-Alkali Creek, and Wasatch-Fort Union aquifers (fig. 6)." (Bartos, et al., 2015). "The Laney Member of the Green River Formation consists of **interbedded layers** and lenses of **oil shale; marlstone; tuffaceous sandstone and siltstone; and gray, tan, or green sandstone and mudstone** (Roehler, 1991a, 1991b, 1992a, 1992b, 1992c)." (Bartos et al., 2015).
Wilkins Peak confining unit	Sedimentary **aquitard** (consolidated or semi-consolidated rock)	"**Confining units** are the **Wilkins Peak** and Tipton **confining units**;". (Bartos et al., 2015).
Farson Sandstone-Alkali Creek Aquifer	Clastic sedimentary **aquifer** (consolidated or semi-consolidated rock) *(Groundwater in the sandstone aquifers is under unconfined (water table) and confined (artesian) conditions.)*	"**Aquifers**, from top to bottom, are the Bridger, Laney, **Farson Sandstone-Alkali Creek**, and Wasatch-Fort Union aquifers (fig. 6)." (Bartos, et al., 2015). "The laterally equivalent Farson **Sandstone** Member of the Green River Formation consists of gray, tan, and brown **sandstone; thin interbedded**, **gray shale and siltstone of lacustrine origin; and locally occurring conglomerate**." (Bartos et al., 2015).
Tipton confining unit	Sedimentary **aquitard** (consolidated or semi-consolidated rock)	"**Confining units** are the Wilkins Peak and **Tipton confining units**;". (Bartos et al., 2015).

Formation name	Category	Quote
Wasatch zone of the Wasatch-Fort Union aquifer	Clastic sedimentary **aquifer** *(Groundwater in the sandstone aquifers is under unconfined (water table) and confined (artesian) conditions.)*	"**Aquifers**, from top to bottom, are the Bridger, Laney, Farson Sandstone-Alkali Creek, and **Wasatch-Fort Union aquifers** (fig. 6)." (Bartos, et al., 2015). "Wasatch Formation consists of **interbedded freshwater-deposited brown, green, and gray sandstone, siltstone, mudstone, and shale; and locally conglomeratic lenses** (Roehler, 1991a, 1992a, 1992b, 1992c)." (Bartos et al., 2015).
Uinta Mountain	Endogenous bedrock	"**Transmissivity also is less along the Uinta Mountains resulting in relatively steep hydraulic gradients** compared to the central part of the basin." (Bartos, et al., 2015). "The Uinta Mountain group (Uinta quartzite of previous reports) a **series of brick-red to purplish-red quartzite and sandstone beds of pre-Cambrian age**, aggregating more than 12,000 feet in thickness, makes up the central mass of the range." (Bradley, 1936).

Bartos, T.T., Hallberg, L.L., Eddy-Miller, C.A. (2015). *Hydrogeology, groundwater levels, and generalized potentiometric-surface map of the Green River Basin Lower Tertiary aquifer system, 2010–14, in the northern Green River structural basin* (No. 2015-5090). US Geological Survey. Accessed August 10, 2022 via https://pubs.usgs.gov/sir/2015/5090/sir20155090.pdf

Bradley, W. H. (1936). *Geomorphology of the north flank of the Uinta Mountains* (No. 185-I, pp. 163-199). United States Government Printing Office. Accessed August 10, 2022 via https://pubs.er.usgs.gov/publication/pp185I

States West Water Resources Corporation (2001). Green River Basin Water Planning Process. Wyoming Water Development Commission Basin Planning Program. (Final report, pp. 178) accessed 5/17/2022 via https://waterplan.state.wy.us/plan/green/finalrept/finalrept_lores.pdf

3.64 Ozark Plateaus Aquifer System

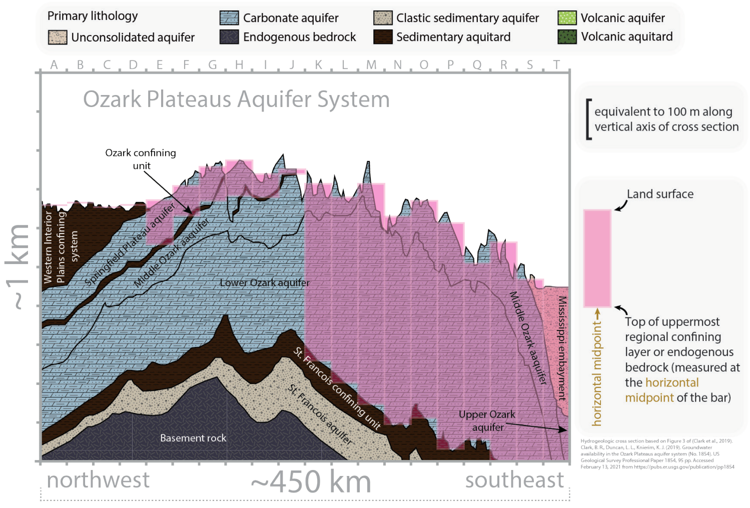

Supplementary Fig. 222. Hydrogeologic cross section. 20 equally spaced transparent pink bars overlie the cross section; each shaded bar depicts the vertical offset from the land surface to the top of the uppermost confining unit or endogenous bedrock.

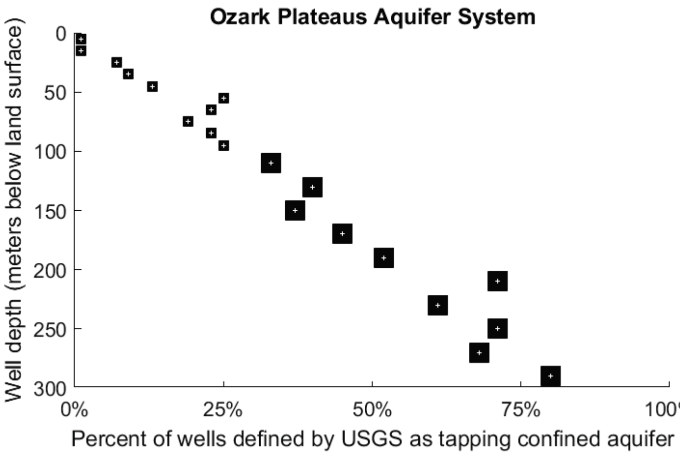

Supplementary Fig. 223. Vertical variations in the prevalence of wells that have been defined as tapping an unconfined or a confined aquifer by the USGS. The smaller squares represent 10 m depth intervals from the land surface to 100 m; the larger squares represent 20 m intervals from 100 m to 300 m below the land surface.

The Ozark Plateaus Aquifer System is located in southern Missouri, northwestern Arkansas, northeastern Oklahoma, and southeastern Kansas.

(i) A hydrogeologic cross section presented in Fig. 3 by Clark et al. (2019) shows thick sequences of carbonate rock with interbedded confining units. The St. Francois confining unit exists at deep depths (>300 m) and, in the northwest, a shallower and thin confining unit exists (the Ozark confining unit).

(ii) We analysed wells within the study area that the USGS has defined as either unconfined or confined. Most (>80%) wells at depths of 280-300 m and at depths exceeding 280 m are defined as tapping a confined aquifer.

Depth to confined conditions: 280-300 meters below land surface (based on (ii) above)

Reference: Clark, B. R., Duncan, L. L., Knierim, K. J. (2019). Groundwater availability in the Ozark Plateaus aquifer system. US Geological Survey Professional Paper 1854, 95 pp. Accessed February 13, 2021 from https://pubs.er.usgs.gov/publication/pp1854

The table below presents a series of published quotes (see quotation marks denoting text quoted from another publication, which is cited following the quotation marks with the full reference written in full below the table). The leftmost column lists a title of a hydrogeologic formation depicted in the cross section on the previous page. The rightmost column presents a quote from a hydrogeological study (see base of table for citation). The quote has been annotated with colored text to highlight how we categorized each layer (i.e., see categories in the center column in the table). Specifically: (i) blue text highlights portions of a quote that provide insights into the degree of consolidation of the formation, (ii) red text highlights portions of a quote that categorize the formation as an aquifer or an aquitard (i.e., higher versus lower permeability in the context of local hydrogeologic formations), and (iii) green text highlights portions of a quote that provide information about the lithology of the formation.

Supplementary Table 67. Hydrostratigraphy details for the Ozark Plateau aquifer system

**Formation name**	**Category**	**Quote**
Western Interior Plain confining unit	Sedimentary **aquitard** (consolidated or semi-consolidated rock)	"The Ozark system is regionally overlain by the **Western Interior Plains confining system** in the southern and western extents of the study area (fig. 1). The **confining system** is mostly composed of **shale with lesser amounts of limestone and sandstone**." (Clark et al., 2019).
Springfield Plateau aquifer	Carbonate aquifer	" The uppermost aquifer of the Ozark system is the **Springfield Plateau aquifer** of Mississippian age, which consists of **limestone with varying chert** abundance and **has a median thickness of 237 ft** (Hays and others, 2016; Westerman and others, 2016a)." (Clark et al., 2019).
Ozark confining unit	Sedimentary **aquitard** (consolidated or semi-consolidated rock)	"The **Ozark confining unit** is **relatively thin (median thickness is 42 ft; Westerman and others, 2016a) to absent** in some areas, which permits hydraulic connection of the underlying Ozark aquifer and overlying Springfield Plateau aquifer (fig. 2). Lithology of the **Ozark confining unit** varies throughout the study area, but is generally composed of **low-permeability limestone, sandstone, and shale units** (Hays and others, 2016). " (Clark et al., 2019).
Middle Ozark aquifer	Carbonate aquifer	""The **Ozark aquifer** includes productive **dolostone units** of the lower Ozark aquifer (median thickness of 885 ft), denser and relatively lower permeability **dolostones** of **the middle Ozark aquifer** (median thickness of 416 ft), and the mixed lithology of **limestone, dolostone, shale, and limited sandstone** units of the upper Ozark aquifer (median thickness of 590 ft) (Hays and others, 2016; Imes and Emmett, 1994; Westerman and others, 2016a) (fig. 2)." (Clark et al., 2019)." (Clark et al., 2019). " The **middle Ozark aquifer** therefore serves as an important groundwater resource, despite **lower permeability than some of the more karstified units** such as the Springfield Plateau aquifer or the lower Ozark aquifer. " (Clark et al., 2019).
Lower Ozark aquifer	Carbonate aquifer	" The **lower Ozark aquifer** is generally the most productive part of the Ozark aquifer owing to the **enhanced secondary and tertiary porosity and permeability from karst formations** (Hays and others, 2016)." (Clark et al., 2019).
St. Francois confining unit	Sedimentary **aquitard** (consolidated or semi-consolidated rock)	" The St. Francois aquifer is confined throughout much of its extent where overlain by the **St. Francois confining unit**. The **St. Francois confining unit of Cambrian age** has a median thickness of 228 ft (Westerman and others, 2016a) and **is composed of low-permeability shale, siltstone,**

Formation name	Category	Quote
		**dolostone, and limestone** (Hays and others, 2016)." (Clark et al., 2019).
St. Francois aquifer	Sedimentary **aquifer** (consolidated or semi-consolidated rock)	" The basal hydrogeologic unit of the Ozark system is the **St. Francois aquifer of Cambrian age** (fig. 2), which has a **median thickness of 291 ft** (Westerman and others, 2016b) and is **composed of permeable sandstones and dolostones** (Hays and others, 2016). " (Clark et al., 2019).
Upper Ozark aquifer	Carbonate aquifer	"The **Ozark aquifer** includes productive **dolostone units** of the lower Ozark aquifer (median thickness of 885 ft), denser and relatively lower permeability **dolostones** of the middle Ozark aquifer (median thickness of 416 ft), and the mixed lithology of **limestone, dolostone, shale, and limited sandstone** units of the **upper Ozark aquifer** (median thickness of 590 ft) (Hays and others, 2016; Imes and Emmett, 1994; Westerman and others, 2016a) (fig. 2)." (Clark et al., 2019).
Mississippi Embayment	Unconsolidated aquifer	"The remainder of **net groundwater outflow** (171 Mgal/d) occurs through the **unconsolidated units** of the **Mississippi embayment** on the eastern margin of the study area (fig. 3*B*), including the McNairy-Nacatoch aquifer of Cretaceous-age and Tertiary-age units." (Clark et al., 2019). " Mississippi embayment remains an important question because of the **high amount of groundwater** use from **alluvial aquifers** and limitations in groundwater-flow models at model boundaries." (Clark et al., 2019).
Basement rock	Endogenous bedrock	"The bottom of the Ozark system is bounded by **metamorphic and igneous rocks of Precambrian age** (**Basement confining unit**) that underlie much of the midwestern aquifers in the central United States (Jorgensen and others, 1993). "The unit exhibits **very low permeability owing** to the **igneous and metamorphic rocks** that compose **the basement complex** (Jorgensen and others, 1993) and therefore was not explicitly modeled for this study." (Clark et al., 2019).

Clark, B.R., Duncan, L.L., Knierim, K.J. (2019). Groundwater availability in the Ozark Plateaus aquifer system: U.S. Geological Survey Professional Paper 1854, 82 p., https://doi.org/10.3133/pp1854

3.65 Pearl and Chattahoochee Aquifer System

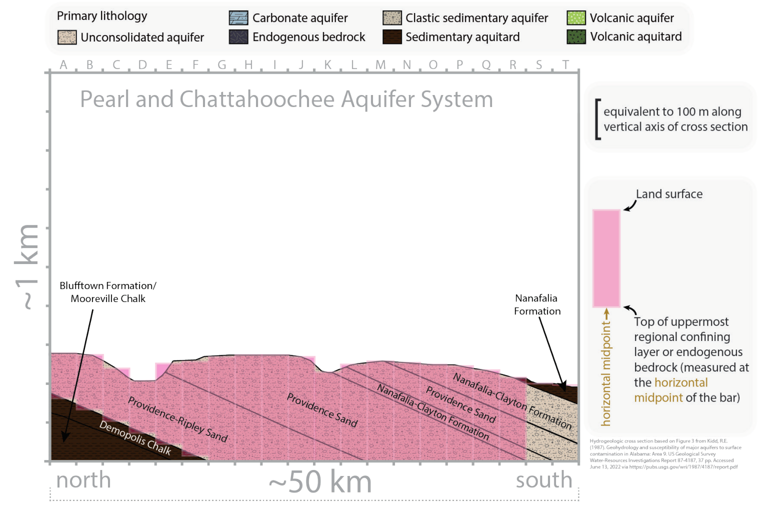

Supplementary Fig. 224. Hydrogeologic cross section. 20 equally spaced transparent pink bars overlie the cross section; each shaded bar depicts the vertical offset from the land surface to the top of the uppermost confining unit or endogenous bedrock.

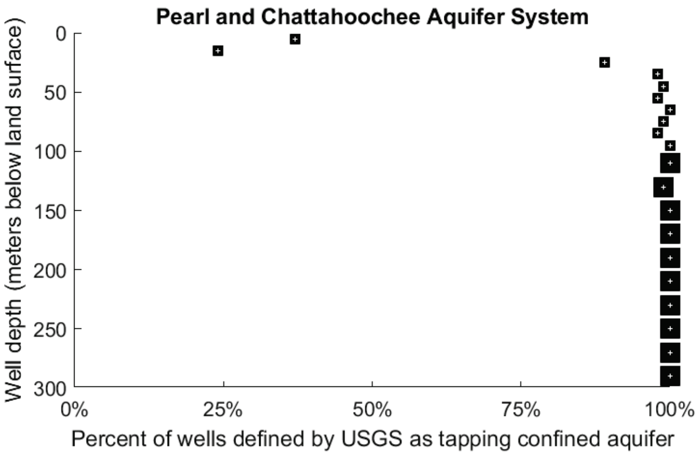

Supplementary Fig. 225. Vertical variations in the prevalence of wells that have been defined as tapping an unconfined or a confined aquifer by the USGS. The smaller squares represent 10 m depth intervals from the land surface to 100 m; the larger squares represent 20 m intervals from 100 m to 300 m below the land surface.

The Pearl and Chattahoochee Aquifer System is located in southwestern Georgia and southeastern Alabama.

(i) A hydrogeologic cross section presented in Fig. 3 by Kidd (1987) depicts a series of dipping sedimentary formations. Note that the cross section presented by Kidd (1987) lacks a quantitative vertical axis; whereas the cross sections we consulted for the other n=73 aquifer systems have quantative vertical axes. We scaled the vertical scale of the cross section by Kidd (1987) by matching the topography depicted in the figure with an elevation profile along the cross section path. We stress that this cross section is more uncertain than others; we did not use the cross section for this study area to estimate the depth to confined conditions (i.e., we use data source (ii) described below).

(ii) We analysed wells within the study area that the USGS has defined as either unconfined or confined. Most (>80%) wells at depths of 20-30 m and at depths exceeding 20 m are defined as tapping a confined aquifer.

Depth to confined conditions: 20-30 meters below land surface (based on (ii) above)

Reference: Kidd, R.E. (1987). Geohydrology and susceptibility of major aquifers to surface contamination in Alabama: Area 9. US Geological Survey Water-Resources Investigations Report 87-4187, 37 pp. Accessed June 13, 2022 via https://pubs.usgs.gov/wri/1987/4187/report.pdf

The table below presents a series of published quotes (see quotation marks denoting text quoted from another publication, which is cited following the quotation marks with the full reference written in full below the table). The leftmost column lists a title of a hydrogeologic formation depicted in the cross section on the previous page. The rightmost column presents a quote from a hydrogeological study (see base of table for citation). The quote has been annotated with colored text to highlight how we categorized each layer (i.e., see categories in the center column in the table). Specifically: (i) blue text highlights portions of a quote that provide insights into the degree of consolidation of the formation, (ii) red text highlights portions of a quote that categorize the formation as an aquifer or an aquitard (i.e., higher versus lower permeability in the context of local hydrogeologic formations), and (iii) green text highlights portions of a quote that provide information about the lithology of the formation.

Supplementary Table 68. Hydrostratigraphy details for the Pearl and Chattahoochee

**Formation name**	**Category**	**Quote**
Nanafalia Formation (Tnf)	Sedimentary **aquitard** (consolidated or semi-consolidated rock)	"**Sand, varicolored, poorly-sorted, clay, grayish-green, silt and gravel**" Table 2 (Kidd, 1987). "The **Nanafalia is not a major aquifer**, but **the basal sand of the formation** is the **uppermost part of the Nanafalia-Clayton aquifer, which is a major aquifer in the study area**." Kidd, 1987).
Clayton Formation (Tc)	Unconsolidated aquifer	"In the outcrop areas, the lower part of the formation consists of **5 to 15 feet of fine- to coarse-grained sand that locally contains gravel, lignite and clay pebbles**. The **base of the bed generally has a thin layer of sandstone**. In the subsurface the basal sand grades upward into **sand overlain by sandy to clayey fossiliferous limestone that may be as much as 25 feet thick**. The **Clayton grades into sandy limestone** in downdip areas. The Clayton Formation is included with the Nanafalia Formation as part of the Nanafalia-Clayton aquifer. **The Nanafalia-Clayton aquifer is considered a major aquifer** in the study area (table 2). The **Clayton will yield about 10 gal/min in most areas south of its outcrop. Yields of 0.5 Mgal/d may be available in the central and southern parts of Pike and Harbour Counties from the limestone**." (Kidd, 1987).
Providence Sand (Kp)	Sedimentary **aquifer** (consolidated or semi-consolidated rock)	"The Providence consists of **fine- to coarse-grained sand that is micaceous and carbonaceous; and laminated to thin-bedded silty clay and massive, lignitic and kaolinitic clay**." (Kidd, 1987). "The **Providence Sand**, the uppermost part of the **Providence-Ripley aquifer**, is a potential source of **0.5 Mgal/d per well** in southern Pike and Harbour Counties (table 2). The **Providence is tapped by wells that supply** the towns of Brundidge, Goshen, Louisville, and Clio; and the South Alabama Electrical Co-op." (Kidd, 1987).
Ripley Formation (Kr)	Sedimentary **aquifer** (consolidated or semi-consolidated rock)	"The Ripley ranges in thickness from 270 to 500 feet. The formation consists of **fine- to coarse-grained sand, carbonaceous clay, sandy clay, and thin beds of gravelly sand and limestone**. The **sand beds** in the Ripley Formation are part of the **Providence-Ripley aquifer that is a major aquifer** in the southern halves of Pike and Barbour Counties where **yields of 0.5 to 1.0 Mgal/d per well** may be obtained (table 2)." (Kidd, 1987).
Demopolis Chalk	Sedimentary **aquitard** (consolidated or	"The Demopolis overlies the Mooreville in the western part of the study area and the Blufftown Formation in central and eastern parts. It consists of **sandy micaceous calcareous clay** that grades eastward into **sandy clay**. The Demopolis is

Formation name	Category	Quote
	semi-consolidated rock)	**relatively impermeable and generally is not a source of water** in the study area." (Kidd, 1987).
Blufftown Formation (Kb)/ Mooreville Chalk (Km)	Sedimentary **aquitard** (consolidated or semi-consolidated rock)	"The Blufftown increases in thickness from about 30 feet in the western part of the study area **to more than 500 feet** in the eastern part. In the eastern part of the study area the lower part of the formation consists of about **200 feet of fine- to coarse-grained sand and sandy clay** and the upper part consists of **200 to 300 feet of calcareous sandy clay** containing some **thin beds of sand and calcareous sandstone**. The Blufftown Formation intertongues with the Mooreville Chalk in Macon, Bullock, and Russell Counties (fig. 3)." (Kidd, 1987). "The **Blufftown** is **not considered a major aquifer**" (Kidd, 1987). "The Mooreville consists of about **500 feet of silty chalk and calcareous clay interbedded with thin layers of limestone and calcareous sandstone**. The **Mooreville Chalk** is **relatively impermeable and is not a source of water** in the study area." (Kidd, 1987).

Kidd, R.E. (1987). Geohydrology and Susceptibility of Major Aquifers to Surface Contamination in Alabama, Area 9. No. 87-4187. Department of the Interior, US Geological Survey, Accessed June 20, 2022 via https://pubs.usgs.gov/wri/1987/4187/report.pdf

3.66 Salinas Valley

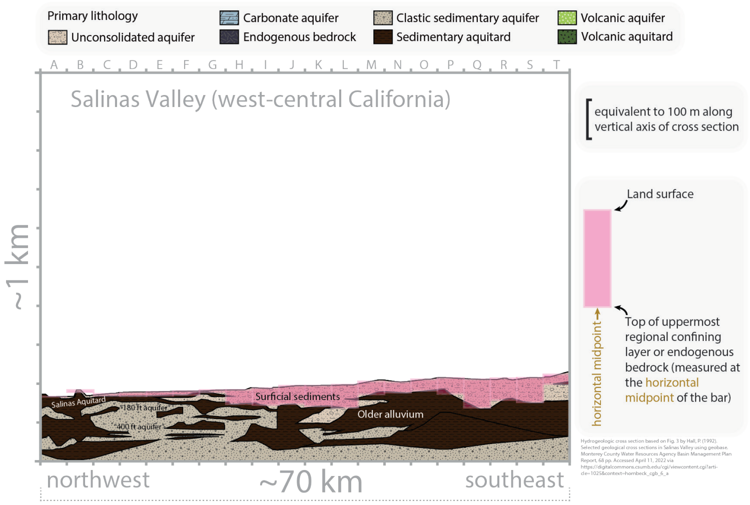

Supplementary Fig. 226. Hydrogeologic cross section. 20 equally spaced transparent pink bars overlie the cross section; each shaded bar depicts the vertical offset from the land surface to the top of the uppermost confining unit or endogenous bedrock.

The Salinas Valley is located in west-central California.

(i) A hydrogeologic cross section presented in Fig. 3 by Hall (1992) demonstrates widespread confining beds (e.g., the Salinas Valley Aquitard in the northwest of the valley). The median depth to the top of the uppermost confined unit is 31 meters (25th-75th percentile range is 9 meters to 49 meters below land surface; see pink transparent bars in cross section to the left).

(ii) USGS well data (defining if the well taps an unconfined or confined aquifer) are insufficient to evaluate the depths at which the aquifer system transitions from unconfined to confined conditions.

(iii) Hall (1992) states, with respect to confining conditions, (quote): *"The blue clay, overlying the 180 foot aquifer, ranges from 25 feet thick at Salinas to more than 100 feet thick at Nashua Road. Is it known as the Salinas Aquitard and is composed mostly of blue marine clays with some silts".* A report by Harding ESE (2001) on the hydrostratigraphy area states that (quote): *"The lateral extent of the 180-Foot Aquifer is generally defined by the overlying* [Salinas Valley Aquitard] *clay, which maintains confined conditions throughout much of the study area."* Vengosh et al. (2002) state that: *"The Salinas Valley has a relatively deep, confined "400-foot" aquifer, overlain by a "180-foot" aquifer and a shallower perched aquifer, all made up of alluvial sand, gravel and clay deposits"*

Depth to confined conditions: 30-40 m based on (i) (i.e., median length of the 20 pink transparent bars overlaid on cross section to the left)

References: Hall, P. (1992). Selected geological cross sections in Salinas Valley using geobase. Monterey County Water Resources Agency Basin Management Plan Report, 68 pp. Accessed April 11, 2022 via https://digitalcommons.csumb.edu/cgi/viewcontent.cgi?article=1025&context=hornbeck_cgb_6_a

Harding ESE (2001). Final Report Hydrogeologic Investigation of the Salinas Valley Basin in the Vicinity of Fort Ord and Marina Salinas Valley, California. Report for the Monterey County Water Resources Agency, 165 pp. Accessed May 20, 2022 via http://svbgsa.org/wp-content/uploads/2020/08/2001-Final-Report-Hydrog.pdf

Vengosh, A., Gill, J., Davisson, M.L., Hudson, B.G. (2002). A multi-isotope (B, Sr, O, H, and C) and age dating (^{3}H–^{3}He and ^{14}C) study of groundwater from Salinas Valley, California: Hydrochemistry, dynamics, and contamination processes. Water Resources Research, 38, 1008.

The table below presents a series of published quotes (see quotation marks denoting text quoted from another publication, which is cited following the quotation marks with the full reference written in full below the table). The leftmost column lists a title of a hydrogeologic formation depicted in the cross section on the previous page. The rightmost column presents a quote from a hydrogeological study (see base of table for citation). The quote has been annotated with colored text to highlight how we categorized each layer (i.e., see categories in the center column in the table). Specifically: (i) blue text highlights portions of a quote that provide insights into the degree of consolidation of the formation, (ii) red text highlights portions of a quote that categorize the formation as an aquifer or an aquitard (i.e., higher versus lower permeability in the context of local hydrogeologic formations), and (iii) green text highlights portions of a quote that provide information about the lithology of the formation.

Supplementary Table 69. Hydrostratigraphy details for the Salinas Valley

Formation name	Category	Quote
Recent Quaternary sediments	Unconfined aquifer	"The **sands and gravels** of this group supply most of the groundwater to the valley" (Hall, 1992). "**Recent sand dunes, stream channel deposits, alluvium, floodplain deposits**" (Hall, 1992). "**low to high well yield**" (Table 1. Hall, 1992).
Older alluvium	Sedimentary **aquitard** (consolidated or semi-consolidated rock)	"It is known as the Salinas aquitard and **is composed mostly of blue marine clays with some silts**. It has low permeability and greatly reduced river seepage and recharge to the 180 foot aquifer. The **clay** pinches out the East-sdie" (Hall, 1992). "**Salinas aquiclude**" (Hall, 1992).
Valley fill materials *(salt water intrusion problem near the coast)*	Clastic sedimentary rock **aquifer** (consolidated or semi-consolidated rock)	"There are **fluvial sands and gravel** associated with old Salinas River channel and also possible delta condition." (Hall, 1992). "**Both the 180 foot aquifer and the upper part of the 400 foot aquifer** may correlate with **Aroma Sands**. The lower part of the 400 foot aquifer may correlate with part of the **Paso Robles formation**." (Hall, 1992).

Hall, P. (1992). Selected geological cross sections in Salinas Valley using geobase. Monterey County Water Resources Agency Basin Management Plan Report, 68 pp. Accessed April 11, 2022 via https://digitalcommons.csumb.edu/cgi/viewcontent.cgi?article=1025&context=hornbeck_cgb_6_a

3.67 Salt Lake Valley

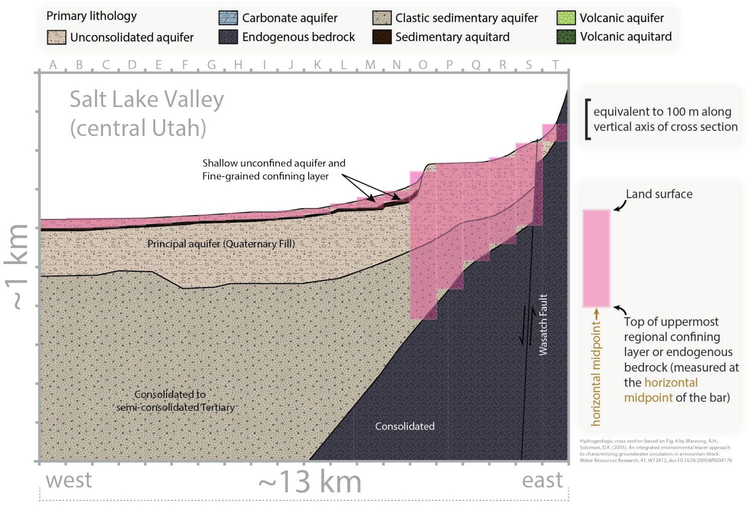

Supplementary Fig. 227. Hydrogeologic cross section. 20 equally spaced transparent pink bars overlie the cross section; each shaded bar depicts the vertical offset from the land surface to the top of the uppermost confining unit or endogenous bedrock.

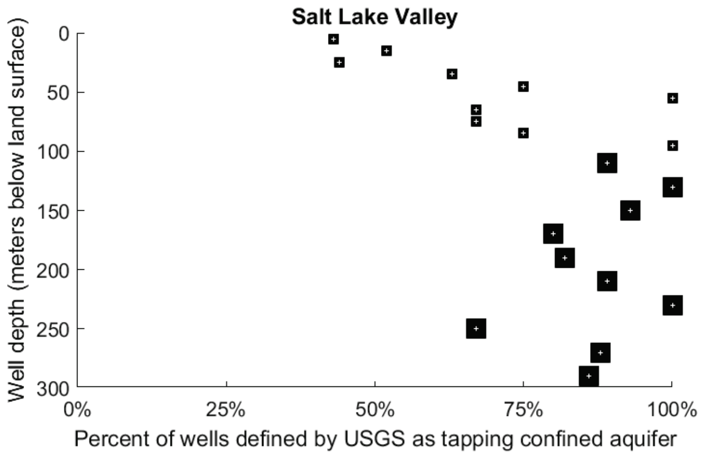

Supplementary Fig. 228. Vertical variations in the prevalence of wells that have been defined as tapping an unconfined or a confined aquifer by the USGS. The smaller squares represent 10 m depth intervals from the land surface to 100 m; the larger squares represent 20 m intervals from 100 m to 300 m below the land surface.

Salt Lake Valley is located in central Utah.

(i) A hydrogeologic cross section presented in Fig. 4 by Manning and Solomon (2005) labels a fine-grained confining layer at shallow depths (<100 m) across much of the study area.

(ii) We analysed wells within the study area that the USGS has defined as either unconfined or confined. Most (>80%) wells at depths of 50-60 m and at depths exceeding 50 m are defined as tapping a confined aquifer.

Depth to confined conditions: 50-60 m (based on (ii) above)

Reference: Manning, A.H., Solomon, D.K. (2005). An integrated environmental tracer approach to characterizing groundwater circulation in a mountain block. Water Resources Research, 41, W12412, doi:10.1029/2005WR004178

The table below presents a series of published quotes (see quotation marks denoting text quoted from another publication, which is cited following the quotation marks with the full reference written in full below the table). The leftmost column lists a title of a hydrogeologic formation depicted in the cross section on the previous page. The rightmost column presents a quote from a hydrogeological study (see base of table for citation). The quote has been annotated with colored text to highlight how we categorized each layer (i.e., see categories in the center column in the table). Specifically: (i) blue text highlights portions of a quote that provide insights into the degree of consolidation of the formation, (ii) red text highlights portions of a quote that categorize the formation as an aquifer or an aquitard (i.e., higher versus lower permeability in the context of local hydrogeologic formations), and (iii) green text highlights portions of a quote that provide information about the lithology of the formation.

Supplementary Table 70. Hydrostratigraphy details for the Salt Lake Valley

Formation name	Category	Quote
Shallow Unconfined aquifer and Fine-grained Confining Layer *(this aquifer has thin layer of confining unit)*	Unconsolidated aquifer *(*underlain by fine grained layer)*	"Where it is confined, the principal aquifer is overlain by a **shallow unconfined aquifer** typically <30 m thick." (Manning & Solomon, 2005). "the principal aquifer is overlain by a layer of **fine-grained sediments that act as a confining layer**." (Manning & Solomon, 2005). "The **unconsolidated sediments** of Quaternary age were deposited mainly as **alluvial fans, by streams, and as deltas and other lacustrine features associated with Lake Bonneville and older paleolakes** that once covered the valley. The **hydraulic conductivity of coarser grained deposits is estimated to be about 200 ft/d, compared to a value of about 1 ft/d for shallow lake-deposited clays** (Lambert, 1995, p. 14)." (Thiros et al., 2010)
Principal aquifer (Quaternary Fill)	Unconsolidated aquifer	"**Production wells are screened within the principal aquifer**, the **deeper Quaternary sediments** composed of **sand and gravel interbedded with lenses of silt and clay**." (Manning & Solomon, 2005).
Consolidated to semi-consolidated Tertiary	Clastic sedimentary **aquifer** (consolidated or semi-consolidated rock)	"Quaternary sediments are 100–300 m thick and **Tertiary sediments are 200–800 m thick throughout most of the basin**." (Manning & Solomon, 2005). "The **Tertiary-age sediments** that crop out along the western and southern margins of the valley were deposited mainly as **alluvial fans, in lakes, and as volcanic ash** and are estimated to have a **hydraulic conductivity of about 1 ft/d** (Lambert, 1995, p. 15)" (Thiros et al., 2010)
Consolidated *(Intrusive, Precambrian and sedimentary rock can be found)*	Endogenous bedrock	"The geology of the **Wasatch mountain block** varies considerably within the study area [Bryant, 1990] and has been summarized by Manning and Solomon [2004]. **Dominant rock types include granitic intrusive rocks, quartzites interbedded with shales, and mixed sedimentary rocks**." (Manning & Solomon, 2005). "The transmissivity of these rocks is dependent on the presence or absence of **fractures and is highly variable**. Hely and others (1971, plate 1) characterized the volcanic rocks as "**rocks of lowest permeability**." (Thiros et al., 2010)

Manning, A.H., Solomon, D.K. (2005). An integrated environmental tracer approach to characterizing groundwater circulation in a mountain block. *Water Resources Research*, 41(12).

Thiros, S. A., Bexfield, L. M., Anning, D. W., & Huntington, J. M. (2010). *Conceptual understanding and groundwater quality of selected basin-fill aquifers in the southwestern United States* (No. 1781). US Geological Survey. Accessed June 3, 2022 via https://pubs.usgs.gov/pp/1781/

3.68 San Pedro Basin

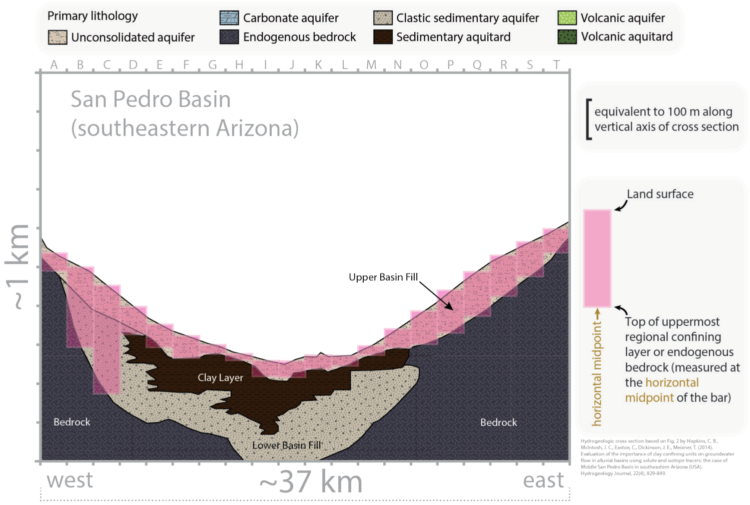

Supplementary Fig. 229. Hydrogeologic cross section. 20 equally spaced transparent pink bars overlie the cross section; each shaded bar depicts the vertical offset from the land surface to the top of the uppermost confining unit or endogenous bedrock.

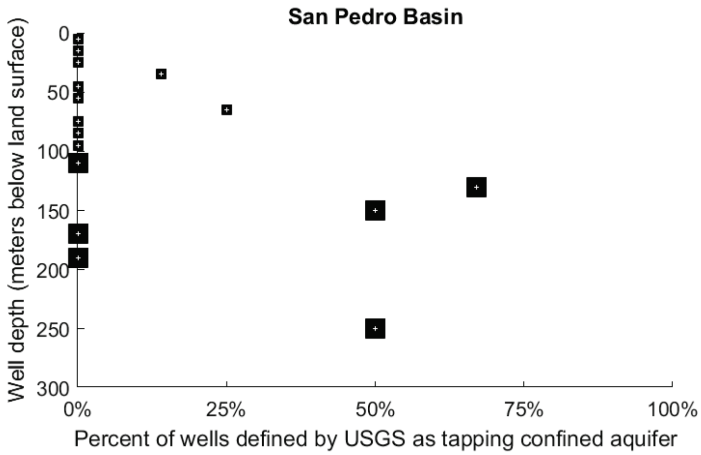

Supplementary Fig. 230. Vertical variations in the prevalence of wells that have been defined as tapping an unconfined or a confined aquifer by the USGS. The smaller squares represent 10 m depth intervals from the land surface to 100 m; the larger squares represent 20 m intervals from 100 m to 300 m below the land surface.

The San Pedro Basin is located in southeastern Arizona.

(i) A hydrogeologic cross section presented in Fig. 2 by Hopkins et al. (2014) depicts a clay layer in the central portion of the basin.

(ii) We analysed wells within the study area that the USGS has defined as either unconfined or confined. Most (>80%) wells at depths of 300-320 m and at depths exceeding 300 m are defined as tapping a confined aquifer.

Depth to confined conditions: 300-320 m (based on (ii) above)

Reference: Hopkins, C. B., McIntosh, J. C., Eastoe, C., Dickinson, J. E., Meixner, T. (2014). Evaluation of the importance of clay confining units on groundwater flow in alluvial basins using solute and isotope tracers: the case of Middle San Pedro Basin in southeastern Arizona (USA). Hydrogeology Journal, 22, 829-849.

The table below presents a series of published quotes (see quotation marks denoting text quoted from another publication, which is cited following the quotation marks with the full reference written in full below the table). The leftmost column lists a title of a hydrogeologic formation depicted in the cross section on the previous page. The rightmost column presents a quote from a hydrogeological study (see base of table for citation). The quote has been annotated with colored text to highlight how we categorized each layer (i.e., see categories in the center column in the table). Specifically: (i) blue text highlights portions of a quote that provide insights into the degree of consolidation of the formation, (ii) red text highlights portions of a quote that categorize the formation as an aquifer or an aquitard (i.e., higher versus lower permeability in the context of local hydrogeologic formations), and (iii) green text highlights portions of a quote that provide information about the lithology of the formation.

Supplementary Table 71. Hydrostratigraphy details for the San Pedro Basin

Formation name	Category	Quote
Upper Basin Fill	Unconsolidated aquifer	"**coarse granular fraction of sedimentary basin fill represented** by **gravels and sands**. It corresponds to the **more hydraulically conductiv**e portions of the Upper- and Lower-Basin Fill." (Callegary et al., 2016).
Clay Layer	Clastic sedimentary **aquitard** (consolidated or semi-consolidated rock)	"the **fine sediments with low hydraulic conductivity** that mainly comprise the upper basin fill. These **low-conductivity silts and clays** occur mainly in the central portion of the basin." (Callegary et al., 2016).
Lower Basin Fill	Clastic sedimentary **aquifer** (consolidated or semi-consolidated rock)	"**fractured-rock aquifers**, among which are the **conglomeratic units of the Báucarit Formation, the Tc unit (See Chapter 4), the Tertiary felsic volcanic rocks that lie between these, and the fractured or weathered portions of the basement, such as limestone**, that could possibly contain **groundwater**." (Callegary et al., 2016).
Bedrock	Endogenous bedrock *(intense fracturing of the basement)*	"the oldest rocks form a **Precambrian basement characterized by the Pinal Schist (1680 million years before present (Ma)) and mesoproterozoic granitic intrusions**" (Callegary et al., 2016). "**Hydrostratigraphic Basement**" (Callegary et al., 2016)

Callegary, J.B., Minjárez Sosa, I., Tapia Villaseñor, E.M., dos Santos, P., Monreal Saavedra, R., Grijalva Noriega, F.J., Huth, A.K., Gray, F., Scott, C.A., Megdal, S.B., Oroz Ramos, L.A., Rangel Medina, M., Leenhouts, J.M. (2016). San Pedro River Aquifer Binational Report: International Boundary and Water Commission, 170 pp. Accessed November 29, 2021 from https://ibwc.gov/Files/San_Pedro_River_Binational%20Report_013116.pdf

3.69 Santa Clara-Calleguas Basin

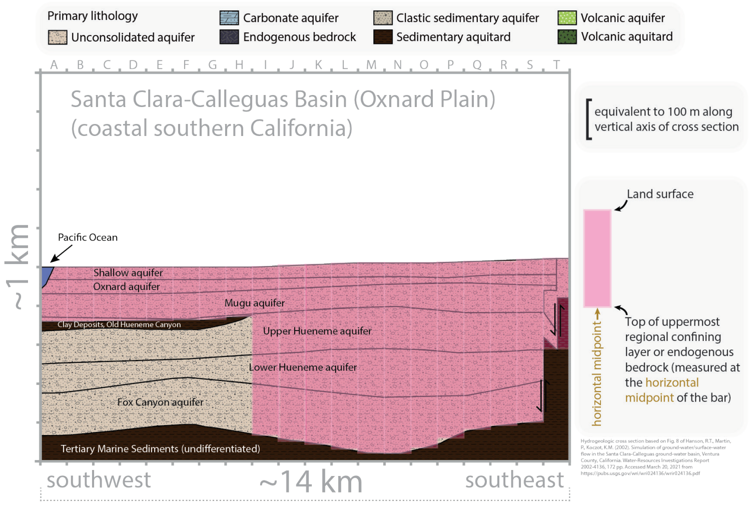

Supplementary Fig. 231. Hydrogeologic cross section. 20 equally spaced transparent pink bars overlie the cross section; each shaded bar depicts the vertical offset from the land surface to the top of the uppermost confining unit or endogenous bedrock.

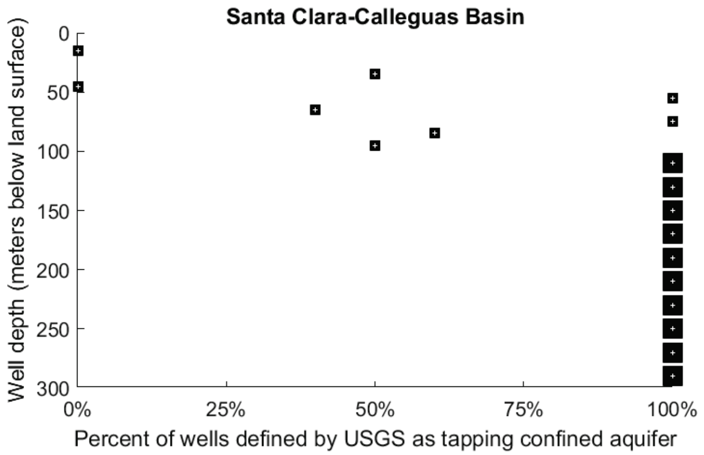

Supplementary Fig. 232. Vertical variations in the prevalence of wells that have been defined as tapping an unconfined or a confined aquifer by the USGS. The smaller squares represent 10 m depth intervals from the land surface to 100 m; the larger squares represent 20 m intervals from 100 m to 300 m below the land surface.

The Santa Clara-Calleguas Basin (Oxnard Plain) is located in coastal southern California.

(i) A hydrogeologic cross section presented in Fig. 8 by Hanson et al. (2002) depicts a multi-layered aquifer system with a confining unit relatively close to the coast.

(ii) We analysed wells within the study area that the USGS has defined as either unconfined or confined. Most (>80%) wells at depths of 50-60 m and at depths exceeding 50 m are defined as tapping a confined aquifer.

Depth to confined conditions: 50-60 m (based on (ii) above)

Reference: Hanson, R.T., Martin, P., Koczot, K.M. (2002). Simulation of ground-water/surface-water flow in the Santa Clara-Calleguas ground-water basin, Ventura County, California. Water-Resources Investigations Report 2002-4136, 172 pp. Accessed March 20, 2021 from https://pubs.usgs.gov/wri/wri024136/wrir024136.pdf

The table below presents a series of published quotes (see quotation marks denoting text quoted from another publication, which is cited following the quotation marks with the full reference written in full below the table). The leftmost column lists a title of a hydrogeologic formation depicted in the cross section on the previous page. The rightmost column presents a quote from a hydrogeological study (see base of table for citation). The quote has been annotated with colored text to highlight how we categorized each layer (i.e., see categories in the center column in the table). Specifically: (i) blue text highlights portions of a quote that provide insights into the degree of consolidation of the formation, (ii) red text highlights portions of a quote that categorize the formation as an aquifer or an aquitard (i.e., higher versus lower permeability in the context of local hydrogeologic formations), and (iii) green text highlights portions of a quote that provide information about the lithology of the formation.

Supplementary Table 72. Hydrostratigraphy details for the Santa Clara-Calleguas Basin

Formation name	Category	Quote
Shallow aquifer	Unconsolidated aquifer	"The **unconsolidated deposits of the late Pleistocene and Holocene epochs** are grouped into the regional **upper-aquifer system**, which includes the **Shallow**, Oxnard, and Mugu **aquifers** (fig. 7B)." (Hanson et al., 2002). "Along the flood plain of the Santa Clara River, **the shallow aquifer consists of predominantly sand and gravel** and is **an important source of ground water**." (Hanson et al., 2002).
Oxnard aquifer	Unconsolidated aquifer	"The **unconsolidated deposits of the late Pleistocene and Holocene epochs** are grouped into the regional **upper-aquifer system**, which includes the Shallow, **Oxnard**, and Mugu **aquifers** (fig. 7B)." (Hanson et al., 2002). "**The Oxnard aquifer** lies at the **base of the Holocene deposits and consists of sand and gravel deposited by the ancestral Santa Clara River and the Calleguas Creek and by their major tributaries**." (Hanson et al., 2002).
Mugu aquifer	Unconsolidated aquifer	"The **unconsolidated deposits of the late Pleistocene and Holocene epochs** are grouped into the regional **upper-aquifer system**, which includes the Shallow, Oxnard, and **Mugu aquifers** (fig. 7B)." (Hanson et al., 2002). "Throughout most of the ground-water basin, **the Mugu aquifer** extends from **about 200 to 400 ft below land surface (fig. 8) and consists of sand and gravel interbedded with silt and clay**." (Hanson et al., 2002).
Upper Hueneme aquifer *(Hueneme aquifer classification is based on electric log data)*	Unconsolidated aquifer	"The **lower-aquifer system** is composed of **complexly faulted and folded unconsolidated deposits of the Pliocene and Pleistocene epochs** and include the **upper** and lower **Hueneme**, Fox Canyon, and Grimes Canyon aquifers (fig. 7B)." (Hanson et al., 2002). "**These aquifers** consist of **lenticular layers of sand, gravel, silt, and clay**. The **sediments constituting the aquifers have been subjected to considerable folding, faulting, and erosion since deposition**." (Hanson et al., 2002).
Lower Hueneme aquifer *(more fine-grained deposits in the Lower than upper Hueneme aquifer)*	Unconsolidated aquifer	"The **lower-aquifer system** is composed of **complexly faulted and folded unconsolidated deposits of the Pliocene and Pleistocene epochs** and include the upper and **lower Hueneme**, Fox Canyon, and Grimes Canyon aquifers (fig. 7B)." (Hanson et al., 2002). "**These aquifers** consist of **lenticular layers of sand, gravel, silt, and clay**. The **sediments constituting the aquifers have been subjected to considerable folding, faulting, and erosion since deposition**." (Hanson et al., 2002).

Formation name	Category	Quote
Fox Canyon aquifer	Unconsolidated aquifer	"The **lower-aquifer system** is composed of **complexly faulted and folded unconsolidated deposits of the Pliocene and Pleistocene epochs** and include the upper and lower Hueneme, **Fox Canyon**, and Grimes Canyon aquifers (fig. 7B)." (Hanson et al., 2002). "**The aquifer** consists of **weakly indurated very fine- to medium-grained fossiliferous sand with occasional gravel and clay layers of shallow marine origin**." (Hanson et al., 2002).
Undifferentiated or non-water bearing rocks. *(Includes Grime Canyon aquifers)*	Sedimentary rock **aquitard** (consolidated or semi-consolidated rock)	"**The Santa Barbara Formation** (Weber and others, 1976), **which consists of non-water-bearing marine sandstone, siltstone, mudstone, and shale**, underlies the Fox Canyon aquifer throughout most of the ground-water basin and is considered the base of the ground-water system throughout most of the basin." (Hanson et al., 2002).

Hanson, R.T., Martin, P., Koczot, K.M. (2002). Simulation of ground-water/surface-water flow in the Santa Clara-Calleguas ground-water basin, Ventura County, California. Water-Resources Investigations Report 2002-4136, 172 pp. Accessed June 1, 2022 from https://pubs.usgs.gov/wri/wri024136/wrir024136.pdf

3.70 Santa Rosa Valley

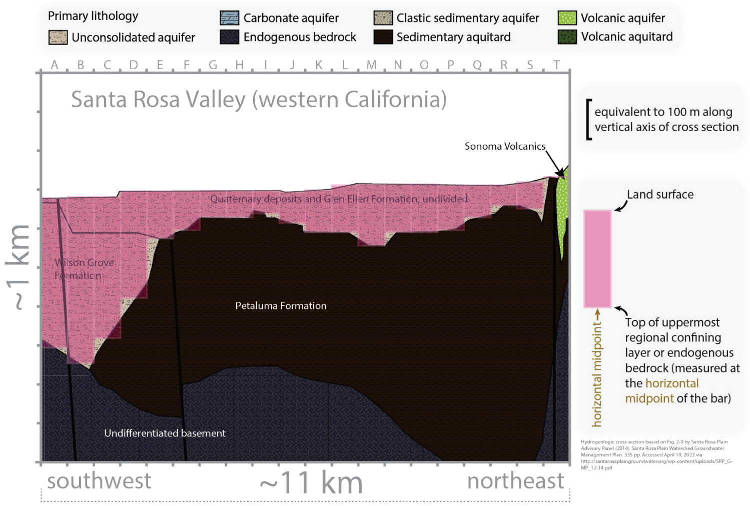

Supplementary Fig. 233. Hydrogeologic cross section. 20 equally spaced transparent pink bars overlie the cross section; each shaded bar depicts the vertical offset from the land surface to the top of the uppermost confining unit or endogenous bedrock.

The Santa Rosa Valley is located in western California.

(i) A hydrogeologic cross section presented in Fig. 2-9 by Santa Rosa Plain Advisory Panel (2014) suggests that the aquifer system does not contain a clear confining unit. The median depth to undifferentiated basement rock is 503 m (see transparent pink shaded bars on cross section; the 25th-75th percentile range of the depth to basement rock is 462-630 meters below land surface). However, the median depth to the top of the Petaluma Formation—which is depicted as low-permeability by Woolfenden and Nishikawa (2014) in their Fig. 5—is 111 m (25th-75th percentile range of the depth to the top of the Petaluma Formation is 68 to 132 meters below land surface).

(ii) There are no USGS wells within the study area that have been defined as tapping an aquifer that is either unconfined or confined.

(iii) Santa Rosa Plain Advisory Panel (2014) state that (following text quoted directly): "*In most parts of the study area, shallow groundwater flow is unconfined, but, at depth, groundwater flow is confined.*" Further, Woolfenden and Nishikawa (2014) states (quote): *"The Petaluma Formation is dominated by fine-grained materials, either in thick beds or as interstitial material in poorly sorted silty and clayey sands or gravels."* (see also their conceptual model in Fig. 5 by Woolfenden and Nishikawa (2014) depicting the Petuluma Formation as a low-permeability formation).

Depth to confined conditions: 100-120 meters below land surface (on the basis of (i))

References: Santa Rosa Plain Advisory Panel (2014). Santa Rosa Plain Watershed Groundwater Management Plan. 335 pp. Accessed April 10, 2022 via http://santarosaplaingroundwater.org/wp-content/uploads/SRP_GMP_12-14.pdf

Woolfenden, L.R., Nishikawa, T. (2014). Simulation of groundwater and surface-water resources of the Santa Rosa Plain watershed, Sonoma County, California. US Geological Survey Scientific Investigations Report 2014–5052, 292 pp. Accessed May 19, 2022 via https://pubs.usgs.gov/sir/2014/5052/pdf/sir2014-5052.pdf

The table below presents a series of published quotes (see quotation marks denoting text quoted from another publication, which is cited following the quotation marks with the full reference written in full below the table). The leftmost column lists a title of a hydrogeologic formation depicted in the cross section on the previous page. The rightmost column presents a quote from a hydrogeological study (see base of table for citation). The quote has been annotated with colored text to highlight how we categorized each layer (i.e., see categories in the center column in the table). Specifically: (i) blue text highlights portions of a quote that provide insights into the degree of consolidation of the formation, (ii) red text highlights portions of a quote that categorize the formation as an aquifer or an aquitard (i.e., higher versus lower permeability in the context of local hydrogeologic formations), and (iii) green text highlights portions of a quote that provide information about the lithology of the formation.

Supplementary Table 73. Hydrostratigraphy details for the Santa Rosa Plain

Formation name	Category	Quote
Quaternary deposits and Glen Ellen Formation, undivided	Unconsolidated aquifer	"Overlying the basement rocks are five geologic units of Cenozoic age that **form the SRP's primary aquifers**. These are: **(1) Quaternary Alluvium, (2) Glen Ellen Formation,** (3) Wilson Grove Formation, (4) Petaluma Formation and (5) Sonoma Volcanics." (Santa Rosa Plain Advisory Panel 2014). "These deposits are dominated by **alluvial fan sediment deposits**, which are materials eroded from rock exposed in the flanking hills. **The deposits generally consist of mixed poorly- to well-sorted sand, silt, clay, gravel, cobbles and boulders, as interfingering, variably thin or thick beds of limited lateral extent (tens to hundreds of feet).**" (Santa Rosa Plain Advisory Panel 2014). The Glen Ellen Formation consists of **clay-rich stratified stream deposits** of **poorly sorted sand, silt, and gravel** (Table 2-4). **Beds of these sediments vary from coarseto fine-grained, commonly over distances of a few tens to a few hundreds of feet, both laterally and vertically**. (Santa Rosa Plain Advisory Panel 2014)
Wilson Grove Formation	Unconsolidated aquifer	"**The Wilson Grove Formation is relatively thick (300 ft to greater than 1000 ft thick), and mostly composed of weakly cemented marine-deposited sandstone, with volcanic ash intervals**. The predominance of relatively **clean sand and the low degree of cementation** in the Wilson Grove Formation result in **moderate to high permeability**. **Well production in the Wilson Grove Formation is high: from 200 to 1,000 gpm or more**." (Santa Rosa Plain Advisory Panel 2014).
Sonoma Volcanics	Volcanic aquifer	"These rocks comprise **a highly variable assemblage of andesitic and basaltic tuffs with interbedded lava flows and explosive volcaniclastic rocks**, having a **broad range of water-bearing properties**." (Santa Rosa Plain Advisory Panel 2014). "**Water production from wells drilled into thick air-fall pumice units may exceed a few hundred gpm, but wells drawing from unfractured lavas or welded tuffs may produce less than 10 gpm and dry holes are encountered occasionally**." (Santa Rosa Plain Advisory Panel 2014)
Petaluma Formation	Clastic sedimentary **aquitard** (consolidated or	"The Petaluma Formation is dominated by **more or less consolidated silt or clay-rich mudstone, with local beds and lenses of poorly-sorted sandstone and minor conglomerate beds**. Due to the large amount of silt- and

Formation name	Category	Quote
	semi-consolidated rock)	clay-sized particles, the specific yields of wells are low, varying from 3 to 7 percent. **Domestic wells drilled into the Petaluma Formation yield on average about 20 gpm and vary from 10 to 50 gpm**." (Santa Rosa Plain Advisory Panel 2014).
Undifferentiated basement	Endogenous bedrock	"**Sandstone, greywacke, chert, serpentine**." (Santa Rosa Plain Advisory Panel 2014). "**Basement rock**". (Santa Rosa Plain Advisory Panel 2014). "**While the basement rocks provide a viable, sole source supply for many households, they are not considered a major water supply source in the SRP groundwater subbasin**.". (Santa Rosa Plain Advisory Panel 2014).

Santa Rosa Plain Advisory Panel (2014). Santa Rosa Plain Watershed Groundwater Management Plan. 335 pp. Accessed 5/17/22 (http://santarosaplaingroundwater.org/wp-content/uploads/SRP_GMP_12-14.pdf)

3.71 South Park Basin

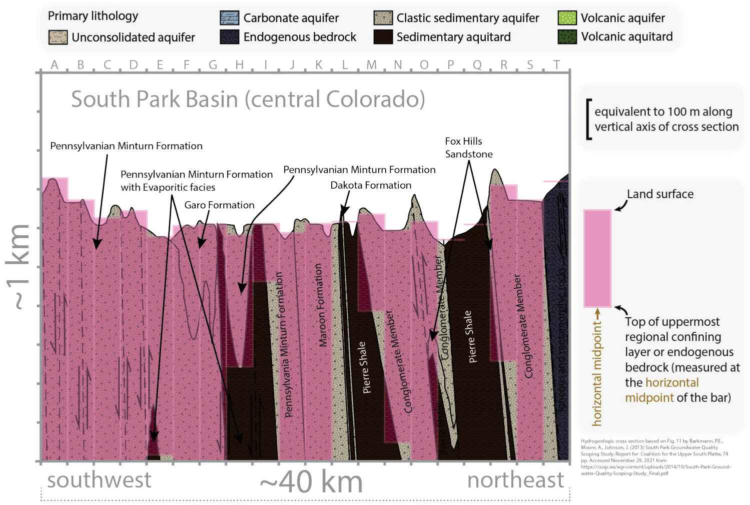

Supplementary Fig. 234. Hydrogeologic cross section. 20 equally spaced transparent pink bars overlie the cross section; each shaded bar depicts the vertical offset from the land surface to the top of the uppermost confining unit or endogenous bedrock.

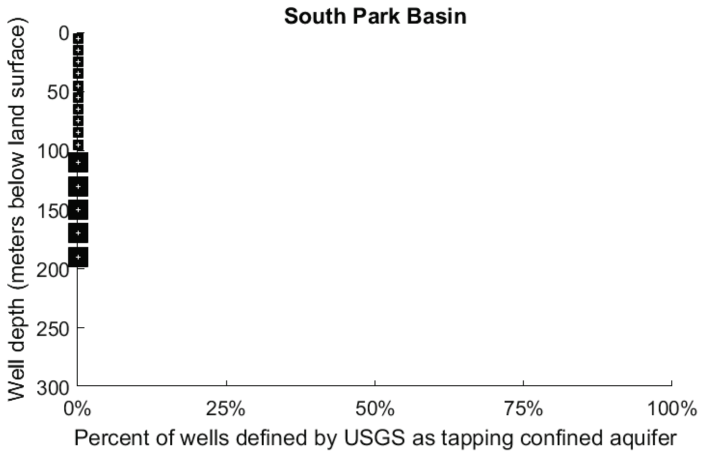

Supplementary Fig. 235. Vertical variations in the prevalence of wells that have been defined as tapping an unconfined or a confined aquifer by the USGS. The smaller squares represent 10 m depth intervals from the land surface to 100 m; the larger squares represent 20 m intervals from 100 m to 300 m below the land surface.

The South Park Basin is located in central Colorado.

(i) A hydrogeologic cross section presented in Fig. 11 by Barkmann et al. (2013) demonstrates the complexity of the sedimentary rock sequences in the South Park Basin. Among the 20 evenly spaced pink transparent bars (the length of each representing the depth to the uppermost confining unit), the median depth to a confining unit is >576 meters below land surface (25th-75th percentile range: 224 m to >619 m).

(ii) We analysed wells within the study area that the USGS has defined as either unconfined or confined. All wells (n=52) in the South Park Basin (depths of 0.15 m to 183 m) are classified as unconfined.

Depth to confined conditions: >576 m (based on (i), consistent with data presented in (ii))

References: Barkmann, P.E., Moore, A., Johnson, J. (2013) South Park Groundwater Quality Scoping Study. Report for Coalition for the Upper South Platte, 74 pp. Accessed November 29, 2021 from https://cusp.ws/wp-content/uploads/2014/10/South-Park-Groundwater-Quality-Scoping-Study_Final.pdf

The table below presents a series of published quotes (see quotation marks denoting text quoted from another publication, which is cited following the quotation marks with the full reference written in full below the table). The leftmost column lists a title of a hydrogeologic formation depicted in the cross section on the previous page. The rightmost column presents a quote from a hydrogeological study (see base of table for citation). The quote has been annotated with colored text to highlight how we categorized each layer (i.e., see categories in the center column in the table). Specifically: (i) blue text highlights portions of a quote that provide insights into the degree of consolidation of the formation, (ii) red text highlights portions of a quote that categorize the formation as an aquifer or an aquitard (i.e., higher versus lower permeability in the context of local hydrogeologic formations), and (iii) green text highlights portions of a quote that provide information about the lithology of the formation.

Supplementary Table 74. Hydrostratigraphy details for the South Park Basin

**Formation name**	**Category**	**Quote**
Conglomerate Member	Clastic sedimentary **aquifer** (consolidated or semi-consolidated rock)	"The Paleocene conglomerate member (informal name) is next and contains **clasts of the older volcanic material mixed with clasts of limited Precambrian basement, Paleozoic rocks, and Cretaceous intrusives indicating a source from the Sawatch uplift to the west** (Widmann and others, 2005; Kirkham and others, 2006)." (Barkmann et al., 2013). "**lenticular conglomerate, sandstone, siltstone and mudstone**" (Donegan, 2018). "**Lower South Park Aquifer**" (Donegan, 2018).
Laramie Formation	Sedimentary **aquitard** (consolidated or semi-consolidated rock) *(some may form Laramie-Fox Hills Aquifer)*	"Laramide hydrogeologic units consist primarily of various sedimentary members that collectively comprise the South Park aquifer and **its confining units**." (Barkmann et al., 2013). "**shale, sandstone, and coal**" (Donegan, 2018). "**confining unit**" (Donegan, 2018).
Fox Hills Sandstone	Clastic sedimentary **aquifer** (consolidated or semi-consolidated rock)	"The Fox Hills **Sandstone** is in turn overlain by, and interfingers with, non-marine Laramie Formation. This upper unit of the group consists of overbank **shale interbedded with lenticular beds of sandstone** deposited on a low-relief coastal plain following the retreat of the Interior Seaway." (Barkmann et al., 2013). (Barkmann et al., 2013). "**Potentially vulnerable aquifers** within Cretaceous seaway hydrogeologic units include the Dakota and **Laramie-Fox Hills aquifers**" (Barkmann et al., 2013).
Pierre Shale	Sedimentary **aquitard** (consolidated or semi-consolidated rock)	"**shale, sandstone, bentonitic layers**" (Donegan, 2018). "**confining unit**, but sand beds and fractures can be a local aquifer" (Donegan, 2018).
Dakota Formation	Clastic sedimentary **aquifer** (consolidated or semi-consolidated rock)	"Conformably above the Morrison is the Cretaceous Dakota Formation which consists of **sandstone, pebble conglomerate, and non-calcareous** shale (Scarbrough, 2001) **deposited** along the shoreline of the advancing Interior Seaway." (Barkmann et al., 2013). "**Potentially vulnerable aquifers** within Cretaceous seaway hydrogeologic units include **the Dakota** and Laramie-Fox Hills **aquifers**" (Barkmann et al., 2013).
Morrison Formation	Sedimentary **aquitard** (consolidated or	"The Morrison Formation consists of **interbedded shale, sandstone, claystone, and basal limestone** (Widmann and

Formation name	Category	Quote
	semi-consolidated rock)	others, 2005).” (Barkmann et al., 2013). “**confining unit, can yield water in porous zones**” (Donegan, 2018).
Garo Formation	Clastic sedimentary **aquifer** (consolidated or semi-consolidated rock) *(Maroon Fm. Unit, variable, with porous zones interbeded with confining shales)*	“The Garo Formation consists of **calcareous sandstone and conglomerate that may conformably** overly the Maroon Formation (De Voto, 1971; Widmann and others, 2005).” (Barkmann et al., 2013). “Paleozoic **hydrogeologic units** in South Park include water-yielding Cambrian through Mississippian rocks, the Minturn-Maroon aquifer, and **the Garo aquifer**” (Barkmann et al., 2013).
Maroon Formation	Clastic sedimentary **aquifer** (consolidated or semi-consolidated rock) *(Maroon Fm. Unit, variable, with porous zones interbeded with confining shales)*	“The Maroon Formation overlies and is gradational with the Minturn Formation. It closely **resembles the Minturn Formation** in composition but **has a redder color and contains less limestone**. The change reflects a transition away from a marine-dominated clastic wedge to a sub-aerial fluvial clastic wedge (De Voto, 1971; Kirkham and others, 2006).” (Barkmann et al., 2013). “Paleozoic **hydrogeologic units in South Park include water-yielding** Cambrian through Mississippian rocks, the **Minturn-Maroon aquifer**, and the Garo aquifer” (Barkmann et al., 2013).
Pennsylvanian Minturn Formation	Clastic sedimentary **aquifer** (consolidated or semi-consolidated rock) *(Minturn Fm. Unit, variable, with porous zones interbeded with confining shales)*	“It **grades upward into the coarser-grained** Minturn Formation which contains **interbedded pebble to cobble conglomerate, sandstone, siltstone, and limestone**, attesting to intensified tectonic activity of the Anazasi uplifts.” (Barkmann et al., 2013). “Paleozoic **hydrogeologic units** in South Park include water-yielding Cambrian through Mississippian rocks, the **Minturn-Maroon aquifer,** and the Garo aquifer” (Barkmann et al., 2013).
Pennsylvanian Minturn Formation with Evaporitic facies	Sedimentary rock **aquitard** (consolidated or semi-consolidated rock) *(water quality issue when groundwater occurred)*	“The **Minturn Formation** also includes **an evaporitic facies** that contains **thick beds** of **salt and gypsum** suggesting **restricted circulation and high evaporation rates** within the subsiding basin (Kirkham and others, 2007)” (Barkmann et al., 2013).
Igneous and metamorphic rocks	Endogenous bedrock	“The basin consists of eastward-dipping Paleozoic and Mesozoic sedimentary rocks preserved between uplifts of **Precambrian igneous and metamorphic rocks** on either side” (Barkmann et al., 2013). “**Groundwater** in **Precambrian igneous and metamorphic rocks** generally is upgradient from oil and gas sites” (Barkmann et al., 2013).

Donegan, K.C. (2018). Groundwater levels in the South Park Basin 2018. Colorado Division of Water Resources Report, 29 pp. Accessed November 29, 2021 from https://dnrweblink.state.co.us/dwr/ElectronicFile.aspx?docid=3351305&dbid=0

Barkmann, P.E., Moore, A., Johnson, J. (2013) South Park Groundwater Quality Scoping Study. Report for Coalition for the Upper South Platte, 74 pp. Accessed November 29, 2021 from https://cusp.ws/wp-content/uploads/2014/10/South-Park-Groundwater-Quality-Scoping-Study_Final.pdf

3.72 Tijuana-San Diego Basin

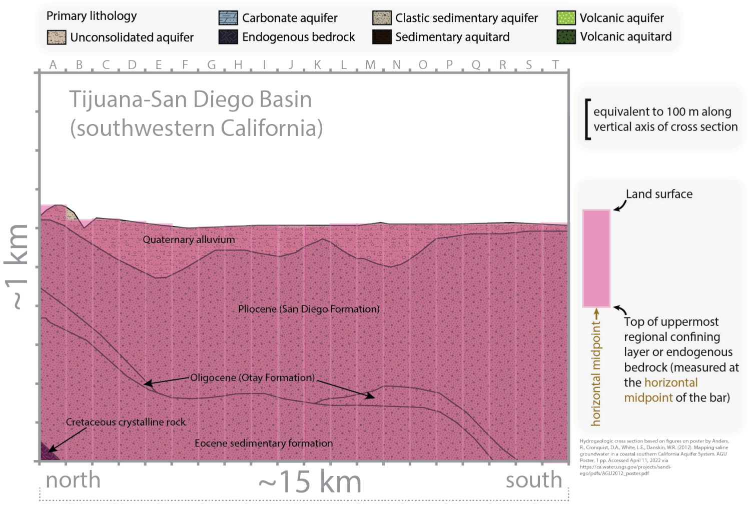

Supplementary Fig. 236. Hydrogeologic cross section. 20 equally spaced transparent pink bars overlie the cross section; each shaded bar depicts the vertical offset from the land surface to the top of the uppermost confining unit or endogenous bedrock.

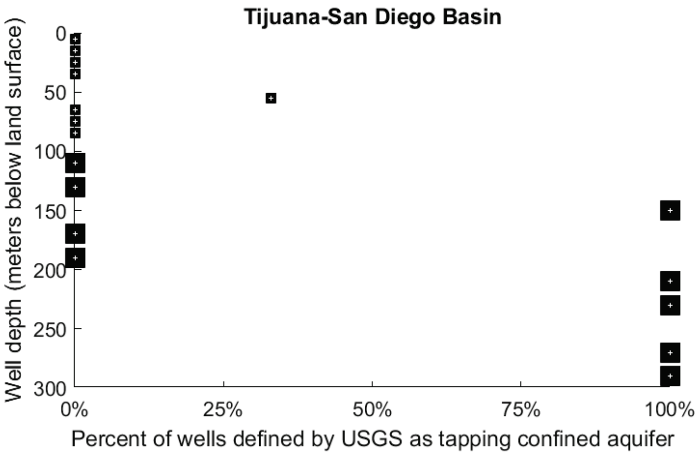

Supplementary Fig. 237. Vertical variations in the prevalence of wells that have been defined as tapping an unconfined or a confined aquifer by the USGS. The smaller squares represent 10 m depth intervals from the land surface to 100 m; the larger squares represent 20 m intervals from 100 m to 300 m below the land surface.

The Tijuana-San Diego Basin is located in northwestern Baja California (Mexico) and southwestern California (US).

(i) A hydrogeologic cross section presented in a poster by Anders et al. (2012) depicts a series of sedimentary sequences including shallow Quaternary-aged alluvium underlain by Pliocene- to Eocene-aged formations.

(ii) We analysed wells within the study area that the USGS has defined as either unconfined or confined. Most (>80%) wells at depths of 140-160 m and at depths exceeding 140 m are defined as tapping a confined aquifer.

Depth to confined conditions: 140-160 m (based on (ii) above)

Reference: Anders, R., Cronquist, D.A., White, L.E., Danskin, W.R. (2012). Mapping saline groundwater in a coastal southern California Aquifer System. AGU Poster, 1 pp. Accessed April 11, 2022 via https://ca.water.usgs.gov/projects/sandiego/pdfs/AGU2012_poster.pdf

The table below presents a series of published quotes (see quotation marks denoting text quoted from another publication, which is cited following the quotation marks with the full reference written in full below the table). The leftmost column lists a title of a hydrogeologic formation depicted in the cross section on the previous page. The rightmost column presents a quote from a hydrogeological study (see base of table for citation). The quote has been annotated with colored text to highlight how we categorized each layer (i.e., see categories in the center column in the table). Specifically: (i) blue text highlights portions of a quote that provide insights into the degree of consolidation of the formation, (ii) red text highlights portions of a quote that categorize the formation as an aquifer or an aquitard (i.e., higher versus lower permeability in the context of local hydrogeologic formations), and (iii) green text highlights portions of a quote that provide information about the lithology of the formation.

Supplementary Table 75. Hydrostratigraphy details for the Tijuana-San Diego Basin

Formation name	Category	Quote
Quaternary alluvium	Unconsolidated aquifer	"**Groundwater production** comes primarily from **shallow alluvial aquifers** composed of **Holocene-age sediments** in numerous small basins (Fig 13, San Diego Formation of Pliocene age (Kennedy et al., 1975; Moore and Kennedy, 1975, Abbott, 1999) occurs predominantly south of Mission Valley and extends past the United States-Mexican border to locations beneath the city of Tijuana." (Lee & Normark, 2009)
Pliocene (SD Formation)	Clastic sedimentary **aquifer** (consolidated or semi-consolidated rock)	"**Moderately permeable sedimentary formations**, mostly of **Pliocene** and Eocene age, form **a narrow coastal aquifer** extending from coastal reservoirs to the Pacific Ocean (fig. 1)." (Lee & Normark, 2009) | "**San Diego Formation. This unit consists of Pliocene age well-sorted, medium to coarse sand, silty and clayey sand, sandy silt, and sandy clay** (Huntley and others 1996)." Hydrologic Region South Coast, 2004). | "**The water-bearing units in the basin are the San Diego Formation** (SDCWA, 1997) and Quaternary age alluvium." (Hydrologic Region South Coast, 2004a)
Oligocene (Otay Formation)	Clastic sedimentary **aquifer** (consolidated or semi-consolidated rock)	"**groundwater in the Otay Formation** and the Eocene layer as between 25,000 and 45,000 years before present." (Anders et al., 2012). "The Otay Formation **consists predominantly of 35 to 50 m of white, volcanically derived tuffaceous fine sandstone with thin bentonitic interbeds, marked by a basal breccia conglomerate unit**." (Walsh and Demere 1991 quoting Artim and Pinckey (1973))
Eocene sedimentary rock	Clastic sedimentary **aquifer** (consolidated or semi-consolidated rock)	"**Moderately permeable sedimentary formations**, mostly of Pliocene and **Eocene** age, form **a narrow coastal aquifer** extending from coastal reservoirs to the Pacific Ocean (fig. 1)." (Lee & Normark, 2009)
Cretaceous crystalline rock	Endogenous bedrock	"The basin is surrounded by contacts with semi-permeable rocks of the Eocene Poway Group, **impermeable Cretaceous crystalline rock, and impermeable Jurassic to Cretaceous Santiago Peak volcanic rocks**." Hydrologic Region South Coast (2004b)

Lee, H.J., Normark, W.R. (2009). Earth science in the urban ocean: The Southern California continental borderland (Vol. 454). Geological Society of America.

Anders, R., Cronquist, D. A., White, L. E., & Danskin, W. R. (2012, December). Mapping Saline Groundwater in a Coastal Southern California Aquifer System. In *AGU Fall Meeting Abstracts* (Vol. 2012,

pp. H53B-1524). I Walsh, Stephen L., and Thomas A. Demere (1991). Age and stratigraphy of the Sweetwater and Otay formations, San Diego County, California. 131-148.

Hydrologic Region South Coast (2004a). Tijuana Groundwater Basin. California's Groundwater Bulletin 118. Accessed 5/26/2022 via https://water.ca.gov/-/media/DWR-Website/Web-Pages/Programs/Groundwater-Management/Bulletin-118/Files/2003-Basin-Descriptions/9_019_Tijuana.pdf

Hydrologic Region South Coast (2004b). San Diego River Valley Groundwater Basin. California's Groundwater Bulletin 118. Accessed 5/26/2022 via https://water.ca.gov/-/media/DWR-Website/Web-Pages/Programs/Groundwater-Management/Bulletin-118/Files/2003-Basin-Descriptions/9_015_SanDiegoRiverValley.pdf

Walsh, S. L., Demere, T. A. (1991). Age and stratigraphy of the Sweetwater and Otay formations, San Diego County, California (https://archives.datapages.com/data/pac_sepm/086/086001/pdfs/131.htm)

Artim, E. R., Pinckney, C. J. (1973). La Nacion Fault System, San Diego, California. Geological Society of America Bulletin, 84, 1075-1080.

3.73 Upper Santa Ana Basin

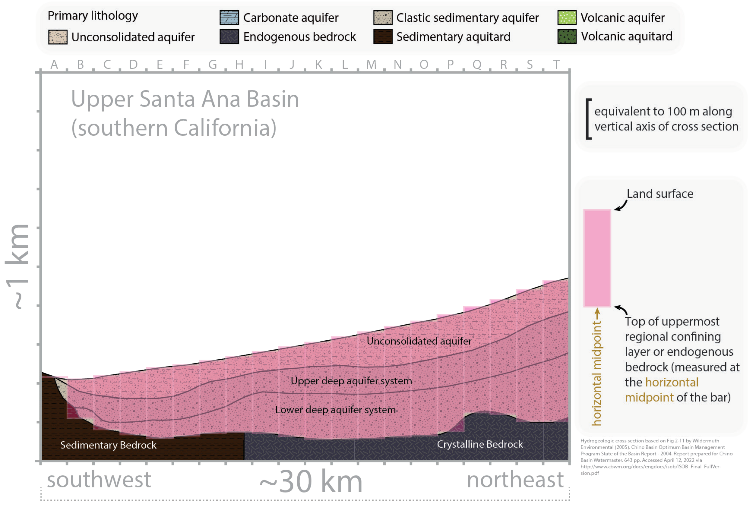

Supplementary Fig. 238. Hydrogeologic cross section. 20 equally spaced transparent pink bars overlie the cross section; each shaded bar depicts the vertical offset from the land surface to the top of the uppermost confining unit or endogenous bedrock.

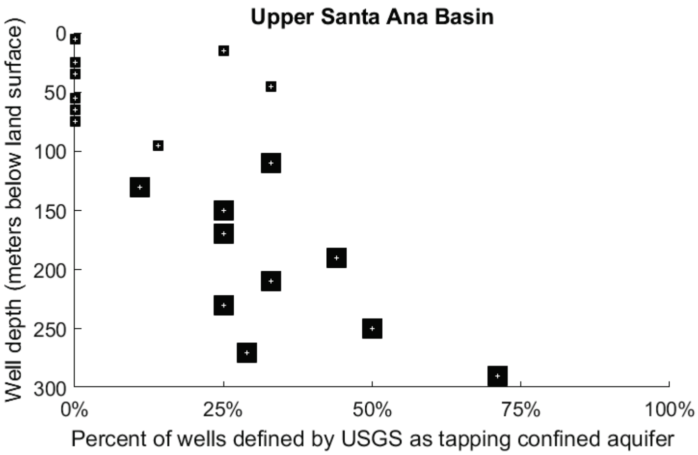

Supplementary Fig. 239. Vertical variations in the prevalence of wells that have been defined as tapping an unconfined or a confined aquifer by the USGS. The smaller squares represent 10 m depth intervals from the land surface to 100 m; the larger squares represent 20 m intervals from 100 m to 300 m below the land surface.

The Upper Santa Ana Basin is located in southern California.

(i) A hydrogeologic cross section presented in Fig. 2-11 by Wildermuth (2005) does not depict a clear continuous confining unit in the Upper Santa Ana Basin.

(ii) We could not identify a depth where most (>80%) wells with the depth range and at deeper depths are defined as tapping a confined aquifer. The three deepest wells in our dataset (depths of 330 m, 338 m, 360 m) are classified as unconfined.

Depth to confined conditions: >360 m (based on (ii) above)

Reference: Wildermuth Environmental (2005). Chino Basin Optimum Basin Management Program State of the Basin Report - 2004. Report prepared for Chino Basin Watermaster. 643 pp. Accessed April 12, 2022 via http://www.cbwm.org/docs/engdocs/isob/ISOB_Final_FullVersion.pdf

The table below presents a series of published quotes (see quotation marks denoting text quoted from another publication, which is cited following the quotation marks with the full reference written in full below the table). The leftmost column lists a title of a hydrogeologic formation depicted in the cross section on the previous page. The rightmost column presents a quote from a hydrogeological study (see base of table for citation). The quote has been annotated with colored text to highlight how we categorized each layer (i.e., see categories in the center column in the table). Specifically: (i) blue text highlights portions of a quote that provide insights into the degree of consolidation of the formation, (ii) red text highlights portions of a quote that categorize the formation as an aquifer or an aquitard (i.e., higher versus lower permeability in the context of local hydrogeologic formations), and (iii) green text highlights portions of a quote that provide information about the lithology of the formation.

Supplementary Table 76. Hydrostratigraphy details for the Upper Santa Ana Basin

Formation name	Category	Quote
Hydrostratigraphic Unit: One	Unconsolidated aquifer	"Layer 1 consists of the upper 200-300 feet of **sediments**, and is generally representative of the **shallow aquifer system** (see Section 2.3.2.3). Layer 1 sediments are typically **coarse-grained (sand and gravel layers)** and, where saturated, **transmit large quantities of groundwater to wells due to high hydraulic conductivities**." (Wildermuth Environmental, 2005).
Hydrostratigraphic Unit: Two	Clastic sedimentary **aquifer** (consolidated or semi-consolidated rock)	"Layer 2 consists of **200-500 feet of sediments** underlying Layer 1, and is representative of the **upper portion of the deep aquifer system** (see Section 2.3.2.3). On the west side of Chino Basin, Layer 2 sediments are primarily **fine-grained (silt and clay layers) with few interbedded sand and gravel layers**." (Wildermuth Environmental, 2005).
Hydrostratigraphic Unit: Three	Clastic sedimentary **aquifer** (consolidated or semi-consolidated rock)	"Layer 3 consists of **100-500 feet of sediments** underlying Layer 2, and is representative of **the lower portion of the deep aquifer system** (see Section 2.3.2.3). Layer 3 sediments are confined to the deepest (central) portions of Chino Basin, and pinch-out toward the basin margins. Layer 3 sediments are **typically coarse-grained (sand and gravel layers),** but **due to their greater age, consolidation, and state of weathering, these sediments have lower permeability than the coarse-grained sediments of Layer 1**." (Wildermuth Environmental, 2005).
Sedimentary Bedrock	Sedimentary **aquitard** (consolidated or semi-consolidated rock)	"The base of the water-bearing sediments in this area occurs within the **sedimentary bedrock** formations that overlie the basement complex, and is recognized as a vertical transition to **very low permeability sediments**." (Wildermuth Environmental, 2005). "Where encountered, **the top of the black clays** are interpreted as the bottom of the aquifer-system." (Wildermuth Environmental, 2005).
Crystalline Bedrock	Endogenous bedrock	"The **basement complex** consists of **deformed and re-crystallized metamorphic rocks that have been invaded and displaced** in places by huge masses of **granitic and related igneous rocks."** (Wildermuth Environmental, 2005).

Wildermuth Environmental (2005). Chino Basin Optimum Basin Management Program State of the Basin Report - 2004. Report prepared for Chino Basin Watermaster. 643 pp. Accessed April 12, 2022 via http://www.cbwm.org/docs/engdocs/isob/ISOB_Final_FullVersion.pdf

3.74 Utah Lake Valley

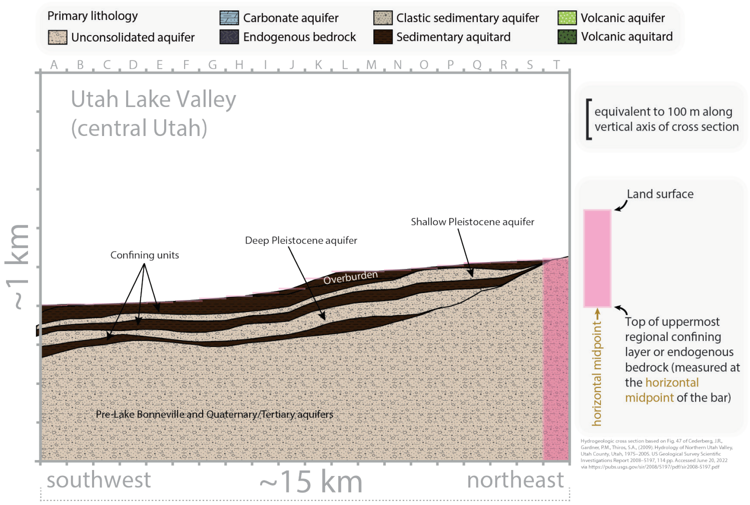

Supplementary Fig. 240. Hydrogeologic cross section. 20 equally spaced transparent pink bars overlie the cross section; each shaded bar depicts the vertical offset from the land surface to the top of the uppermost confining unit or endogenous bedrock.

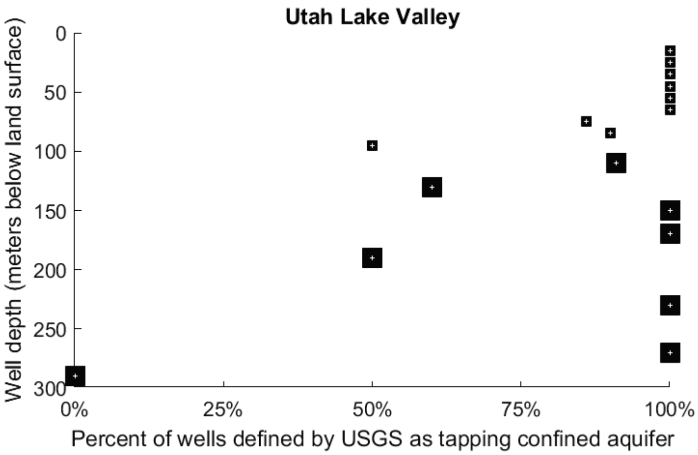

Supplementary Fig. 241. Vertical variations in the prevalence of wells that have been defined as tapping an unconfined or a confined aquifer by the USGS. The smaller squares represent 10 m depth intervals from the land surface to 100 m; the larger squares represent 20 m intervals from 100 m to 300 m below the land surface.

Utah Lake Valley is located in central Utah.

(i) A hydrogeologic cross section presented in Fig. 47 by Cederberg et al. (2009) depicts a series of shallow (i.e., near-surface) confining units.

(ii) We analysed wells within the study area that the USGS has defined as either unconfined or confined. Most (>80%) wells at depths of 10-20 m and at depths exceeding 10 m are defined as tapping a confined aquifer.

Depth to confined conditions: 10-20 m (based on (ii) above)

Reference: Cederberg, J.R., Gardner, P.M., Thiros, S.A., (2009). Hydrology of Northern Utah Valley, Utah County, Utah, 1975–2005. US Geological Survey Scientific Investigations Report 2008–5197, 114 pp. Accessed June 20, 2022 via https://pubs.usgs.gov/sir/2008/5197/pdf/sir2008-5197.pdf

Supplementary Table 77. Hydrostratigraphy details for the Utah Lake Valley

Formation name	Category	Quote
Overburden	Sedimentary **aquitard** (consolidated or semi-consolidated rock)	"all material above the first occurrence of a **clay layer or above the water table in the pre-Lake Bonneville deposits** was classified as **overburden**." (Cederberg et al., 2009)
Confining unit 1	Sedimentary **aquitard** (consolidated or semi-consolidated rock)	"It underlies the **uppermost confining unit (CF1)**, **a blue clay layer** that is the most distinguishable and continuous layer identifiable in drillers' logs. This **confining unit ranges in thickness from 50 to 150 ft**." (Cederberg et al., 2009)
Shallow Pleistocene aquifer	Unconsolidated aquifer	"Hunt and others (1953) originally distinguished **three confined aquifers in the basin-fill deposits of northern Utah Valley** based on their **relative depths and suspected age of sediment deposition** associated with each aquifer. The naming convention applied with depth is: **the shallow confined aquifer in deposits of Pleistocene age (SP aquifer), the deep confined aquifer in deposits of Pleistocene age (DP aquifer), and the confined aquifer in deposits of Quaternary/Tertiary age (QT aquifer)**." (Cederberg et al., 2009). "The **SP aquifer** is the **shallowest confined aquifer in northern Utah Valley** and occurs throughout the middle and lower parts of the basin (fig. 10). The **aquifer** generally consists of **deposits of silt, sand, and gravel** with **a thickness ranging from 10 to 150 ft**." (Cederberg et al., 2009).
Confining unit 2	Sedimentary **aquitard** (consolidated or semi-consolidated rock)	"Underlying the SP aquifer is the next **major confining clay layer (CF2)** that is often described in drillers' logs as **blue or tan clay** with a highly variable **thickness ranging from 10 to 200 ft**." (Cederberg et al., 2009).
Deep Pleistocene aquifer	Unconsolidated aquifer	"The **DP aquifer** is **highly variable in thickness and locally may consist of multiple coarser material layers separated by thin clay and silt layers**. The **aquifer** consists of **mixed sands and gravels close to the mountain front and grades to silty sand near the valley lowlands**." (Cederberg et al., 2009).
Confining unit 3	Sedimentary **aquitard** (consolidated or semi-consolidated rock)	"Often, wells do not fully penetrate the DP aquifer and thicknesses listed on drillers' logs may not represent the full thickness of the aquifer. When penetrated, the **underlying clays** forming the **lowest confining unit (CF3)** are described as **white clays, hardpan, or conglomerate**." (Cederberg et al., 2009).
Pre-Lake Bonneville & Quaternary /Tertiary aquifers	Unconsolidated aquifer	"The **QT aquifer** is the least penetrated, developed, and documented aquifer in the basin-fill deposits of northern Utah Valley. Deposits are often described in drillers' logs as **coarse-grained gravels and sands interbedded with clays and silts** that are not correlated among wells and are assumed to be discontinuous." (Cederberg et al., 2009). "The **unconsolidated sediments west** of the Jordan River and Utah Lake are described in drillers' logs much the same as the **QT aquifer deposits** are described, **consisting of coarse gravels and sands interbedded with clays and silts**." (Cederberg et al., 2009)

Cederberg, J.R., Gardner, P.M., Thiros, S.A. (2009). Hydrology of Northern Utah Valley, Utah County, Utah, 1975–2005: U.S. Geological Survey Scientific Investigations Report 2008–5197, 114 p. Accessed June 4, 2022 via https://pubs.er.usgs.gov/publication/sir20085197

Supplementary Note 4. Multiple rank correlations to account for interrelationships among our two potential explanatory variables

Here we report correlation coefficients between the depth below which modern groundwater becomes scarce and (i) groundwater withdrawals, or (ii) the depth to confined conditions (table below on this page).

Further, because (i) and (ii) may be statistically interrelated (i.e., it is possible that annual groundwater withdrawals are higher in aquifer systems where the depth to confined conditions is deeper), we complete multiple regression on the rank transforms our variables to account for these potential interrelationships and show that the partial regression coefficient between annual groundwater withdrawals and the depth that modern groundwater reaches remains statistically significant (Spearman P-value < 0.05; see table on final page of this Supplementary Note).

Supplementary Table 78. Spearman rank correlation coefficients (ρ) describing statistical relationship between 'the depth below which most* samples contain minimal (<25%) modern water' and two potential explanatory variables. Significant (Spearman P-value < 0.05) correlation coefficients are in **bold**

Calculated depth below which most* samples contain minimal (<25%) modern water	Average groundwater pumping estimated for the year 2015 (mm/year)	Depth to confined conditions (meters below land surface) **
most* = 60%	**ρ = 0.39 (P = 0.0006)**	**ρ = 0.26 (P = 0.0207) *** **
most* = 70%	**ρ = 0.42 (P = 0.0004)**	**ρ = 0.42 (P = 0.0002) *** **
most* = 80%	**ρ = 0.40 (P = 0.0014)**	**ρ = 0.47 (P = 0.0001) *** **

** "most" interpreted as 60% (row 2 of table), 70% (row 3 of table) or 80% (row 4 of table)*

*** see Supplementary Note 3 for details describing how the depth to confined conditions was calculated.*

**** For N=14 of the N=74 study aquifers, we could not identify a range of depths that the aquifer transitioned to confined conditions but, instead, we could confirm that any transition to confined conditions occurs at depths exceeding a given depth (for example, for the Southern High Plains the depth to confined conditions was found to be >270 m). To avoid excluding these aquifers in our rank correlation calculation (as a value of ">" does not readily lend itself to rank correlation), we ascribed the highest rank to these aquifers (i.e., all were given an equal rank that exceeds that of all other aquifers for which we did identify a depth to confined conditions). In an effort to be transparent with respect to the approach we applied to incorporate values of "greater than" in our rank regression, we created the figure on the following page showing the ranked values of "Depth to confined conditions" (see numbers along base of plot)*

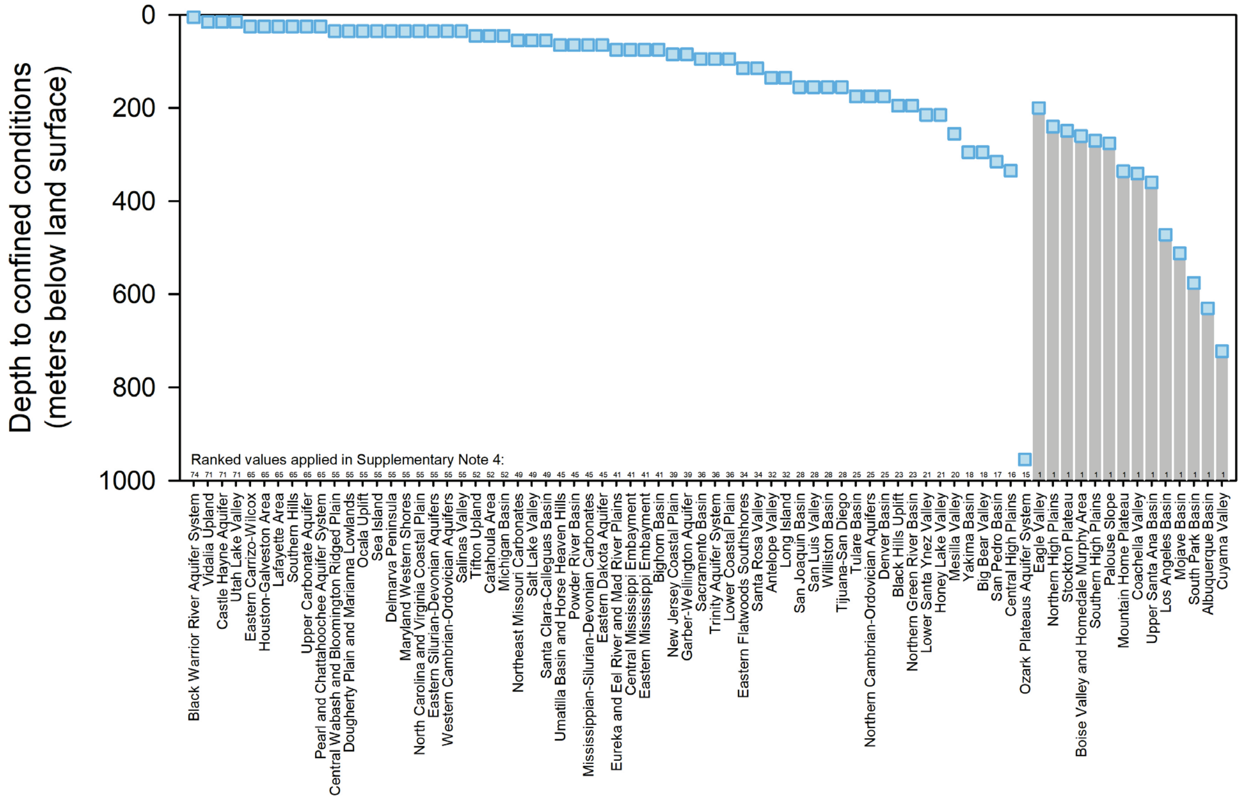

Supplementary Fig. 242. The depth to confined conditions for each of our N=74 study aquifers ranked from lowest (shallowest depth below land surface to confined conditions) to highest (deepest depth below the land surface to confined conditions). Each blue square represents one aquifer system (aquifer system titles shown along the bottom of the plot). Blue squares represent our estimate of the depth below the land surface to confined conditions, calculated on the basis of USGS-defined well conditions, hydrogeologic cross sections in local-scale reports, and quotes within local-scale reports (see Supplementary Note 3). The grey bars on the right side of the plot distinguish aquifer systems for which we could not identify a depth below which the aquifer system was confined; instead, for these aquifers systems, we identified a minimum depth to confined conditions (depicted by the blue square for these aquifer systems). The grey bars highlight that the true depth below which the aquifer system is mostly confined may be at any depth deeper than the blue square (i.e., any depth within the grey bar range). These aquifer systems were given the same (high) value within our rank regression including ‘depth to confined conditions’ (see table on previous page). The relative rank values (from lowest to highest) for the depth to confined conditions are shown as numbers along the bottom of the plot.

Our analyses demonstrate significant (Spearman P-value < 0.05, and in most cases: Spearman P-value < 0.01) rank regressions of the depth below which modern groundwater is scarce versus (A) annual groundwater withdrawals, and (B) depth to confined conditions.

However, because the depth to confined conditions also weakly correlates with annual groundwater withdrawals (Spearman rank correlation coefficient of 0.16, Spearman P-value=0.17), we completed another statistical analysis to account for this interrelationship between our two explanatory variables (i.e., depth to confined conditions, and, annual groundwater withdrawals).

Specifically, we completed multiple regression on the rank transforms of depth to confined conditions and annual groundwater withdrawals. Our resulting partial regression coefficients (β values; table below) demonstrate that annual groundwater withdrawals is significantly (Spearman P-vale < 0.05) correlated with the depth below which modern groundwater becomes scarce across all of our cut-off values (that is, 60%, 70% or 80% values used to determine *the depth below which most samples contain minimal (<25%) modern water*; see second column in table below).

Supplementary Table 79. Multiple regression of the rank transforms coefficients (ρ) describing statistical relationship between 'the depth below which most* samples contain minimal (<25%) modern water' and our two potential explanatory variables: depth to confined conditions, and annual groundwater withdrawals. Significant (P-value < 0.05) partial regression coefficients (β) are in **bold**

Calculated depth below which most* samples contain minimal (<25%) modern water	Average groundwater pumping estimated for the year 2015 (mm/year)	Depth to confined conditions (meters below land surface) **
most* = 60%	**β = 0.34 (P = 0.0027)**	β = 0.18 (P = 0.0968)
most* = 70%	**β = 0.31 (P = 0.0070)**	**β = 0.32 (P = 0.0036)**
most* = 80%	**β = 0.29 (P = 0.0144)**	**β = 0.39 (P = 0.0011)**

** "most" interpreted as 60% (row 2 of table), 70% (row 3 of table) or 80% (row 4 of table)*

Supplementary Note 5. Rank correlations between groundwater withdrawals and the depth below which modern groundwater is scarce if we exclude aquifer systems with shallow depths to confined conditions

We calculated statistical relationships between groundwater withdrawals and the depth below which modern groundwater is scarce in the case where we exclude aquifer systems with shallow confining units. Specifically, we re-ran our correlations after excluding all aquifer systems where the depth to confined conditions was found to be shallower than 50 m, or shallower than 100 m (see Supplementary Tables below). All rank correlations describing the statistical relationship between groundwater withdrawals and the depth below which most groundwater samples contain minimal modern groundwater remain statistically significant (Spearman P-value < 0.05) and have a positive sign (i.e., Spearman ρ values exceeding zero).

Supplementary Table 80. Spearman rank correlation coefficients (ρ) describing statistical relationship between 'the depth below which most* samples contain minimal (<25%) modern water' and two potential explanatory variables *after excluding aquifer systems where the depth to confined conditions was estimated as shallower than 50 m*. Significant (Spearman P-value < 0.05) correlations coefficients are in **bold**

Calculated depth below which most* samples contain minimal (<25%) modern water	Average groundwater pumping estimated for the year 2015 (mm/year)
most* = 60%	**ρ = 0.40 (P = 0.0004)**; sample size of N=51
most* = 70%	**ρ = 0.46 (P = 0.0010)**; sample size of N=48
most* = 80%	**ρ = 0.45 (P = 0.0027)**; sample size of N=41

** "most" interpreted as 60% (row 2 of table), 70% (row 3 of table) or 80% (row 4 of table)*

Supplementary Table 81. Spearman rank correlation coefficients (ρ) describing statistical relationship between 'the depth below which most* samples contain minimal (<25%) modern water' and two potential explanatory variables *after excluding aquifer systems where the depth to confined conditions was estimated as shallower than 100 m*. Significant (Spearman P-value < 0.05) correlations coefficients are in **bold**

Calculated depth below which most* samples contain minimal (<25%) modern water	Average groundwater pumping estimated for the year 2015 (mm/year)
most* = 60%	**ρ = 0.34 (P = 0.0462)**; sample size of N=34
most* = 70%	**ρ = 0.42 (P = 0.0148)**; sample size of N=32
most* = 80%	**ρ = 0.42 (P = 0.0226)**; sample size of N=29

** "most" interpreted as 60% (row 2 of table), 70% (row 3 of table) or 80% (row 4 of table)*

Supplementary Note 6. Potential explanations for deep modern groundwater

To better understand the potential mechanisms by which modern groundwater reaches deep depths (while it is still sufficiently young in its age to be considered 'modern'), we reviewed a number of studies distributed across a subset of our study areas (Supplementary Table 82).

The alternating white/grey shading in the table below delimits generalized categories of features, processes or characteristics (e.g., the first three rows (after the header row) have light grey shading, as all three rows (each row being one study aquifer system) may be receiving artificial recharge via leaking canals or excess irrigation waters—thus, these three rows have the same general category listed in the second column from the left: "Artificial recharge from canals and excess irrigation water may provide additional driving force behind downward-oriented vertical hydraulic gradients, that speed up downward flows of groundwater"). References for quotes in the rightmost column are listed below the table.

Supplementary Table 82. Potential mechanisms by which modern groundwater reaches deep depths while it is sufficiently young in its age to be considered 'modern'. A single aquifer system (and identical quote) may appear more than once in the table if it is applicable to multiple general processes, features or characteristics of the aquifer system that may potentially play a role in determining vertical variations in modern groundwater (i.e., multiple categories in column two)

**Aquifer system**	**General process, feature or characteristic of aquifer system that may play a role in determining vertical variations in modern groundwater**	**Quote from primary literature** (underlines added here to highlight components of the quote relevant to the statement in the second column from the left)
**Salt Lake Valley**	Artificial recharge from canals and excess irrigation water may provide additional driving force behind downward-oriented vertical hydraulic gradients, that speed up downward flows of groundwater	"This age difference is probably affected by the primary recharge area on the west side of the valley being upgradient from two major components of recharge in the area under modern conditions: losses from canals and infiltration from irrigated fields." Thiros et al. (2010)
**Utah Lake Valley**	Artificial recharge from canals and excess irrigation water may provide additional driving force behind downward-oriented vertical hydraulic gradients, that speed up downward flows of groundwater	"The young component of water in this sample (apparent age of 18 years, fig. 45) combined with an enriched δ18O value relative to δD (fig. 41) indicates that recharge to the WU aquifer in this area is from an evaporated source, such as seepage of unconsumed irrigation water or from canals carrying water from Utah Lake." Cederberg et al. (2008)
**Upper Santa Cruz Basin**	Artificial recharge from excess irrigation water and urban runoff may provide additional driving force behind downward-oriented vertical hydraulic gradients, that speed up downward flows of groundwater	"Large changes in tritium in areas of focused infiltration indicated enhanced displacement of older groundwaters by modern recharge…Findings from our study also showed anomalously high CFC concentrations in groundwaters, and non-point source pollution from urban runoff or recharge was identified as the most likely source of elevated CFCs." Carlson et al. (2011)
**San Joaquin Basin**	Artificial recharge from excess irrigation water may provide additional driving force behind downward-oriented vertical hydraulic gradients, that speed up downward flows of groundwater	"Tritium concentrations somewhat higher than in surrounding areas that are found in the southern San Joaquin Valley are also likely the result of intense pumping and recharge via return flow of imported irrigation water." Visser et al. (2014) | "Groundwater classified as young was most often located closer to the valley trough than groundwater classified as old (fig. 10C, table 6), possibly because of infiltration of young irrigation water in the San Joaquin Valley..." Bennett et al. (2010)
**Albuquerque Basin**	Groundwater moves downwards through area(s) of constructed wells, potentially introducing shallower (younger) groundwater into deeper portions of the aquifer near to the well	"Whether enhancement of downward gradients by pumping or by direct movement of water through well bores is the primary mechanism of vertical mixing within the study area, the end result is the presence across broad areas of young, potentially contaminated water at greater depths than would be expected in the absence of deep supply wells…" Bexfield et al. (2012)
**San Joaquin Basin**	Groundwater moves downwards through area(s) of constructed wells, potentially introducing shallower (younger)	"During static conditions, strong vertical hydraulic gradients cause shallow ground water to migrate from the upper part of the well screen to the near bottom of the well screen and into the

Aquifer system	General process, feature or characteristic of aquifer system that may play a role in determining vertical variations in modern groundwater	Quote from primary literature (underlines added here to highlight components of the quote relevant to the statement in the second column from the left)
	groundwater into deeper portions of the aquifer near to the well	deep part of the aquifer, where it was stored until the next pumping cycle." Jurgens et al. (2008)
**Northern High Plains**	Groundwater moves downwards through area(s) of constructed wells, potentially introducing shallower (younger) groundwater into deeper portions of the aquifer near to the well	"Irrigation withdrawals from the confined aquifer result in large downward hydraulic head gradients, creating conditions where water from the unconfined aquifer can move downward to the confined aquifer through boreholes that cross the confining unit" Landon et al. (2010)
**Boise Valley and Homedale Murphy Area**	Groundwater moves downwards through area(s) of constructed wells, potentially introducing shallower (younger) groundwater into deeper portions of the aquifer near to the well	"Tritium is present in shallow aquifers, such as those underlying the New York Canal (Hutchings and Petrich, 2002b). Tritium is virtually non-existent in deeper, regional ground waters, except where well construction has allowed inter-aquifer mixing" Petrich and Urban (2004)
**Houston-Galveston Area**	Groundwater moves downwards through area(s) of constructed wells, potentially introducing shallower (younger) groundwater into deeper portions of the aquifer near to the well	"Additionally, there may be nearby wells with multiple screened intervals in the aquifer(s), allowing for leakage of younger water to the older reservoirs of water, providing a conduit for cross-formational flow and biasing the resulting apparent age." Oden and Truini (2013)
**San Joaquin Basin**	Groundwater moves downwards through area(s) of constructed wells, potentially introducing shallower (younger) groundwater into deeper portions of the aquifer near to the wel	“Furthermore, the groundwater age distribution indicated younger water at depth near the industrial supply well (average of 27 years on Fig. 9) relative to the surrounding groundwater by a factor of as much as 100%. These observations indicated that the industrial supply well acted as a vertical conduit for downward flow and nitrate migration and that the impact to groundwater quality extended more than 1,000 ft (300 m) from the well.” Gailey (2017)*
**Salt Lake Valley**	Groundwater flows downward, potentially crossing boundaries between geologic formations with different hydraulic conductivities (i.e., cross formational flow)	"The primary exception is an area of young water (<20 years old) in the middle of the study area cored by wells 23, 33, and 36; ages from these wells are 5 – 15 years younger than ages from wells located immediately to the east, closer to the mountain front. This local reversal of the regional age gradient is consistent with the X_{min} results, which indicate that wells in this area are drawing in locally recharged water from the shallow unconfined aquifer." Manning and Solomon (2005)
**Eastern Mississippi Embayment**	Groundwater flows downward, potentially crossing boundaries between geologic formations with different hydraulic conductivities (i.e., cross formational flow); in this case, these cross formational flows may be related to discontinuities in a confining unit (‘windows’ in the aquitard)	"The age-date tracer data provide an indication of the intrinsic vulnerability... vulnerability along these flow paths is related to a combination of factors that include the presence of contaminant sources, aquifer conditions (confined or unconfined), proximity to windows in the confining unit, redox conditions, and the degree to which groundwater withdrawals have induced downward flow from shallow parts of the aquifer system." and “…a component of young water is present in the aquifer at most locations along both flow paths, which is consistent with previous studies at Memphis that documented leakage of shallow water into the Memphis aquifer locally where the overlying confining unit is thin of absent.” Kingsbury et al. (2017)
**San Pedro Basin**	Groundwater flows downward, potentially crossing boundaries between geologic formations with different hydraulic conductivities (i.e., cross formational flow)	"There are a few wells in the lower basin-fill aquifer with anomalous geochemistries that may represent areas of potential mixing or recharge from the floodplain aquifer or upper basin-fill aquifer." Hopkins et al. (2014)
**San Luis Valley**	Groundwater flows **upward*, potentially crossing boundaries between geologic formations with different hydraulic conductivities (i.e., cross formational flow)	"Because there is upward migration from the upper and lower confined aquifers into the unconfined aquifer in the ancestral sump area, this unconfined aquifer water has a mixed origin." Mayo et al. (2010)
**Ocala Uplift**	Groundwater flows downward, potentially crossing boundaries between geologic formations with different hydraulic conductivities (i.e., cross formational flow); in this case, these cross formational flows may be related to discontinuities in a confining unit (‘windows’ in the aquitard)	"The Upper Floridan Aquifer and the surficial aquifer system are separated by a discontinuous clay-rich confining unit. A number of localized surface or buried depressions called sinkholes disrupt this layered geologic framework. Breaches in this clay unit result from localized subsidence activity that occurs when the underlying limestone dissolves, causing the collapse of overlying sediments. Many of these breaches in the intermediate confining unit serve as preferential flow paths to the underlying Upper Floridan Aquifer" and "Depth-dependent and monitoring well sampling results indicate that the highly transmissive zone intersecting the PSW below 49 m bls is hydraulically connected

Aquifer system	General process, feature or characteristic of aquifer system that may play a role in determining vertical variations in modern groundwater	Quote from primary literature (underlines added here to highlight components of the quote relevant to the statement in the second column from the left)
		to the surficial aquifer system, probably through sinkholes that breach the confining unit, and results in a mixture of water from the surficial aquifer system and the Upper Floridan Aquifer within the PSW" Landon et al. (2010)
**Black Hills Uplift**	Permeable conduits formed in carbonate (karst) formations within the aquifer system potentially support relatively fast groundwater transport rates	"Anomalies of young groundwater based on chlorofluorocarbons (CFCs), tritium, and electrical conductivity (EC) indicated fast moving, focused flow and thus the likely presence of conduits" Long et al. (2008) | "Tritium was used as a metric to characterize ground-water age in the area near a possible conduit. A straight line fit by linear regression between tritium concentration and distance to the possible conduit shown in figure 1 for the six wells closest to the conduit shows an inverse relation between these two parameters (fig. 15)." Putnam and Long (2007)
**Salt Lake Valley**	Extensive perforated intervals along wells support mixing of younger (shallower) and older (deeper) groundwaters when water is drawn from the well	"Because public-supply wells generally have long open (screened or perforated) intervals (typically 150-500 ft), the samples likely contain mixtures of water with different ages." Thiros et al. (2010)
**Los Angeles Basin**	Extensive perforated intervals along wells support mixing of younger (shallower) and older (deeper) groundwaters when water is drawn from the well	"As noted above, the ages reported here are the mean of a mixed age, which may have a broad distribution, especially since the groundwater is in most cases produced from wells with very long screened intervals." Hudson et al. (2002)
**Tulare Basin**	Extensive perforated intervals along wells support mixing of younger (shallower) and older (deeper) groundwaters when water is drawn from the well	"...wells with long-screens, like many public supply wells, can be screened in both systems and therefore capture varying amounts of pre- and post-1950s groundwater…" Hansen et al. (2018)
**Upper Santa Ana Basin**	Intentional (i.e., 'managed') aquifer recharge sustains and drives downward-oriented vertical hydraulic gradients, which potentially speed up downward-oriented groundwater flow rates	"In the Hemet area, tritium was detected more frequently in shallower wells (70 percent) than in deeper wells (44 percent; fig. 16A). The more frequent detection of tritium in shallower wells suggests recharge by younger water, primarily from above. However, the shallower wells are also located closer to engineered recharge sources than are the deeper wells ($p = 0.04$; Wilcoxon test)." and "In the comparison of ground-water basins, the relative amount of young water was used as an indicator of engineered recharge." Hamlin et al. (2005)
**Los Angeles Basin**	Intentional (i.e., 'managed') aquifer recharge sustains and drives downward-oriented vertical hydraulic gradients, which potentially speed up downward-oriented groundwater flow rates	-"...intense managed aquifer recharge and deep groundwater pumping draws modern groundwater down to greater depths" Visser et al. (2014)
**Salt Lake Valley**	Pumping from wells draws recently recharged (i.e., modern) groundwater down to deeper depths	"Withdrawals from the principal aquifer in the area may have allowed recently recharged water to move downward into the aquifer in the vicinity of these wells." Thiros and Manning (2003)
**Los Angeles Basin**	Pumping from wells draws recently recharged (i.e., modern) groundwater down to deeper depths	"...intense managed aquifer recharge and deep groundwater pumping draws modern groundwater down to greater depths" Visser et al. (2014)
**San Joaquin Basin**	Pumping from wells draws recently recharged (i.e., modern) groundwater down to deeper depths	"The application of irrigation water and pumping from deep parts of the groundwater system have increased the downward movement of shallow groundwater and stratified both the chemistry and age of water in the aquifer" Jurgens et al. (2016)
**Northern High Plains**	Pumping from wells draws recently recharged (i.e., modern) groundwater down to deeper depths	"Irrigation withdrawals from the confined aquifer result in large downward hydraulic head gradients, creating conditions where water from the unconfined aquifer can move downward to the confined aquifer through boreholes that cross the confining unit" Landon et al. (2010)
**Upper Carbonate Aquifer**	Thicker sequences of confining units or relatively low permeability overlying sediment slows downward groundwater flow rates, limiting the depth that modern groundwater reaches	"A comparison of water quality from relatively well protected areas (where the aquifer is overlain by a bedrock confining unit or more than 100 feet of Quaternary-age deposits) and relatively poorly protected areas (where the aquifer is not overlain by a bedrock confining unit or is overlain by less than 100 feet of Quaternary-age deposits) of the Silurian- Devonian and Upper Carbonate aquifers in the study area was performed. Tritium-based ground-water ages were significantly older ($p=0.024$) in relatively well protected areas of the aquifers than in relatively poorly protected areas (fig. 7)" Savoca et al. (1999)

Aquifer system	General process, feature or characteristic of aquifer system that may play a role in determining vertical variations in modern groundwater	Quote from primary literature (underlines added here to highlight components of the quote relevant to the statement in the second column from the left)
**Upper Santa Ana Basin**	Unconfined conditions support more widespread modern groundwater, whereas confining units limit groundwater flow rates and thus the ubiquity of modern groundwater	"Tritium was detected more frequently in the unconfined area (97 percent of sampled wells) than in the confined area (59 percent of sampled wells; fig. 22A), indicating that ground water is younger in the unconfined area than in the confined area, and flows laterally from the unconfined to the confined area" Hamlin et al. (2005)
**Coachella Valley**	Losing rivers serve as a source of modern groundwater recharge, increasing the prevalence of modern groundwater in portions of the aquifer system that are closer to these losing rivers	"Samples collected from the four wells in the Beaumont storage unit had tritium concentrations in excess of 0.2 TU, ranging from 0.5 to 1.9 TU indicating that these wells have received recharge within the past 50 years. Wells 24E2, 28A1, 7E2, and 12K1 are adjacent to stream channels (fig. 36); infiltration along these stream channels probably is the source of the recent recharge to these wells" Rewis et al. (2006).

Bennett, G.L., Fram, M.S., Belitz, K., Jurgens, B.C. (2010). Status and Understanding of Groundwater Quality in the Northern San Joaquin Basin, 2005: California GAMA Priority Basin Project. US Geological Survey Scientific Investigations Report 2010–5175, 96 pp. Accessed May 31, 2022 via https://pubs.usgs.gov/sir/2010/5175/pdf/sir20105175.pdf

Bexfield, L.M., Jurgens, B.C., Crilley, D.M., Christenson, S.C. (2012). Hydrogeology, water chemistry, and transport processes in the zone of contribution of a public-supply well in Albuquerque, New Mexico, 2007–9. US Geological Survey Scientific Investigations Report 2011–5182, 114 pp. Accessed July 5, 2022 via https://pubs.usgs.gov/sir/2011/5182/

Carlson, M. A., Lohse, K. A., McIntosh, J. C., McLain, J. E. (2011). Impacts of urbanization on groundwater quality and recharge in a semi-arid alluvial basin. Journal of Hydrology, 409, 196-211.

Cederberg, J.R., Gardner, P.M., Thiros, S.A. (2008). Hydrology of Northern Utah Valley, Utah County, Utah, 1975–2005. US Geological Survey Scientific Investigations Report 2008–5197, 128 pp. Accessed May 31, 2022 via https://pubs.usgs.gov/sir/2008/5197/pdf/sir2008-5197.pdf

Gailey, R.M. (2017). Inactive supply wells as conduits for flow and contaminant migration: conditions of occurrence and suggestions for management. Hydrogeology Journal, 25, 2163-2183.

Hamlin, S.N., Belitz, K., Johnson, T. (2005). Occurrence and Distribution of Volatile Organic Compounds and Pesticides in Ground Water in Relation to Hydrogeologic Characteristics and Land Use in the Santa Ana Basin, Southern California. US Geological Survey Scientific Investigations Report 2005-5032, 49 pp. Accessed May 31, 2022 via https://pubs.usgs.gov/sir/2005/5032/sir2005-5032.pdf

Hansen, J.A., Jurgens, B.C., Fram, M.S. (2018). Quantifying anthropogenic contributions to century-scale groundwater salinity changes, San Joaquin Valley, California, USA. Science of the Total Environment, 642, 125-136.

Hopkins, C.B., McIntosh, J.C., Eastoe, C., Dickinson, J.E., Meixner, T. (2014). Evaluation of the importance of clay confining units on groundwater flow in alluvial basins using solute and isotope tracers: the case of Middle San Pedro Basin in southeastern Arizona (USA). Hydrogeology Journal, 22, 829-849.

Hudson, G.B., Moran, J.E., Eaton, G.F. (2002). Interpretation of Tritium-3 Helium Groundwater Ages and Associated Dissolved Noble Gas Results from Public Water Supply Wells in the Los Angeles Physiographic Basin. Report to the California State Water Resources Control Board, 59 pp. Accessed May 31, 2022 via https://water.llnl.gov/sites/water/files/2020-09/cas_llnl_la_orange.pdf

Jurgens, B.C., Böhlke, J.K., Kauffman, L.J., Belitz, K., Esser, B.K. (2016). A partial exponential lumped parameter model to evaluate groundwater age distributions and nitrate trends in long-screened wells. Journal of Hydrology, 543, 109-126.

Jurgens, B.C., Burow, K.R., Dalgish, B.A., Shelton, J.L. (2008). Hydrogeology, Water chemistry, and factors affecting the transport of contaminants in the zone of contribution of a public-supply well in Modesto, Eastern San Joaquin Valley, California. US Geological Survey Scientific Investigations Report 2008–5156, 94 pp. Accessed May 31, 2022 via https://pubs.usgs.gov/sir/2008/5156/pdf/sir20085156.pdf

Landon, M. K., Jurgens, B.C., Katz, B.G., Eberts, S.M., Burow, K.R., Crandall, C.A. (2010). Depth-dependent sampling to identify short-circuit pathways to public-supply wells in multiple aquifer settings in the United States. Hydrogeology Journal, 18, 577-593.

Kingsbury, J.A., Barlow, J.R., Jurgens, B.C., McMahon, P.B. Carmichael, J.K. (2017). Fraction of young water as an indicator of aquifer vulnerability along two regional flow paths in the Mississippi embayment aquifer system, southeastern USA. Hydrogeology Journal 25, 1661–1678.

Long, A. J., Sawyer, J. F., Putnam, L. D. (2008). Environmental tracers as indicators of karst conduits in groundwater in South Dakota, USA. Hydrogeology Journal, 16, 263-280.

Manning, A. H., Solomon, D. K. (2005). An integrated environmental tracer approach to characterizing groundwater circulation in a mountain block. Water Resources Research, 41, W12412.

Mayo, A. L., Davey, A., Christiansen, D. (2007). Groundwater flow patterns in the San Luis Valley, Colorado, USA revisited: an evaluation of solute and isotopic data. Hydrogeology Journal, 15, 383-408.

Oden, T.D., Truini, M. (2013). Estimated rates of groundwater recharge to the Chicot, Evangeline and Jasper aquifers by using environmental tracers in Montgomery and adjacent counties, Texas, 2008 and 2011. US Geological Survey Scientific Investigations Report 2013-5024, 61 pp. Accessed July 5, 2022 via https://pubs.usgs.gov/sir/2013/5024/SIR2013-5024.pdf

Petrich, C.R. Urben, S.M. (2004). Characterization of ground water flow in the Lower Boise River Basin, Idaho Depart. of Water Resources, 158 pp. Accessed May 31, 2022 via https://idwr.idaho.gov/wp-content/uploads/sites/2/projects/treasure-valley/TVHP-Characterization.pdf

Putnam, L.D., Long, A.J. (2007). Characterization of Ground-Water Flow and Water Quality for the Madison and Minnelusa Aquifers in Northern Lawrence County, South Dakota. US Geological Survey Scientific Investigations Report 2007–5001, 39 pp. Accessed May 31, 2022 via https://pubs.usgs.gov/sir/2007/5001/pdf/SIR07-5001webSpread.pdf

Rewis, D.L., Christensen, A.H., Matti, J.C., Hevesi, J.A., Nishikawa, T., Martin, P. (2006). Geology, Ground-Water Hydrology, Geochemistry, and Ground-Water Simulation of the Beaumont and Banning Storage Units, San Gorgonio Pass Area, Riverside County, California. US Geological Survey Scientific Investigations Report 2006–5026, 191 pp. Accessed May 31, 2022 via https://pubs.usgs.gov/sir/2006/5026/pdf/sir_2006-5026.pdf

Savoca, M.E., Sadorf, E.M., Akers, K.K. (1999). Ground-water quality in the eastern part of the Silurian-Devonian and Upper Carbonate Aquifers in the eastern Iowa basins, Iowa and Minnesota, 1996. US Geological Survey Water-Resources Investigations Report 98-4224, 35 pp. Accessed May 18, 2022 via https://pubs.usgs.gov/wri/1998/wri984224/pdf/wri98-4224.pdf

Thiros, S.A., Bexfield, L.M., Anning, D.W., Huntington, J.M., eds., (2010). Conceptual understanding and groundwater quality of selected basin-fill aquifers in the Southwestern United States. US Geological Professional Paper 1781, 288 pp. Accessed May 19, 2022 via https://pubs.usgs.gov/pp/1781/

Thiros, S.A., Manning, A.H. (2003). Quality and sources of ground water used for public supply in Salt Lake Valley, Salt Lake County, Utah, 2001. US Geological Survey Water-Resources Investigations Report 2003-4325, 24 pp. Accessed May 31, 2022 via https://pubs.usgs.gov/pp/1781/pdf/pp1781_section2.pdf

Visser, A., Moran, J.E., Singleton, M.J., Esser, B.K. (2014). California GAMA Special Study: Geostatistical analysis of groundwater age and other noble gas derived parameters in California groundwater. Lawrence Livermore National Laboratory Report to the State Water Resources Control Board, 44 pp. Accessed May 31, 2022 via https://www.waterboards.ca.gov/rwqcb5/water_issues/owts/2014_10_swrcb_basinwide_gama_rpt.pdf

(1) Upper Santa Cruz Basin (Tucson Basin): "Findings from our study also showed anomalously high CFC concentrations in groundwaters, and non-point source pollution from urban runoff or recharge was identified as the most likely source of elevated CFCs" quoting Carlson et al. (2011)

(2) Los Angeles Basin: "...intense managed aquifer recharge and deep groundwater pumping draws modern groundwater down to greater depths" quoting Visser et al. (2014)

(3) San Joaquin Basin: "Groundwater classified as young was most often located closer to the valley trough than groundwater classified as old... possibly because of infiltration of young irrigation water in the San Joaquin Valley" quoting Bennett et al. (2010)

(4) Utah Lake Valley: "The young component of water in this sample... combined with an enriched $\delta^{18}O$ value relative to δD... indicates that recharge to the WU aquifer in this area is from an evaporated source, such as seepage of unconsumed irrigation water or from canals carrying water from Utah Lake" quoting Cederberg et al. (2008)

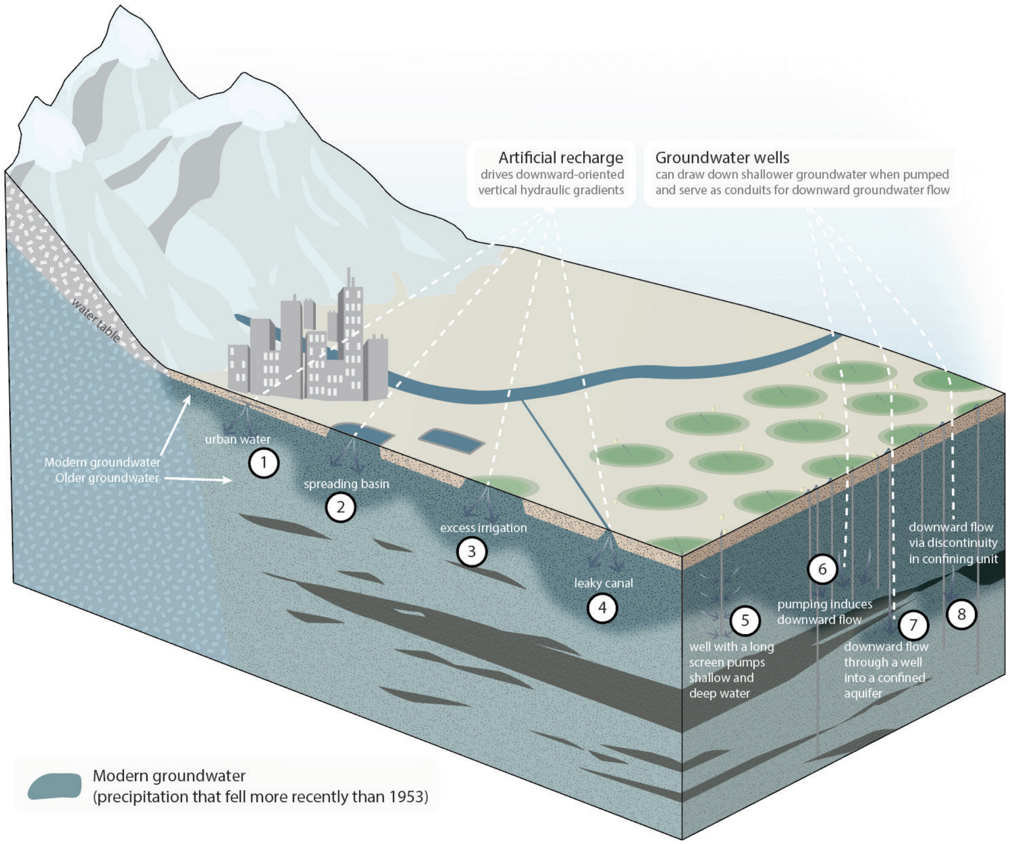

(5) Tulare Basin: "...wells with long-screens, like many public supply wells, can be screened in both systems and therefore capture varying amounts of pre- and post-1950s groundwater..." quoting Hansen et al. (2018)

(6) Salt Lake Valley: "Withdrawals from the principal aquifer in the area may have allowed recently recharged water to move downward into the aquifer in the vicinity of these wells" quoting Thiros and Manning (2003)

(7) Northern High Plains: "Irrigation withdrawals from the confined aquifer result in large downward hydraulic head gradients, creating conditions where water from the unconfined aquifer can move downward to the confined aquifer through boreholes that cross the confining unit" quoting Landon et al. (2010)

(8) Eastern Mississippi Embayment: "...a component of young water is present in the aquifer at most locations along both flow paths, which is consistent with previous studies at Memphis that documented leakage of shallow water into the Memphis aquifer locally where the overlying confining unit is thin of absent" quoting Kingsbury et al. (2017)

Supplementary Fig. 243. Some of the processes that potentially influence vertical distributions of modern groundwater. Each potential recharge-source/process is labelled (numbers 1-8 on figure). The numbered sources/processes correspond to a quote from a publication (see numbers 1-8 surrounding the schematic; full citations are available on the preceding pages).

Supplementary Note 7. Modern groundwater prevalence in wells defined by the USGS as tapping a confined aquifer

To better understand the potential importance of confining units in determining the prevalence of modern water, we subset our groundwater tritium measurements to examine samples collected from confined aquifers. Specifically, we examined n=1,831 groundwater ^3H measurements for water samples collected from a well that the USGS has defined tapping confined conditions ("aqfr_type_cd" defined as "Confined single aquifer" or as "Confined multiple aquifers"). These groundwater ^3H data are presented in panel a below, and their associated modern groundwater fractions are categorized in panel b in the figure below.

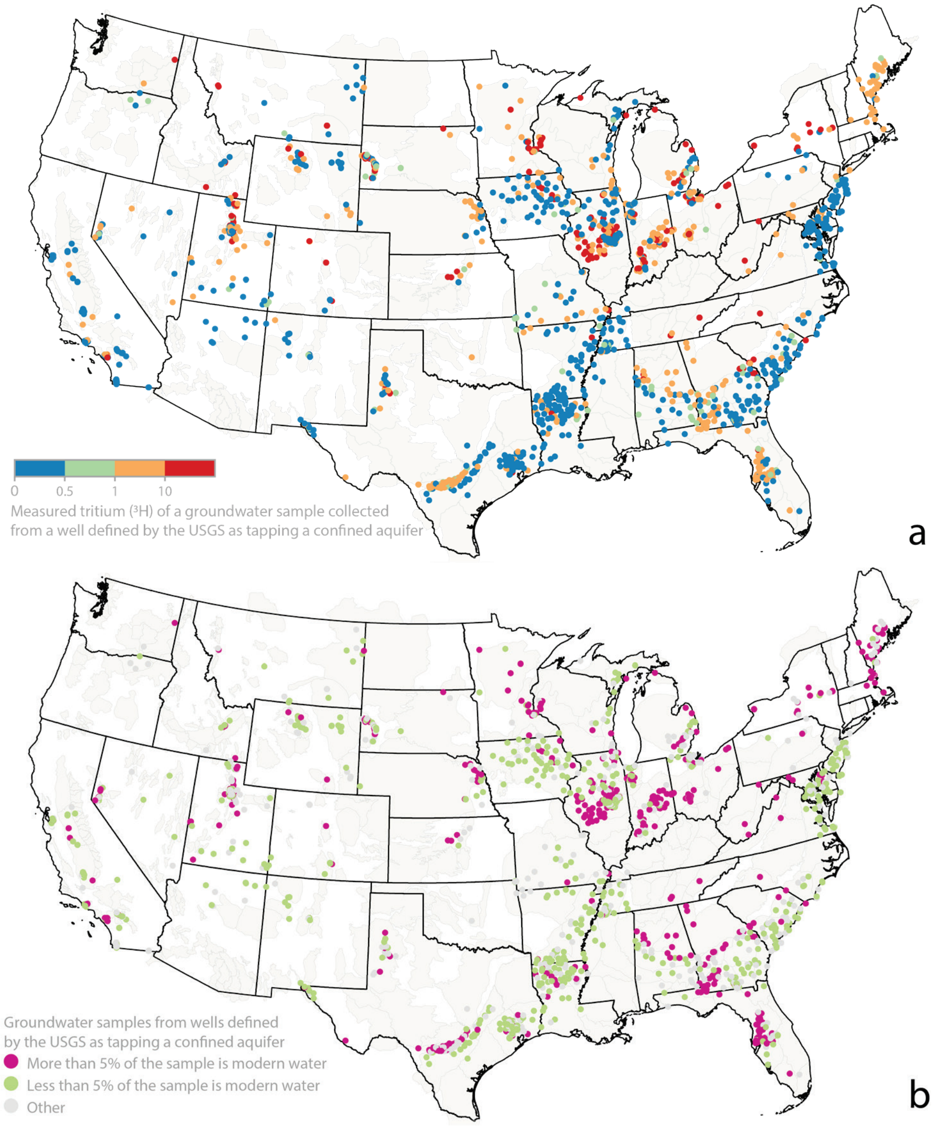

Supplementary Fig. 244. Prevalence of modern groundwater in groundwater samples collected from a well defined by the USGS as tapping a confined aquifer. a) Measured tritium (in tritium units) or well waters collected from a well defined as confined by the USGS. High-^3H water samples collected from confined aquifers are marked by orange and red circles. b) Locations of wells defined as tapping a confined aquifer that have at least 5% modern groundwater (i.e., the *minimum* $F_{Post\text{-}1953}$ exceeds 5% shown as pink circles in panel b). Aquifers by GebreEgziabher et al. (2022) (https://www.nature.com/articles/s41467-022-29678-7) are shown as light grey background polygons, and correspond to the table on the following page.

We grouped the points displayed in panel b in the figure on the previous page according to the aquifer system that they overlie (see aquifer system boundaries basemap in the figure on the previous page); we present the percent of all samples collected from a well tapping a confined aquifer with more than 5% modern water (rightmost column).

Supplementary Table 83. Proportion of samples from confined aquifers with some (more than 5%) modern water

Aquifer system title	Number of well water samples collected from confined aquifer	Percent of all samples collected from a well tapping a confined aquifer that have more than 5% modern water
Dougherty Plain and Marianna Lowlands, Floridan Aquifer System	25	84% of samples from confined aquifers
Springfield Plain, Central Lowland Till Plain	29	83% of samples from confined aquifers
Ocala Uplift, Floridan Aquifer System	63	81% of samples from confined aquifers
Newcastle Till Plain, Central Lowland Till Plain	39	79% of samples from confined aquifers
Western Cambrian-Ordovician Aquifers, Northern Midwest Aquifer System	27	70% of samples from confined aquifers
Volcanic Rift Zone, Eastern Snake River Plain	12	67% of samples from confined aquifers
Balcones Fault Zone, Edwards-Trinity Aquifer System	58	64% of samples from confined aquifers
Los Angeles Basin	27	56% of samples from confined aquifers
Michigan Basin	20	55% of samples from confined aquifers
Salt Lake Valley	81	54% of samples from confined aquifers
Central Wabash and Bloomington Ridged Plain, Central Lowland Till Plain	66	50% of samples from confined aquifers
Mount Vernon Hill County, Central Lowland Till Plain	10	50% of samples from confined aquifers
Black Hills Uplift	53	43% of samples from confined aquifers
Utah Lake Valley	16	38% of samples from confined aquifers
Eastern Silurian-Devonian Aquifers, Northern Midwest Aquifer System	31	35% of samples from confined aquifers
Eastern Carrizo-Wilcox, Carrizo-Wilcox	26	35% of samples from confined aquifers
Northern High Plains, High Plains	38	32% of samples from confined aquifers
Santa Clara-Calleguas Basin	16	31% of samples from confined aquifers
Pearl and Chattahoochee Aquifer System	24	29% of samples from confined aquifers
Black Warrior River Aquifer System	28	29% of samples from confined aquifers
Intermediate Aquifer, Floridan Aquifer System	12	25% of samples from confined aquifers
Southern High Plains, High Plains	21	24% of samples from confined aquifers
Mississippian-Silurian-Devonian Carbonates, Northern Midwest Aquifer System	56	23% of samples from confined aquifers
San Joaquin Basin, California Central Valley	15	20% of samples from confined aquifers
Central Mississippi Embayment, Mississippi Embayment	58	19% of samples from confined aquifers
Vidalia Upland, Floridan Aquifer System	17	18% of samples from confined aquifers
Peedee and Black Creek and Cape Fear Aquifers	23	17% of samples from confined aquifers
Eastern Cambrian-Ordovician Aquifers, Northern Midwest Aquifer System	12	17% of samples from confined aquifers
Bighorn Basin	14	14% of samples from confined aquifers
Eastern Mississippi Embayment, Mississippi Embayment	37	14% of samples from confined aquifers
Delmarva Peninsula, North Atlantic Coastal Plain	24	13% of samples from confined aquifers
Western Carrizo-Wilcox, Carrizo-Wilcox	26	12% of samples from confined aquifers
Tifton Upland, Floridan Aquifer System	64	11% of samples from confined aquifers
Houston-Galveston Area, Gulf Coast Regional Aquifer System	80	10% of samples from confined aquifers
Central Carrizo-Wilcox, Carrizo-Wilcox	11	9% of samples from confined aquifers
Lafayette Area, Gulf Coast Regional Aquifer System	12	8% of samples from confined aquifers
Tulare Basin, California Central Valley	14	7% of samples from confined aquifers
Upper Santa Ana Basin	18	6% of samples from confined aquifers
Tijuana-San Diego	22	5% of samples from confined aquifers
New Jersey Coastal Plain, North Atlantic Coastal Plain	29	3% of samples from confined aquifers
Lower Coastal Plain, Floridan Aquifer System	30	3% of samples from confined aquifers
Ozark Plateaus Aquifer System	30	3% of samples from confined aquifers
Western Mississippi Embayment, Mississippi Embayment	46	2% of samples from confined aquifers
Maryland Western Shores, North Atlantic Coastal Plain	23	0% of samples from confined aquifers
North Carolina and Virginia Coastal Plain, North Atlantic Coastal Plain	22	0% of samples from confined aquifers
Powder River Basin, Northern Great Plains	13	0% of samples from confined aquifers

Supplementary Note 8. Locations of hydrogeologic cross sections

To ensure all of the locally relevant hydrogeologic cross sections depicted within Supplementary Figs. 99-240 intersect with portions of our n=74 study areas, we delineated maps from each publication depicting the location of each cross section. Next, we traced the polyline from these georeferenced maps to develop a geospatial dataset detailing the locations of all of our studied cross sections (see dark blue lines in Supplementary Fig. 245).

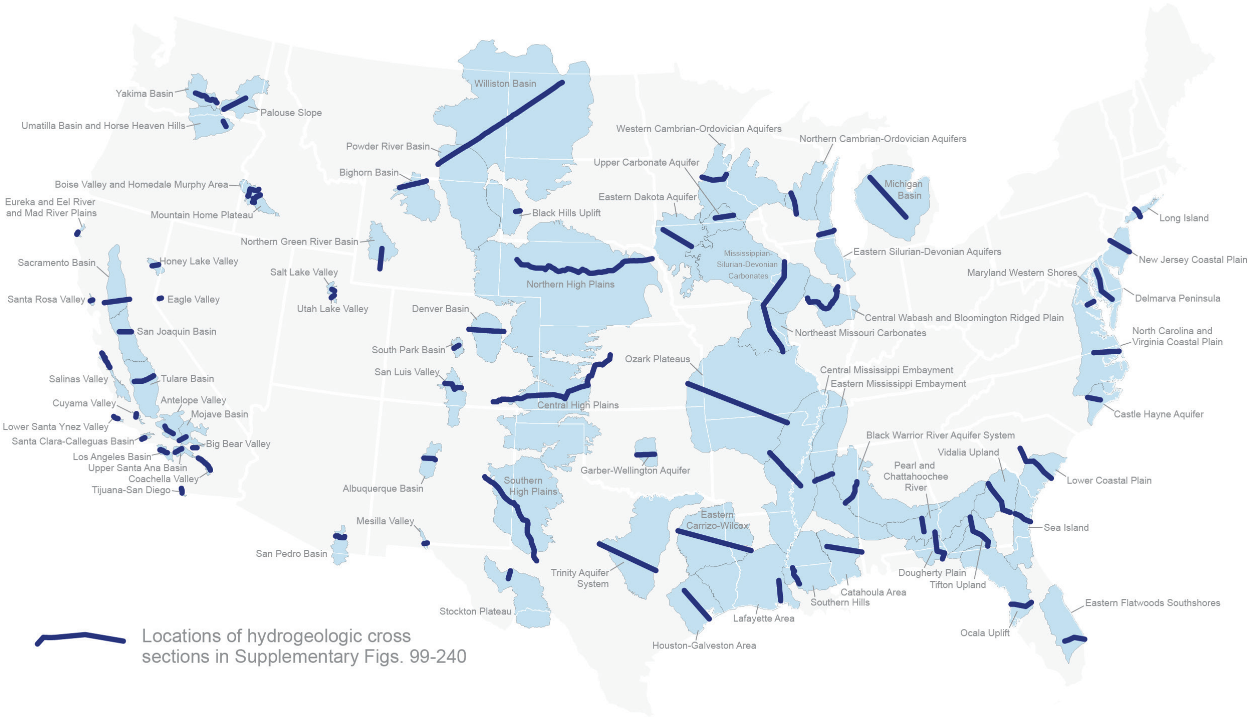

Supplementary Fig. 245. Locations of cross sections overlapping each of our 74 study areas. The light blue polygons are the locations of the n=74 study areas we focus on in this project. Each dark blue line represents the location of a cross section depicted in one of the Supplementary Figures in Supplementary Note 3. The label next to each dark blue line states the specific study area for which a given cross section is relevant to. For the original maps that each georeferenced cross section line captures see citations in Supplementary Notes 3.1-3.74.
